# Asymmetric Three-Component Radical Cascade Reactions Enabled by Synergistic Photoredox/Brønsted Acid Catalysis: Access to α-Amino Acid Derivatives

**DOI:** 10.1021/acscentsci.4c00970

**Published:** 2024-08-16

**Authors:** Chao Che, Yi-Nan Lu, Ting Fang, Guang-Jin Zhen, Xiaotian Qi, Chun-Jiang Wang

**Affiliations:** † College of Chemistry and Molecular Sciences, Wuhan University, Wuhan 430072, China; ‡ State Key Laboratory of Elemento-organic Chemistry, Nankai University, Tianjin 300071, China

## Abstract

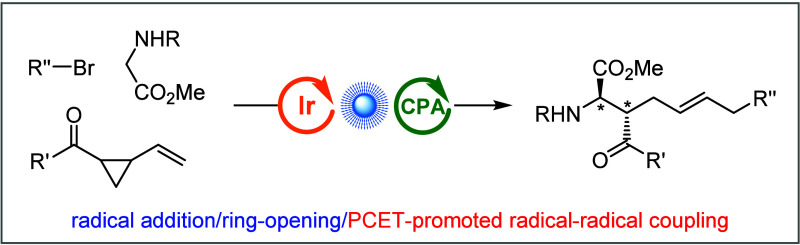

Multicomponent reactions (MCRs), highly sought-after methods to produce atom-, step-, and energy-economic organic syntheses, have been developed extensively. However, catalytic asymmetric MCRs, especially those involving radical species, remain largely unexplored owing to the difficulty in stereoselectively regulating the extraordinarily high reactivity of open-shell radical species. Herein, we report a conceptually novel catalytic asymmetric three-component radical cascade reaction of readily accessible glycine esters, α-bromo carbonyl compounds and 2-vinylcyclopropyl ketones via synergistic photoredox/Brønsted acid catalysis, in which three sequential C–C (σ/π/σ) bond-forming events occurred through a radical addition/ring-opening/radical–radical coupling protocol, affording an array of valuable enantioenriched unnatural α-amino acid derivatives bearing two contiguous stereogenic centers and an alkene moiety in moderate to good yield with high diastereoselectivity, excellent enantioselectivity and good *E*-dominated geometry under mild reaction conditions. The radical relay cascade process, especially a unique proton-coupled electron transfer (PCET)-promoted radical–radical coupling, is supported by mechanistic investigations and quantum mechanics calculations and should garner broad interest and further inspire the development of asymmetric multicomponent radical reactions.

## Abstract 2

Via synergistic photoredox/CPA catalysis, a novel three-component radical addition/ring-opening/PCET-promoted radical−radical coupling reaction enables quick access to chiral α-amino acid derivatives.

Vinyl cyclopropanes (VCPs), which are known as crucial chemical building blocks, have drawn particular interest in organic synthesis due to their exceptional reactivity and diverse reaction modes.^1^ VCPs are employed as C-3 or C-5 synthons, depending on whether vinyl substituents are engaged in the reaction. The current cycloaddition and 1,5-difunctionalization processes enabled by C–C bond cleavage of VCPs to produce different skeletons efficiently via the two-electron transfer pathway have been well investigated.^2^ Vinyl cyclopropanes are readily activated by various transition metal catalysts, including Pd, Ir, Rh, Ru, Ni, Fe, etc., to produce the corresponding zwitterionic metal-π-allyl species bearing a pendent ***C***-nucleophile, which are the key intermediates in the ensuing annulation or 1,5-difunctionalization scenario (Scheme 1a). Despite the elegant advances realized, there is a paucity of diversity of substrate scopes and the reaction patterns in the ionic (two-electron) pathway. Alternatively, in the course of mechanistic studies, VCPs were frequently utilized as radical clock reagents for the identification of the potential transient radical intermediates.^3^ The terminal olefin of VCPs can readily trap the external radical via radical addition, which further induce the subsequent C–C bond homolytic cleavage of the cyclopropane ring, driven by the release of the high ring-strain energy (28 kcal/mol),^1i^ to relocate the single electron with the concomitant formation of an internal olefin moiety. However, to our knowledge, the synthetic utilities of VCPs via single-electron transfer in asymmetric multicomponent reactions (MCRs)^4^ remain underdeveloped,^5^ probably due to the formidable challenge in stereoselectively regulating the extraordinarily high reactivity of open-shell radical species in the asymmetric radical cascade reaction.

Optically active nonproteinogenic α-amino acids (α-AAs) have been widely utilized in the design of biologically important unnatural peptides and drug compounds.^6^ The development of novel and efficient methodologies for catalytic asymmetric synthesis of unnatural α-AAs is of great importance in organic synthesis and pharmaceutical sciences.^7^ However, radical three-component reactions for the catalytic asymmetric synthesis of α-AAs remain an unexplored endeavor. In continuation of our research interest in catalytic asymmetric synthesis of enantioenriched α-AAs via synergistic catalysis,^8^ we wondered whether VCPs could serve as a synthetic relay handle in three-component radical-relay reactions to realize modular synthesis of chiral α-AAs with structural diversity. More specifically, an initially formed radical from α-bromo carbonyl compound reacts with VCPs followed by ring rupture to generate an α-carbonyl radical, which can be sequentially captured by an α-amino radical or a cationic iminium generated *in situ* from *N*-substituted glycine esters via synergistic photoredox/Brønsted acid catalysis to afford densely functionalized chiral α-AAs (Scheme 1b). Nevertheless, there are several challenges in this radical three-component reaction design: (1) the competition radical coupling or radical additions of the initially formed radical and the relay radical to the in situ formed α-amino radical or cationic iminium species; (2) the undesired radical–radical homocoupling or cross-coupling side products caused by diffusion-controlled radical reactions; (3) multiple diastereoselectivity, enantioselectivity and *E*/*Z* geometry controls associated with the designed radical relay cascade reaction.

**Scheme 1:**
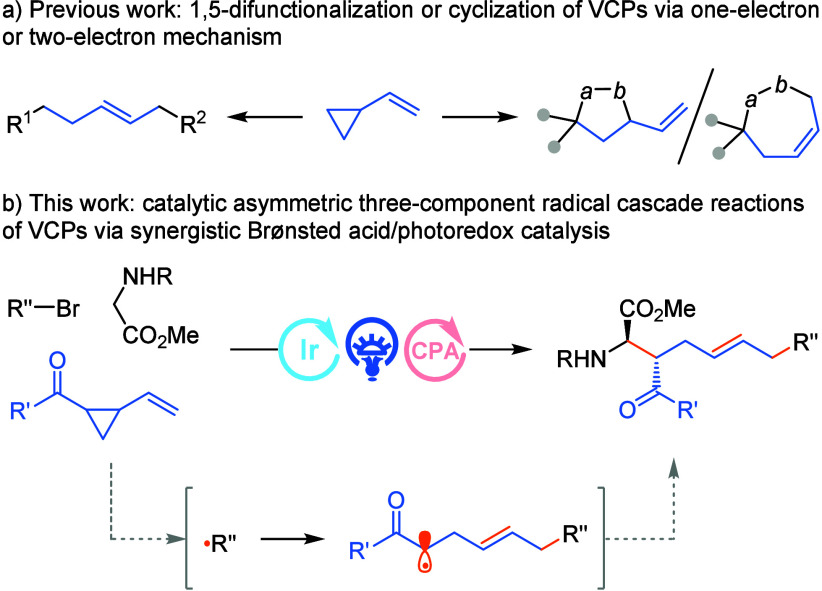
Reaction Modes of Vinyl Cyclopropanes (VCPs) and Catalytic Asymmetric Three-Component Radical Cascade Reactions Enabled by Synergistic Photoredox/Brønsted Acid Catalysis

Herein, we reported the development of stereoselective assembly of enantioenriched unnatural α-amino acid derivatives with readily available starting materials via a catalytic asymmetric three-component radical cascade reaction enabled by synergistic photoredox/Brønsted acid catalysis.^9^ The densely functionalized chiral α-amino acid derivatives were obtained in good yields with high diastereoselectivity, excellent enantioselectivity, and high *E*-dominated internal olefin moiety under mild reaction conditions. The stereochemical outcome of the three-component radical cascade reaction is remarkable in view of three sequential C–C (σ/π/σ) bond-forming events through radical addition/ring-opening/radical–radical coupling protocol. Density functional theory (DFT) studies revealed that a unique proton-coupled electron transfer (PCET)-promoted radical–radical coupling was the enantio-determining step.

Based on our design, optimization studies were initially performed with *N*-phenyl glycine methyl ester (**1a**), *trans*-phenyl(2-vinylcyclopropyl)methanone (**2a**), and commercially available ethyl bromodifluoroacetate (**3a**) as the model substrates in the presence of a catalytic amount of photosensitizer and chiral Brønsted acid^10^ under irradiation of light. Exhaustive screening of the reaction parameters revealed that the three-component radical cascade reaction is best performed with 2 mol % of Ir(dF(CF_3_)ppy)_2_(dtbbpy)PF_6_ (**Ir–I**) as a photosensitizer, 10 mol % of chiral phosphoric acid (*R*)-**C1**, NaHCO_3_ as a base, and 3 Å molecular sieves (MSs) in the mixed solvent of 1,4-dioxane/MeCN (1:1 volume ratio) under irradiation with a 7 W blue LED at 20 °C for 12 h (see Supporting Information for the comprehensive survey of reaction conditions). The α-amino acid ester **4a** incorporating two vicinal stereogenic centers and an internal olefin moiety was obtained in 65% yield with 8:1 *E*/*Z* geometry and excellent diastereo-/enantioselectivity (>20:1 dr, 92% ee) (Table 1, entry 1). Deviations from the optimal reaction conditions with other chiral phosphoric acids did not improve the yield and stereoselective control of the chiral product (entries 2–6). Employing other Ir- or Ru-based or organic photoredox catalysts (**Ir–II** (Ir[dFppy]_2_(dtbbpy)PF_6_), **Ir–III** (Ir[dF(CF_3_)ppy]_2_(bpy)PF_6_), **Ir–IV** (Ir[dF(CF_3_)ppy]_2_(4,4′-dCF_3_-bpy)PF_6_), **Ir–V** (Ir[dF(CF_3_)ppy]_2_(5,5′-dCF_3_-bpy)PF_6_), **Ir–VI** (Ir(ppy)_3_), **Ru–I** (Ru(bpy)_3_]Cl_2_·6H_2_O), Eosin Y, Rose Bengal, and Mes-Acr^+^) did not lead to the effective formation of product with higher catalytic efficiency and better diastereo-/enantioselectivity as well as *E*/*Z* geometry control (entries 7–13). Considering that 1,4-dioxane provides superior yields and acetonitrile provides better stereoselectivity control, a mixed solvent of acetonitrile and 1,4-dioxane (1:1 volume ratio) was selected as the optimal choice (entries 14 and 15). Control experiments revealed the necessity of photocatalysis since no reaction occurred in the absence of either visible-light or iridium photocatalyst (entries 16 and 17). Without (*R*)-**CPA-1** as the chiral Brønsted acid, the three-component radical cascade reaction proceeded smoothly to generate the corresponding racemic **4a** in 39% yield with a sharply diminished diastereoselectivity (4:1 dr) (entry 18), which demonstrated a remarkably chiral-catalyst-accelerated effect to efficiently suppress the background reaction. In the absence of base, the yield of the adduct dropped significantly, albeit with similar stereoselectivity control (entry 19). This synergistic photoredox and chiral Brønsted acid-catalyzed asymmetric three-component radical relay protocol could be readily performed on a 2 mmol scale without significant erosion in both reactivity and *E*/*Z* geometry and diastereo-/enantioselectivity control (entry 20). When the reaction was interrupted under the standard reaction conditions within 4 h, the recovered **2a** was observed as racemate, which demonstrates no kinetic resolution of racemic *trans*-**2a** in this cascade transformation.

**Table 1:**
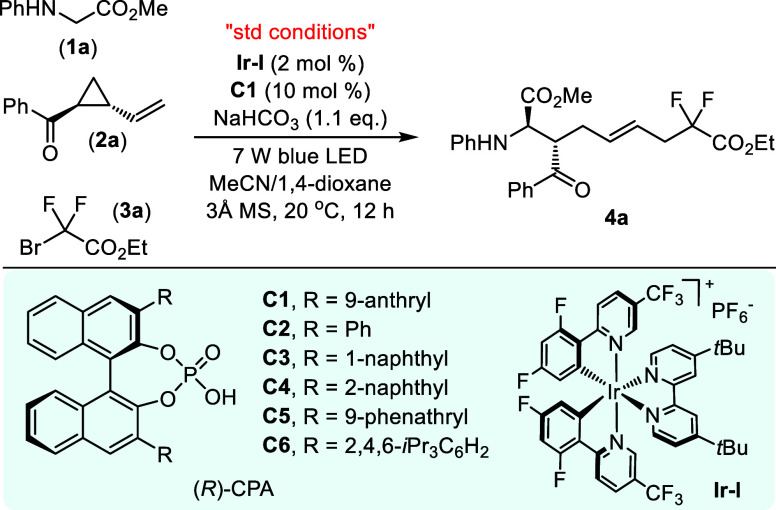
Optimization of Reaction Conditions^a^

entry	variation from standard conditions	yield (%)^b^	*E*/*Z*^c^	dr^d^	ee (%)^e^
1	none	65	8:1	>20:1	92
2	(*R*)-**C2** instead of (*R*)-**C1**	37	6:1	8:1	50
3	(*S*)-**C3** instead of (*R*)-**C1**	49	6:1	7:1	–68
4	(*S*)-**C4** instead of (*R*)-**C1**	51	7:1	6:1	–64
5	(*S*)-**C5** instead of (*R*)-**C1**	43	6:1	11:1	–68
6	(*S*)-**C6** instead of (*R*)-**C1**	51	8:1	>20:1	–89
7	**Ir–II** instead of **Ir–I**	50	6:1	>20:1	88
8	**Ir–III** instead of **Ir–I**	53	7:1	12:1	74
9	**Ir–IV** instead of **Ir–I**	35	6:1	>20:1	86
10	**Ir–V** instead of **Ir–I**	trace			
11	**Ir–VI** instead of **Ir–I**	29	5:1	>20:1	90
12	**Ru–I** instead of **Ir–I**	trace			
13	Eosin Y, Rose Bengal, or Mes-Acr^+^ instead of **Ir–I**	trace			
14	1,4-dioxane only	52	5:1	>20:1	88
15	MeCN only	44	8:1	>20:1	93
16	without PC	N.R.			
17	without *hv*	N.R.			
18	without (*R*)-**C1**	39	6:1	4:1	0
19	without base	16	6:1	>20:1	88
20^f^	none	62	7:1	>20:1	92

a Conditions: **1a** (0.2 mmol), **2a** (0.3 mmol), **3a** (0.8 mmol), **PC-1** (2 mol %), (*R*)-**C1** (10 mol %), NaHCO_3_ (1.1 equiv) in 2 mL of 1,4-dioxane/MeCN (1:1) at 20 °C under irradiation of 7 W blue LED.

b Isolated yield.

c *E*/*Z* was determined by ^19^F NMR.

d To determine the diastereoselectivity of **4a**, the saturated compound **4a′** was achieved via Pd/C-catalzyed hydrogenation. The dr value of **4a** is equal to that of **4a′**, which was determined by ^1^H NMR.

e ee was determined by HPLC analysis.

f Reaction was carried out with 2 mmol of **1a**.

With the optimal reaction conditions in hand, we first examined the substrate scope of this visible-light-enabled asymmetric three-component radical cascade reaction by treating *trans*-phenyl(2-vinylcyclopropyl)methanone (**2a**) and bromodifluoroacetate (**3a**) with different *N*-aryl glycinate esters. The representative results are summarized in Table 2. Various *N*-aryl glycinate methyl esters bearing electron-deficient (*para*-Cl, *meta*-Cl, or *ortho*-Cl), electron-neutral, and electron-rich substituents (*para*-MeO, *para*-Me, *meta*-Me, or *ortho*-Me) on the aromatic ring in a different substituent manner were all compatible with this synergistic Brønsted acid/photoredox catalytic system, affording the desired biologically important *N*-aryl functionalized fluorine-containing α-amino acid derivatives^11^**4b**–**4h** are in moderate yields (31–65%) with good *E*/*Z* geometry (7:1 to 14:1), exclusive diastereoselectivity (>20:1 dr), and high enantioselectivity (87–94% ee). Relatively lower yields were observed with *N*-aryl glycinate esters bearing *ortho*-substituents on the phenyl ring (**4e** and **4h**) probably due to the disfavored steric hindrance. In addition, 3,5-dimethyl-substituted glycinate methyl ester was well tolerated in this radical cascade reaction, affording the corresponding product **4i** in 50% yield with 13:1 *E*/*Z* geometry and excellent diastereo-/enantioselectivity (>20:1 dr, 92% ee). Next, a range of 2-vinylcyclopropyl ketones was studied in this radical relay reaction with *N*-phenyl glycine methyl ester (**1a**) and bromodifluoroacetate (**3a**). As shown in Table 2, the variations in electronic properties on the aryl ketone have little effect on the reactivity and *E*/*Z* geometry and diastereo-/enantioselectivity control, leading to the corresponding products (**4j**, **4k**, **4m**, and **4n**) in moderate yields (40–56%) with synthetically useful *E*/*Z* geometry (7:1 to 9:1), exclusive diastereoselectivity (>20:1 dr), and good enantioselectivity (88–92% ee). Sterically hindered *ortho*-methyl substituted aryl ketone could also be tolerated in this cascade reaction, affording the desired product **4l** with good *E*/*Z* geometry diastereoselectivity control, albeit with significantly reduced enantioselectivity. Notably, 2-thienyl substituted heteroaryl ketone was a viable substrate in this catalytic system, giving rise to the corresponding product **4o** in 55% yield, 9:1 *E*/*Z* geometry, and 92% ee without erosion of diastereoselectivity. In addition to VCPs containing aryl ketone, cyclohexyl ketone derived VCP substrate performed well in this three-component radical cascade reaction, affording the expected product **4p** in a 51% yield with 7:1 *E*/*Z* geometry, exclusive diastereoselectivity, and 81% ee. The absolute configuration of product **4o** was unambiguously determined to be (7*S*,8*R*,*E*) through X-ray analysis (CCDC 2268647). Having demonstrated the substrate scope of *N*-aryl α-amino acid esters and 2-vinylcyclopropyl ketones, we turned our attention to exploring the scope with respect to C-radical precursors **3**. A diverse range of α-bromocarbonyl compounds (such as α-bromo esters, α-bromo amides, and α-bromo ketones) are suitable for this asymmetric three-component radical cascade reaction. Methyl bromodifluoroacetate reacted well as a difluorinating reagent, affording the desired product **4q** in 54% yield with 8:1 *E*/*Z* and 90% ee. α-Bromoketone was also well tolerated in this catalytic system, and the target product **4r** was obtained in moderate yield with 10:1 *E*/*Z* and 93% ee without erosion of diastereoselectivity. Also, two different difluoro amide derivatives could be efficiently converted into the desired products **4s** and **4t** in moderate yields with high *E*/*Z* geometry and excellent diastereo-/enantioselectivity. Significantly, industrial dibromodifluoromethane (Freon 12B2) performed well in this radical delay reaction, delivering the desired product **4u** bearing a bromodifluororomethyl substituent in 46% yield with 8:1 *E*/*Z* and 92% ee. Further studies revealed that the radical cascade reaction could be readily extended to the commonly used electrophilic primary, secondary, and tertiary alkyl radical precursors, such as diethyl 2-bromo-2-fluoro-malonate (**3v**), diethyl 2-bromo-2-methyl-malonate (**3w**), diethyl bromo-malonate (**3x**), and methyl bromoacetate (**3y**).

**Table 2:**
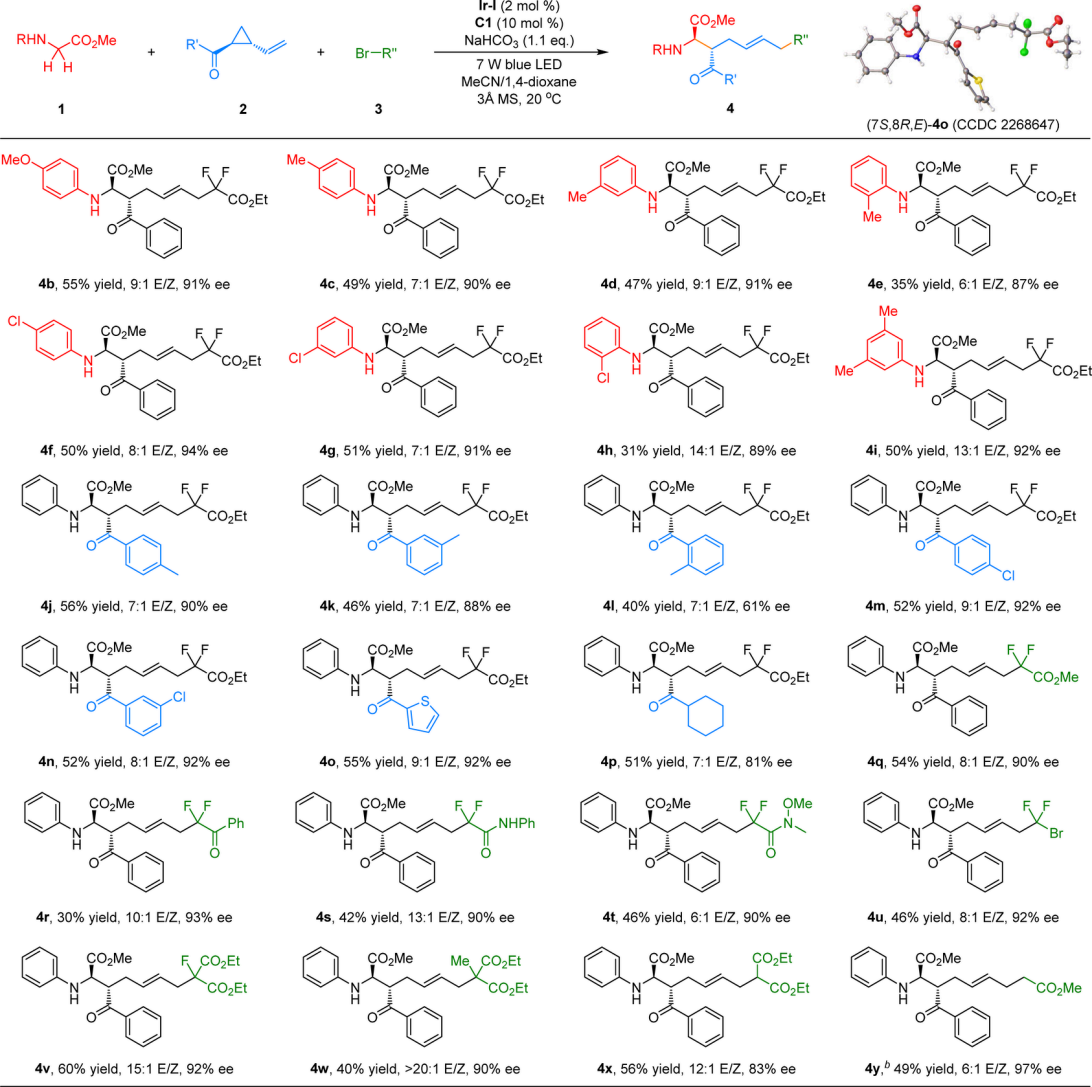
Substrate Scope with Respect to *N*-Aryl α-Amino Acid Esters, 2-Vinylcyclopropyl Ketones, and Alkyl Bromides^a^

a All reactions were carried out with 0.40 mmol of **1**, 0.60 mmol of **2**, 0.80 mmol of **3**, 0.44 mmol of NaHCO_3_, and 3 Å MS (400 mg) in degassed 4 mL of MeCN/1,4-dioxane (1:1) at 20 °C for 12 h. Yields refer to the isolated products after chromatography. *E*/*Z* was determined by ^19^F NMR. Ee was determined by HPLC analysis.

b Reaction was carried out in degassed fluorobenzene.

Encouraged by the promising results of fluorinated α-amino acid esters **4** incorporating two adjacent tertiary/tertiary stereogenic centers with VCPs **2** containing two vicinal substituents on the cyclopropyl ring, we wondered whether the current visible-light-induced asymmetric three-component radical cascade reaction could be robust enough to construct a more challenging α-amino acid analogy bearing a β-quaternary stereogenic center.^12^ Very gratifyingly, when (1-methyl-2-vinylcyclopropyl)-(phenyl) methanone **5** bearing two vicinal quaternary/tertiary carbon centers was examined as the reaction partner under the optimized reaction conditions, the radical relay reaction occurred smoothly followed by a Pd/C-mediated hydrogenation, affording the corresponding product **6** bearing a unique quaternary stereogenic center in 48% yield with 5.5:1 dr and 85% ee (Scheme 2, upside). Consistent with the behavior of racemic *trans*-**2a**, no kinetic resolution of racemic **5** was observed in this cascade transformation. To further verify the generality of this asymmetric radical relay protocol, several more complex bromodifluoroacetate substrates derived from the natural products *L*-menthol, *L*-isoborneol, and cholesterol were subjected to this three-component radical cascade reaction under the standard reaction conditions with (*R*)-**C1** as the chiral phosphoric acid followed by C=C bond reduction, delivering the corresponding fluorinated α-amino acid ester derivatives **10**–**12**, respectively, in good yields with high diastereoselectivities (Scheme 2, downside). When switching chiral CPA (*R*)-**C1** with (*S*)-**C1**, as expected, the corresponding three complementary diastereomers (**10′**–**12′**) could be readily isolated with similar results via the same protocol. These results underscore the broad generality of this novel protocol and the insensitivity of the first step of radical addition to 2-vinylcyclopropyl ketones.

**Scheme 2:**
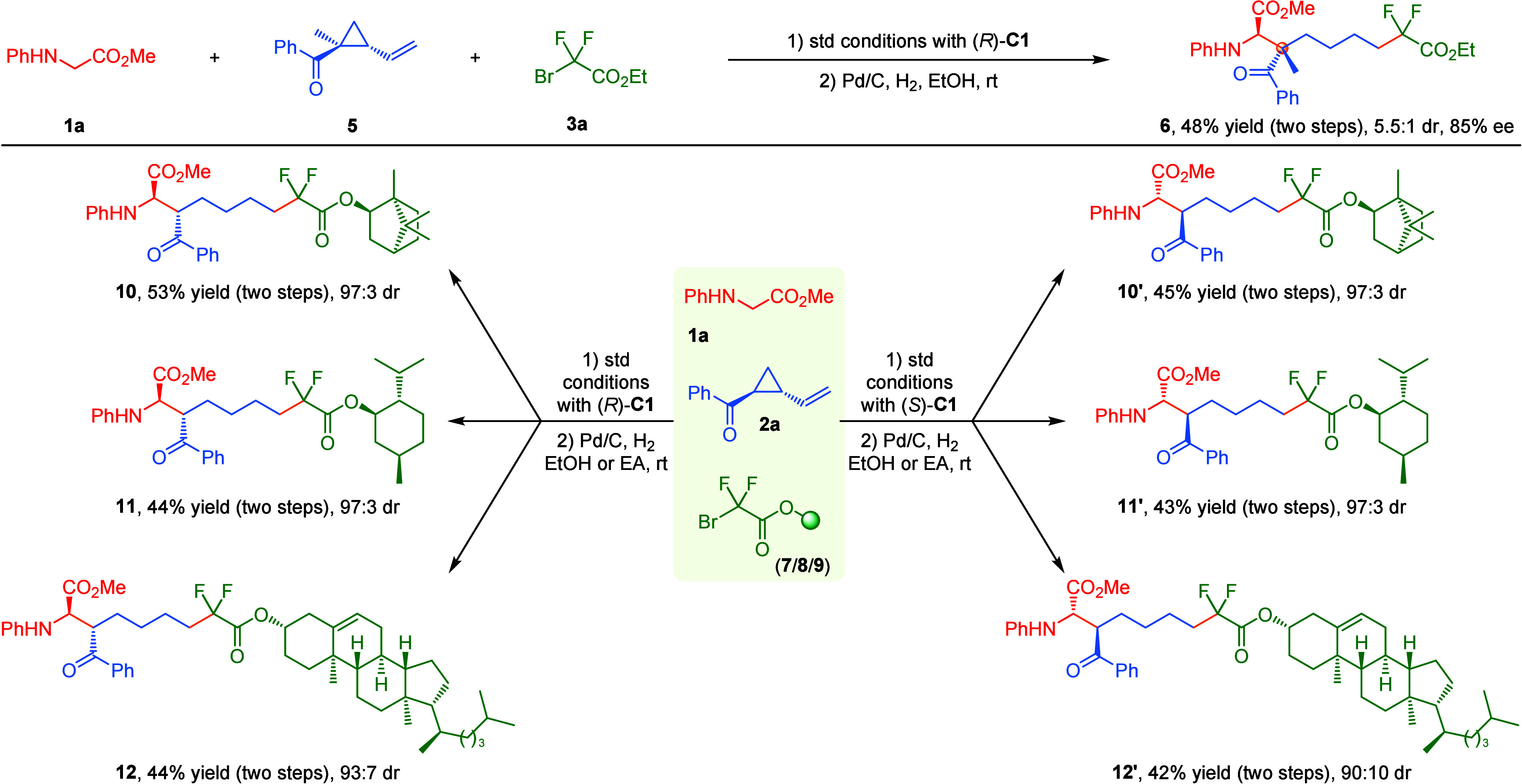
Asymmetric Synthesis of Fluorinated α-Amino Acid Esters Incorporating Quaternary/Tertiary Stereogenic Centers or Core-Structures of Natural Products

The synthetic utility of this asymmetric three-component radical cascade reaction is demonstrated in Scheme 3. The internal C=C double bond of compound **4a** could be readily reduced via Pd/C-catalyzed hydrogenation to afford the saturated compound **13** in 85% yield without loss of the diastereo-/enantioselectivity. On the other hand, I_2_-mediated intramolecular cyclization of compound **4a** provided the enantioenriched pyrrole derivative **14** in 75% yield with 7:1 dr and 92% ee. The carbonyl group of ketone **4a** can be reduced by BH_3_·Me_2_S in the presence of TiCl_4_ to give the corresponding chiral alcohol **15** in high yield with an exclusive diastereoselective manner. Upon treatment with silica gel alkalized by Et_3_N, lactonization of compound **15** occurred smoothly to access the enantioenriched α-butyrolactone-**16** bearing three contiguous stereogenic centers in good yield with maintained diastereo-/enantioselectivity. Cleavage of the *p*-methoxyphenyl (PMP) group in compound **4b** could be realized with CAN (cerium(IV) ammonium nitrate) to generate the corresponding compound **17** in good yield without any loss of *E*/*Z* geometry and diastereo-/enantioselectivity.

**Scheme 3:**
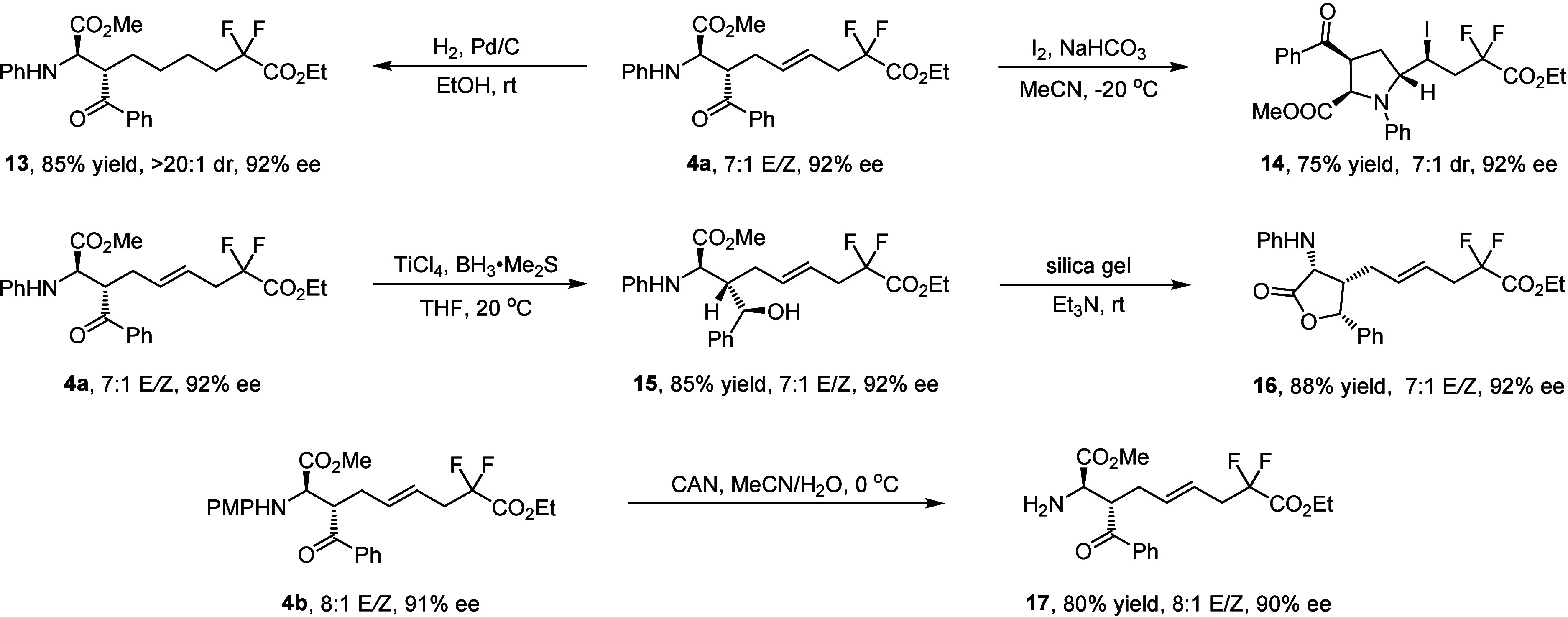
Demonstration of Synthetic Utility

To investigate the mechanism of this visible-light-induced asymmetric three-component radical cascade reaction, a series of control experiments were carried out. Steady-state Stern–Volmer quenching experiments suggested that the excited photocatalyst [Ir(dF(CF_3_)ppy)_2_(dtbbpy)]PF_6_ (*E*_1/2_*^III/II^ = +1.21 V vs SCE)^13^ was reductively quenched by glycine ester **1a** (*E*_1/2_^ox^ = +0.95 V vs SCE)^8b^ (see the Supporting Information for the details), which is the initial step of this cascade reaction. Next, radical trapping experiments were performed (Scheme 4). The desired cascade reaction was completely suppressed in the presence of typical C-radical trapping reagents, such as 2,2,6,6-tetramethylpiperidine-*N*-oxyl (TEMPO) or 1,1-diphenylethene. The radical scavenger TEMPO (3 equiv) annihilated the formation of **4a**, and the radical-trapping adduct **18** was detected by HMRS analysis [eq (1), Scheme 4], which suggested that the three-component cascade reaction follows a radical pathway. The addition of 1,1-diphenylethene resulted in the formation of the adduct **19** in 64% yield [eq (2), Scheme 4], which further verified the formation of radical ·CF_2_CO_2_Et in the reaction medium. During the investigation of the substrate scope of glycine ester under the standard reaction conditions, N-PMP glycine imino ester **20** could be isolated in 10% yield as a byproduct from the glycine ester **1b** [eq (3), Scheme 4], which revealed the presence of an imino ester intermediate in this cascade reaction. The performance of two enantioenriched (1*S*,2*R*)-**2a** and (1*R*,2*S*)-**2a** was also investigated in this cascade transformation, and the same reactivities were observed with maintained stereoselectivity control, which further confirmed that no kinetic resolution occurred in this three-component radical cascade reaction.

**Scheme 4:**
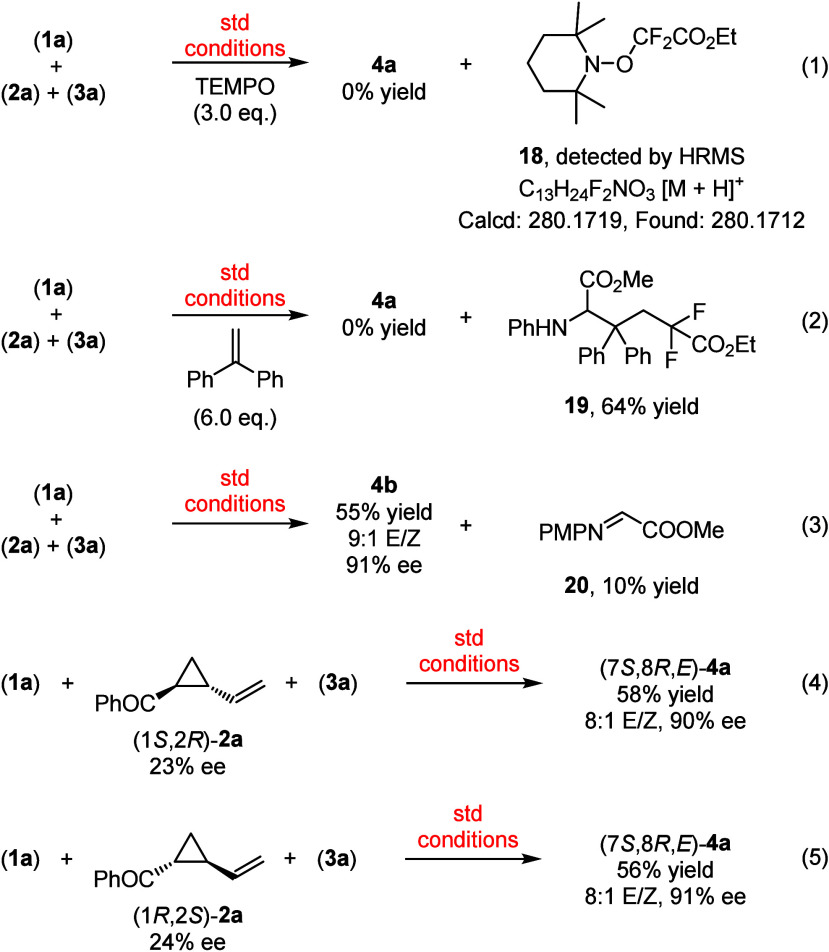
Control Experiments

Based on the experimental results, mechanistic studies, and previous reports, a plausible mechanism was proposed as shown in Scheme 5 for this catalytic asymmetric three-component radical cascade reaction. Upon visible-light irradiation, the excited-state photocatalyst [Ir^III^*] undergoes single electron oxidation(s) with glycine ester **1a** to generate the α-amino radical **B** or cationic iminium intermediate^14^**C** and the reduced-state [Ir^II^]. Single electron transfer between the [Ir^II^] species (*E*_1/2_^III/II^ = −1.37 V vs SCE)^13^ and compound **3a** (*E*_1/2_^red^ = −0.57 V vs SCE)^15^ forms the electrophilic C-radical **D** with regeneration of the ground-state [Ir^III^].^16^ Radical **D** undergoes a fast addition to the less hindered and electron-rich terminal olefinic carbon center of 2-vinylcyclopropyl ketone **2**, followed by a spontaneous ring-opening process to generate α-carbonyl radical **E** incorporating an *E*-dominated internal olefin moiety. Radical **E** will not further add to 2-vinylcyclopropyl ketone probably due to the steric hindrance, and the direct radical addition of strongly electrophilic radical **D** to the cationic iminium intermediate **C** is also not occurring probably due to a strong polarity mismatching, which explains the excellent chemoselectivity for this challenging radical relay cascade reaction. When methyl 2-bromo-2-methylpropanoate was employed under the standard reaction conditions, a two-component radical addition reaction occurred without the participation of VCP (**2a**) since the corresponding tertiary radical generated from methyl 2-bromo-2-methylpropanoate is less electrophilic (see Supporting Information for details). The cationic iminium **C** and α-carbon radical **E** are assembled by the bifunctional chiral phosphoric acid catalyst through H-bonding interactions to forge the enantioenriched cationic radical **F** via asymmetric radical addition. Subsequently, **F** is further reduced by the reduced-state [Ir^II^]^17^ to afford the final three-component adduct chiral fluorinated α-amino acid ester **4a** and complete the catalytic cycles. Alternatively, the stereodetermining step could proceed through an asymmetric radical–radical coupling pathway between α-amino radical **B** and α-carbon radical **E**.

**Scheme 5:**
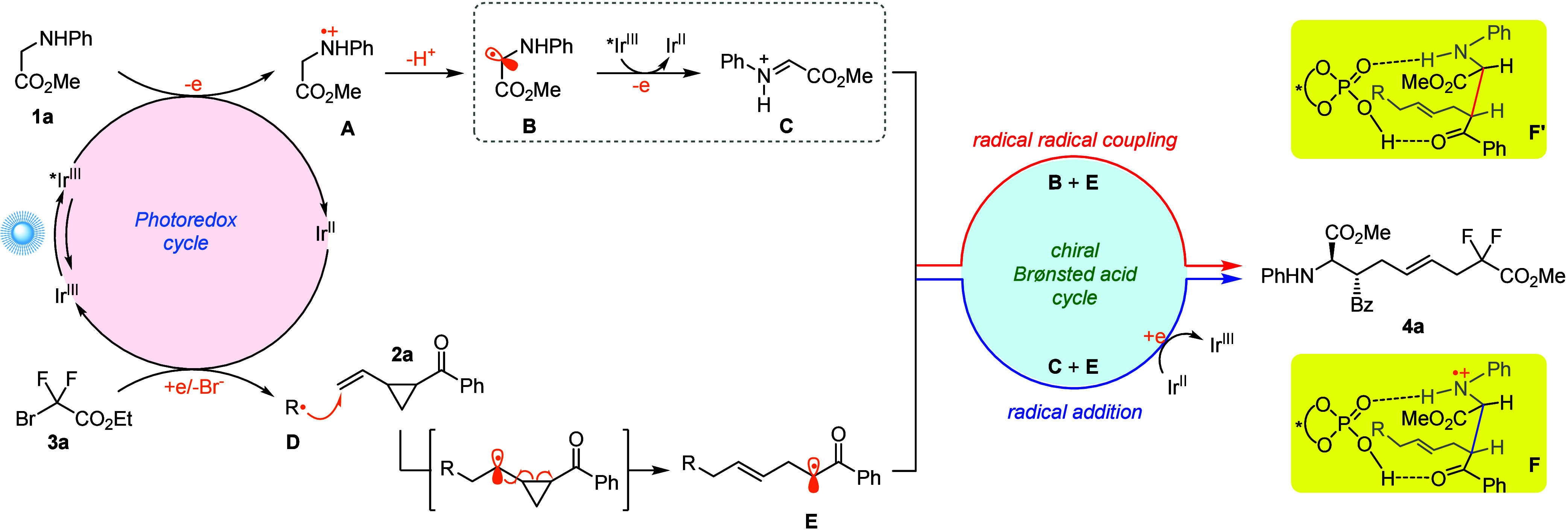
Plausible Mechanism

A DFT calculation was performed to investigate the mechanism of the three-component radical cascade reaction. As shown in Figure 1a, the radical addition of electrophilic radical **D** to alkene **2a** (via **TS-1**) followed by ring-opening (via **TS-2**) leads to the formation of α-carbonyl radical **E**, which is highly exergonic by 28.6 kcal/mol with respect to **2a** and radical **D**. The activation free energies are 13.4 and 5.2 kcal/mol, respectively. It should be noted that the generation of *E*-dominated internal olefin moiety is favored in the ring-opening process due to its higher stability and less steric repulsion with the difluoroacetate group. To reveal the most reliable reaction mechanism, the radical/iminium ion coupling pathway is initially studied. Computational studies suggest that the single electron oxidation of α-amino radical **B** by the excited-state photocatalyst [Ir^III^*] to generate the iminium ion requires an activation free energy of 14.7 kcal/mol (Figure 1b). Subsequent CPA-catalyzed asymmetric radical addition to the iminium ion through (*R*,*S*)-**TS-3** requires an activation free energy of 13.9 kcal/mol.

**Figure 1:**
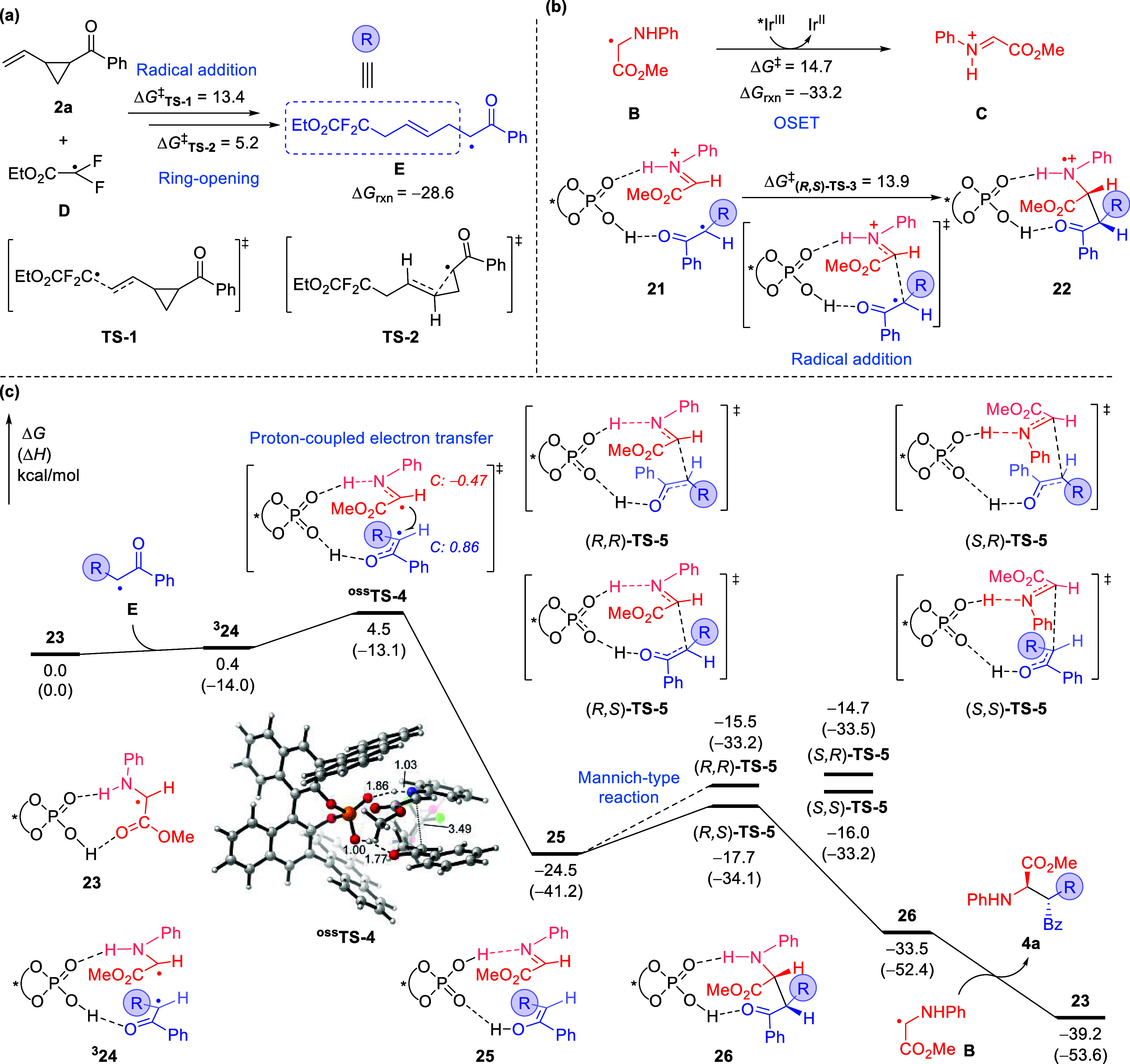
Computational studies of the three-component radical cascade reaction mechanism. (a) DFT study of the formation of α-carbonyl radical. (b) DFT study of the radical/iminium ion coupling pathway through radical addition. (c) Free energy profile of the radical–radical coupling pathway. The chiral phosphoric acid (*R*)-**C1** is used in the DFT calculation. The italic numbers in ^**OSS**^**TS-4** denote the Mulliken atomic spin densities on α-carbonyl and α-amino carbon. The bond lengths are in angstrom and energies are in kcal/mol. All energies were calculated at M06-2X/6-311+G(d,p)-SDD/SMD(acetonitrile)//M06-2X/6-31G(d) level of theory. See SI for computational details.

While in the computational study of the radical–radical coupling pathway, the direct radical–radical coupling transition state is not located. Instead, a proton-coupled electron transfer (PCET)^18^ transition state ^**OSS**^**TS-4** (Δ*G*^‡^ = 4.5 kcal/mol) can be obtained by subtly diminishing the distance between the α-carbonyl carbon and the α-amino carbon (Figure 1c). The PCET characteristic of this transition state has been verified by analyzing the variation of Mulliken atomic spin population and natural population charge (NPA) along the reaction coordinate^19^ (see Figure S9 in SI for details). Moreover, the intrinsic reaction coordinate (IRC) calculation demonstrates that the open-shell singlet transition state ^**OSS**^**TS-4** does not lead to C–C bond formation, which is consistent with the quite long C–C distance in ^**OSS**^**TS-4** (3.49 Å). Instead, a closed-shell singlet complex **25**, which contains the imine and enol moieties ligated to CPA through hydrogen bonding, is generated after the PCET. The formation of **25** is exergonic by 24.5 kcal/mol, which indicates that the multisite PCET through ^**OSS**^**TS-4** has a strong driving force. CPA plays a critical role as the chiral proton shuttle to promote the formation of enol and imine.^20^ From **25**, the CPA-catalyzed intramolecular Mannich-type reaction between the enol and imine moieties can produce the final product **4a** irreversibly through the transition state (*R*,*S*)-**TS-5** (Δ*G*^‡^ = 6.8 kcal/mol). We surmise that the CPA-mediated proton transfer in (*R*,*S*)-**TS-5** can enhance the nucleophilicity of the enol moiety and, thus, promote the nucleophilic addition to imine. It is also noteworthy that this radical–radical coupling pathway has a much lower activation free energy than that of the radical addition pathway shown in Figure 1b (Δ*G*^‡^ = 13.9 kcal/mol), which indicates the radical–radical coupling mechanism is more favored for this three-component radical cascade reaction.

In the radical–radical coupling pathway, the Mannich-type reaction turns out to be the stereodetermining step. Comparison of the enantiomers and diastereomers of **TS-5** shows that (*R*,*S*)-**TS-5** has the lowest energy barrier and verifies the absolute configuration of the product. As shown in Figure 2, the higher energy barrier of (*R*,*R*)-**TS-5** can be attributed to the steric clash of the difluoroacetate group with the 9-anthryl group of CPA. Moreover, the steric clash between the phenyl group of the enol moiety and the 9-anthryl group of CPA is also witnessed in transition states (*S*,*S*)-**TS-5** and (*S*,*R*)-**TS-5**, thereby accounting for their higher energy barriers with respect to (*R*,*S*)-**TS-5**.

**Figure 2:**
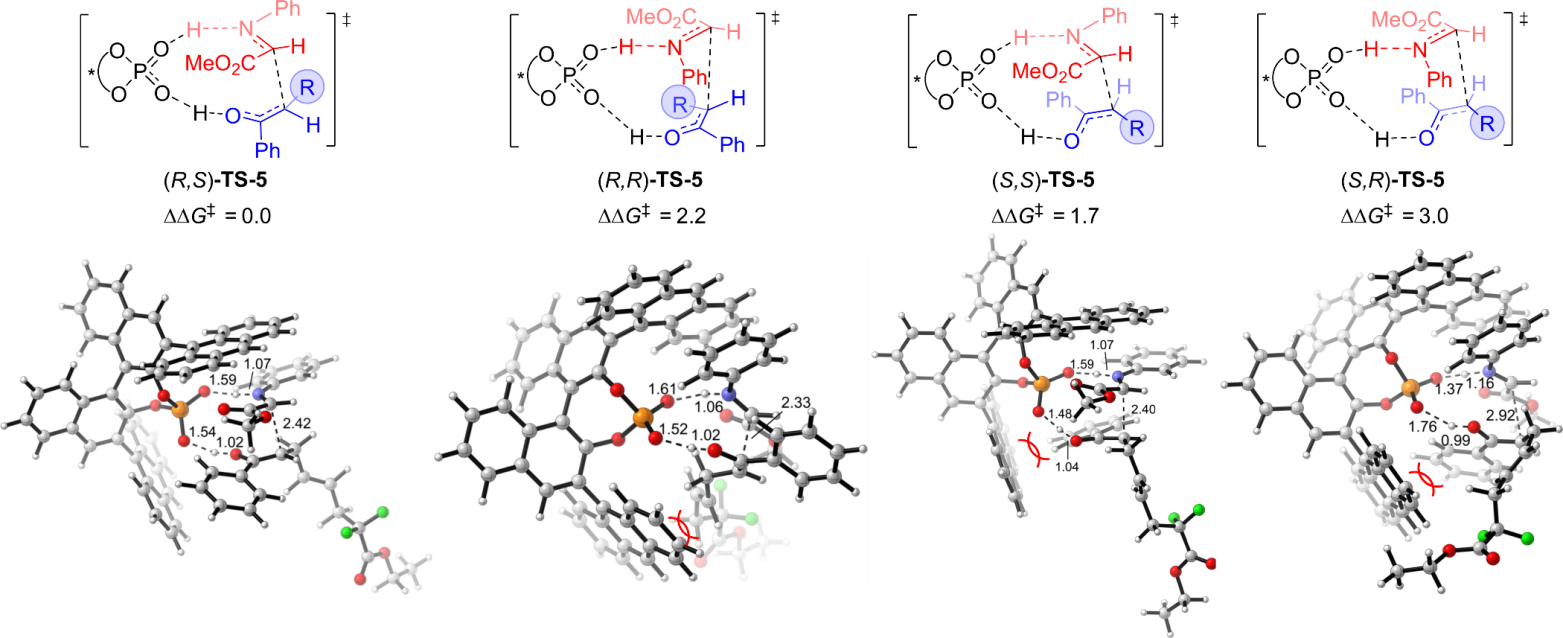
Optimized structures of the stereodetermining transition states. The bond lengths are in angstrom, and energies are in kcal/mol.

In summary, we have established an unprecedented catalytic asymmetric three-component radical cascade reaction via synergistic Brønsted acid/photoredox catalysis, affording a wide range of highly valuable chiral α-amino acid derivatives bearing two contiguous stereogenic centers and an internal *E*-dominated alkene moiety. This visible-light-induced radical relay cascade performed well with readily available glycine esters, α-bromo carbonyl compounds, and 2-vinylcyclopropyl ketones, and this novel protocol has opened a conceptually novel prospect for the facile buildup of enantioenriched α-amino acid derivatives with molecular complexity that cannot be easily accessed by the known two-electron reaction pathway. Control experiments and DFT computational mechanistic studies revealed that this three-component transformation undergoes through a radical relay protocol and a unique proton-coupled electron transfer (PCET)-promoted radical–radical coupling was the enantio-determining step. Further endeavors will be focused on applications of this three-component radical cascade reaction in organic synthesis, investigating a detailed reaction mechanism to reveal the origin of stereoselectivity control.

## Supporting Information Available

Experimental details and procedures, characterization and crystallographic data, and spectra of all unknown compounds (PDF)
X-ray crystallographic data for (7*S*,8*R*,*E*)-**4o** (CIF)
Transparent Peer Review report available (PDF)

## Supplementary Material

oc4c00970_si_001.pdf

oc4c00970_si_002.cif
