# Supplementary material for: Asymmetric Three-Component Radical Cascade Reactions Enabled by Synergistic Photoredox/Brønsted Acid Catalysis: Access to α-Amino Acid Derivatives
Source: ACS Cent Sci. 2024 Aug 16;11(1):36–45. doi: 10.1021/acscentsci.4c00970 (PMC11758273; doi:10.1021/acscentsci.4c00970)
Supplement: Supplementary file 1 — oc4c00970_si_001.pdf [file oc4c00970_si_001.pdf]

Supporting Information for

Asymmetric Three-Component Radical Cascade Reactions Enabled by Synergistic Photoredox/Brønsted Acid Catalysis: Access to α-Amino Acid Derivatives

Chao Che,[1,+] Yi-Nan Lu,[1,+] Ting Fang,[1,+] Guangjin Zhen,[1] Xiaotian Qi,[1]* and Chun-Jiang Wang[1,2]*

[1]College of Chemistry and Molecular Sciences, Wuhan University, Wuhan 430072, China
[2]State Key Laboratory of Elemento-organic Chemistry, Nankai University, Tianjin 300071, China
[+]These authors contributed equally to this work

E-mail: qi7xiaotian@whu.edu.cn (X.Q.); cjwang@whu.edu.cn (C.-J.W.)

Table of Contents

I. General Remarks....S2

II. Optimization of Reaction Conditions....S3

III. Preparation and characterization data of *trans*-2-vinylcyclopropyl ketones **2**....S5

IV. General Procedure for Asymmetric Three-Component Radical Cascade Reactions Enabled by Synergistic Photoredox/Brønsted Acid Catalysis: Access to α-Amino Acid Derivatives....S10

V. Spectral Characterization Data for the Products....S11

VI. Synthetic Transformations....S33

VII. Control Experiments....S38

VIII. Mechanistic Investigations....S41

IX. References....S44

X. NMR and HPLC Spectra....S45

XI. Computational details....S153

I General Remarks

^1H NMR spectra were recorded on a Bruker 400 MHz spectrometer in $CDCl_3$. Chemical shifts are reported in ppm with the internal chloroform signal at 7.26 ppm as a standard. The data are reported as (s = single, d = double, t = triple, q = quarte, m = multiple or unresolved, coupling constant(s) in Hz, integration). ^{13}C NMR spectra were recorded on a Bruker 100 MHz spectrometer in $CDCl_3$. Chemical shifts are reported in ppm with the internal chloroform signal at 77.0 ppm as a standard. ^{19}F NMR spectra were recorded on a Bruker 376 MHz spectrometer or Bruker 377 MHz NMR spectrometer in $CDCl_3$. Commercially obtained reagents were used without further purification. High resolution mass spectra (HR-MS) were recorded on a LTQ-Orbitrap Elite mass spectrometer with MeOH as solvent for the measurements. Commercially obtained reagents were used without further purification. Solvents were purified prior to use according to the standard methods. Unless otherwise noted, all reactions were performed under an atmosphere of N_2 in fire dried glassware, and set up on the bench top and conducted under nitrogen atmosphere while subject to irradiation from blue LED. All reactions were monitored by TLC with silica gel coated plates. Flash column chromatography was performed using 200-300 mesh silica gel. The enantiomeric excesses (ee) of the products were determined by high-performance liquid chromatography (HPLC) analysis performed on Agilent 1200 and 1260 Series chromatographs using a Diacel chiral column (25 cm)· Optical rotations were measured on an Rudolph Research Analytical Autopol VI polarimeter with $[\alpha]_D$ values reported in degrees; concentration (c) is in g/100 mL. The racemic products were obtained by running reactions with racemic catalysts or blending equal amount of two enantiomers. The absolute configurations of compound **4o** was determined unequivocally according to the X-ray diffraction analysis, and those of other adducts were deduced on the basis of these results. The *E*/*Z* ratio of the products was determined by ^{19}F NMR unless otherwise noted.

II Optimization of Reaction Conditions

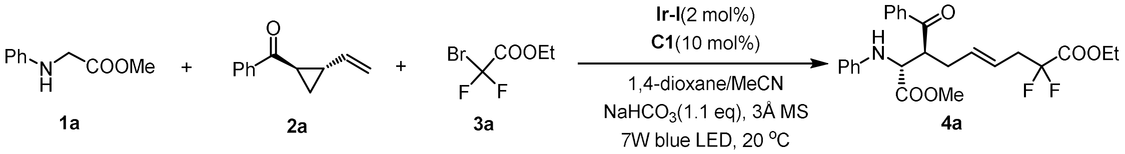

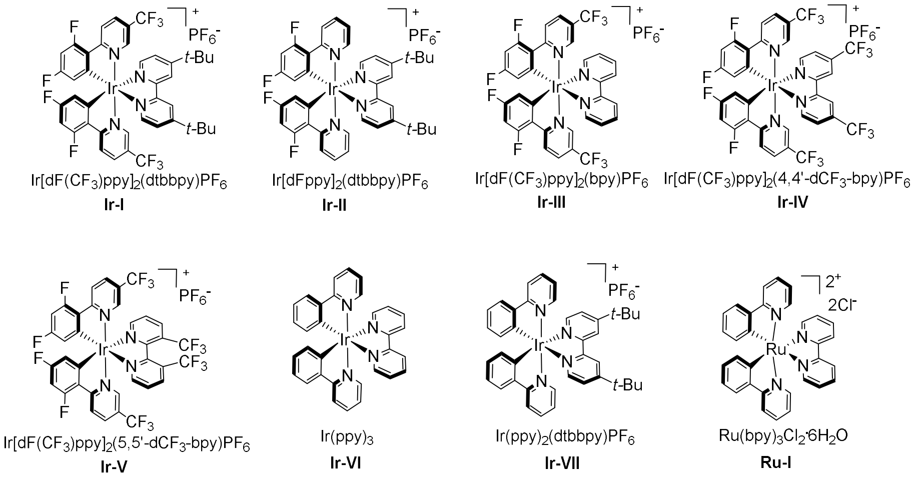

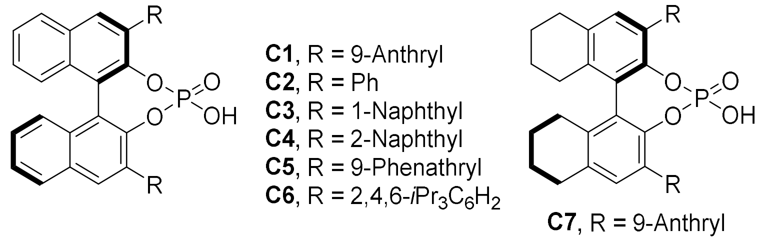

Entry	Variation from standard conditions	yield(%)[d]	*E*/*Z*[e]	*dr*[f]	*ee*(%)[g]
1[a]	**Ir-I**	58	7:1	>20:1	91
2[b]	**Ir-I**	60	7:1	>20:1	91
3[c]	**Ir-I**	65	8:1	>20:1	92
4	**Ir-II**	50	6:1	>20:1	88
5	**Ir-III**	53	7:1	12:1	74
6	**Ir-IV**	35	6:1	>20:1	86
7	**Ir-V**	trace	-	-	-
8	**Ir-VI**	29	5:1	>20:1	90
9	**Ir-VII**	51	6:1	>20:1	86
10	**Ru-I**	trace	-	-	-
11	Mes-Acr+	trace	-	-	-
12	Eosin Y	trace	-	-	-
13	Rose Bengal	trace	-	-	-
14	**C2**	37	6:1	8:1	50
15	**(*S*)-C3**	49	6:1	7:1	-68
16	**(*S*)-C4**	51	7:1	6:1	-64
17	**(*S*)-C5**	43	6:1	11:1	-68
18	**(*S*)-C6**	51	8:1	>20:1	-89
19	**C7**	51	8:1	>20:1	88
20	1,4-dioxane only	52	5:1	>20:1	88

21	MeCN only	44	8:1	>20:1	93
22	ClPh instead of 1,4-dioxane and MeCN	42	6:1	>20:1	86
23	DCM instead of1,4-dioxane and MeCN	34	6:1	>20:1	76
24	3W blue LED instead of 7W blue LED	46	7:1	>20:1	92
25	12W blue LED instead of 7W blue LED	49	6:1	14:1	90
26	Na_2CO_3 instead of $NaHCO_3$	Mess			
27	Et_3N instead of $NaHCO_3$	Mess			
28	10 °C	50	7:1	>20:1	91
29	30 °C	55	7:1	>20:1	84
30	Without PC	N.R.			
31	Without *hv*	N.R.			
32	Without CPA	39	6:1	4:1	0
33	Without base	16	6:1	>20:1	88

[a]Conditions: **1a** (0.2 mmol), **2a** (0.4 mmol), **3a** (0.6 mmol), **Ir-I** (2 mol %), (*R*)-**C1** (10 mol %), $NaHCO_3$ (1.1 equiv.) in 2 mL of 1,4-dioxane and MeCN at 20 °C under irradiation of 7W blue LED for 12 h. [b]**1a** (0.2 mmol), **2a** (0.3 mmol), **3a** (0.6 mmol) [c]**1a** (0.2 mmol), **2a** (0.3 mmol), **3a** (0.8 mmol) [d]Isolated yields. [e]*E*/*Z* was determined by ^{19}F NMR. [f] To determine the diastereoselectivity of **4a**, the corresponding suturated compound **4a'** could be achieved through Pd/C-catalyzed hydrogenation. The diastereoselectivity of **4a** is equal to that of **4a'**, which was deteremined by ^{1}H NMR. [g] *Ee* was determined by HPLC analysis.

III. Preparation and characterization data of 2-vinylcyclopropyl ketones 2

2-vinylcyclopropyl ketones **2** were prepared according to the procedure as shown below.

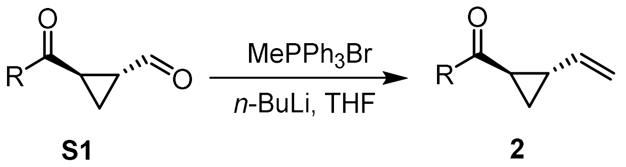

A solution of the Methyltriphenylphosphonium bromide (11 mmol, 1.1 equiv) in anhydrous THF (20 mL) was stirred at -10 °C for 15 min. Then, *n*-BuLi (2.5 M) (11 mmol, 1.1 equiv) was added dropwise under argon at 0 °C and the mixture was stirred for 20 min. Then, a solution of **S1**[1](10 mmol, 1 equiv) in anhydrous THF (20 mL) was stirred at -10 °C for 15 min. The readymade witting reagents were added dropwise under argon to the solution over a period of 1 to 1.5 hours. The reaction mixture was allowed to stir at -10 °C untill the initial material is completely consumed (as determined by TLC). After that, the reaction was quenched with sat. aq. NH_4Cl and allowed to warm to room temperature, and the solution was extracted with ethyl acetate (3 × 30 mL). The organic layers were combined and dried over Na_2SO_4. The solvent was evaporated, and the residue was subjected to column chromatography (silica gel) using petroleum ether/ethyl acetate to afford the corresponding product **2**.

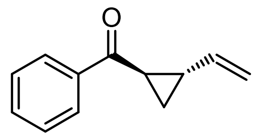

(*rac*)-**2a**

(*trans*)-phenyl(2-vinylcyclopropyl)methanone (*rac*-**2a**):[2]

yield (1.2 g, 70%); faint yellow oil.

^{1}H NMR (400 MHz, $CDCl_3$) δ 8.01 – 7.96 (m, 2H), 7.59 – 7.54 (m, 1H), 7.52 – 7.44 (m, 2H), 5.55 (ddd, *J* = 17.0, 10.2, 8.4 Hz, 1H), 5.25 – 5.17 (m, 1H), 5.04 (dd, *J* = 10.2, 1.4 Hz, 1H), 2.73 – 2.65 (m, 1H), 2.27 – 2.13 (m, 1H), 1.76 – 1.65 (m, 1H), 1.22 – 1.14 (m, 1H). ^{13}C NMR (100 MHz, $CDCl_3$) δ 198.7, 138.5, 137.8, 132.8, 128.5, 128.0, 115.0, 29.5, 26.6, 18.1.

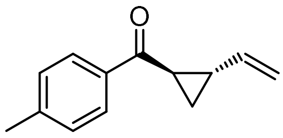

(*rac*)-**2j**

(*trans*)-*p*-tolyl(2-vinylcyclopropyl)methanone (*rac*-**2j**):

yield (1.19 g, 64%); yellow oil.

^{1}H NMR (400 MHz, $CDCl_3$) δ 7.89 (d, *J* = 8.2 Hz, 2H), 7.26 (d, *J* = 8.0 Hz, 2H), 5.54 (ddd, *J* = 17.0, 10.2, 8.4 Hz, 1H), 5.20 (dd, *J* = 17.0, 1.4 Hz, 1H), 5.02 (dd, *J* = 10.2, 1.4 Hz, 1H), 2.70 – 2.63 (m, 1H), 2.40 (s, 3H), 2.24 – 2.11 (m, 1H), 1.75 – 1.62 (m, 1H), 1.22 – 1.10 (m, 1H). ^{13}C NMR (100 MHz, $CDCl_3$) δ 198.1, 143.5, 138.5, 135.2, 129.1, 128.1, 114.8, 29.1, 26.3, 21.5, 17.8. HRMS (ESI+) Calcd. For $C_{13}H_{15}O^+$ ($[M+H]^+$): 187.1117, found: 187.1111.

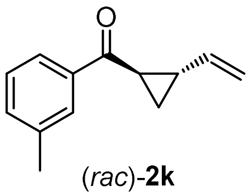

(*rac*)-**2k**

(*trans*)-*m*-tolyl(2-vinylcyclopropyl)methanone (*rac*-**2k**):

yield (1.21 g, 65%); faint yellow oil.

^{1}H NMR (400 MHz, $CDCl_3$) δ 7.83 – 7.72 (m, 2H), 7.42 – 7.32 (m, 2H), 5.54 (ddd, *J* = 17.0, 10.2, 8.4 Hz, 1H), 5.21 (dd, *J* = 17.0, 1.4 Hz, 1H), 5.04 (dd, *J* = 10.2, 1.4 Hz, 1H), 2.72 – 2.64 (m, 1H), 2.42 (s, 3H), 2.24 – 2.15 (m, 1H), 1.74 – 1.65 (m, 1H), 1.21 – 1.13 (m, 1H). ^{13}C NMR (100 MHz, $CDCl_3$) δ 198.8, 138.5, 138.3, 137.8, 133.5, 128.5, 128.4, 125.2, 114.9, 29.3, 26.6, 21.3, 18.1. HRMS (ESI+) Calcd. For $C_{13}H_{15}O^+$ ($[M+H]^+$): 187.1117, found: 187.1110.

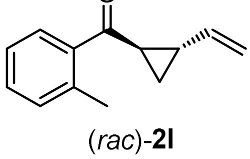

(*rac*)-**2l**

(*trans*)-*o*-tolyl(2-vinylcyclopropyl)methanone (*rac*-**2l**):

yield (1.25 g, 67%); faint yellow oil.

^{1}H NMR (400 MHz, $CDCl_3$) δ 7.69 – 7.61 (m, 1H), 7.40 – 7.32 (m, 1H), 7.31 – 7.20 (m, 2H), 5.51 (ddd, *J* = 17.0, 10.2, 8.4 Hz, 1H), 5.22 (dd, *J* = 17.0, 1.4 Hz, 1H), 5.04 (dd, *J* = 10.2, 1.4 Hz, 1H), 2.48 (s, 3H), 2.47 – 2.42 (m, 1H), 2.25 – 2.15 (m, 1H), 1.75 – 1.67 (m, 1H), 1.22 – 1.12 (m, 1H). ^{13}C NMR (100 MHz, $CDCl_3$) δ 202.9, 139.4, 138.4, 137.0, 131.5, 130.9, 128.3, 125.6, 115.0, 30.0, 29.8, 20.7, 18.3. HRMS (ESI+) Calcd. For $C_{13}H_{15}O^+$ ($[M+H]^+$): 187.1117, found: 187.1123.

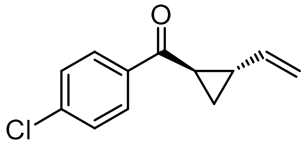

(*rac*)-**2m**

(*trans*)-(4-chlorophenyl)(-2-vinylcyclopropyl)methanone (*rac*-**2m**):

yield (1.18 g, 57%); yellow oil.

^{1}H NMR (400 MHz, $CDCl_3$) δ 7.95 – 7.81 (m, 2H), 7.42 – 7.34 (m, 2H), 5.50 (ddd, *J* = 17.0, 10.2, 8.4 Hz, 1H), 5.18 (dd, *J* = 17.0, 1.4 Hz, 1H), 5.01 (dd, *J* = 10.2, 1.4 Hz, 1H), 2.64 – 2.53 (m, 1H), 2.22 – 2.10 (m, 1H), 1.71 – 1.63 (m, 1H), 1.19 – 1.12 (m, 1H). ^{13}C NMR (100 MHz, $CDCl_3$) δ 197.1, 139.0, 138.1, 135.9, 129.3, 128.6, 115.1, 29.5, 26.4, 18.1. HRMS (ESI+) Calcd. For $C_{12}H_{11}ClONa^+$ ($[M+Na]^+$): 229.0391, found: 229.0391.

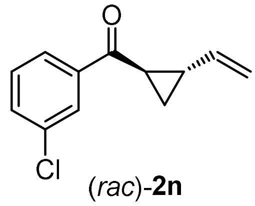

(*rac*)-**2n**

(*trans*)-(3-chlorophenyl)(-2-vinylcyclopropyl)methanone (*rac*-**2n**):

yield (1.14 g, 55%); yellow oil.

^{1}H NMR (400 MHz, $CDCl_3$) δ 7.97 – 7.91 (m, 1H), 7.90 – 7.82 (m, 1H), 7.57 – 7.50 (m, 1H), 7.46 – 7.38 (m, 1H), 5.54 (ddd, *J* = 17.0, 10.2, 8.4 Hz, 1H), 5.30 – 5.17 (m, 1H), 5.06 (dd, *J* = 10.2, 1.5 Hz, 1H), 2.68 – 2.57 (m, 1H), 2.27 – 2.16 (m, 1H), 1.75 – 1.66 (m, 1H), 1.26 – 1.19 (m, 1H). ^{13}C NMR (100 MHz, $CDCl_3$) δ 197.4, 139.4, 138.1,

134.9, 132.7, 129.9, 128.2, 126.1, 115.4, 29.9, 26.7, 18.4. HRMS (ESI+) Calcd. For $C_{12}H_{12}ClO^+$ ([M+H]$^+$): 207.0571, found: 207.0580.

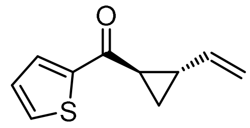

(*rac*)-**2o**

(*trans*)-thiophen-2-yl(2-vinylcyclopropyl)methanone (*rac*-**2o**):

yield (0.91 g, 51%); yellow oil.

^{1}H NMR (400 MHz, $CDCl_3$) δ 7.84 – 7.76 (m, 1H), 7.69 – 7.59 (m, 1H), 7.17 – 7.10 (m, 1H), 5.51 (ddd, *J* = 17.0, 10.2, 8.4 Hz, 1H), 5.24 – 5.14 (m, 1H), 5.05 – 4.97 (m, 1H), 2.62 – 2.48 (m, 1H), 2.27 – 2.14 (m, 1H), 1.71 – 1.61 (m, 1H), 1.21 – 1.11 (m, 1H). ^{13}C NMR (100 MHz, $CDCl_3$) δ 190.9, 144.8, 138.2, 133.4, 131.6, 128.1, 115.0, 28.9, 27.3, 17.8. HRMS (ESI+) Calcd. For $C_{10}H_{10}OSNa^+$ ([M+Na]$^+$): 201.0345, found: 201.0354.

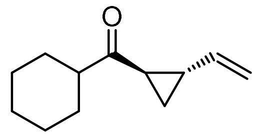

(*rac*)-**2p**

(*trans*)-cyclohexyl(2-vinylcyclopropyl)methanone yield (*rac*-**2p**):

yield (0.89 g, 50%); faint yellow oil.

^{1}H NMR (400 MHz, $CDCl_3$) δ 5.40 (ddd, *J* = 17.0, 10.2, 8.4 Hz, 1H), 5.12 (dd, *J* = 17.0, 1.4 Hz, 1H), 4.95 (dd, *J* = 10.2, 1.4 Hz, 1H), 2.52 – 2.41 (m, 1H), 2.04 – 1.83 (m, 4H), 1.81 – 1.61 (m, 3H), 1.43 – 1.37 (m, 1H), 1.37 – 1.28 (m, 3H), 1.28 – 1.14 (m, 2H), 0.99 – 0.92 (m, 1H). ^{13}C NMR (100 MHz, $CDCl_3$) δ 211.75, 138.54, 114.46, 77.32, 77.00, 76.68, 51.53, 28.26, 28.24, 28.15, 25.88, 25.64, 25.60, 17.25. HRMS (ESI+) Calcd. For $C_{12}H_{19}O^+$ ([M+H]$^+$): 179.1430, found: 179.1424.

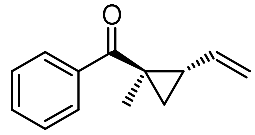

(*rac*)-**5**

(*trans*)-(1-methyl-2-vinylcyclopropyl)(phenyl)methanone (*rac*-**5**):

yield (1.34 g, 72%); faint yellow oil.

^{1}H NMR (400 MHz, $CDCl_3$) δ 7.77 – 7.73 (m, 2H), 7.54 – 7.48 (m, 1H), 7.47 – 7.41 (m, 2H), 5.70 (ddd, *J* = 17.0, 10.2, 8.4 Hz, 1H), 5.34 – 5.28 (m, 1H), 5.27 – 5.23 (m, 1H), 2.04 – 1.94 (m, 1H), 1.87 – 1.79 (m, 1H), 1.40 (s, 3H), 0.83 – 0.79 (m, 1H). ^{13}C NMR (100 MHz, $CDCl_3$) δ 203.0, 137.1, 135.5, 131.9, 128.5, 128.3, 117.6, 31.8, 29.5, 19.9, 17.1. HRMS (ESI+) Calcd. For $C_{13}H_{15}O^+$ ($[M+H]^+$): 187.1117, found: 187.1112.

IV General Procedure for Asymmetric Three-Component Radical Cascade Reactions Enabled by Synergistic Photoredox/Brønsted Acid Catalysis: Access to α-Amino Acid Derivatives

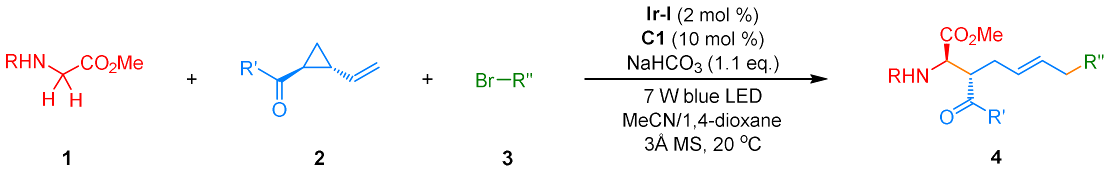

1 **2** **3** **4**

A flame dried Schlenk tube was cooled to ambient temperature. To this flask were added **1** (0.40 mmol, 1.0 equiv), **2** (0.60 mmol, 1.5 equiv), **3** (1.60 mmol, 4 equiv), $[Ir(dF(CF_3)ppy)_2(dtbbpy)]PF_6$ (0.008 mmol, 2 mol %), (*R*)-**C1** (0.04 mmol, 10 mol %), $NaHCO_3$ (0.44 mmol, 1.1 equiv), 3Å MS (400 mg). Subsequently, MeCN(2.0 mL)/1,4-Dioxane (2.0 mL) were added. Then, this mixture solution was degassed 3 times via 'freeze-pump-thaw' procedure. After that, this resulting solution was stirred at a distance of ~3 cm under irradiation by 7W blue LED at 20 °C for 12 h. After filtration and evaporation, the residue was purified by column chromatography using petroleum ether/ethyl acetate as eluent (15:1 to 5:1) to generate the desired products.

V Spectral Characterization Data for the Products

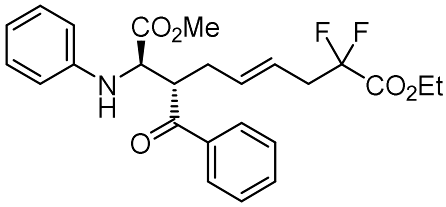

4a

1-ethyl 9-methyl (7*S*,8*R*,*E*)-7-benzoyl-2,2-difluoro-8-(phenylamino)non-4-enedioate (**4a**):

yield (119.4 mg, 65%); *E*/*Z* = 7:1; yellow oil; $[\alpha]_D^{28}$ = -7.5 (*c* 1.5, acetone); ^{1}H NMR (400 MHz, $CDCl_3$) δ 7.94 – 7.89 (m, 2H), 7.65 – 7.56 (m, 1H), 7.53 – 7.46 (m, 2H), 7.21 – 7.12 (m, 2H), 6.77 – 6.71 (m, 1H), 6.69 – 6.61 (m, 2H), 5.71 – 5.58 (m, 1H), 5.50 – 5.37 (m, 1H), 4.80 (d, *J* = 10.7 Hz, 1H), 4.45 – 4.35 (m, 1H), 4.25 (q, *J* = 7.2 Hz, 2H), 4.14 – 4.06 (m, 1H), 3.50 (s, 3H), 2.79 – 2.64 (m, 2H), 2.61 – 2.45 (m, 2H), 1.29 (t, *J* = 7.2 Hz, 3H). ^{13}C NMR (100 MHz, $CDCl_3$) δ 201.8, 173.4, 163.8 (t, *J* = 32.7 Hz), 146.9, 136.8, 133.6, 133.5, 129.3, 128.8, 128.4, 122.4 (t, *J* = 5.0 Hz), 118.5, 115.1 (t, *J* = 249.5 Hz), 112.6, 62.8, 57.6, 52.2, 47.5, 37.9 (t, *J* = 23.7 Hz), 32.5, 14.0. ^{19}F NMR (376 MHz, $CDCl_3$) δ -105.5 (t, *J* = 16.3 Hz). HRMS (ESI+) Calcd. For $C_{25}H_{28}F_2NO_5^+$ ($[M+H]^+$): 460.1930, found: 460.1931. The product was analyzed by HPLC to determine the enantiomeric excess: 92% *ee* (Chiralpak AD-H, *i*-propanol/hexane = 3/97, flow rate 1.0 mL/min, λ = 254 nm); t_r = 42.02 and 59.40 min.

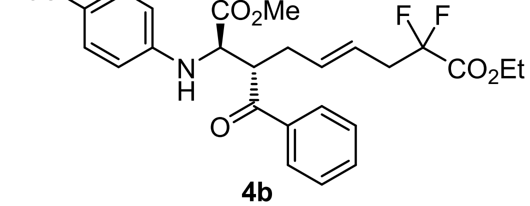

4b

1-ethyl 9-methyl (7*S*,8*R*,*E*)-7-benzoyl-2,2-difluoro-8-((4-methoxyphenyl)amino)non-4-enedioate (**4b**):

yield (107.6 mg, 55%); *E*/*Z* = 9:1; yellow oil; $[\alpha]_D^{28}$ = -11.1 (*c* 0.8, acetone); ^{1}H NMR (400 MHz, $CDCl_3$) δ 7.94 – 7.87 (m, 2H), 7.63 – 7.54 (m, 1H), 7.52 – 7.44 (m, 2H), 6.79 – 6.72 (m, 2H), 6.66 – 6.59 (m, 2H), 5.69 – 5.57 (m, 1H), 5.49 – 5.36 (m, 1H),

4.31 (d, J = 5.8 Hz, 1H), 4.25 (q, J = 7.2 Hz, 2H), 4.09 – 4.01 (m, 1H), 3.73 (s, 3H), 3.51 (s, 3H), 2.77 – 2.63 (m, 2H), 2.61 – 2.45 (m, 2H), 1.29 (t, J = 7.2 Hz, 3H). ^{13}C NMR (100 MHz, $CDCl_3$) δ 201.5, 173.5, 163.7 (t, J = 32.7 Hz), 152.9, 140.8, 136.9, 133.5, 133.5, 128.7, 128.3, 122.2 (t, J = 5.1 Hz), 115.5, 115.0 (t, J = 250.0 Hz), 114.8, 62.8, 59.2, 55.6, 52.0, 47.6, 37.9 (t, J = 23.9 Hz), 32.4, 13.9. ^{19}F NMR (376 MHz, $CDCl_3$) δ -105.5 (t, J = 16.3 Hz). HRMS (ESI+) Calcd. For $C_{26}H_{29}F_2NO_6Na^+$ ($[M+Na]^+$): 512.1855, found: 512.1856. The product was analyzed by HPLC to determine the enantiomeric excess: 91% *ee* (Chiralpak AD-H, *i*-propanol/hexane = 5/95, flow rate 1.0 mL/min, λ = 254 nm); t_r = 41.95 and 60.92 min.

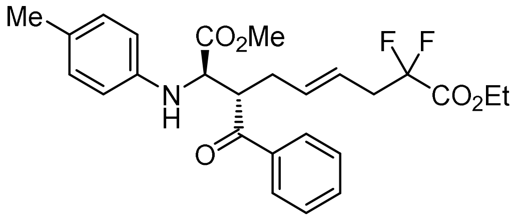

4c

1-ethyl 9-methyl (7*S*,8*R*,*E*)-7-benzoyl-2,2-difluoro-8-(*p*-tolylamino)non-4-enedioate (**4c**):

yield (92.0 mg, 49%); *E*/*Z* = 7:1; yellow oil; $[\alpha]_D^{28}$ = -9.8 (*c* 0.7, acetone); ^{1}H NMR (400 MHz, $CDCl_3$) δ 7.96 – 7.87 (m, 2H), 7.63 – 7.55 (m, 1H), 7.52 – 7.43 (m, 2H), 7.04 – 6.90 (m, 2H), 6.62 – 6.52 (m, 2H), 5.71 – 5.56 (m, 1H), 5.50 – 5.37 (m, 1H), 4.37 (d, J = 5.6 Hz, 1H), 4.25 (q, J = 7.2 Hz, 2H), 4.12 – 4.02 (m, 1H), 3.51 (s, 3H), 2.78 – 2.64 (m, 2H), 2.62 – 2.43 (m, 2H), 2.23 (s, 3H), 1.30 (t, J = 7.2 Hz, 3H). ^{13}C NMR (100 MHz, $CDCl_3$) δ 201.6, 173.5, 163.7 (t, J = 32.1 Hz), 144.5, 136.8, 133.5, 129.7, 128.8, 128.3, 127.8, 122.2 (t, J = 5.2 Hz), 115.0 (t, J = 249.7 Hz), 113.8, 62.8, 58.1, 52.1, 47.5, 37.9 (t, J = 23.7 Hz), 32.4, 20.3, 13.9. ^{19}F NMR (376 MHz, $CDCl_3$) δ -105.5 (t, J = 16.3 Hz). HRMS (ESI+) Calcd. For $C_{26}H_{30}F_2NO_5^+$ ($[M+H]^+$): 474.2087, found: 474.2090. The product was analyzed by HPLC to determine the enantiomeric excess: 90% *ee* (Chiralpak ID, *i*-propanol/hexane = 5/95, flow rate 1.0 mL/min, λ = 254 nm); t_r = 21.87 and 43.25 min.

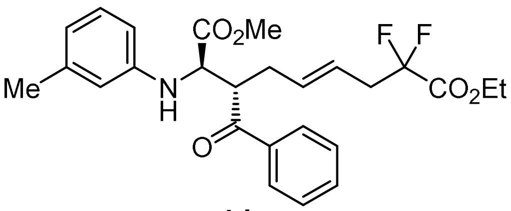

4d

1-ethyl 9-methyl (7*S*,8*R*,*E*)-7-benzoyl-2,2-difluoro-8-(*m*-tolylamino)non-4-enedioate (**4d**):

yield (89.0 mg, 47%); *E*/*Z* = 9:1; yellow oil; $[\alpha]_D^{28}$ = -11.7 (*c* 0.7, acetone); ^{1}H NMR (400 MHz, $CDCl_3$) δ 7.94 – 7.89 (m, 2H), 7.63 – 7.56 (m, 1H), 7.53 – 7.46 (m, 2H), 7.10 – 7.02 (m, 1H), 6.57 (d, *J* = 7.4 Hz, 1H), 6.51 – 6.41 (m, 2H), 5.70 – 5.58 (m, 1H), 5.51 – 5.35 (m, 1H), 4.41 (d, *J* = 5.4 Hz, 1H), 4.25 (q, *J* = 7.2 Hz, 2H), 4.13 – 4.04 (m, 1H), 3.51 (s, 3H), 2.78 – 2.65 (m, 2H), 2.63 – 2.45 (m, 2H), 2.27 (s, 3H), 1.30 (t, *J* = 7.2 Hz, 3H). ^{13}C NMR (100 MHz, $CDCl_3$) δ 201.7, 173.4, 163.8 (t, *J* = 32.8 Hz), 146.9, 139.1, 136.9, 133.6, 133.5, 129.2, 128.8, 128.4, 122.4 (t, *J* = 5.0 Hz), 119.5, 115.1 (t, *J* = 249.9 Hz), 114.6, 110.7, 62.8, 57.6, 52.2, 47.5, 37.9 (t, *J* = 23.7 Hz), 32.5, 21.6, 13.9. ^{19}F NMR (376 MHz, $CDCl_3$) δ -105.5 (td, *J* = 16.2, 4.4 Hz). HRMS (ESI+) Calcd. For $C_{26}H_{30}F_2NO_5^+$ ($[M+H]^+$): 474.2087, found: 474.2083. The product was analyzed by HPLC to determine the enantiomeric excess: 91% *ee* (Chiralpak IE, *i*-propanol/hexane = 5/95, flow rate 1.0 mL/min, λ = 254 nm); t_r = 18.91 and 39.26 min.

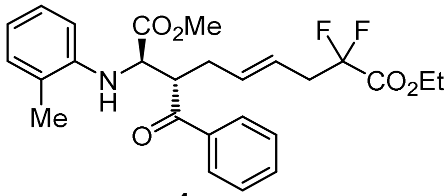

4e

1-ethyl 9-methyl (7*S*,8*R*,E)-7-benzoyl-2,2-difluoro-8-(*o*-tolylamino)non-4-enedioate (**4e**):

yield (66.1 mg, 35%); *E*/*Z* = 6:1; yellow oil; $[\alpha]_D^{28}$ = -9.8 (*c* 0.8, acetone); ^{1}H NMR (400 MHz, $CDCl_3$) δ 7.98 – 7.88 (m, 2H), 7.63 – 7.57 (m, 1H), 7.55 – 7.44 (m, 2H), 7.14 – 6.99 (m, 2H), 6.74 – 6.64 (m, 1H), 6.58 – 6.48 (m, 1H), 5.70 – 5.57 (m, 1H), 5.47 – 5.36 (m, 1H), 4.84 (s, 1H), 4.42 (d, *J* = 5.4 Hz, 1H), 4.24 (q, *J* = 7.2 Hz, 2H), 4.16 – 4.08 (m, 1H), 3.50 (s, 3H), 2.77 – 2.66 (m, 2H), 2.64 – 2.58 (m, 1H), 2.56 – 2.46 (m,

1H), 2.18 (s, 3H), 1.29 (t, J = 7.2 Hz, 3H). ^{13}C NMR (100 MHz, $CDCl_3$) δ 202.0, 173.5, 163.8 (t, J = 32.2 Hz), 144.9, 137.0, 133.6, 133.4, 130.3, 128.8, 128.3, 127.0, 123.1, 122.4 (t, J = 5.1 Hz), 118.0, 115.0 (t, J = 250.0 Hz), 110.2, 62.8, 57.7, 52.2, 47.2, 37.9, 32.7 (t, J = 23.8 Hz), 17.4, 13.9. ^{19}F NMR (376 MHz, $CDCl_3$) δ -105.5 (t, J = 16.0 Hz). HRMS (ESI+) Calcd. For $C_{26}H_{30}F_2NO_5^+$ ($[M+H]^+$): 474.2087, found: 474.2081. The product was analyzed by HPLC to determine the enantiomeric excess: 87% *ee* (Chiralpak IE, *i*-propanol/hexane = 5/95, flow rate 1.0 mL/min, λ = 254 nm); t_r = 12.38 and 19.24 min.

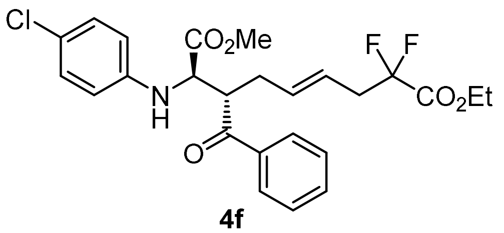

4f

1-ethyl 9-methyl (7*S*,8*R*,*E*)-7-benzoyl-8-((4-chlorophenyl)amino)-2,2-difluoronon-4-enedioate **(4f)**:

yield (98.7 mg, 50%); *E*/*Z* = 8:1; yellow oil; $[\alpha]_D^{28}$ = -13.0 (*c* 0.8, acetone); ^{1}H NMR (400 MHz, $CDCl_3$) δ 7.95 – 7.87 (m, 2H), 7.63 – 7.56 (m, 1H), 7.52 – 7.44 (m, 2H), 7.15 – 7.08 (m, 2H), 6.62 – 6.54 (m, 2H), 5.70 – 5.58 (m, 1H), 5.50 – 5.34 (m, 1H), 4.91 (d, J = 10.9 Hz, 1H), 4.37 – 4.30 (m, 1H), 4.25 (q, J = 7.2 Hz, 2H), 4.15 – 4.05 (m, 1H), 3.51 (s, 3H), 2.79 – 2.66 (m, 2H), 2.62 – 2.40 (m, 2H), 1.29 (t, J = 7.2 Hz, 3H). ^{13}C NMR (100 MHz, $CDCl_3$) δ 201.8, 173.0, 163.7 (t, J = 32.5 Hz), 145.6, 136.5, 133.7, 133.2, 129.0, 128.8, 128.3, 122.9, 122.6 (t, J = 50.7 Hz), 115.0 (t, J = 250.0 Hz), 114.7, 62.8, 57.4, 52.2, 47.2, 37.8 (t, J = 23.9 Hz), 32.4, 13.9. ^{19}F NMR (376 MHz, $CDCl_3$) δ -105.4 (t, J = 16.3 Hz). HRMS (ESI+) Calcd. For $C_{25}H_{27}ClF_2NO_5^+$ ($[M+H]^+$): 494.1540, found: 494.1538. The product was analyzed by HPLC to determine the enantiomeric excess: 94% *ee* (Chiralpak AD-H and IF-3, *i*-propanol/hexane = 10/90, flow rate 1.0 mL/min, λ = 254 nm); t_r = 29.44 and 49.13 min.

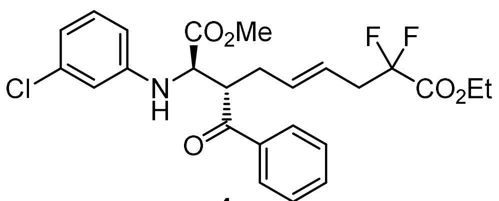

4g

1-ethyl 9-methyl (7*S*,8*R*,*E*)-7-benzoyl-8-((3-chlorophenyl)amino)-2,2-difluoronon-4-enedioate (**4g**):

yield (101.1 mg, 51%); *E*/*Z* = 7:1; yellow oil; $[\alpha]_D^{28}$ = -11.0 (*c* 1.0, acetone); ^{1}H NMR (400 MHz, $CDCl_3$) δ 8.00 – 7.84 (m, 2H), 7.65 – 7.55 (m, 1H), 7.55 – 7.45 (m, 2H), 7.12 – 7.00 (m, 1H), 6.74 – 6.68 (m, 1H), 6.64 – 6.60 (m, 1H), 6.56 – 6.48 (m, 1H), 5.72 – 5.55 (m, 1H), 5.49 – 5.35 (m, 1H), 5.02 (s, 1H), 4.42 – 4.31 (m, 1H), 4.26 (q, *J* = 7.1 Hz, 2H), 4.17 – 4.06 (m, 1H), 3.52 (s, 3H), 2.82 – 2.64 (m, 2H), 2.62 – 2.44 (m, 2H), 1.30 (t, *J* = 7.1 Hz, 3H). ^{13}C NMR (100 MHz, $CDCl_3$) δ 201.9, 172.9, 163.8 (t, *J* = 32.2 Hz), 148.2, 136.5, 135.0, 133.8, 133.2, 130.3, 128.9, 128.4, 122.8 (t, *J* = 5.1 Hz), 118.3, 115.0 (t, *J* = 254.7 Hz), 113.4, 111.8, 62.9, 57.1, 52.3, 47.2, 37.9 (t, *J* = 23.7 Hz), 32.5, 13.9. ^{19}F NMR (376 MHz, $CDCl_3$) δ -105.5 (t, *J* = 16.1 Hz). HRMS (ESI+) Calcd. For $C_{25}H_{27}ClF_2NO_5^+$ ($[M+H]^+$): 494.1540, found: 494.1536. The product was analyzed by HPLC to determine the enantiomeric excess: 91% *ee* (Chiralpak ID, *i*-propanol/hexane = 10/90, flow rate 1.0 mL/min, λ = 254 nm); t_r = 13.62 and 39.01 min.

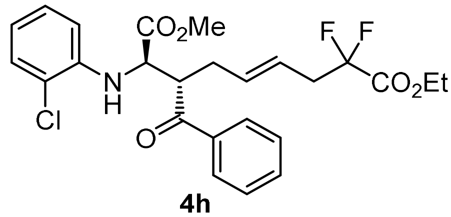

4h

1-ethyl 9-methyl (7*S*,8*R*,*E*)-7-benzoyl-8-((2-chlorophenyl)amino)-2,2-difluoronon-4-enedioate (**4h**):

yield (61.2 mg, 31%); *E*/*Z* = 14:1; yellow oil; $[\alpha]_D^{28}$ = -17.5 (*c* 0.5, acetone); ^{1}H NMR (400 MHz, $CDCl_3$) δ 7.97 – 7.90 (m, 2H), 7.64 – 7.57 (m, 1H), 7.54 – 7.46 (m, 2H), 7.28 – 7.22 (m, 1H), 7.15 – 7.06 (m, 1H), 6.71 – 6.59 (m, 2H), 5.69 – 5.56 (m, 2H), 5.47 – 5.33 (m, 1H), 4.45 – 4.37 (m, 1H), 4.25 (q, *J* = 7.2 Hz, 2H), 4.19 – 4.09 (m, 1H), 3.54 (s, 3H), 2.76 – 2.64 (m, 2H), 2.62 – 2.45 (m, 2H), 1.30 (t, *J* = 7.2 Hz, 3H). ^{13}C

NMR (100 MHz, $CDCl_3$) δ 201.7, 172.8, 163.8 (t, *J* = 32.4 Hz), 143.0, 136.6, 133.7, 133.2, 129.4, 128.8, 128.4, 127.7, 122.6 (t, *J* = 5.3 Hz), 120.2, 118.3, 115.0 (t, *J* = 249.4 Hz), 111.6, 62.8, 57.2, 52.4, 47.1, 37.9 (t, *J* = 23.7 Hz), 32.5, 13.9. ^{19}F NMR (377 MHz, $CDCl_3$) δ -105.5. HRMS (ESI+) Calcd. For $C_{25}H_{27}ClF_2NO_5^+$ ($[M+H]^+$): 494.1540 found: 494.1538. The product was analyzed by HPLC to determine the enantiomeric excess: 89% *ee* (Chiralpak IE, *i*-propanol/hexane = 2/98, flow rate 1.0 mL/min, λ = 254 nm); t_r = 16.32 and 26.24 min.

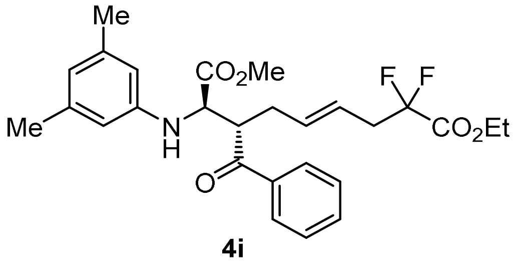

4i

1-ethyl 9-methyl (7*S*,8*R*,*E*)-7-benzoyl-8-((3,5-dimethylphenyl)amino)-2,2-difluoronon-4-enedioate **(4i)**:

yield (97.2 mg, 50%); *E*/*Z* = 13:1; yellow oil; $[\alpha]_D^{28}$ = -14.9 (*c* 0.7, acetone); ^{1}H NMR (400 MHz, $CDCl_3$) δ 7.96 – 7.87 (m, 2H), 7.63 – 7.56 (m, 1H), 7.53 – 7.44 (m, 2H), 6.40 (s, 1H), 6.31 – 6.26 (m, 2H), 5.70 – 5.59 (m, 1H), 5.51 – 5.37 (m, 1H), 4.42 – 4.38 (m, 1H), 4.25 (q, *J* = 7.2 Hz, 2H), 4.11 – 4.03 (m, 1H), 3.51 (s, 3H), 2.79 – 2.64 (m, 2H), 2.61 – 2.44 (m, 2H), 2.23 (s, 6H), 1.30 (t, *J* = 7.2 Hz, 3H). ^{13}C NMR (100 MHz, $CDCl_3$) δ 201.6, 173.4, 163.7 (t, *J* = 32.7 Hz), 146.8, 138.9, 136.9, 133.6, 133.5, 128.8, 128.3, 122.2 (t, *J* = 5.5 Hz), 120.5, 115.1 (t, *J* = 250.0 Hz), 111.5, 62.8, 57.6, 52.1, 47.5, 37.9 (t, *J* = 23.9Hz), 32.3, 21.4, 13.9. ^{19}F NMR (376 MHz, $CDCl_3$) δ -105.5 (td, *J* = 16.2, 11.1 Hz). HRMS (ESI+) Calcd. For $C_{27}H_{32}F_2NO_5^+$ ($[M+H]^+$): 488.2243, found: 488.2245. The product was analyzed by HPLC to determine the enantiomeric excess: 92% *ee* (Chiralpak IE, *i*-propanol/hexane = 5/95, flow rate 1.0 mL/min, λ = 254 nm); t_r = 15.31 and 38.29 min.

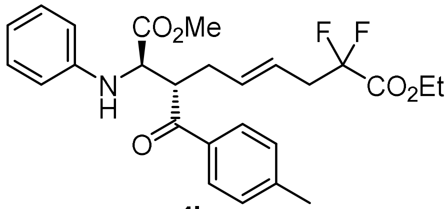

4j

1-ethyl 9-methyl (7*S*,8*R*,*E*)-2,2-difluoro-7-(4-methylbenzoyl)-8-(phenylamino)non-4-enedioate (**4j**):

yield (105.6 mg, 56%); *E*/*Z* = 7:1; yellow oil; $[\alpha]_D^{28}$ = -13.9 (*c* 0.8, acetone); ^{1}H NMR (400 MHz, $CDCl_3$) δ 7.85 – 7.79 (m, 2H), 7.32 – 7.26 (m, 2H), 7.21 – 7.12 (m, 2H), 6.76 – 6.70 (m, 1H), 6.66 – 6.59 (m, 2H), 5.71 – 5.56 (m, 1H), 5.49 – 5.35 (m, 1H), 4.83 (s, 1H), 4.41 – 4.35 (m, 1H), 4.25 (q, *J* = 7.1 Hz, 2H), 4.13 – 4.01 (m, 1H), 3.50 (s, 3H), 2.78 – 2.63 (m, 2H), 2.60 – 2.46 (m, 2H), 2.42 (s, 3H), 1.29 (t, *J* = 7.1 Hz, 3H). ^{13}C NMR (100 MHz, $CDCl_3$) δ 201.3, 173.4, 163.8 (t, *J* = 32.8 Hz), 146.9, 144.6, 134.2, 133.5, 129.5, 129.3, 128.5, 122.3 (t, *J* = 5.5 Hz), 118.4, 115.1 (t, *J* = 250.0 Hz), 113.6, 62.8, 57.6, 52.1, 47.2, 37.9 (t, *J* = 23.9 Hz), 32.6, 21.7, 13.9. ^{19}F NMR (376 MHz, $CDCl_3$) δ -105.5 (t, *J* = 16.3 Hz). HRMS (ESI+) Calcd. For $C_{26}H_{30}F_2NO_5^+$ ($[M+H]^+$): 474.2087, found: 474.2094. The product was analyzed by HPLC to determine the enantiomeric excess: 90% *ee* (Chiralpak ID, *i*-propanol/hexane = 10/90, flow rate 1.0 mL/min, λ = 254 nm); t_r = 18.82 and 28.18 min.

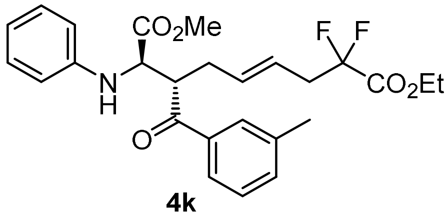

4k

1-ethyl 9-methyl (7*S*,8*R*,*E*)-2,2-difluoro-7-(3-methylbenzoyl)-8-(phenylamino)non-4-enedioate (**4k**):

yield (86.1 mg, 46%); *E*/*Z* = 7:1; yellow oil; $[\alpha]_D^{28}$ = -8.8 (*c* 1.3, acetone); ^{1}H NMR (400 MHz, $CDCl_3$) δ 7.76 – 7.67 (m, 2H), 7.43 – 7.33 (m, 2H), 7.20 – 7.12 (m, 2H), 6.77 – 6.69 (m, 1H), 6.67 – 6.61 (m, 2H), 5.71 – 5.55 (m, 1H), 5.49 – 5.36 (m, 1H), 4.80 (s, 1H), 4.40 (d, *J* = 5.4 Hz, 1H), 4.24 (q, *J* = 7.2 Hz, 2H), 4.12 – 4.04 (m, 1H), 3.51 (s, 3H), 2.79 – 2.64 (m, 2H), 2.60 – 2.46 (m, 2H), 2.42 (s, 3H), 1.29 (t, *J* = 7.2 Hz, 3H).

^{13}C NMR (100 MHz, $CDCl_3$) δ 201.8, 173.3, 163.7 (t, J = 32.7 Hz), 146.9, 138.6, 136.8, 134.3, 133.5, 129.2, 128.8, 128.6, 125.5, 122.3 (t, J = 5.1 Hz), 118.4, 115.0 (t, J = 249.9 Hz), 113.6, 62.7, 57.5, 52.1, 47.4, 37.9 (t, J = 23.6 Hz), 32.4, 21.3, 13.9. ^{19}F NMR (376 MHz, $CDCl_3$) δ -105.5 (t, J = 16.3 Hz). HRMS (ESI+) Calcd. For $C_{26}H_{30}F_2NO_5^+$ ($[M+H]^+$): 474.2087, found: 474.2083. The product was analyzed by HPLC to determine the enantiomeric excess: 88% *ee* (Chiralpak ID, *i*-propanol/hexane = 10/90, flow rate 1.0 mL/min, λ = 254 nm); t_r = 13.16 and 27.25 min.

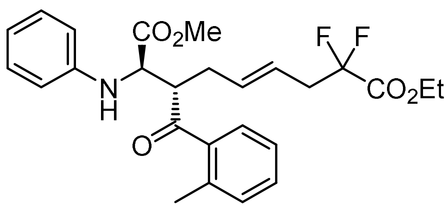

4l

1-ethyl 9-methyl (7*S*,8*R*,*E*)-2,2-difluoro-7-(2-methylbenzoyl)-8-(phenylamino)non-4-enedioate (**4l**):

yield (75.7 mg, 40%); *E*/*Z* = 7:1; yellow oil; $[\alpha]_D^{28}$ = +1.4 (*c* 1.3, acetone); ^{1}H NMR (400 MHz, $CDCl_3$) δ 7.63 – 7.56 (m, 1H), 7.44 – 7.35 (m, 1H), 7.32 – 7.28 (m, 1H), 7.28 – 7.24 (m, 1H), 7.21 – 7.13 (m, 2H), 6.78 – 6.72 (m, 1H), 6.67 – 6.60 (m, 2H), 5.71 – 5.56 (m, 1H), 5.49 – 5.36 (m, 1H), 4.35 (d, J = 5.3 Hz, 1H), 4.25 (q, J = 7.2 Hz, 2H), 4.01 – 3.91 (m, 1H), 3.56 (s, 3H), 2.79 – 2.66 (m, 2H), 2.58 – 2.49 (m, 1H), 2.48 – 2.37 (m, 1H), 2.43 (s, 3H), 1.29 (t, J = 7.2 Hz, 3H). ^{13}C NMR (100 MHz, $CDCl_3$) δ 205.2, 173.2, 163.8 (t, J = 32.3 Hz), 146.9, 138.5, 137.8, 133.7, 132.0, 131.6, 129.3, 128.2, 125.7, 122.2 (t, J = 5.2 Hz), 118.6, 115.0 (t, J = 249.8 Hz), 113.7, 62.8, 57.6, 52.2, 50.8, 37.9 (t, J = 23.9 Hz), 31.8, 20.7, 13.9. ^{19}F NMR (376 MHz, $CDCl_3$) δ -105.5 (t, J = 16.1 Hz). HRMS (ESI+) Calcd. For $C_{26}H_{30}F_2NO_5^+$ ($[M+H]^+$): 474.2087, found: 474.2088. The product was analyzed by HPLC to determine the enantiomeric excess: 61% *ee* (Chiralpak IE, *i*-propanol/hexane = 2/98, flow rate 1.0 mL/min, λ = 254 nm); t_r = 20.22 and 26.85 min.

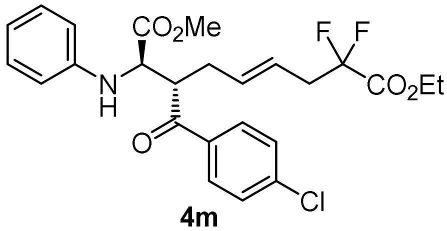

4m

1-ethyl 9-methyl (7*S*,8*R*,*E*)-7-(4-chlorobenzoyl)-2,2-difluoro-8-(phenylamino)non-4-enedioate (**4m**):

yield (102.2 mg, 52%); *E*/*Z* = 9:1; yellow oil; $[\alpha]_D^{28}$= -9.3 (*c* 1.7, acetone); ^{1}H NMR (400 MHz, $CDCl_3$) δ 7.89 – 7.80 (m, 2H), 7.50 – 7.42 (m, 2H), 7.20 – 7.12 (m, 2H), 6.78 – 6.71 (m, 1H), 6.68 – 6.59 (m, 2H), 5.67 – 5.56 (m, 1H), 5.49 – 5.35 (m, 1H), 4.73 (d, *J* = 10.9 Hz, 1H), 4.45 – 4.34 (m, 1H), 4.26 (q, *J* = 7.1 Hz, 2H), 4.06 – 3.98 (m, 1H), 3.54 (s, 3H), 2.82 – 2.62 (m, 2H), 2.60 – 2.42 (m, 2H), 1.30 (t, *J* = 7.2 Hz, 3H). ^{13}C NMR (100 MHz, $CDCl_3$) δ 199.6, 172.3, 162.8 (t, *J* = 32.2 Hz), 145.8, 139.2, 134.1, 132.2, 128.7, 128.3, 128.1, 121.6 (t, *J* = 5.1 Hz), 117.7, 114.0 (t, *J* = 249.9 Hz),, 112.7, 61.8, 56.7, 51.3, 46.5, 36.8 (t, *J* = 24.0 Hz),, 31.5, 12.9. ^{19}F NMR (376 MHz, $CDCl_3$) δ -105.6 (t, *J* = 16.3 Hz). HRMS (ESI+) Calcd. For $C_{25}H_{27}ClF_2NO_5^+$ ([M+H]$^+$): 494.1540, found: 494.1547. The product was analyzed by HPLC to determine the enantiomeric excess: 92% *ee* (Chiralpak IE, *i*-propanol/hexane = 2/98, flow rate 1.0 mL/min, λ = 254 nm); t_r = 22.73 and 32.39 min.

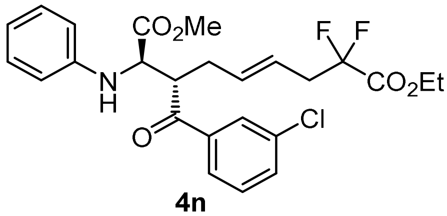

4n

1-ethyl 9-methyl (7*S*,8*R*,*E*)-7-(3-chlorobenzoyl)-2,2-difluoro-8-(phenylamino)non-4-enedioate (**4n**):

yield (101.8 mg, 52%); *E*/*Z* = 8:1; yellow oil; $[\alpha]_D^{28}$ = -5.8 (*c* 0.6, acetone); ^{1}H NMR (400 MHz, $CDCl_3$) δ 7.92 – 7.84 (m, 1H), 7.83 – 7.73 (m, 1H), 7.61 – 7.51 (m, 1H), 7.47 – 7.40 (m, 1H), 7.21 – 7.13 (m, 2H), 6.79 – 6.71 (m, 1H), 6.68 – 6.61 (m, 2H), 5.67 – 5.57 (m, 1H), 5.48 – 5.36 (m, 1H), 4.70 (d, *J* = 10.8 Hz, 1H), 4.45 – 4.36 (m, 1H), 4.26 (q, *J* = 7.2 Hz, 2H), 4.05 – 3.97 (m, 1H), 3.56 (s, 3H), 2.78 – 2.64 (m, 2H),

2.60 – 2.43 (m, 2H), 1.30 (t, J = 7.2 Hz, 3H). ^{13}C NMR (100 MHz, $CDCl_3$) δ 200.5, 173.2, 163.7 (t, J = 32.7 Hz), 146.7, 138.4, 135.2, 133.5, 133.1, 130.1, 129.3, 128.4, 126.4, 122.7 (t, J = 5.1 Hz), 118.7, 115.0 (t, J = 250.1 Hz), 113.8, 62.8, 57.7, 52.3, 47.8, 37.8 (t, J = 24.0 Hz), 32.4, 13.9. ^{19}F NMR (376 MHz, $CDCl_3$) δ -105.5 (t, J = 16.3 Hz). HRMS (ESI+) Calcd. For $C_{25}H_{27}ClF_2NO_5^+$ ($[M+H]^+$): 494.1540, found: 494.1547. The product was analyzed by HPLC to determine the enantiomeric excess: 92% *ee* (Chiralpak ID, *i*-propanol/hexane = 10/90, flow rate 1.0 mL/min, λ = 254 nm); t_r = 11.71 and 22.34 min.

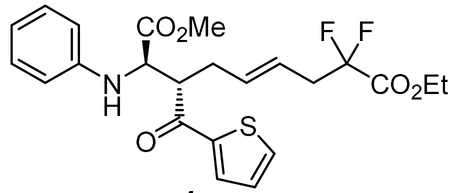

4o

1-ethyl 9-methyl (7*S*,8*R*,*E*)-2,2-difluoro-8-(phenylamino)-7-(thiophene-2-carbonyl)non-4-enedioate (**4o**):

yield (103 mg, 55%); *E*/*Z* = 9:1; brown solid; m.p. 80 - 82 ºC; $[\alpha]_D^{28}$ = -3.6 (*c* 0.6, acetone); ^{1}H NMR (400 MHz, $CDCl_3$) δ 7.75 – 7.68 (m, 2H), 7.20 – 7.11 (m, 3H), 6.73 (t, J = 7.3 Hz, 1H), 6.63 (d, J = 7.9 Hz, 2H), 5.70 – 5.54 (m, 1H), 5.51 – 5.36 (m, 1H), 4.78 (s, 1H), 4.39 (d, J = 5.4 Hz, 1H), 4.25 (q, J = 7.0 Hz, 2H), 3.88 – 3.81 (m, 1H), 3.51 (s, 3H), 2.77 – 2.65 (m, 2H), 2.64 – 2.47 (m, 2H), 1.30 (t, J = 7.0 Hz, 3H). ^{13}C NMR (100 MHz, $CDCl_3$) δ 193.7, 173.2, 163.7 (t, J = 32.3 Hz), 146.7, 144.3, 135.2, 133.2, 132.7, 129.3, 128.4, 122.5 (t, J = 5.1 Hz), 118.5, 115.0 (t, J = 250.0 Hz), 113.6, 62.8, 57.9, 52.1, 49.1, 37.9 (t, J = 23.6 Hz), 32.8, 13.9. ^{19}F NMR (376 MHz, $CDCl_3$) δ -105.5 (t, J = 16.1 Hz). HRMS (ESI+) Calcd. For $C_{23}H_{26}F_2NO_5S^+$ ($[M+H]^+$): 466.1494, found: 466.1502. The product was analyzed by HPLC to determine the enantiomeric excess: 92% *ee* (Chiralpak IC, *i*-propanol/hexane = 5/95, flow rate 1.0 mL/min, λ = 254 nm); t_r = 39.39 and 43.55 min.

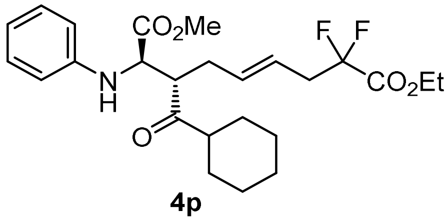

4p

1-ethyl 9-methyl (7*S*,8*R*,*E*)-7-(cyclohexanecarbonyl)-2,2-difluoro-8-(phenylamino)non-4-enedioate (**4p**):

yield (94.6 mg, 51%); *E*/*Z* = 7:1; yellow oil; $[\alpha]_D^{28}$ = +9.7 (*c* 0.7, acetone); ^{1}H NMR (400 MHz, $CDCl_3$) δ 7.20 – 7.11 (m, 2H), 6.77 – 6.68 (m, 1H), 6.65 – 6.58 (m, 2H), 5.66 – 5.54 (m, 1H), 5.47 – 5.34 (m, 1H), 4.75 (s, 1H), 4.30 (q, *J* = 7.2 Hz, 2H), 4.21 (d, *J* = 5.2 Hz, 1H), 3.64 (s, 3H), 3.34 – 3.25 (m, 1H), 2.84 – 2.68 (m, 2H), 2.47 – 2.29 (m, 3H), 1.89 – 1.63 (m, 5H), 1.32 (t, *J* = 7.2 Hz, 3H), 1.30 – 1.16 (m, 5H). ^{13}C NMR (100 MHz, $CDCl_3$) δ 214.8, 173.5, 163.8 (t, *J* = 32.4 Hz), 146.9, 133.6, 129.3, 122.3 (t, *J* = 5.0 Hz), 118.5, 115.0 (t, *J* = 250.0 Hz), 113.6, 62.8, 57.2, 52.2, 51.1, 51.0, 37.9 (t, *J* = 24,0 Hz), 31.9, 28.1, 27.8, 25.7, 25.6, 25.5, 13.9. ^{19}F NMR (377 MHz, $CDCl_3$) δ -105.5 (d, *J* = 16.3 Hz). HRMS (ESI+) Calcd. For $C_{25}H_{34}F_2NO_5^+$ ($[M+H]^+$): 466.2400, found: 466.2401. The product was analyzed by HPLC to determine the enantiomeric excess: 81% *ee* (Chiralpak IE, *i*-propanol/hexane = 10/90, flow rate 1.0 mL/min, λ = 254 nm); t_r = 11.45 and 19.17 min.

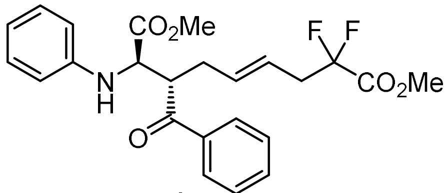

4q

dimethyl (7*S*,8*R*,*E*)-7-benzoyl-2,2-difluoro-8-(phenylamino)non-4-enedioate (**4q**):

yield (96.4 mg, 54%); *E*/*Z* = 8:1; yellow oil; $[\alpha]_D^{28}$ =-10.8 (*c* 0.8, acetone); ^{1}H NMR (400 MHz, $CDCl_3$) δ 7.95 – 7.88 (m, 2H), 7.65 – 7.56 (m, 1H), 7.54 – 7.44 (m, 2H), 7.20 – 7.12 (m, 2H), 6.81 – 6.70 (m, 1H), 6.67 – 6.59 (m, 2H), 5.72 – 5.57 (m, 1H), 5.49 – 5.35 (m, 1H), 4.79 (d, *J* = 10.9 Hz, 1H), 4.45 – 4.33 (m, 1H), 4.15 – 4.03 (m, 1H), 3.80 (s, 3H), 3.51 (s, 3H), 2.80 – 2.65 (m, 2H), 2.61 – 2.46 (m, 2H). ^{13}C NMR (100 MHz, $CDCl_3$) δ 201.7, 173.4, 164.2 (t, *J* = 32.6 Hz), 146.9, 136.8, 133.6, 133.5,

129.3, 128.8, 128.3, 122.3 (t, J = 5.4 Hz), 118.5, 115.1 (t, J = 249.5 Hz), 113.6, 57.6, 53.3, 52.2, 47.4, 37.9 (t, J = 24.0 Hz), 32.5. ^{19}F NMR (377 MHz, $CDCl_3$) δ -105.3. HRMS (ESI+) Calcd. For $C_{24}H_{26}F_2NO_5^+$ ($[M+H]^+$): 446.1774, found: 446.1771. The product was analyzed by HPLC to determine the enantiomeric excess: 90% *ee* (Chiralpak IE, *i*-propanol/hexane = 10/90, flow rate 1.0 mL/min, λ = 254 nm); t_r = 14.42 and 26.25 min.

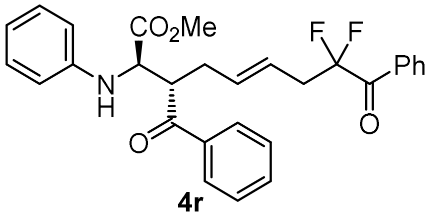

4r

methyl (2*R*,3*S*,*E*)-3-benzoyl-8,8-difluoro-9-oxo-9-phenyl-2-(phenylamino)non-5-enoate (**4r**):

yield (59.2 mg, 30%); *E*/*Z* = 10:1; yellow oil; $[\alpha]_D^{28}$ = -5.9 (*c* 0.7, acetone); ^{1}H NMR (400 MHz, $CDCl_3$) δ 8.11 – 8.00 (m, 2H), 7.97 – 7.86 (m, 2H), 7.68 – 7.56 (m, 2H), 7.53 – 7.42 (m, 4H), 7.19 – 7.06 (m, 2H), 6.76 – 6.67 (m, 1H), 6.66 – 6.61 (m, 2H), 5.71 – 5.60 (m, 1H), 5.57 – 5.46 (m, 1H), 4.41 (d, J = 5.2 Hz, 1H), 4.16 – 4.06 (m, 1H), 3.51 (s, 3H), 2.95 – 2.79 (m, 2H), 2.64 – 2.44 (m, 2H). ^{13}C NMR (100 MHz, $CDCl_3$) δ 201.9, 189.0 (t, J = 31.3 Hz), 173.4, 146.9, 136.8, 134.3, 133.5, 133.1, 131.9 (t, J = 2.6 Hz), 130.1 (t, J = 3.4 Hz), 129.2, 128.7, 128.6, 128.3, 123.1 (t, J = 5.2 Hz), 118.5 (t, J = 240.0 Hz), 118.4, 113.6, 57.5, 52.1, 47.5, 37.3 (t, J = 23.3 Hz), 32.6. ^{19}F NMR (376 MHz, $CDCl_3$) δ -99.2 (td, J = 16.9, 6.5 Hz). HRMS (ESI+) Calcd. For $C_{29}H_{28}F_2NO_4^+$ ($[M+H]^+$): 492.1980, found: 492.1976. The product was analyzed by HPLC to determine the enantiomeric excess: 93% *ee* (Chiralpak AD-H, *i*-propanol/hexane = 10/90, flow rate 1.0 mL/min, λ = 254 nm); t_r = 17.62 and 24.92 min.

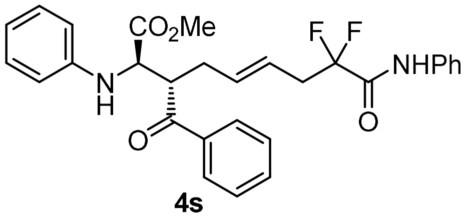

4s

methyl (2*R*,3*S*,*E*)-3-benzoyl-8,8-difluoro-9-oxo-2,9-bis(phenylamino)non-5-enoate (**4s**):

yield (84.8 mg, 42%); *E*/*Z* = 13:1; yellow oil; $[\alpha]_D^{28}$ = -4.0 (*c* 0.7, acetone); ^{1}H NMR (400 MHz, $CDCl_3$) δ 7.96 – 7.84 (m, 3H), 7.62 – 7.50 (m, 3H), 7.49 – 7.42 (m, 2H), 7.40 – 7.32 (m, 2H), 7.22 – 7.10 (m, 3H), 6.77 – 6.70 (m, 1H), 6.68 – 6.59 (m, 2H), 5.76 – 5.63 (m, 1H), 5.54 – 5.39 (m, 1H), 4.80 (s, 1H), 4.41 (d, *J* = 5.3 Hz, 1H), 4.16 – 4.04 (m, 1H), 3.49 (s, 3H), 2.97 – 2.74 (m, 2H), 2.66 – 2.41 (m, 2H). ^{13}C NMR (100 MHz, $CDCl_3$) δ 201.7, 173.3, 161.6 (t, *J* = 28.4 Hz), 146.9, 136.7, 135.9, 133.6, 133.5, 129.3, 129.1, 128.8, 128.3, 125.6, 122.5 (t, *J* = 5.2 Hz), 120.3, 118.5, 116.9 (t, *J* = 253.4 Hz), 113.6, 57.6, 52.1, 47.4, 37.2 (t, *J* = 23.9 Hz), 32.4. ^{19}F NMR (376 MHz, $CDCl_3$) δ -105.15 – -105.45 (m). HRMS (ESI+) Calcd. For $C_{29}H_{29}F_2N_2O_4^+$ ($[M+H]^+$): 507.2090, found: 507.2084 The product was analyzed by HPLC to determine the enantiomeric excess: 90% *ee* (Chiralpak IE, *i*-propanol/hexane = 10/90, flow rate 1.0 mL/min, λ = 254 nm); t_r = 34.86 and 85.11 min.

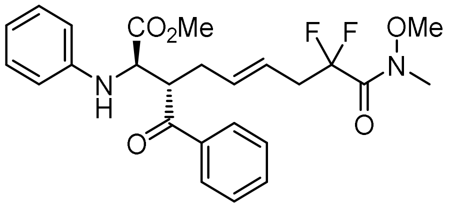

4t

methyl (2*R*,3*S*,*E*)-3-benzoyl-8,8-difluoro-9-(methoxy(methyl)amino)-9-oxo-2-(phenylamino)non-5-enoate (**4t**):

yield (88.0 mg, 46%); *E*/*Z* = 6:1 was determined by ^{1}H NMR; yellow oil; $[\alpha]_D^{28}$ = -7.3(*c* 0.7, acetone); ^{1}H NMR (400 MHz, $CDCl_3$) δ 7.95 – 7.88 (m, 2H), δ 7.61 – 7.55 (m, 1H), 7.51 – 7.44 (m, 2H), 7.20 – 7.13 (m, 2H), 6.76 – 6.69 (m, 1H), 6.68 – 6.60 (m, 2H), 5.69 – 5.56 (m, 1H), 5.54 – 5.42 (m, 1H), 4.42 (d, *J* = 5.3 Hz, 1H), 4.16 – 4.03 (m, 1H), 3.69 (s, 3H), 3.51 (s, 3H), 3.19 (s, 3H), 2.88 – 2.71 (m, 2H), 2.62 – 2.44 (m, 2H). ^{13}C NMR (100 MHz, $CDCl_3$) δ 201.9, 173.4, 146.9, 136.7, 133.5, 132.8, 129.2, 128.7, 128.3, 123.1 (t, *J* = 5.0 Hz), 118.3, 113.6, 61.9, 57.5, 52.1, 47.5, 37.8 (t, *J* = 24.0 Hz), 32.4, 27.5. ^{19}F NMR (377 MHz, $CDCl_3$) δ -103.1. HRMS (ESI+) Calcd. For $C_{25}H_{29}F_2N_2O_5^+$ ($[M+H]^+$): 475.2039, found: 475.2044. The product was analyzed by HPLC to

determine the enantiomeric excess: 90% *ee* (Chiralpak AD-H, *i*-propanol/hexane = 10/90, flow rate 1.0 mL/min, λ = 254 nm); t_r = 22.22 and 30.74 min.

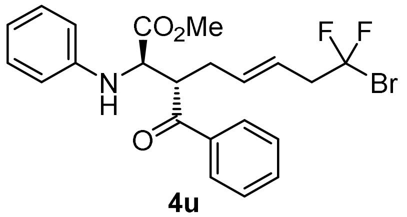

4u

methyl (2*R*,3*S*,*E*)-3-benzoyl-8-bromo-8,8-difluoro-2-(phenylamino)oct-5-enoate (**4u**): yield (85.6 mg, 46%); *E*/*Z* = 8:1; white solid; m.p. 96 - 98 °C;; $[\alpha]_D^{28}$ = -9.2 (*c* 0.7, acetone); ^{1}H NMR (400 MHz, $CDCl_3$) δ 7.99 – 7.89 (m, 2H), 7.65 – 7.57 (m, 1H), 7.55 – 7.45 (m, 2H), 7.23 – 7.11 (m, 2H), 6.78 – 6.71 (m, 1H), 6.69 – 6.61 (m, 2H), 5.78 – 5.64 (m, 1H), 5.58 – 5.42 (m, 1H), 4.83 (s, 1H), 4.44 (d, *J* = 5.3 Hz, 1H), 4.21 – 4.08 (m, 1H), 3.52 (s, 3H), 3.06 – 2.89 (m, 2H), 2.68 – 2.47 (m, 2H). ^{13}C NMR (100 MHz, $CDCl_3$) δ 201.7, 173.3, 146.9, 136.8, 134.0, 133.6, 129.3, 128.8, 128.3, 123.3 (t, *J* = 3.9 Hz), 121.5 (t, *J* = 303.9 Hz), 118.5, 113.6, 57.7, 52.2, 47.3 (t, *J* = 22.6 Hz), 47.2, 32.5. ^{19}F NMR (376 MHz, $CDCl_3$) δ -44.7 (t, *J* = 13.7 Hz). HRMS (ESI+) Calcd. For $C_{22}H_{23}BrF_2NO_3^+$ ($[M+H]^+$): 466.0824, found: 466.0819. The product was analyzed by HPLC to determine the enantiomeric excess: 92% *ee* (Chiralpak AD-H, *i*-propanol/hexane = 5/95, flow rate 1.0 mL/min, λ = 290 nm); t_r = 20.8 and 22.91 min.

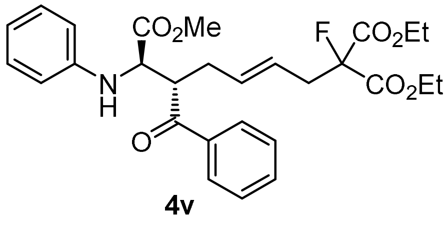

4v

1,1-diethyl 7-methyl (6*S*,7*R*,*E*)-6-benzoyl-1-fluoro-7-(phenylamino)hept-3-ene-1,1,7-tricarboxylate (**4v**):

yield (123.2 mg, 60%); *E*/*Z* = 15:1 was determined by HPLC; yellow oil; $[\alpha]_D^{28}$ = -6.2 (*c* 0.8, acetone); ^{1}H NMR (400 MHz, $CDCl_3$) δ 7.98 – 7.85 (m, 2H), 7.62 – 7.52 (m, 1H), 7.52 – 7.45 (m, 2H), 7.22 – 7.12 (m, 2H), 6.76 – 6.69 (m, 1H), 6.66 – 6.59 (m, 2H), 5.67 – 5.54 (m, 1H), 5.50 – 5.39 (m, 1H), 4.79 (s, 1H), 4.39 (d, *J* = 5.2 Hz, 1H),

4.27 – 4.17 (m, 4H), 4.11 – 4.02 (m, 1H), 3.50 (s, 3H), 2.86 – 2.71 (m, 2H), 2.60 – 2.41 (m, 2H), 1.29 – 1.21 (m, 6H). ^{13}C NMR (100 MHz, $CDCl_3$) δ 201.8, 173.3, 165.7 (t, *J* = 25.7 Hz), 165.7 (t, *J* = 25.0 Hz), 146.9, 136.8, 133.5, 132.4, 129.2, 128.8, 128.3, 124.5 (t, *J* = 3.0 Hz), 118.4, 113.6, 94.0 (t, *J* = 198.1 Hz), 62.6, 57.5, 52.1, 47.6, 37.3 (t, *J* = 21.2 Hz), 32.4, 13.9. ^{19}F NMR (376 MHz, $CDCl_3$) δ -166.2 (t, *J* = 23.6 Hz). HRMS (ESI+) Calcd. For $C_{28}H_{32}FNO_7Na^+$ ($[M+Na]^+$): 536.2055, found: 536.2057. The product was analyzed by HPLC to determine the enantiomeric excess: 92% *ee* (Chiralpak IE, *i*-propanol/hexane = 10/90, flow rate 1.0 mL/min, λ = 254 nm); t_r = 45.35 and 81.99 min.

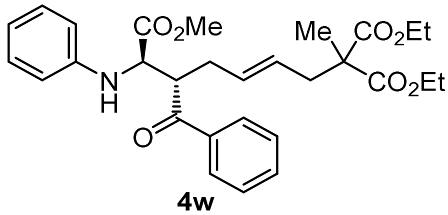

4w

7,7-diethyl 1-methyl (1*R*,2*S*,*E*)-2-benzoyl-1-(phenylamino)oct-4-ene-1,7,7-tricarboxylate (**4w**):

yield (81.5 mg, 40%); *E*/*Z* > 20:1 was determined by HPLC; yellow oil; $[\alpha]_D^{28}$ = -6.5 (*c* 0.2, acetone); ^{1}H NMR (400 MHz, $CDCl_3$) δ 7.96 – 7.84 (m, 2H), 7.63 – 7.55 (m, 1H), 7.52 – 7.45 (m, 2H), 7.20 – 7.13 (m, 2H), 6.77 – 6.69 (m, 1H), 6.67 – 6.60 (m, 2H), 5.54 – 5.43 (m, 1H), 5.41 – 5.26 (m, 1H), 4.39 (d, *J* = 5.3 Hz, 1H), 4.18 – 4.01 (m, 5H), 3.50 (s, 3H), 2.59 – 2.36 (m, 4H), 1.25 (s, 3H), 1.23 – 1.17 (m, 6H). ^{13}C NMR (100 MHz, $CDCl_3$) δ 202.0, 173.5, 171.9, 171.8, 147.0, 136.9, 133.5, 130.5, 129.3, 128.8, 128.4, 128.1, 118.4, 113.6, 61.2, 57.7, 53.4, 52.1, 47.6, 38.7, 32.6, 19.5, 14.0. HRMS (ESI+) Calcd. For $C_{29}H_{35}NO_7Na^+$ ($[M+Na]^+$): 532.2305, found: 532.2311. The product was analyzed by HPLC to determine the enantiomeric excess: 91% *ee* (Chiralpak IE, *i*-propanol/hexane = 10/90, flow rate 1.0 mL/min, λ = 254 nm); t_r = 27.78 and 54.78 min.

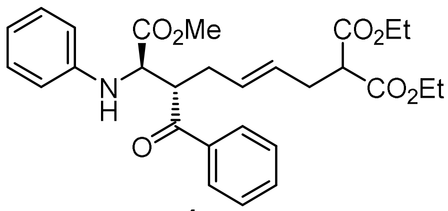

4x

1,1-diethyl 7-methyl (6*S*,7*R*,*E*)-6-benzoyl-7-(phenylamino)hept-3-ene-1,1,7-tricarboxylate (**4x**):

yield (111.3 mg, 56%); *E*/*Z* = 12:1 was determined by HPLC; yellow oil; $[\alpha]_D^{28}$ = -7.1 (*c* 1.1, acetone); ^{1}H NMR (400 MHz, $CDCl_3$) δ 7.97 – 7.87 (m, 2H), 7.63 – 7.54 (m, 1H), 7.52 – 7.44 (m, 2H), 7.21 – 7.12 (m, 2H), 6.75 – 6.69 (m, 1H), 6.68 – 6.61 (m, 2H), 5.55 – 5.47 (m, 1H), 5.46 – 5.36 (m, 1H), 4.39 (d, *J* = 5.2 Hz, 1H), 4.23 – 4.10 (m, 4H), 4.10 – 4.03 (m, 1H), 3.49 (s, 3H), 3.27 (t, *J* = 7.5 Hz, 1H), 2.56 – 2.41 (m, 4H), 1.26 – 1.19 (m, 6H). ^{13}C NMR (100 MHz, $CDCl_3$) δ 202.0, 173.4, 168.7, 147.0, 136.7, 133.4, 129.7, 129.2, 129.0, 128.7, 128.3, 118.3, 113.5, 61.3, 61.3, 57.3, 52.0, 51.7, 47.5, 32.3, 31.5, 14.0. HRMS (ESI+) Calcd. For $C_{28}H_{33}NO_7Na^+$ ($[M+Na]^+$): 518.2149, found: 518.2157. The product was analyzed by HPLC to determine the enantiomeric excess: 83% *ee* (Chiralpak AD-H, *i*-propanol/hexane = 10/90, flow rate 1.0 mL/min, λ = 254 nm); t_r = 26.74 and 40.50 min.

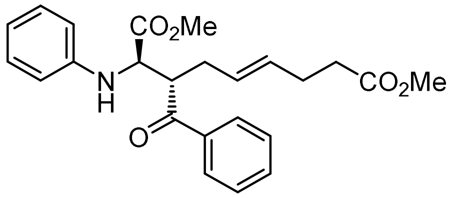

4y

dimethyl (7*S*,8*R*,*E*)-7-benzoyl-8-(phenylamino)non-4-enedioate (**4y**):

yield (79.6 mg, 49%); *E*/*Z* = 6:1 was determined by ^{1}H NMR; yellow oil; $[\alpha]_D^{28}$ = -12.1 (*c* 0.4, acetone); ^{1}H NMR (400 MHz, $CDCl_3$) δ 7.93 – 7.88 (m, 2H), 7.62 – 7.56 (m, 1H), 7.53 – 7.44 (m, 2H), 7.19 – 7.12 (m, 2H), 6.78 – 6.69 (m, 1H), 6.67 – 6.62 (m, 2H), 5.46 – 5.40 (m, 2H), 4.87 (d, *J* = 10.5 Hz, 1H), 4.44 – 4.36 (m, 1H), 4.12 – 4.03 (m, 1H), 3.63 (s, 3H), 3.51 (s, 3H), 2.56 – 2.39 (m, 2H), 2.28 – 2.16 (m, 4H). ^{13}C NMR (100 MHz, $CDCl_3$) δ 202.3, 173.6, 173.4, 147.1, 136.9, 133.5, 132.4, 129.2, 128.8,

128.3, 126.9, 118.4, 118.3, 113.5, 57.5, 52.1, 51.5, 47.7, 33.5, 32.5, 27.7. HRMS (ESI+) Calcd. For $C_{24}H_{28}NO_5^+$ ($[M+H]^+$): 410.1962, found: 410.1964. The product was analyzed by HPLC to determine the enantiomeric excess: 97% *ee* (Chiralpak AD-H, *i*-propanol/hexane = 10/90, flow rate 1.0 mL/min, λ = 254 nm); t_r = 19.86 and 26.26 min.

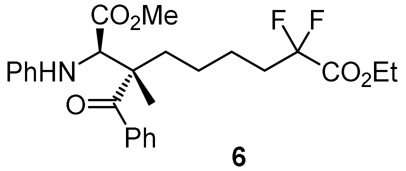

6

1-ethyl 9-methyl (7*S*,8*R*)-7-benzoyl-2,2-difluoro-7-methyl-8-(phenylamino)nonanedioate (**6**):

yield of two steps (85.1 mg, 48%); *dr* = 5.5:1 was determined by ^{1}H NMR; yellow oil; $[\alpha]_D^{28}$ = +6.1 (*c* 0.7, acetone); ^{1}H NMR (400 MHz, $CDCl_3$) δ 7.55 – 7.48 (m, 2H), 7.47 – 7.33 (m, 3H), 7.14 – 7.08 (m, 2H), 6.80 – 6.49 (m, 3H), 4.53 (s, 1H), 4.43 – 4.23 (m, 3H), 3.68 (s, 3H), 2.21 – 1.89 (m, 3H), 1.84 – 1.61 (m, 1H), 1.63 – 1.41 (m, 3H), 1.39 – 1.28 (m, 7H). ^{13}C NMR (100 MHz, $CDCl_3$) δ 207.2, 172.3, 164.2 (t, *J* = 32.7 Hz), 146.5, 139.6, 130.7, 129.2, 128.3, 126.9, 119.4, 116.0 (t, *J* = 248.6 Hz), 114.5, 63.6, 62.8, 53.9, 52.1, 37.2, 34.1 (t, *J* = 23.2Hz), 24.2, 21.9(t, *J* = 4.0 Hz), 18.6, 13.9. ^{19}F NMR (376 MHz, $CDCl_3$) δ -106.1 (t, *J* = 16.5 Hz). HRMS (ESI+) Calcd. For $C_{26}H_{32}F_2NO_5^+$ ($[M+H]^+$): 476.2243, found: 476.2238. The product was analyzed by HPLC to determine the enantiomeric excess: 85% *ee* (Chiralpak AS-H, *i*-propanol/hexane = 5/95, flow rate 1.0 mL/min, λ = 254 nm); t_r = 10.57 and 12.09 min.

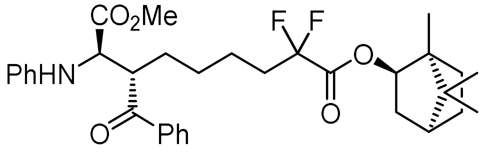

10

9-methyl 1-((1*S*,2*R*,4*S*)-1,7,7-trimethylbicyclo[2.2.1]heptan-2-yl) (7*S*,8*R*)-7-benzoyl-2,2-difluoro-8-(phenylamino)nonanedioate (**10**):

yield of two steps (111.1 mg, 53%); yellow oil; $[\alpha]_D^{28}$ = -15.1 (*c* 0.8, acetone); ^{1}H NMR (400 MHz, $CDCl_3$) δ 7.97 – 7.90 (m, 2H), 7.65 – 7.56 (m, 1H), 7.50 (t, *J* = 7.6 Hz, 2H), 7.20 – 7.12 (m, 2H), 6.77 – 6.71 (m, 1H), 6.69 – 6.61 (m, 2H), 5.04 – 4.95 (m, 1H), 4.75 (s, 1H), 4.41 (d, *J* = 5.8 Hz, 1H), 4.10 – 4.00 (m, 1H), 3.51 (s, 3H), 2.45 – 2.33 (m, 1H), 2.07 – 1.84 (m, 4H), 1.81 – 1.65 (m, 3H), 1.52 – 1.17 (m, 6H), 1.05 – 0.97 (m, 1H), 0.91 (s, 3H), 0.88 (s, 3H), 0.82 (s, 3H). ^{13}C NMR (100 MHz, $CDCl_3$) δ 202.2, 173.3, 164.4 (t, *J* = 32.4 Hz), 146.8, 137.2, 133.6, 129.3, 128.8, 128.2, 118.5, 116.1 (t, *J* = 238.4 Hz), 113.6, 82.8, 58.3, 52.1, 49.0, 47.9, 47.2, 44.7, 36.4, 34.1 (t, *J* = 23.2 Hz), 29.5, 27.9, 26.9, 21.6 (t, *J* = 4.3 Hz), 19.6, 18.7, 13.3. ^{19}F NMR (376 MHz, $CDCl_3$) δ -105.8 (t, *J* = 16.6 Hz). HRMS (ESI+) Calcd. For $C_{33}H_{42}F_2NO_5^+$ ($[M+H]^+$): 570.3026, found: 570.3027. The product was analyzed by HPLC to determine the diastereoisomer ratio: 97:3 *dr* (Chiralpak IA, *i*-propanol/hexane = 10/90, flow rate 1.0 mL/min, λ = 254 nm); t_r = 7.43 and 8.40 min.

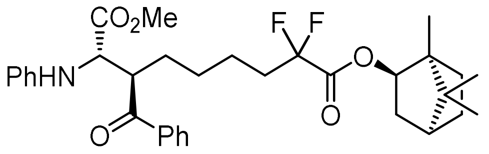

10'

9-methyl 1-((1*S*,2*R*,4*S*)-1,7,7-trimethylbicyclo[2.2.1]heptan-2-yl) (7*R*,8*S*)-7-benzoyl-2,2-difluoro-8-(phenylamino)nonanedioate (**10'**):

yield of two steps (110.1 mg, 45%); yellow oil; $[\alpha]_D^{28}$ = -12.1 (*c* 1.11, acetone); ^{1}H NMR (400 MHz, $CDCl_3$) δ 7.98 – 7.90 (m, 2H), 7.64 – 7.57 (m, 1H), 7.52 – 7.46 (m, 2H), 7.20 – 7.13 (m, 2H), 6.77 – 6.71 (m, 1H), 6.66 – 6.62 (m, 2H), 5.03 – 4.93 (m, 1H), 4.77 – 4.70 (m, 1H), 4.45 – 4.38 (m, 1H), 4.10 – 4.00 (m, 1H), 3.51 (s, 3H), 2.43 – 2.33 (m, 1H), 2.05 – 1.84 (m, 4H), 1.76 – 1.67 (m, 3H), 1.52 – 1.18 (m, 6H), 1.03 – 0.96 (m, 1H), 0.91 (s, 3H), 0.89 (s, 3H), 0.83 (s, 3H). ^{13}C NMR (100 MHz, $CDCl_3$) δ 202.2, 173.3, 164.4 (t, *J* = 32.8 Hz), 146.8, 137.2, 133.6, 129.2, 128.8, 128.2, 118.5, 116.1 (t, *J* = 278.6 Hz), 113.6, 82.8, 58.3, 52.1, 49.0, 47.9, 47.2, 44.7, 36.4, 34.1 (t, *J* = 23.3 Hz), 29.5, 27.9, 26.9, 21.6 (t, *J* = 3.9 Hz), 19.6, 18.7, 13.4. ^{19}F NMR (376 MHz, $CDCl_3$) δ - -105.7 (td, *J* = 16.5, 4.3 Hz). HRMS (ESI+) Calcd. For $C_{33}H_{42}F_2NO_5^+$ ($[M+H]^+$):

570.3026, found: 570.3028. The product was analyzed by HPLC to determine the diastereoisomer ratio: 97:3 *dr* (Chiralpak IA, *i*-propanol/hexane = 10/90, flow rate 1.0 mL/min, λ = 254 nm); t_r = 7.43 and 8.36 min.

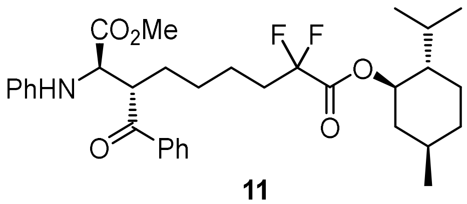

11

1-((1*R*,2*S*,5*R*)-2-isopropyl-5-methylcyclohexyl) 9-methyl (7*S*,8*R*)-7-benzoyl-2,2-difluoro-8-(phenylamino)nonanedioate (**11**):

yield of two steps (101.5 mg, 44%); yellow oil; $[\alpha]^{28}_{D}$ = -28.5 (*c* 1.3, acetone); ^{1}H NMR (400 MHz, $CDCl_3$) δ 7.98 – 7.92 (m, 2H), 7.63 – 7.56 (m, 1H), 7.53 – 7.47 (m, 2H), 7.20 – 7.13 (m, 2H), 6.79 – 6.71 (m, 1H), 6.68 – 6.61 (m, 2H), 4.81 – 4.71 (m, 2H), 4.48 – 4.38 (m, 1H), 4.10 – 4.01 (m, 1H), 3.51 (s, 3H), 2.04 – 1.78 (m, 5H), 1.75 – 1.65 (m, 3H), 1.54 – 1.32 (m, 6H), 1.12 – 1.00 (m, 2H), 0.93 (d, *J* = 6.6 Hz, 3H), 0.88 (d, *J* = 7.0 Hz, 3H), 0.74 (d, *J* = 7.0 Hz, 3H). ^{13}C NMR (100 MHz, $CDCl_3$) δ 202.2, 173.3, 163.7 (t, *J* = 32.5 Hz), 146.8, 137.1, 133.5, 129.2, 128.8, 128.2, 118.5, 116.1 (t, *J* = 248.5 Hz), 113.6, 77.2, 58.3, 52.0, 47.2, 46.6, 40.2, 34.0 (t, *J* = 23.3 Hz), 33.9, 31.3, 29.4, 26.8, 26.1, 23.2, 21.8, 21.5 (t, *J* = 4.3 Hz), 20.5, 16.0. ^{19}F NMR (376 MHz, $CDCl_3$) δ -106.0 (t, *J* = 16.6 Hz). HRMS (ESI+) Calcd. For $C_{33}H_{44}F_2NO_5^+$ ($[M+H]^+$): 572.3182, found: 572.3184. The product was analyzed by HPLC to determine the diastereoisomer ratio: 97:3 *dr* (Chiralpak IA, *i*-propanol/hexane = 10/90, flow rate 1.0 mL/min, λ = 254 nm); t_r = 7.57 and 8.70 min.

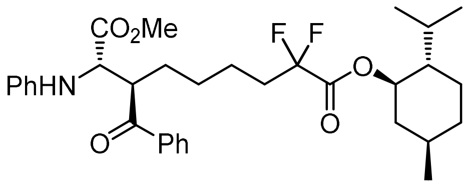

11'

1-((1*R*,2*S*,5*R*)-2-isopropyl-5-methylcyclohexyl) 9-methyl (7*R*,8*S*)-7-benzoyl-2,2-difluoro-8-(phenylamino)nonanedioate (**11'**):

yield of two steps (101.2 mg, 43%); yellow oil; $[\alpha]_D^{28}$ = -23.2 (*c* 1.4, acetone); ^{1}H NMR (400 MHz, $CDCl_3$) δ 7.99 – 7.91 (m, 2H), 7.63 – 7.57 (m, 1H), 7.52 – 7.46 (m, 2H), 7.20 – 7.14 (m, 2H), 6.77 – 6.71 (m, 1H), 6.67 – 6.62 (m, 2H), 4.78 (td, *J* = 10.9, 4.5 Hz, 1H), 4.42 (d, *J* = 5.9 Hz, 1H), 4.09 – 4.01 (m, 1H), 3.51 (s, 3H), 2.04 – 1.80 (m, 5H), 1.75 – 1.64 (m, 3H), 1.55 – 1.32 (m, 6H), 1.12 – 1.01 (m, 2H), 0.93 – 0.87 (m, 7H), 0.75 (d, *J* = 6.9 Hz, 3H). ^{13}C NMR (100 MHz, $CDCl_3$) δ 202.2, 173.3, 163.7 (t, *J* = 32.2 Hz), 146.8, 137.2, 133.6, 129.3, 128.8, 128.2, 118.6, 116.1 (t, *J* = 275.8 Hz), 113.6, 77.3, 58.4, 52.0, 47.2, 46.6, 40.2, 34.0 (t, *J* = 23.1 Hz), 33.8, 31.3, 29.4, 26.9, 26.1, 23.2, 21.8, 21.5 (t, *J* = 4.3 Hz), 20.5, 16.0. ^{19}F NMR (376 MHz, $CDCl_3$) δ -105.9 (t, *J* = 16.7 Hz). HRMS (ESI+) Calcd. For $C_{33}H_{44}F_2NO_5^+$ ($[M+H]^+$): 572.3182, found: 572.3186. The product was analyzed by HPLC to determine the diastereoisomer ratio: 97:3 *dr* (Chiralpak IA, *i*-propanol/hexane = 10/90, flow rate 1.0 mL/min, λ = 254 nm); t_r = 7.62 and 8.68 min.

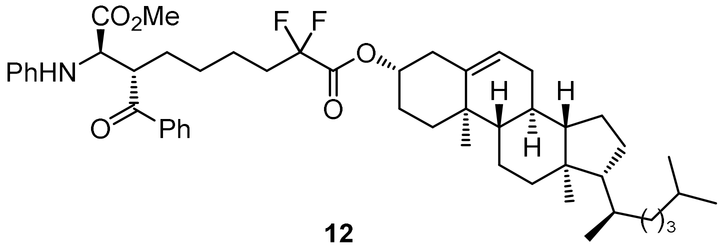

12

1-((3*S*,8*S*,9*S*,10*R*,13*R*,14*S*,17*R*)-10,13-dimethyl-17-((*R*)-6-methylheptan-2-yl)-2,3,4,7,8,9,10,11,12,13,14,15,16,17-tetradecahydro-1*H*-cyclopenta[*a*]phenanthren-3-yl) 9-methyl (7*S*,8*R*)-7-benzoyl-2,2-difluoro-8-(phenylamino)nonanedioate (**12**): yield of two steps (77.5 mg, 44%); yellow oil; $[\alpha]_D^{28}$ = -10.7 (*c* 0.5, acetone); ^{1}H NMR (400 MHz, $CDCl_3$) δ 7.96 – 7.90 (m, 2H), 7.64 – 7.57 (m, 1H), 7.54 – 7.47 (m, 2H), 7.20 – 7.13 (m, 2H), 6.78 – 6.71 (m, 1H), 6.67 – 6.62 (m, 2H), 5.43 – 5.37 (m, 1H), 4.77 – 4.64 (m, 1H), 4.41 (d, *J* = 5.8 Hz, 1H), 4.08 – 4.00 (m, 1H), 3.51 (s, 3H), 2.42 – 2.28 (m, 2H), 2.06 – 1.78 (m, 8H), 1.77 – 1.62 (m, 2H), 1.58 – 1.43 (m, 8H), 1.40 – 1.27 (m, 6H), 1.26 – 1.07 (m, 9H), 1.02 (s, 3H), 1.00 – 0.95 (m, 2H), 0.92 (d, *J* = 6.5 Hz, 3H), 0.88 (d, *J* = 1.8 Hz, 3H), 0.86 (d, *J* = 1.8 Hz, 3H), 0.68 (s, 3H). ^{13}C NMR (100 MHz, $CDCl_3$) δ 202.3, 173.4, 163.6 (t, *J* = 32.2 Hz), 146.8, 138.8, 137.2, 133.6, 129.3,

128.8, 128.2, 123.4, 118.6, 116.0 (t, J = 235.5 Hz), 113.6, 76.8, 58.4, 56.6, 56.1, 52.1, 49.9, 47.2, 42.3, 39.7, 39.5, 37.6, 36.8, 36.5, 36.1, 35.8, 34.1 (t, J = 22.9 Hz), 31.9, 31.8, 29.5, 28.2, 28.0, 27.4, 26.9, 24.2, 23.8, 22.8, 22.5, 21.5 (t, J = 3.8 Hz), 21.0, 19.2, 18.7, 11.8. ^{19}F NMR (376 MHz, $CDCl_3$) δ -106.1 (t, J = 16.7 Hz). HRMS (ESI+) Calcd. For $C_{50}H_{69}F_2NO_5Na^+$ ($[M+Na]^+$): 824.5036, found: 824.5030. The product was analyzed by HPLC to determine the diastereoisomer ratio: 93:7 *dr* (Chiralcel OD-H, *i*-propanol/hexane = 5/95, flow rate 1.0 mL/min, λ = 254 nm); t_r = 7.13 and 8.69 min.

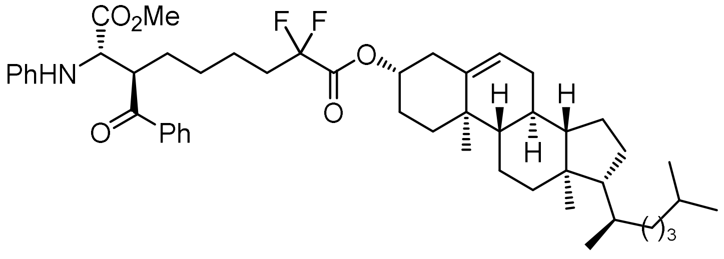

12'

1-((3*S*,8*S*,9*S*,10*R*,13*R*,14*S*,17*R*)-10,13-dimethyl-17-((*R*)-6-methylheptan-2-yl)-2,3,4,7,8,9,10,11,12,13,14,15,16,17-tetradecahydro-1*H*-cyclopenta[*a*]phenanthren-3-yl) 9-methyl (7*R*,8*S*)-7-benzoyl-2,2-difluoro-8-(phenylamino)nonanedioate (**12'**): yield of two steps (76.4 mg, 42%); yellow oil; $[\alpha]_D^{28}$ = -12.8 (*c* 0.8, acetone); ^{1}H NMR (400 MHz, $CDCl_3$) δ 7.97 – 7.90 (m, 2H), 7.65 – 7.55 (m, 1H), 7.53 – 7.46 (m, 2H), 7.21 – 7.12 (m, 2H), 6.78 – 6.70 (m, 1H), 6.65 – 6.61 (m, 2H), 5.43 – 5.37 (m, 1H), 4.76 – 4.65 (m, 1H), 4.41 (d, J = 5.8 Hz, 1H), 4.09 – 3.98 (m, 1H), 3.51 (s, 3H), 2.42 – 2.31 (m, 2H), 2.07 – 1.79 (m, 8H), 1.75 – 1.63 (m, 2H), 1.61 – 1.41 (m, 8H), 1.40 – 1.24 (m, 6H), 1.22 – 1.05 (m, 8H), 1.02 (s, 3H), 1.01 – 0.94 (m, 3H), 0.92 (d, J = 6.5 Hz, 3H), 0.88 (d, J = 1.8 Hz, 3H), 0.86 (d, J = 1.8 Hz, 3H), 0.68 (s, 3H). ^{13}C NMR (100 MHz, $CDCl_3$) δ 202.3, 173.4, 163.6 (t, J = 32.8 Hz), 146.8, 138.8, 137.2, 133.6, 129.3, 128.8, 128.2, 123.4, 118.6, 116.0 (t, J = 249.0 Hz), 113.6, 76.8, 58.4, 56.6, 56.1, 52.1, 49.9, 47.2, 42.3, 39.7, 39.5, 37.6, 36.8, 36.5, 36.2, 35.8, 34.1 (t, J = 23.1 Hz), 31.9, 31.8, 29.5, 28.2, 28.0, 27.4, 26.9, 24.2, 23.8, 22.8, 22.5, 21.5 (t, J = 3.2 Hz), 21.0, 19.2, 18.7, 11.8. ^{19}F NMR (376 MHz, $CDCl_3$) δ -106.1 (t, J = 16.8 Hz). HRMS (ESI+) Calcd. For $C_{50}H_{69}F_2NO_5Na^+$ ($[M+Na]^+$): 824.5036, found: 824.5031. The product was analyzed

by HPLC to determine the diastereoisomer ratio: 90:10 *dr* (Chiralcel OD-H, *i*-propanol/hexane = 5/95, flow rate 1.0 mL/min, λ = 254 nm); t_r = 7.21 and 8.94 min.

VI Synthetic Transformations

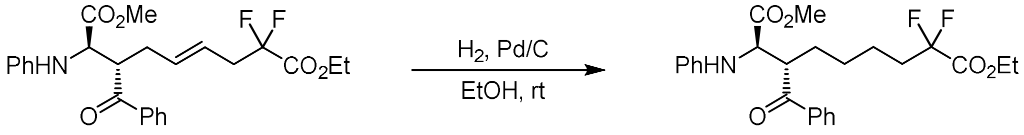

4a, 7:1 *E/Z*, 92% ee **13**, 85% yield, >20:1 dr, 92% ee

Pd/C (palladium on activated carbon, 10% Pd basis, 10 mol %) was added to a solution of **4a** (0.2 mmol) in anhydrous EtOH (2 mL). The reaction mixture was stirred under H_2 atmosphere (1 atm) at room temperature for about 4 h. After the reaction was completed, the crude reaction mixture was filtrated with celite and washed with DCM. The solvent was removed under reduced pressure, then the residue was purified by a flash column chromatography (PE/EA = 10/1) to afford the product **13**.

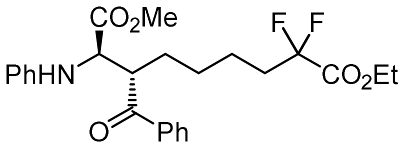

13

1-ethyl 9-methyl (7*S*,8*R*)-7-benzoyl-2,2-difluoro-8-(phenylamino)nonanedioate (**13**): yield (78.4 mg, 85%); *dr* > 20:1; yellow oil; $[\alpha]_D^{28}$ = -5.6 (*c* 0.8, acetone); ^{1}H NMR (400 MHz, $CDCl_3$) δ 7.96 – 7.90 (m, 2H), 7.65 – 7.56 (m, 1H), 7.53 – 7.46 (m, 2H), 7.19 – 7.12 (m, 2H), 6.74 (t, *J* = 7.3 Hz, 1H), 6.66 – 6.61 (m, 2H), 4.72 (s, 1H), 4.41 (d, *J* = 5.8 Hz, 1H), 4.28 (q, *J* = 7.2 Hz, 2H), 4.09 – 3.99 (m, 1H), 3.51 (s, 3H), 2.09 – 1.83 (m, 3H), 1.76 – 1.63 (m, 1H), 1.50 – 1.35 (m, 4H), 1.32 (t, *J* = 7.2 Hz, 3H). ^{13}C NMR (100 MHz, $CDCl_3$) δ 202.3, 173.4, 164.2 (t, *J* = 32.5 Hz), 146.8, 137.2, 133.6, 129.3, 128.8, 128.2, 118.6, 116.0 (t, *J* = 248.8 Hz), 113.6, 62.8, 58.4, 52.1, 47.2, 34.0 (t, *J* = 23.1 Hz), 29.5, 26.9, 21.4, 13.9. ^{19}F NMR (376 MHz, $CDCl_3$) δ -106.04 (t, *J* = 16.9 Hz). HRMS (ESI+) Calcd. For $C_{25}H_{30}F_2NO_5^+$ ($[M+H]^+$): 462.2087, found: 462.2089. The product was analyzed by HPLC to determine the enantiomeric excess: 92% *ee* (Chiralpak AD-H, *i*-propanol/hexane = 10/90, flow rate 1.0 mL/min, λ = 254 nm); t_r = 11.75 and 13.81 min.

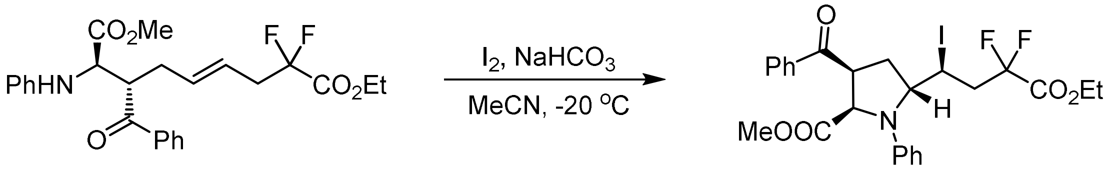

4a, 7:1 *E/Z*, 92% ee **14**, 75% yield, 7:1 dr, 92% ee

I_2 (0.4 mmol) was added to a MeCN (2 mL) solution of **4a** (0.2 mmol) and dry $NaHCO_3$ (0.3 mmol) under argon at -20 °C. The mixture was stirred for 12 h before quenched with addition of DCM (5 mL) and saturated $NaHSO_3$. Extracted with DCM, washed with brine, dried over anhydrous Na_2SO_4, and concentrated, and the residue was purified by flash column chromatography (PE/EA = 8/1) to afford the product **14**.

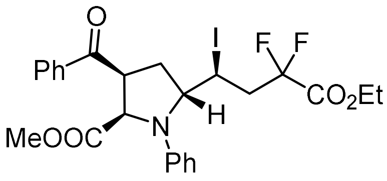

14

methyl (2*R*,3*S*,5*R*)-3-benzoyl-5-((*S*)-4-ethoxy-3,3-difluoro-1-iodo-4-oxobutyl)-1-phenylpyrrolidine-2-carboxylate (**14**):

yield (87.1 mg, 75%); *dr* = 7:1 was determined by crude ^{1}H NMR; yellow oil; $[\alpha]_D^{28}$ = -5.0 (*c* 0.7, acetone); ^{1}H NMR (400 MHz, $CDCl_3$) δ 8.07 – 8.01 (m, 2H), 7.68 – 7.59 (m, 1H), 7.58 – 7.50 (m, 2H), 7.29 – 7.20 (m, 2H), 6.87 – 6.80 (m, 1H), 6.78 – 6.73 (m, 2H), 5.08 – 5.02 (m, 2H), 5.01 – 4.96 (m, 1H), 4.38 (q, *J* = 7.2 Hz, 2H), 3.79 – 3.72 (m, 1H), 3.44 (s, 3H), 3.06 – 2.75 (m, 3H), 2.14 – 2.03 (m, 1H), 1.39 (t, *J* = 7.2 Hz, 3H). ^{13}C NMR (100 MHz, $CDCl_3$) δ 196.8, 171.3, 163.2 (t, *J* = 31.6 Hz), 144.5, 136.6, 133.7, 129.5, 128.9, 128.2, 119.5, 115.2, 115.1 (t, *J* = 251,9 Hz), 65.6, 63.5, 61.4, 51.7, 47.3, 42.5 (t, *J* = 23.1 Hz), 31.0, 28.4, 13.9. ^{19}F NMR (376 MHz, $CDCl_3$) δ -101.0 – -102.0 (m), -104.97 – -105.89 (m). HRMS (ESI+) Calcd. For $C_{25}H_{26}F_2NO_5Na^+$ ($[M+Na]^+$): 608.0716, found: 608.0712. The product was analyzed by HPLC to determine the enantiomeric excess: 92% *ee* (Chiralpak ID, *i*-propanol/hexane = 10/90, flow rate 1.0 mL/min, λ = 254 nm); t_r = 25.83 and 52.97 min.

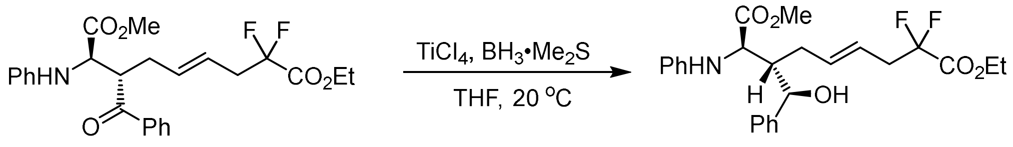

4a, 7:1 E/Z, 92% ee

15, 85% yield, 7:1 E/Z, 92% ee

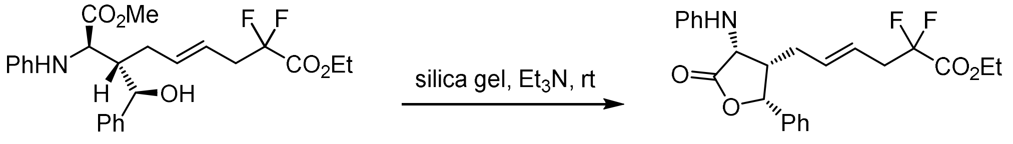

15, 7:1 E/Z, 92% ee

16, 88% yield, 7:1 E/Z, 92% *ee*

a) Under nitrogen atmosphere, to a solution of **4a** (0.2 mmol, 1.0 equiv) in 5.0 mL of dry THF at -40 °C was added $TiCl_4$ (0.3 mmol, 1.5 equiv) to give immediately a yellow solution, which was stirred for 30 min at this temperature. The complex BH_3•Me_2S (0.4 mmol, solution 2.0 M in THF) was then added. After stirring 12 hours at 20 °C, the reaction mixture was quenched with water, extracted with EtOAc, washed with brine, dried over anhydrous Na_2SO_4, and concentrated, and the residue was purified by flash column chromatography (PE/EA = 10/1) to afford the product **15**.

b) **15** was purified by flash column chromatography with 10% Et_3N (PE/EA = 10/1) to afford the product **16**.

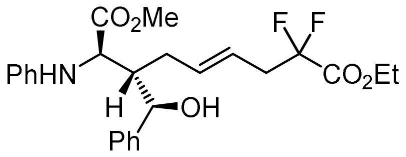

15

1-ethyl 9-methyl (7*S*,8*R*,*E*)-2,2-difluoro-7-((*R*)-hydroxy(phenyl)methyl)-8-(phenylamino)non-4-enedioate (**15**):

yield (78.7 mg, 85%); *E*/*Z* = 7:1 was determined by ^{1}H NMR; yellow oil; $[\alpha]_D^{28}$ = 33.8 (*c* 0.3, acetone); ^{1}H NMR (400 MHz, $CDCl_3$) δ 7.41 – 7.26 (m, 5H), 7.20 – 7.10 (m, 2H), 6.77 – 6.70 (m, 1H), 6.59 – 6.55 (m, 2H), 5.57 – 5.45 (m, 1H), 5.36 – 5.25 (m, 1H), 5.10 – 5.04 (m, 1H), 4.30 – 4.20 (m, 3H), 3.75 (s, 3H), 2.77 – 2.59 (m, 2H), 2.41 – 2.25 (m, 2H), 2.20 – 2.10 (m, 1H), 1.28 (t, *J* = 7.1 Hz, 3H). ^{13}C NMR (100 MHz, $CDCl_3$) δ 174.4, 163.9 (t, *J* = 32.7 Hz), 147.0, 142.6, 135.6, 129.3, 128.4, 127.6, 125.7,

121.2 (t, J = 5.1 Hz), 118.3, 115.2 (t, J = 249.8 Hz), 113.5, 74.0, 62.8, 58.3, 52.3, 47.5, 38.0 (t, J = 23.9 Hz), 27.6, 13.9. ^{19}F NMR (376 MHz, $CDCl_3$) δ -105.5 (t, J = 16.3 Hz). HRMS (ESI+) Calcd. For $C_{25}H_{30}F_2NO_5^+$ ($[M+H]^+$): 462.2087, found: 462.2084. The product was analyzed by HPLC to determine the enantiomeric excess: 92% *ee* (Chiralpak AS-H, *i*-propanol/hexane = 2/98, flow rate 1.0 mL/min, λ = 254 nm); t_r = 34.15 and 70.72 min.

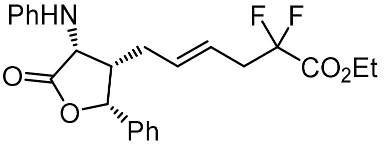

16

ethyl (*E*)-2,2-difluoro-6-((2*R*,3*S*,4*R*)-5-oxo-2-phenyl-4-(phenylamino)tetrahydrofuran-3-yl)hex-4-enoate (**16**):

yield (75.6 mg, 88%); *E*/*Z* = 7:1 was determined by ^{1}H NMR; yellow oil; $[\alpha]_D^{28}$ = -46.0 (*c* 0.2, acetone); ^{1}H NMR (400 MHz, $CDCl_3$) δ 7.46 – 7.37 (m, 2H), 7.39 – 7.30 (m, 3H), 7.26 – 7.20 (m, 2H), 6.87 – 6.77 (m, 1H), 6.72 – 6.65 (m, 2H), 5.72 (d, J = 4.8 Hz, 1H), 5.09 – 5.00 (m, 1H), 5.00 – 4.91 (m, 1H), 4.50 – 4.42 (m, 2H), 4.25 (q, J = 7.2 Hz, 2H), 3.27 – 3.15 (m, 1H), 2.45 – 2.31 (m, 2H), 2.04 – 1.94 (m, 1H), 1.87 – 1.74 (m, 1H), 1.27 (t, J = 7.2 Hz, 3H). ^{13}C NMR (100 MHz, $CDCl_3$) δ 175.1, 163.8 (t, J = 32.1 Hz), 146.3, 134.9, 133.7, 129.5, 128.7, 128.3, 125.2, 120.9 (t, J = 5.6 Hz), 118.8, 115.0 (t, J = 249.5 Hz), 113.0, 80.9, 62.7, 58.7, 46.4, 37.7 (t, J = 24.0 Hz), 26.7, 13.9. ^{19}F NMR (376 MHz, $CDCl_3$) δ -105.5 – -105.7 (m). HRMS (ESI+) Calcd. For $C_{24}H_{26}F_2NO_4^+$ ($[M+H]^+$): 430.1824, found: 430.1823. The product was analyzed by HPLC to determine the enantiomeric excess: 92% *ee* (Chiralpak AS-H, *i*-propanol/hexane = 10/90, flow rate 1.0 mL/min, λ = 254 nm); t_r = 30.05 and 60.90 min.

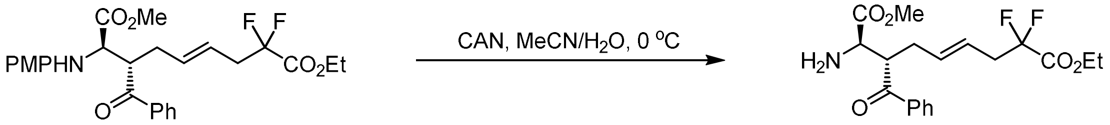

4b, 8:1 E/Z, 91% ee

17, 80% yield, 8:1 E/Z, 90% ee

A solution of **4b** (0.1 mmol) in 3.0 mL of MeCN was cooled to 0 °C and a solution of ammonium cerium nitrate (CAN, 0.22 mmol, in 1.5 mL H_2O) was directly added. The resulting solution was stirred under 0 °C for 30 min. The reaction mixture was quenched with $NaHSO_3$, extracted with EtOAc, washed with brine, dried over anhydrous Na_2SO_4, and concentrated, and the residue was purified by flash column chromatography (PE/EA = 1/1) to afford the product **17**.

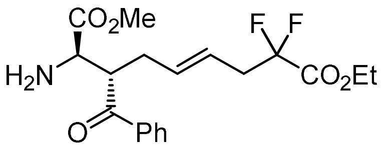

17

1-ethyl 9-methyl (7*S*,8*R*,*E*)-8-amino-7-benzoyl-2,2-difluoronon-4-enedioate (**17**): yield (30.7 mg, 80%); *E*/*Z* = 8:1 was determined by ^{1}H NMR; yellow oil; $[\alpha]_D^{28}$ = 11.5 (*c* 0.3, acetone); ^{1}H NMR (400 MHz, $CDCl_3$) δ 7.93 – 7.86 (m, 2H), 7.61 – 7.53 (m, 1H), 7.47 (t, *J* = 7.6 Hz, 2H), 5.70 – 5.57 (m, 1H), 5.51 – 5.40 (m, 1H), 4.26 (q, *J* = 7.1 Hz, 2H), 3.97 – 3.89 (m, 1H), 3.78 – 3.73 (m, 1H), 3.61 (s, 3H), 2.78 – 2.65 (m, 2H), 2.56 – 2.43 (m, 2H), 1.31 (t, *J* = 7.1 Hz, 3H). ^{13}C NMR (100 MHz, $CDCl_3$) δ 201.8, 174.8, 163.8 (t, *J* = 32.5 Hz), 137.0, 133.7, 133.3, 128.7, 128.3, 121.8 (t, *J* = 5.2 Hz), 115.1 (t, *J* = 249.5 Hz), 62.8, 55.7, 52.1, 48.7, 37.9 (t, *J* = 23.6Hz), 32.4, 13.9. ^{19}F NMR (376 MHz, $CDCl_3$) δ -105.6 (t, *J* = 16.5 Hz). HRMS (ESI+) Calcd. For $C_{19}H_{24}F_2NO_5^+$ ($[M+H]^+$): 384.1617, found: 384.1614. The product was analyzed by HPLC to determine the enantiomeric excess: 90% *ee* (Chiralpak AD-H, *i*-propanol/hexane = 10/90, flow rate 1.0 mL/min, λ = 254 nm); t_r = 17.12 and 18.70 min.

VII Control Experiments

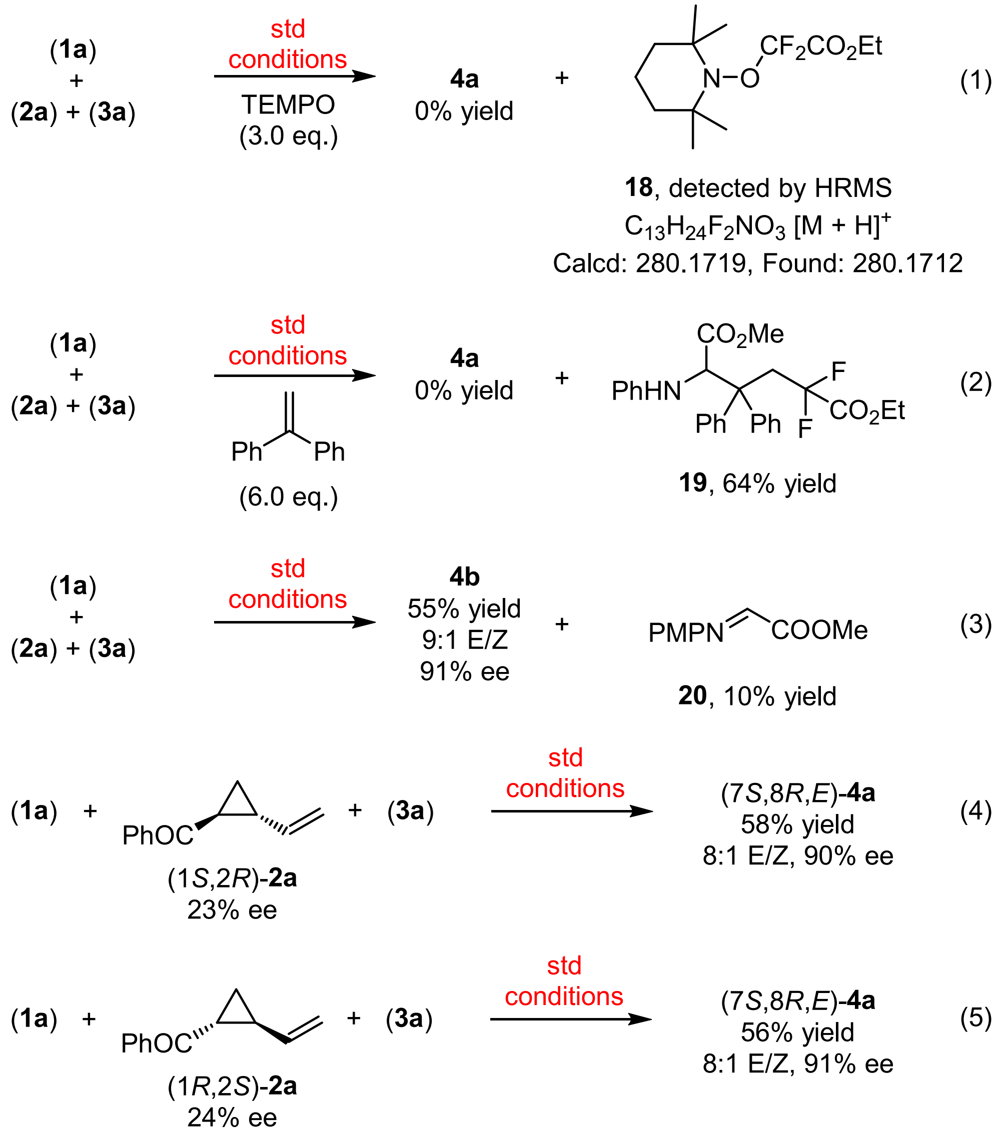

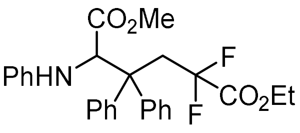

19

1-ethyl 6-methyl 2,2-difluoro-4,4-diphenyl-5-(phenylamino)hexanedioate (**19**): yield (115.2 mg, 64%); yellow oil; ^{1}H NMR (400 MHz, $CDCl_3$) δ 7.37 – 7.15 (m, 12H), 6.79 (t, J = 7.3 Hz, 1H), 6.74 – 6.67 (m, 2H), 5.39 (s, 1H), 3.83 – 3.73 (m, 2H), 3.48 (s, 3H), 3.47 – 3.37 (m, 2H), 1.16 (t, J = 7.2 Hz, 3H). ^{13}C NMR (100 MHz, $CDCl_3$) δ 172.1, 163.0 (t, J = 32.4 Hz), 146.2, 141.4, 141.1, 129.7, 129.4, 129.3, 127.7, 127.6, 127.5, 127.2, 119.2, 115.9 (t, J = 250.0 Hz), 114.3, 62.6, 60.3 (t, J = 3.2 Hz), 51.8, 51.5 (t, J =

2.9 Hz), 42.4 (t, J = 22.9 Hz), 13.5. ^{19}F NMR (376 MHz, $CDCl_3$) δ -94.7 – -96.6 (m), -100.0 – -102.0 (m). HRMS (ESI+) Calcd. For $C_{27}H_{28}F_2NO_4^+$ ($[M+H]^+$): 468.1981, found: 468.1973.

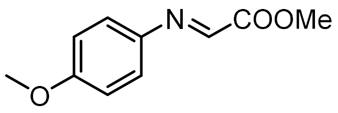

20

methyl (*E*)-2-((4-methoxyphenyl)imino)acetate (**20**): [3]

yield (8.0 mg, 10%); ^1H NMR (400 MHz, $CDCl_3$) δ 7.95 (s, 1H), 7.36 (d, J = 8.9 Hz, 2H), 6.93 (d, J = 9.0 Hz, 2H), 3.94 (s, 3H), 3.84 (s, 3H). ^{13}C NMR (100 MHz, $CDCl_3$) δ 164.1, 160.6, 147.4, 141.1, 123.7, 114.6, 55.5, 52.8.

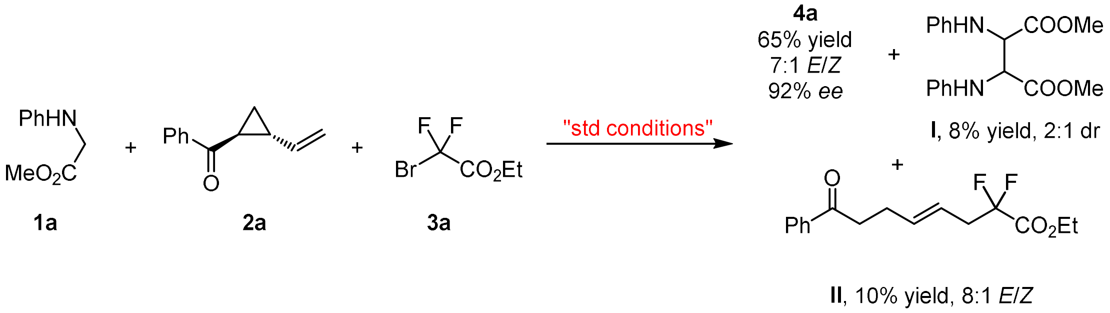

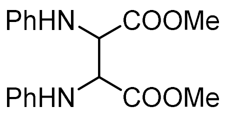

I

dimethyl 2,3-bis(phenylamino)succinate (**I**)[4]

yield (10.5 mg, 8%); *dr* = 2:1; yellow oil; ^1H NMR (400 MHz, $CDCl_3$) δ 7.24 – 7.15 (m, 4H), 6.85 – 6.76 (m, 2H), 6.73 – 6.66 (m, 4H), 4.67 (s, 2H), 4.55 (s, 2H), 3.79 (s, 6H). ^{13}C NMR (100 MHz, $CDCl_3$) δ 171.2, 146.3, 129.4, 119.3, 114.2, 59.1, 52.7.

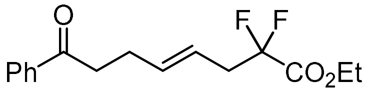

II

ethyl (*E*)-2,2-difluoro-8-oxo-8-phenyloct-4-enoate (**II**)

yield (11.8 mg, 10%); *E*/*Z* = 8:1; white yellow oil; ^{1}H NMR (400 MHz, $CDCl_3$) δ 7.99 – 7.91 (m, 2H), 7.61 – 7.53 (m, 1H), 7.50 – 7.42 (m, 2H), 5.76 (dt, *J* = 15.2, 6.9 Hz, 1H), 5.52 – 5.38 (m, 1H), 4.29 (q, *J* = 7.1 Hz, 2H), 3.05 (t, *J* = 7.3 Hz, 2H), 2.84 – 2.71 (m, 2H), 2.52 – 2.44 (m, 2H), 1.32 (t, *J* = 7.2 Hz, 3H). ^{13}C NMR (100 MHz, $CDCl_3$) δ 199.1, 163.9 (t, *J* = 32.1 Hz), 136.8, 136.5, 133.1, 128.6, 128.0, 119.4 (t, *J* = 5.6 Hz), 115.4 (t, *J* = 248.5 Hz), 62.7, 38.1 (t, *J* = 24.0 Hz), 37.8, 27.0, 14.0. ^{19}F NMR (376 MHz, $CDCl_3$) δ -105.61 (t, *J* = 16.0 Hz). HRMS (ESI+) Calcd. For $C_{16}H_{19}F_2O_3^+$ ($[M+H]^+$): 297.1297, found: 297.1302.

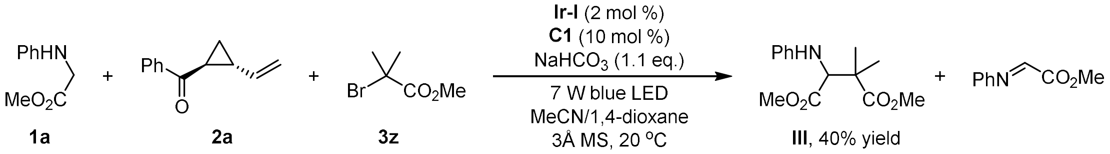

dimethyl 2,2-dimethyl-3-(phenylamino)succinate (**III**):

^{1}H NMR (400 MHz, $CDCl_3$) δ 7.22 – 7.13 (m, 2H), 6.80 – 6.71 (m, 1H), 6.74 – 6.67 (m, 2H), 4.46 (d, *J* = 10.4 Hz, 1H), 4.35 (d, *J* = 10.4 Hz, 1H), 3.71 (s, 3H), 3.68 (s, 3H), 1.31 (s, 3H), 1.27 (s, 3H).

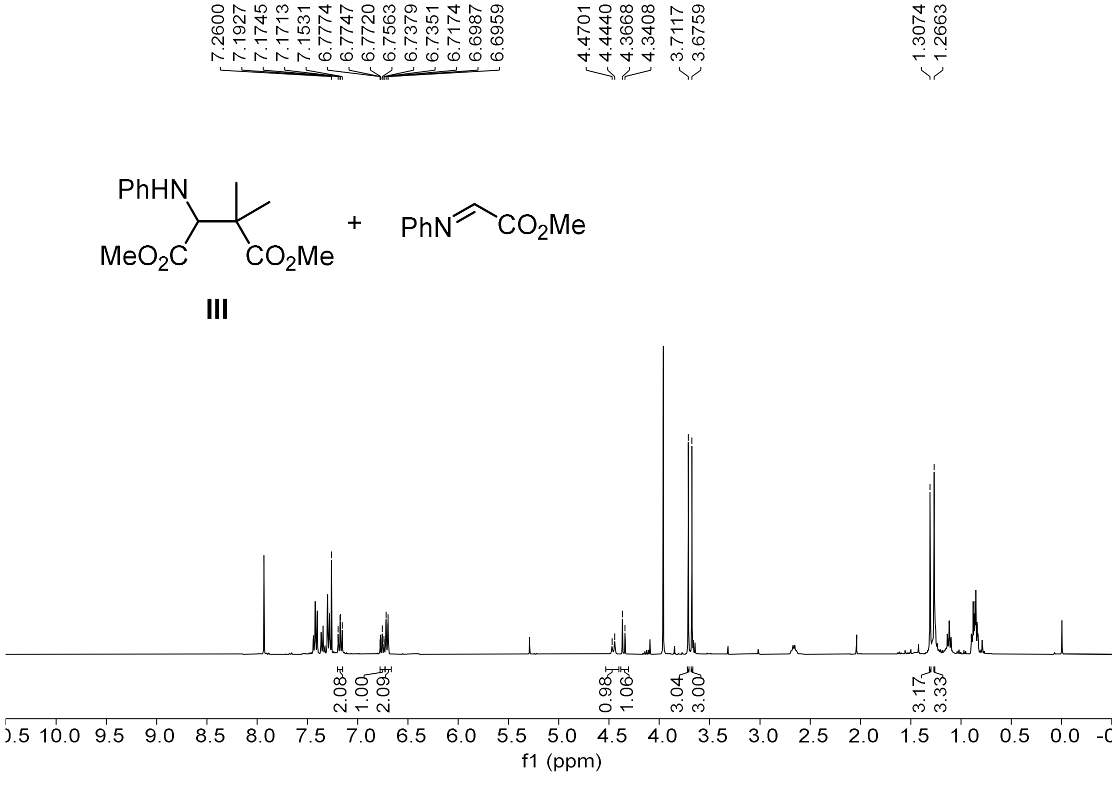

VIII Mechanistic Investigation

Luminescence Quenching Experiments:

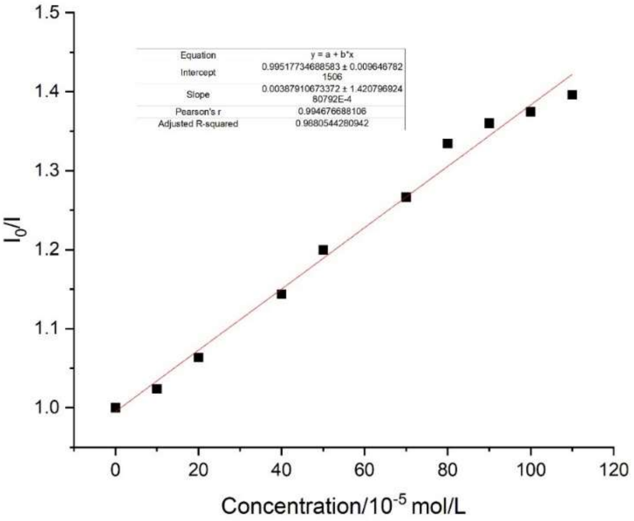

Figure S1. **Ir-I** emission quenching by **1a**

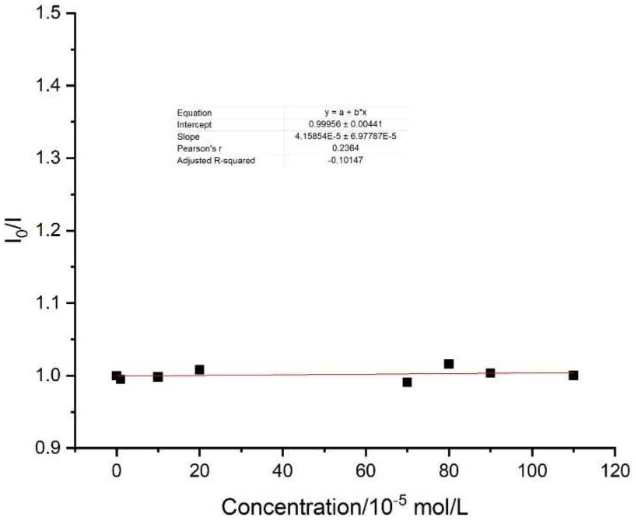

Figure S2. **Ir-I** emission quenching by **2a**

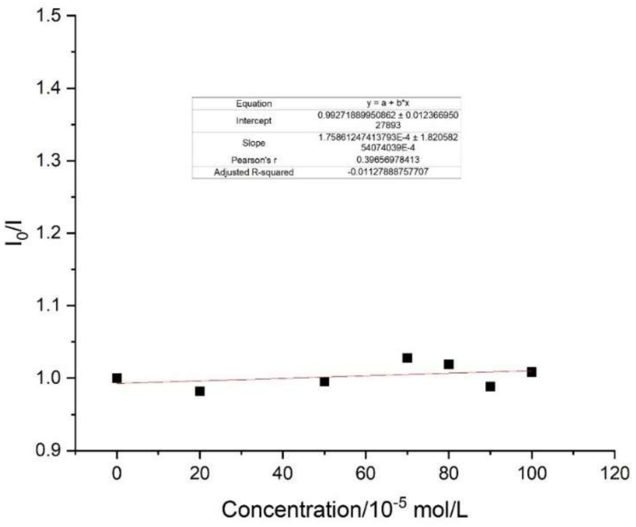

Figure S3. **Ir-I** emission quenching by **3a**

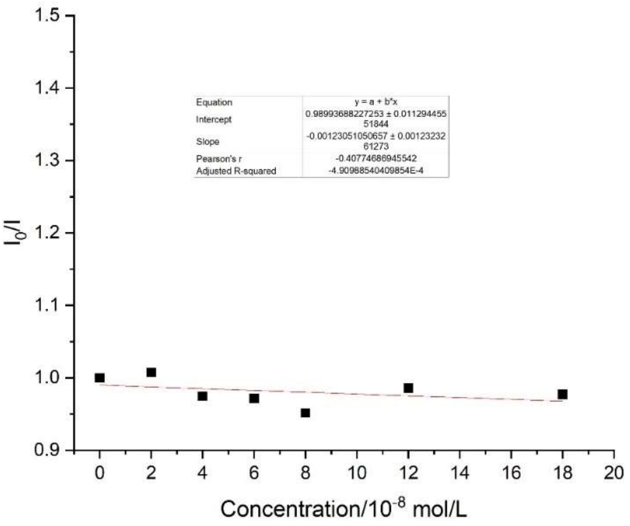

Figure S4. **Ir-I** emission quenching by **C1**

Fluorescence quenching studies were performed on F-4600 Fluorescence Spectrophotometer. Ir[dF(CF_3)ppy]$_2$(dtbbpy)PF_6 solutions were excited at 380 nm and the emission intensity at 478 nm was observed. In a typical experiment, the emission spectrum of a 1×10^{-5} M solution of Ir[dF(CF_3)ppy]$_2$(dtbbpy)PF_6 in 1,4-dioxane/MeCN (1:1 volume ratio) was collected. As shown in n Figure **S1** and Figure **S2** as well as **S3**, both substrate **1a** and **3a** could quench the excited state of Ir[dF(CF_3)ppy]$_2$(dtbbpy)PF_6. However, the quenching rate constant of **1a** is much larger than that of **3a**. It might support our hypothesis on the initiation of this enantioselective radical reaction through

reductive quenching of the excited state of the photocatalyst by **1a**.

IX. Reference

[1] a) R. K. Kunz, D. W. C. MacMillan, *J. Am. Chem. Soc.* **2005**, *127*, 3240-3241; b) A. Hartikka, P. I. Arvidsson, *J. Org. Chem.* **2007**, 72, 5874-5877; c) J. Lv, Y. Nong, K. Chen, Q. Wang, J. Jin, T. Li, Z. Jin, Y. R. Chi, *Chin. Chem. Lett.* **2023**, *34*, 107570.

[2] M. E. Jung, D. L. Sun, T. A. Dwight, P. Yu, W. Li, K. N. Houk, *Org. Lett.* **2016**, *18*, 5138-5141.

[3] M. Kojima, K. Mikami, *Chem. Eur. J.* **2011**, *17*, 13950-13953.

[4] Wang, C.; Yu, Y.; Liu, W.-L.; Duan, W.-L. *Org. Lett.* **2019**, *21*, 9147-9152.

X NMR and HPLC Spectra

^{1}H、^{13}C and ^{19}F NMR Spectra

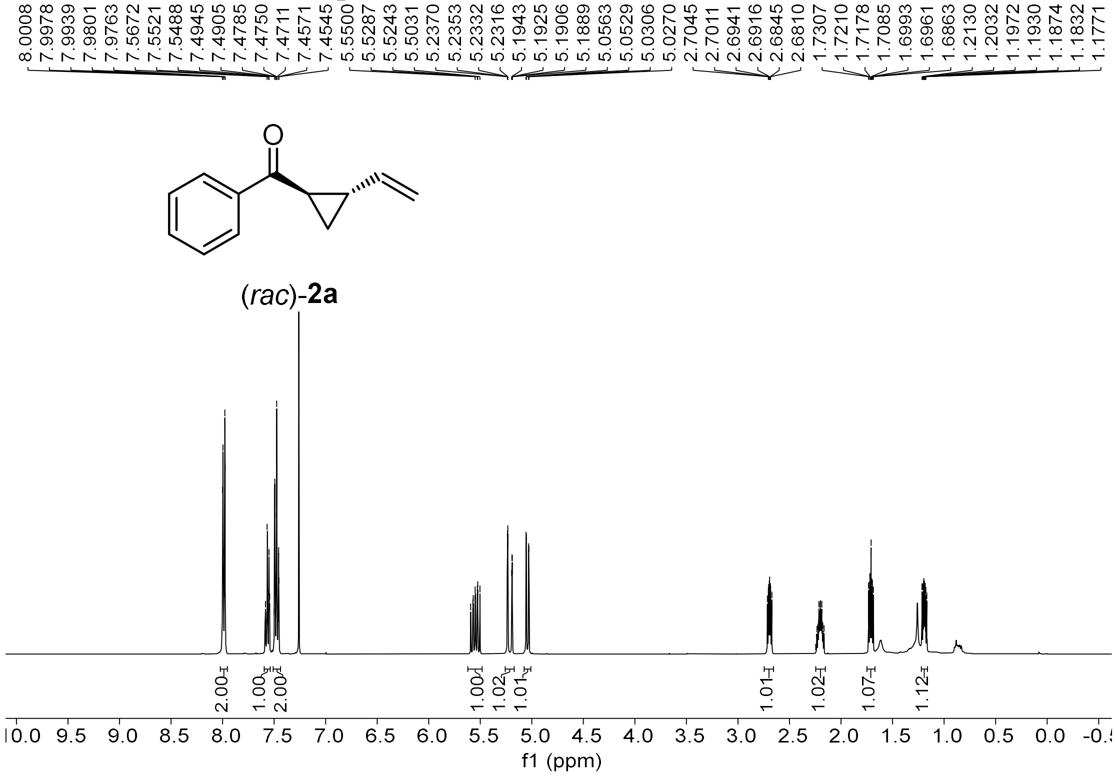

^{1}**H NMR** (400 MHz, $CDCl_3$) of **2a**

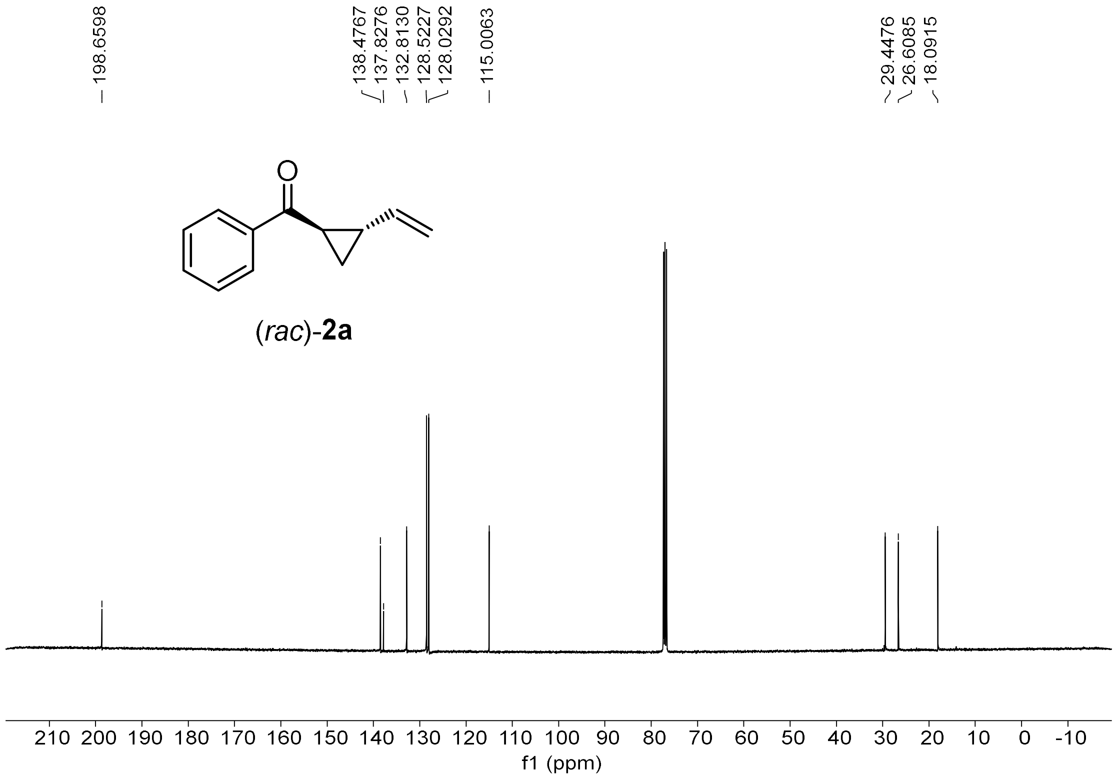

^{13}**C NMR** (100 MHz, $CDCl_3$) of **2a**

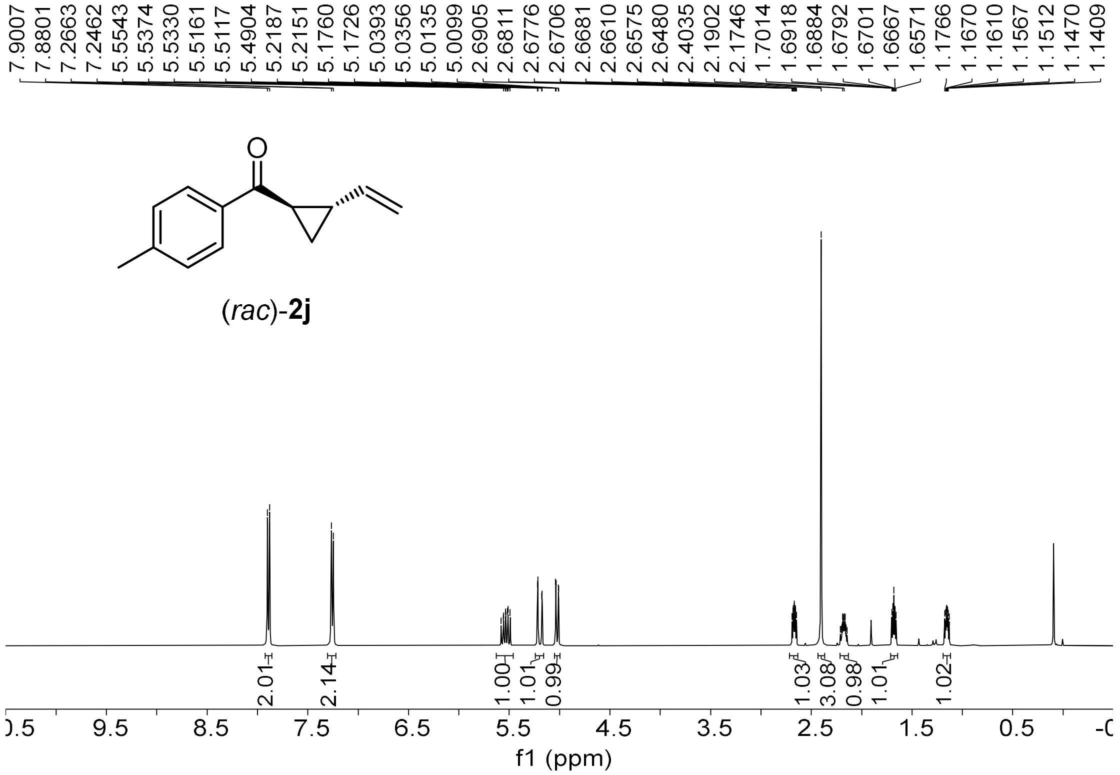

^{1}H NMR (400 MHz, $CDCl_3$) of **2j**

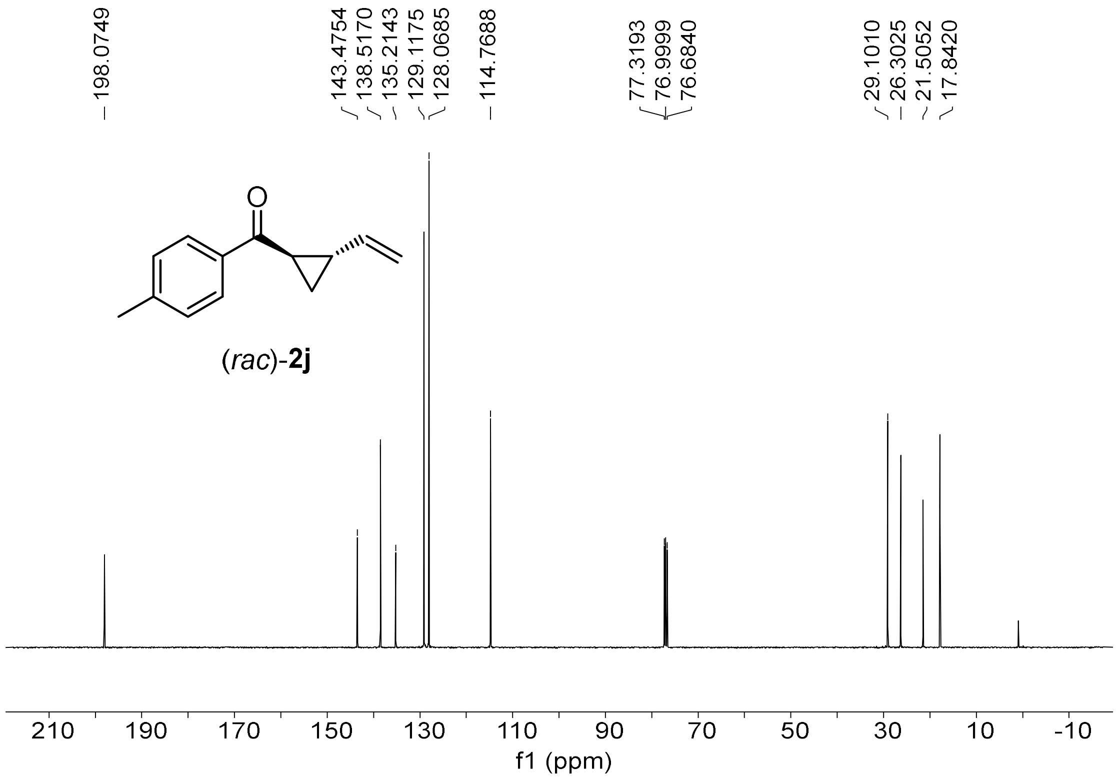

^{13}C NMR (100 MHz, $CDCl_3$) of **2j**

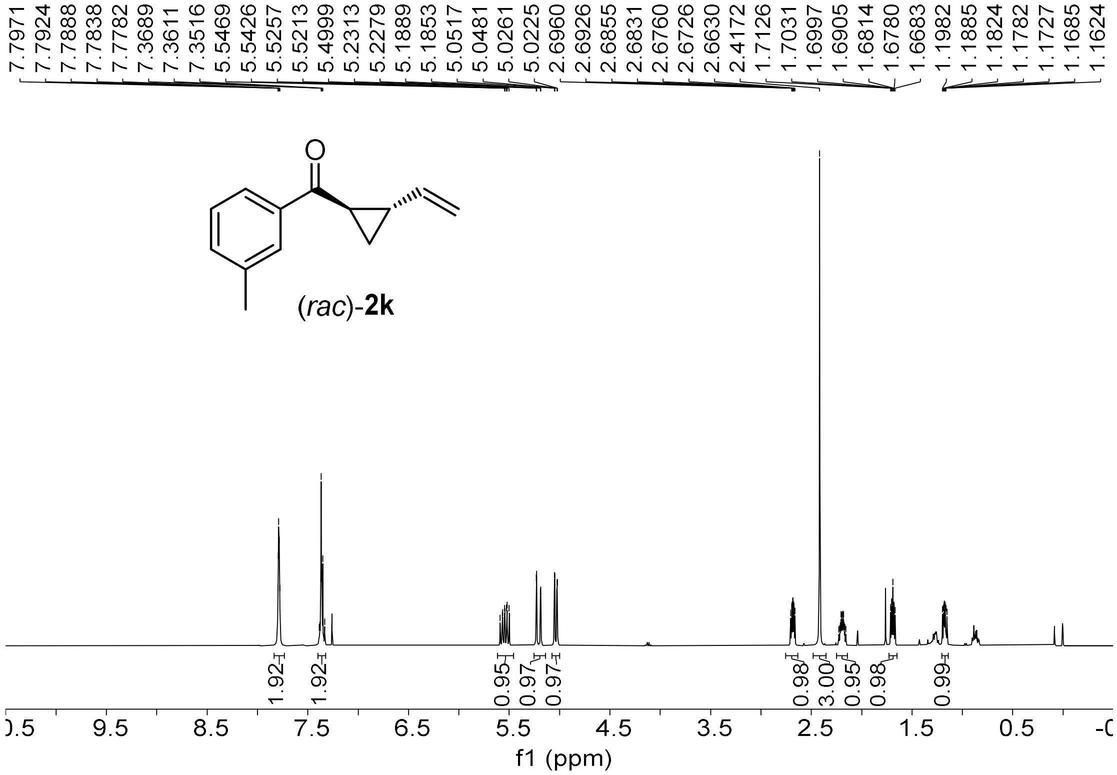

^{1}H NMR (400 MHz, $CDCl_3$) of **2k**

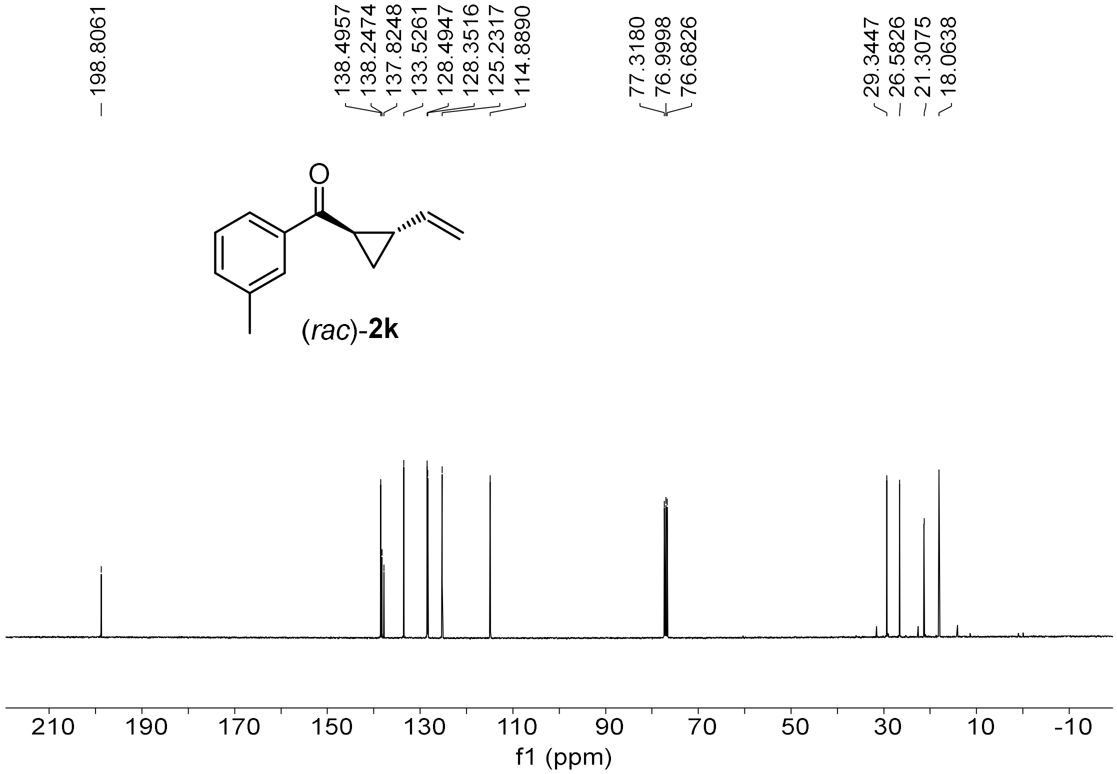

^{13}C NMR (100 MHz, $CDCl_3$) of **2k**

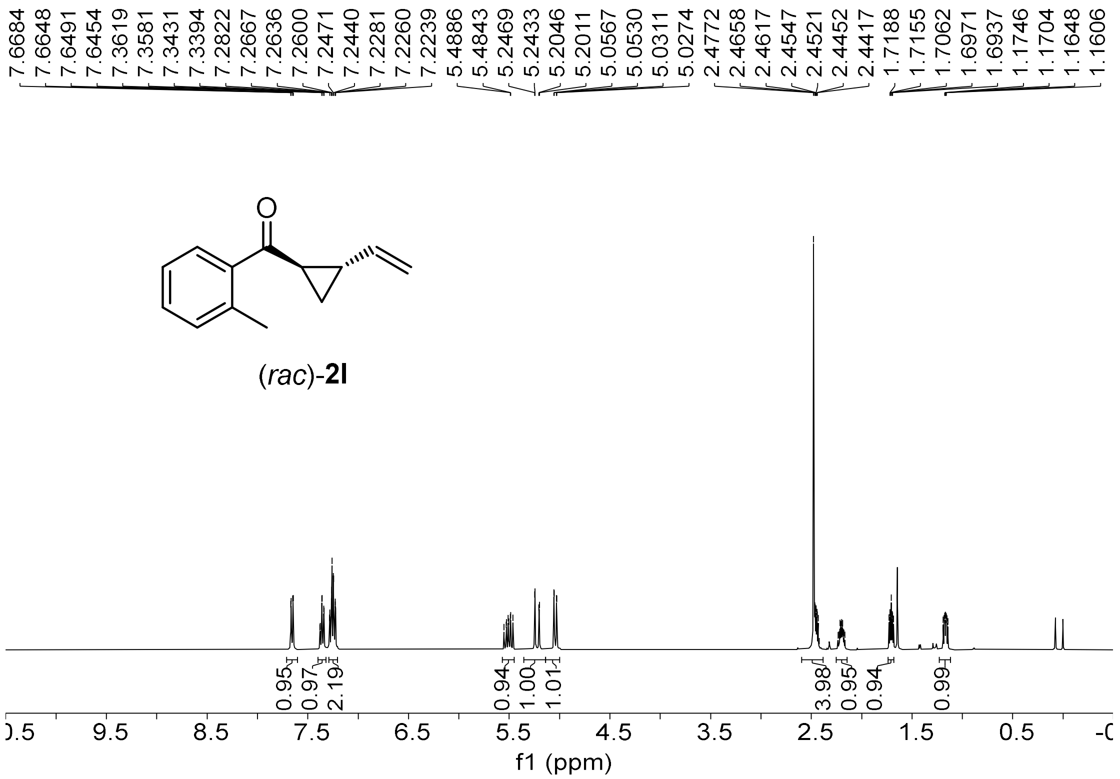

^{1}H NMR (400 MHz, $CDCl_3$) of **2l**

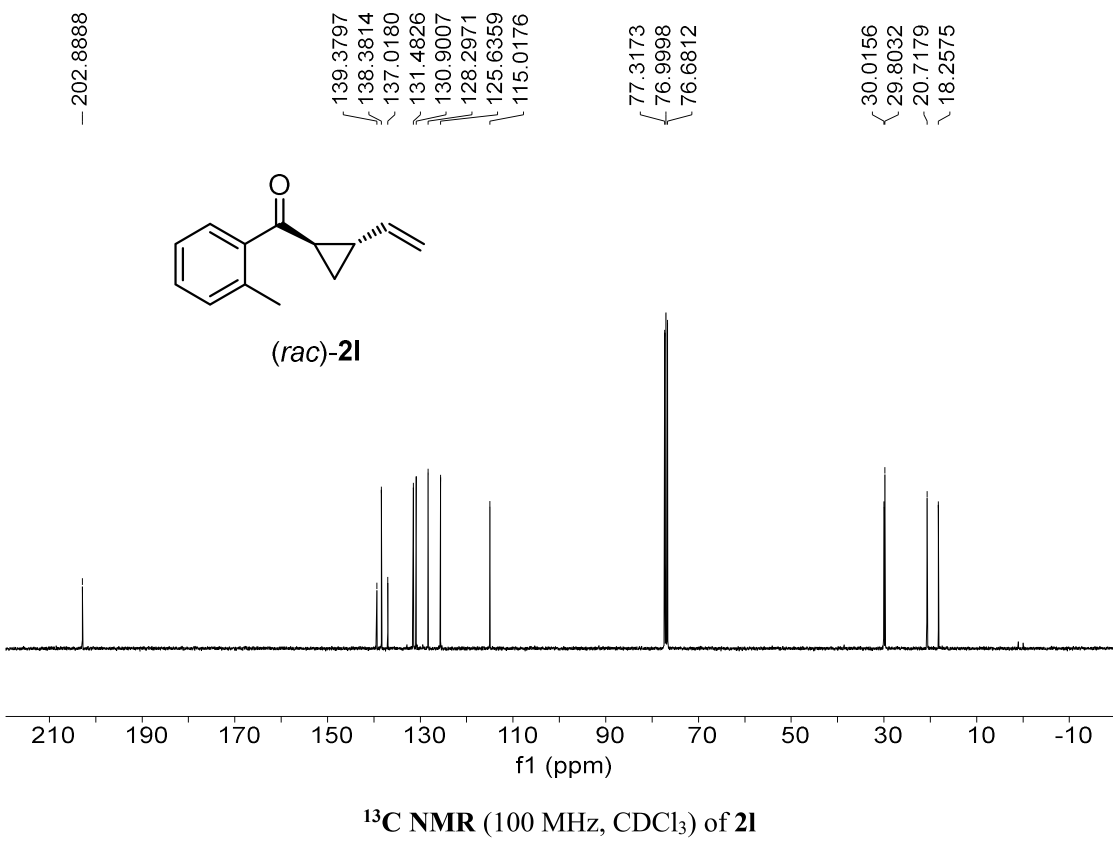

^{13}C NMR (100 MHz, $CDCl_3$) of **2l**

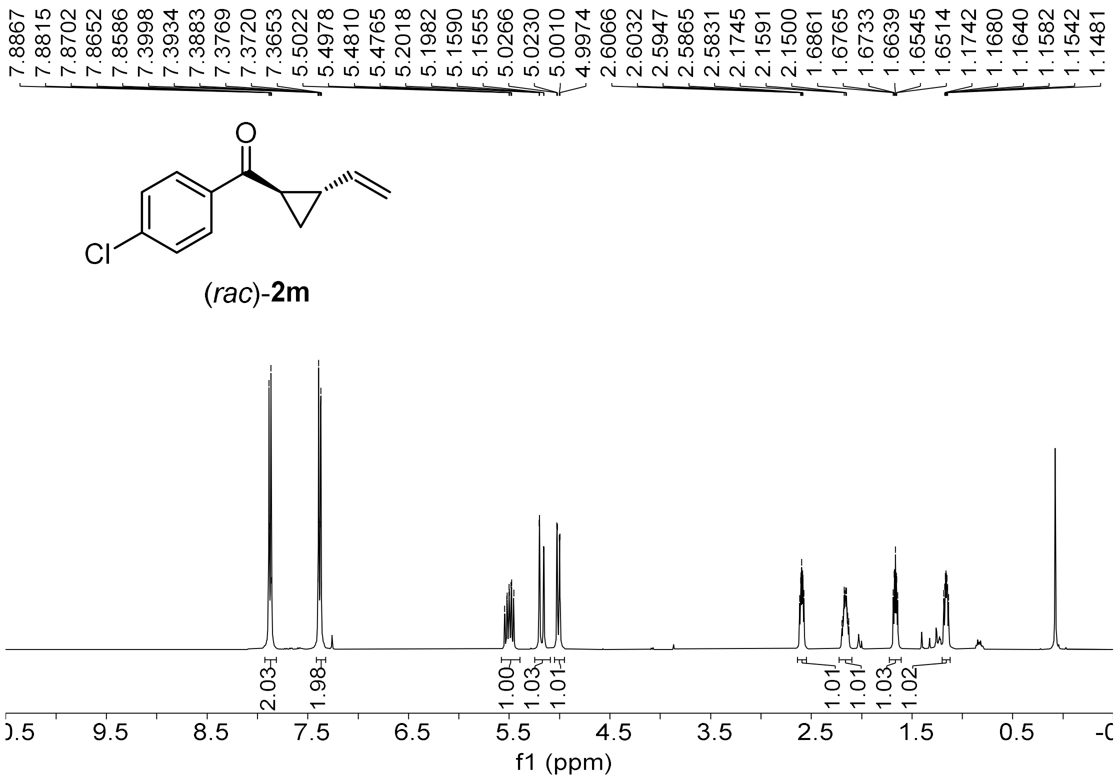

^{1}H NMR (400 MHz, $CDCl_3$) of **2m**

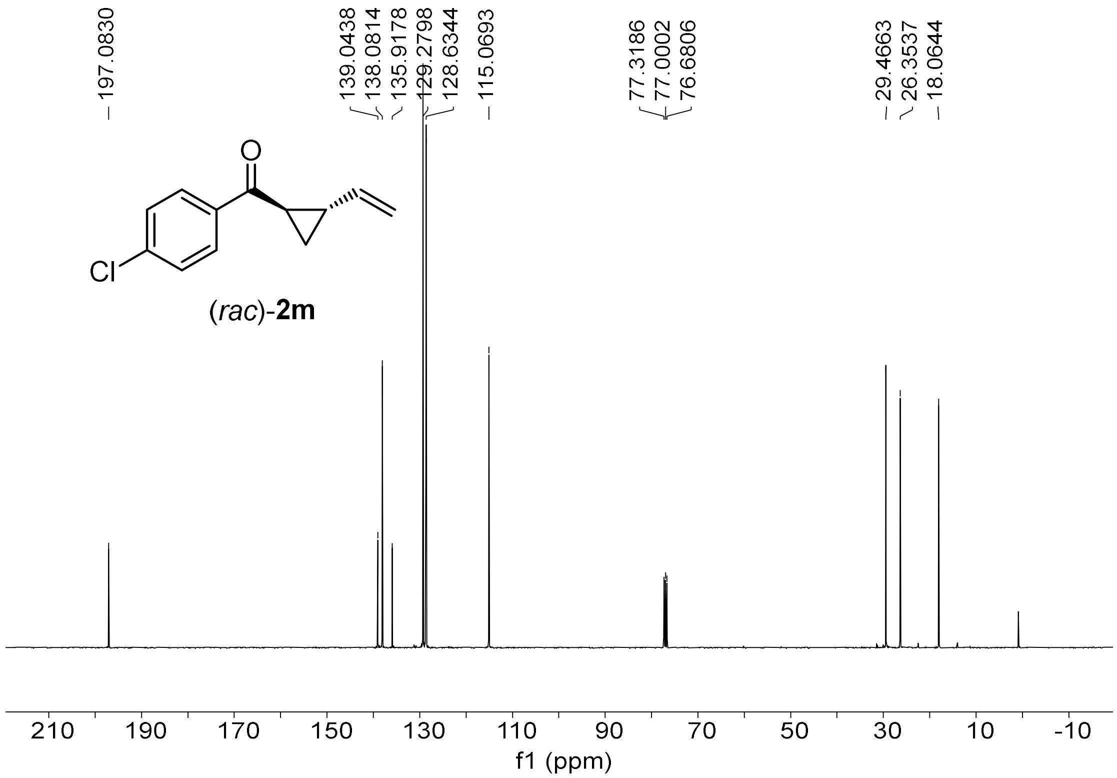

^{13}C NMR (100 MHz, $CDCl_3$) of **2m**

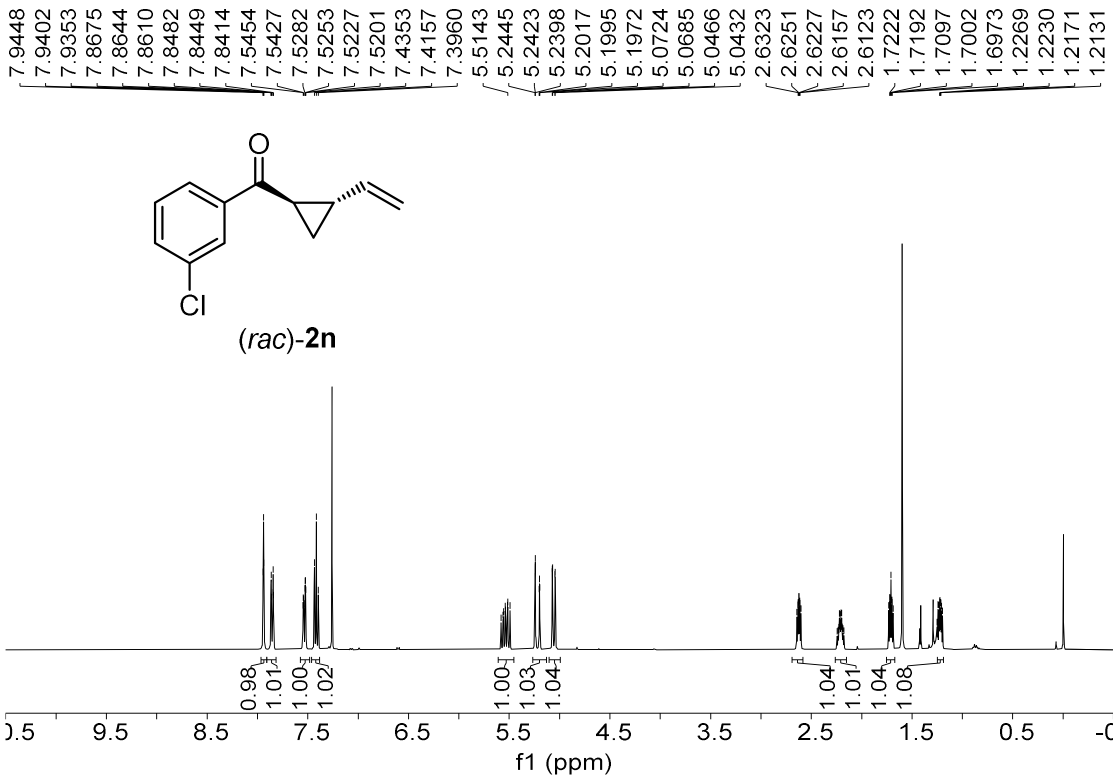

^{1}H NMR (400 MHz, $CDCl_3$) of **2n**

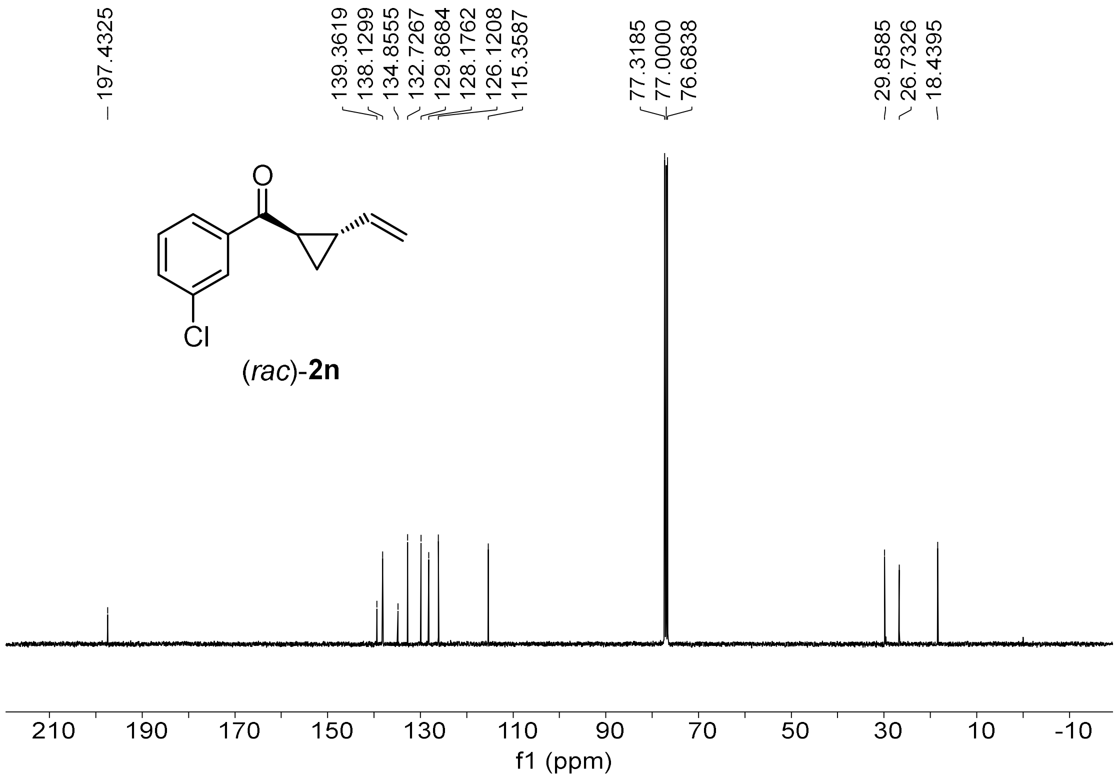

^{13}C NMR (100 MHz, $CDCl_3$) of **2n**

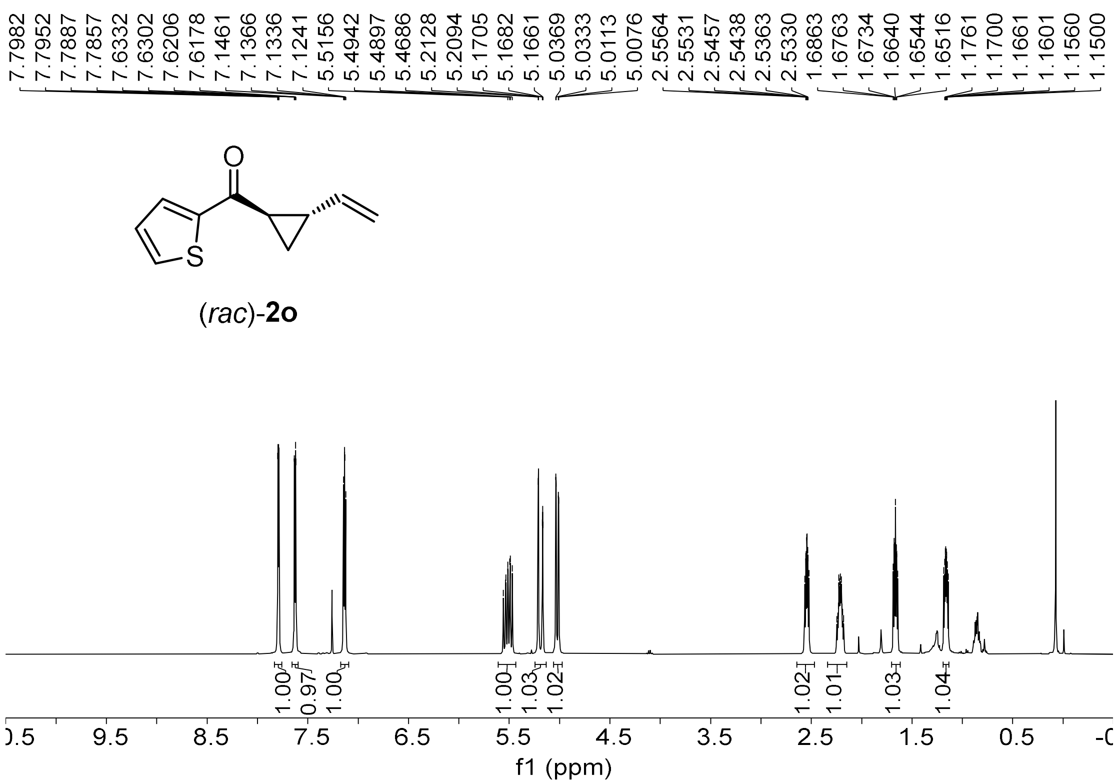

¹H NMR (400 MHz, CDCl$_3$) of **2o**

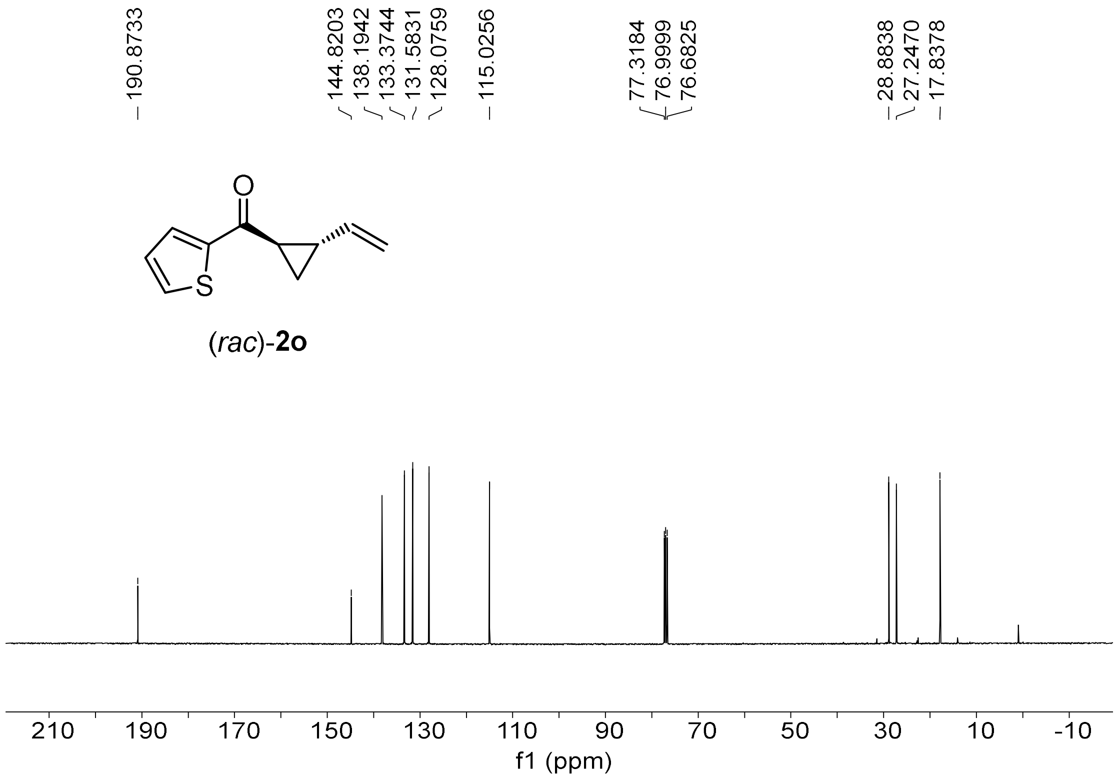

¹³C NMR (100 MHz, CDCl$_3$) of **2o**

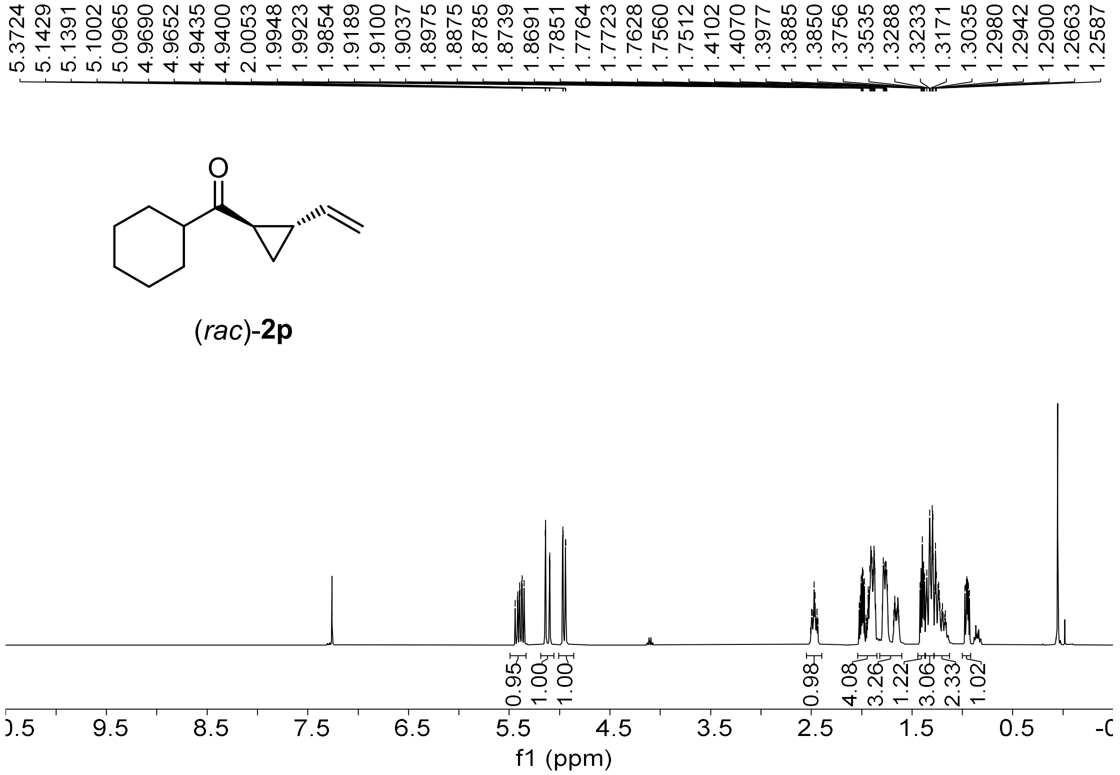

^{1}H NMR (400 MHz, $CDCl_3$) of **2p**

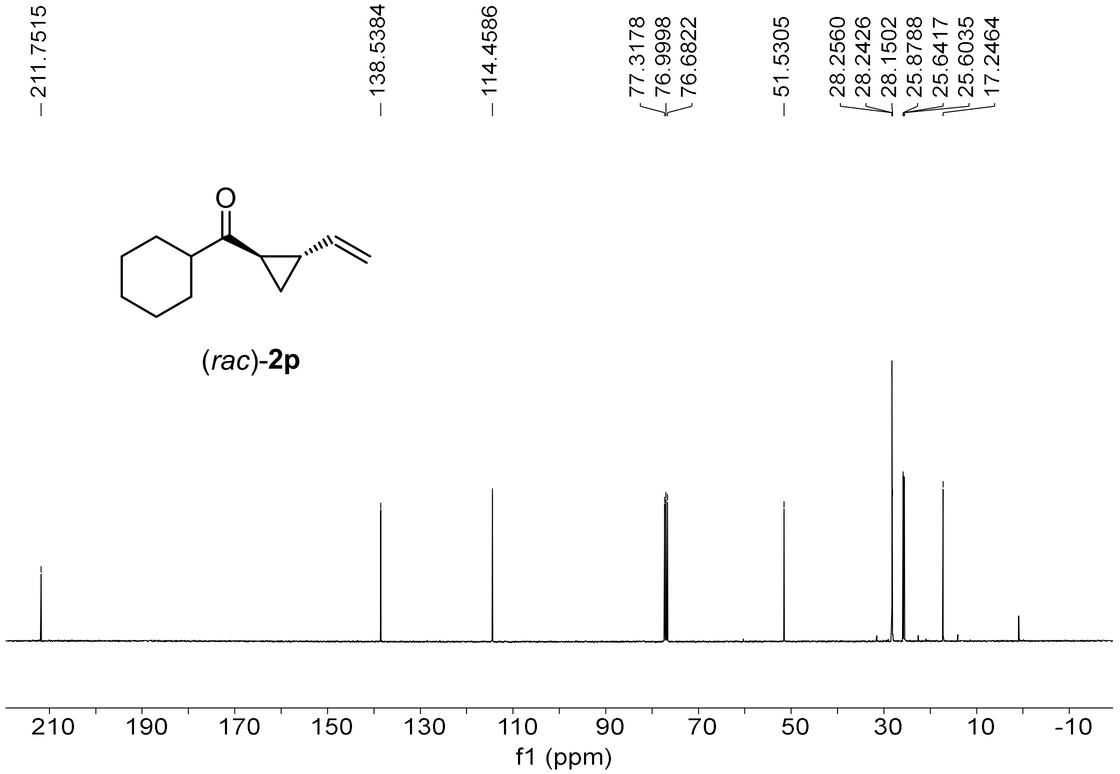

^{13}C NMR (100 MHz, $CDCl_3$) of **2p**

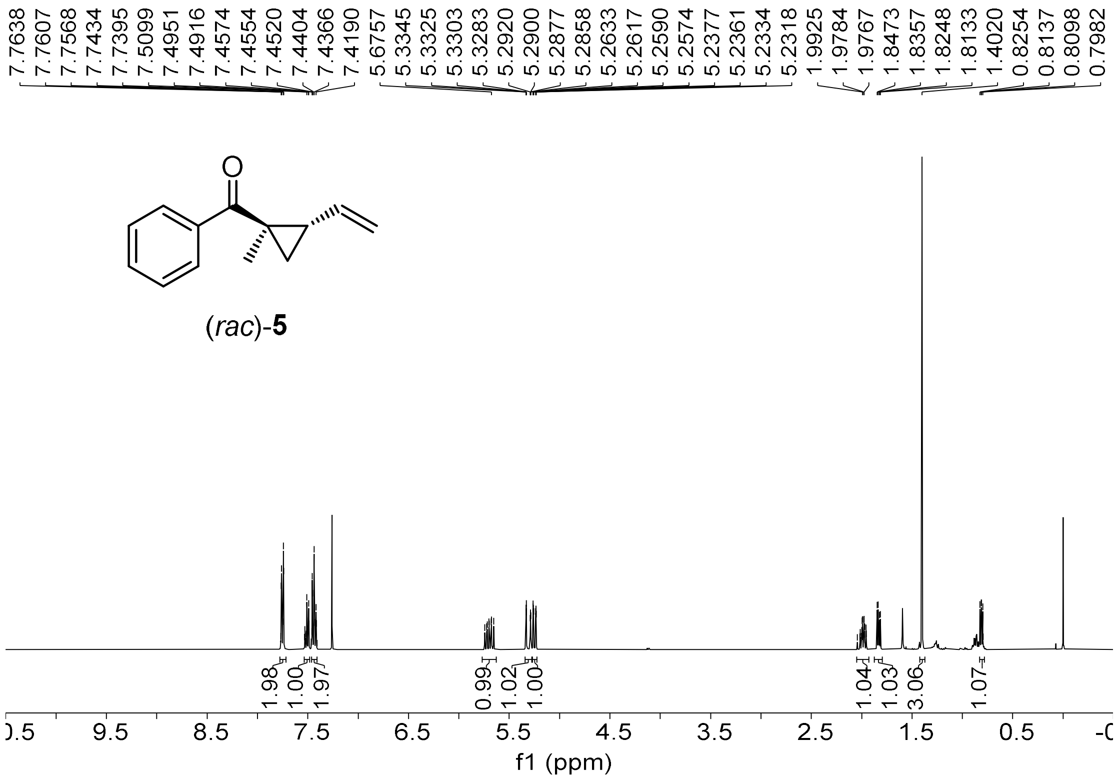

^{1}H NMR (400 MHz, $CDCl_3$) of **5**

NOE 1D-NMR spectrum of compound **5** in $CDCl_3$ and the determination of the relative stereochemistry

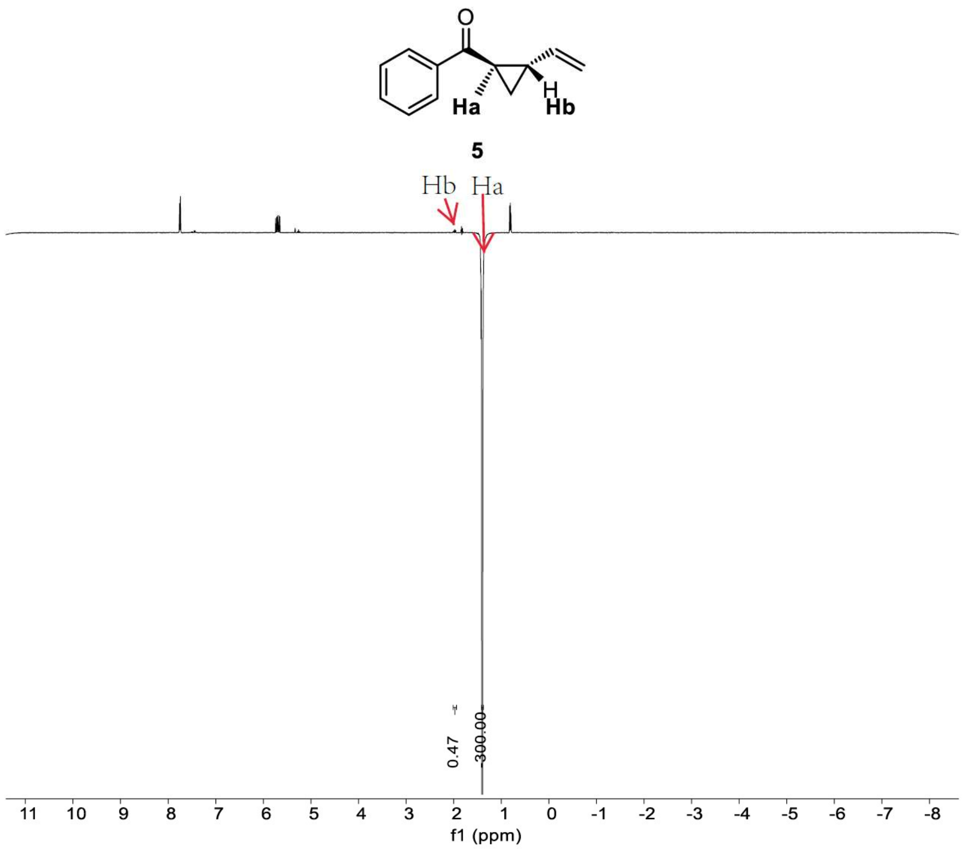

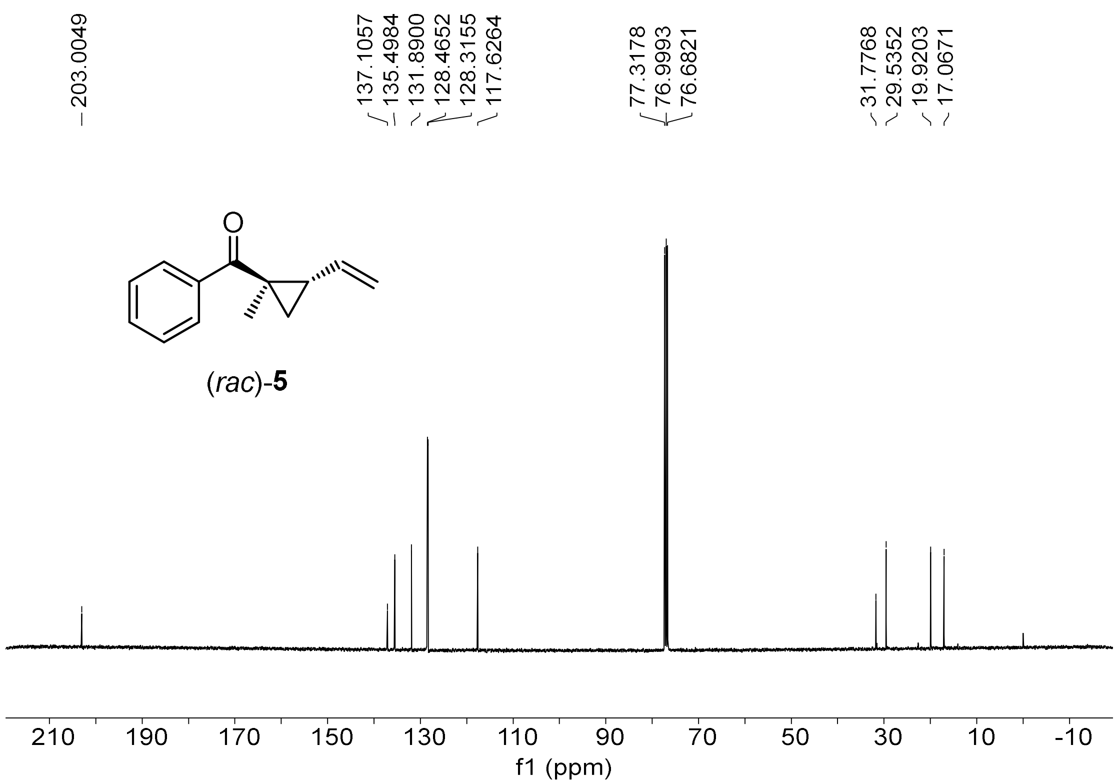

^{13}C NMR (100 MHz, $CDCl_3$) of **5**

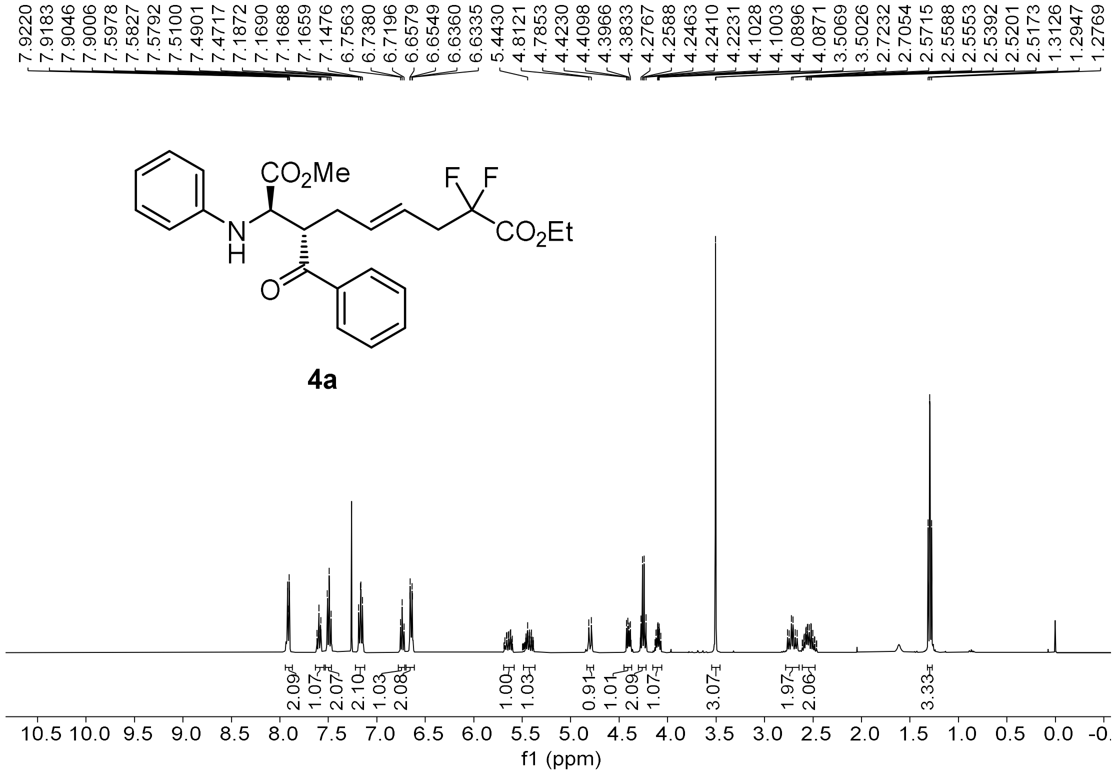

^{1}H NMR (400 MHz, $CDCl_3$) of **4a**

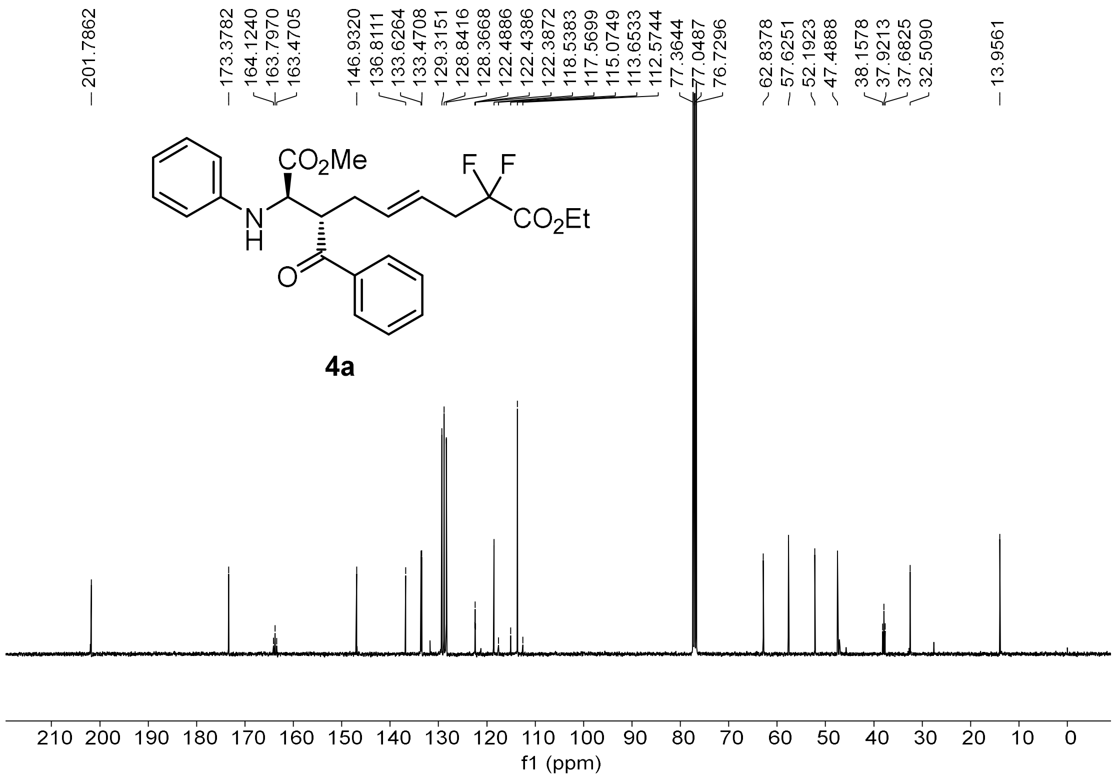

^{13}C NMR (100 MHz, $CDCl_3$) of **4a**

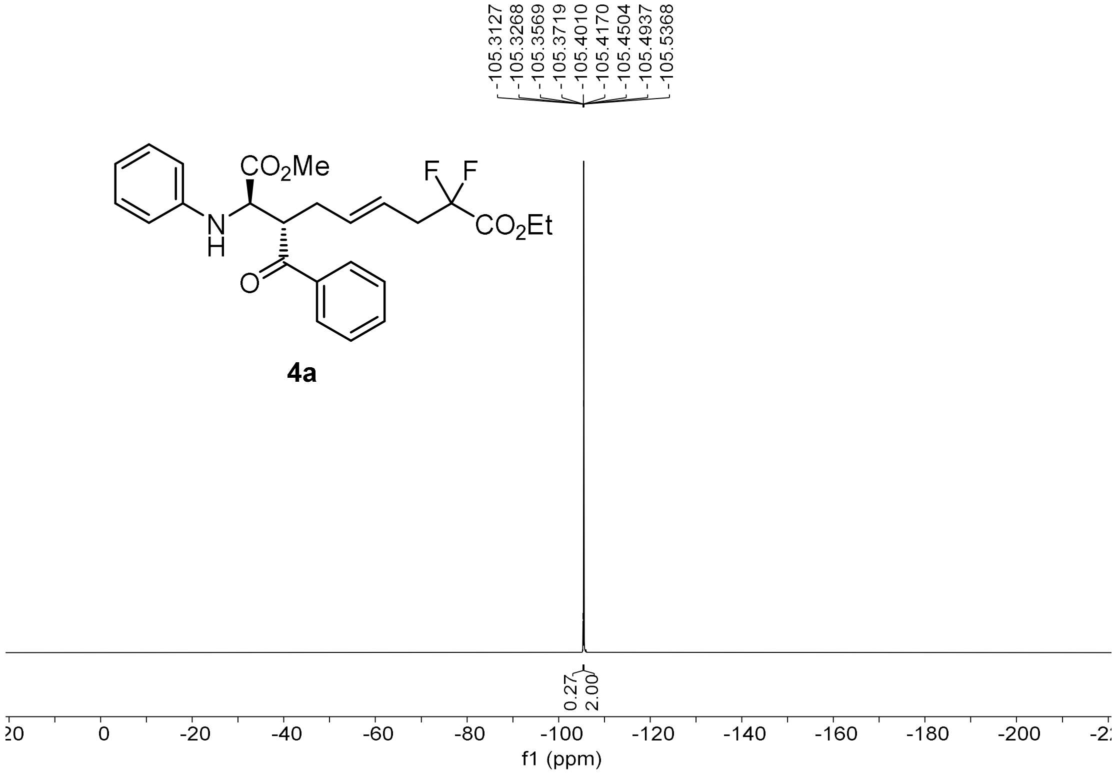

^{19}F NMR (376 MHz, $CDCl_3$) of **4a**

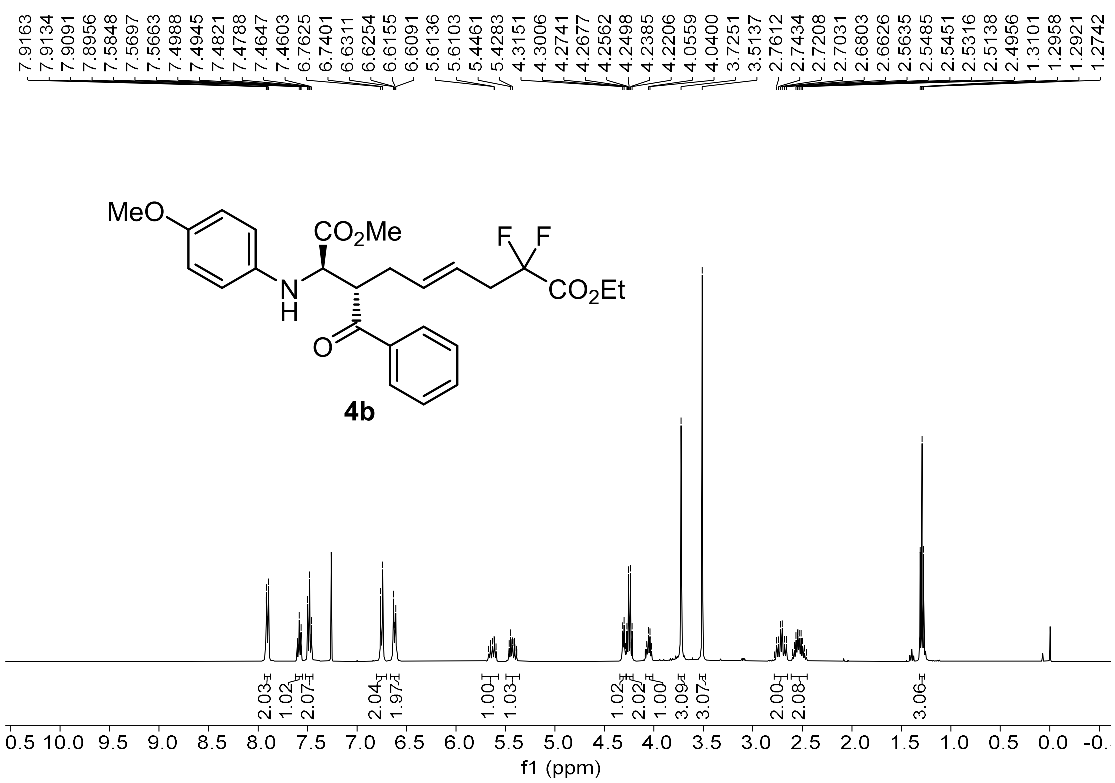

^{1}H NMR (400 MHz, $CDCl_3$) of **4b**

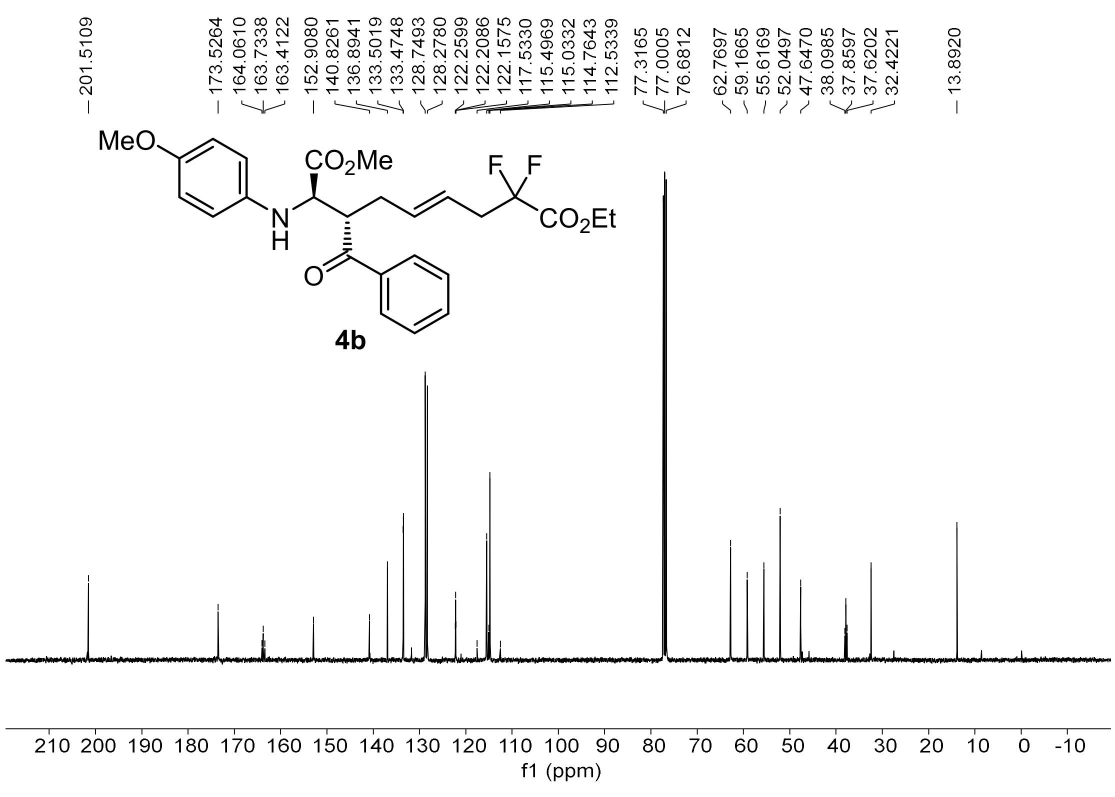

^{13}C NMR (100 MHz, $CDCl_3$) of **4a**

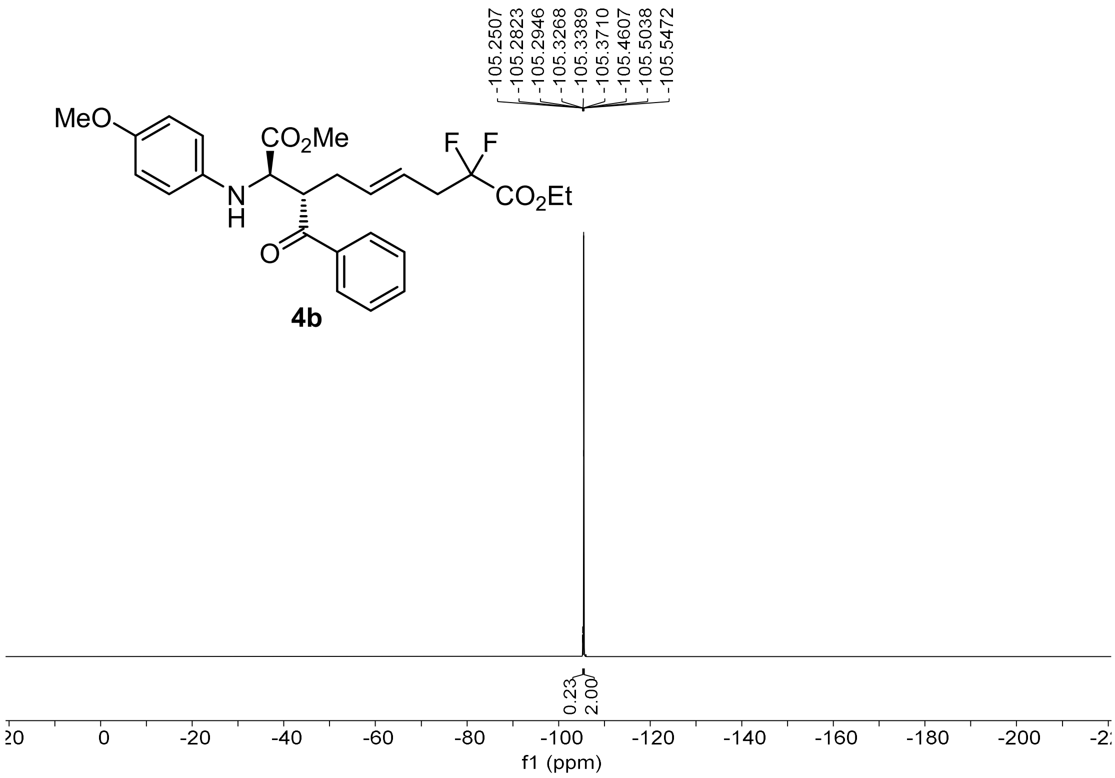

^{19}**F NMR** (376 MHz, $CDCl_3$) of **4b**

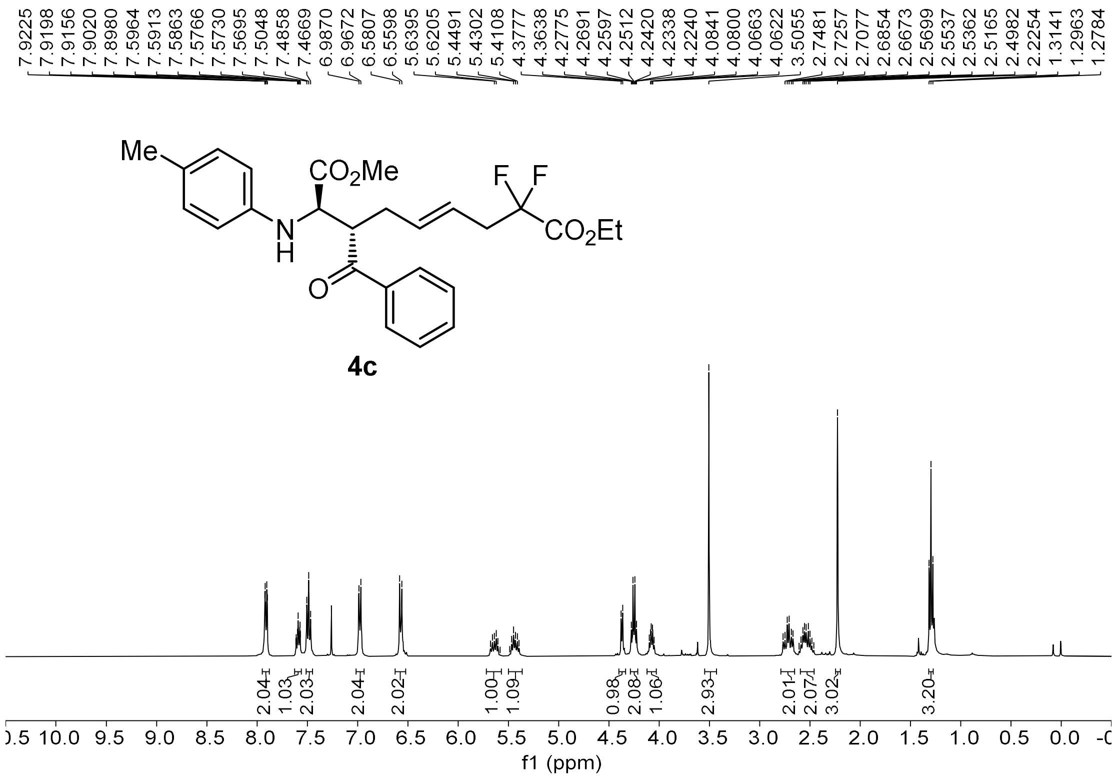

^{1}**H NMR** (400 MHz, $CDCl_3$) of **4c**

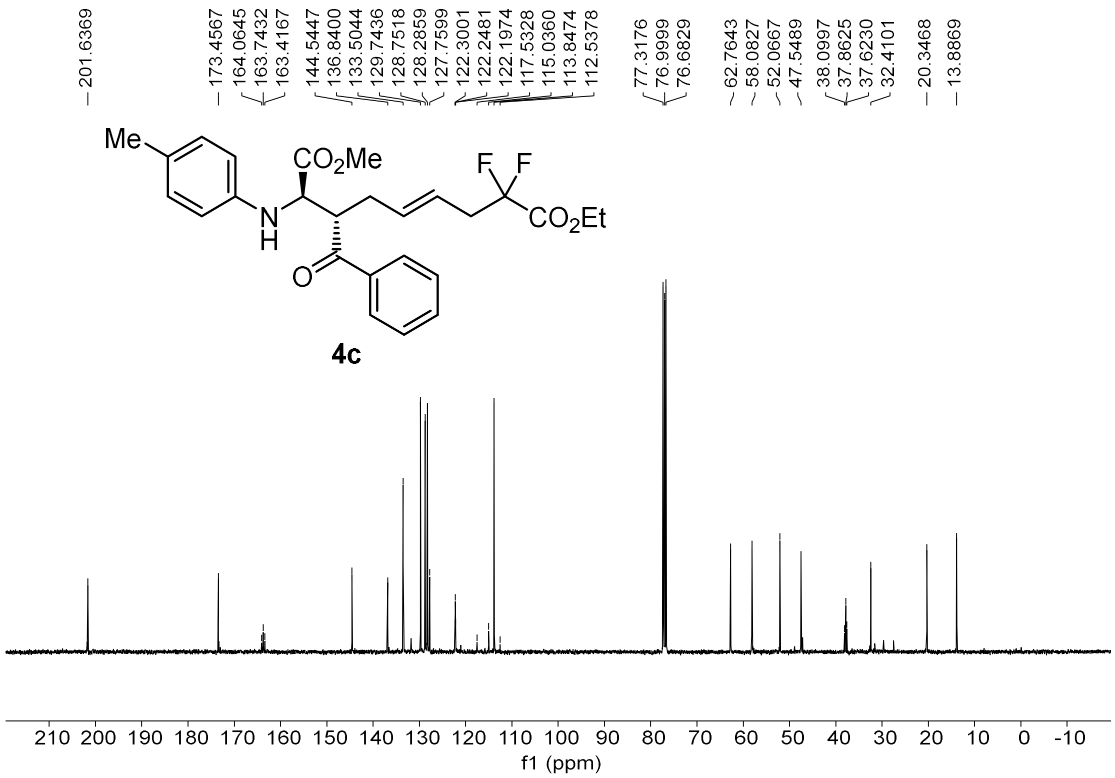

^{13}**C NMR** (100 MHz, $CDCl_3$) of **4c**

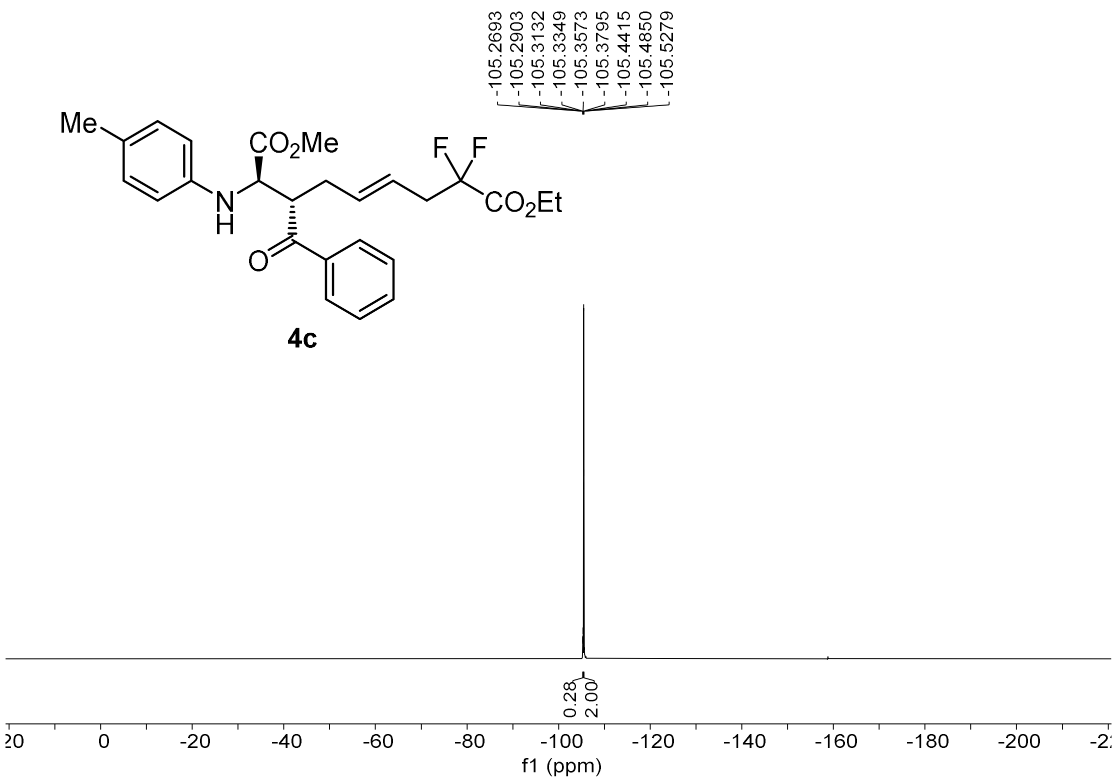

^{19}**F NMR** (376 MHz, $CDCl_3$) of **4c**

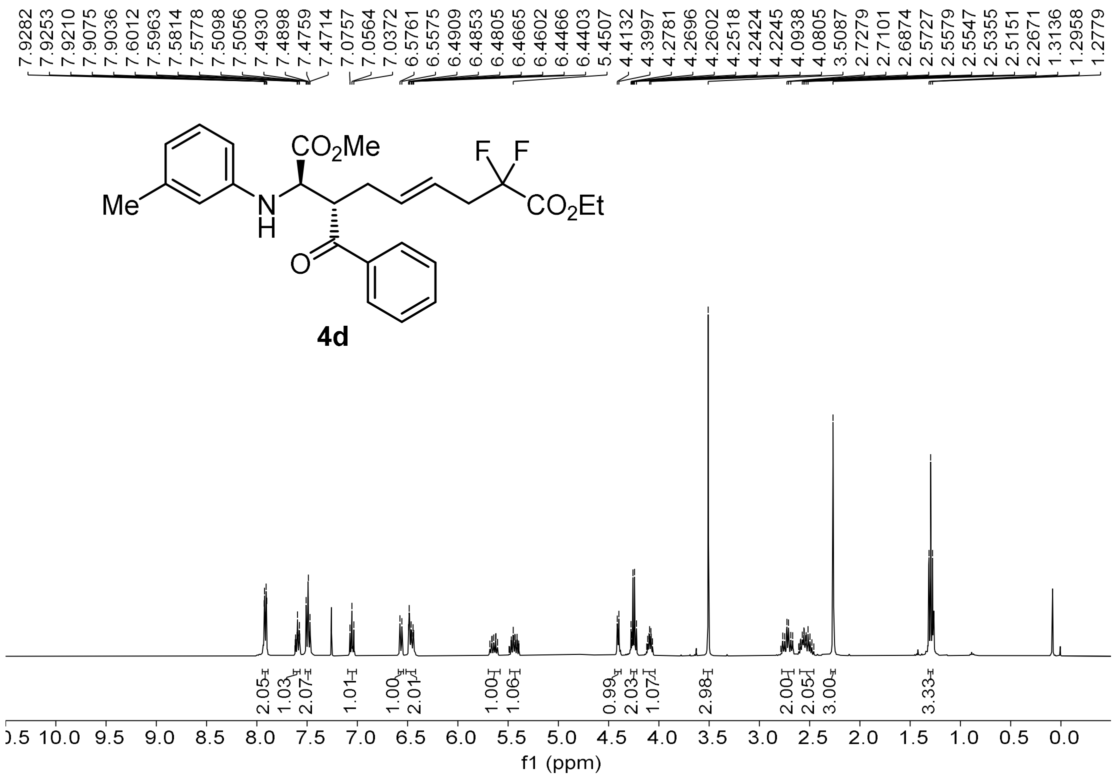

^{1}**H NMR** (400 MHz, CDCl$_3$) of **4d**

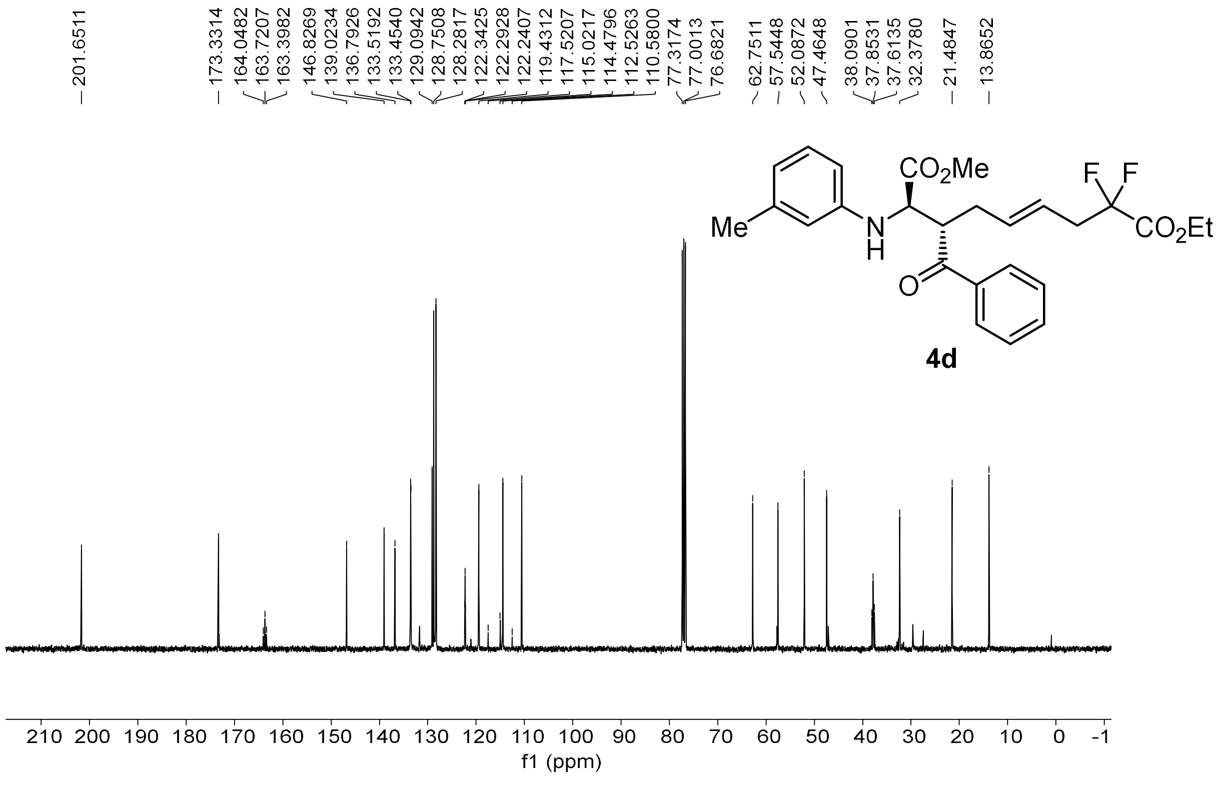

^{13}**C NMR** (100 MHz, CDCl$_3$) of **4d**

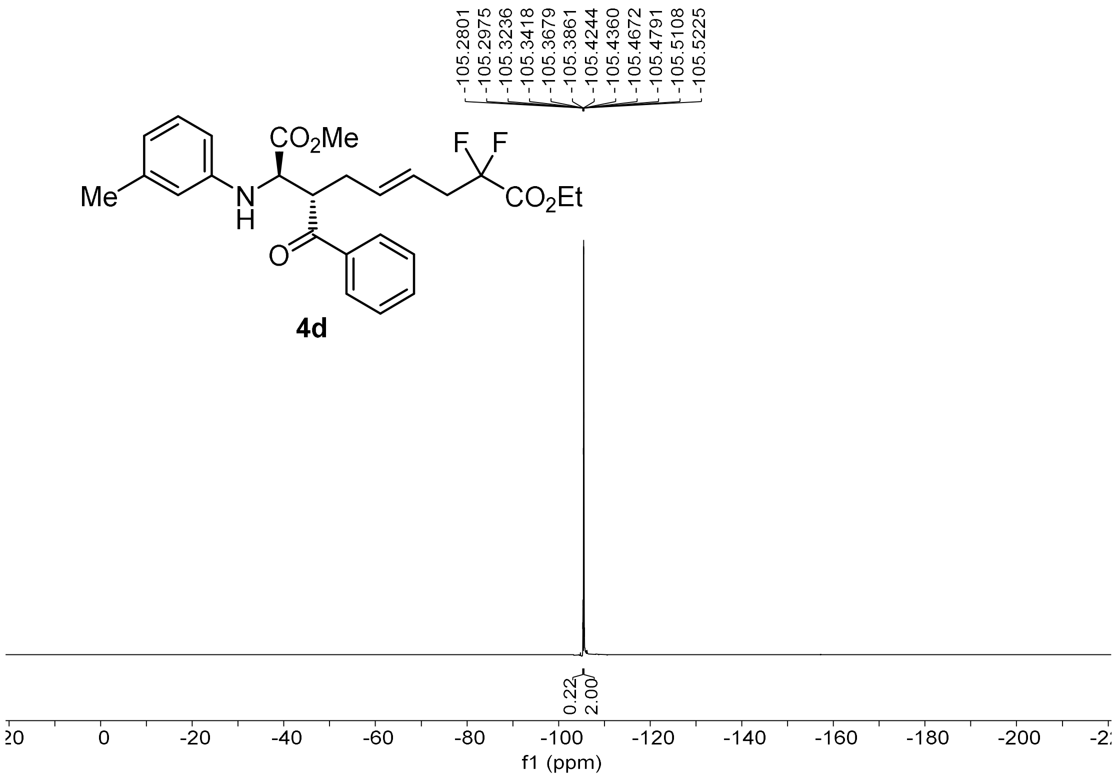

^{19}**F NMR** (376 MHz, CDCl$_3$) of **4d**

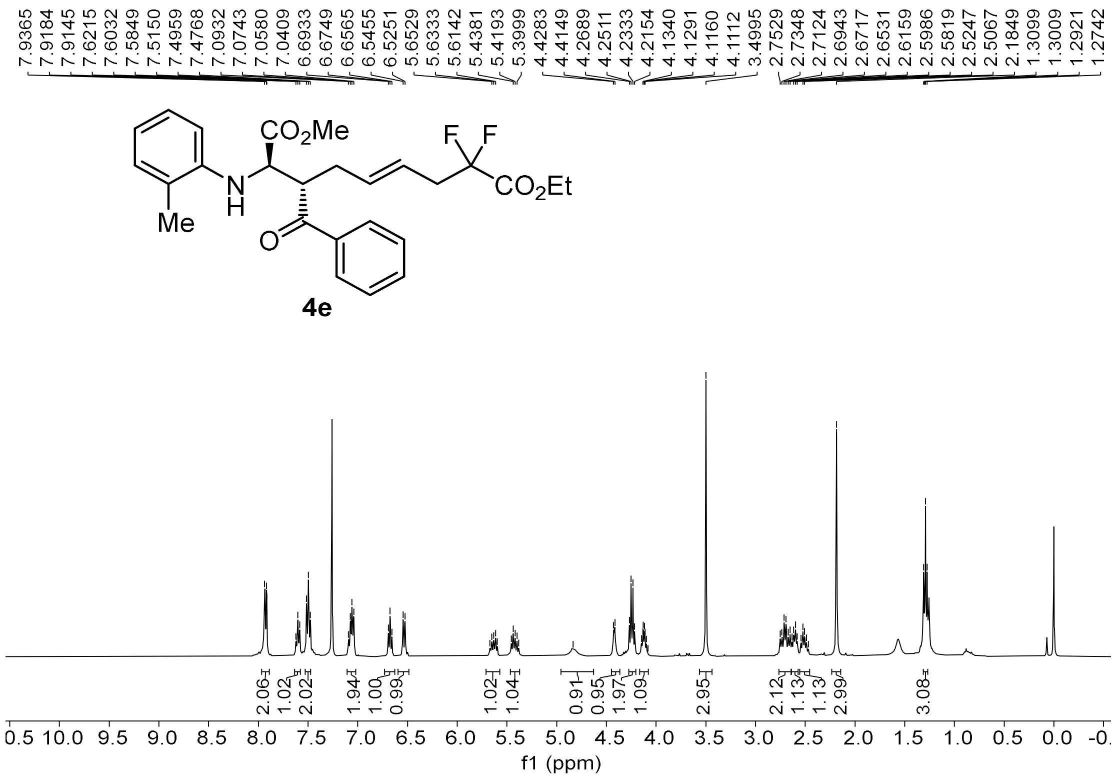

^{1}**H NMR** (400 MHz, CDCl$_3$) of **4e**

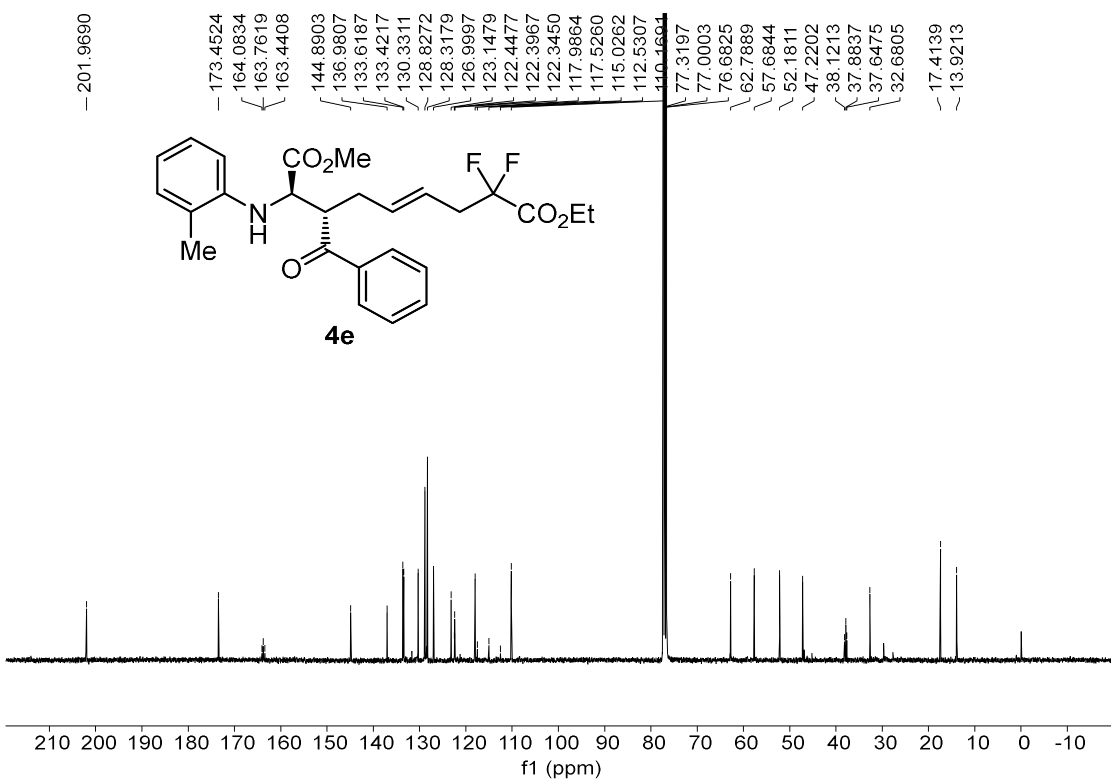

^{13}C NMR (100 MHz, $CDCl_3$) of **4e**

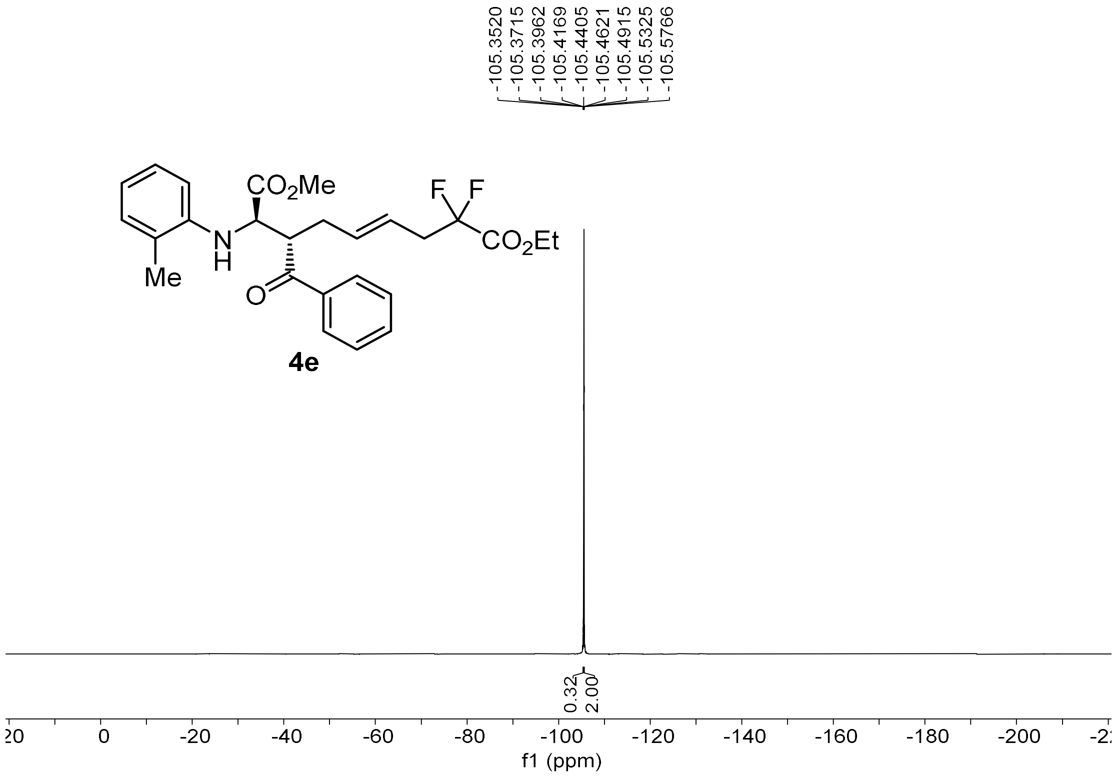

^{19}F NMR (376 MHz, $CDCl_3$) of **4e**

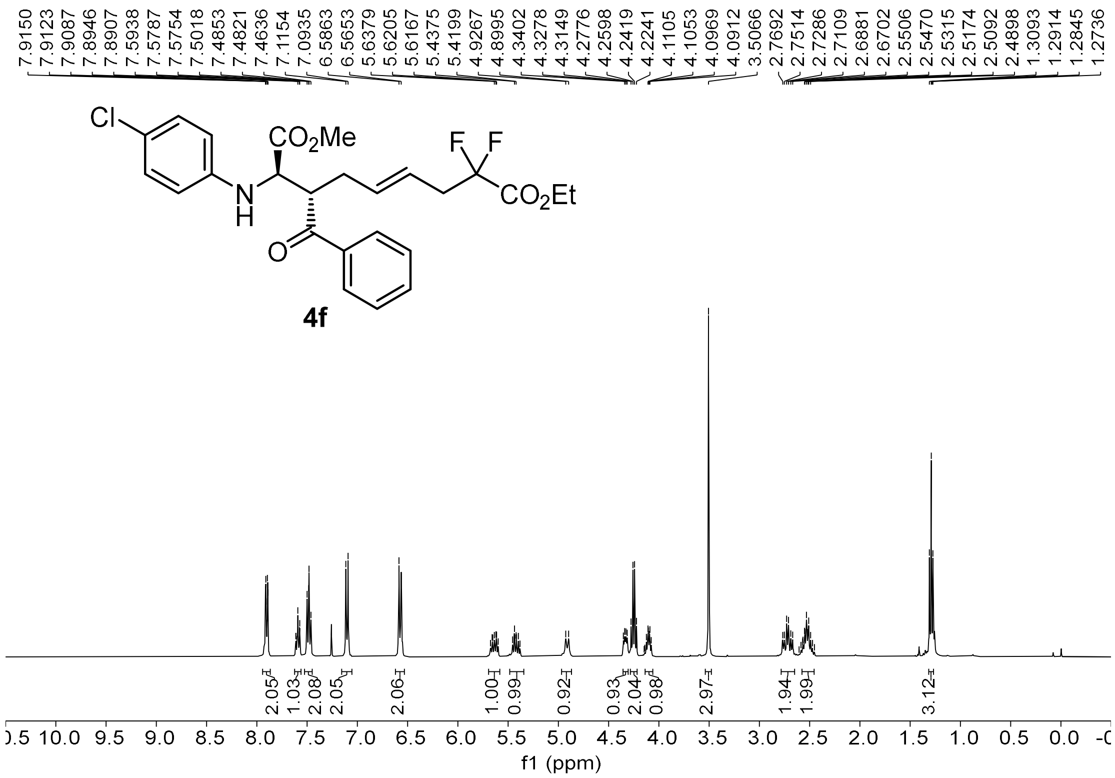

¹H NMR (400 MHz, CDCl$_3$) of **4f**

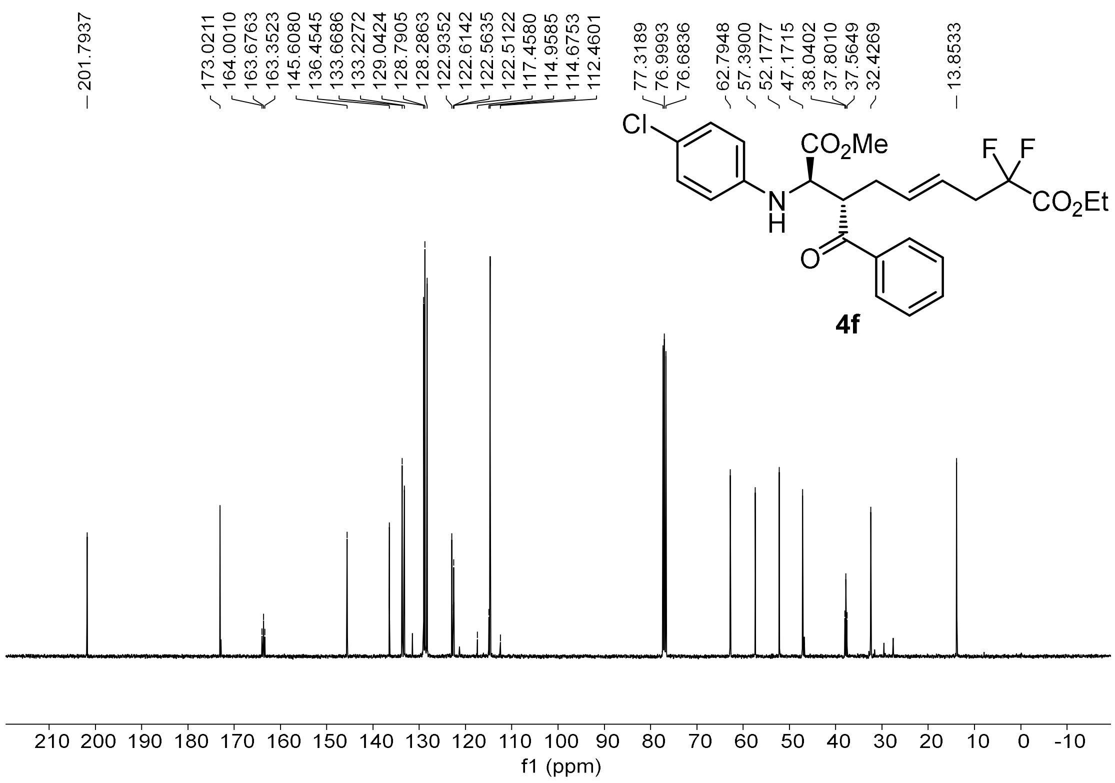

¹³C NMR (100 MHz, CDCl$_3$) of **4f**

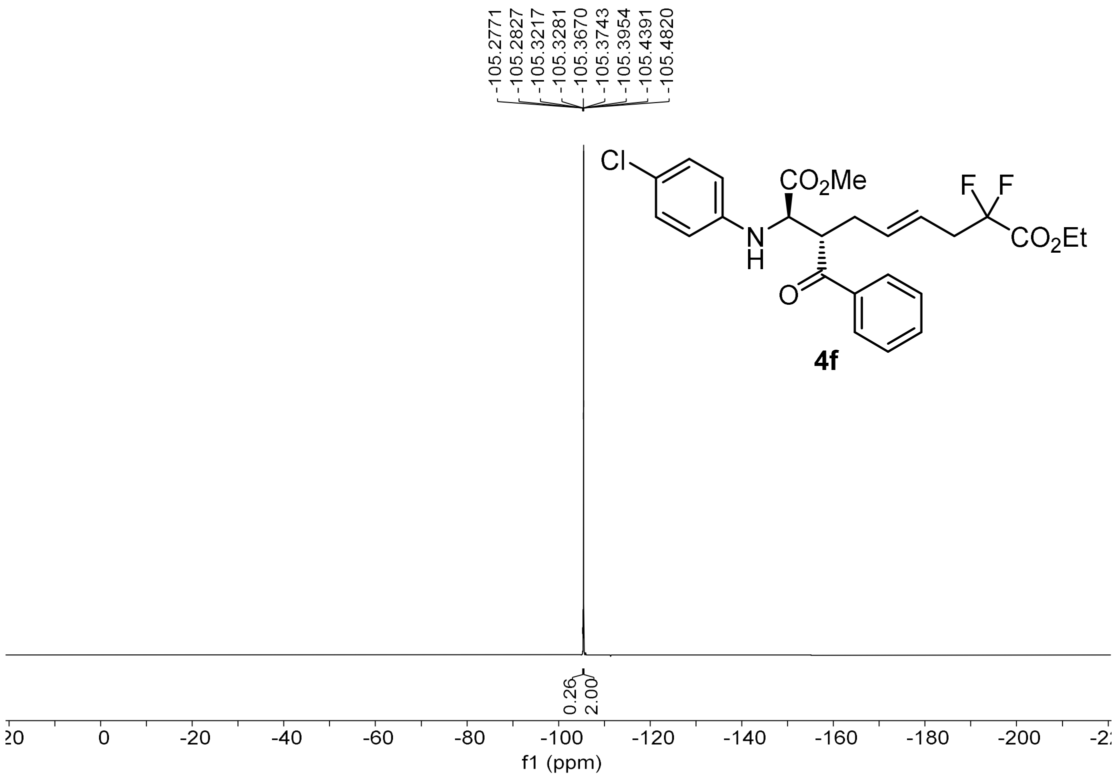

^{19}F NMR (376 MHz, $CDCl_3$) of **4f**

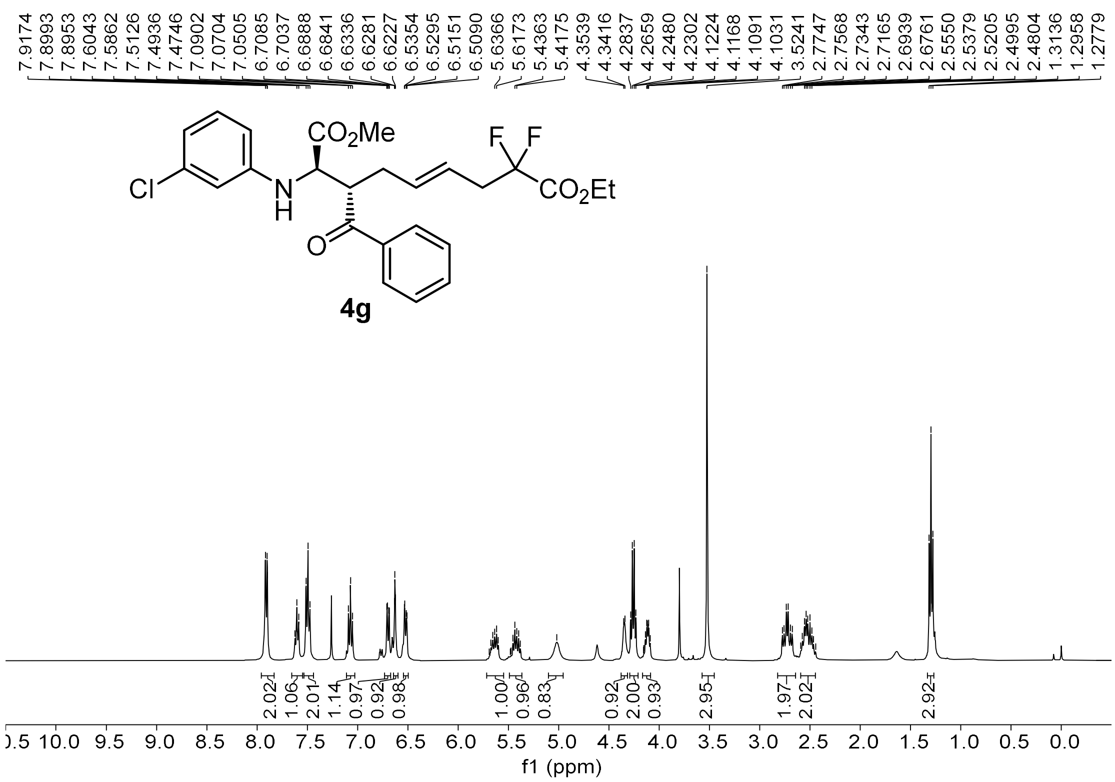

^{1}H NMR (400 MHz, $CDCl_3$) of **4g**

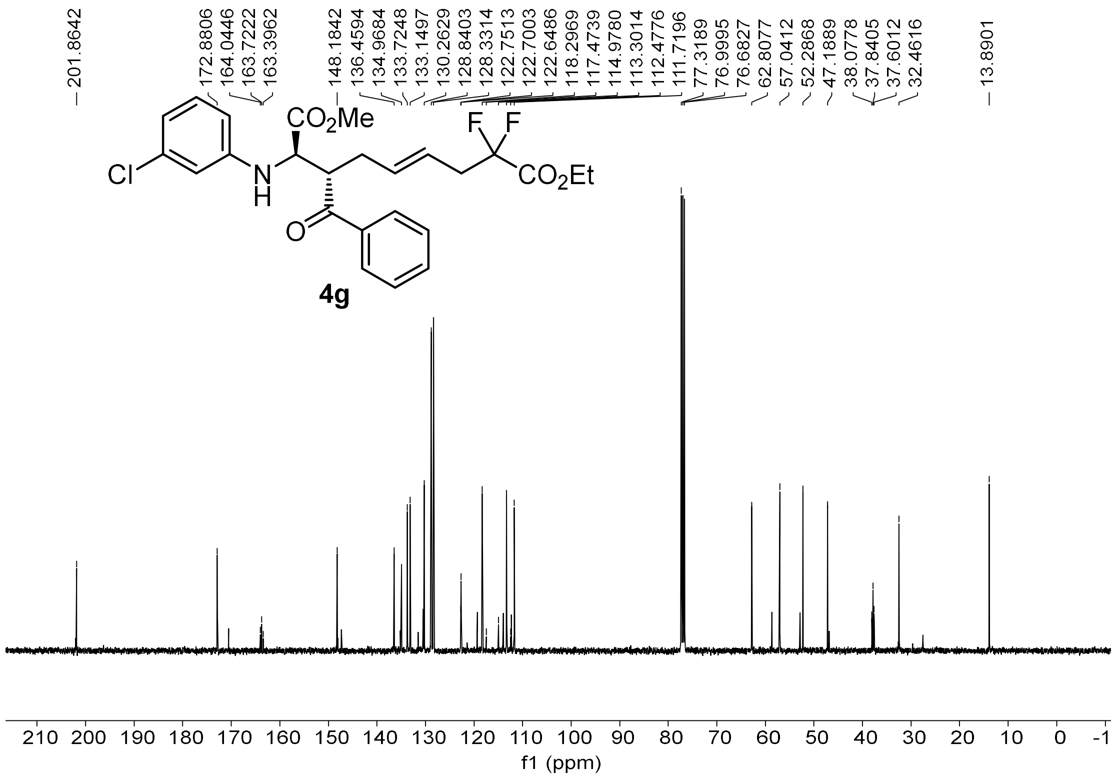

^{13}C NMR (100 MHz, $CDCl_3$) of **4g**

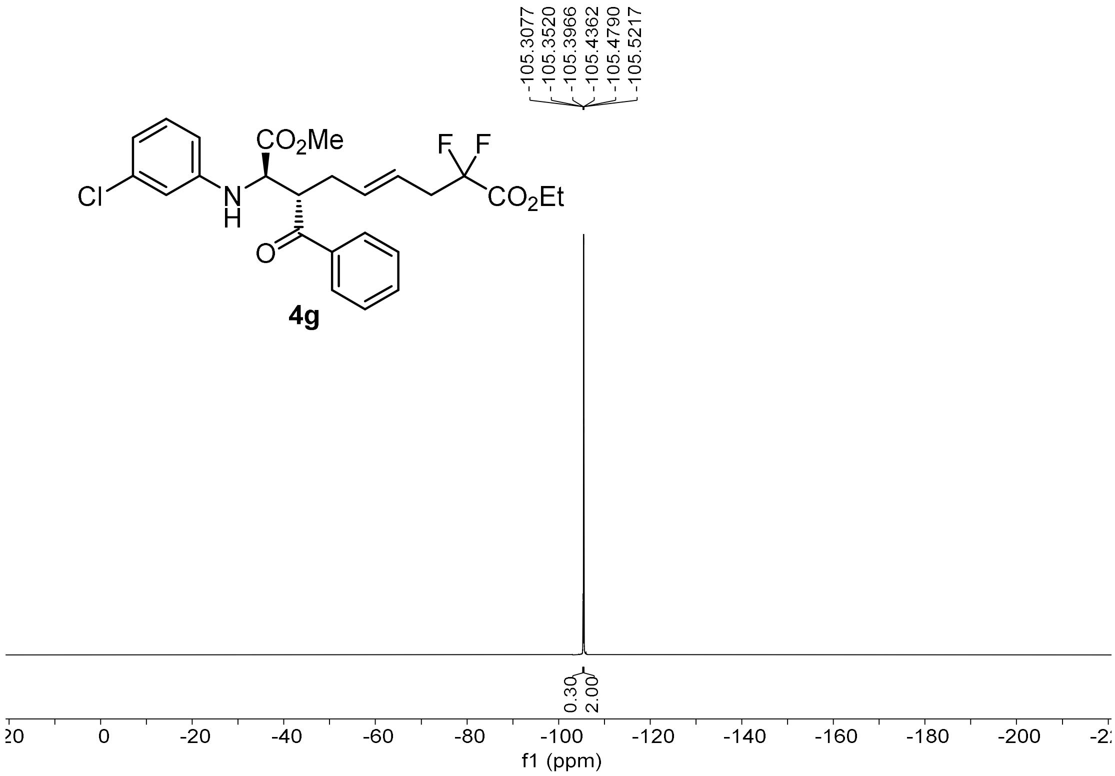

^{19}F NMR (376 MHz, $CDCl_3$) of **4g**

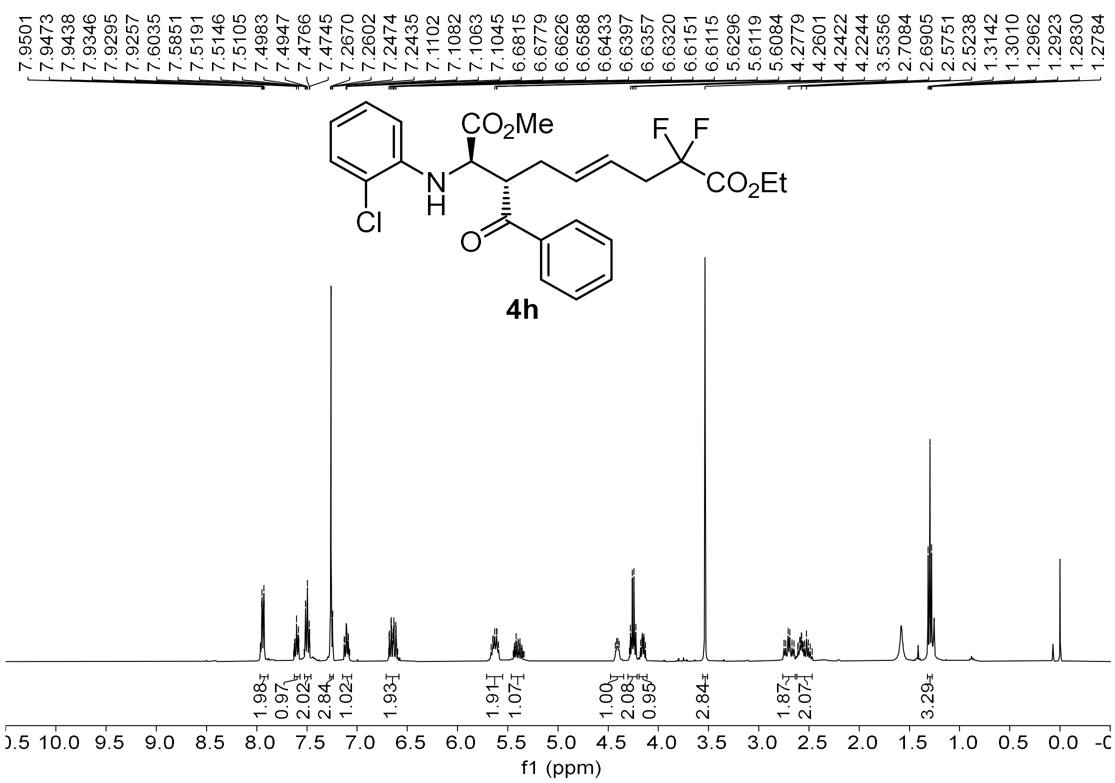

^{1}H NMR (400 MHz, $CDCl_3$) of **4h**

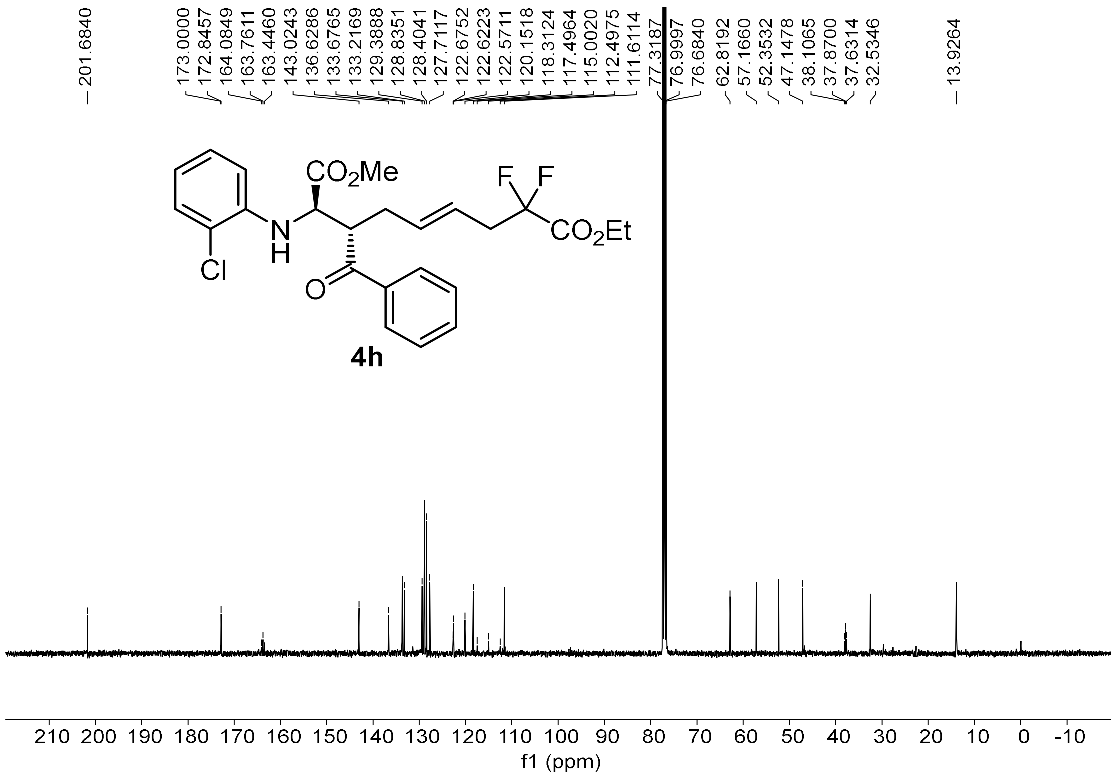

^{13}C NMR (100 MHz, $CDCl_3$) of **4h**

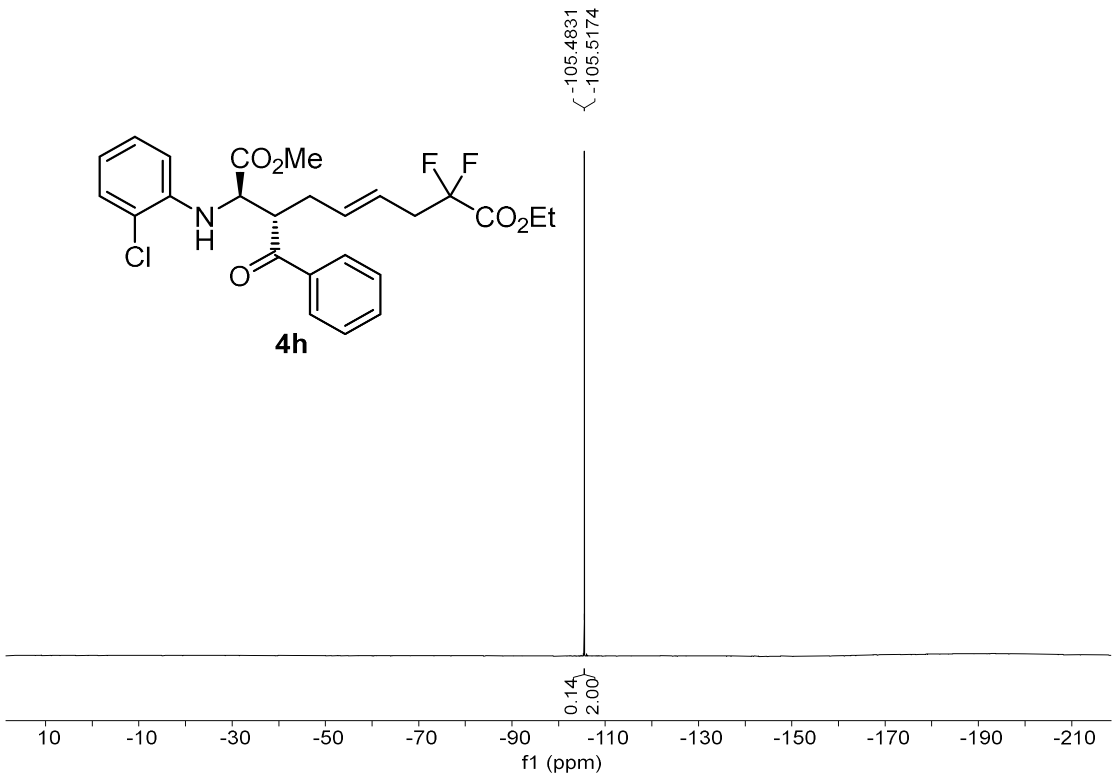

^{19}F NMR (377 MHz, $CDCl_3$) of **4h**

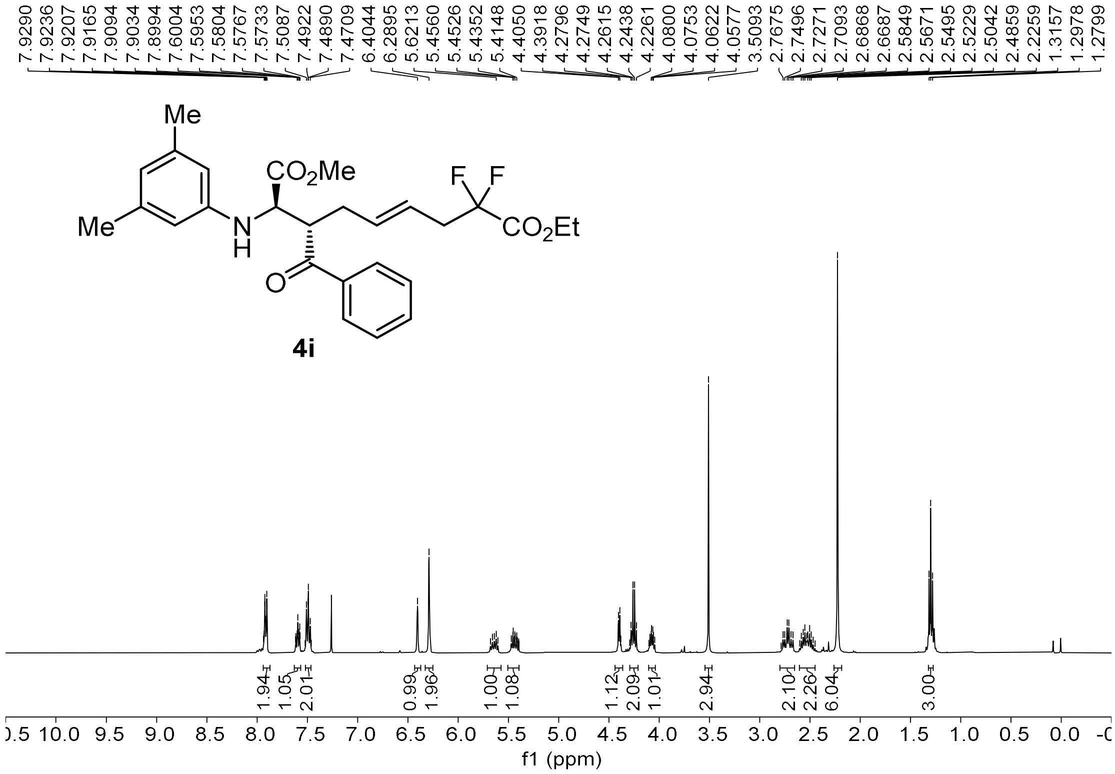

^{1}H NMR (400 MHz, $CDCl_3$) of **4i**

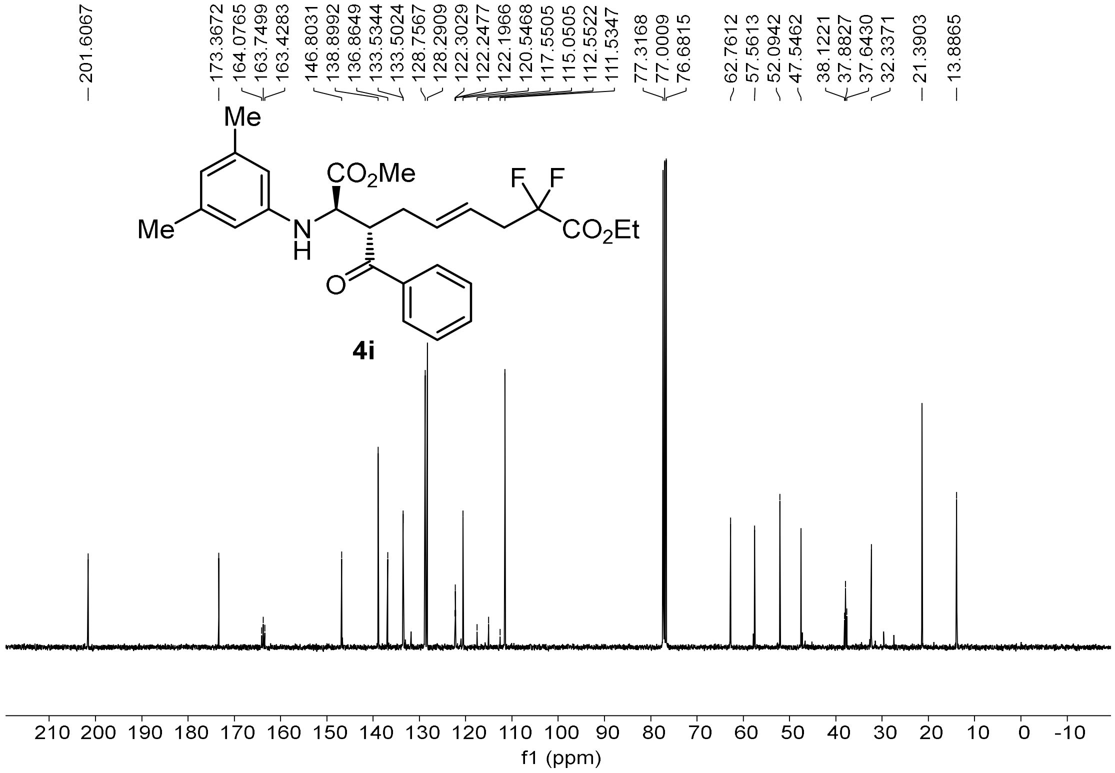

^{13}C NMR (100 MHz, $CDCl_3$) of **4i**

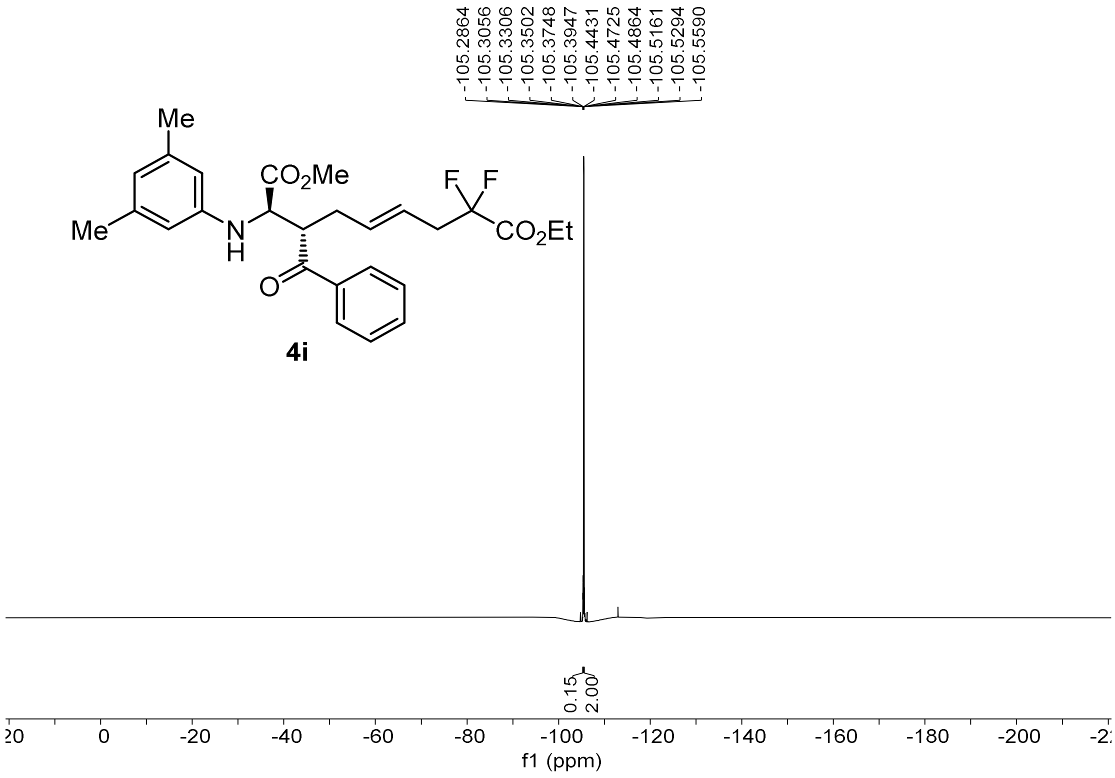

^{19}F NMR (376 MHz, $CDCl_3$) of **4i**

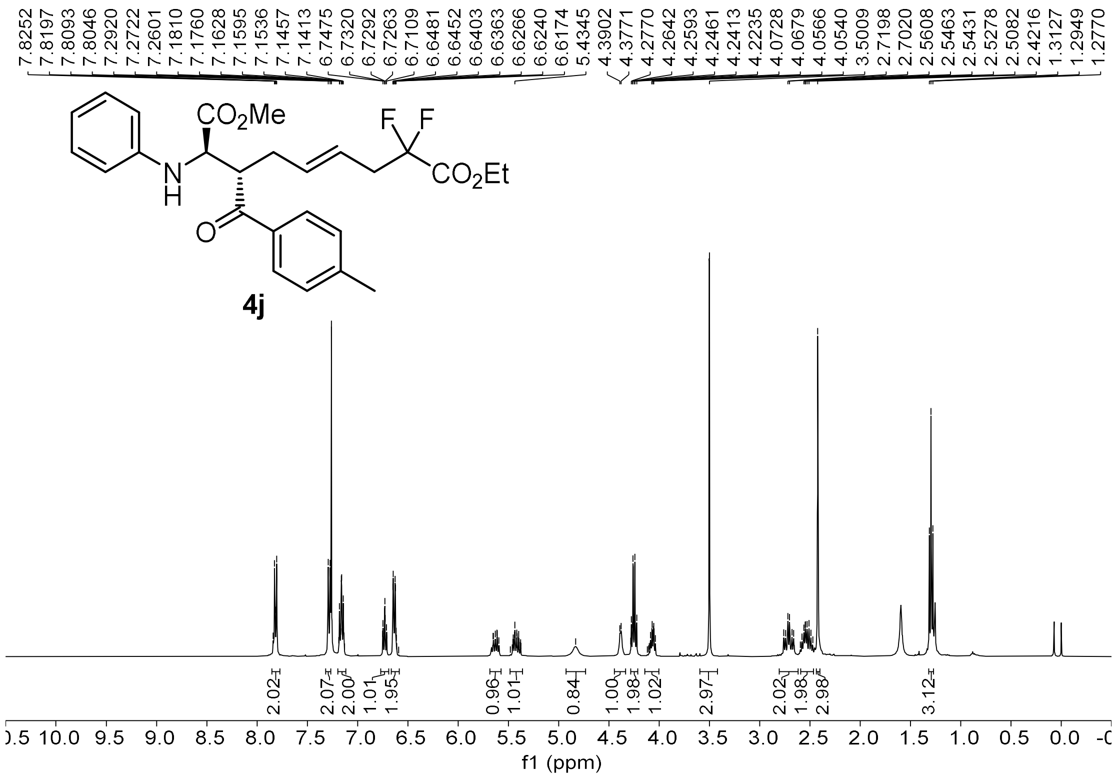

^{1}H NMR (400 MHz, $CDCl_3$) of **4j**

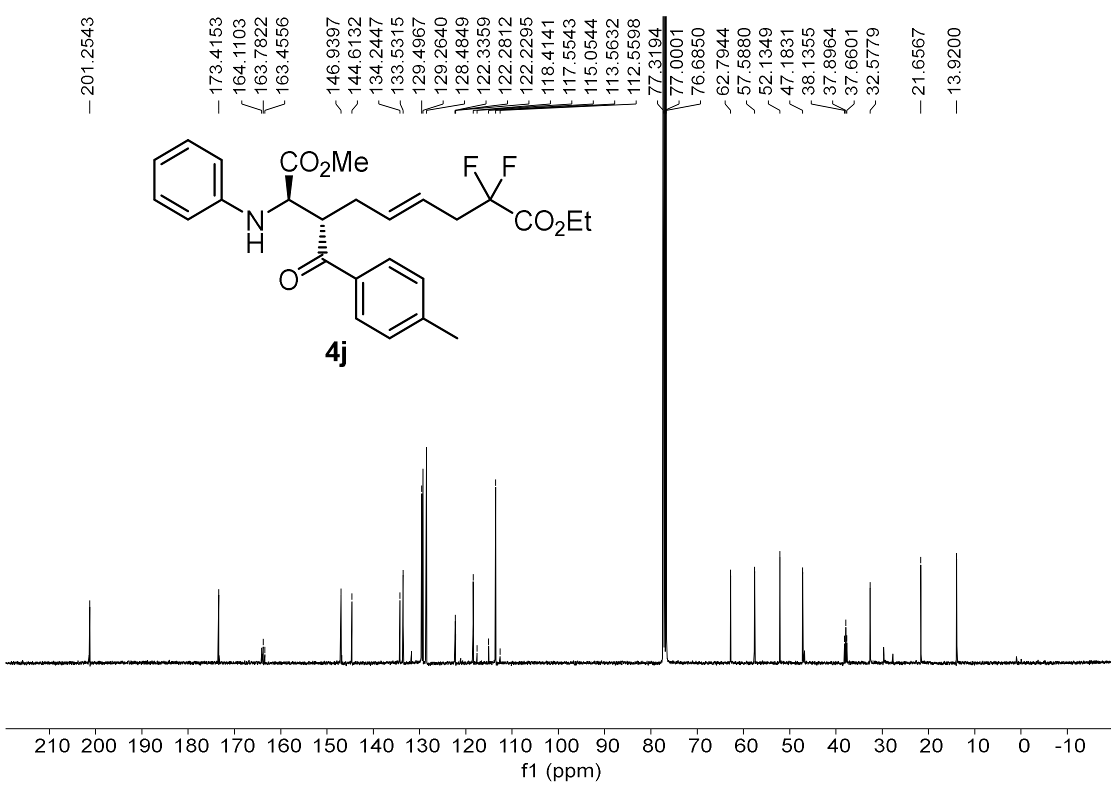

^{13}C NMR (100 MHz, $CDCl_3$) of **4j**

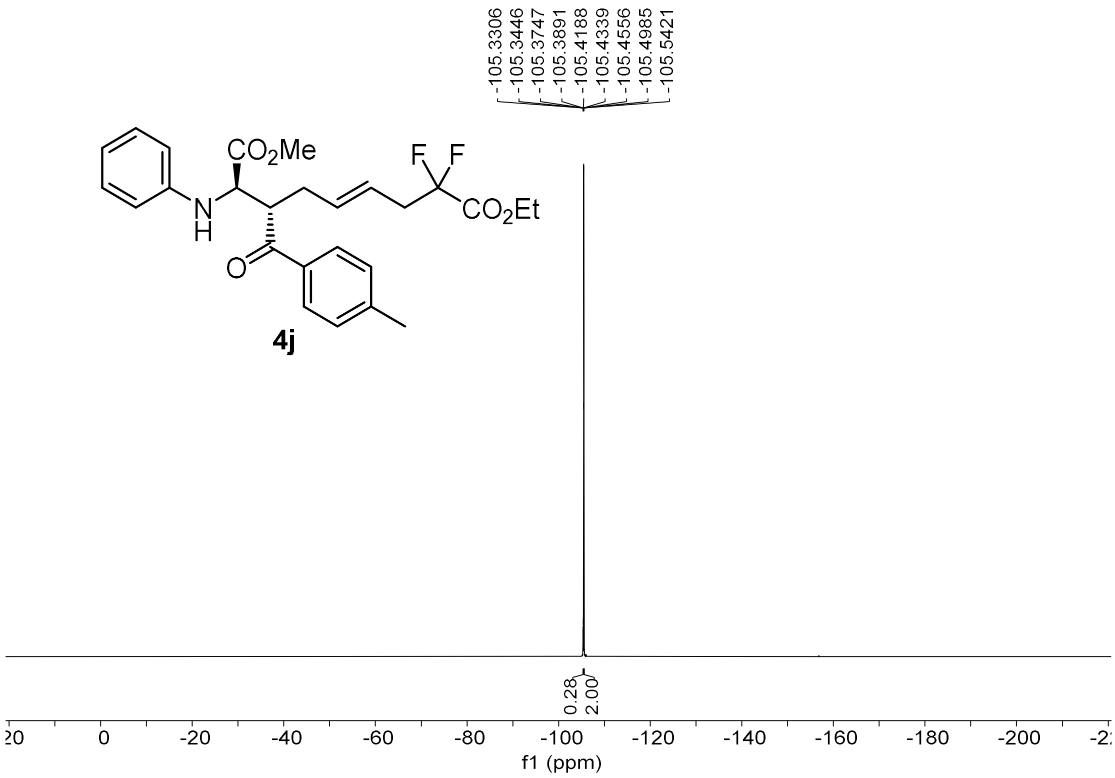

^{19}F NMR (376 MHz, $CDCl_3$) of **4j**

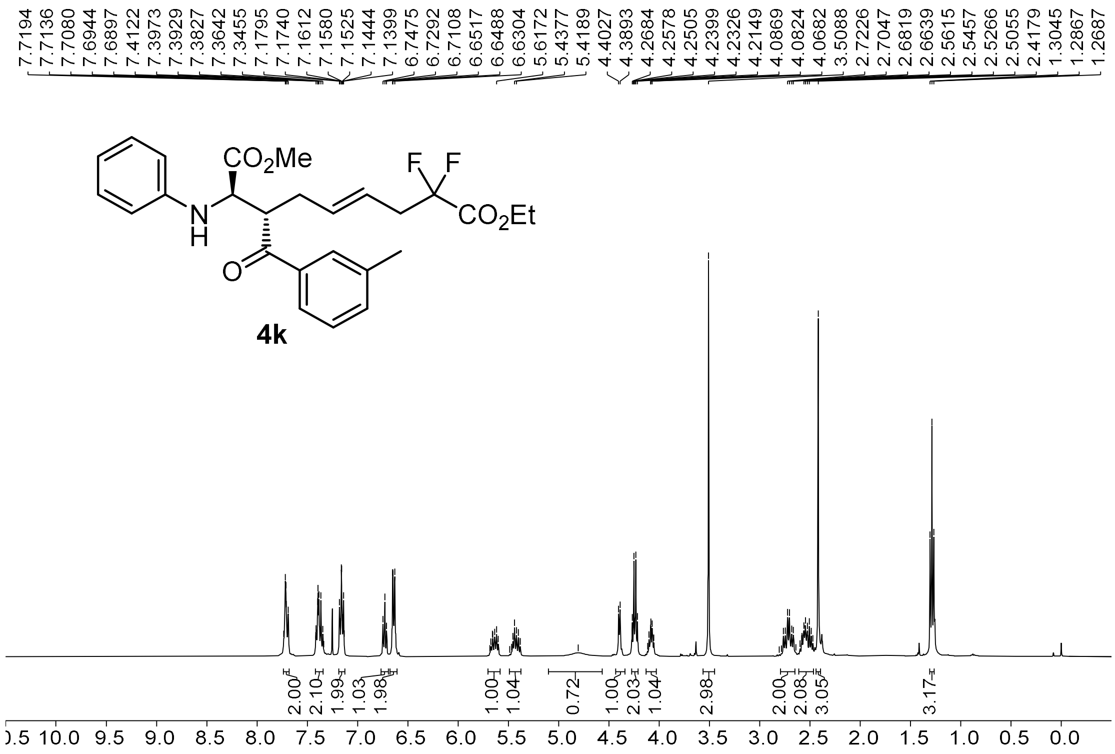

^{1}H NMR (400 MHz, $CDCl_3$) of **4k**

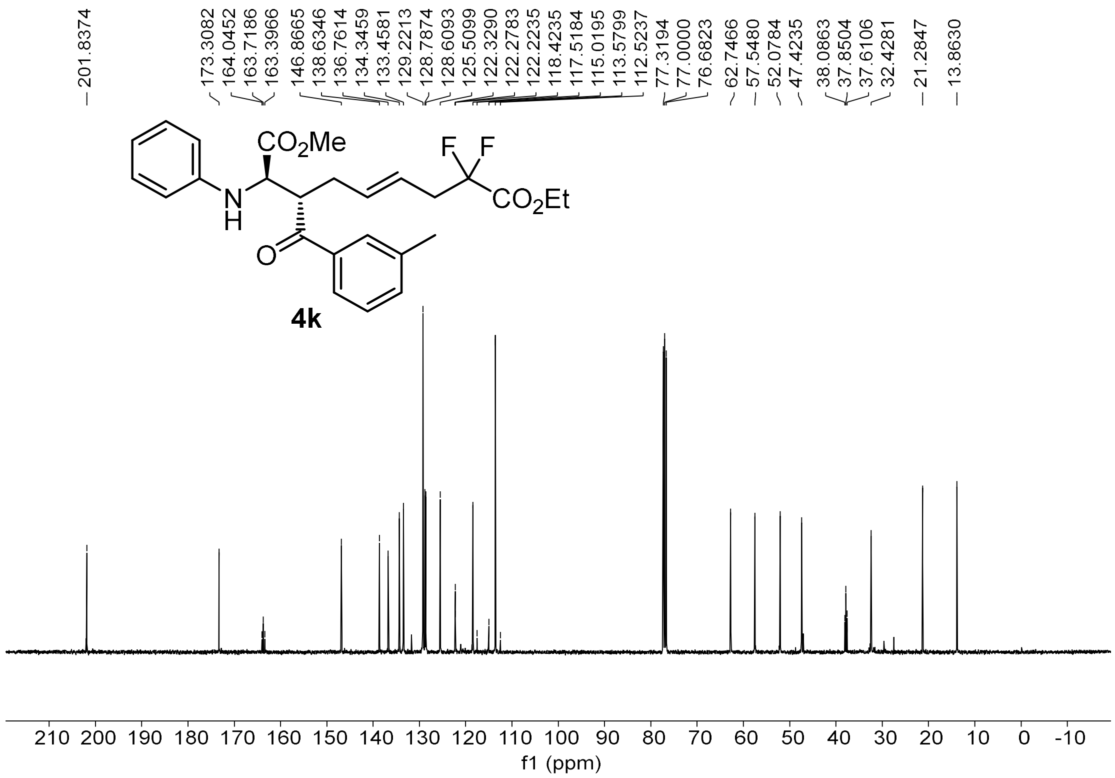

^{13}C NMR (100 MHz, $CDCl_3$) of **4k**

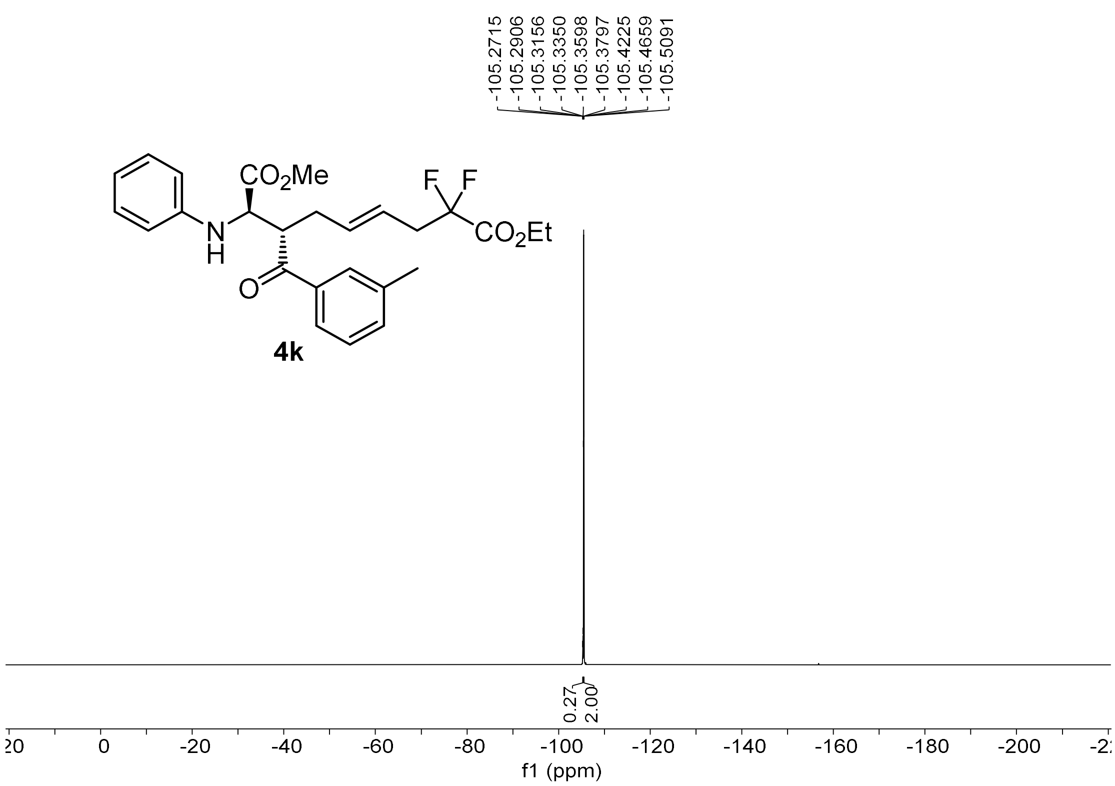

^{19}F NMR (376 MHz, $CDCl_3$) of **4k**

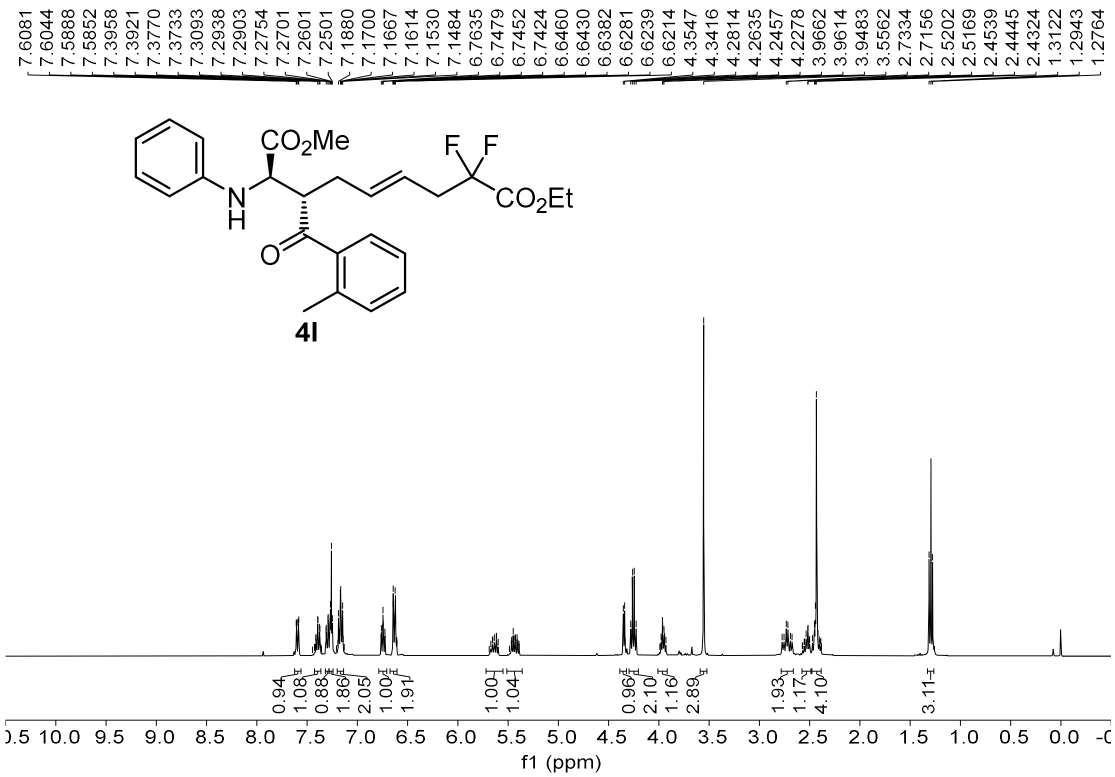

^{1}**H NMR** (400 MHz, $CDCl_3$) of **4l**

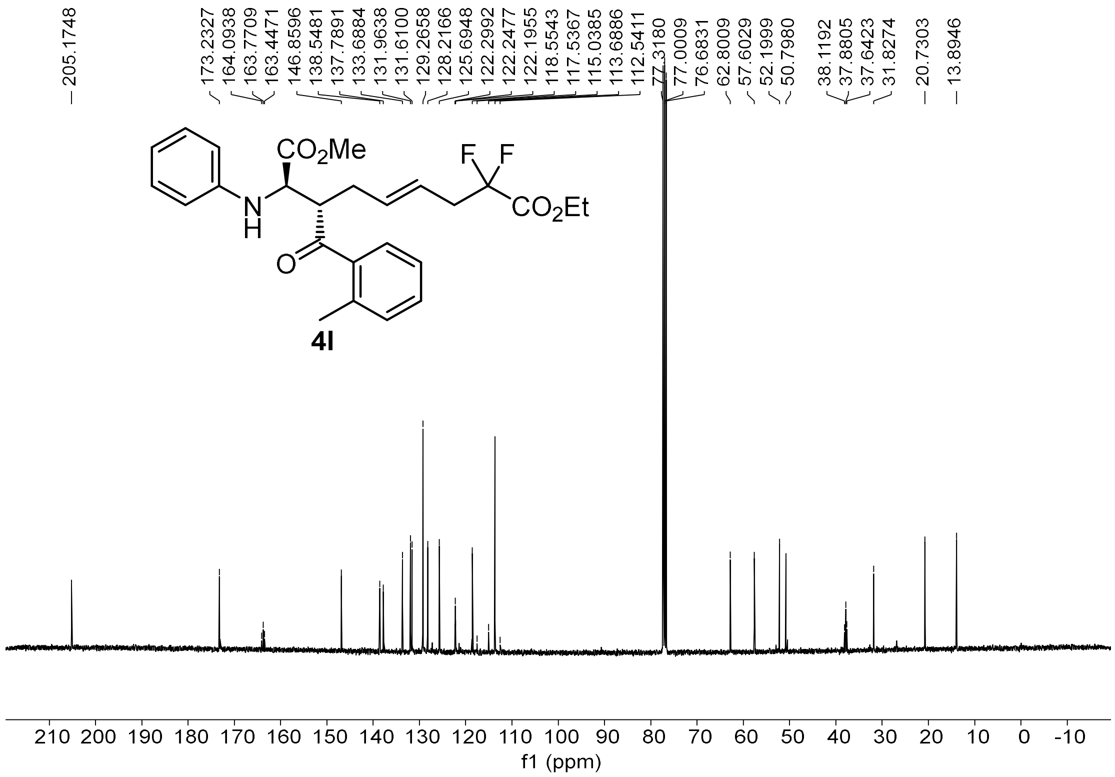

^{13}**C NMR** (100 MHz, $CDCl_3$) of **4l**

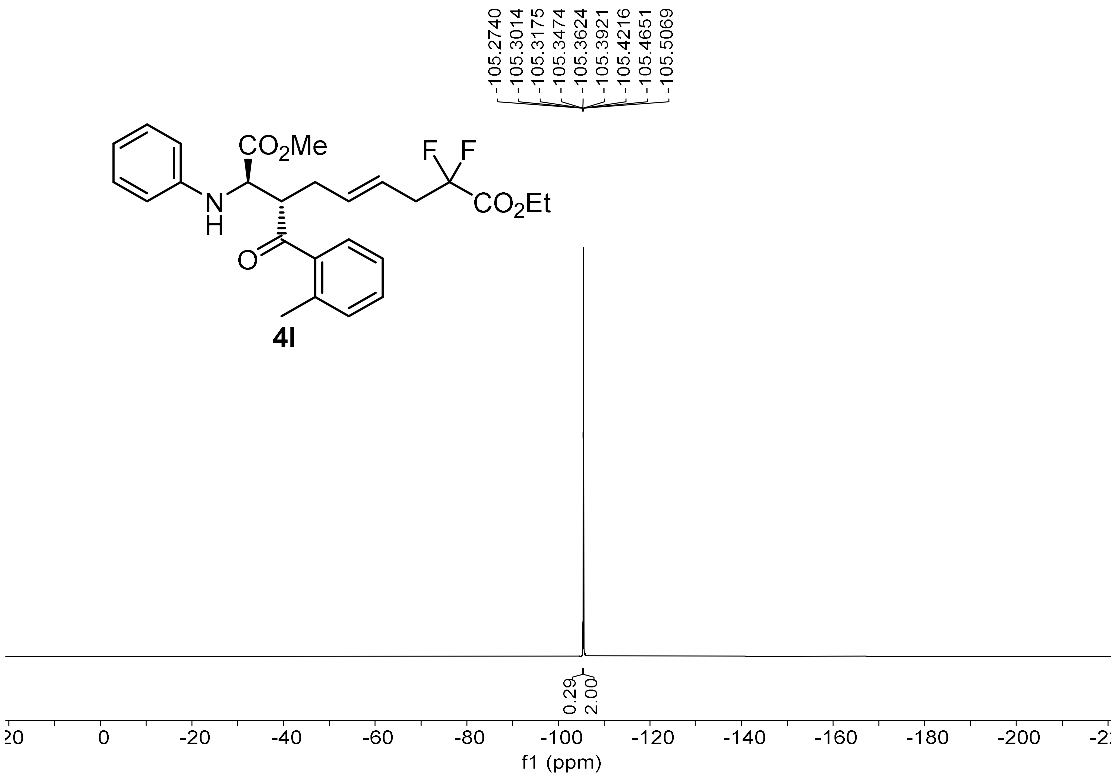

^{19}F NMR (376 MHz, $CDCl_3$) of **4l**

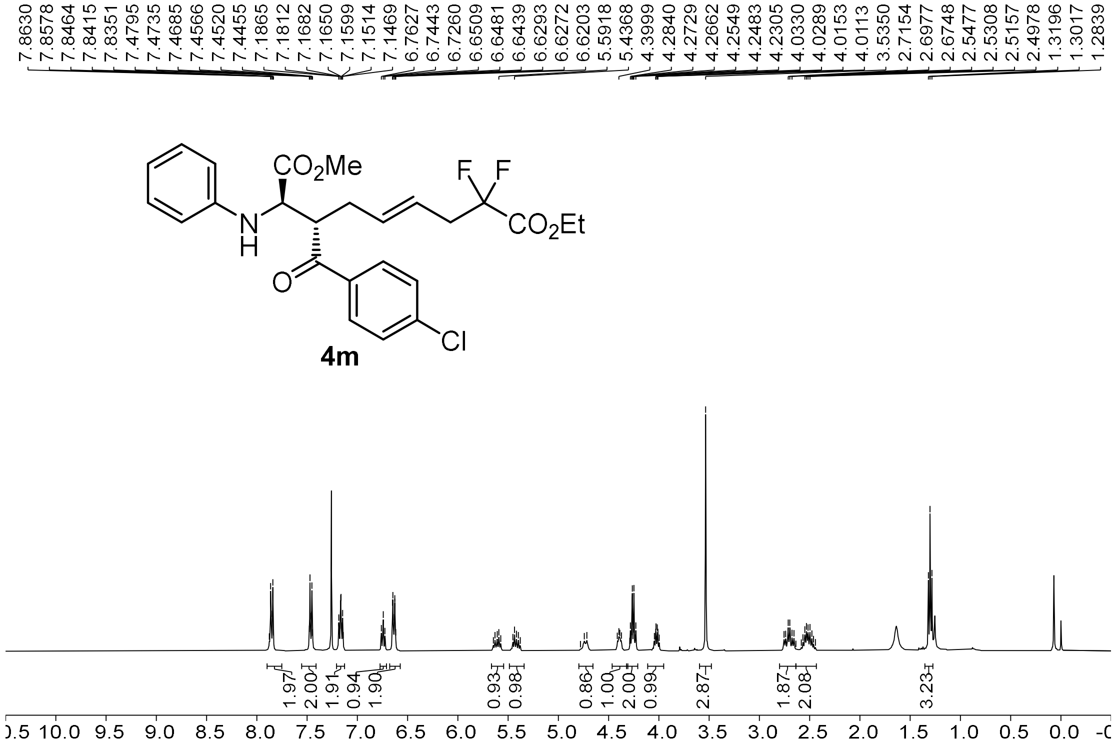

^{1}H NMR (400 MHz, $CDCl_3$) of **4m**

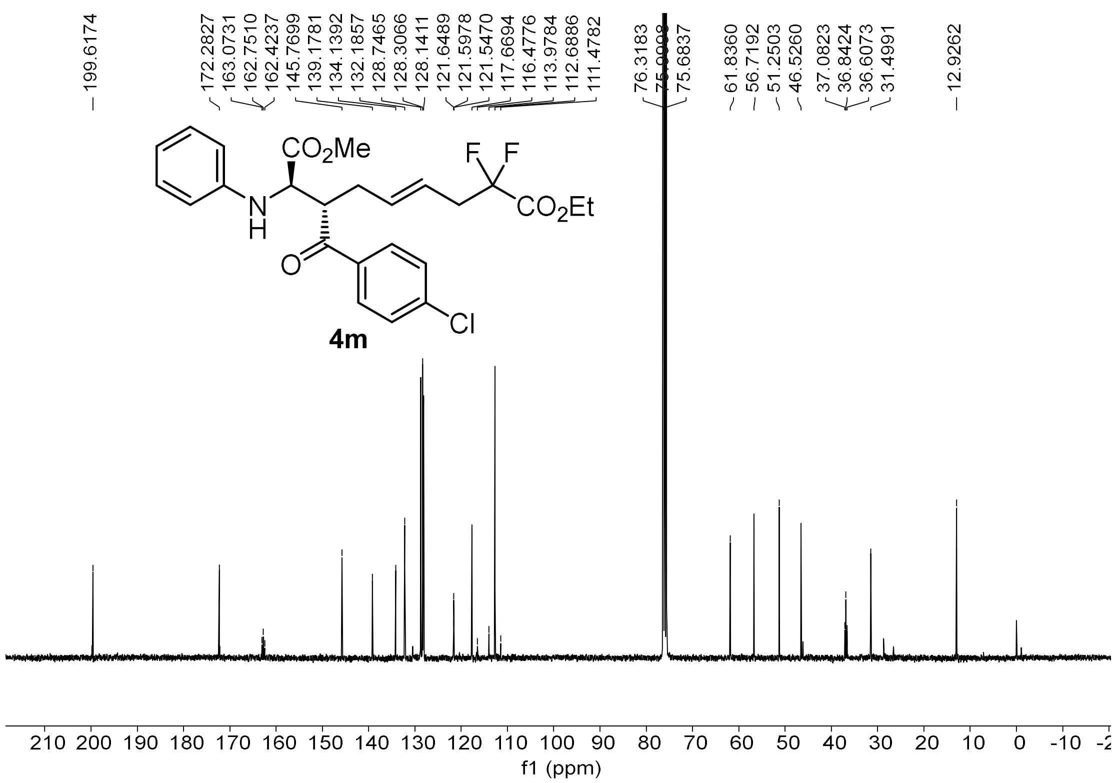

^{13}C NMR (100 MHz, $CDCl_3$) of **4m**

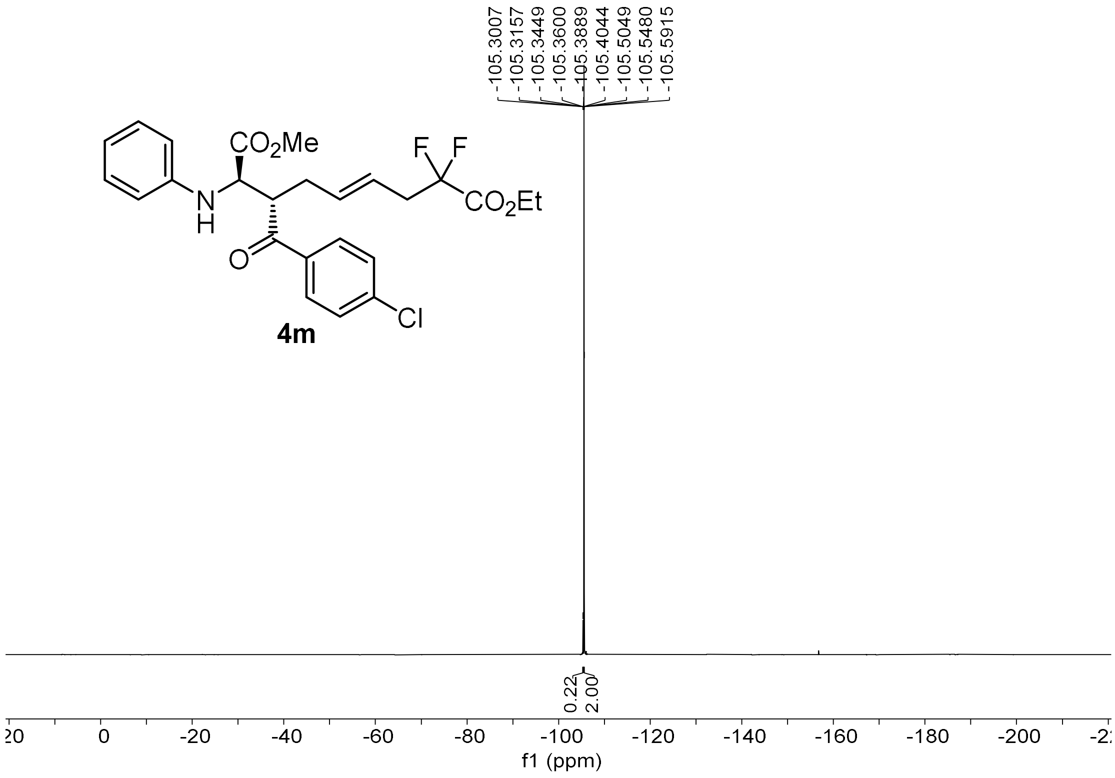

^{19}F NMR (376 MHz, $CDCl_3$) of **4m**

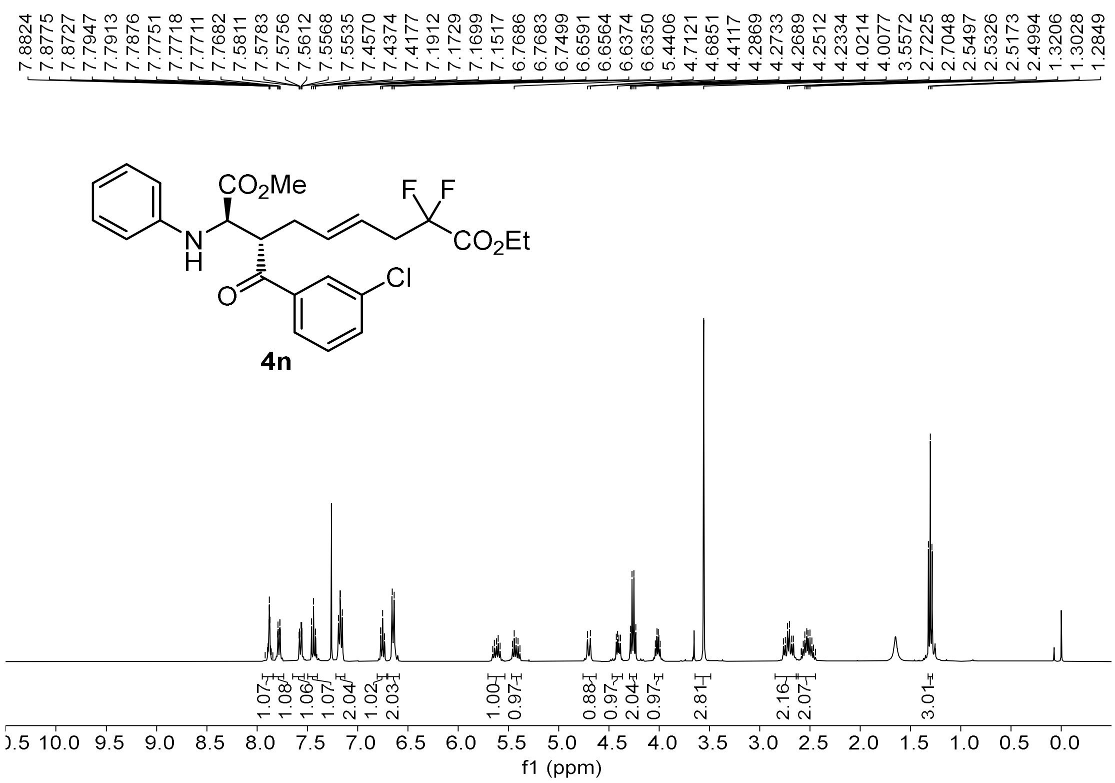

^{1}H NMR (400 MHz, $CDCl_3$) of **4n**

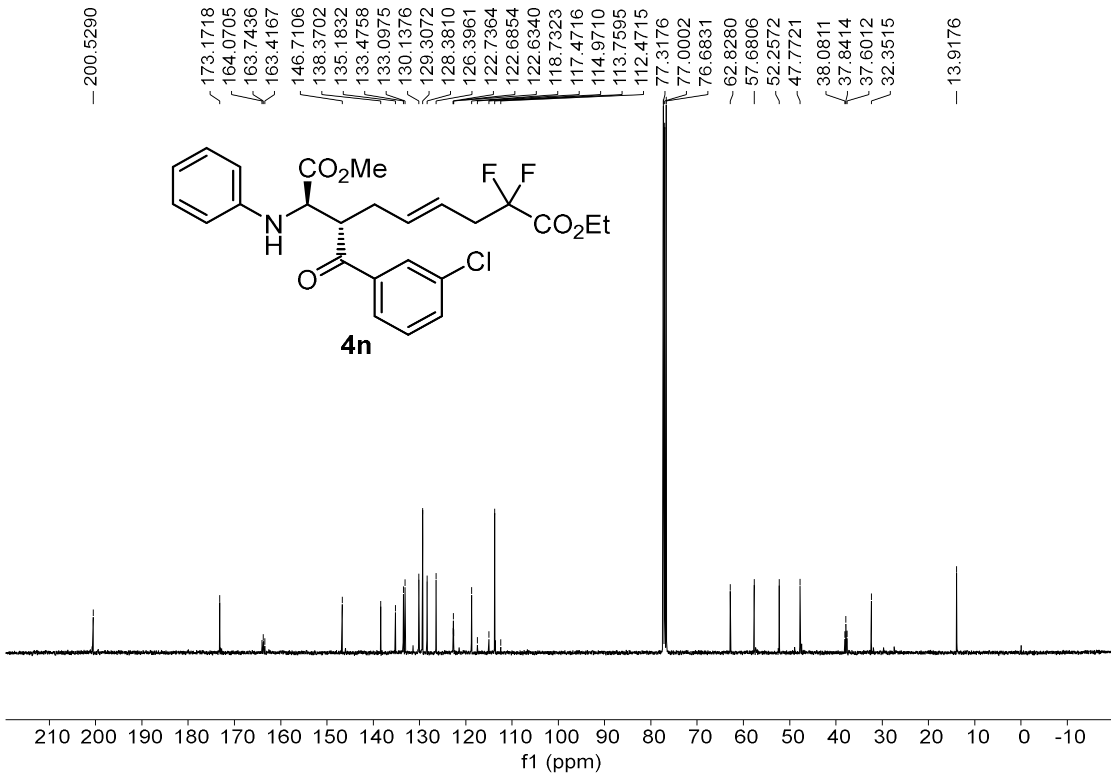

^{13}C NMR (100 MHz, $CDCl_3$) of **4n**

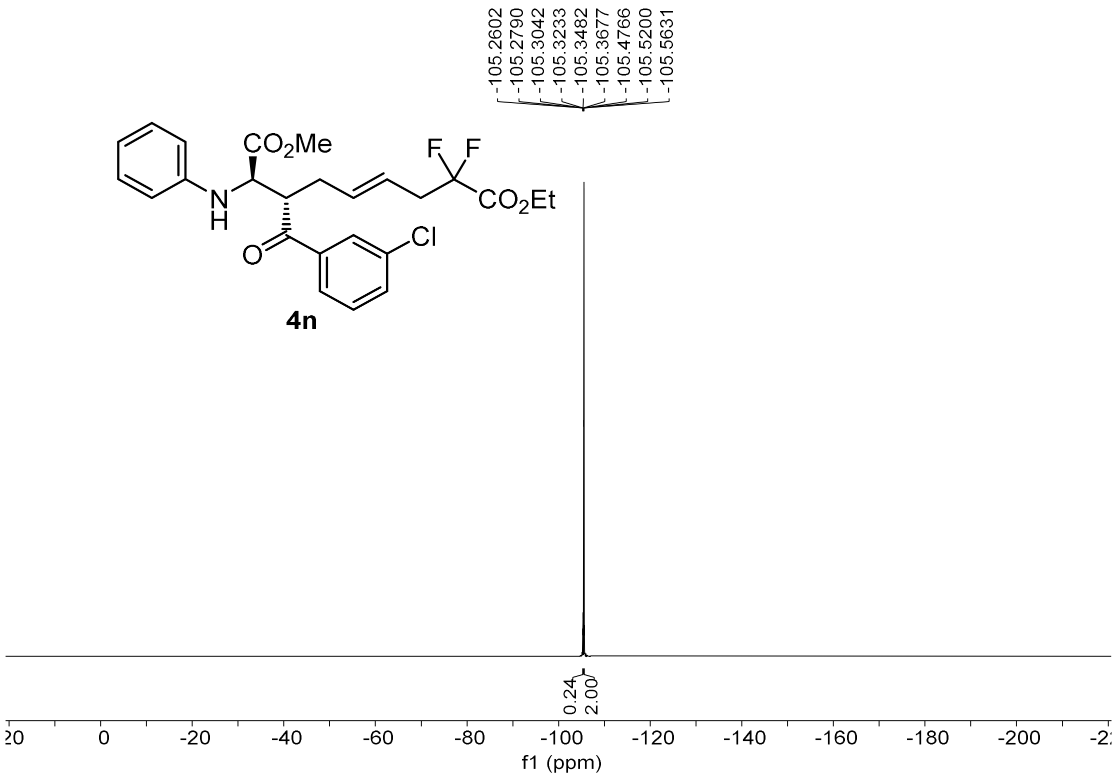

^{19}**F NMR** (376 MHz, CDCl$_3$) of **4n**

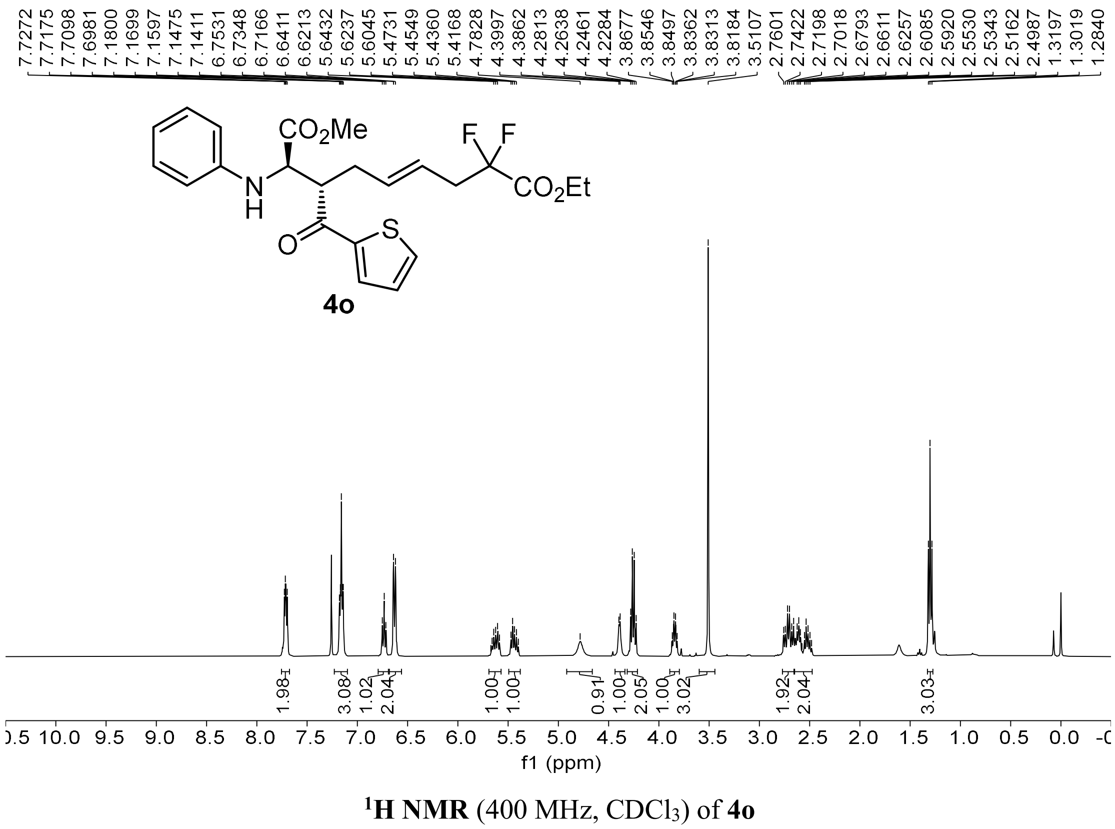

^{1}**H NMR** (400 MHz, CDCl$_3$) of **4o**

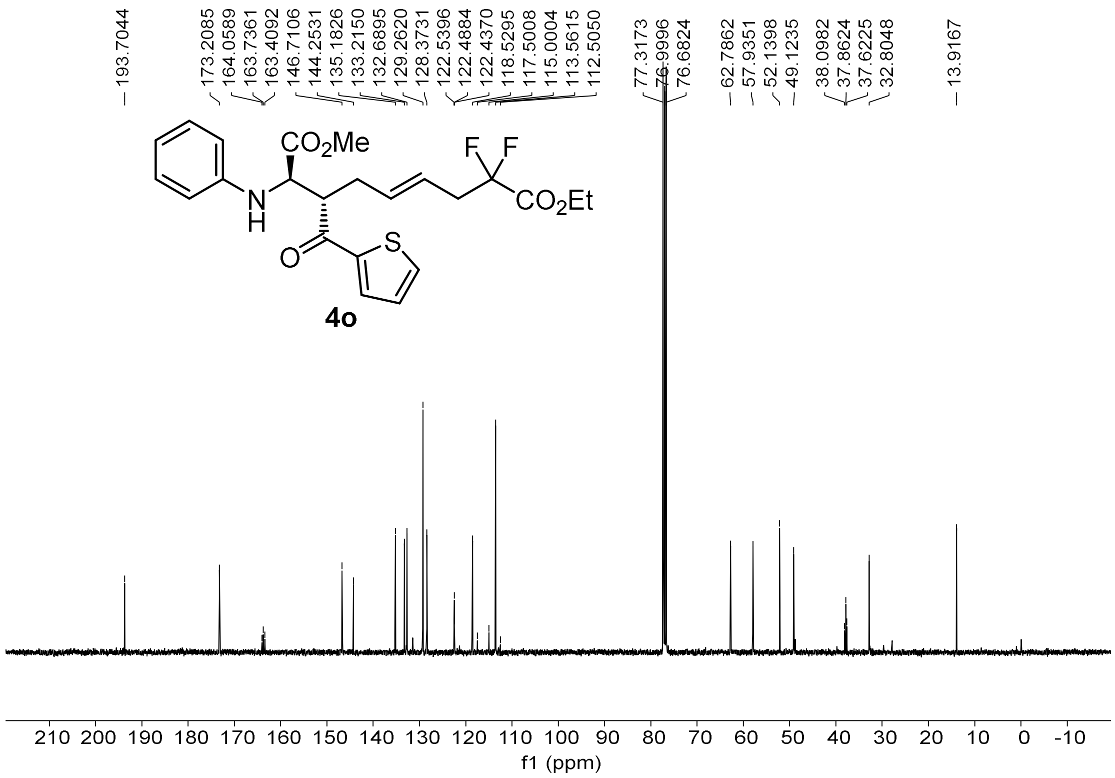

^{13}C NMR (100 MHz, $CDCl_3$) of **4o**

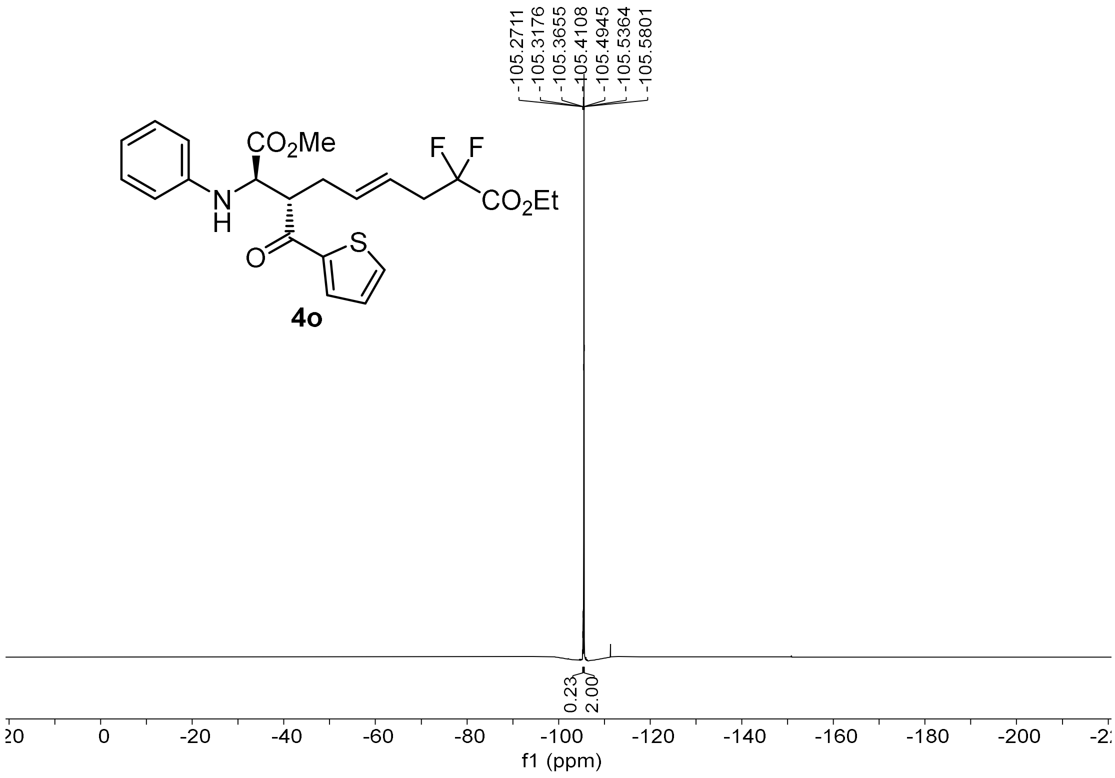

^{19}F NMR (376 MHz, $CDCl_3$) of **4o**

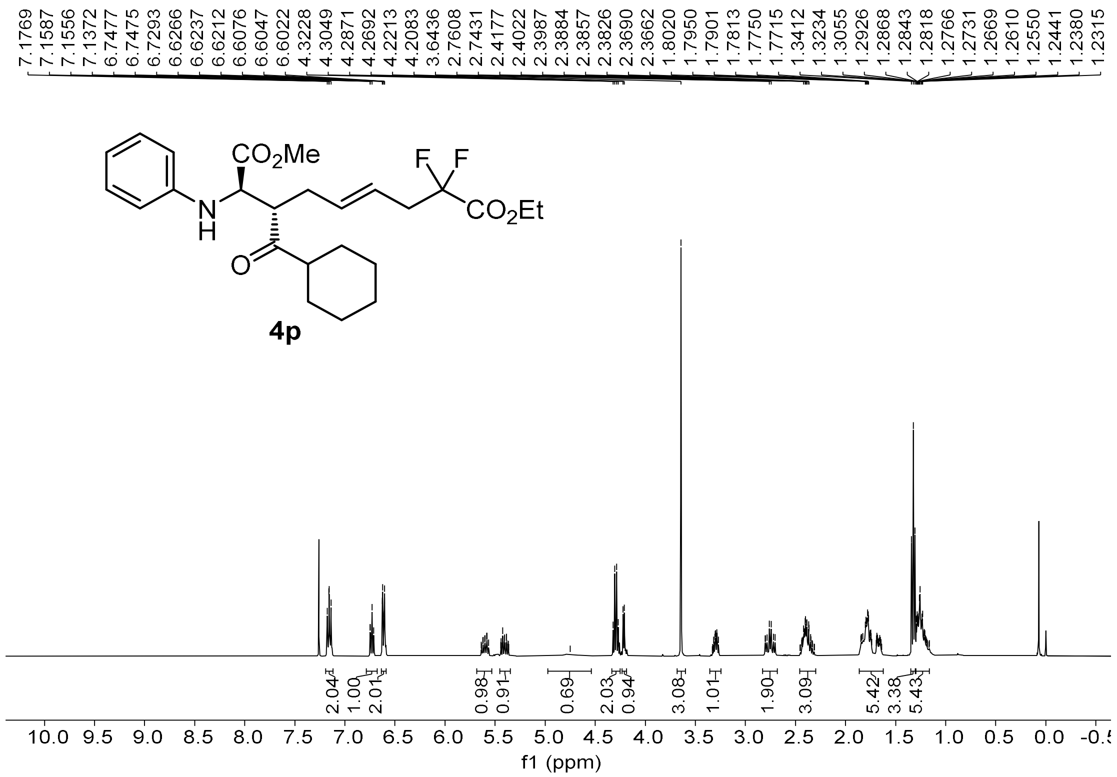

^{1}H NMR (400 MHz, $CDCl_3$) of **4p**

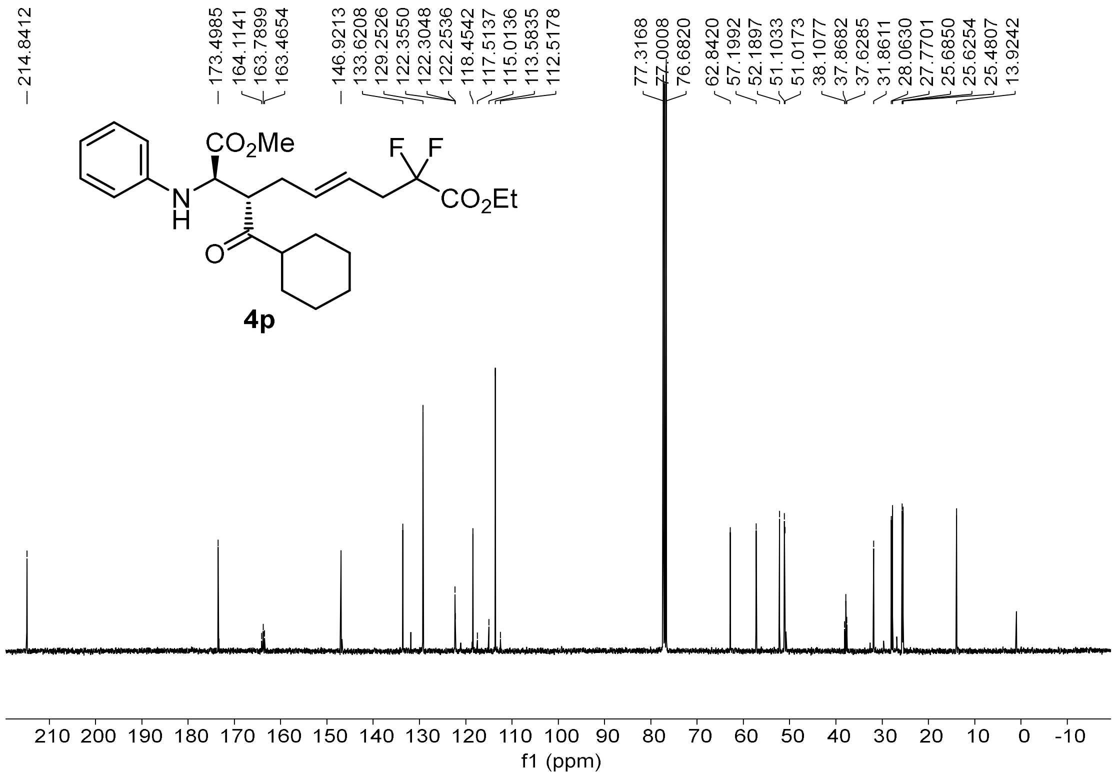

^{13}C NMR (100 MHz, $CDCl_3$) of **4p**

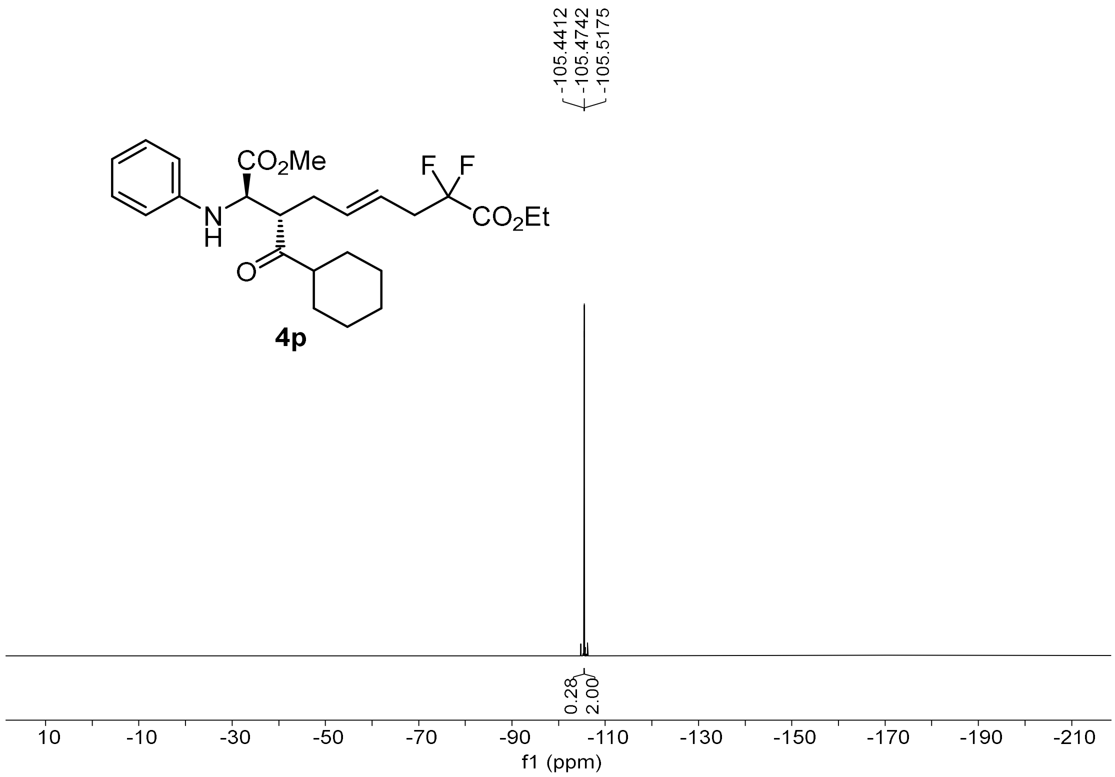

^{19}F NMR (377 MHz, $CDCl_3$) of **4p**

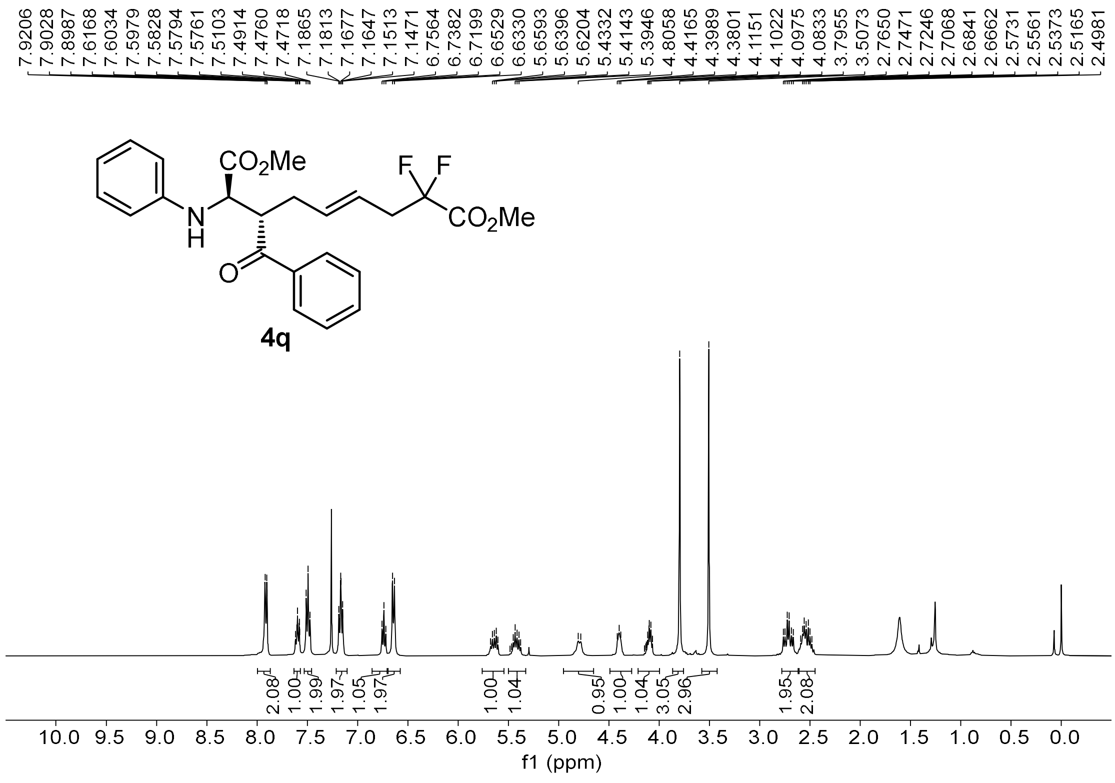

^{1}H NMR (400 MHz, $CDCl_3$) of **4q**

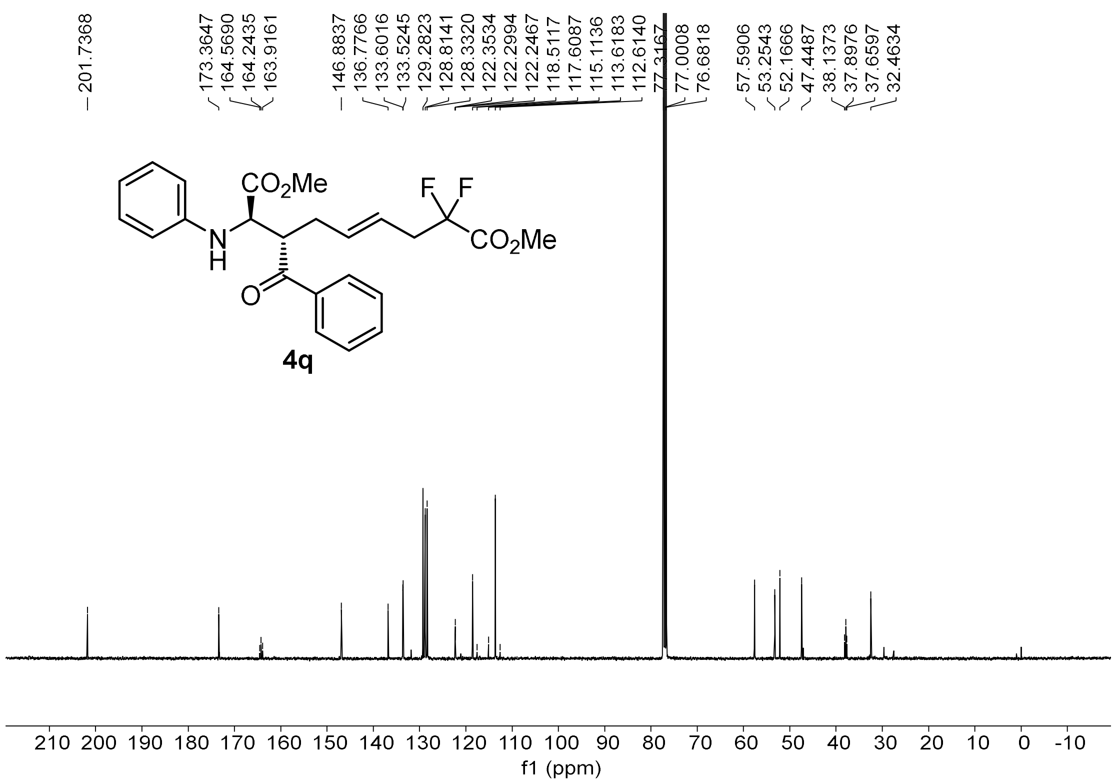

^{13}**C NMR** (100 MHz, $CDCl_3$) of **4q**

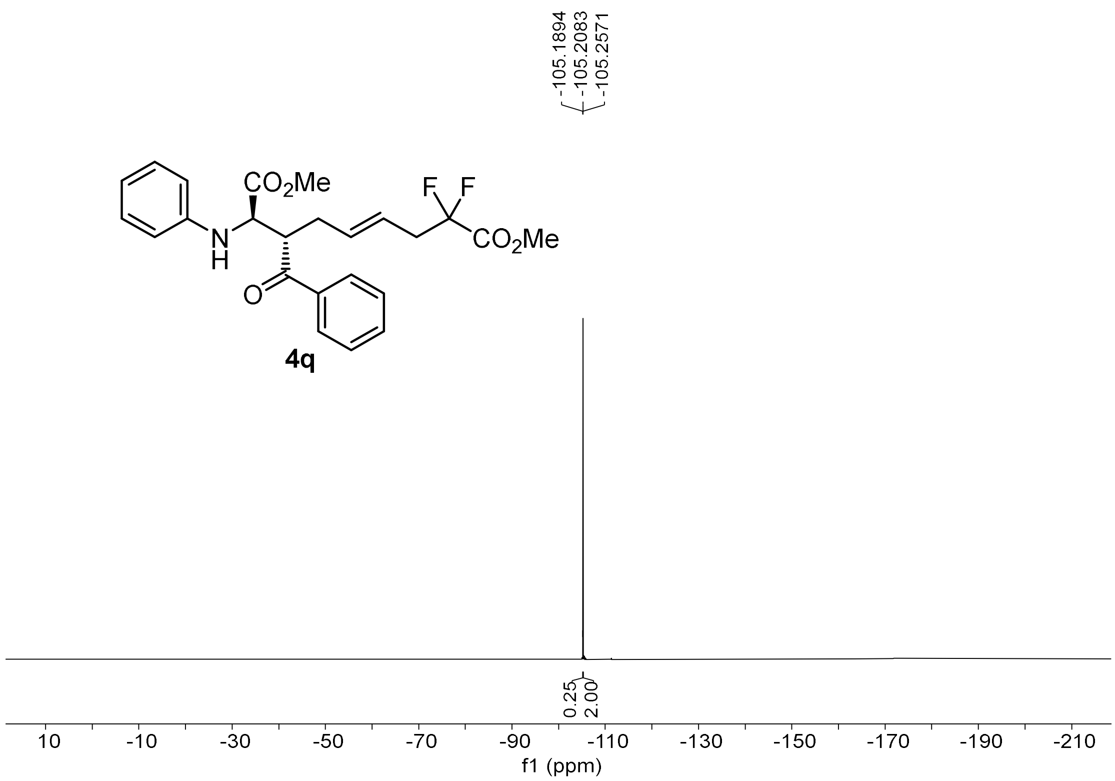

^{19}**F NMR** (377 MHz, $CDCl_3$) of **4q**

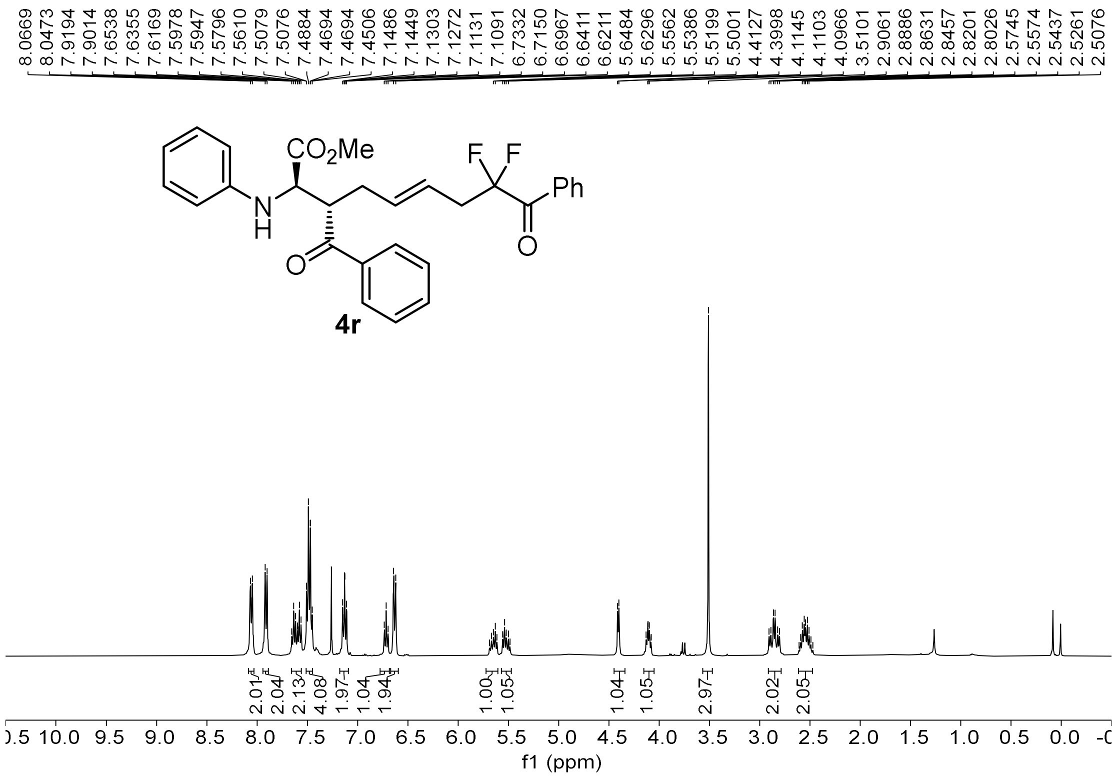

^{1}H NMR (400 MHz, $CDCl_3$) of **4r**

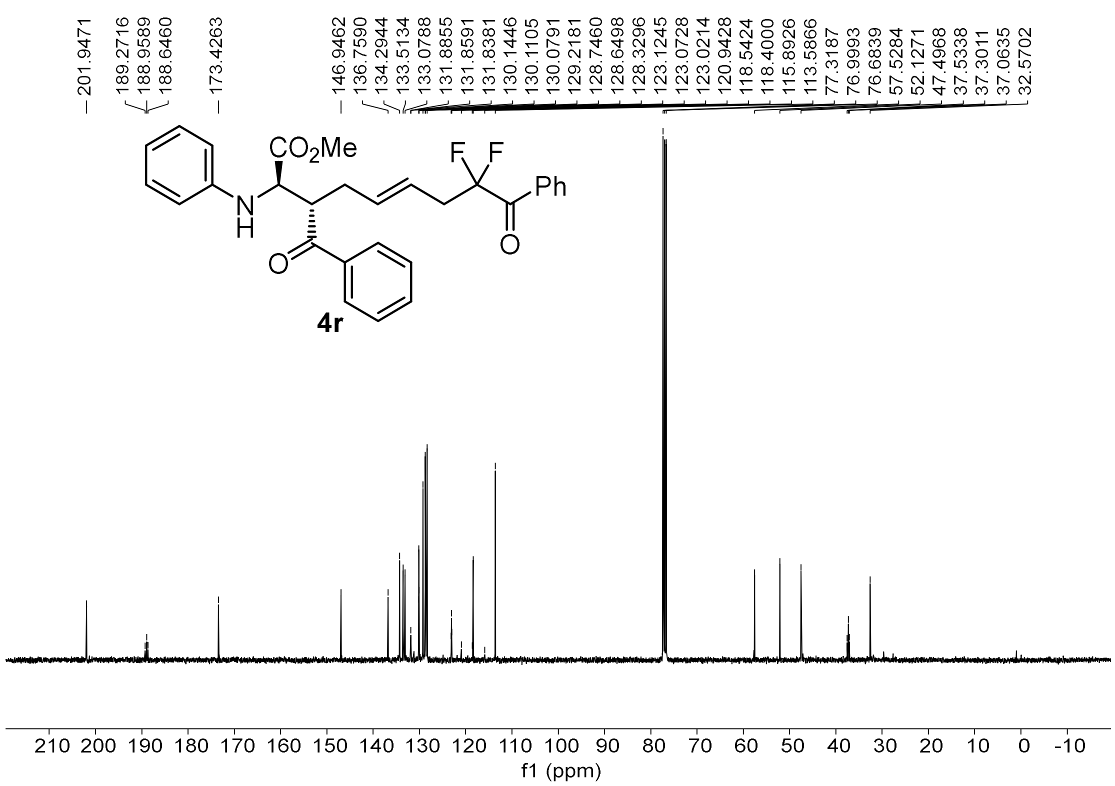

^{13}C NMR (100 MHz, $CDCl_3$) of **4r**

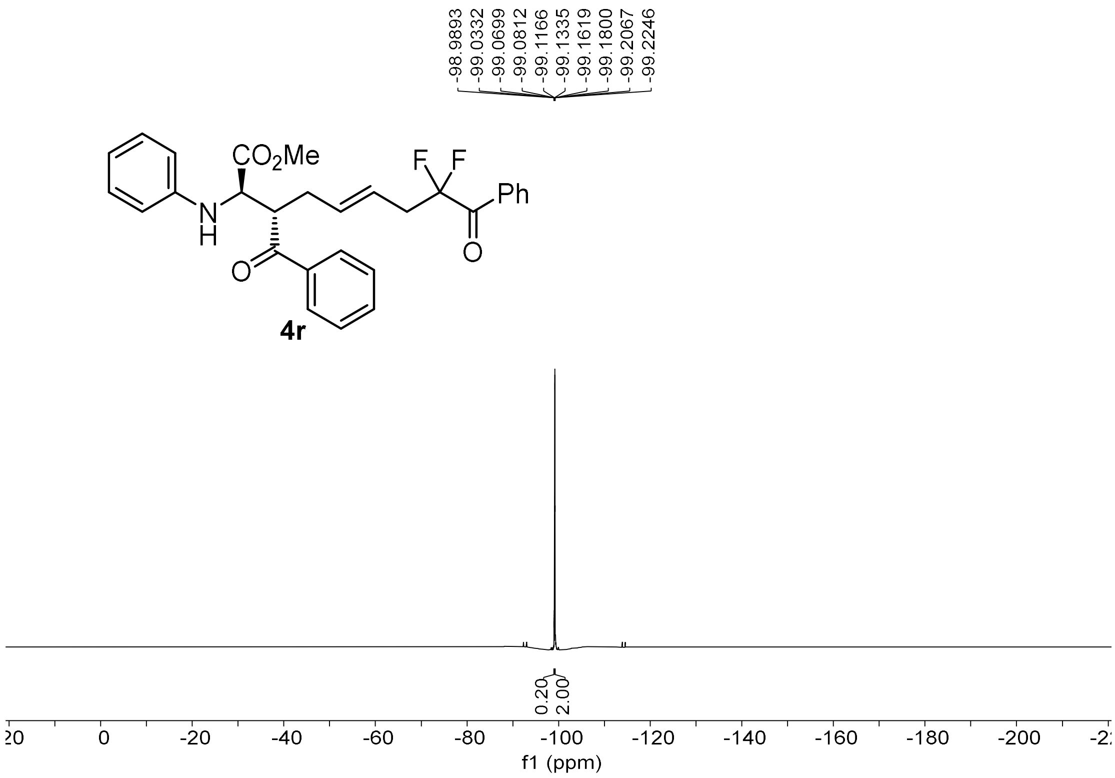

^{19}F NMR (376 MHz, $CDCl_3$) of **4r**

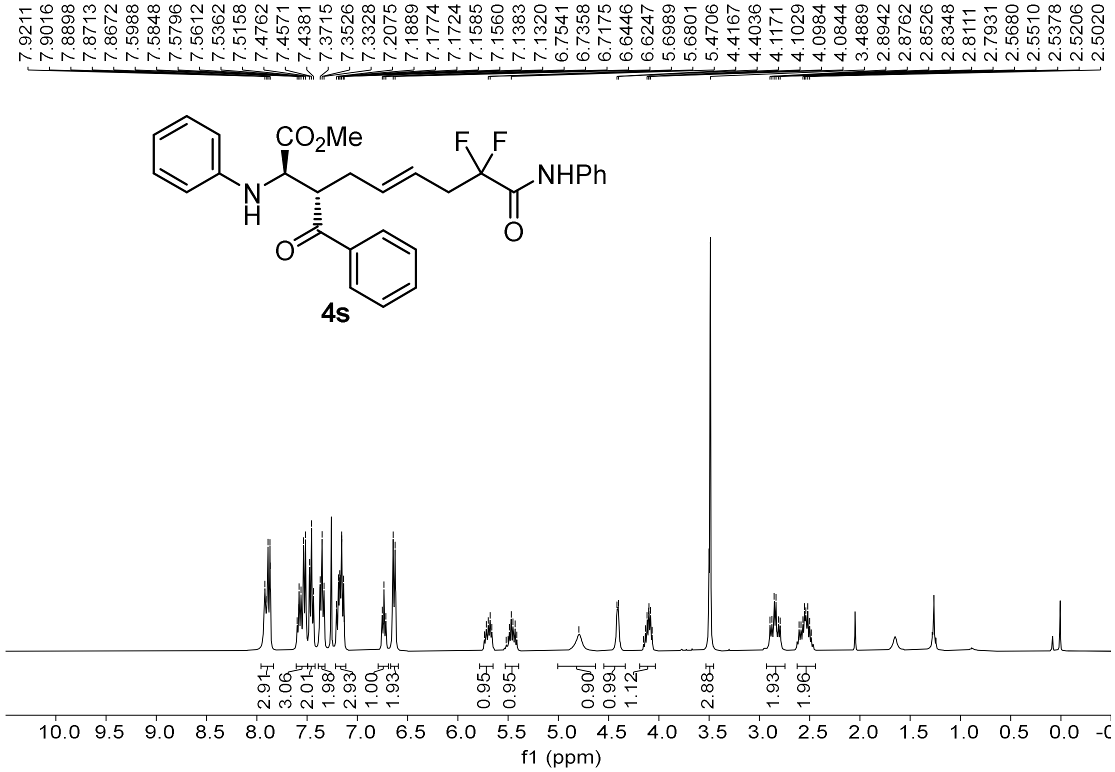

^{1}H NMR (400 MHz, $CDCl_3$) of **4s**

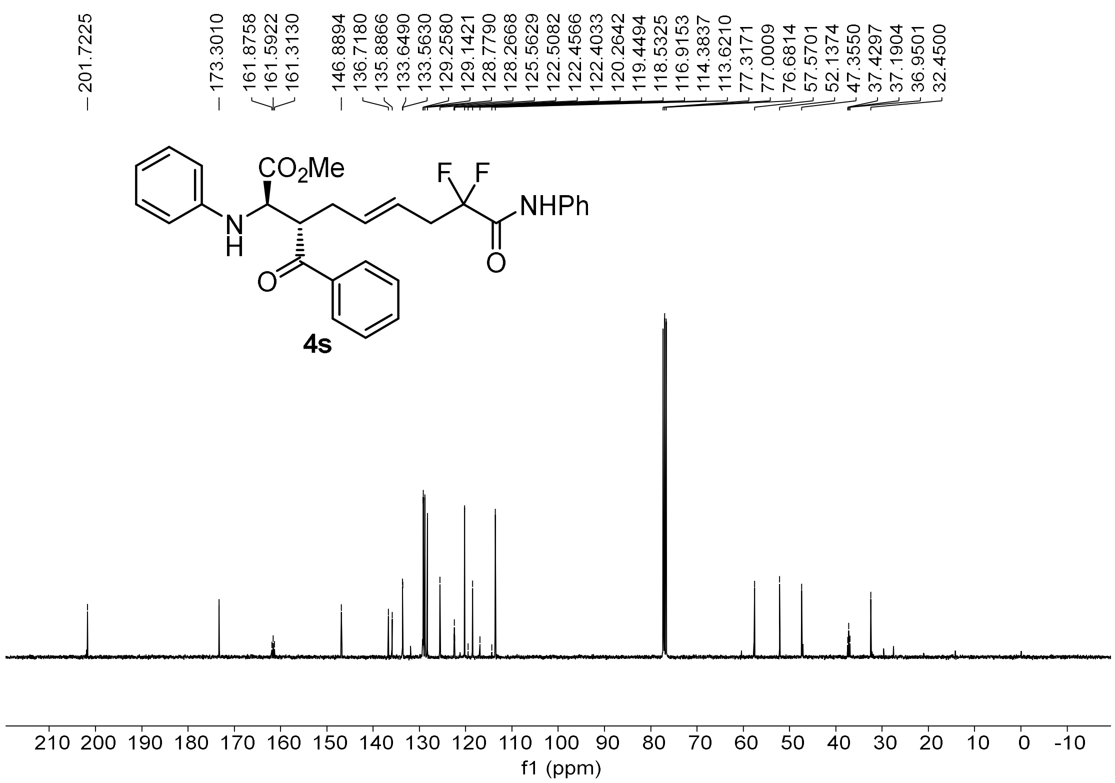

^{13}C NMR (100 MHz, $CDCl_3$) of **4s**

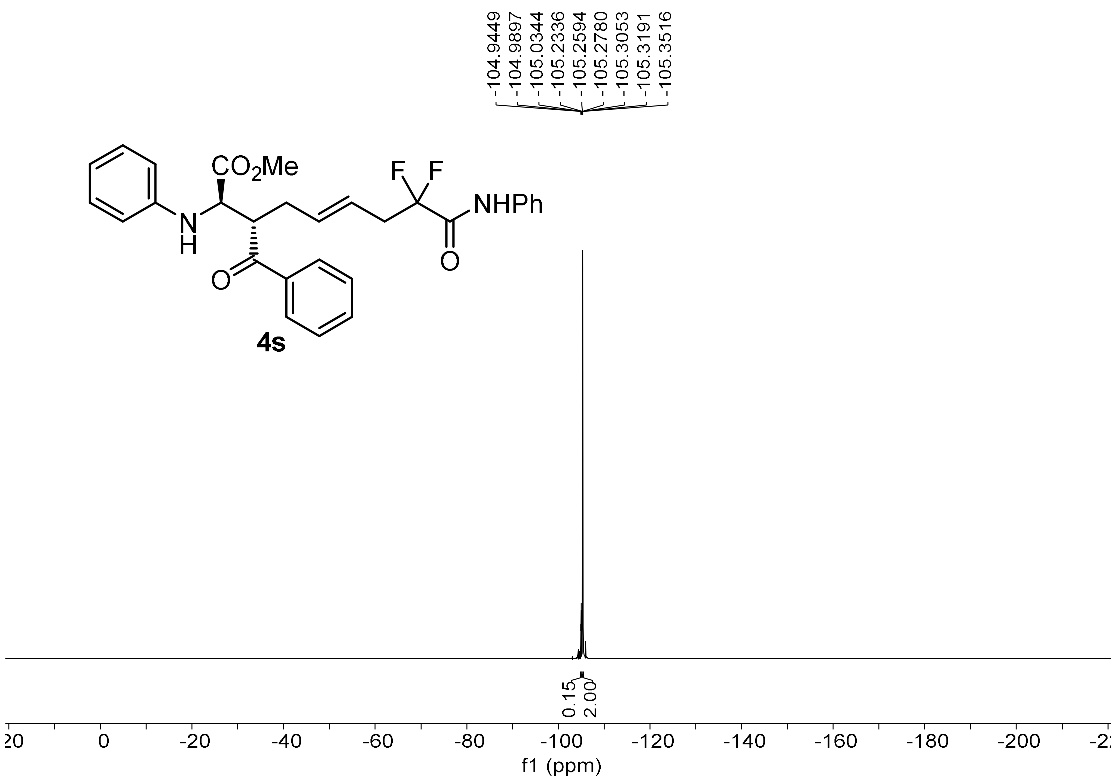

^{19}F NMR (376 MHz, $CDCl_3$) of **4s**

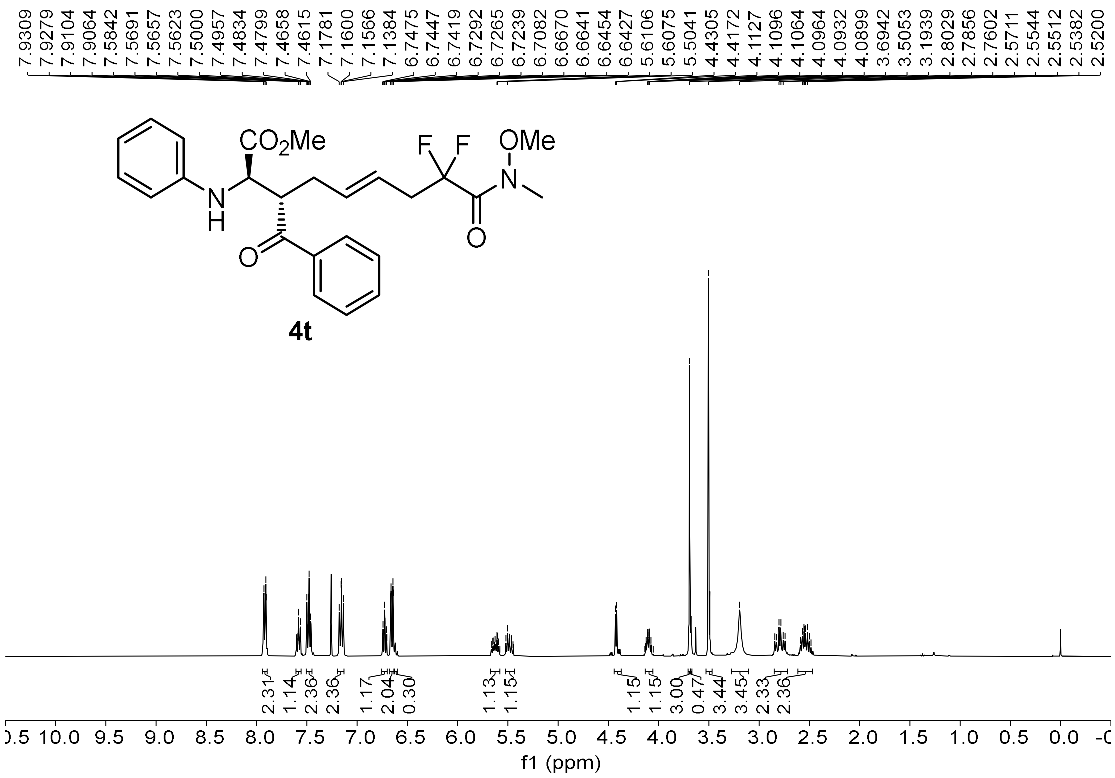

^{1}H NMR (400 MHz, $CDCl_3$) of **4t**

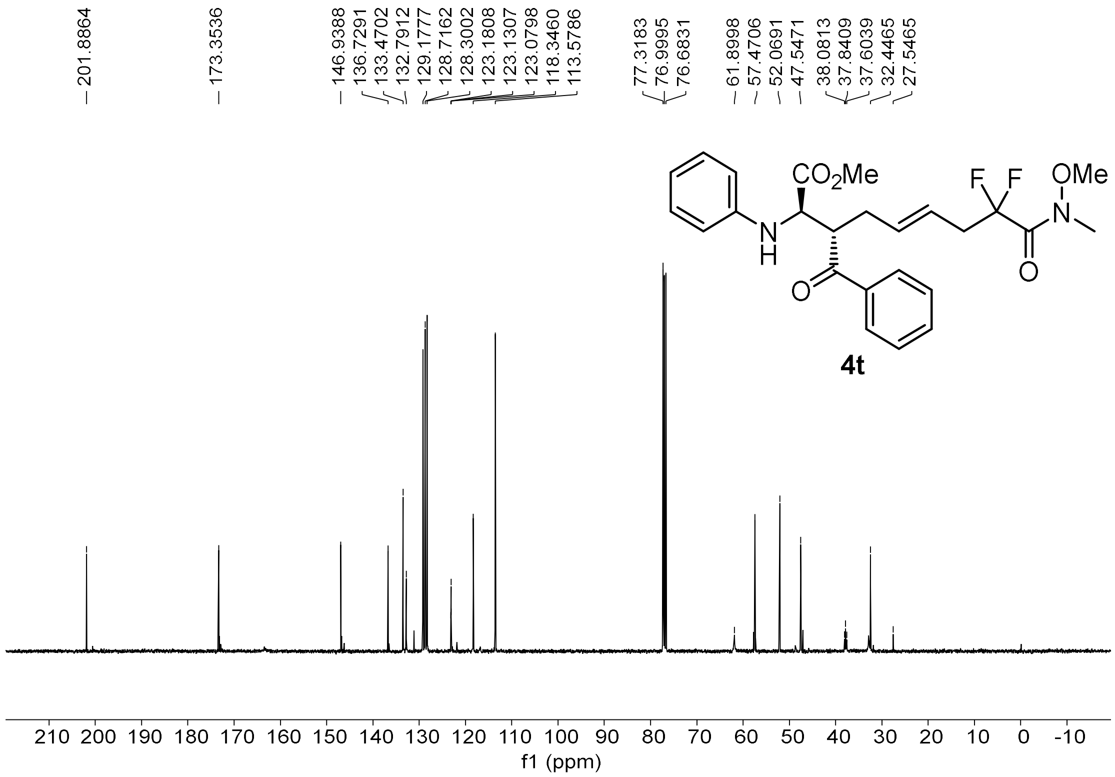

^{13}C NMR (100 MHz, $CDCl_3$) of **4t**

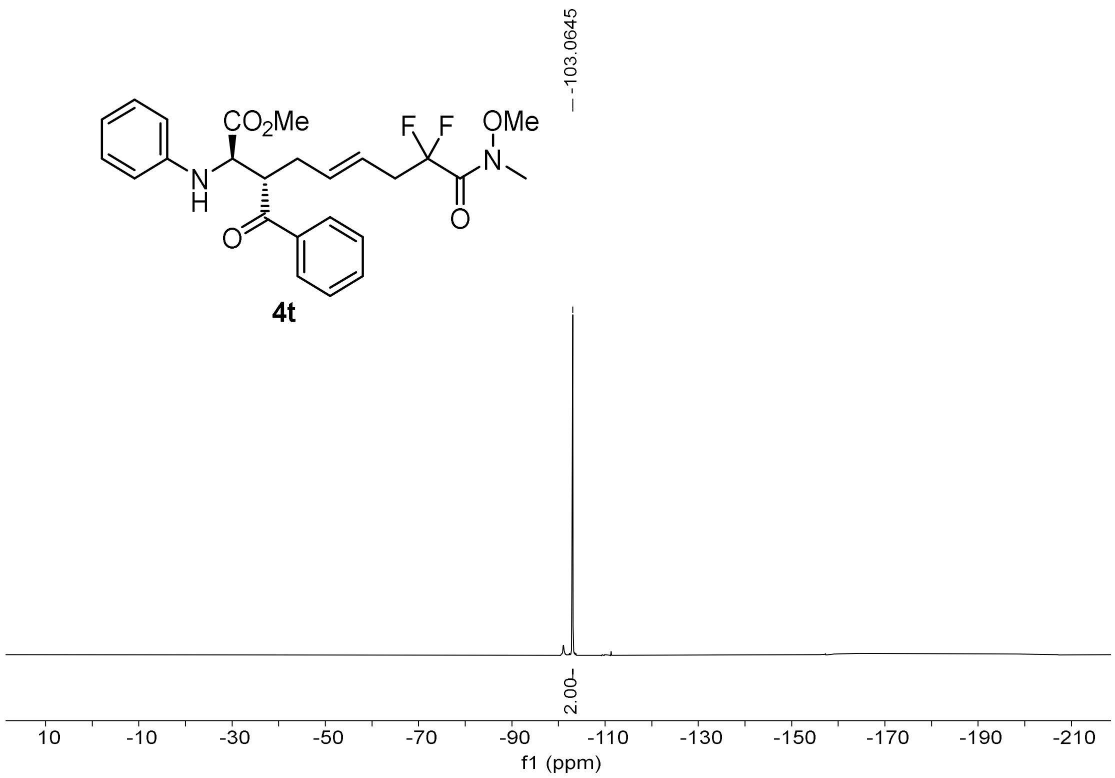

^{19}F NMR (377 MHz, $CDCl_3$) of **4t**

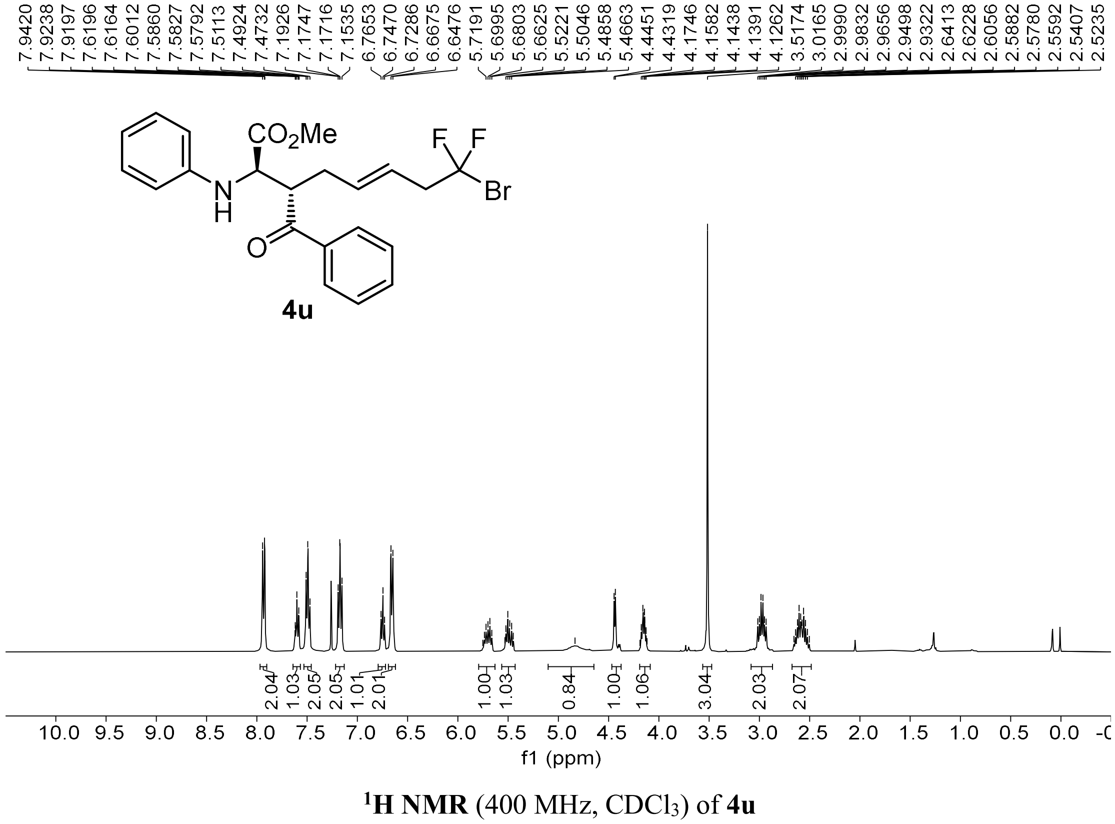

^{1}H NMR (400 MHz, $CDCl_3$) of **4u**

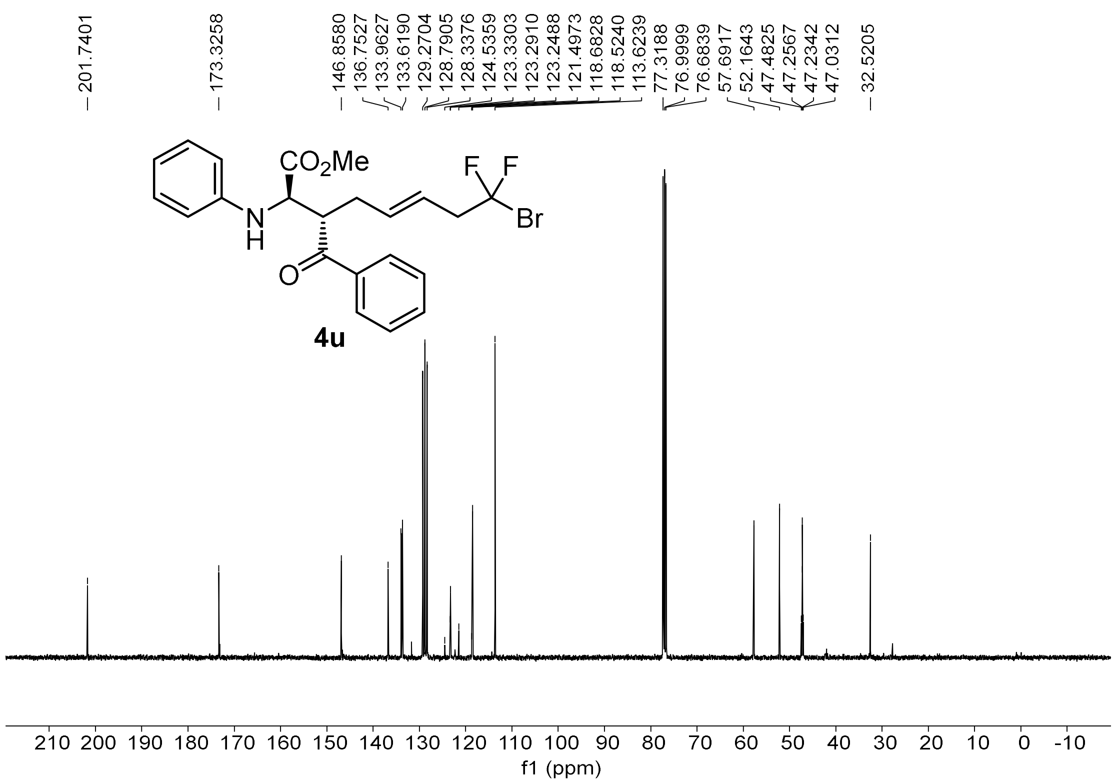

[13]**C NMR** (100 MHz, $CDCl_3$) of **4u**

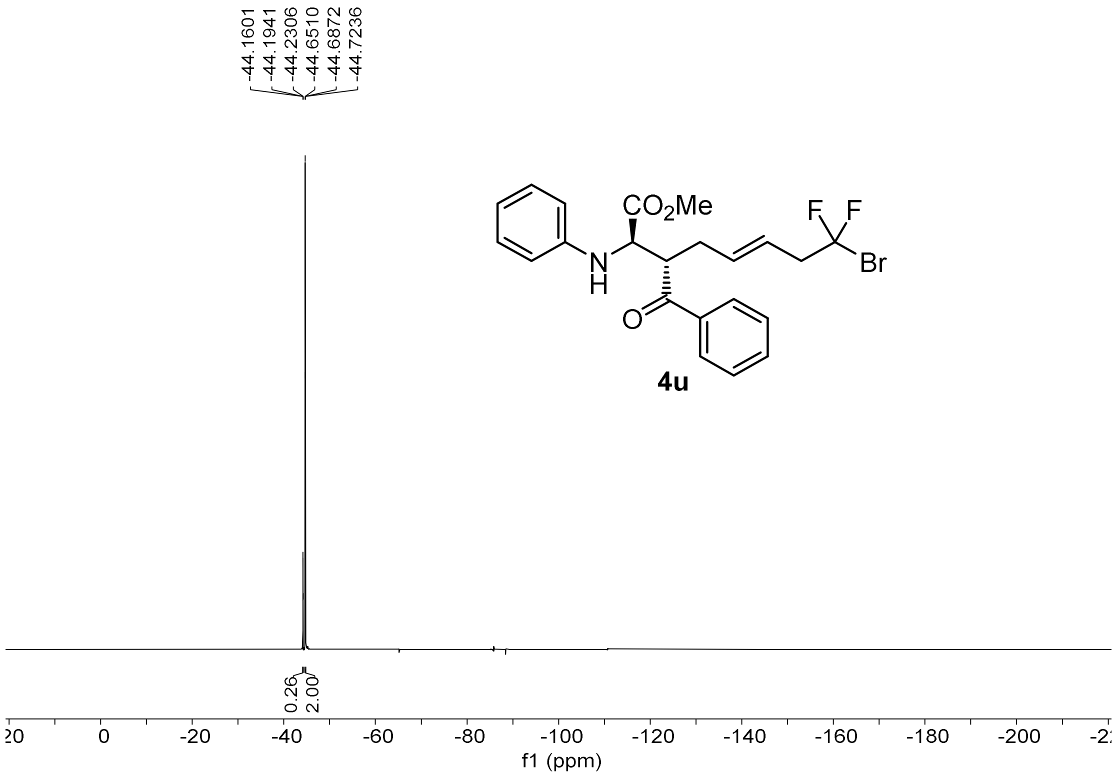

[19]**F NMR** (376 MHz, $CDCl_3$) of **4u**

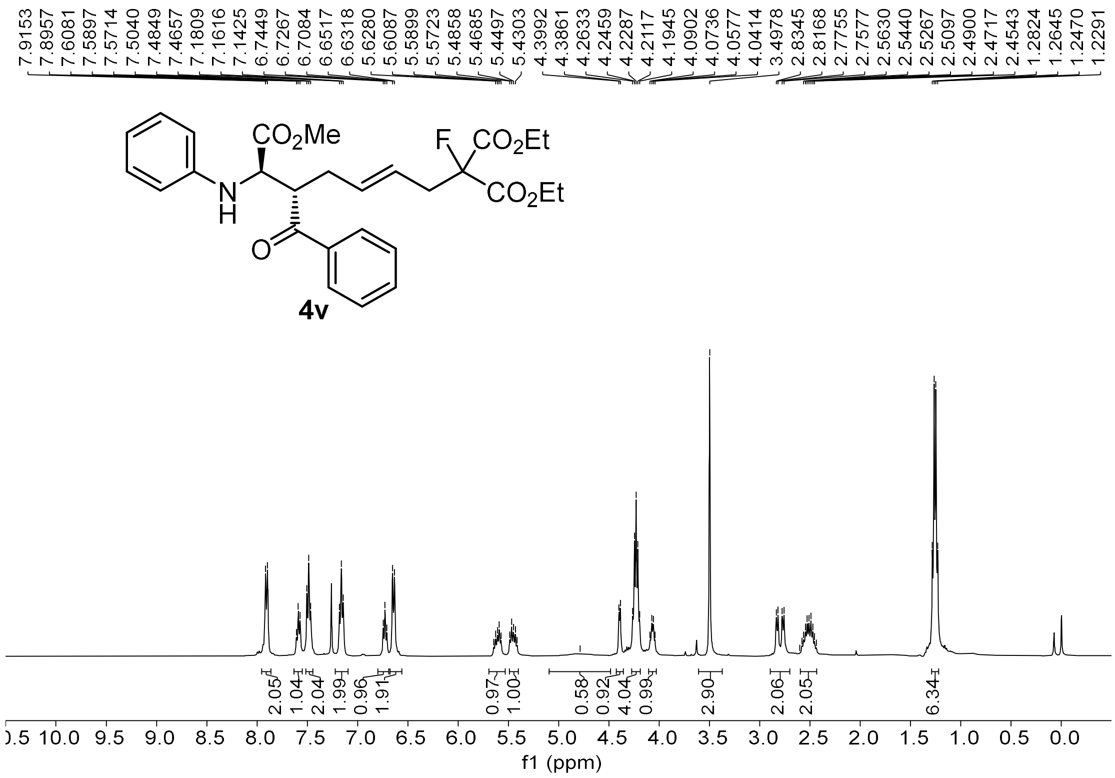

^{1}H NMR (400 MHz, $CDCl_3$) of **4v**

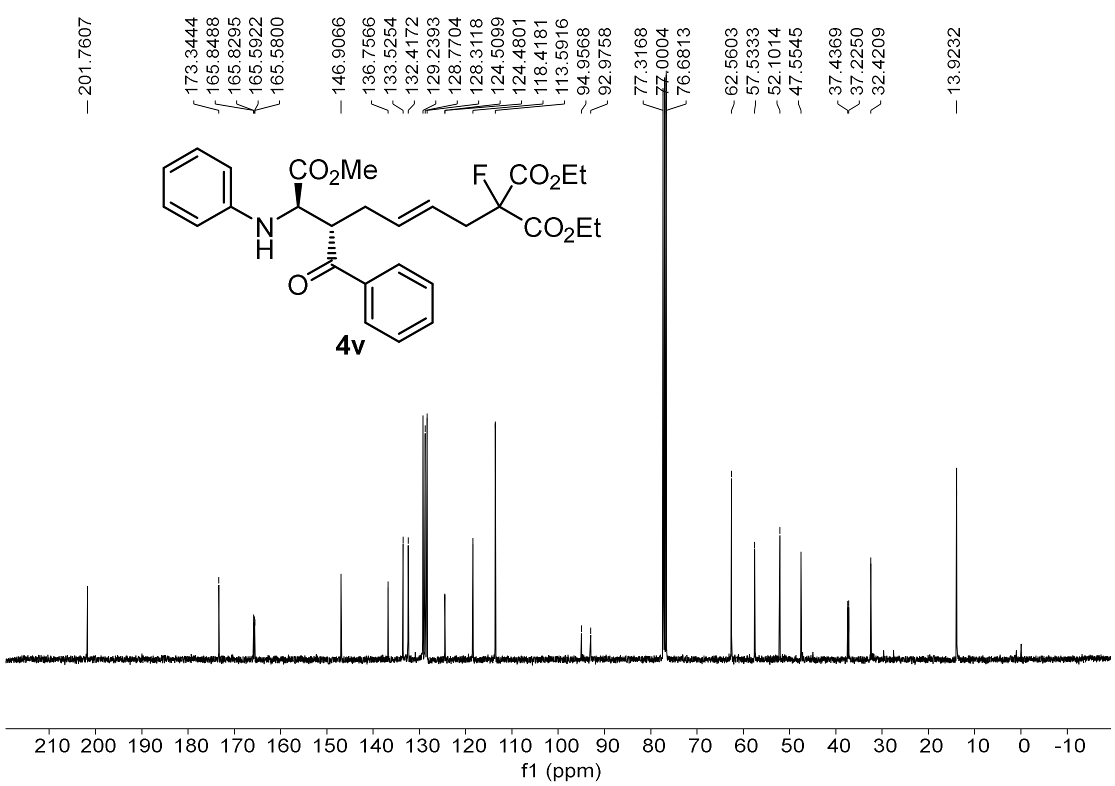

^{13}C NMR (100 MHz, $CDCl_3$) of **4v**

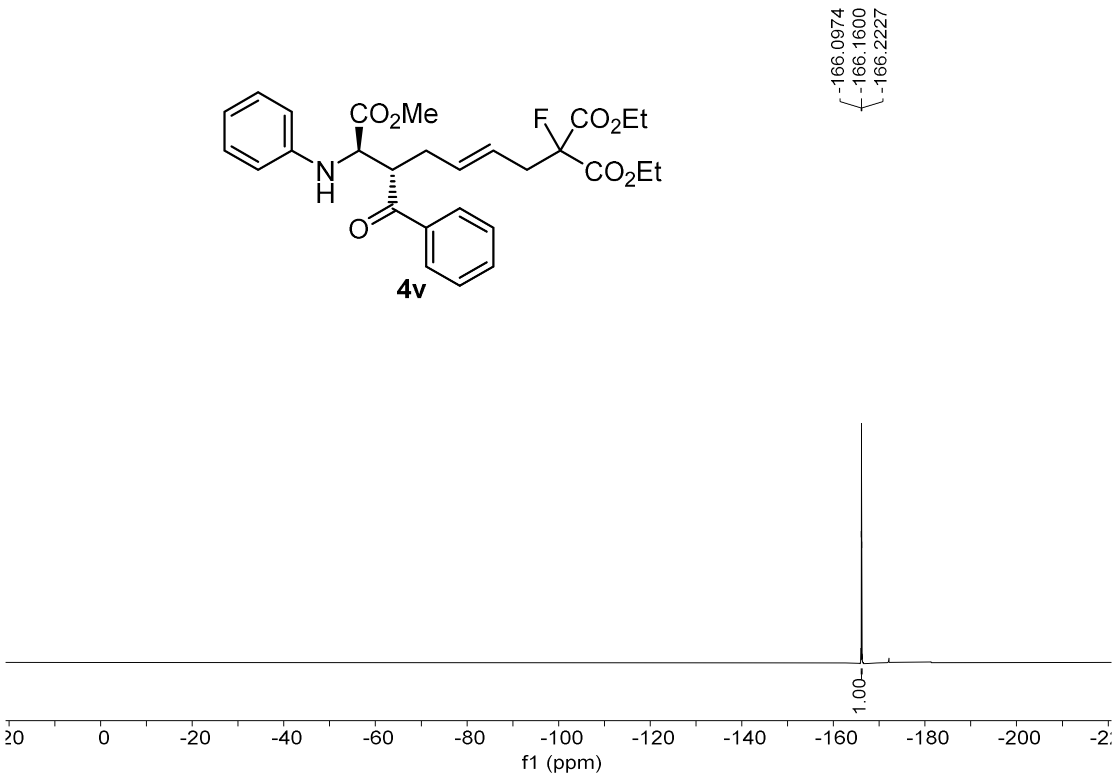

^{19}F NMR (376 MHz, $CDCl_3$) of **4v**

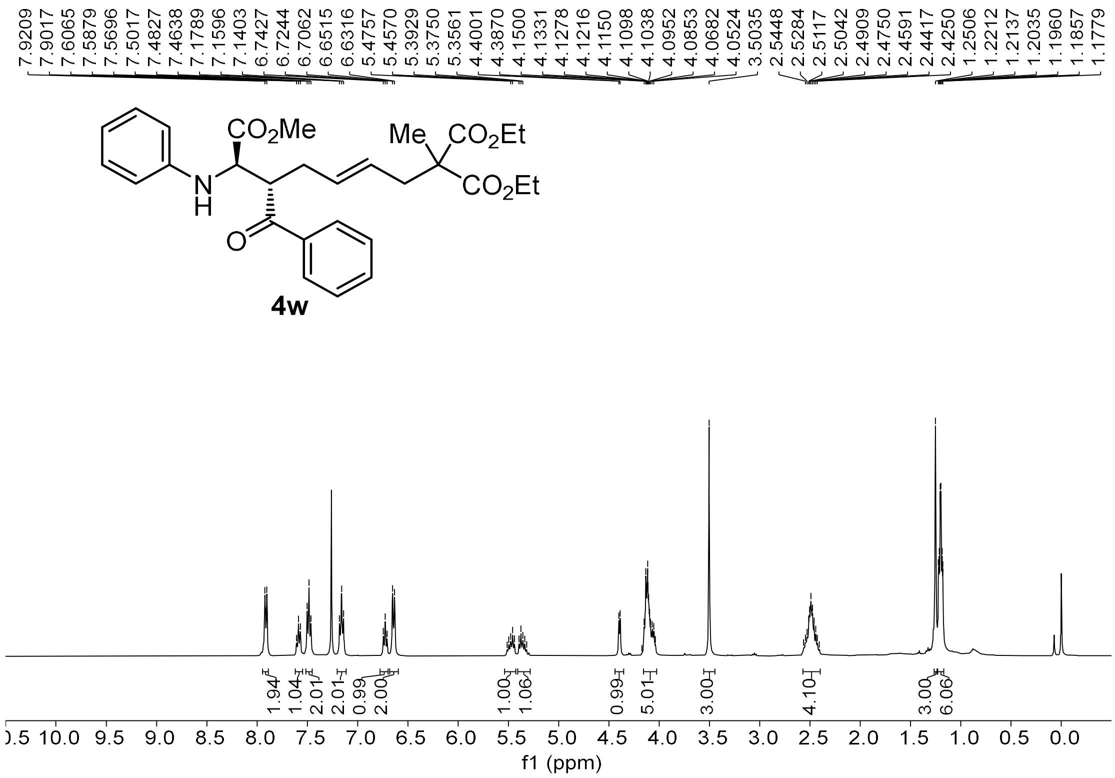

^{1}H NMR (400 MHz, $CDCl_3$) of **4w**

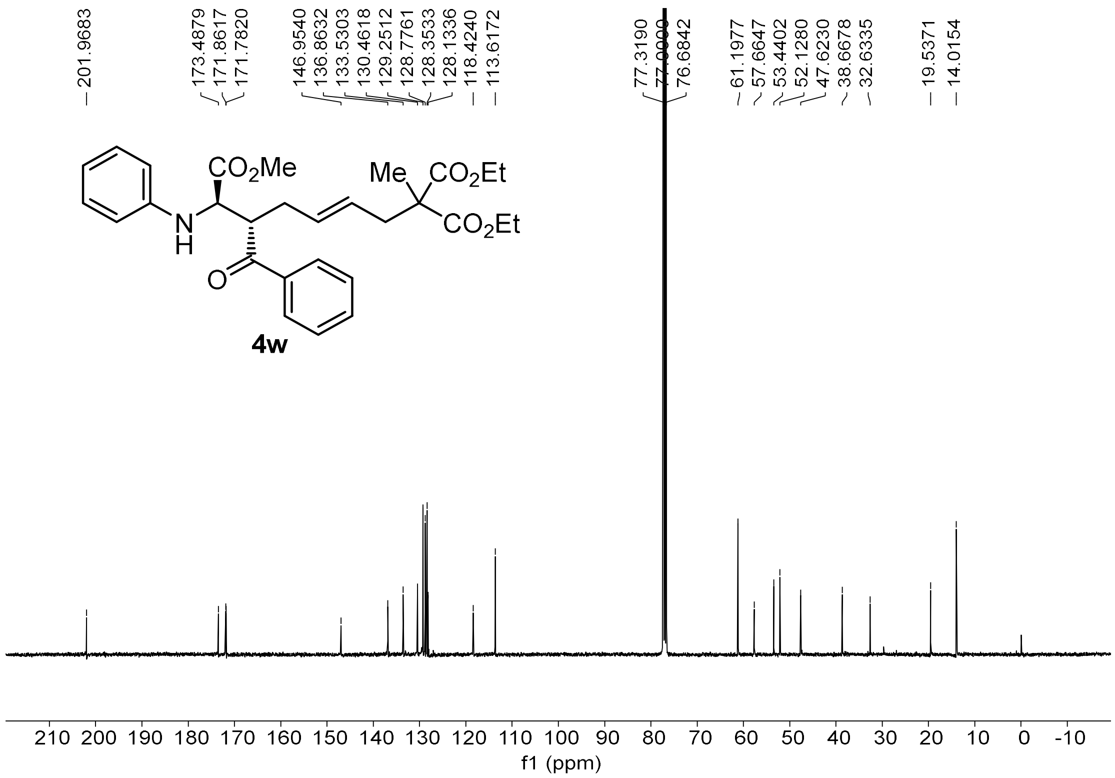

^{13}C NMR (100 MHz, $CDCl_3$) of **4w**

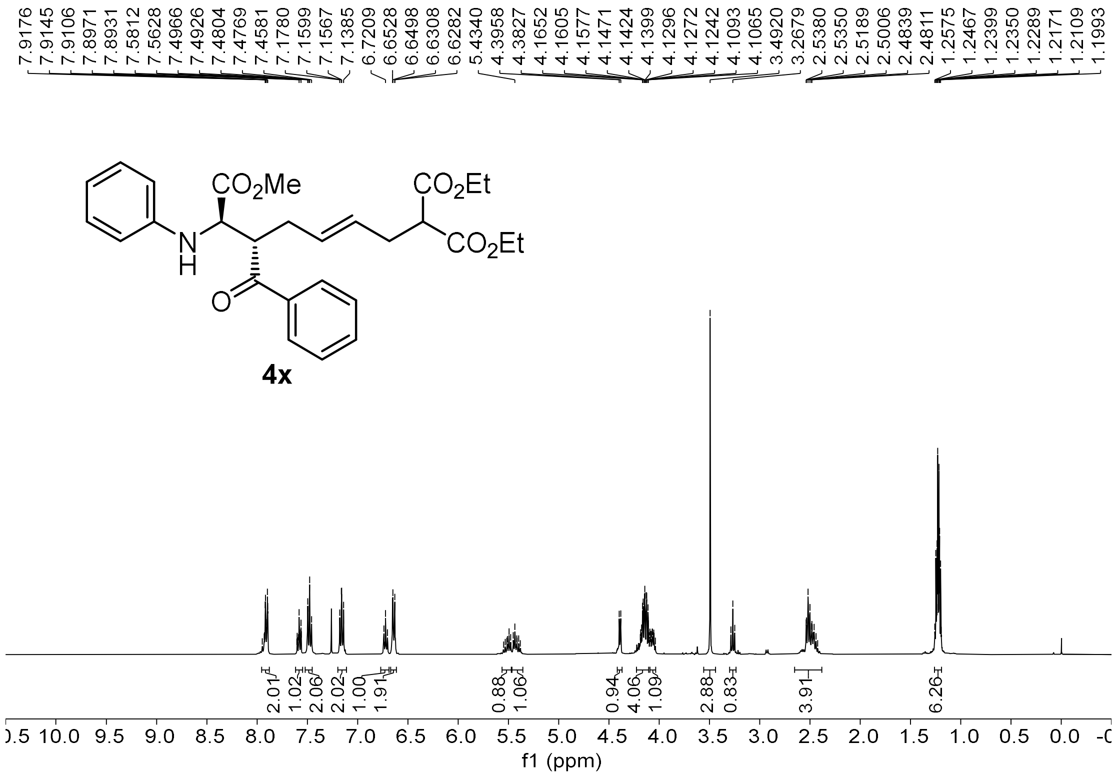

^{1}H NMR (400 MHz, $CDCl_3$) of **4x**

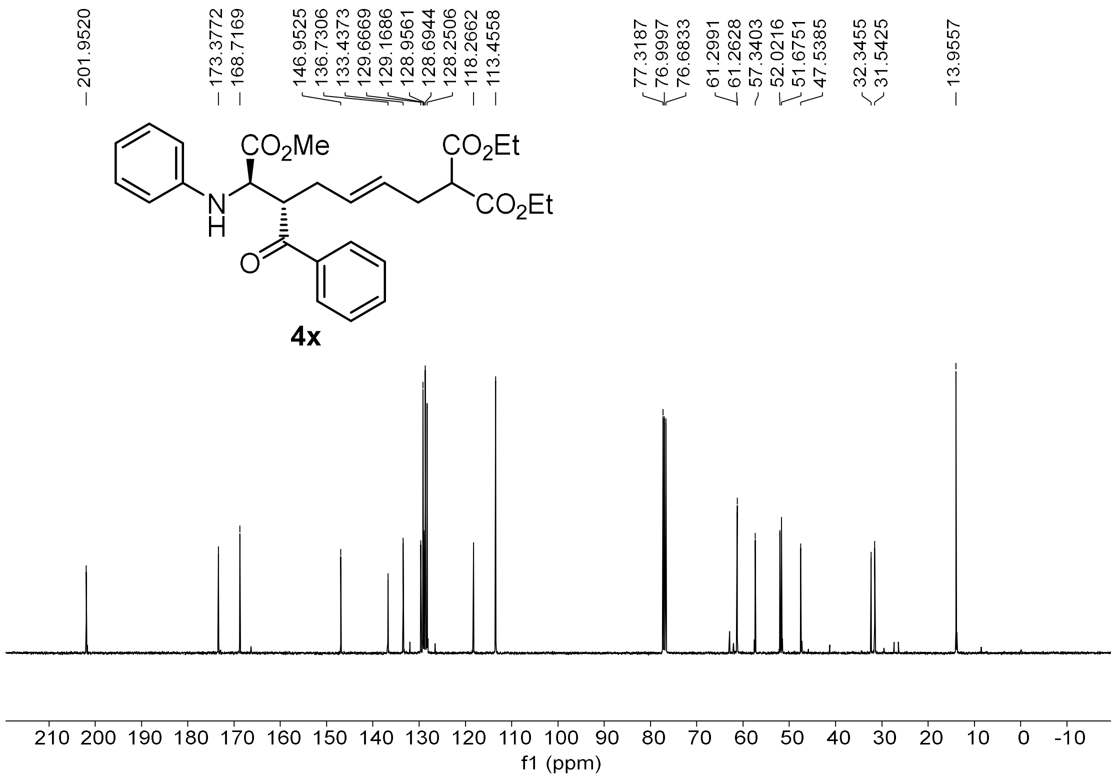

^{13}C NMR (100 MHz, CDCl$_3$) of **4x**

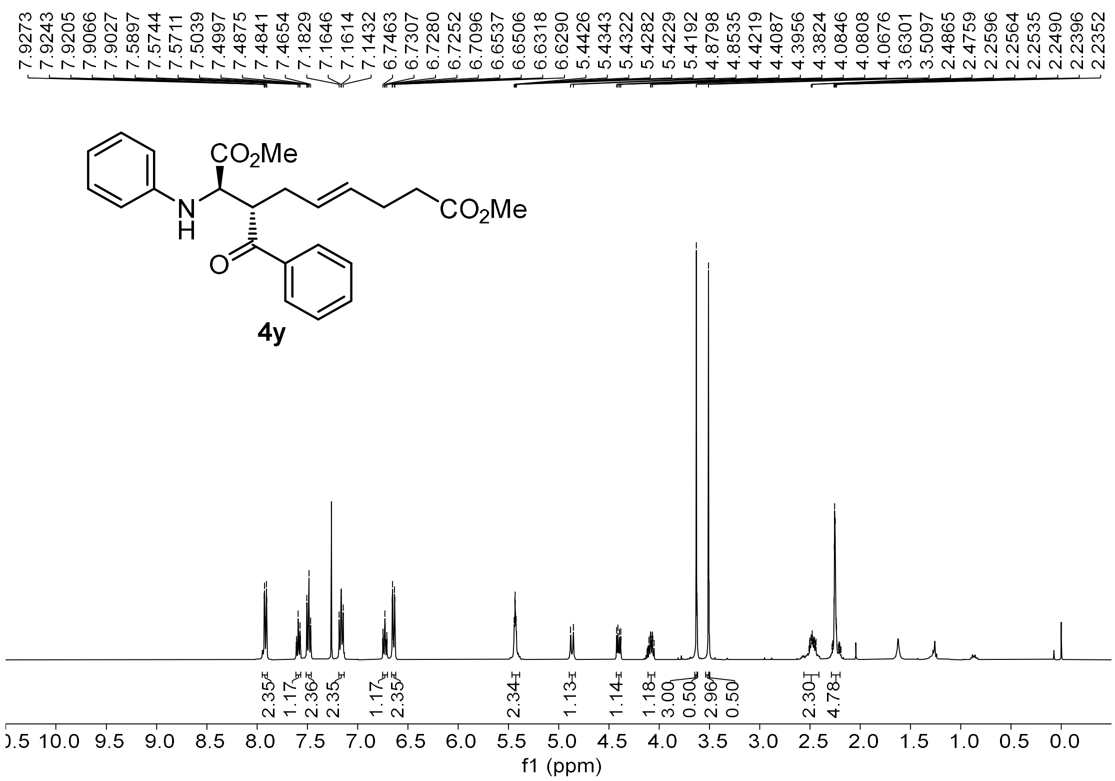

^{1}H NMR (400 MHz, CDCl$_3$) of **4y**

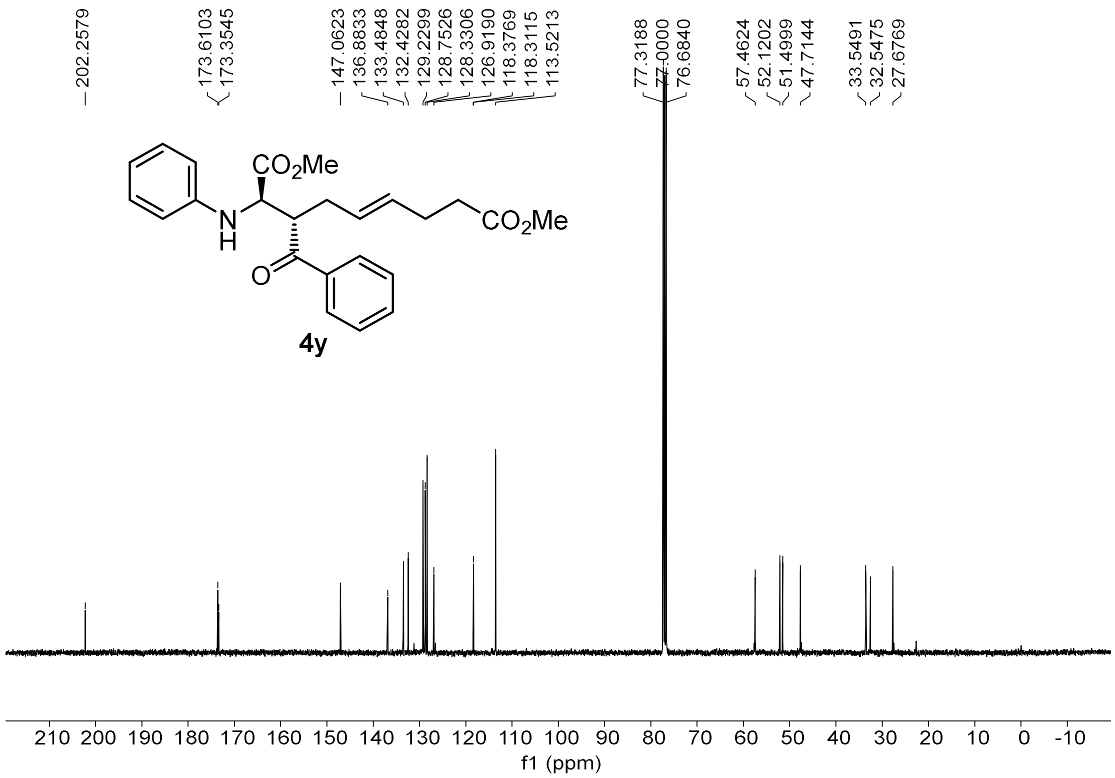

^{13}C NMR (100 MHz, $CDCl_3$) of **4y**

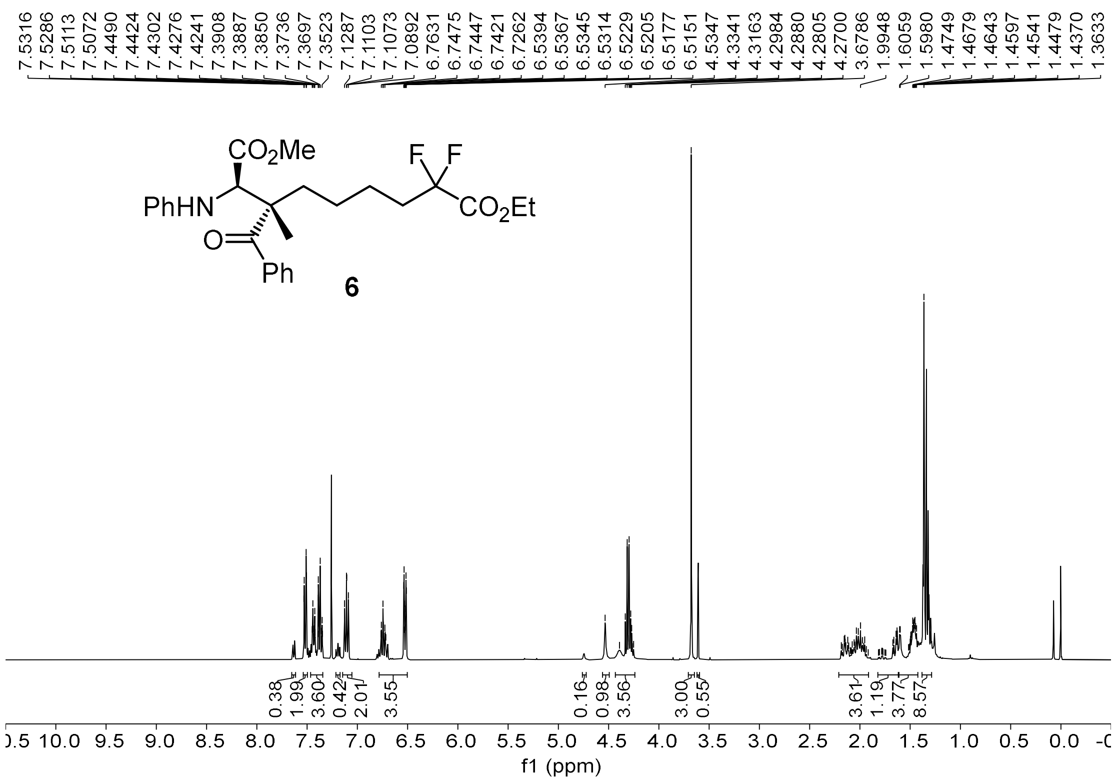

^{1}H NMR (400 MHz, $CDCl_3$) of **6**

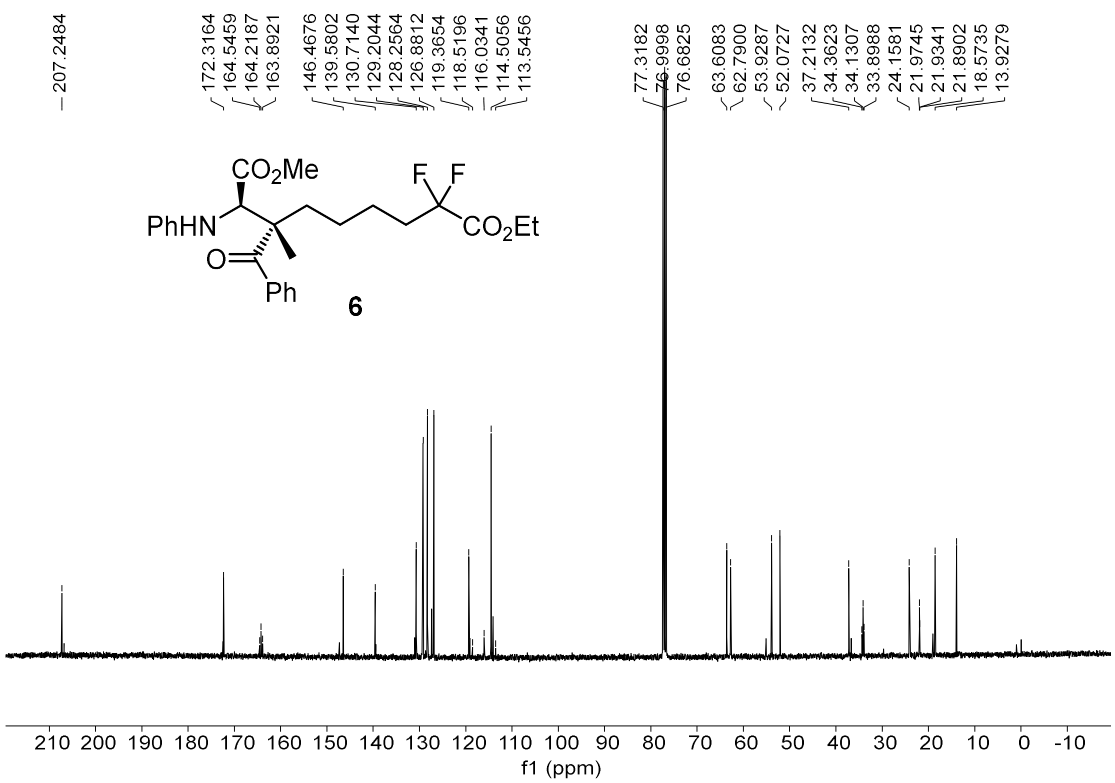

^{13}C NMR (100 MHz, $CDCl_3$) of **6**

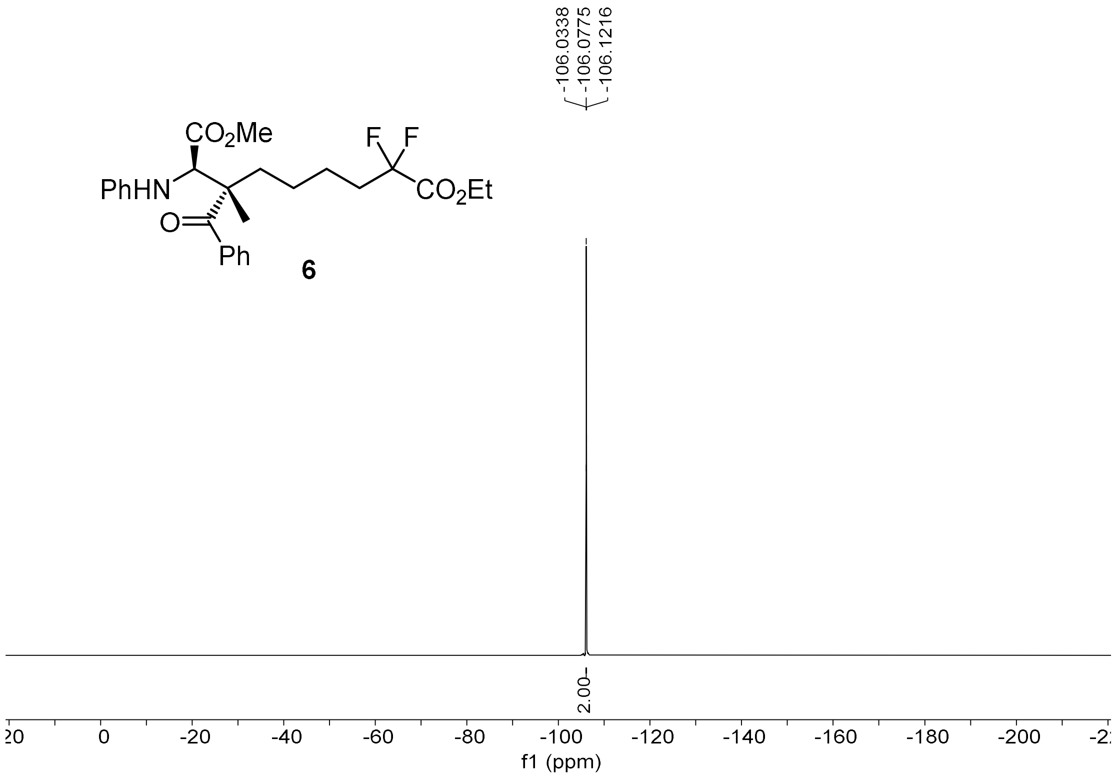

^{19}F NMR (376 MHz, $CDCl_3$) of **6**

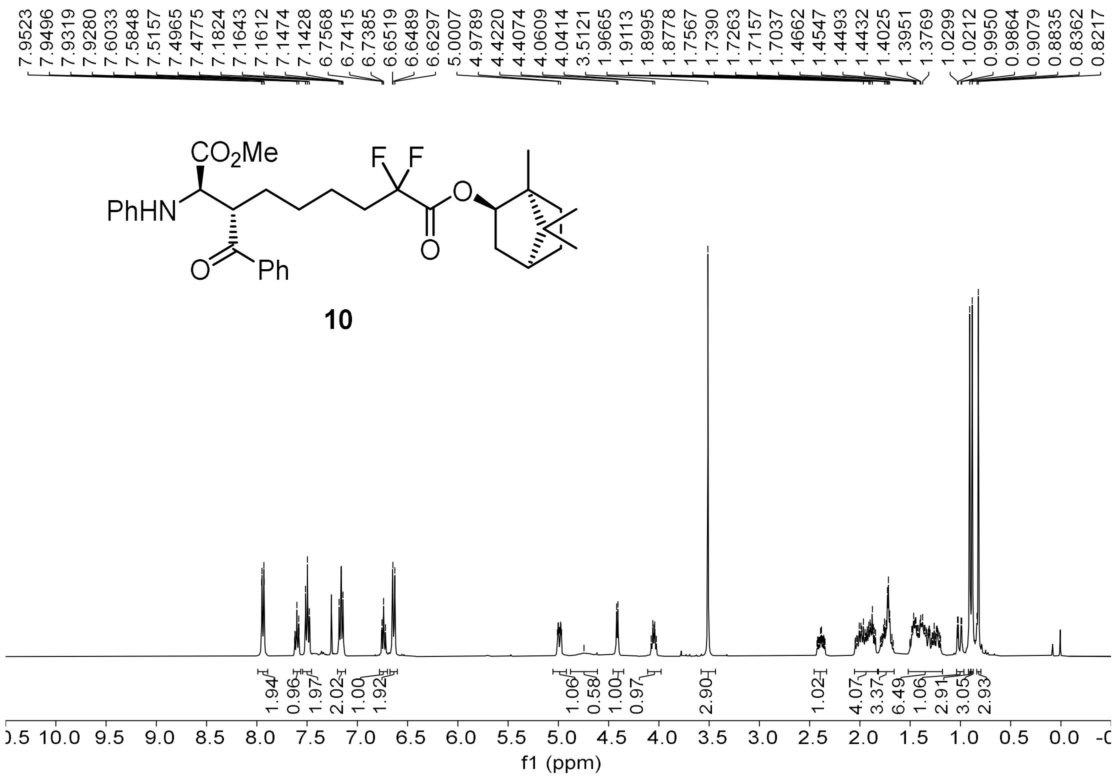

^{1}H NMR (400 MHz, $CDCl_3$) of **10**

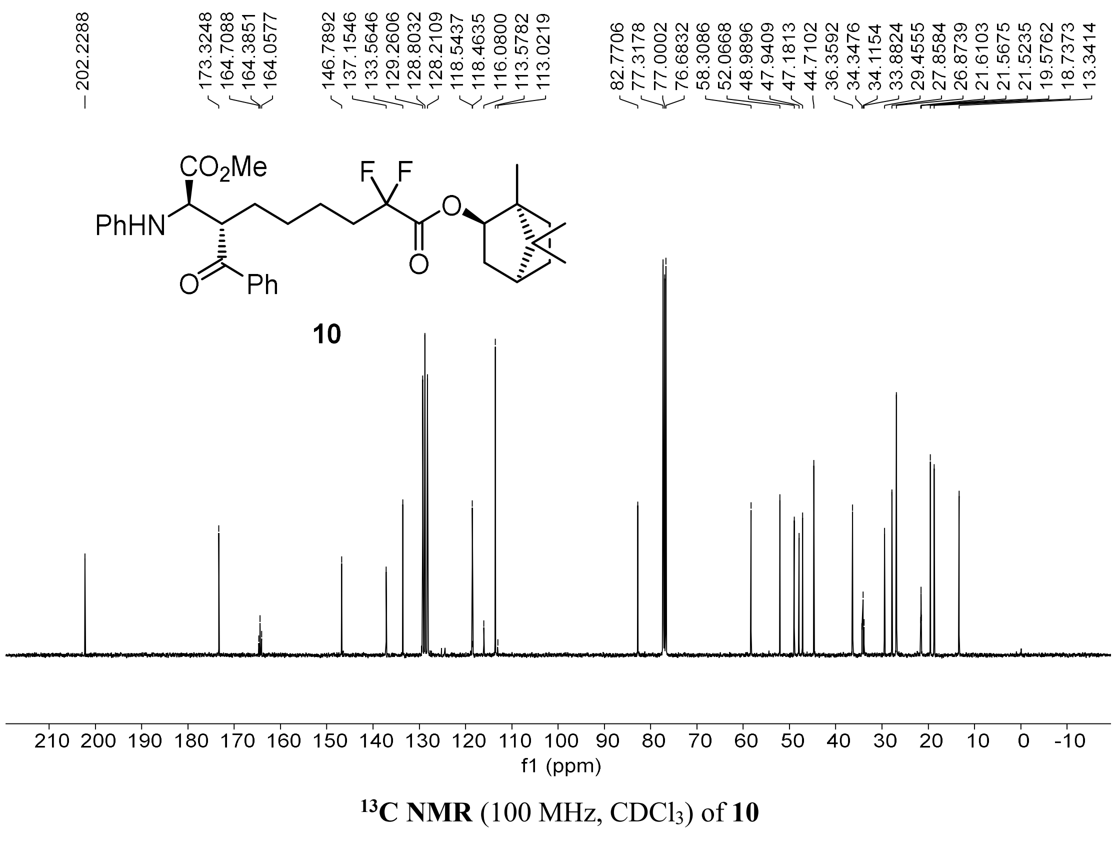

^{13}C NMR (100 MHz, $CDCl_3$) of **10**

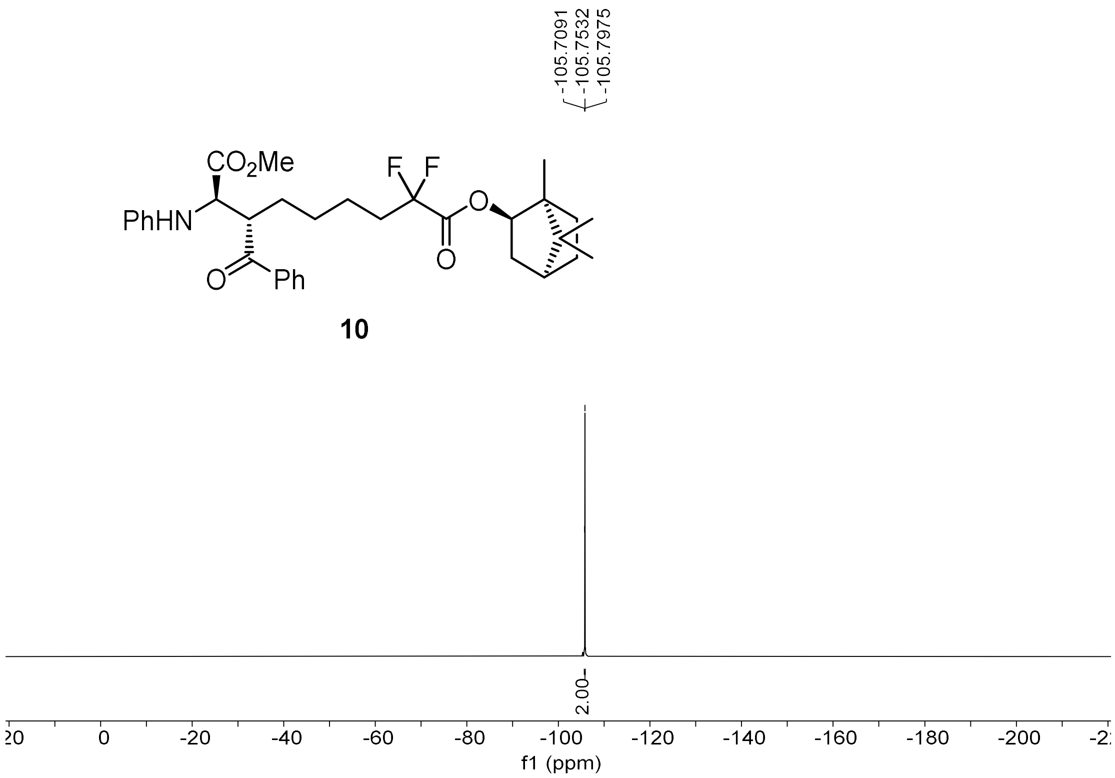

^{19}**F NMR** (377 MHz, $CDCl_3$) of **10**

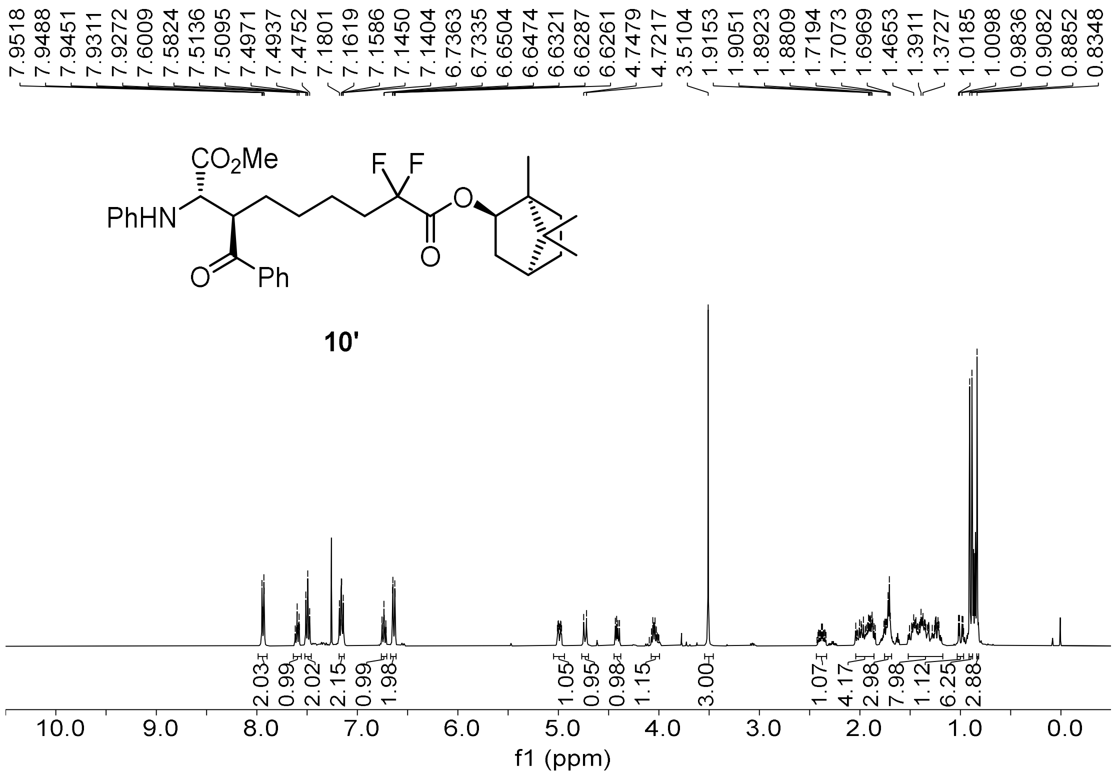

^{1}**H NMR** (400 MHz, $CDCl_3$) of **10’**

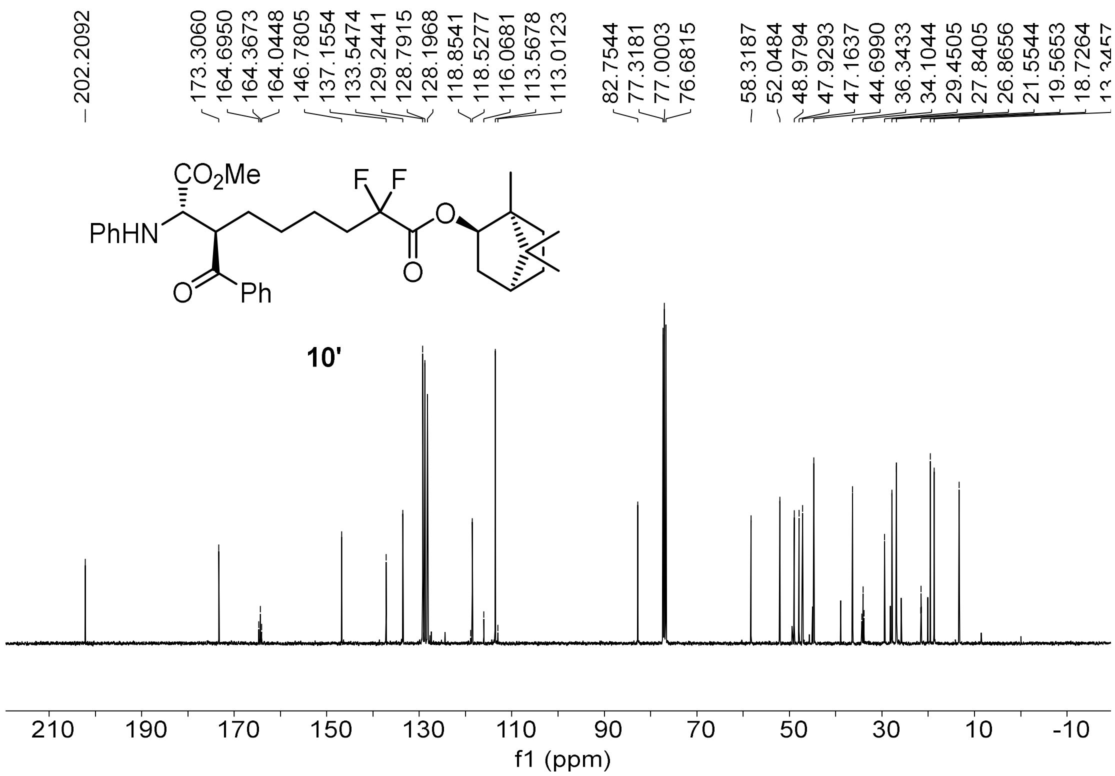

^{13}C NMR (100 MHz, $CDCl_3$) of **10’**

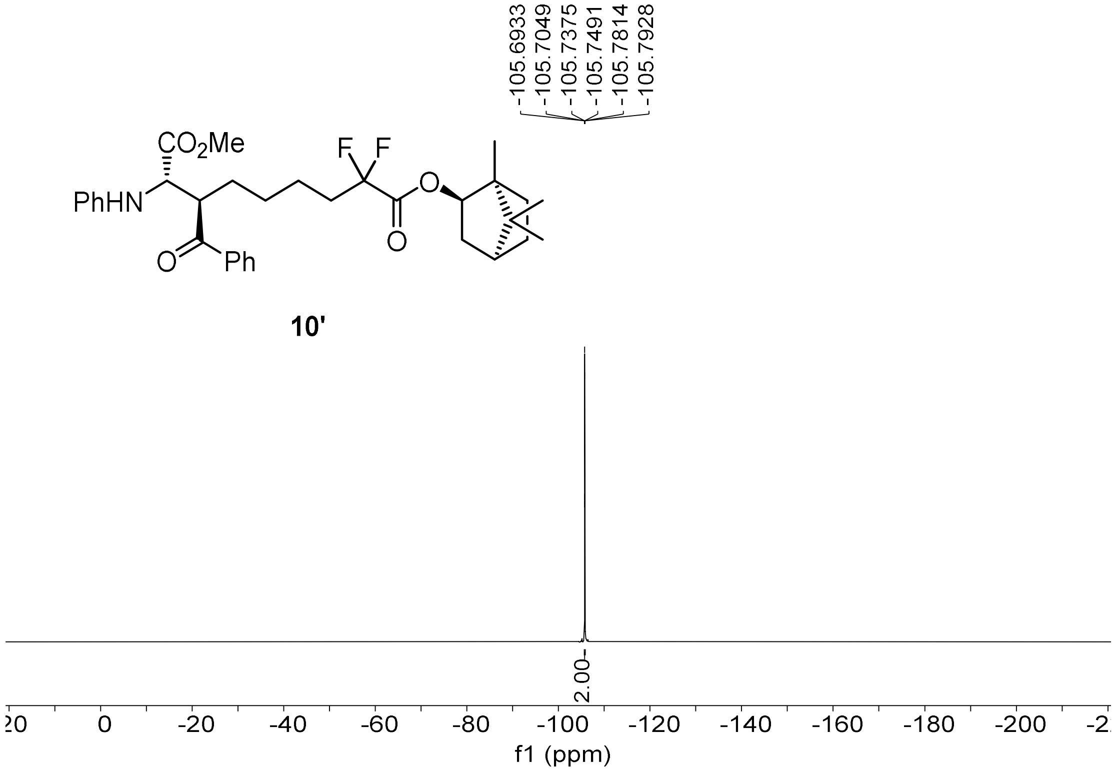

^{19}F NMR (376 MHz, $CDCl_3$) of **10’**

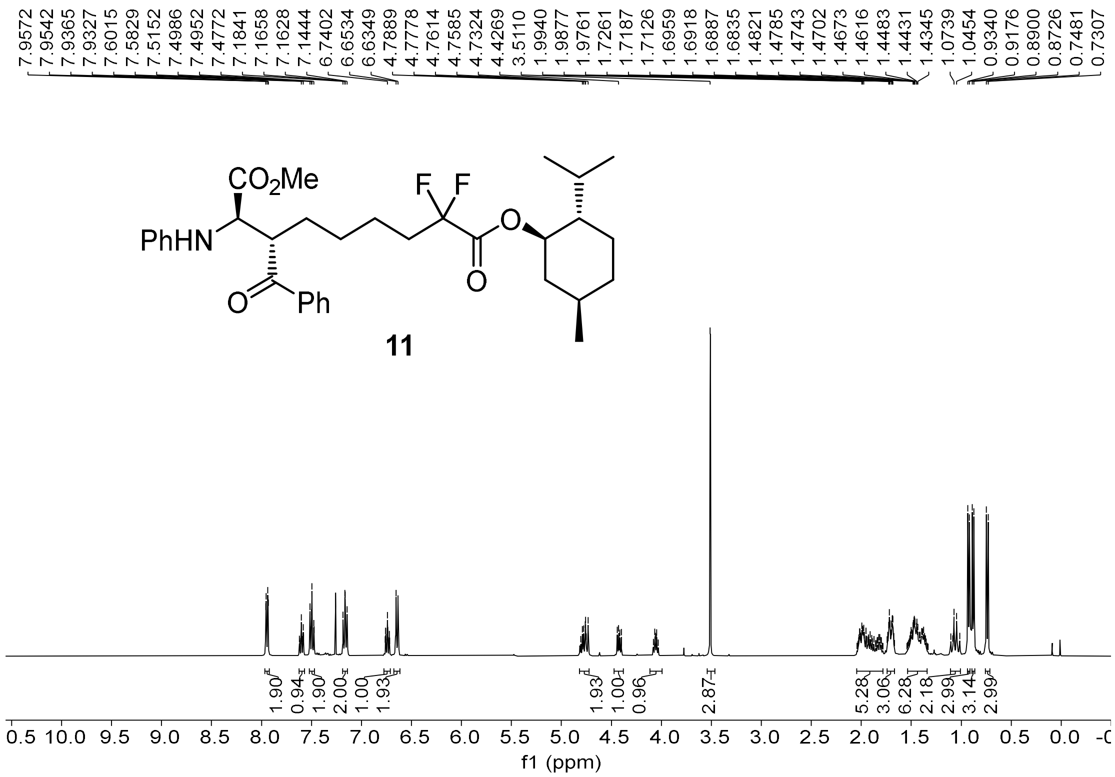

^{1}H NMR (400 MHz, $CDCl_3$) of **11**

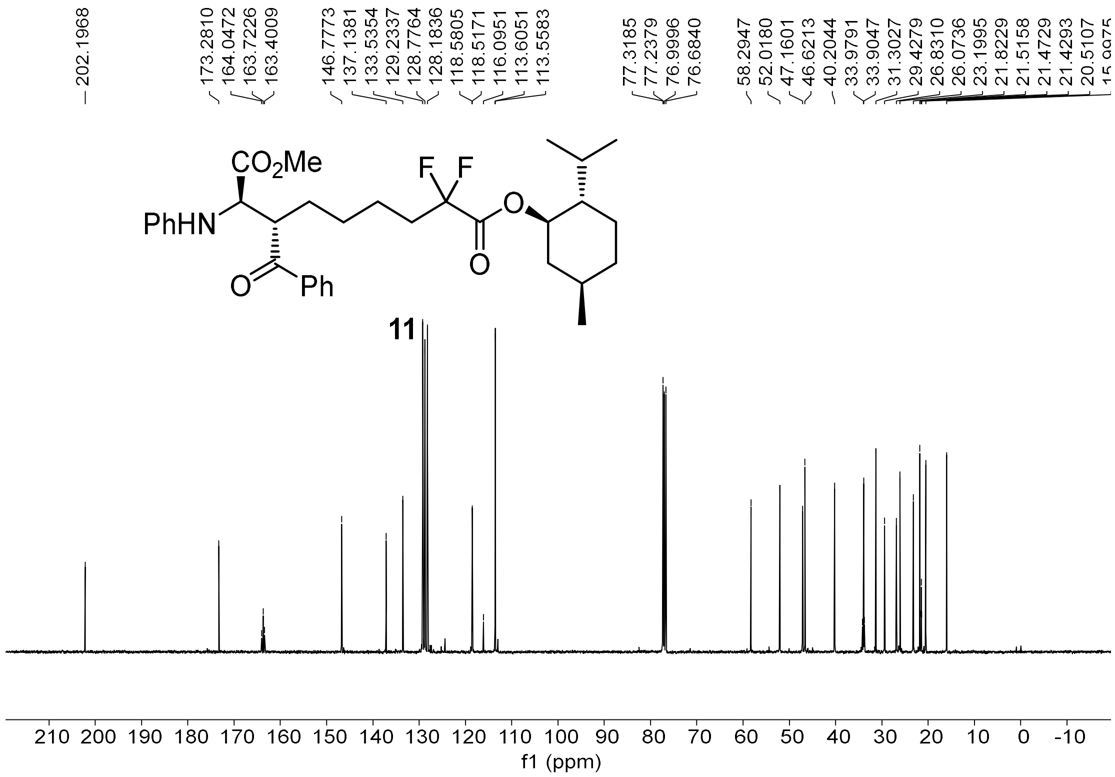

^{13}C NMR (100 MHz, $CDCl_3$) of **11**

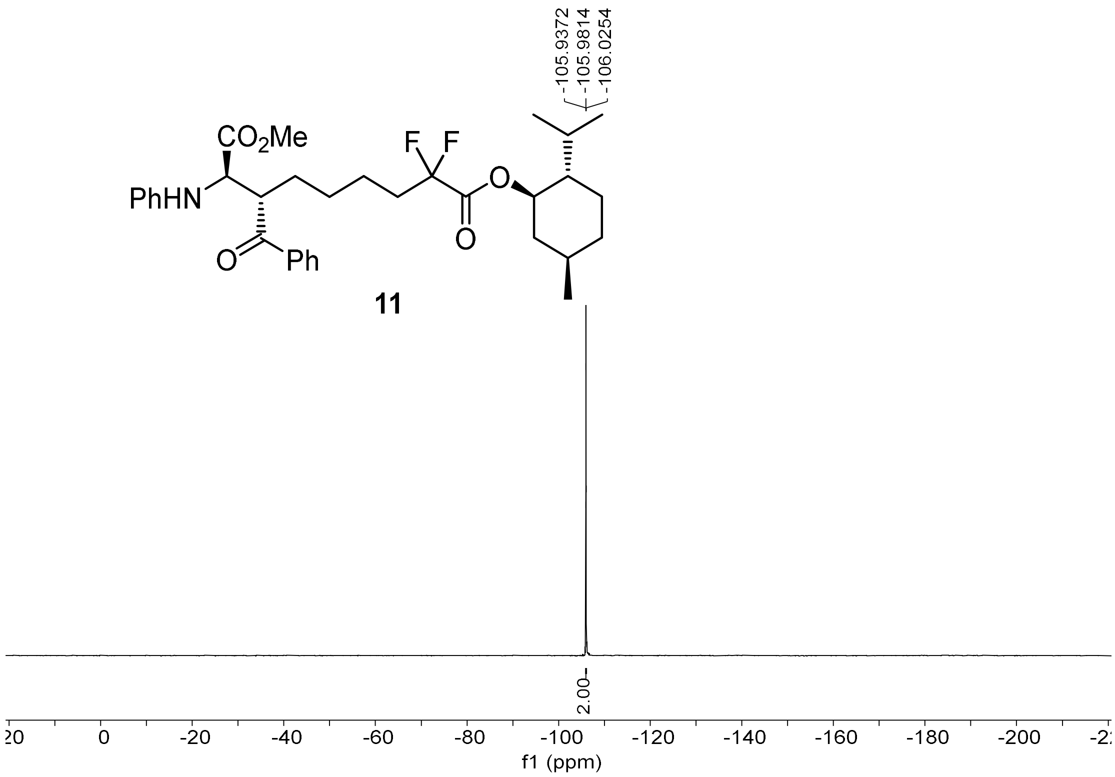

^{19}F NMR (376 MHz, $CDCl_3$) of **11**

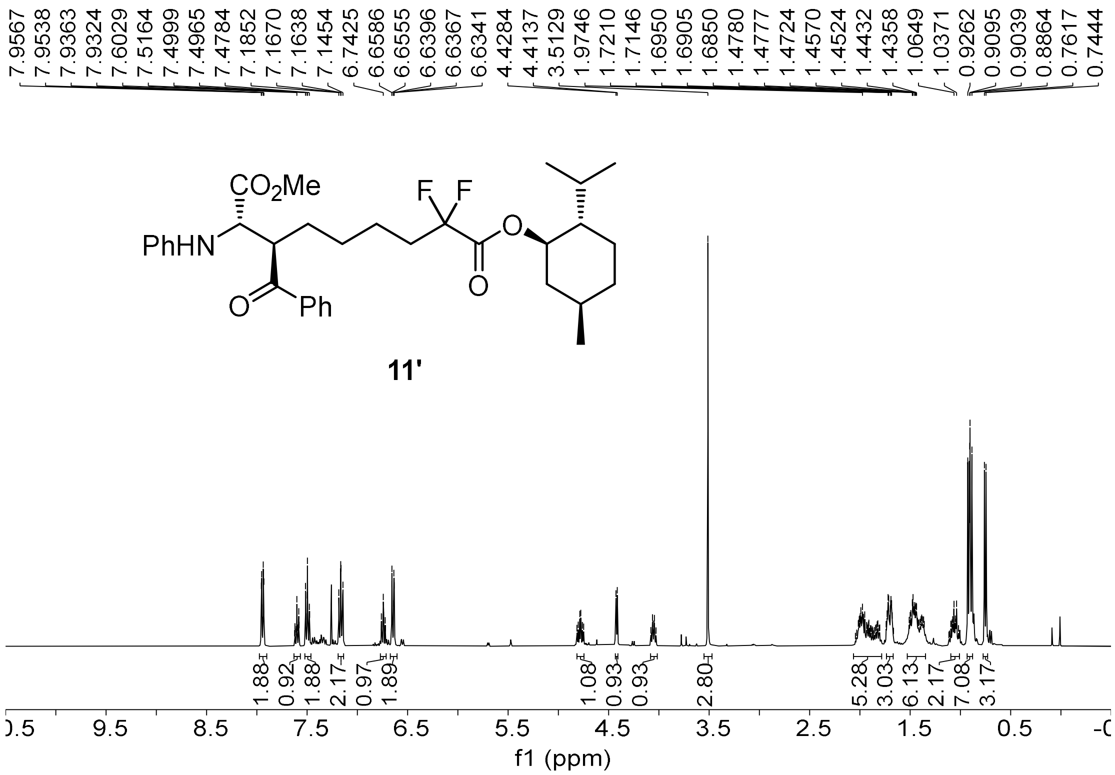

^{1}H NMR (400 MHz, $CDCl_3$) of **11'**

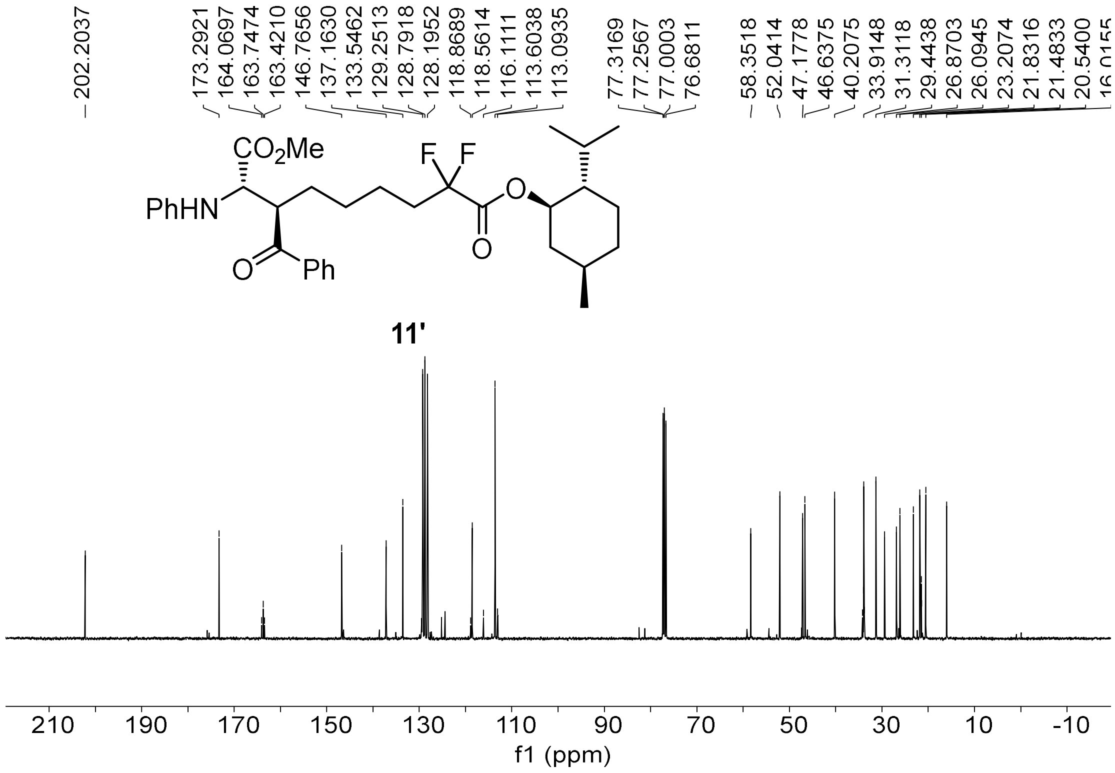

^{13}C NMR (100 MHz, $CDCl_3$) of **11’**

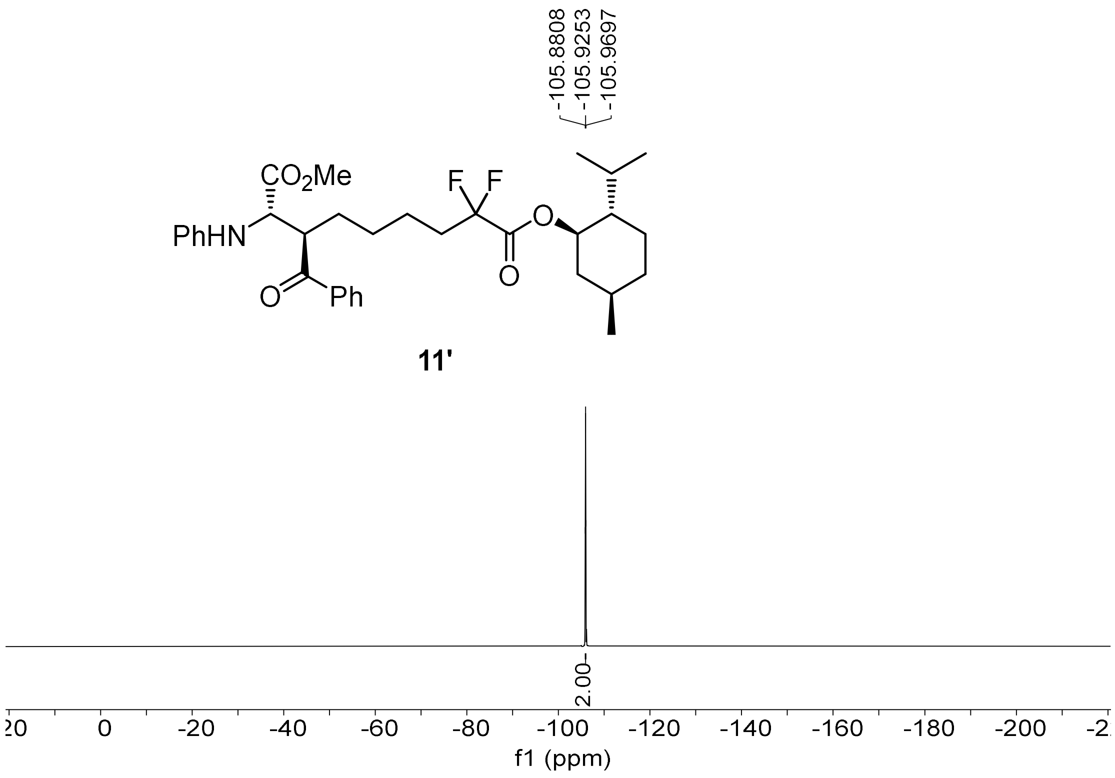

^{19}F NMR (376 MHz, $CDCl_3$) of **11’**

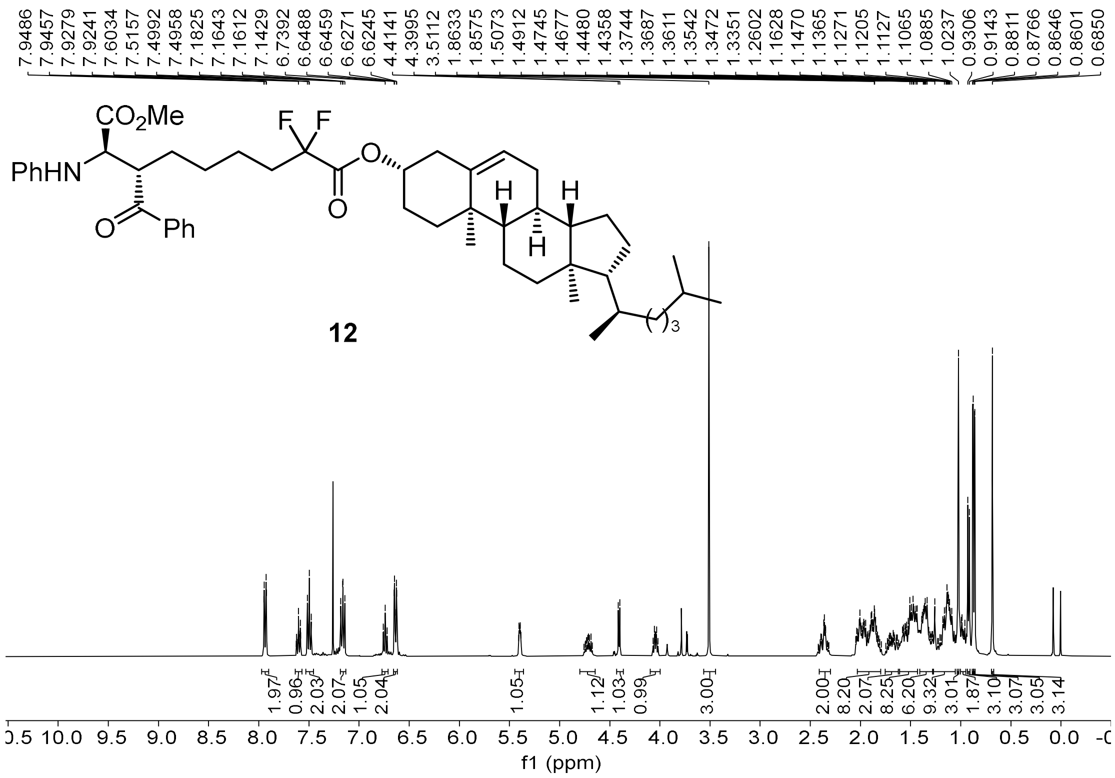

^{1}**H NMR** (400 MHz, $CDCl_3$) of **12**

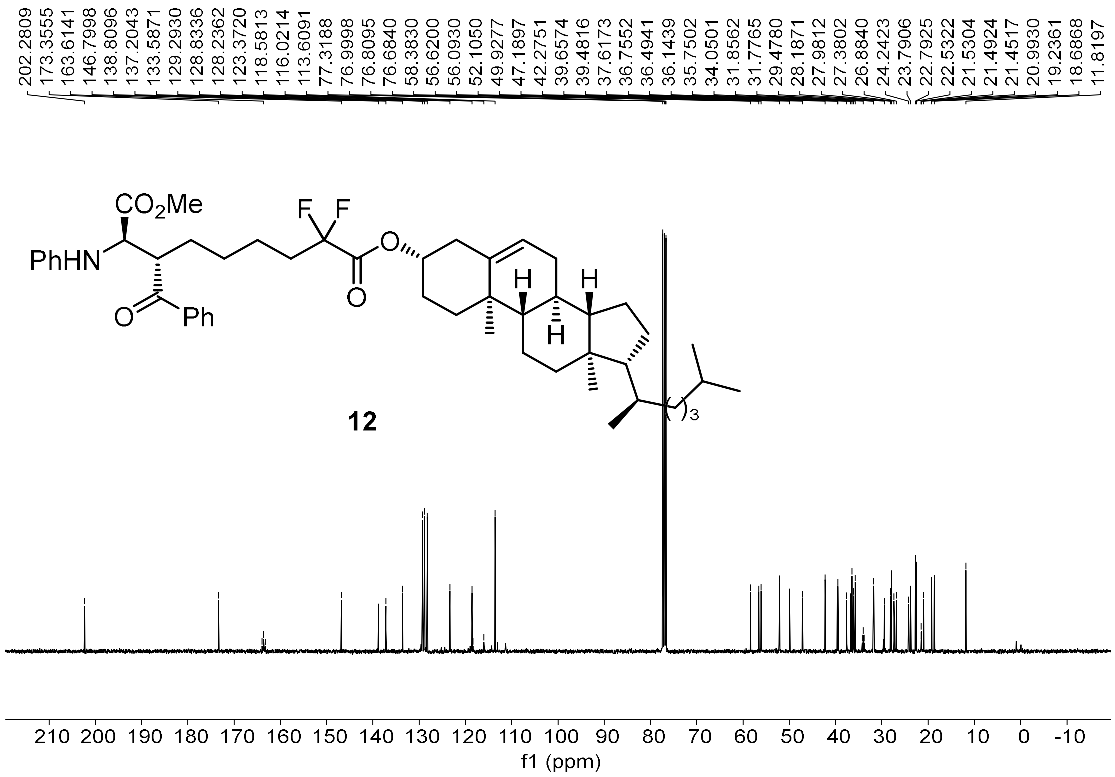

^{13}**C NMR** (100 MHz, $CDCl_3$) of **12**

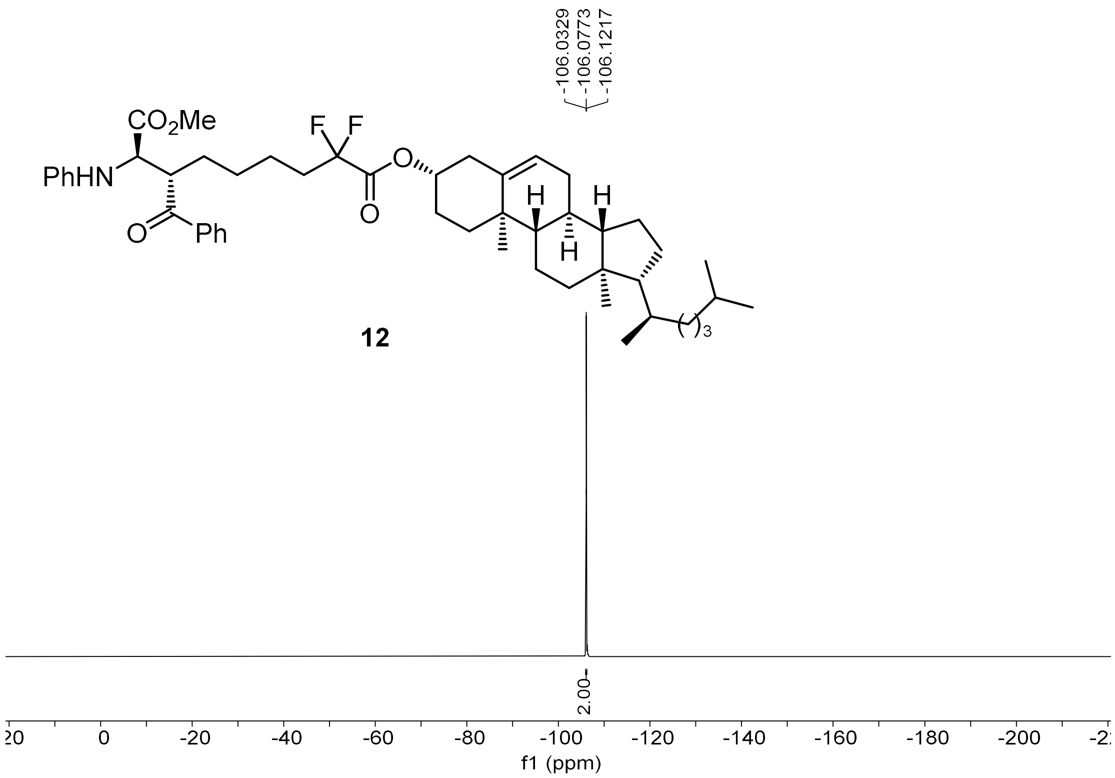

^{19}F NMR (376 MHz, $CDCl_3$) of **12**

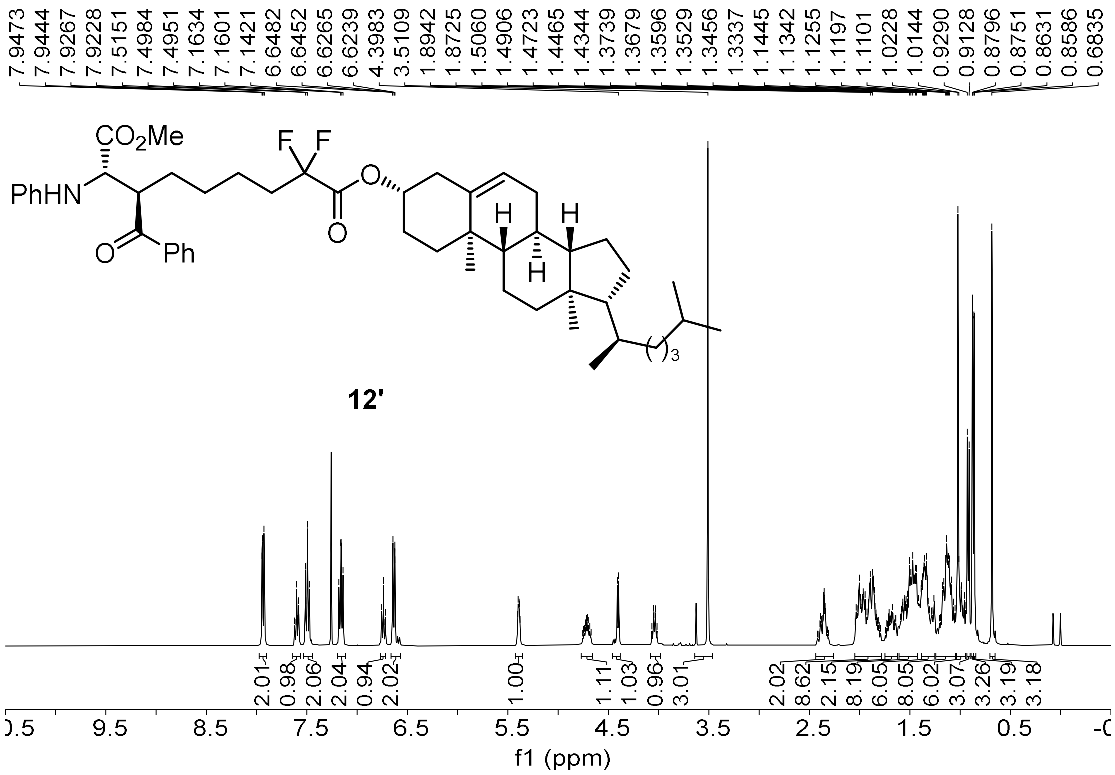

^{1}H NMR (400 MHz, $CDCl_3$) of **12'**

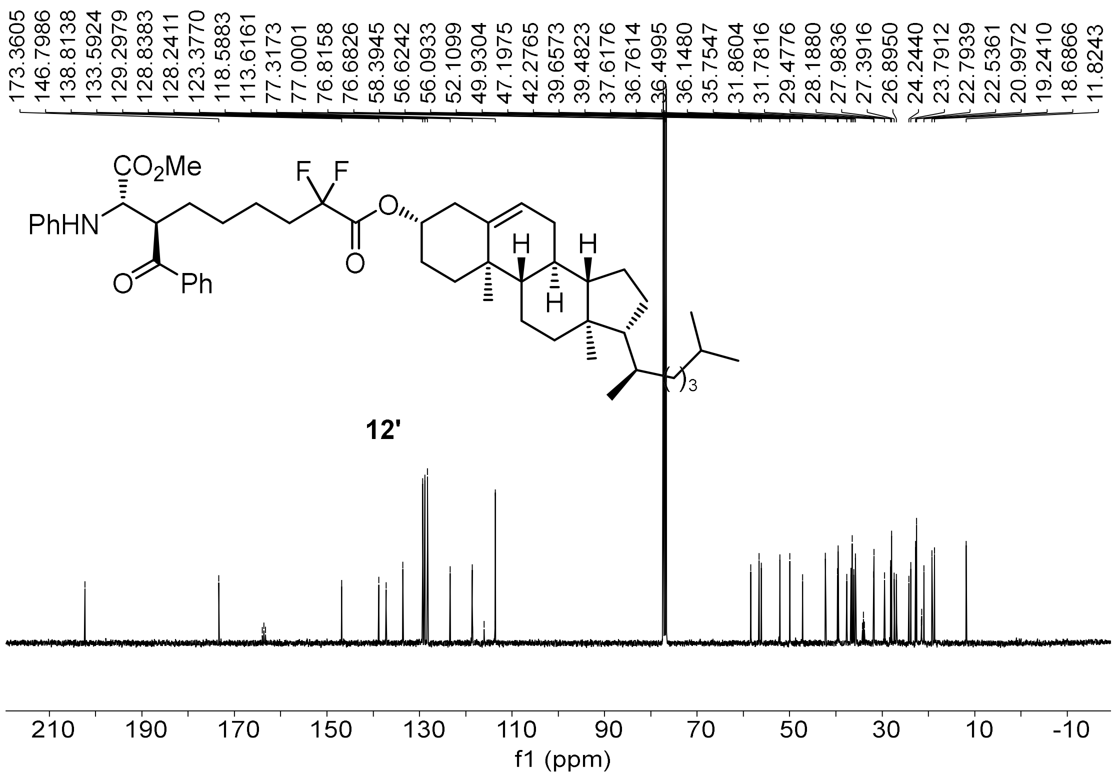

^{13}C NMR (100 MHz, CDCl3) of **12'**

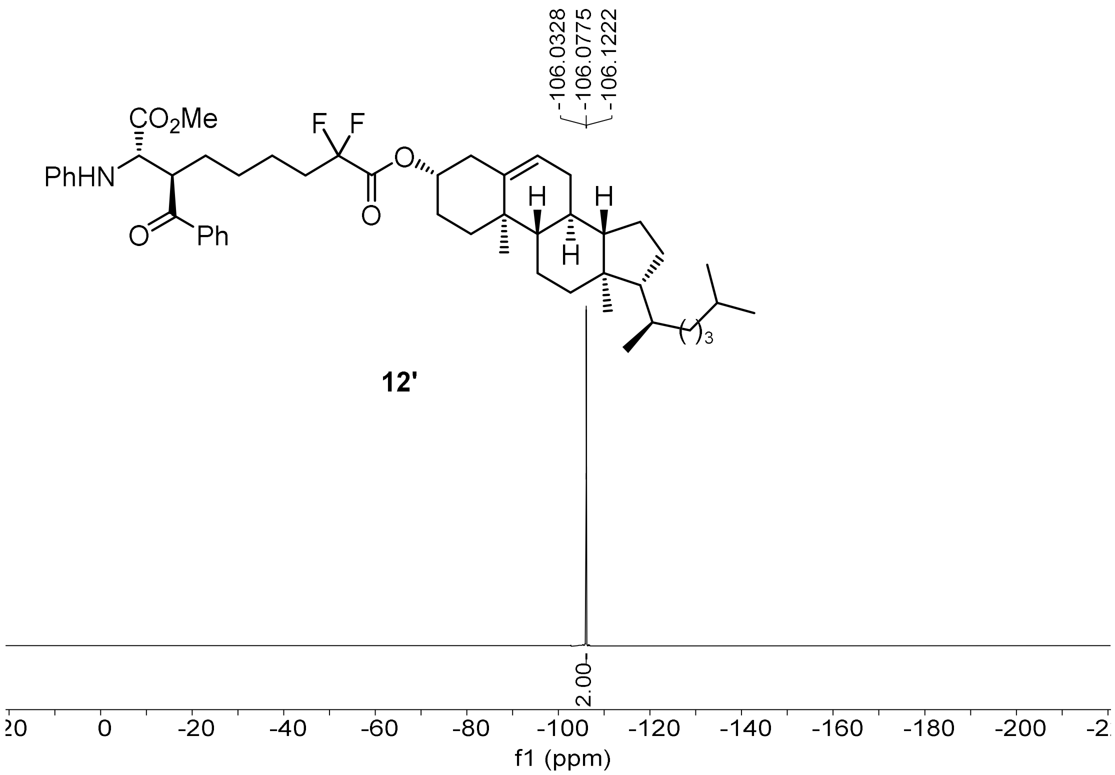

^{19}F NMR (376 MHz, CDCl3) of **12'**

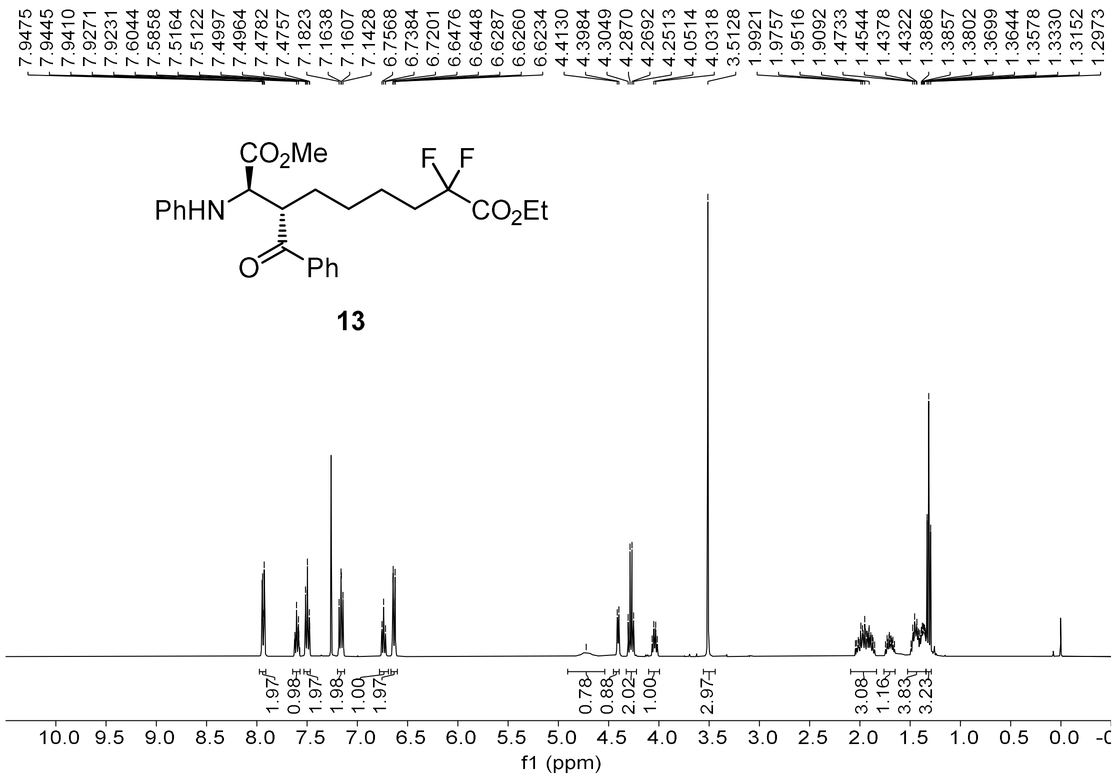

¹H NMR (400 MHz, $CDCl_3$) of **13**

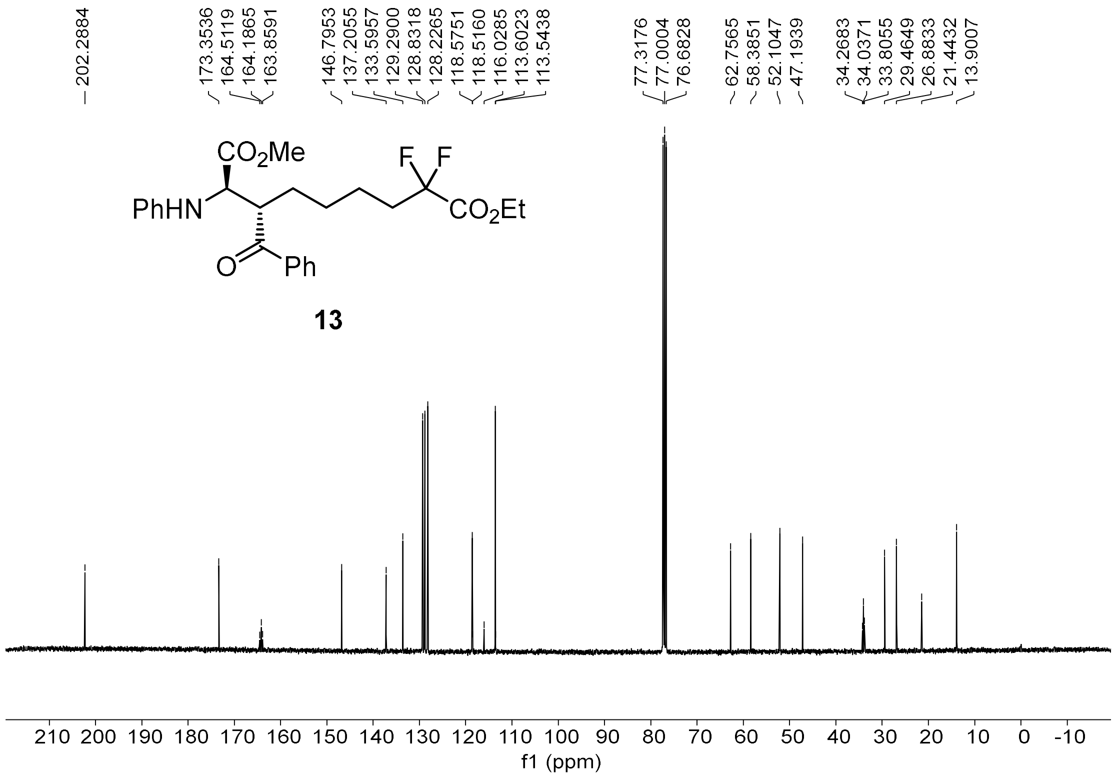

¹³C NMR (100 MHz, $CDCl_3$) of **13**

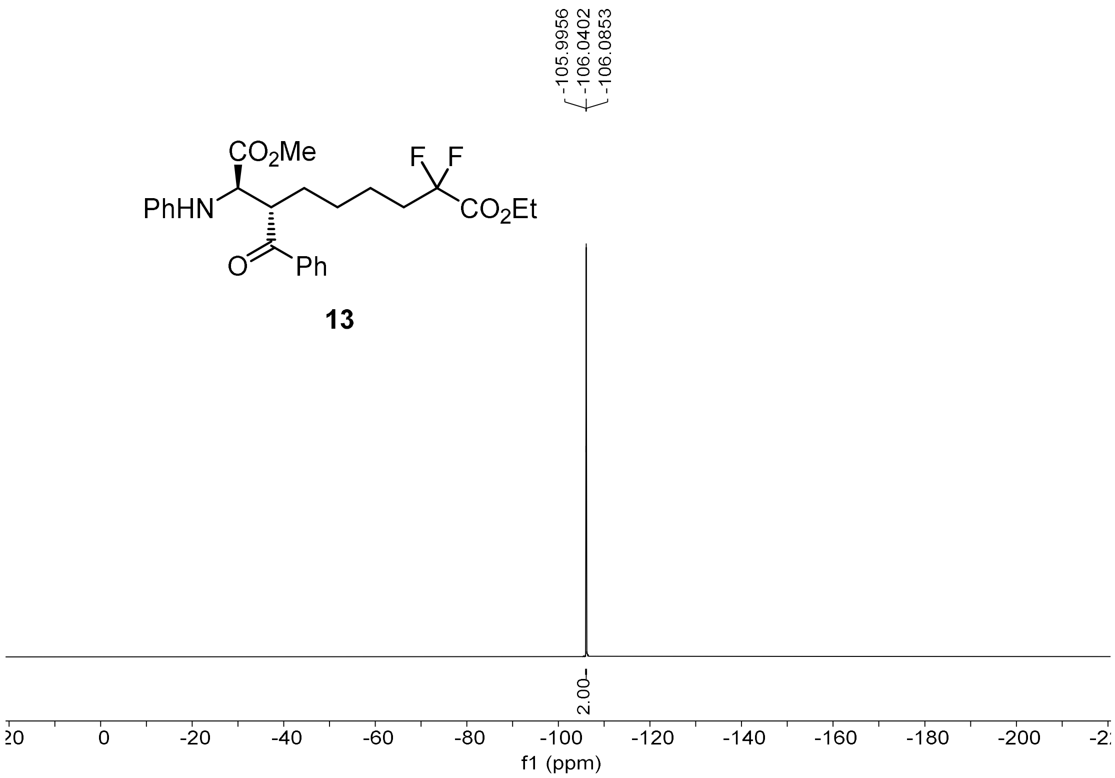

^{19}F NMR (376 MHz, $CDCl_3$) of **13**

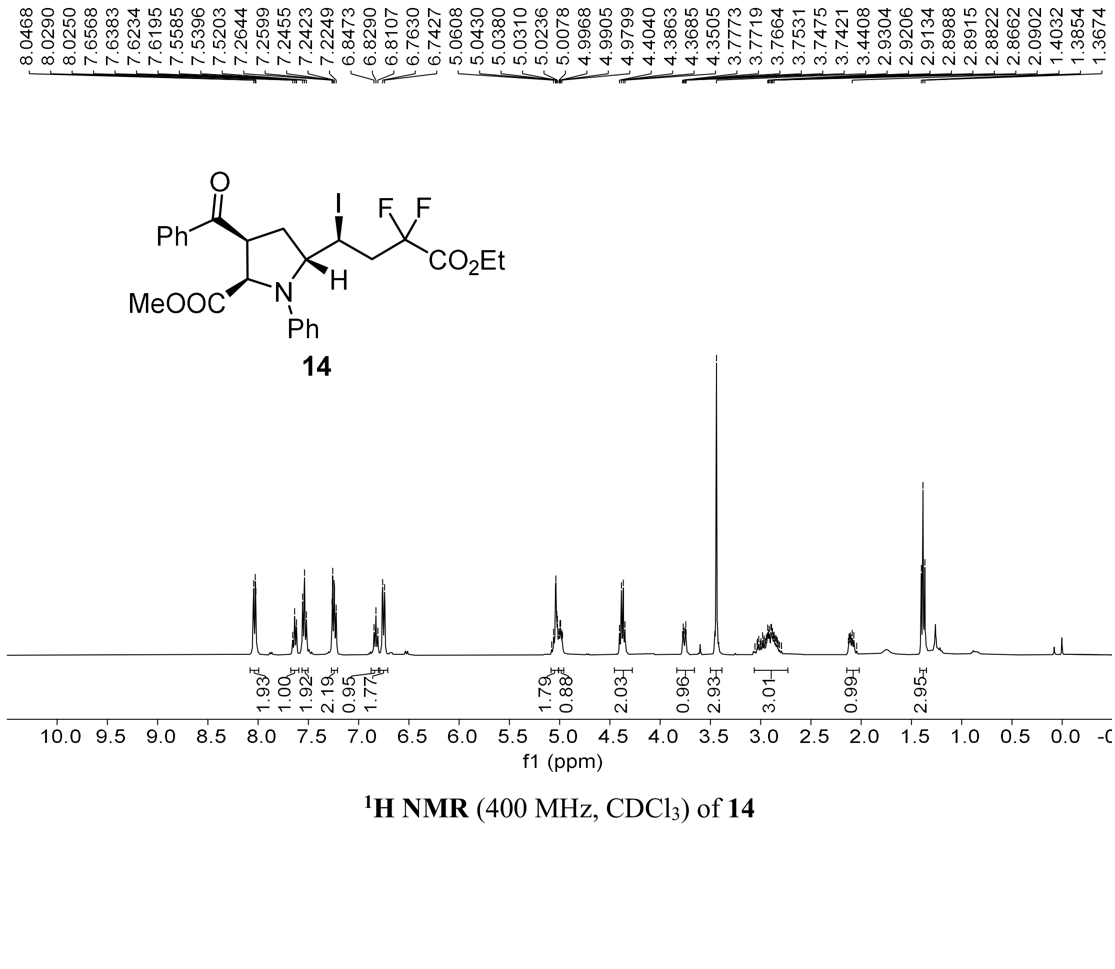

^{1}H NMR (400 MHz, $CDCl_3$) of **14**

HMBC 2D-NMR spectrum of compound **14** in $CDCl_3$

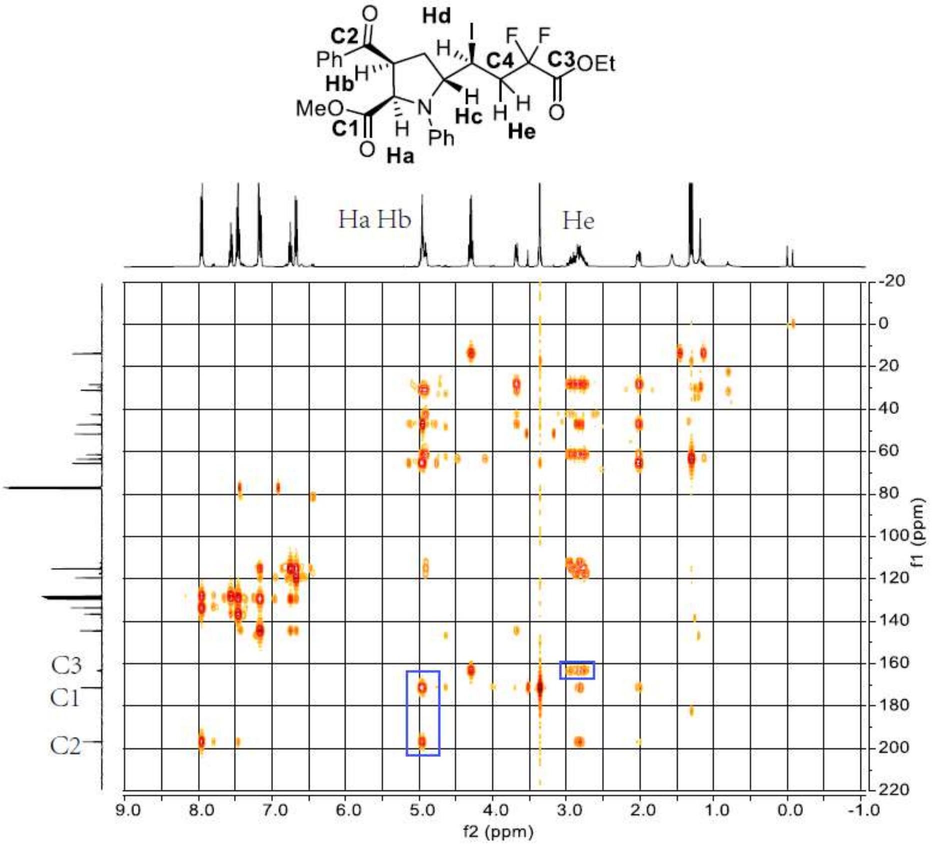

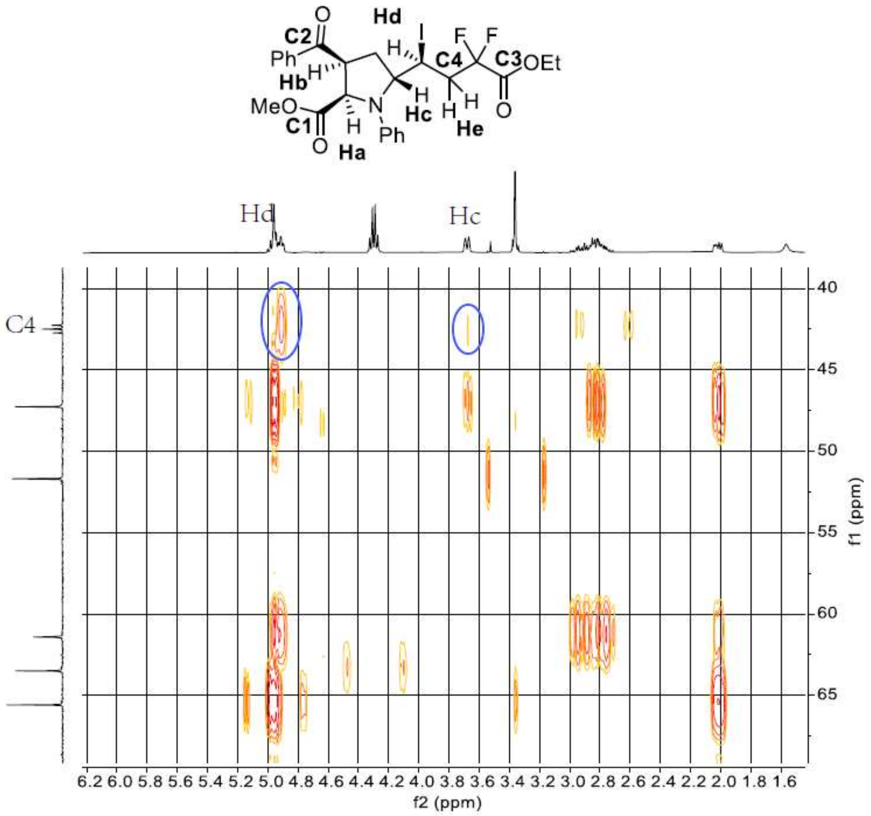

NOE 1D-NMR spectrum of compound **14** in $CDCl_3$ and the determination of the relative stereochemistry

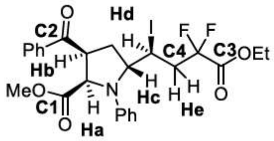

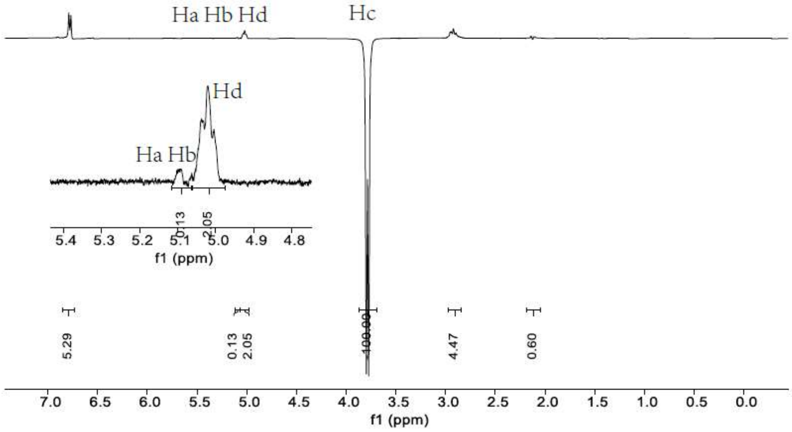

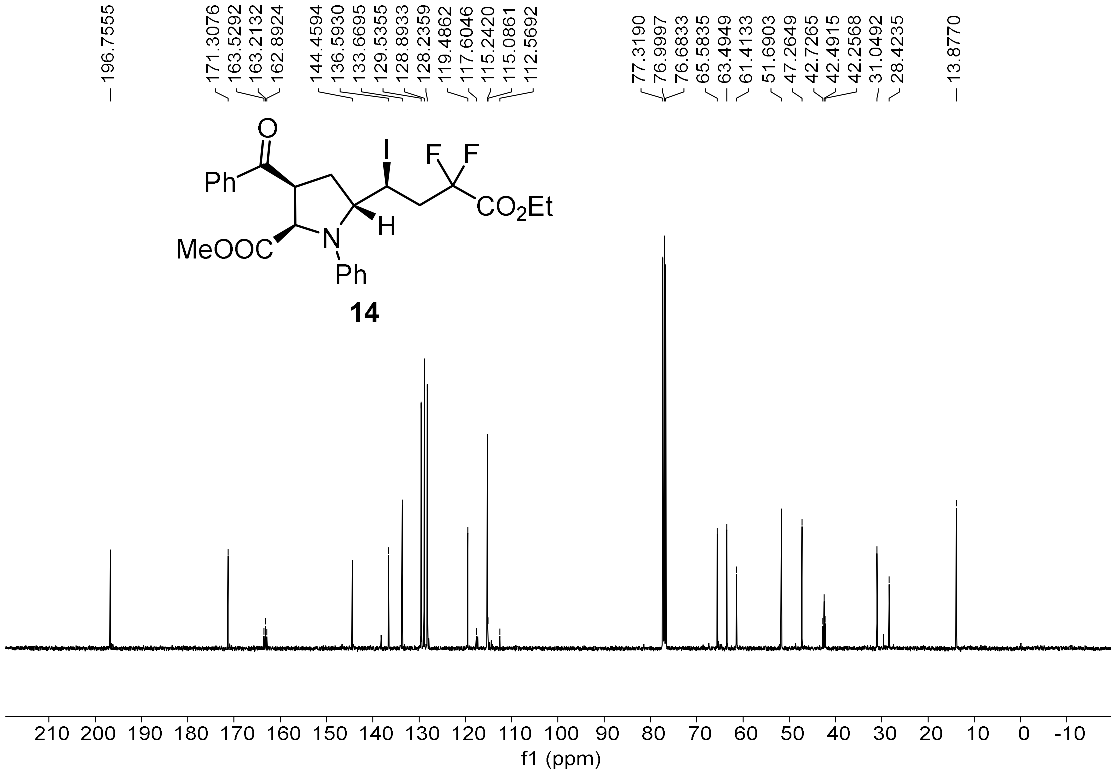

^{13}C NMR (100 MHz, $CDCl_3$) of **14**

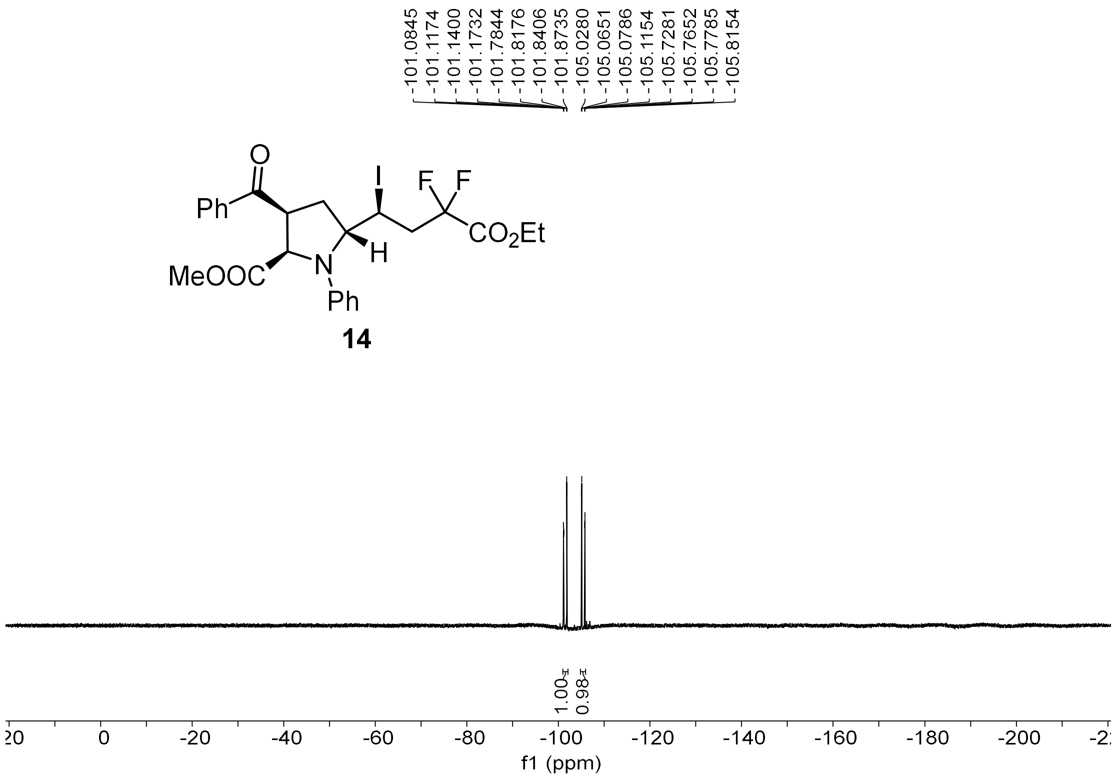

^{19}F NMR (376 MHz, $CDCl_3$) of **14**

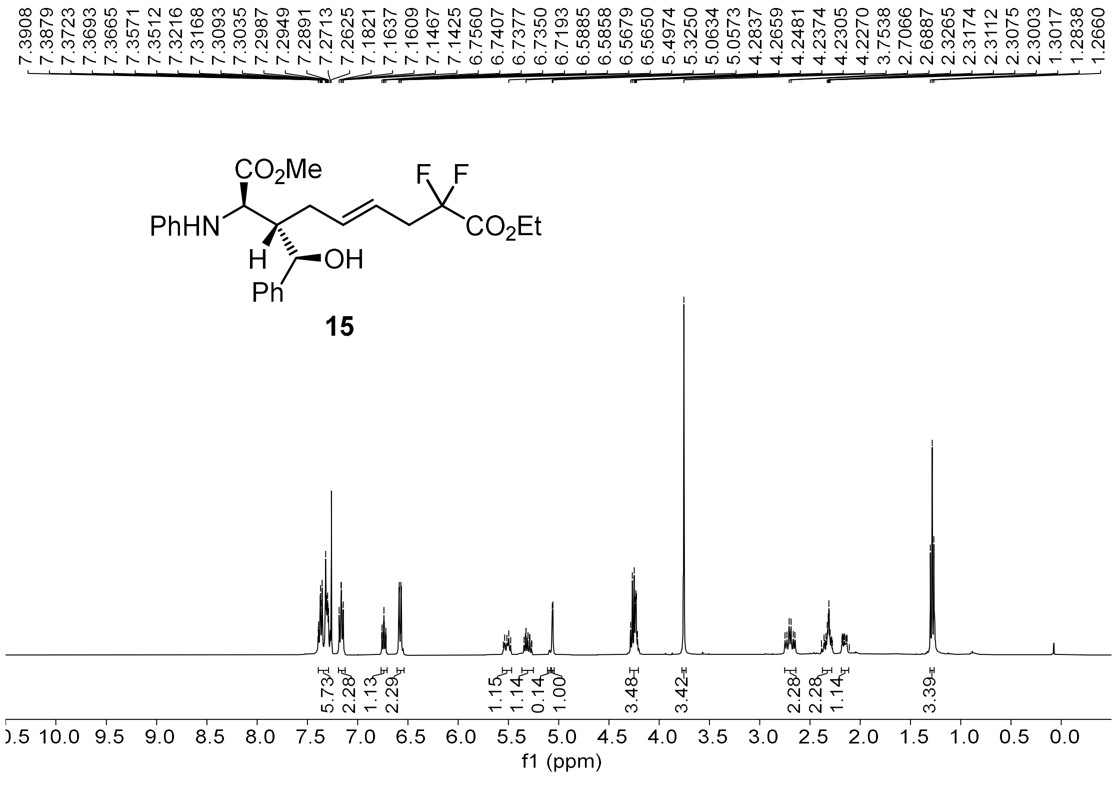

^{1}H NMR (400 MHz, $CDCl_3$) of **15**

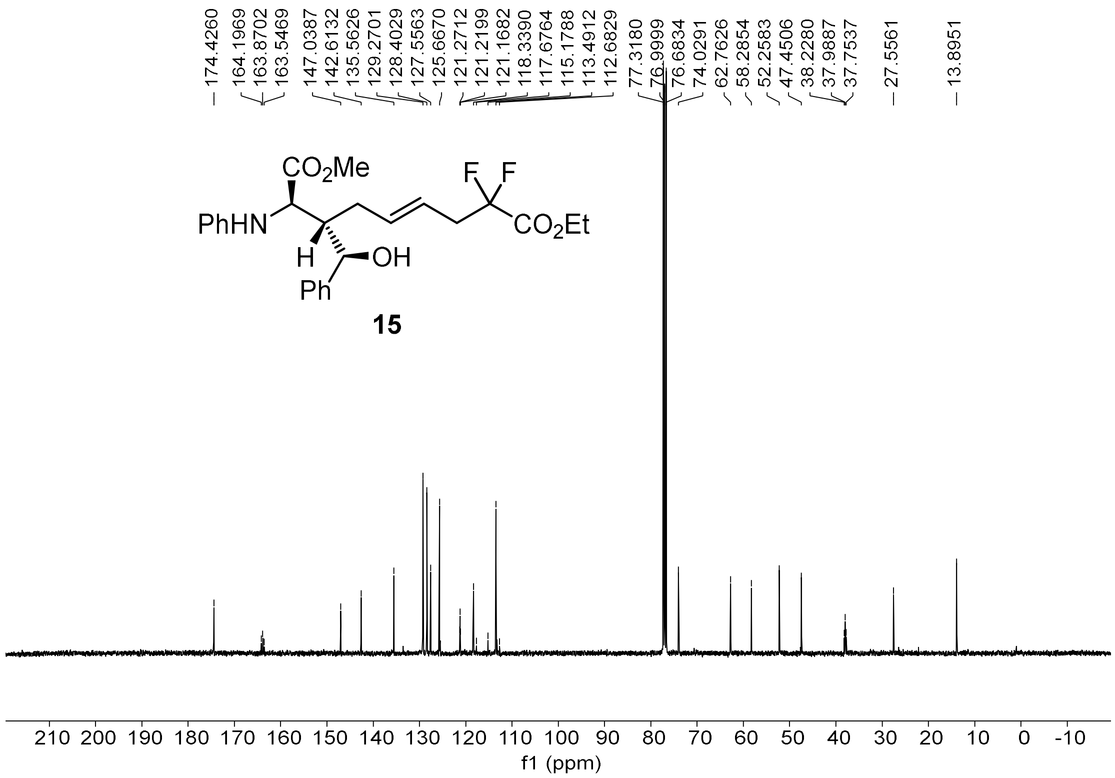

^{13}**C NMR** (100 MHz, $CDCl_3$) of **15**

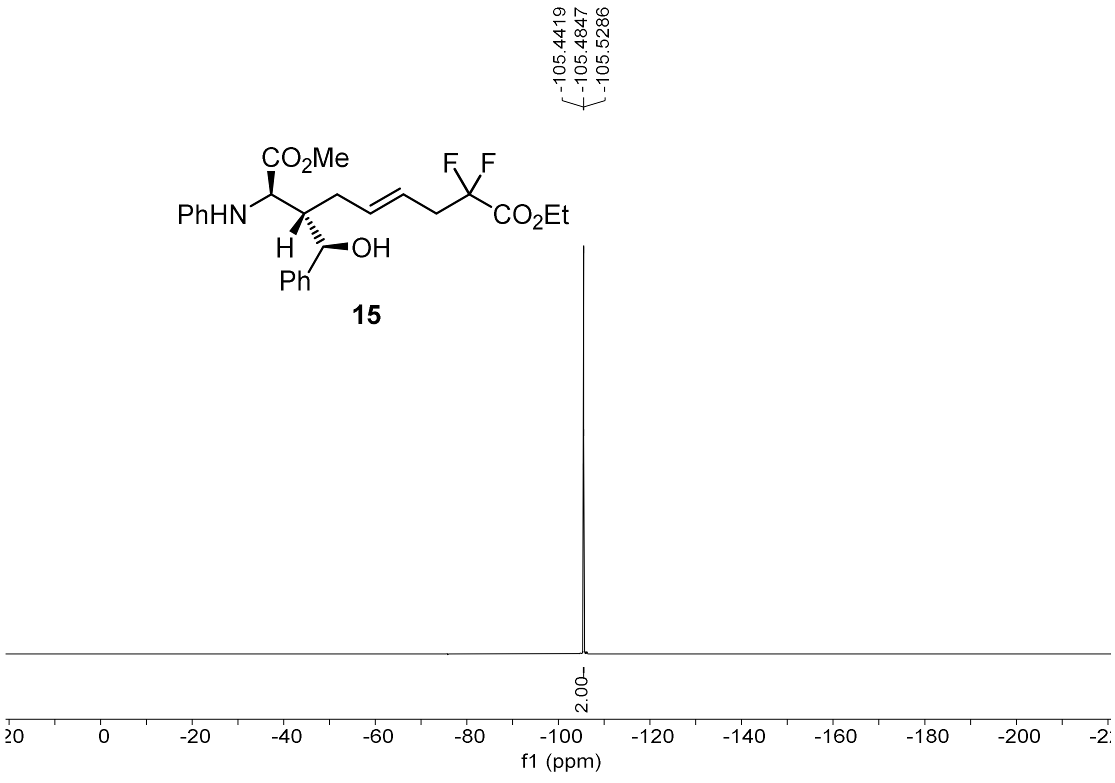

^{19}**F NMR** (376 MHz, $CDCl_3$) of **15**

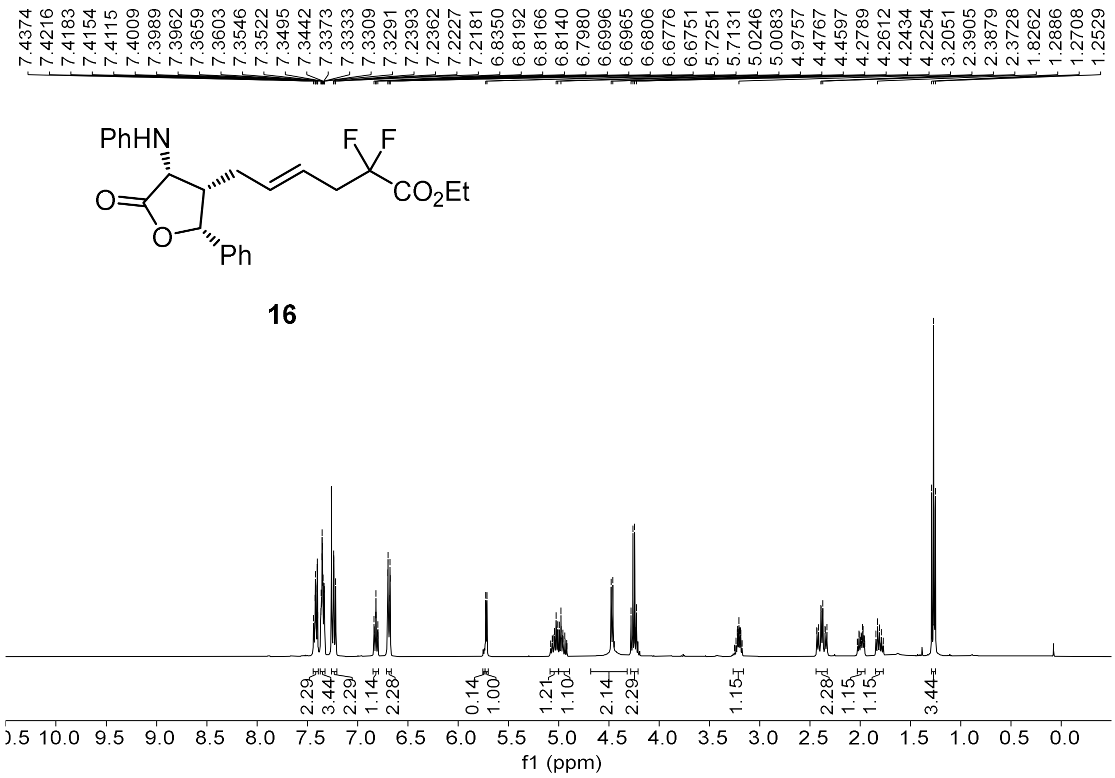

¹H NMR (400 MHz, $CDCl_3$) of **16**

NOE 1D-NMR spectrum of compound **16** in $CDCl_3$ and the determination of the relative stereochemistry

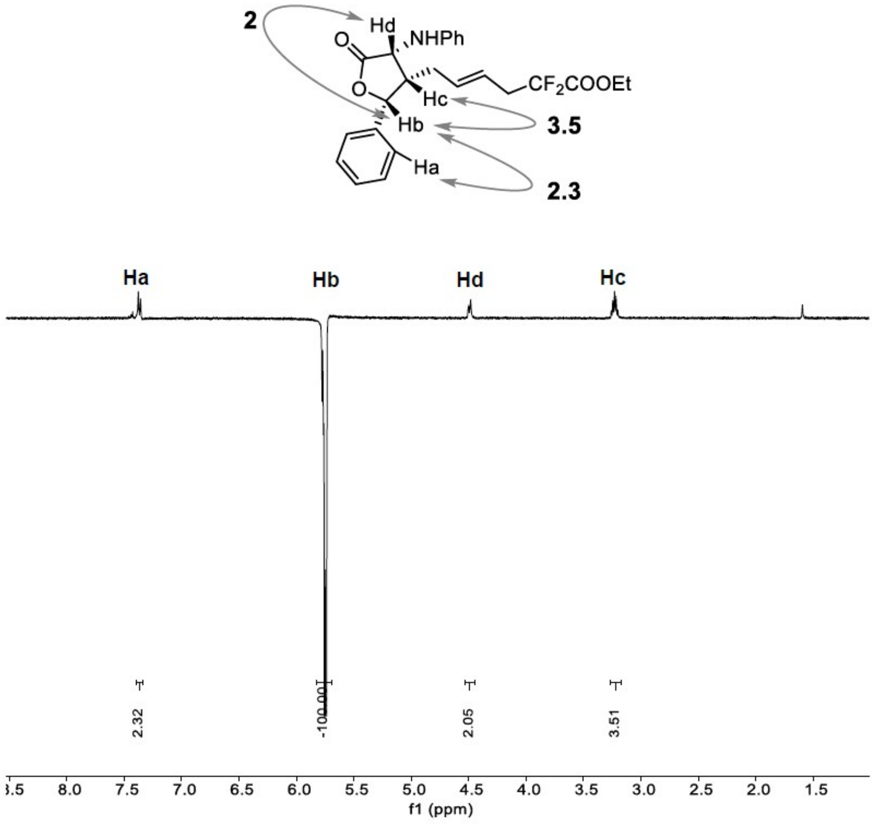

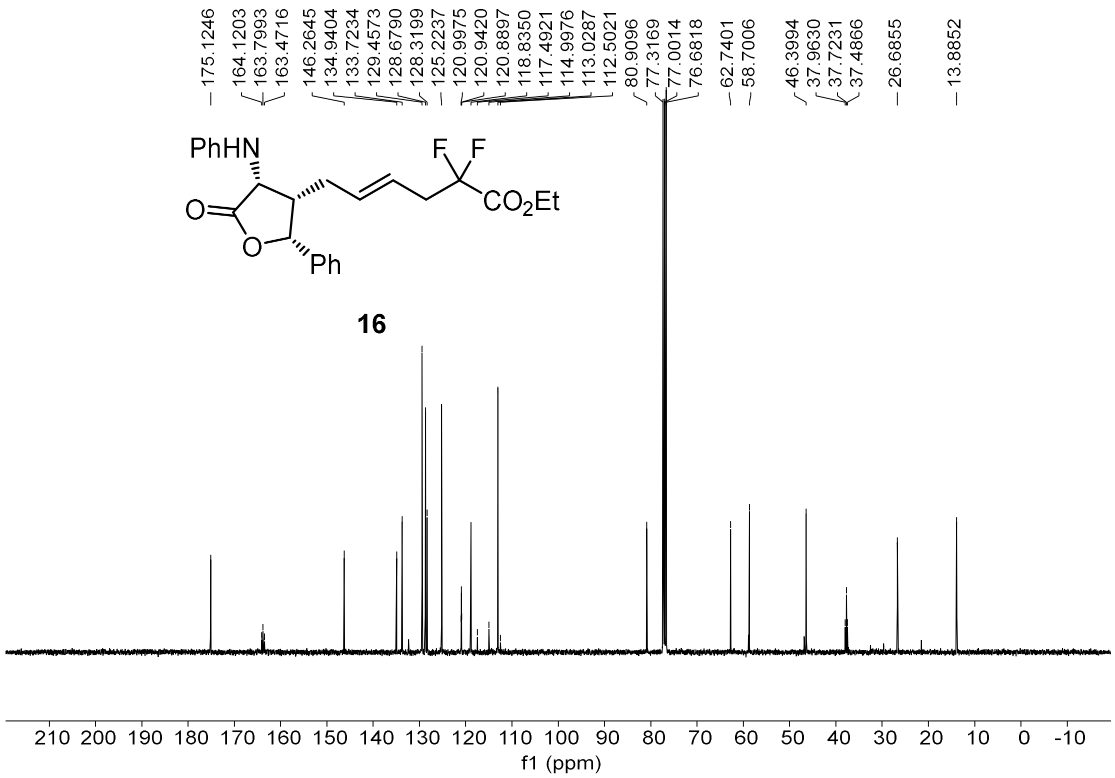

^{13}C NMR (100 MHz, $CDCl_3$) of **16**

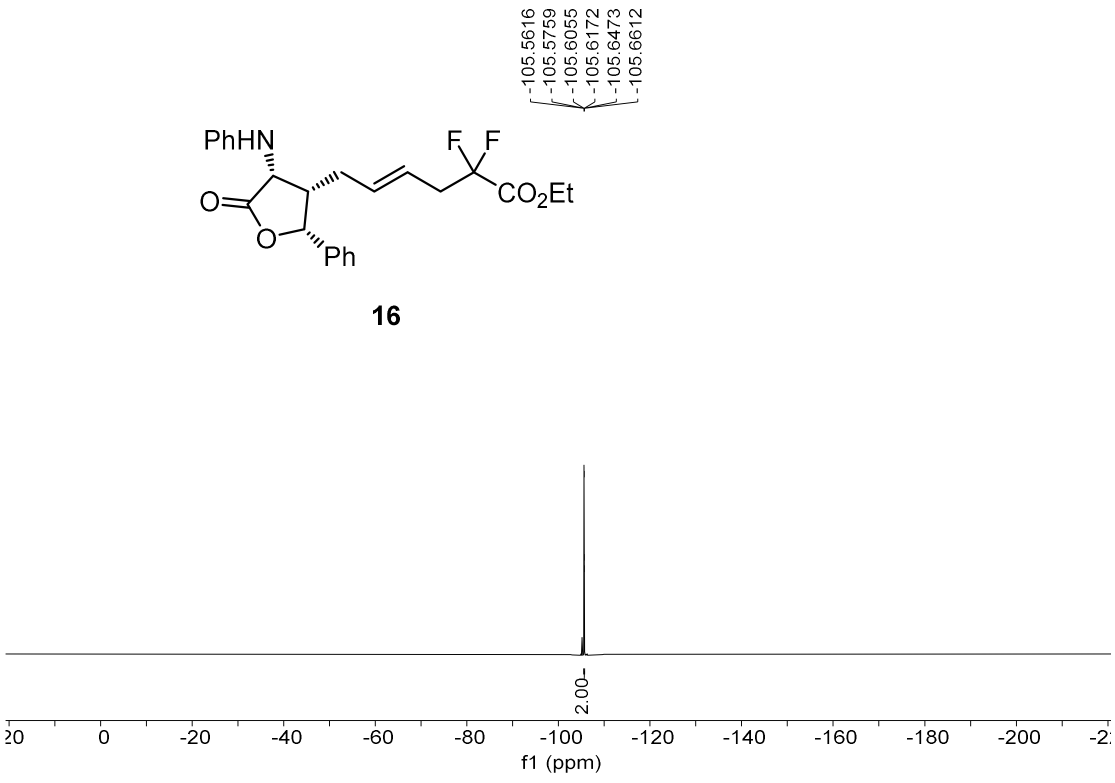

^{19}F NMR (376 MHz, $CDCl_3$) of **16**

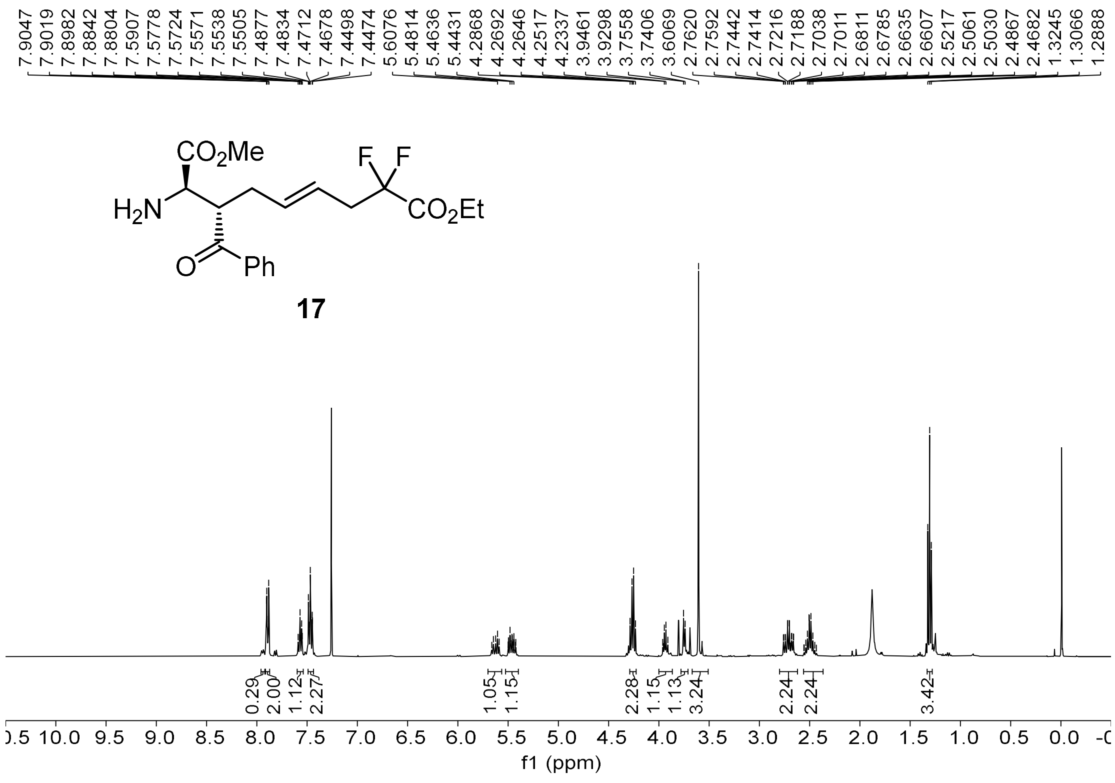

^{1}**H NMR** (400 MHz, $CDCl_3$) of **17**

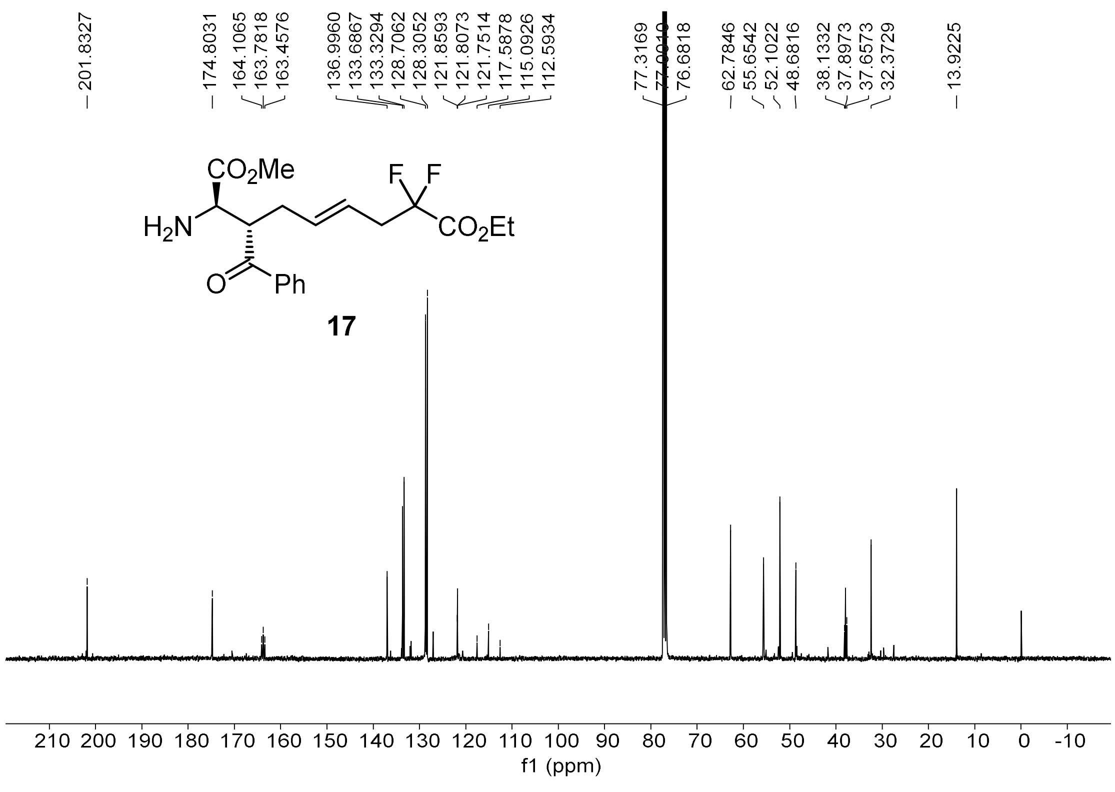

^{13}**C NMR** (100 MHz, $CDCl_3$) of **17**

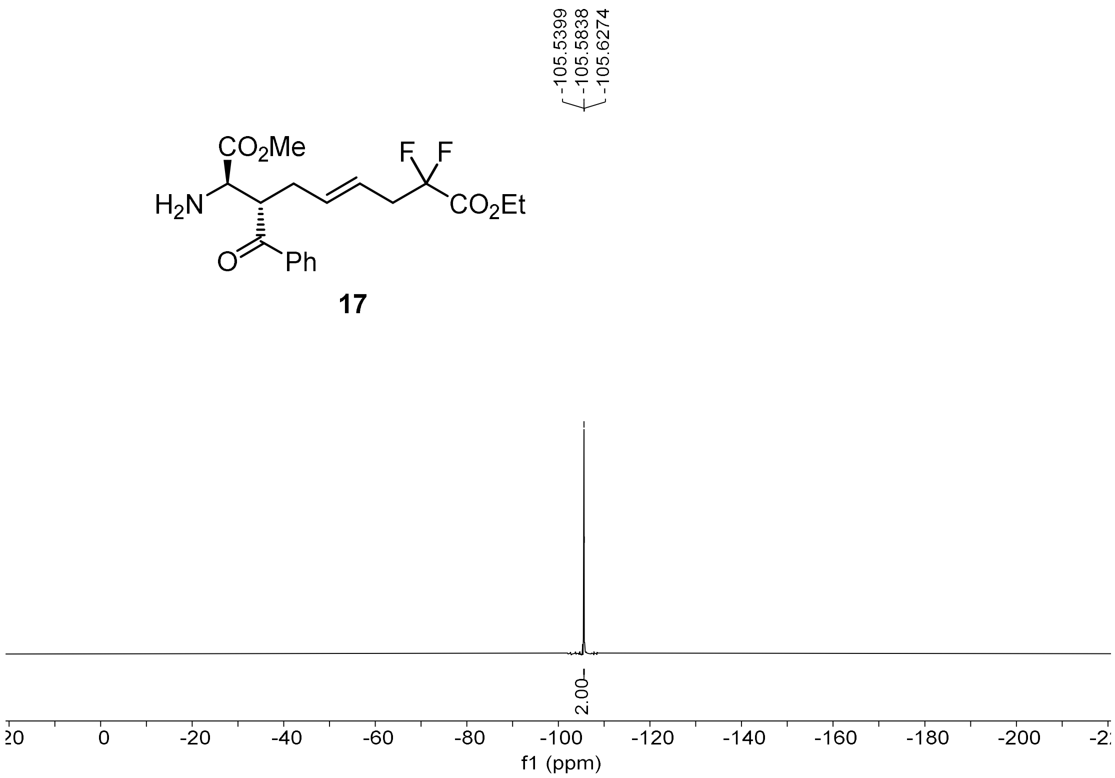

^{19}F NMR (376 MHz, $CDCl_3$) of **17**

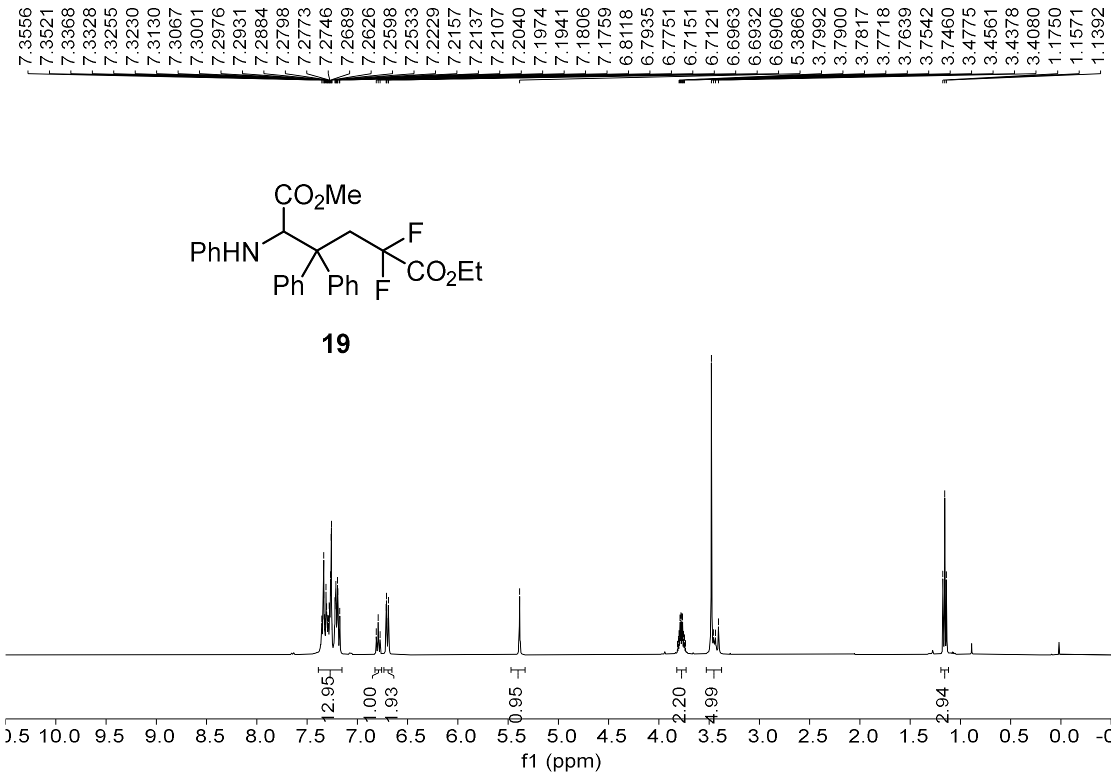

^{1}H NMR (400 MHz, $CDCl_3$) of **19**

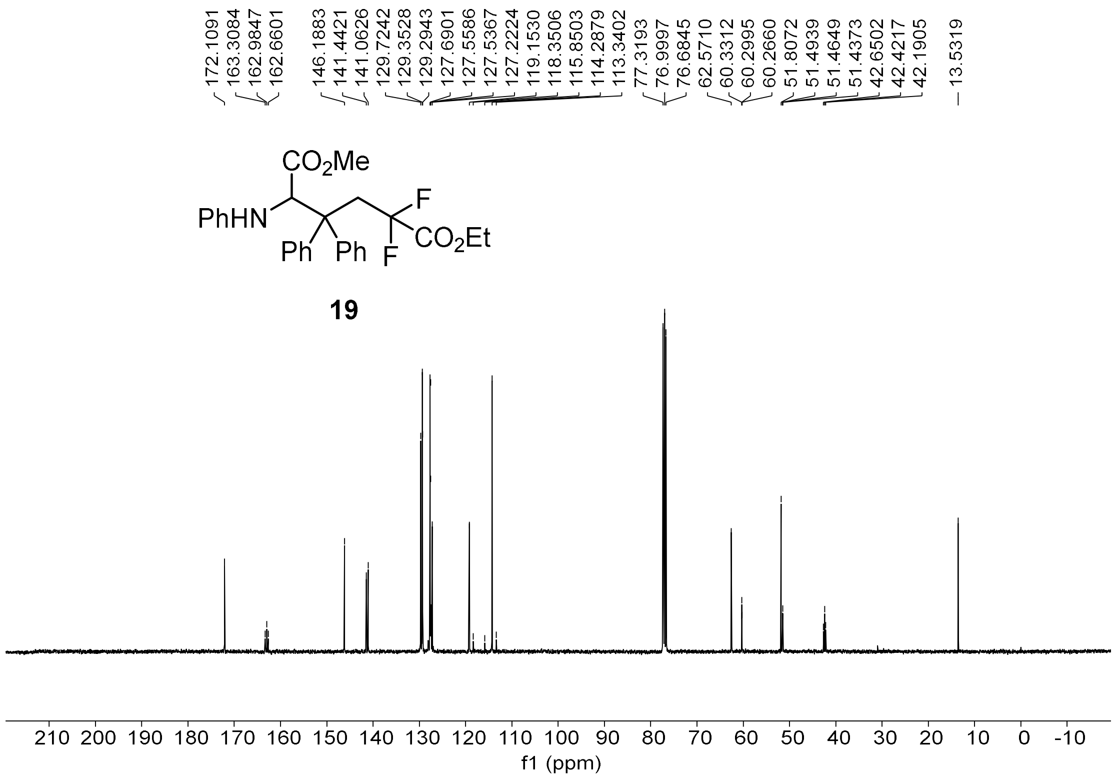

^{13}**C NMR** (100 MHz, $CDCl_3$) of **19**

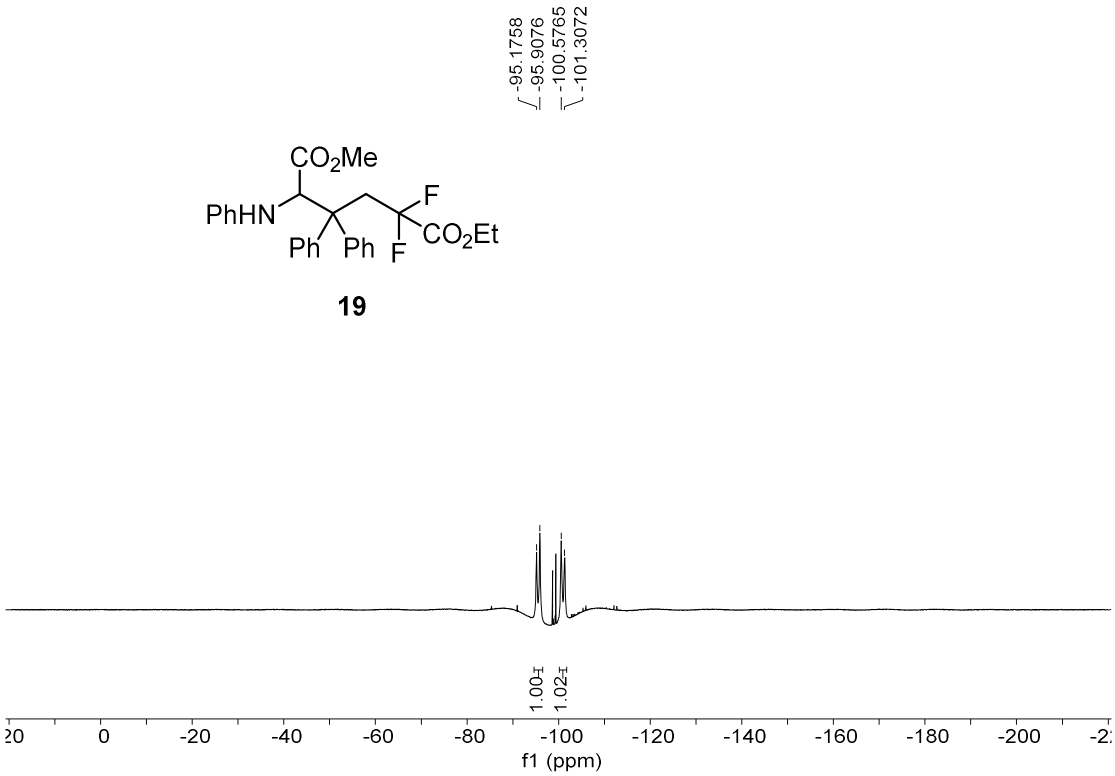

^{19}**F NMR** (376 MHz, $CDCl_3$) of **19**

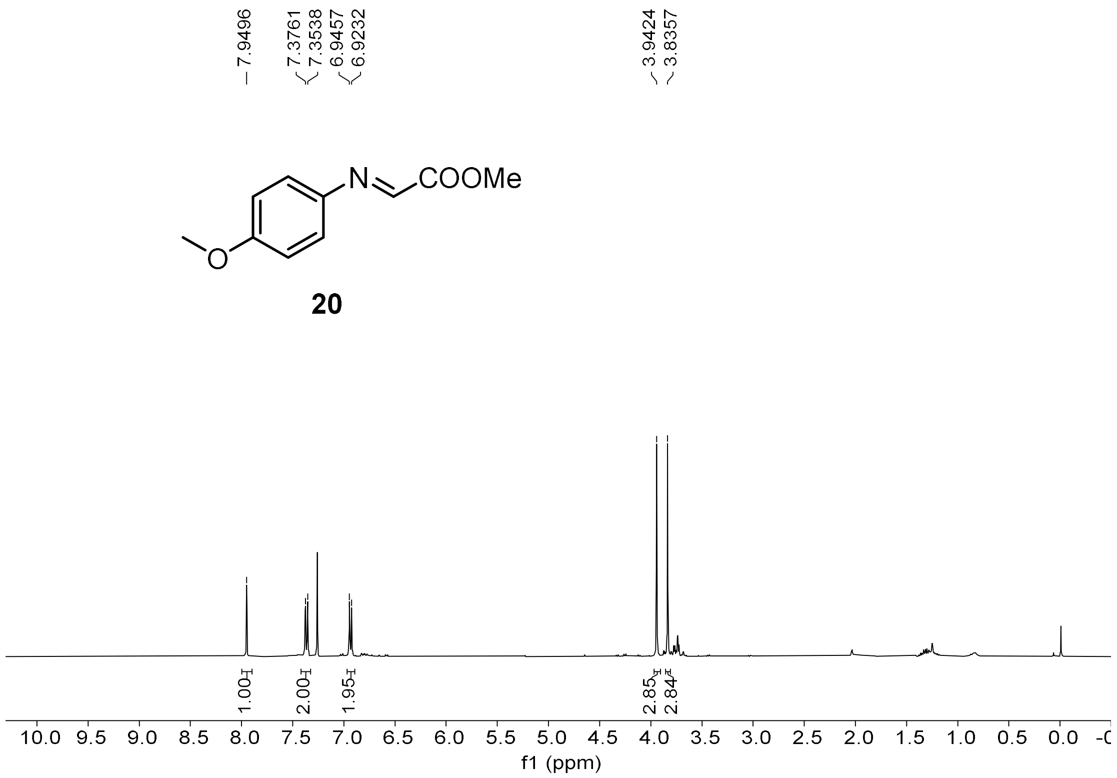

^{1}H NMR (400 MHz, $CDCl_3$) of **20**

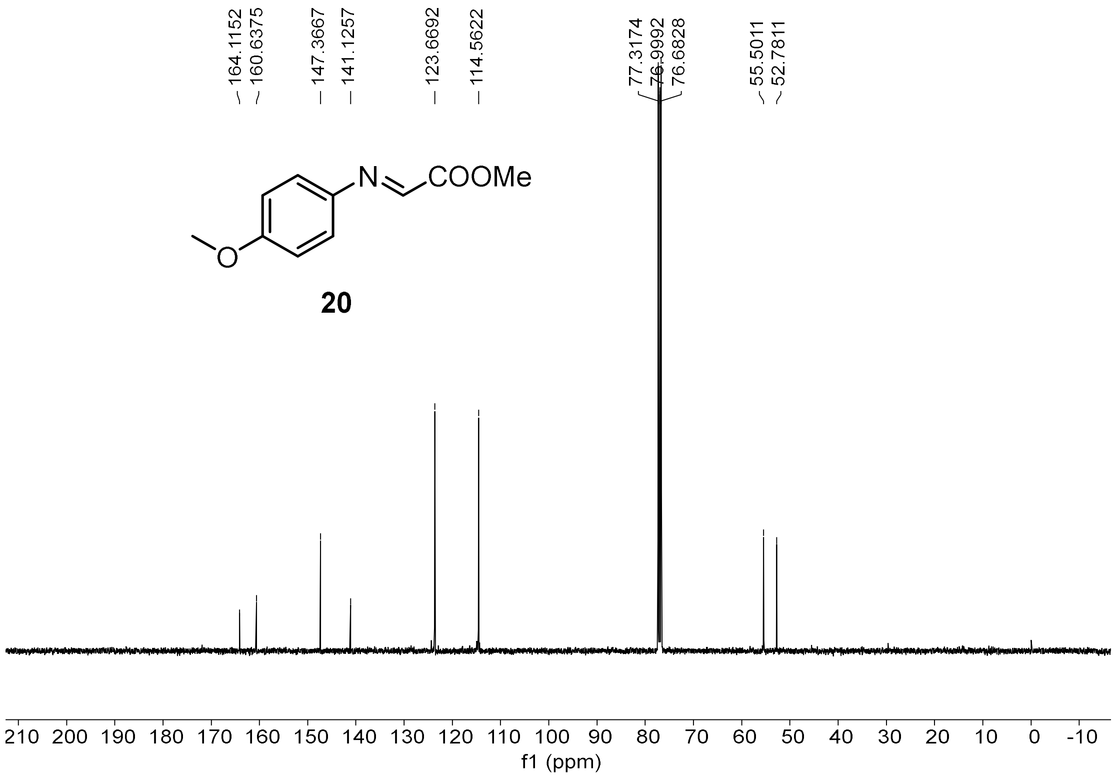

^{13}C NMR (100 MHz, $CDCl_3$) of **20**

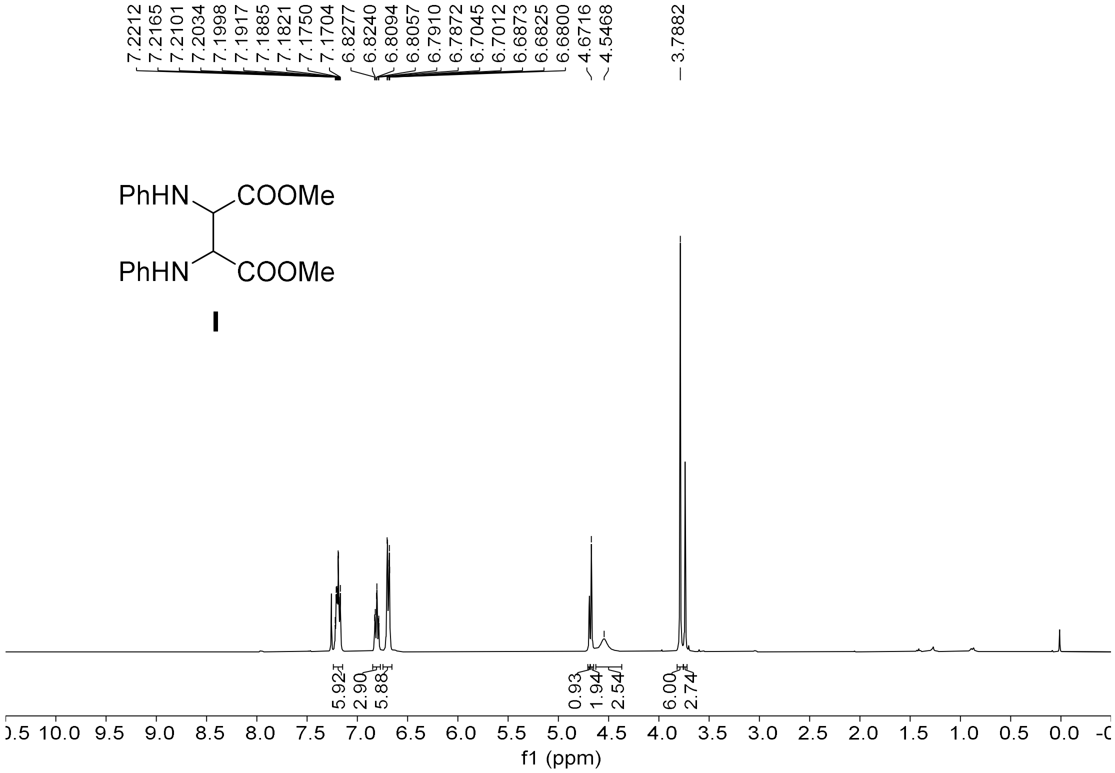

^{1}H NMR (400 MHz, $CDCl_3$) of **I**

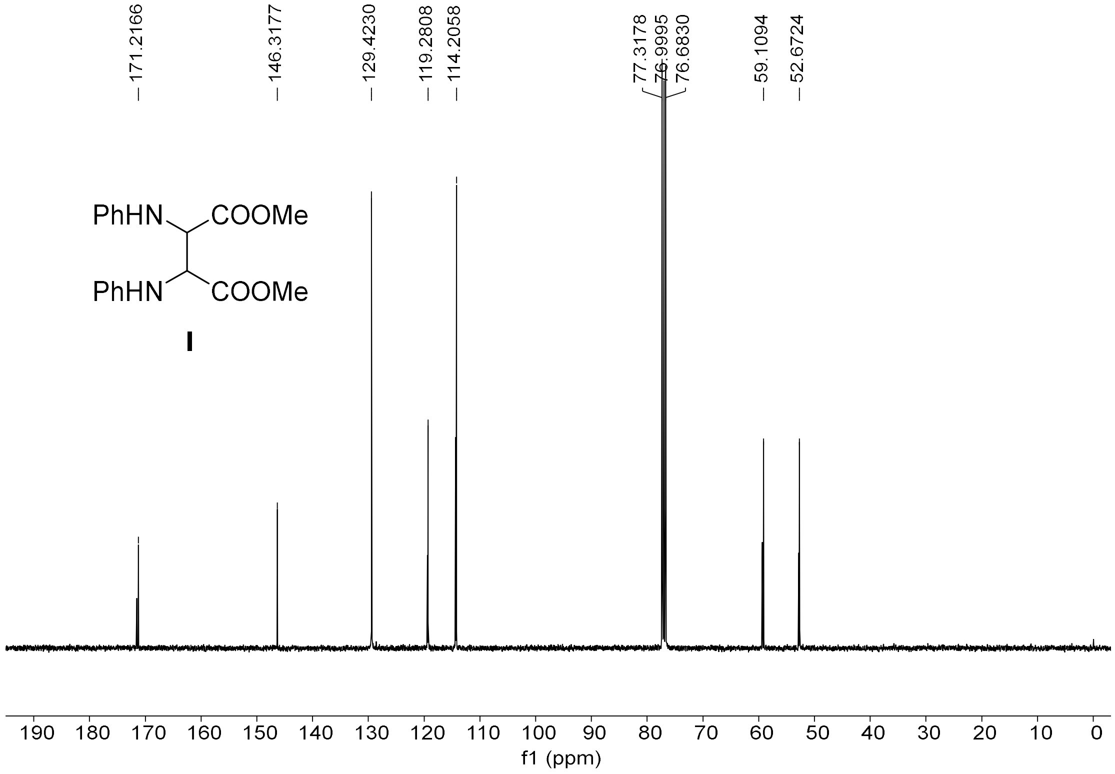

^{13}C NMR (100 MHz, $CDCl_3$) of **I**

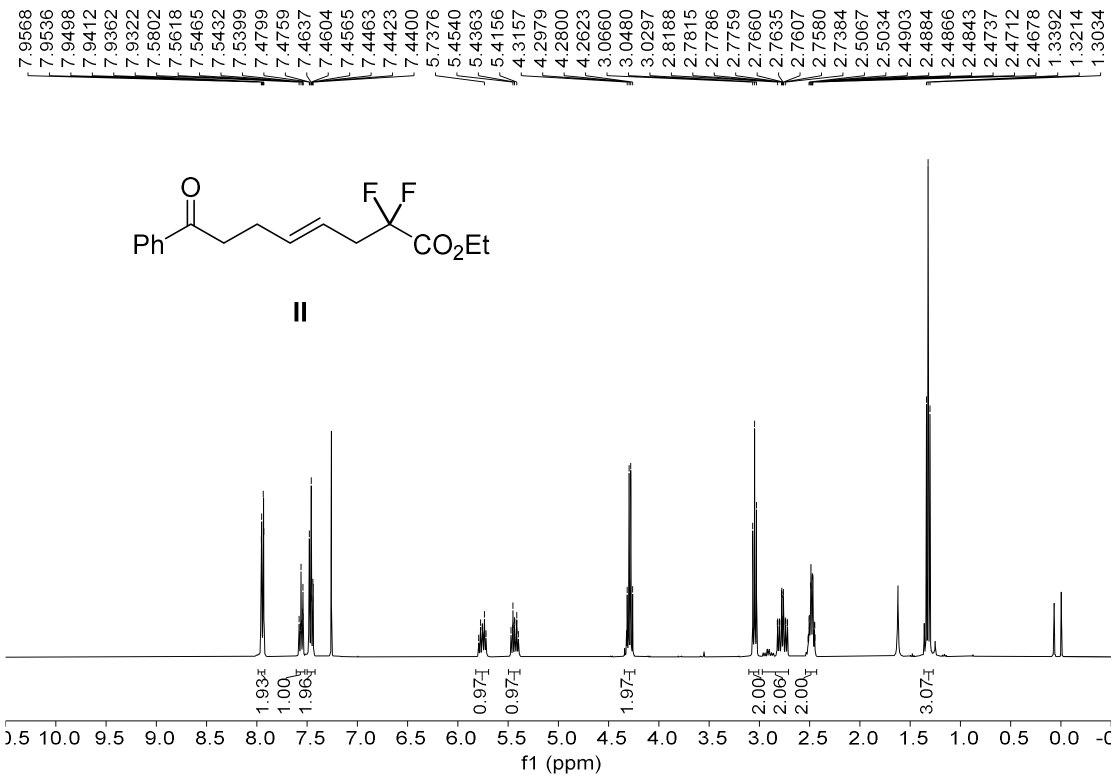

^{1}H NMR (400 MHz, $CDCl_3$) of **II**

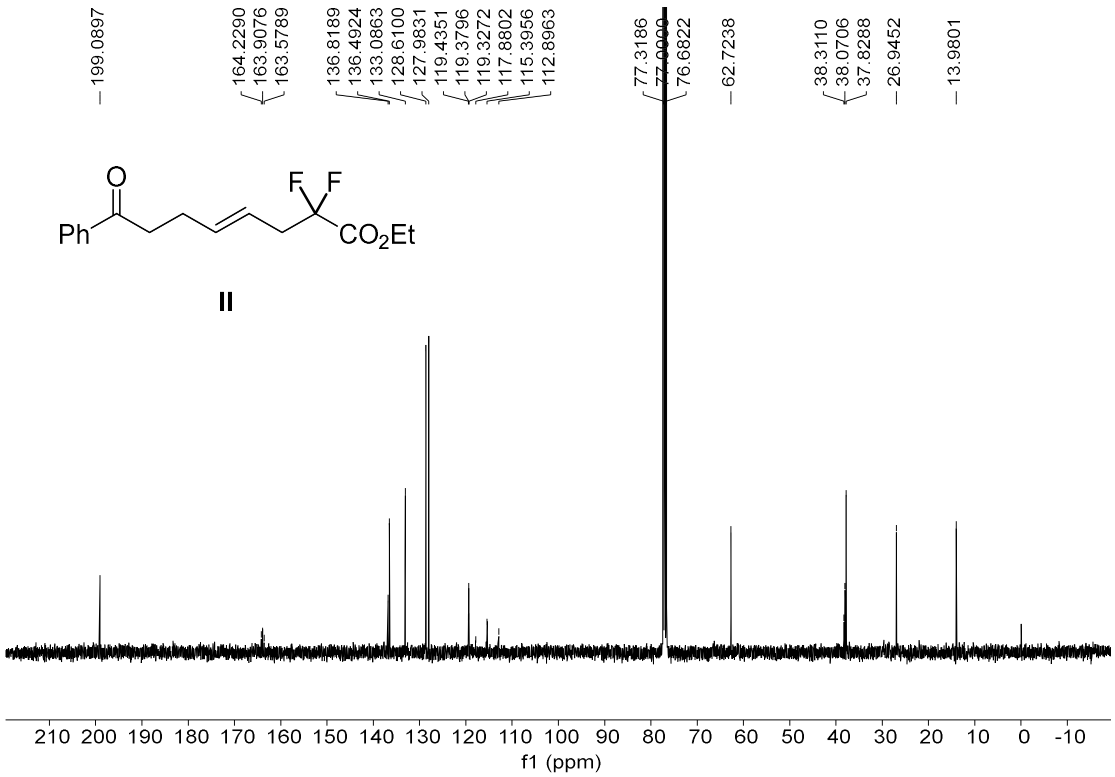

^{13}C NMR (100 MHz, $CDCl_3$) of **II**

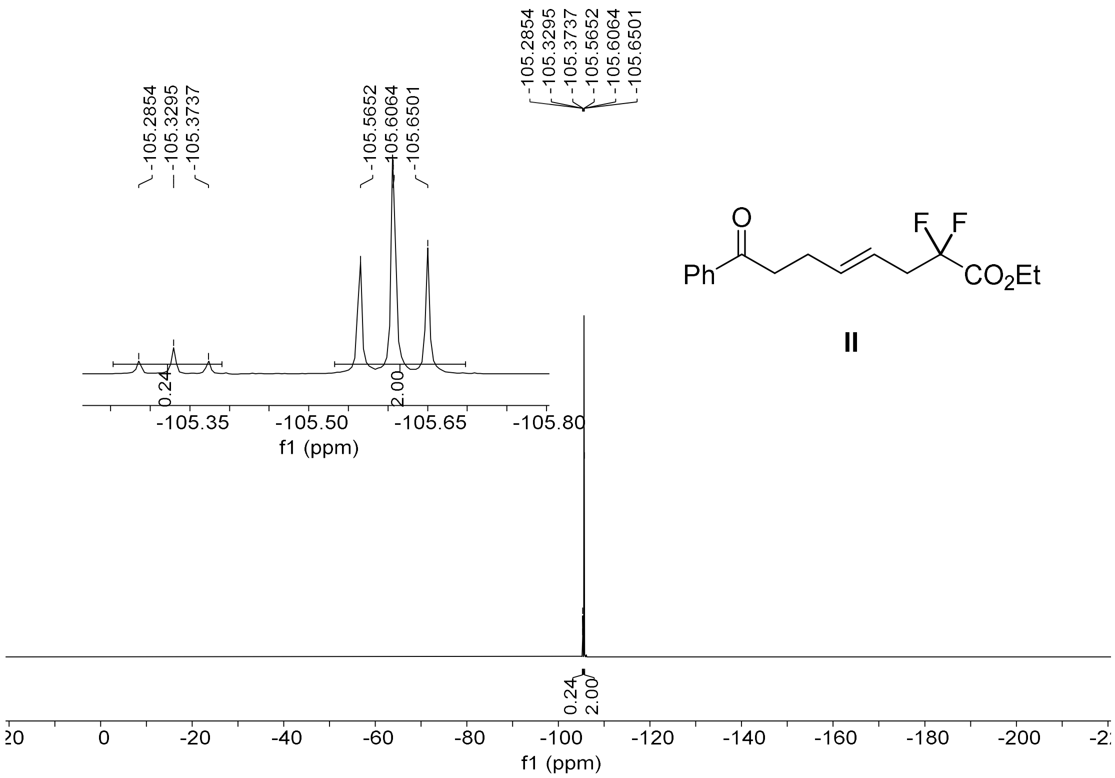

^{19}F NMR (376 MHz, $CDCl_3$) of **II**

HPLC spectra

HPLC chromatogram of compound (*rac*)-4a

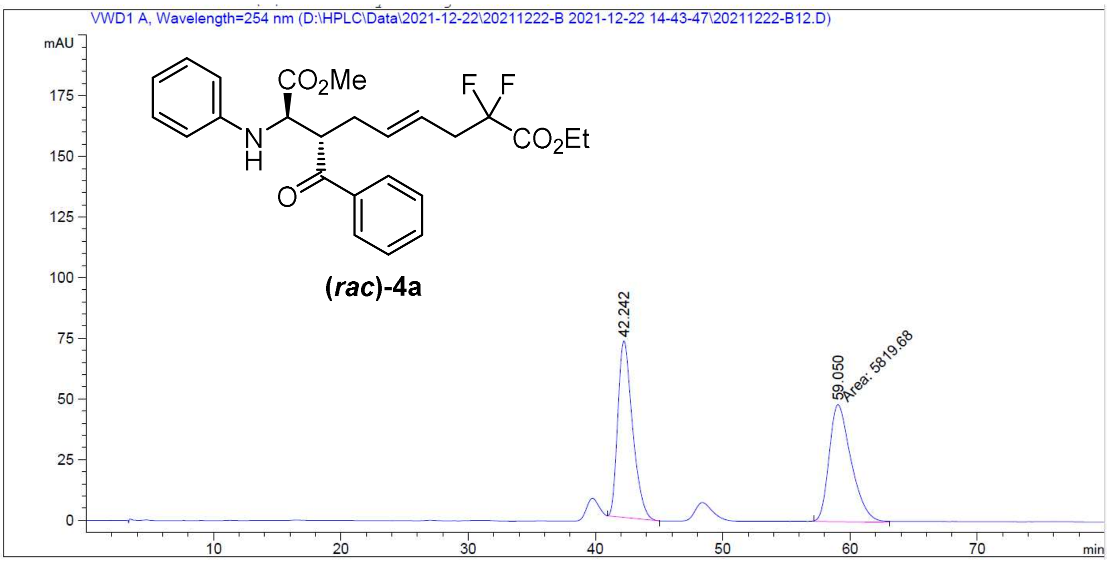

Peak #	RetTime [min]	Type	Width [min]	Area [mAU*s]	Height [mAU]	Area %
1	42.242	BB	1.2047	5845.75244	72.73142	50.1117
2	59.050	MM	2.0102	5819.68262	48.25151	49.8883

HPLC chromatogram of compound 4a

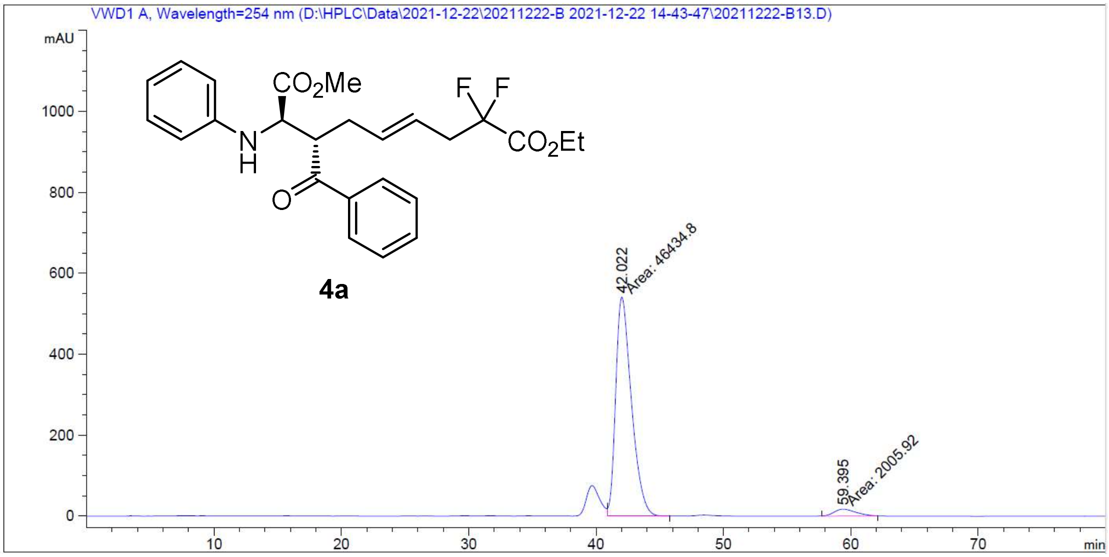

Peak #	RetTime [min]	Type	Width [min]	Area [mAU*s]	Height [mAU]	Area %
1	42.022	FM	1.4311	4.64348e4	540.77051	95.8590
2	59.395	MM	1.9599	2005.91663	17.05787	4.1410

HPLC chromatogram of compound (*rac*)-4b

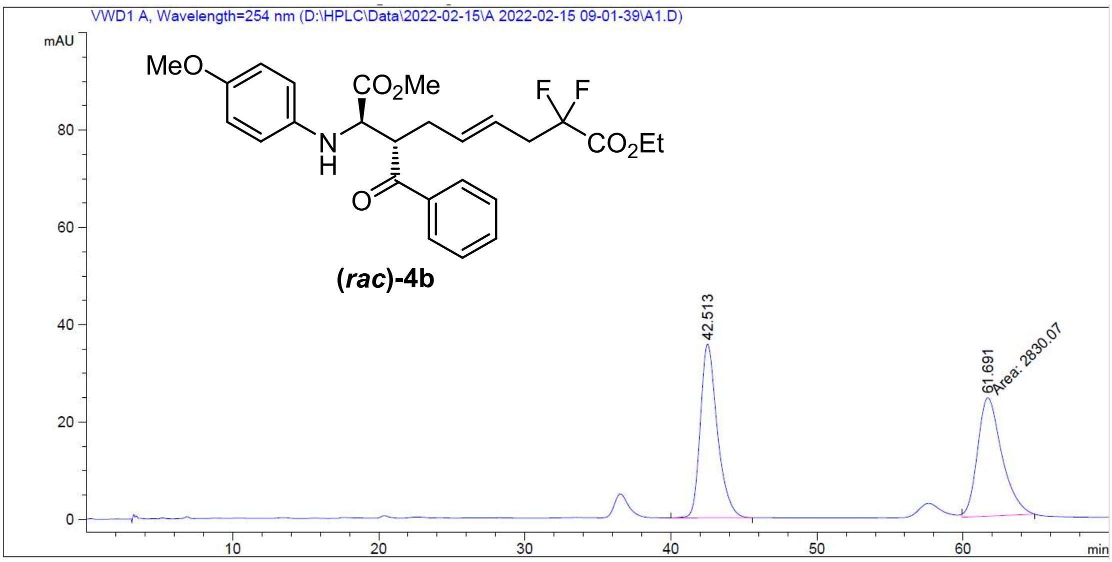

Peak #	RetTime [min]	Type	Width [min]	Area [mAU*s]	Height [mAU]	Area %
1	42.513	BB	1.2155	2892.95825	35.65883	50.5494
2	61.691	FM	1.9388	2830.07397	24.32845	49.4506

HPLC chromatogram of compound 4b

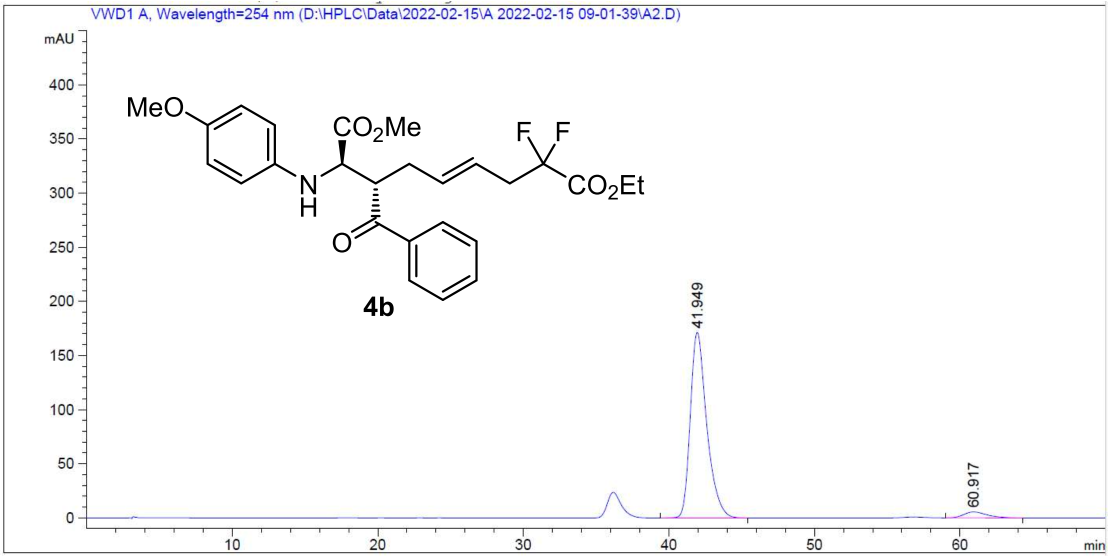

Peak #	RetTime [min]	Type	Width [min]	Area [mAU*s]	Height [mAU]	Area %
1	41.949	BB	1.1784	1.34882e4	171.34164	95.5364
2	60.917	BB	1.4055	630.19775	5.61468	4.4636

HPLC chromatogram of compound (*rac*)-4c

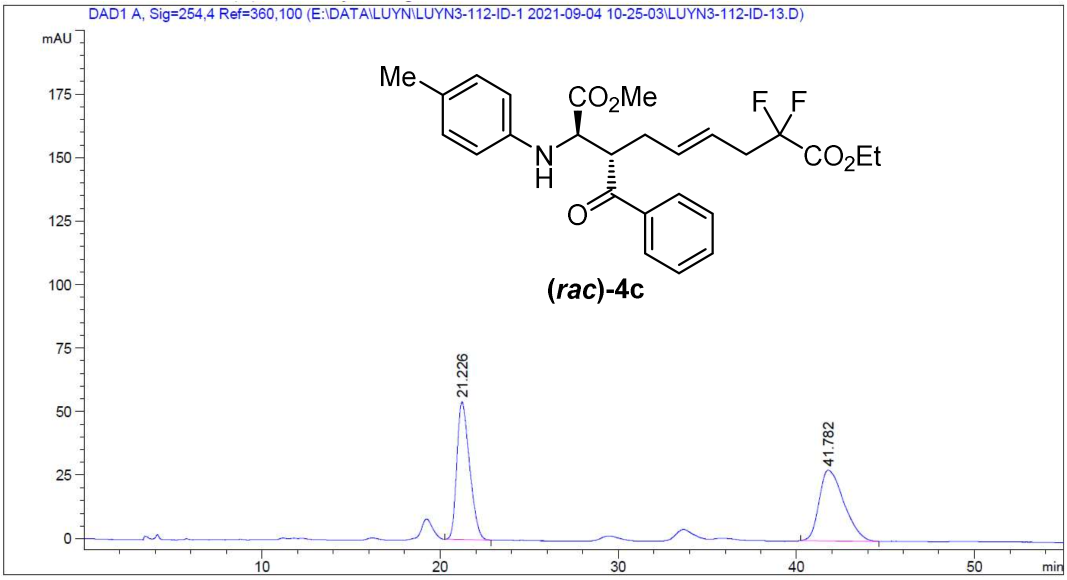

Peak #	RetTime [min]	Type	Width [min]	Area [mAU*s]	Height [mAU]	Area %
1	21.226	BB	0.7269	2694.20093	54.20417	50.0532
2	41.782	BB	1.1322	2688.47339	27.82532	49.9468

HPLC chromatogram of compound 4c

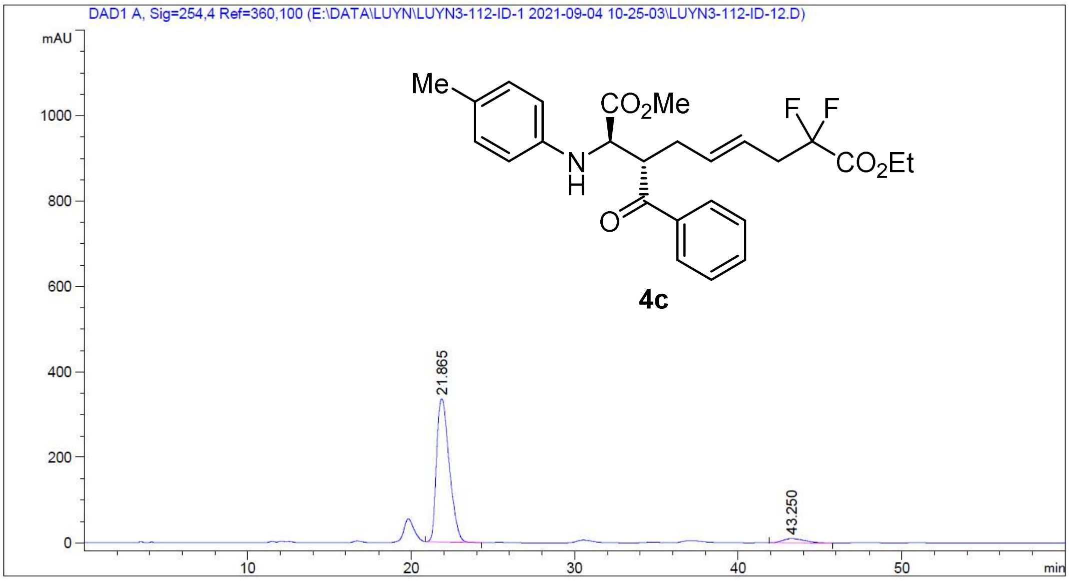

Peak #	RetTime [min]	Type	Width [min]	Area [mAU*s]	Height [mAU]	Area %
1	21.865	BB	0.8106	1.81544e4	335.34503	95.1812
2	43.250	BB	1.0863	919.11395	9.97251	4.8188

HPLC chromatogram of compound 4d

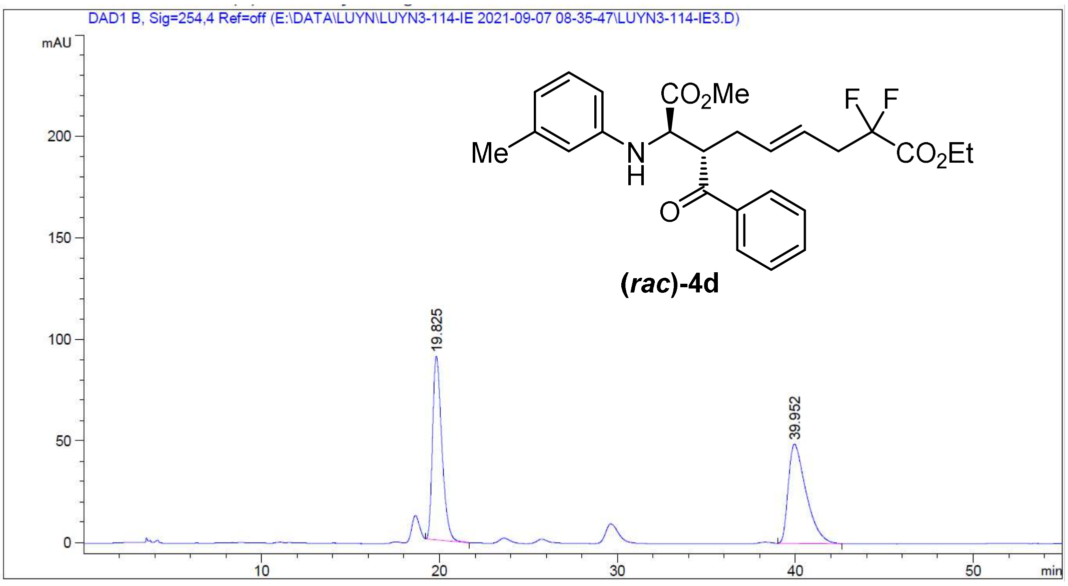

Peak #	RetTime [min]	Type	Width [min]	Area [mAU*s]	Height [mAU]	Area %
1	19.825	BB	0.5522	3287.11133	90.39571	49.0920
2	39.952	BB	0.9160	3408.71118	48.91398	50.9080

HPLC chromatogram of compound 4d

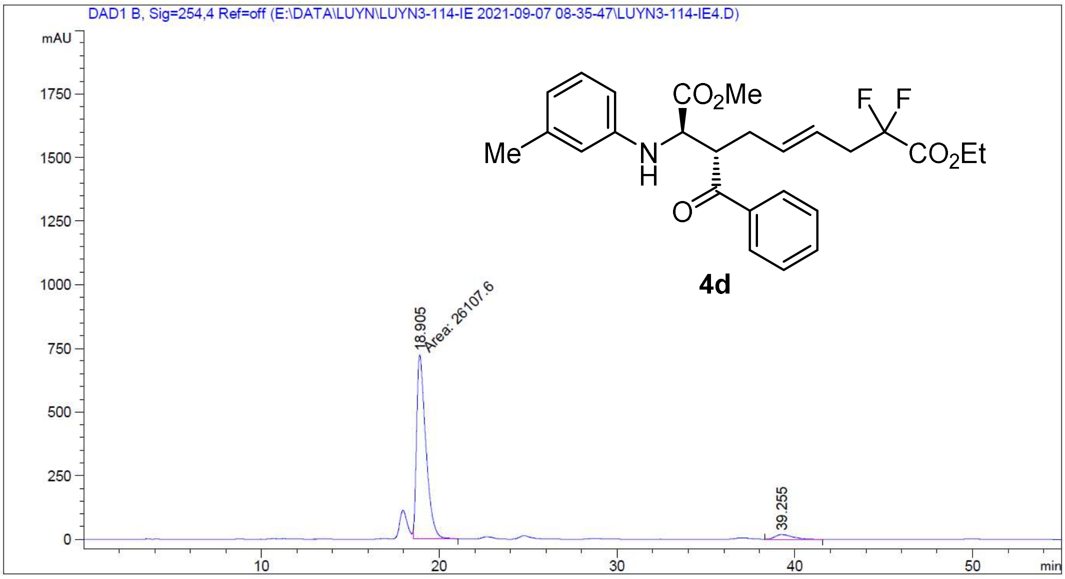

Peak #	RetTime [min]	Type	Width [min]	Area [mAU*s]	Height [mAU]	Area %
1	18.905	FM	0.6026	2.61076e4	722.07123	95.5148
2	39.255	BB	0.7897	1225.96533	18.20501	4.4852

HPLC chromatogram of compound (*rac*)-4e

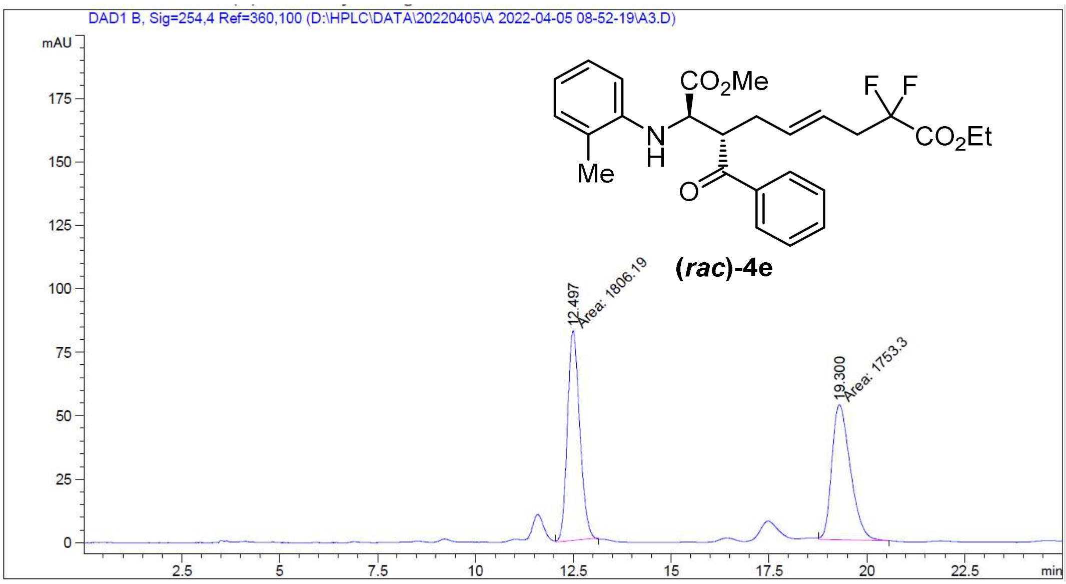

Peak #	RetTime [min]	Type	Width [min]	Area [mAU*s]	Height [mAU]	Area %
1	12.497	MM	0.3648	1806.18713	82.51814	50.7429
2	19.300	MM	0.5494	1753.30298	53.19041	49.2571

HPLC chromatogram of compound 4e

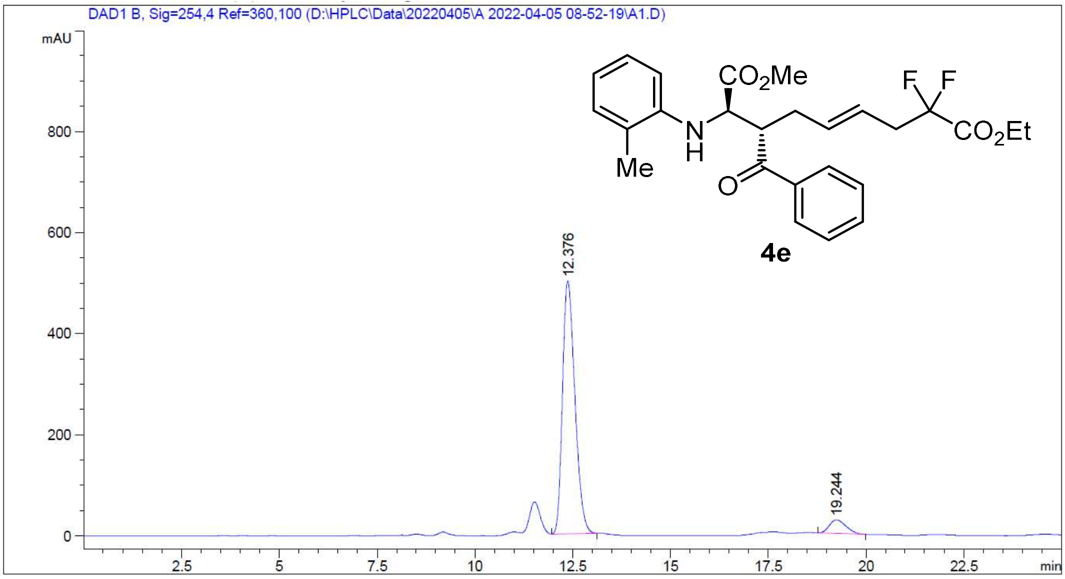

Peak #	RetTime [min]	Type	Width [min]	Area [mAU*s]	Height [mAU]	Area %
1	12.376	VB	0.3371	1.10277e4	500.49216	93.3214
2	19.244	BB	0.4241	789.20862	26.83383	6.6786

HPLC chromatogram of compound (*rac*)-4f

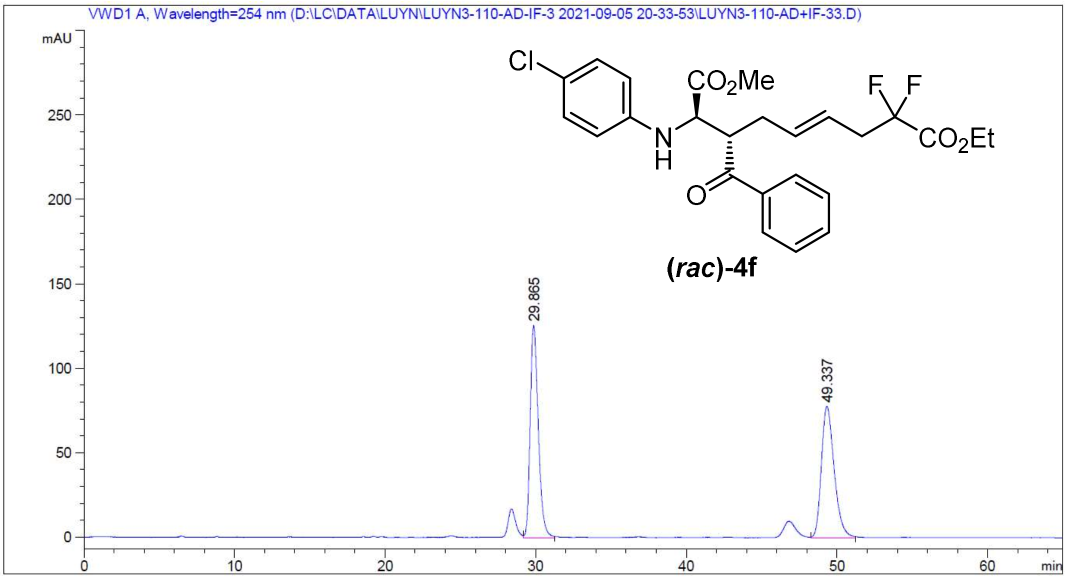

Peak #	RetTime [min]	Type	Width [min]	Area [mAU*s]	Height [mAU]	Area %
1	29.865	VB	0.5704	4701.57959	125.68415	50.4956
2	49.337	VV	0.8913	4609.29736	77.73145	49.5044

HPLC chromatogram of compound 4f

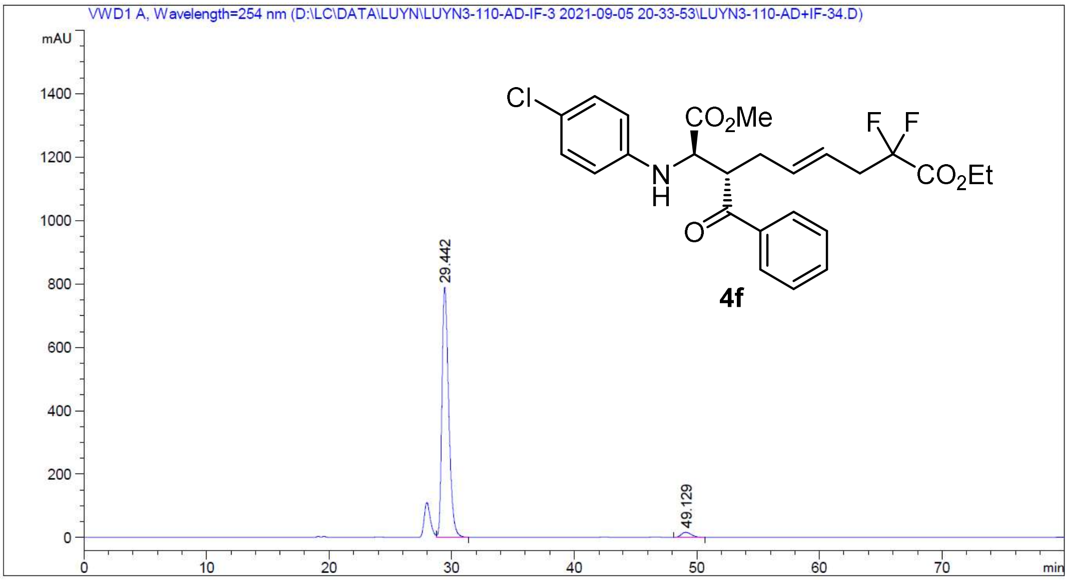

Peak #	RetTime [min]	Type	Width [min]	Area [mAU*s]	Height [mAU]	Area %
1	29.442	VV	0.5931	3.04388e4	791.03802	96.7695
2	49.129	VV	0.7242	1016.14496	16.84435	3.2305

HPLC chromatogram of compound (*rac*)-4g

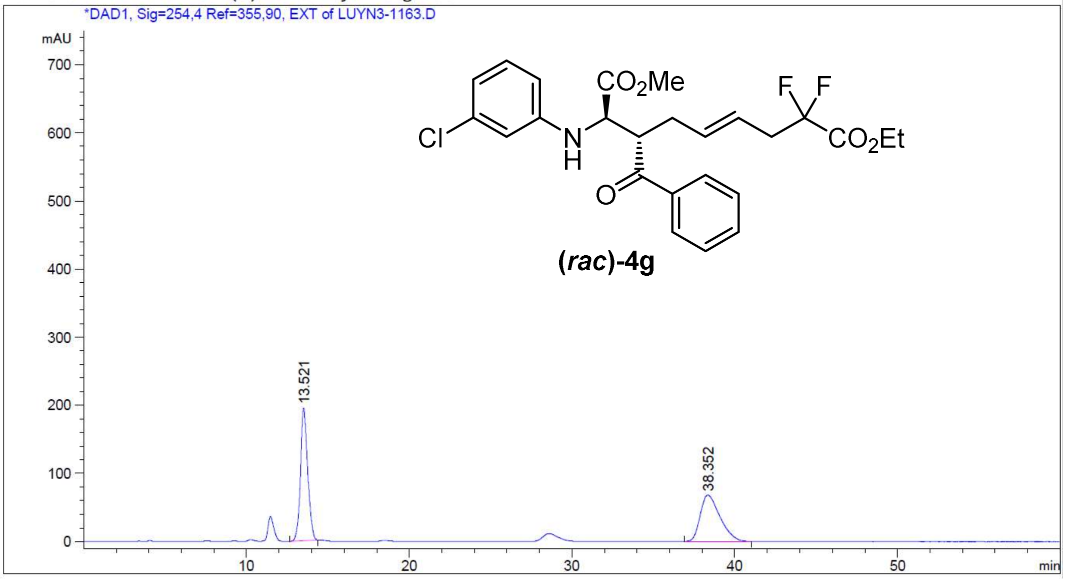

Peak #	RetTime [min]	Type	Width [min]	Area [mAU*s]	Height [mAU]	Area %
1	13.521	BB	0.4537	5973.25098	194.83295	51.0522
2	38.352	BB	1.0538	5727.02588	68.05382	48.9478

HPLC chromatogram of compound 4g

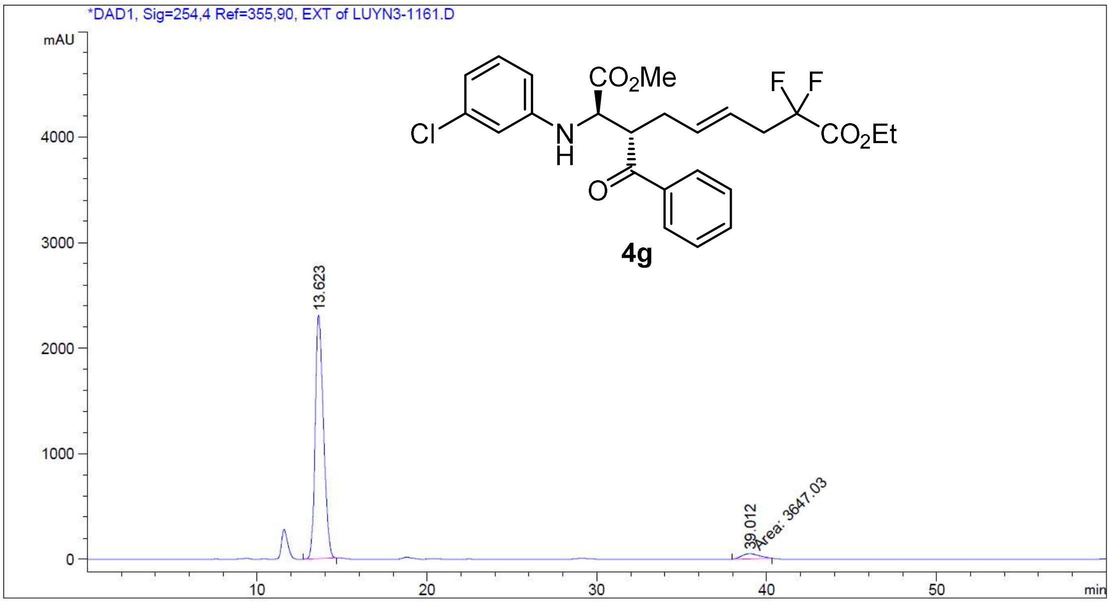

Peak #	RetTime [min]	Type	Width [min]	Area [mAU*s]	Height [mAU]	Area %
1	13.623	BB	0.5070	7.77702e4	2303.10986	95.5206
2	39.012	MM	0.8787	3647.02734	49.28520	4.4794

HPLC chromatogram of compound (*rac*)-4h

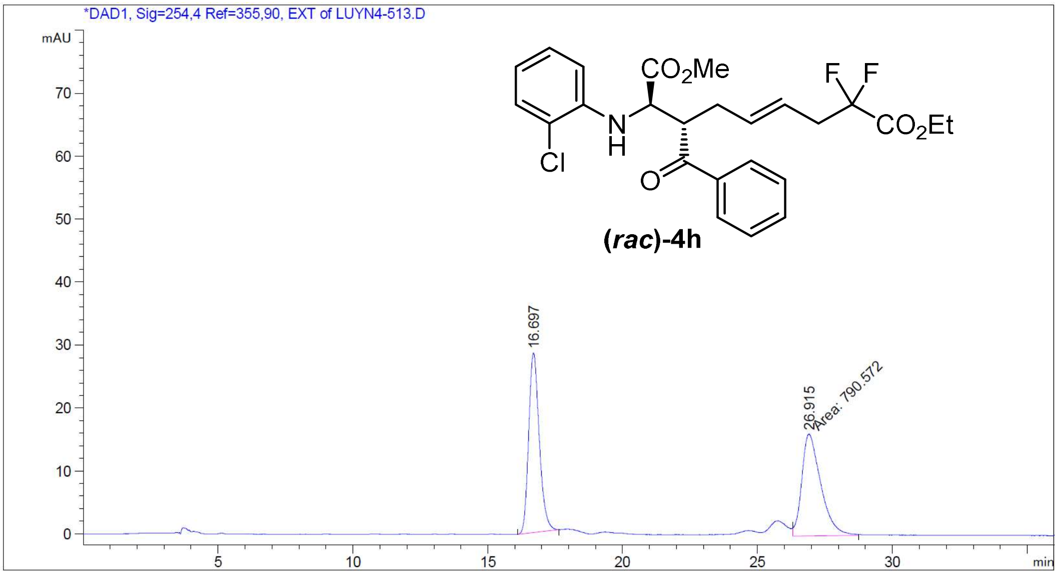

Peak #	RetTime [min]	Type	Width [min]	Area [mAU*s]	Height [mAU]	Area %
1	16.697	BB	0.4085	778.86682	28.52939	49.6271
2	26.915	FM	0.8153	790.57153	16.16183	50.3729

HPLC chromatogram of compound 4h

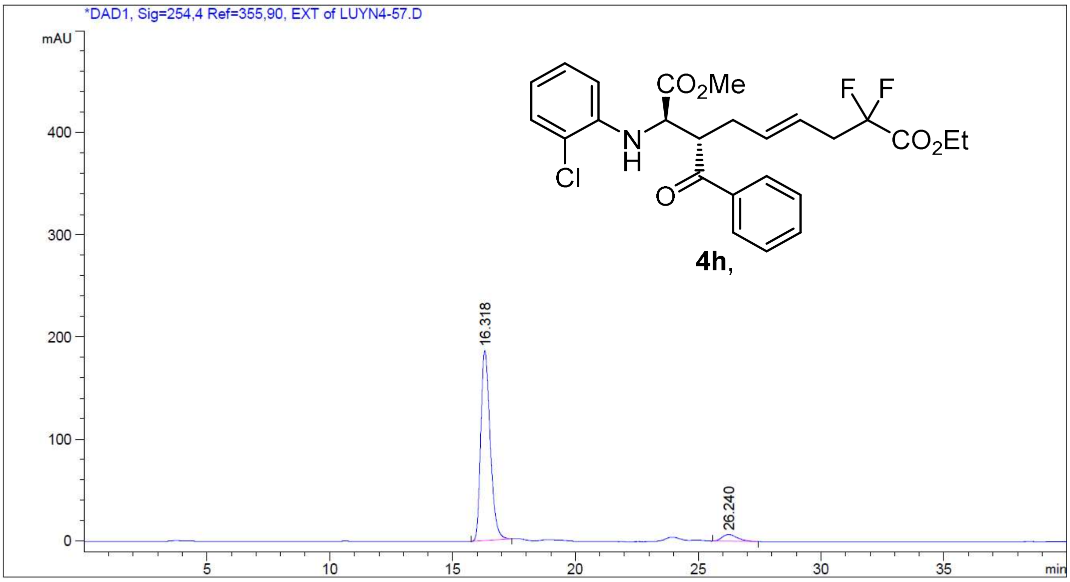

Peak #	RetTime [min]	Type	Width [min]	Area [mAU*s]	Height [mAU]	Area %
1	16.318	BB	0.4152	4977.98682	186.02182	94.4885
2	26.240	BB	0.5168	290.36435	6.60543	5.5115

HPLC chromatogram of compound (*rac*)-4i

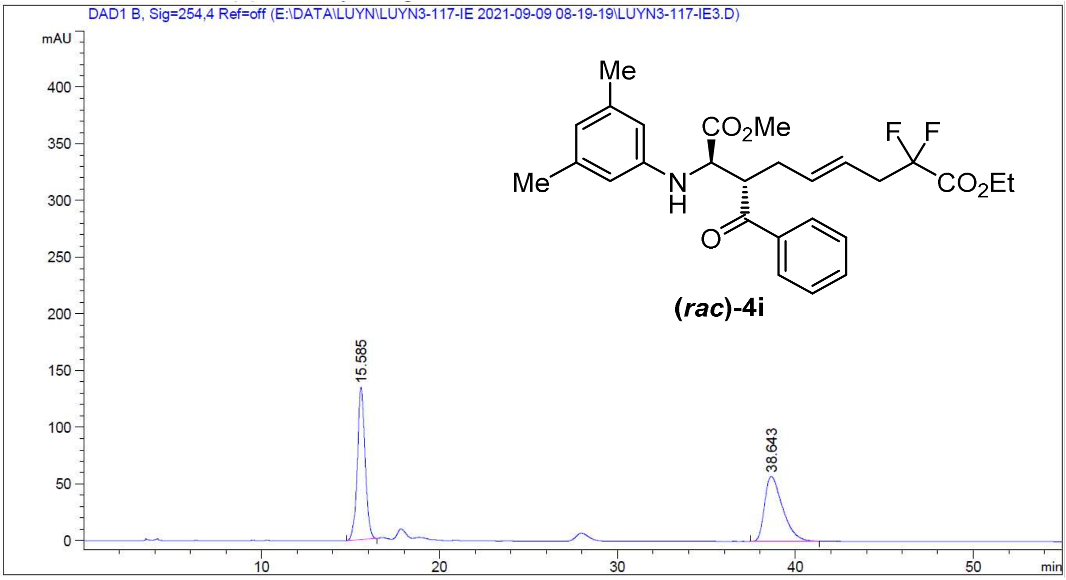

Peak #	RetTime [min]	Type	Width [min]	Area [mAU*s]	Height [mAU]	Area %
1	15.585	BB	0.4469	4053.10107	134.38417	50.2452
2	38.643	BB	0.9453	4013.53687	57.29015	49.7548

HPLC chromatogram of compound 4i

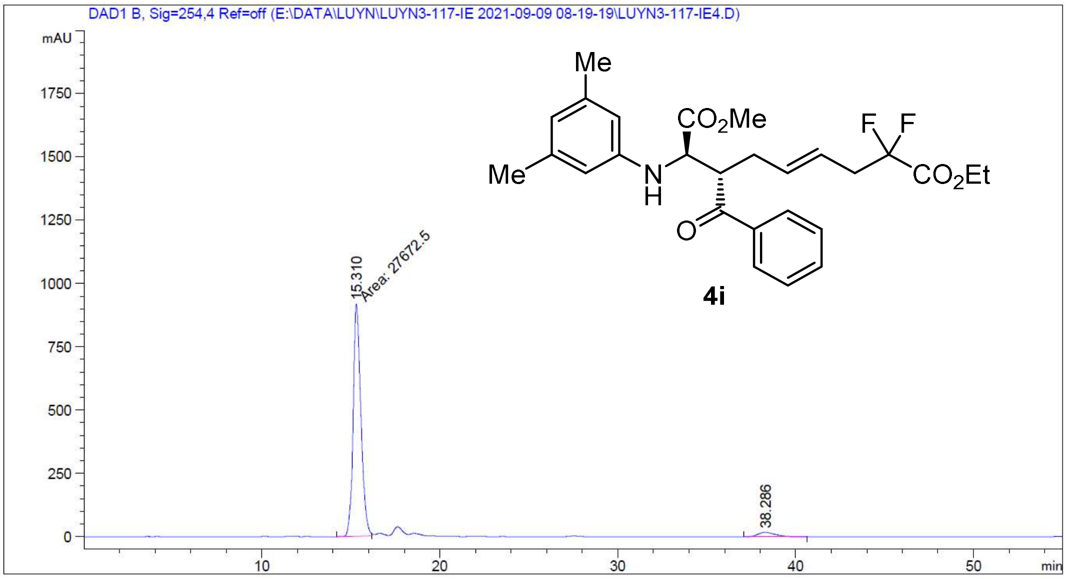

Peak #	RetTime [min]	Type	Width [min]	Area [mAU*s]	Height [mAU]	Area %
1	15.310	MF	0.5036	2.76725e4	915.81689	95.7382
2	38.286	BB	0.8157	1231.83484	17.92512	4.2618

HPLC chromatogram of compound (*rac*)-4j

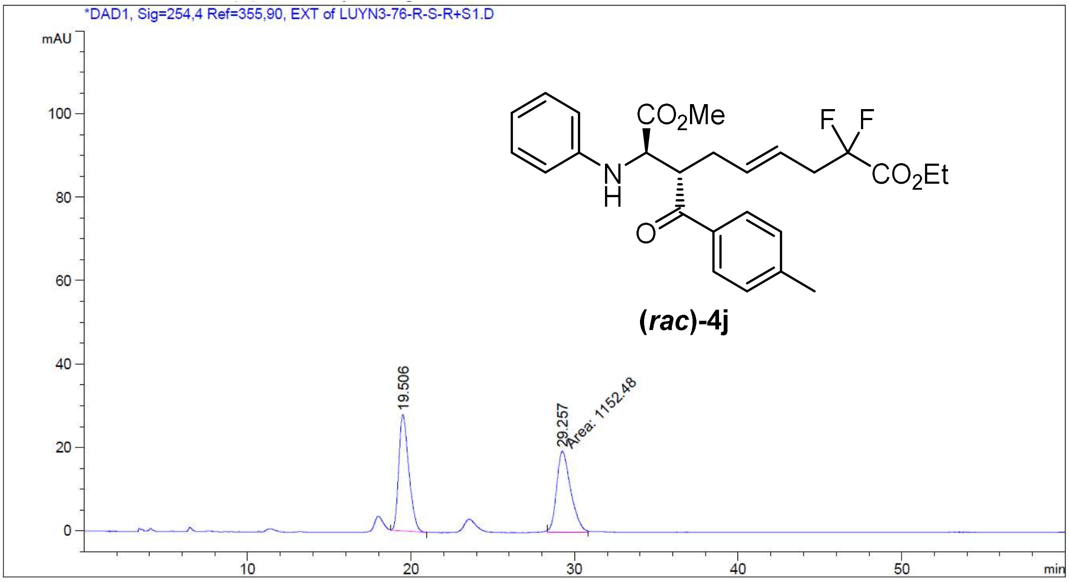

Peak #	RetTime [min]	Type	Width [min]	Area [mAU*s]	Height [mAU]	Area %
1	19.506	BB	0.5700	1187.41125	27.87202	50.7465
2	29.257	MM	0.9921	1152.47766	19.36137	49.2535

HPLC chromatogram of compound 4j

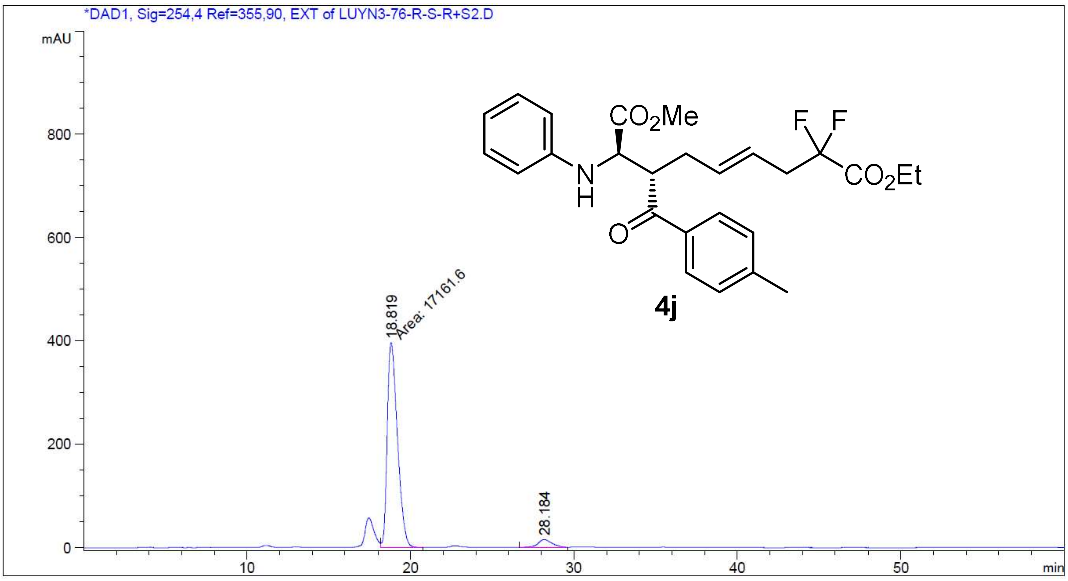

Peak #	RetTime [min]	Type	Width [min]	Area [mAU*s]	Height [mAU]	Area %
1	18.819	FM	0.7220	1.71616e4	396.16452	95.0608
2	28.184	BB	0.7029	891.69556	14.99307	4.9392

HPLC chromatogram of compound (*rac*)-4k

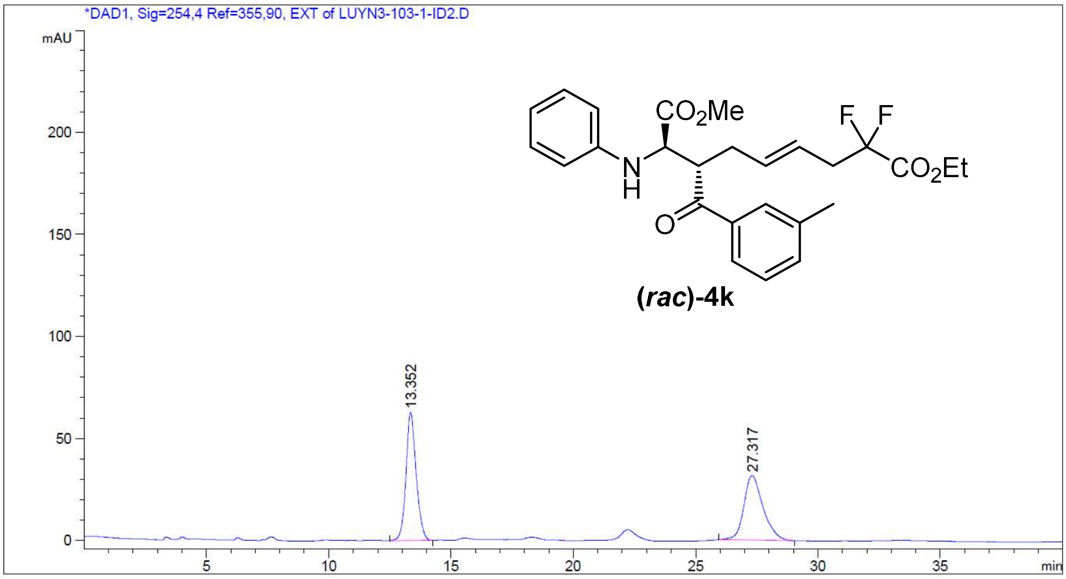

Peak #	RetTime [min]	Type	Width [min]	Area [mAU*s]	Height [mAU]	Area %
1	13.352	BB	0.4193	1816.13330	62.81435	51.3365
2	27.317	BB	0.7060	1721.57031	31.58039	48.6635

HPLC chromatogram of compound 4k

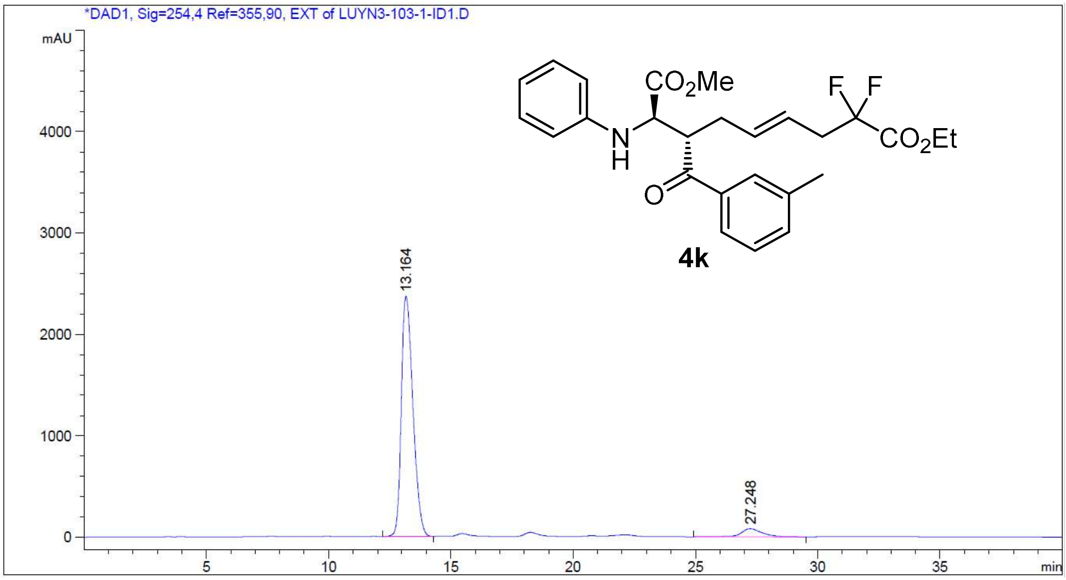

Peak #	RetTime [min]	Type	Width [min]	Area [mAU*s]	Height [mAU]	Area %
1	13.164	BB	0.4390	7.80281e4	2370.59839	94.1807
2	27.248	BB	0.8521	4821.28662	79.76994	5.8193

HPLC chromatogram of compound (*rac*)-4l

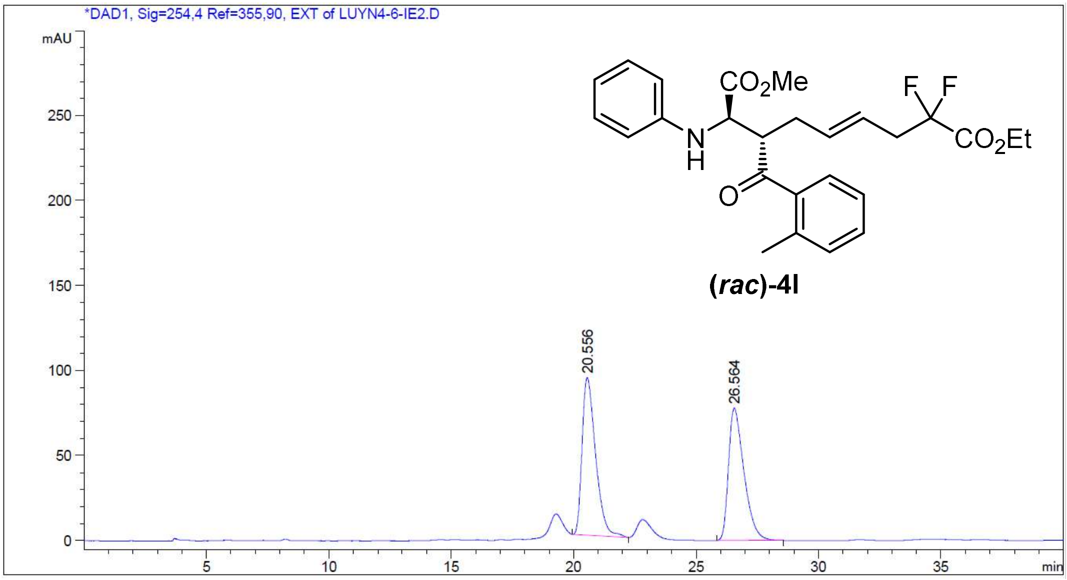

Peak #	RetTime [min]	Type	Width [min]	Area [mAU*s]	Height [mAU]	Area %
1	20.556	BB	0.5724	3564.16016	92.73376	51.1256
2	26.564	BB	0.6280	3407.22363	77.78680	48.8744

HPLC chromatogram of compound 4l

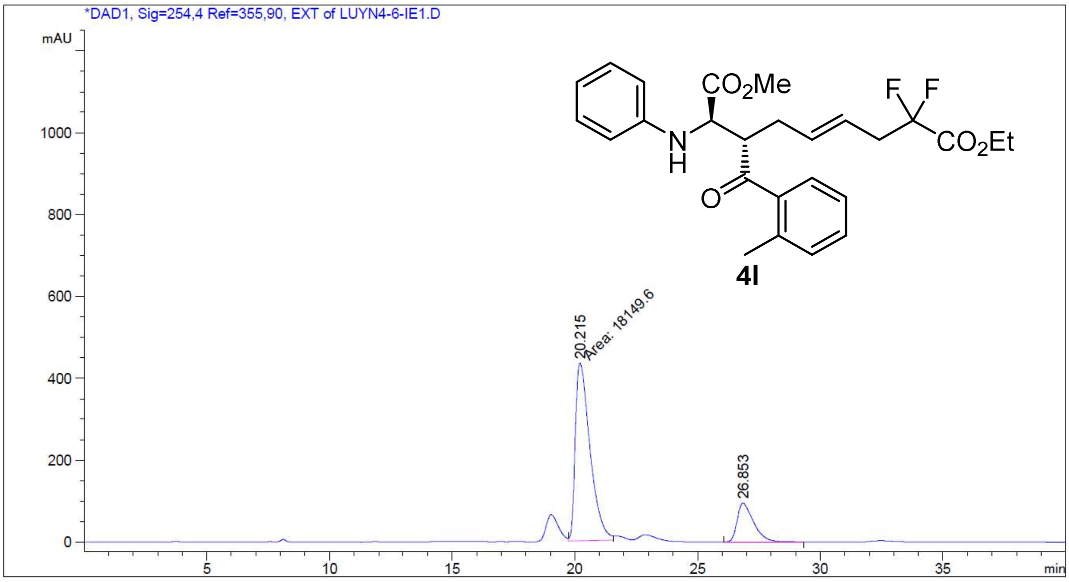

Peak #	RetTime [min]	Type	Width [min]	Area [mAU*s]	Height [mAU]	Area %
1	20.215	MF	0.6972	1.81496e4	433.90009	80.6123
2	26.853	BB	0.6473	4365.06689	94.77248	19.3877

HPLC chromatogram of compound (*rac*)-4m

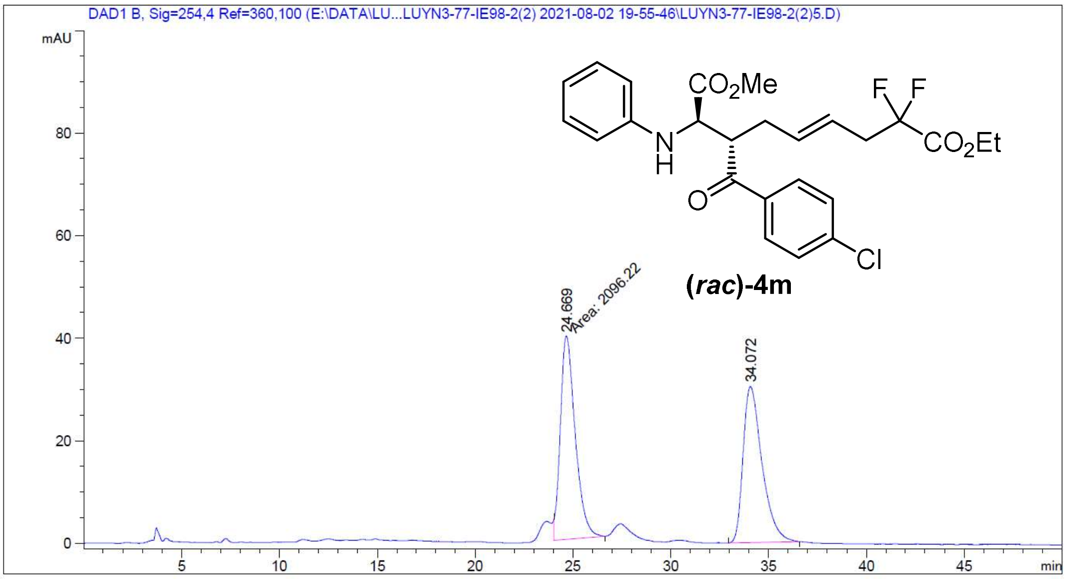

Peak #	RetTime [min]	Type	Width [min]	Area [mAU*s]	Height [mAU]	Area %
1	24.669	FM	0.8804	2096.22144	39.68259	50.5910
2	34.072	BB	0.8539	2047.24329	30.39001	49.4090

HPLC chromatogram of compound 4m

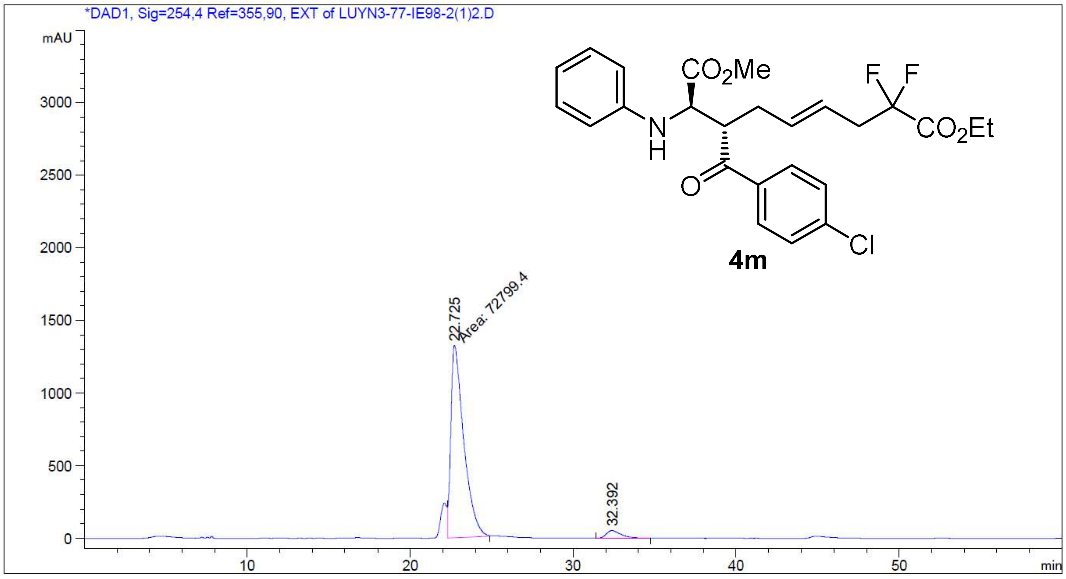

Peak #	RetTime [min]	Type	Width [min]	Area [mAU*s]	Height [mAU]	Area %
1	22.725	FM	0.9156	7.27994e4	1325.21533	95.7946
2	32.392	BB	0.7666	3195.92578	52.03406	4.2054

HPLC chromatogram of compound (*rac*)-4n

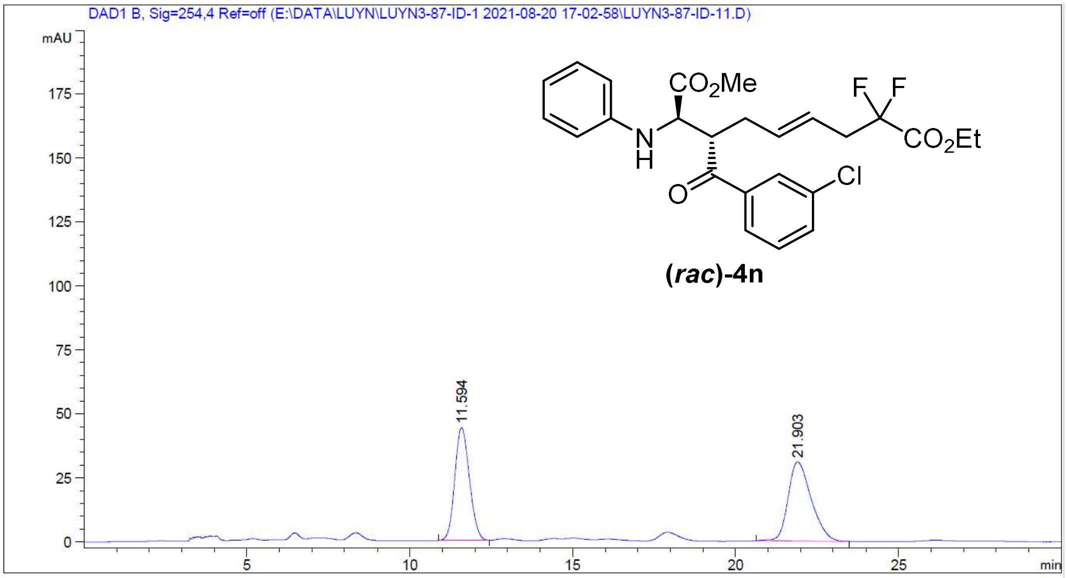

Peak #	RetTime [min]	Type	Width [min]	Area [mAU*s]	Height [mAU]	Area %
1	11.594	BB	0.4789	1375.13306	44.08582	47.4311
2	21.903	BB	0.6869	1524.08887	30.92839	52.5689

HPLC chromatogram of compound 4n

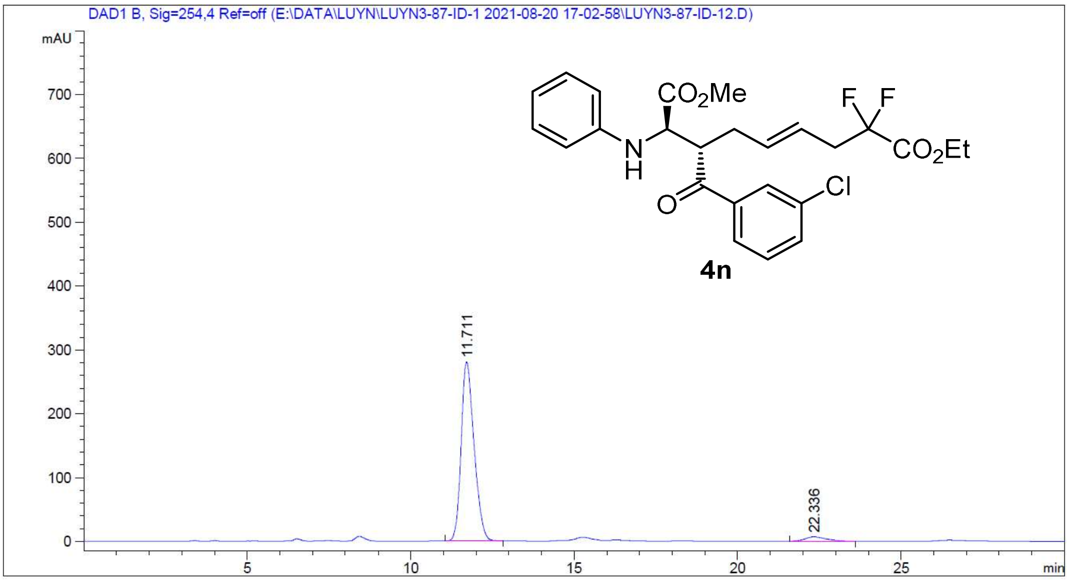

Peak #	RetTime [min]	Type	Width [min]	Area [mAU*s]	Height [mAU]	Area %
1	11.711	BB	0.4140	7697.70020	280.66037	96.0998
2	22.336	BB	0.5395	312.40665	6.80638	3.9002

HPLC chromatogram of compound (*rac*)-4o

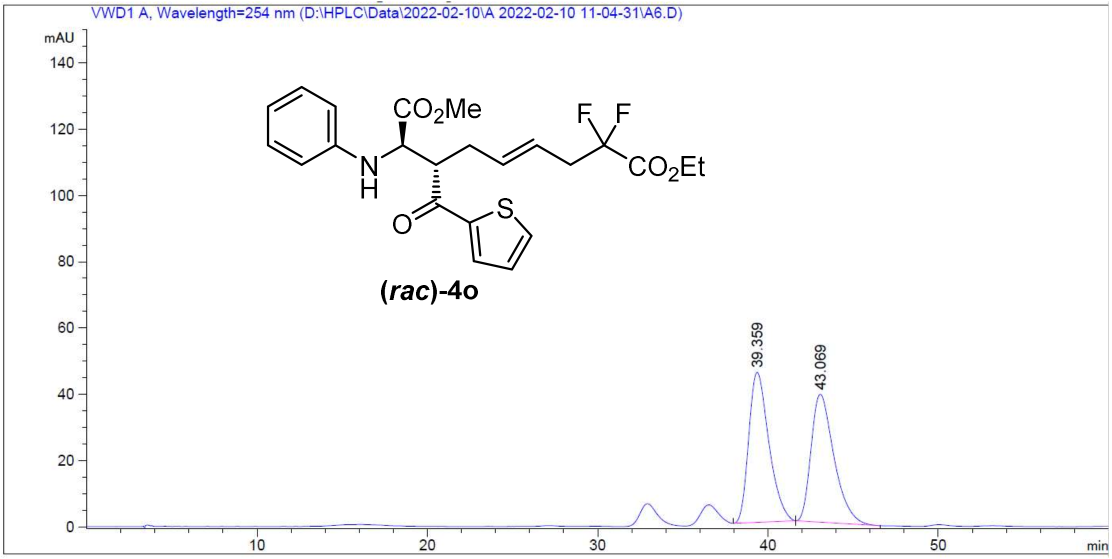

Peak #	RetTime [min]	Type	Width [min]	Area [mAU*s]	Height [mAU]	Area %
1	39.359	BB	1.2407	3731.76514	45.31676	50.8074
2	43.069	BB	1.3749	3613.15845	38.60524	49.1926

HPLC chromatogram of compound 4o

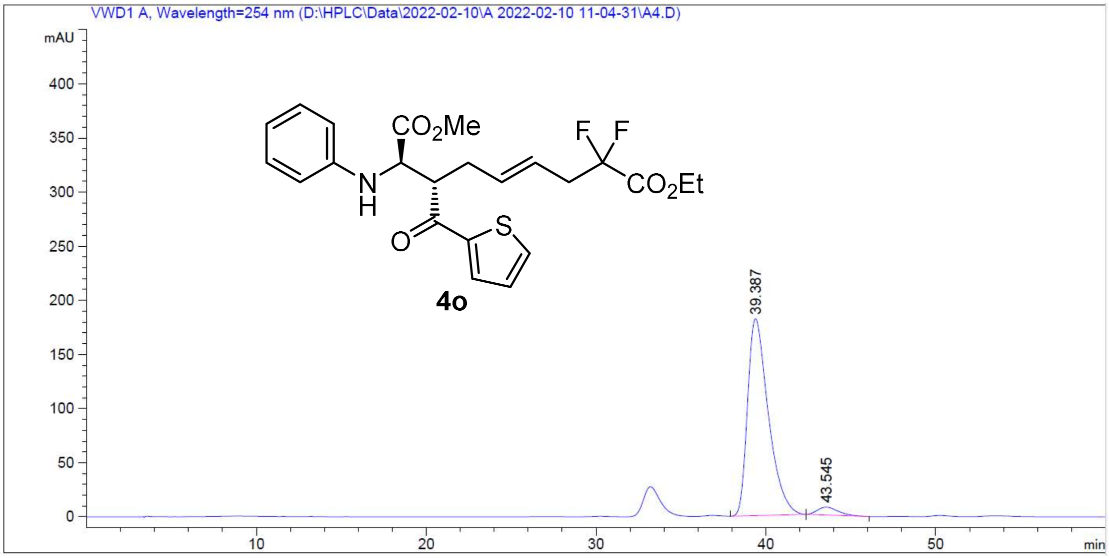

Peak #	RetTime [min]	Type	Width [min]	Area [mAU*s]	Height [mAU]	Area %
1	39.387	BB	1.2817	1.54699e4	181.98436	96.1492
2	43.545	BB	1.1685	619.56598	7.29000	3.8508

HPLC chromatogram of compound (*rac*)-4p

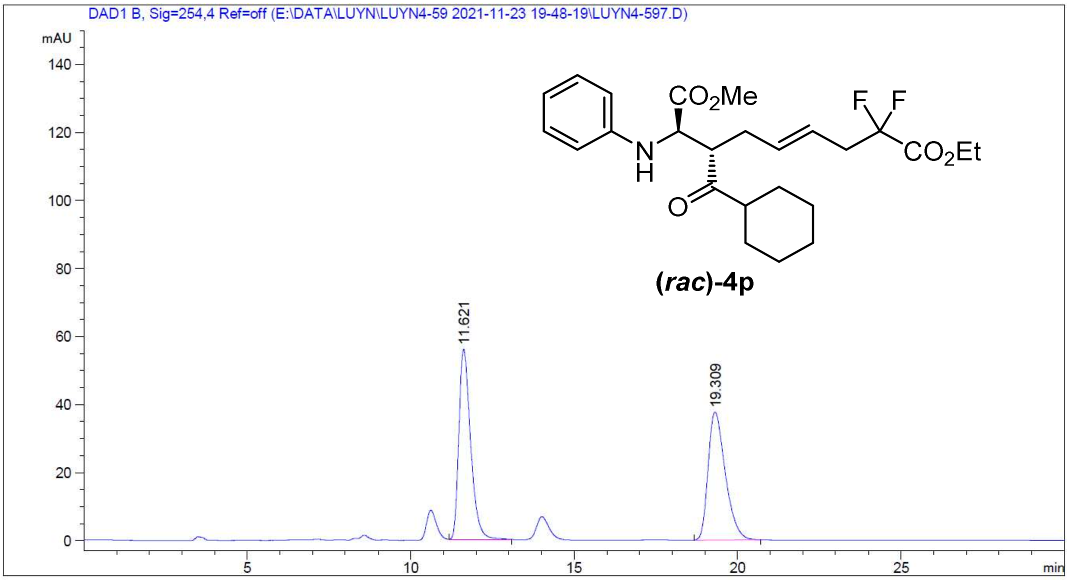

Peak #	RetTime [min]	Type	Width [min]	Area [mAU*s]	Height [mAU]	Area %
1	11.621	BB	0.3774	1397.38611	55.93901	50.3933
2	19.309	BB	0.5375	1375.57532	37.62614	49.6067

HPLC chromatogram of compound 4ap

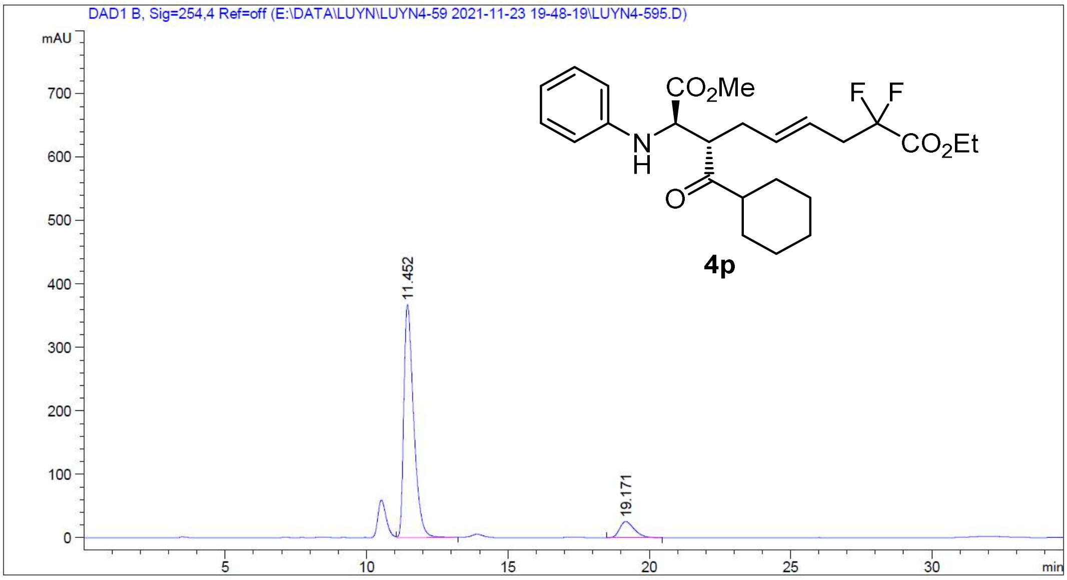

Peak #	RetTime [min]	Type	Width [min]	Area [mAU*s]	Height [mAU]	Area %
1	11.452	VB	0.3627	8830.17188	366.96182	90.5896
2	19.171	BB	0.5243	917.27448	25.46260	9.4104

HPLC chromatogram of compound (*rac*)-4q

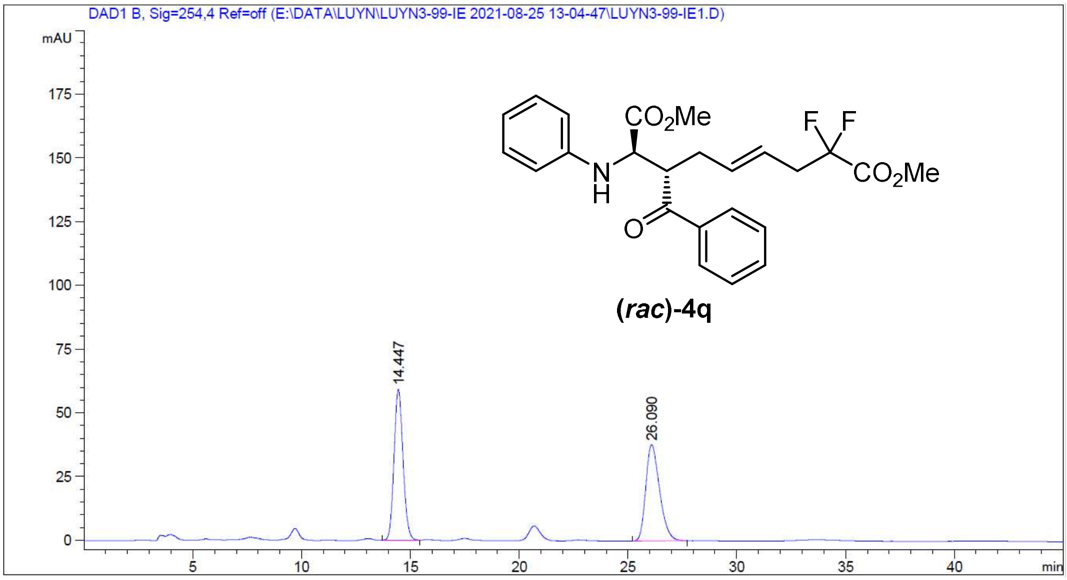

Peak #	RetTime [min]	Type	Width [min]	Area [mAU*s]	Height [mAU]	Area %
1	14.447	BB	0.4368	1702.96167	59.16632	50.5679
2	26.090	BB	0.6244	1664.71191	37.75824	49.4321

HPLC chromatogram of compound 4q

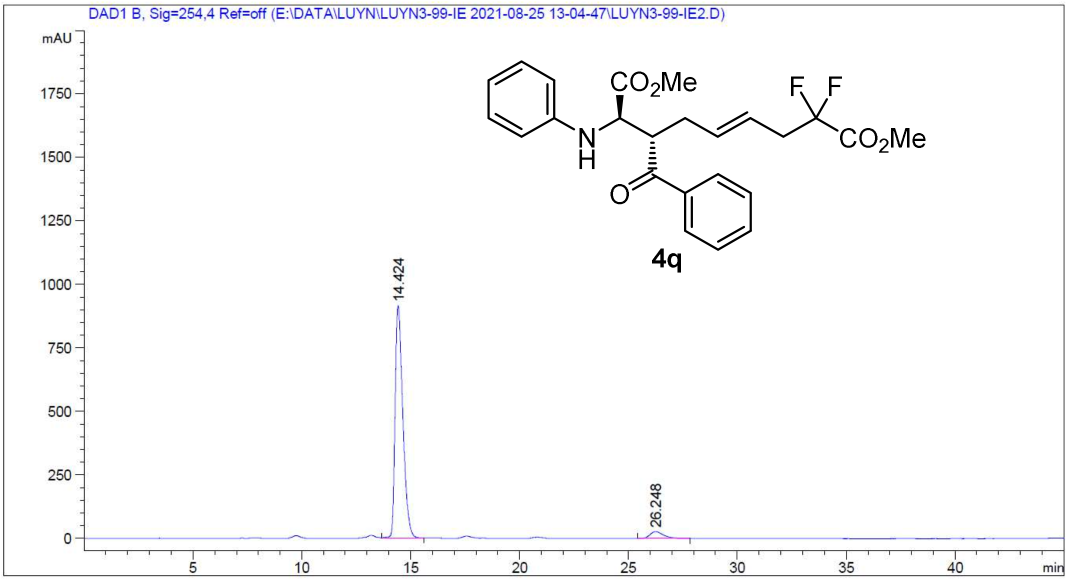

Peak #	RetTime [min]	Type	Width [min]	Area [mAU*s]	Height [mAU]	Area %
1	14.424	BB	0.3708	2.25559e4	914.35175	95.0383
2	26.248	BB	0.6043	1177.57959	27.24357	4.9617

HPLC chromatogram of compound (*rac*)-4r

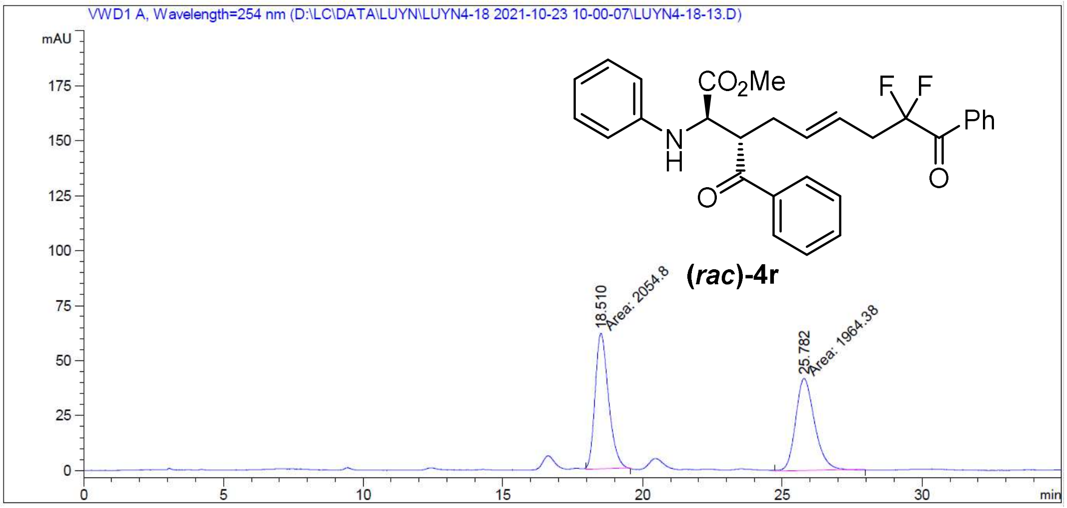

Peak #	RetTime [min]	Type	Width [min]	Area [mAU*s]	Height [mAU]	Area %
1	18.510	MM	0.5563	2054.80322	61.56619	51.1249
2	25.782	MM	0.7839	1964.38086	41.76451	48.8751

HPLC chromatogram of compound 4r

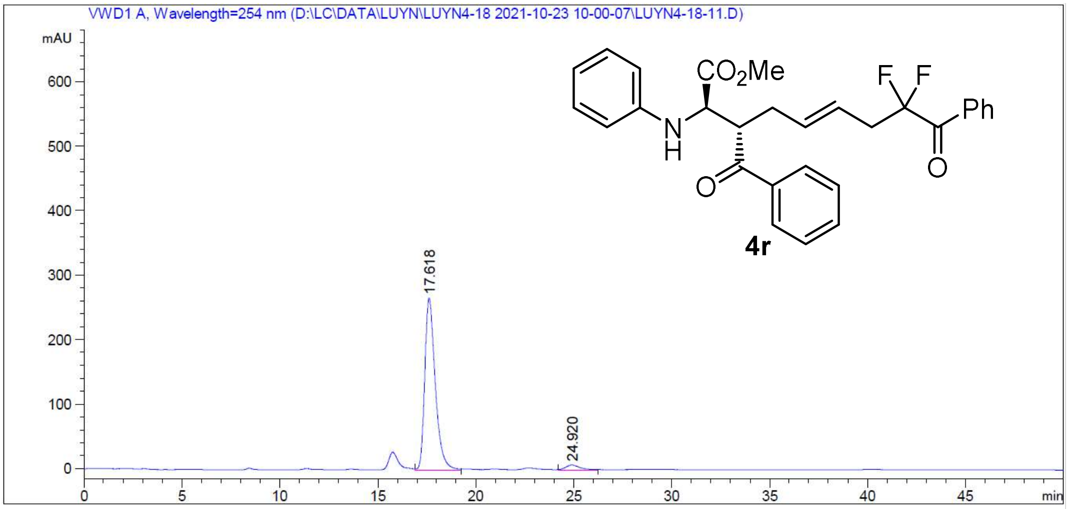

Peak #	RetTime [min]	Type	Width [min]	Area [mAU*s]	Height [mAU]	Area %
1	17.618	VB	0.5622	9909.00195	266.35208	96.4067
2	24.920	VV	0.5952	369.32916	7.38339	3.5933

HPLC chromatogram of compound (*rac*)-4s

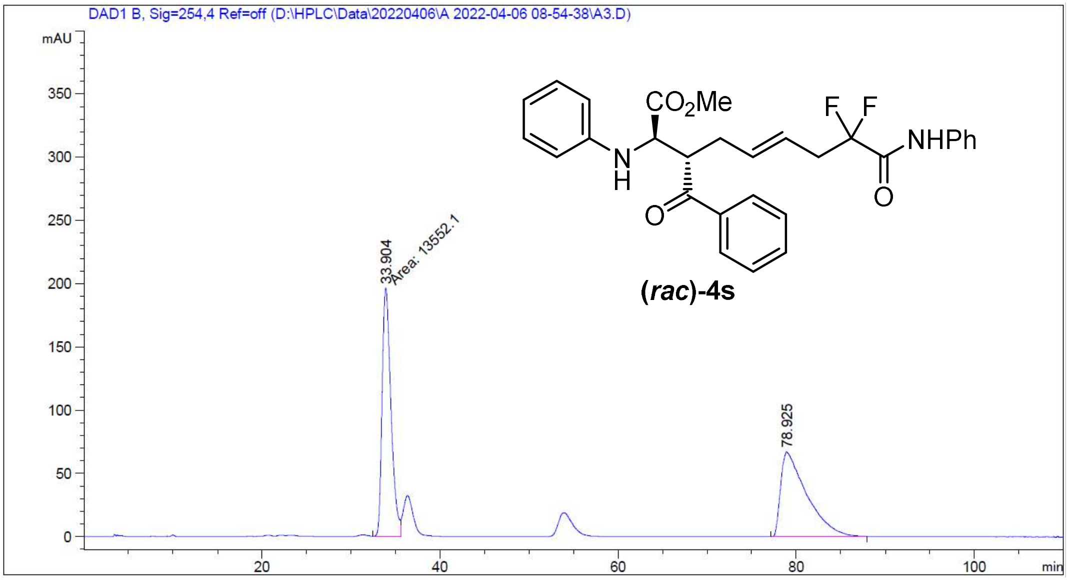

Peak #	RetTime [min]	Type	Width [min]	Area [mAU*s]	Height [mAU]	Area %
1	33.904	MF	1.1496	1.35521e4	196.47023	50.0064
2	78.925	BB	2.4174	1.35486e4	67.10788	49.9936

HPLC chromatogram of compound 4s

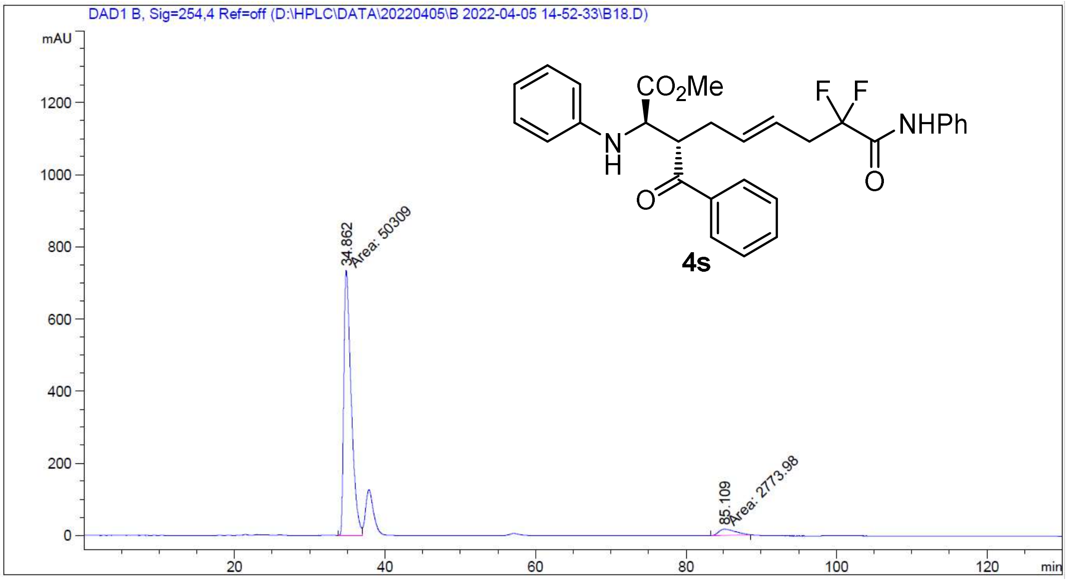

Peak #	RetTime [min]	Type	Width [min]	Area [mAU*s]	Height [mAU]	Area %
1	34.862	MF	1.1405	5.03090e4	735.21478	94.7743
2	85.109	MM	2.6064	2773.98071	17.73825	5.2257

HPLC chromatogram of compound (*rac*)-4t

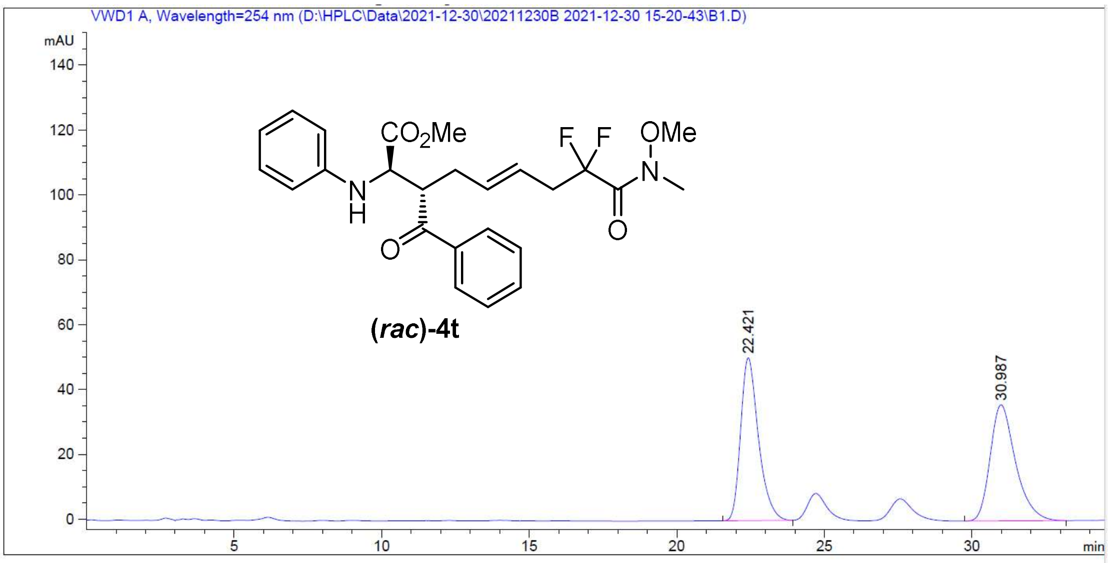

Peak #	RetTime [min]	Type	Width [min]	Area [mAU*s]	Height [mAU]	Area %
1	22.421	BB	0.6359	2112.48267	50.20748	49.6496
2	30.987	BB	0.8992	2142.29761	35.79738	50.3504

HPLC chromatogram of compound 4t

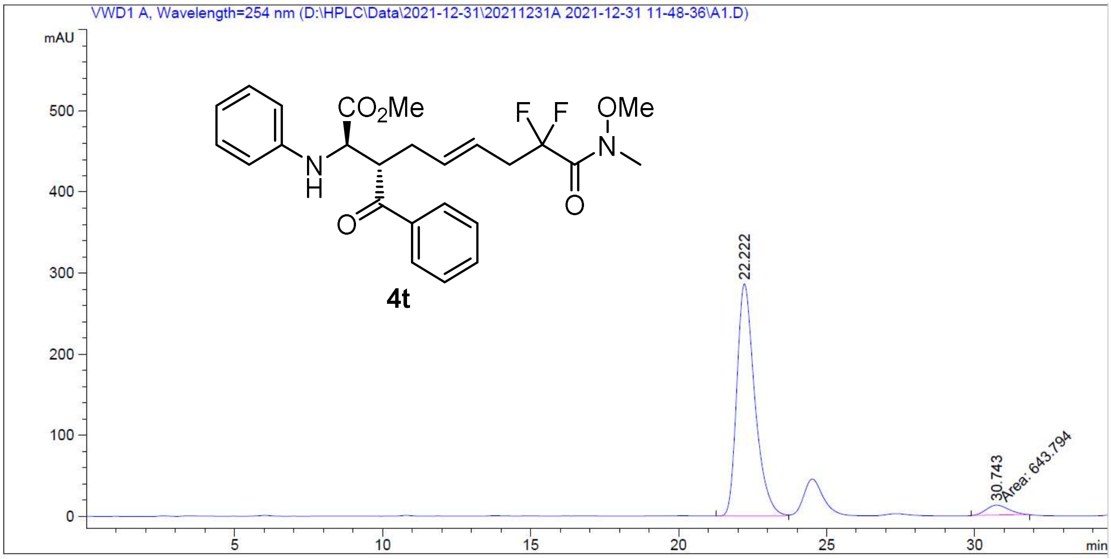

Peak #	RetTime [min]	Type	Width [min]	Area [mAU*s]	Height [mAU]	Area %
1	22.222	BV	0.6392	1.20941e4	286.06912	94.9458
2	30.743	MM	0.8792	643.79407	12.20429	5.0542

HPLC chromatogram of compound (*rac*)-4u

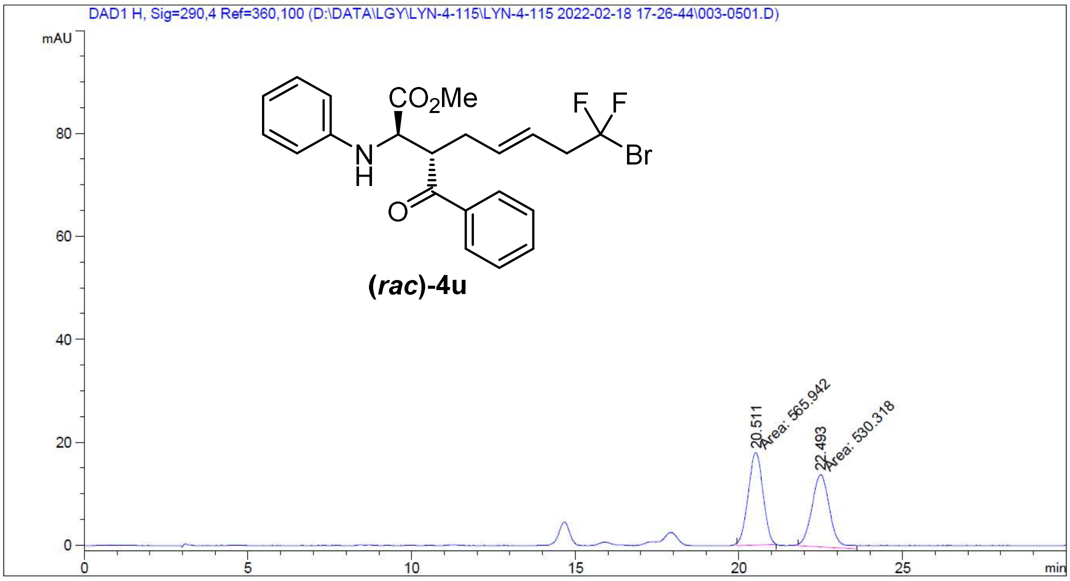

Peak #	RetTime [min]	Type	Width [min]	Area [mAU*s]	Height [mAU]	Area %
1	20.511	MP	0.5250	565.94208	17.96741	51.6248
2	22.493	MM T	0.6311	530.31824	14.00489	48.3752

HPLC chromatogram of compound 4u

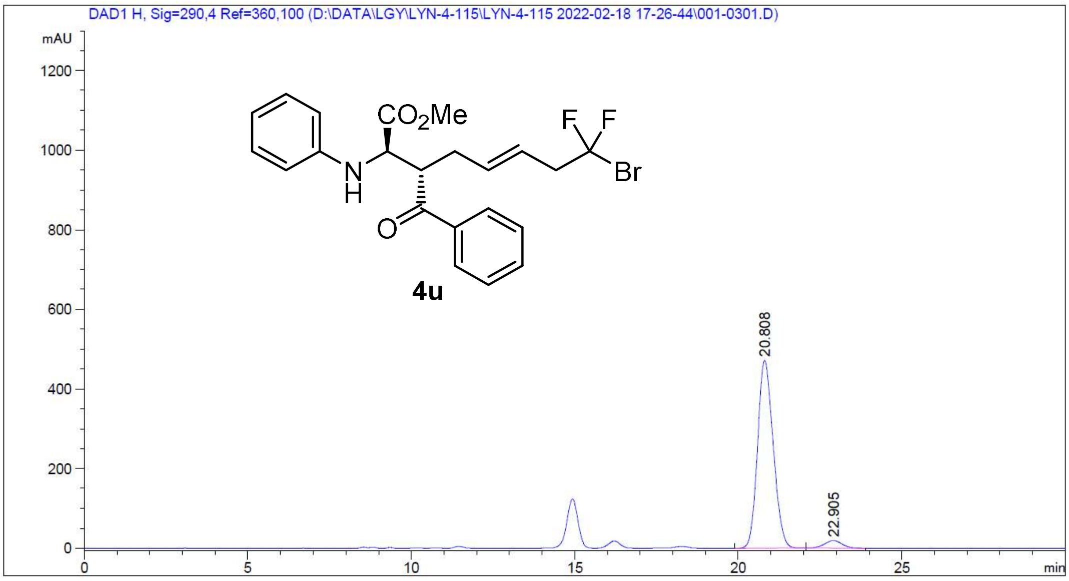

Peak #	RetTime [min]	Type	Width [min]	Area [mAU*s]	Height [mAU]	Area %
1	20.808	BB	0.5151	1.56183e4	470.77405	95.8233
2	22.905	BB	0.5686	680.76709	18.61929	4.1767

HPLC chromatogram of compound (*rac*)-4v

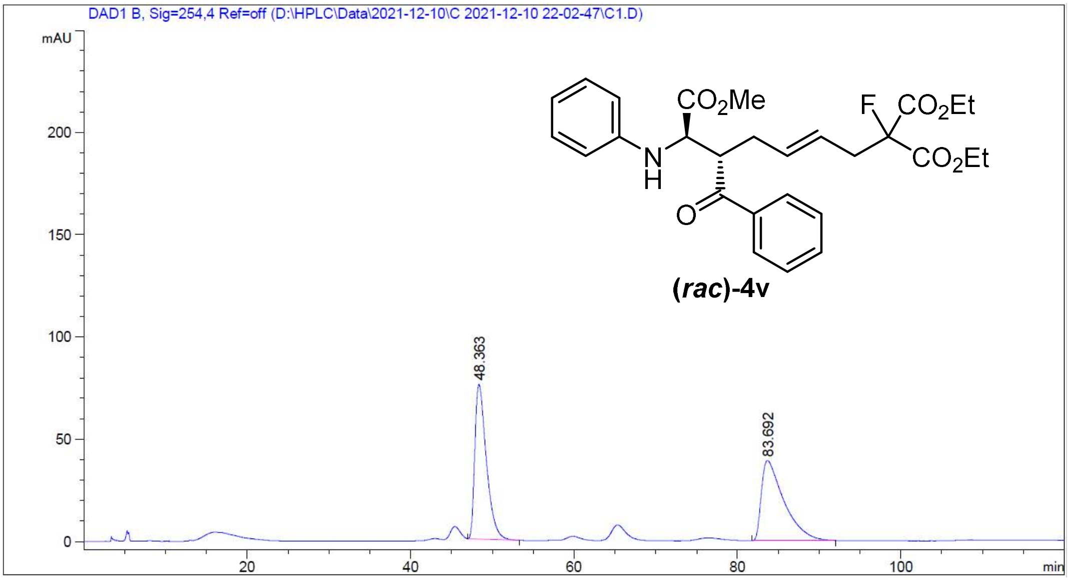

Peak #	RetTime [min]	Type	Width [min]	Area [mAU*s]	Height [mAU]	Area %
1	48.363	BB	1.3936	7430.94434	75.67799	49.7717
2	83.692	BB	2.4801	7499.12842	39.19666	50.2283

HPLC chromatogram of compound 4v

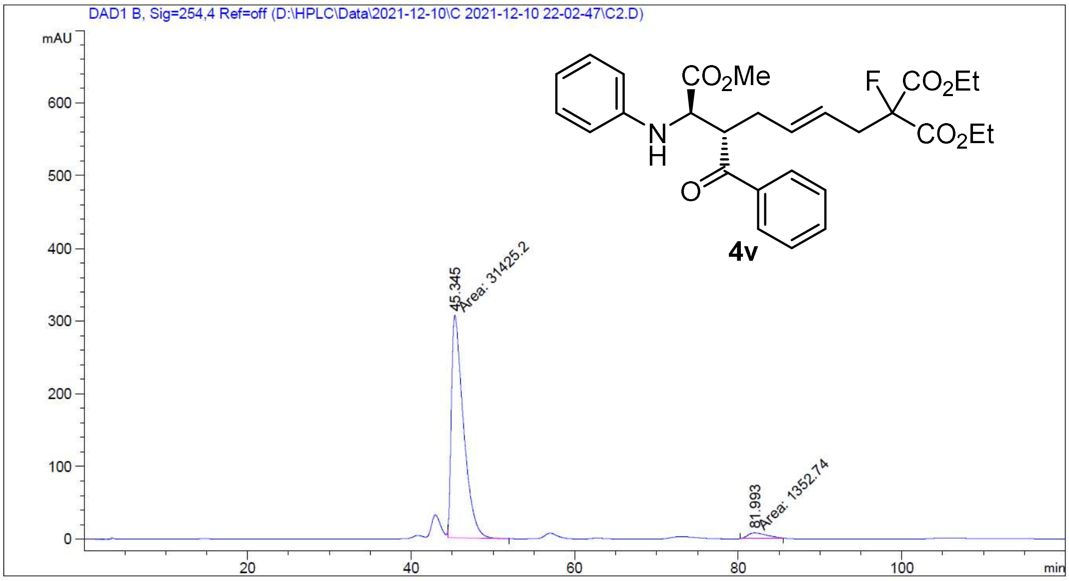

Peak #	RetTime [min]	Type	Width [min]	Area [mAU*s]	Height [mAU]	Area %
1	45.345	FM	1.7123	3.14252e4	305.88541	95.8730
2	81.993	MM	2.8400	1352.74487	7.93870	4.1270

HPLC chromatogram of compound (*rac*)-4v

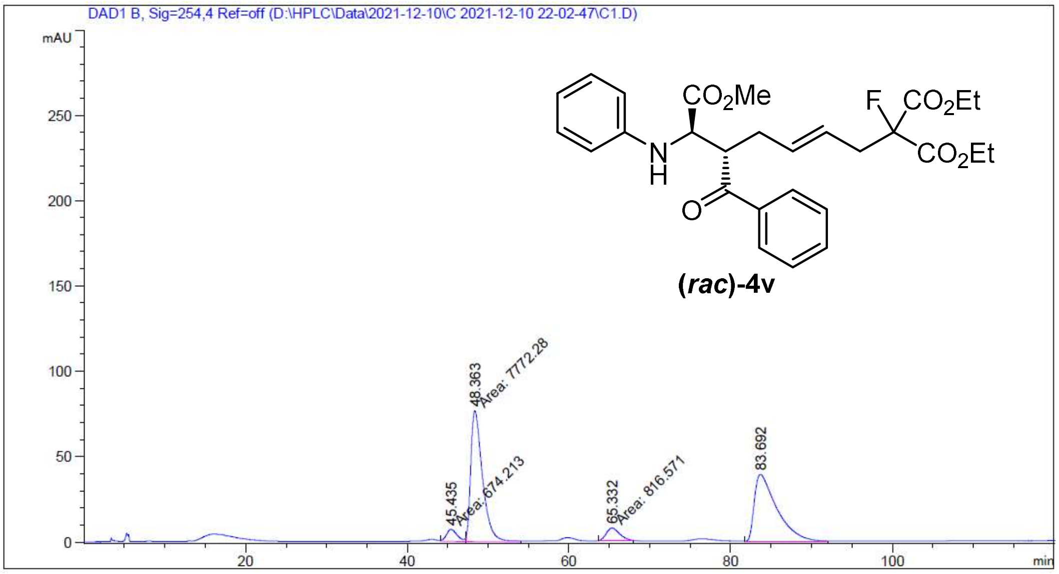

Peak #	RetTime [min]	Type	Width [min]	Area [mAU*s]	Height [mAU]	Area %
1	45.435	MF	1.5799	674.21277	7.11259	4.0222
2	48.363	FM	1.6884	7772.27539	76.72393	46.3679
3	65.332	PP	1.8720	816.57141	7.27017	4.8715
4	83.692	BB	2.4801	7499.12842	39.19666	44.7384

HPLC chromatogram of compound 4v

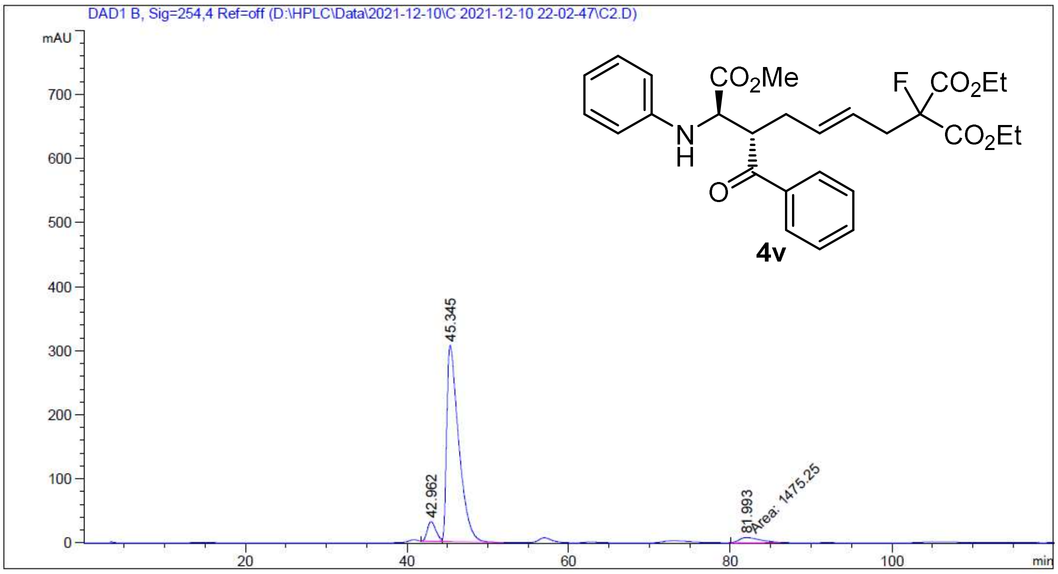

Peak #	RetTime [min]	Type	Width [min]	Area [mAU*s]	Height [mAU]	Area %
1	42.962	BV E	1.0802	2261.98340	30.75047	6.4187
2	45.345	VB R	1.4836	3.15035e4	305.88434	89.3951
3	81.993	MM	2.9576	1475.24951	8.31342	4.1862

HPLC chromatogram of compound (*rac*)-4w

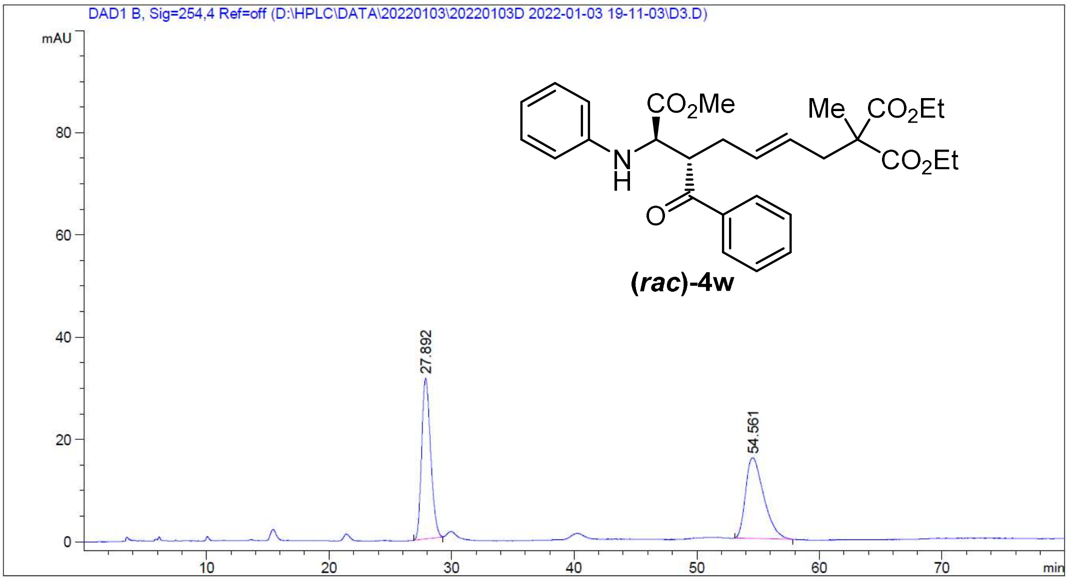

Peak #	RetTime [min]	Type	Width [min]	Area [mAU*s]	Height [mAU]	Area %
1	27.892	BB	0.6779	1600.65967	31.39158	49.6647
2	54.561	BB	1.2047	1622.27307	15.75007	50.3353

HPLC chromatogram of compound 4w

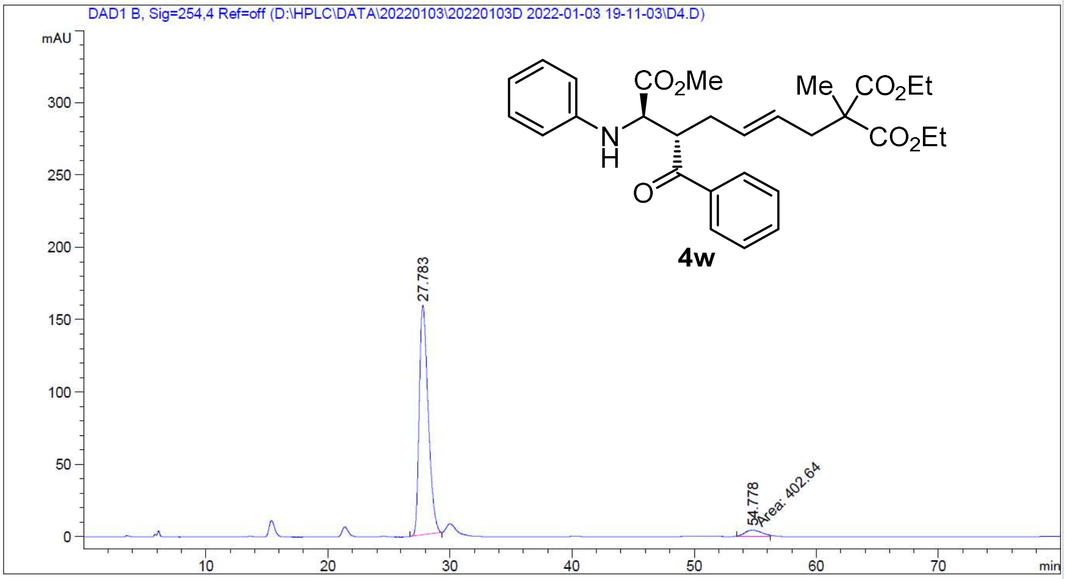

Peak #	RetTime [min]	Type	Width [min]	Area [mAU*s]	Height [mAU]	Area %
1	27.783	BB	0.7540	8087.80273	158.58018	95.2577
2	54.778	MM	1.5412	402.64029	4.35430	4.7423

HPLC chromatogram of compound (*rac*)-4w

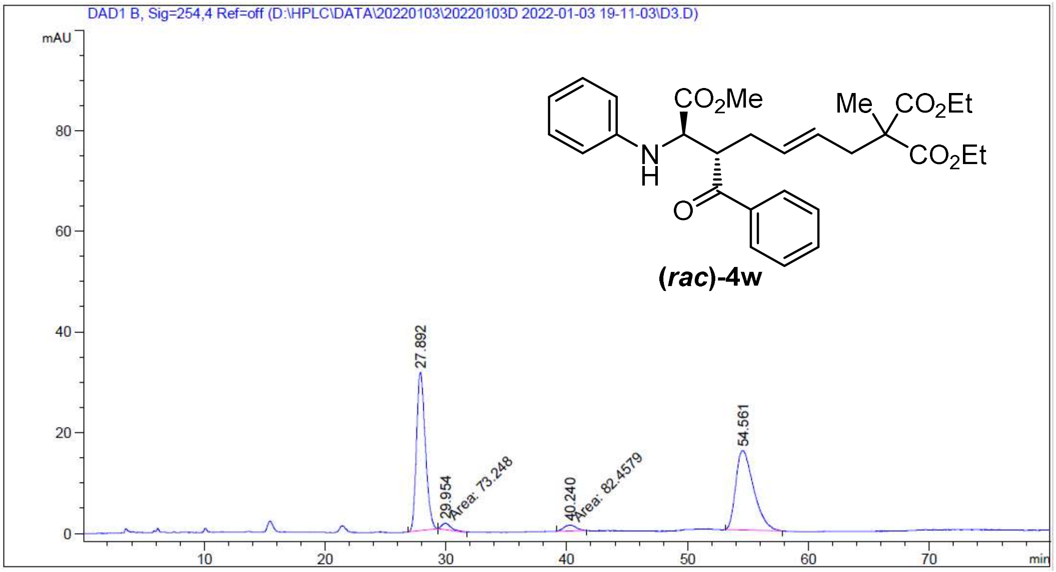

Peak #	RetTime [min]	Type	Width [min]	Area [mAU*s]	Height [mAU]	Area %
1	27.892	BB	0.6779	1600.65967	31.39158	47.3759
2	29.954	MM T	0.9291	73.24799	1.31389	2.1680
3	40.240	MP	1.1863	82.45789	1.15847	2.4406
4	54.561	BB	1.2047	1622.27307	15.75007	48.0156

HPLC chromatogram of compound 4w

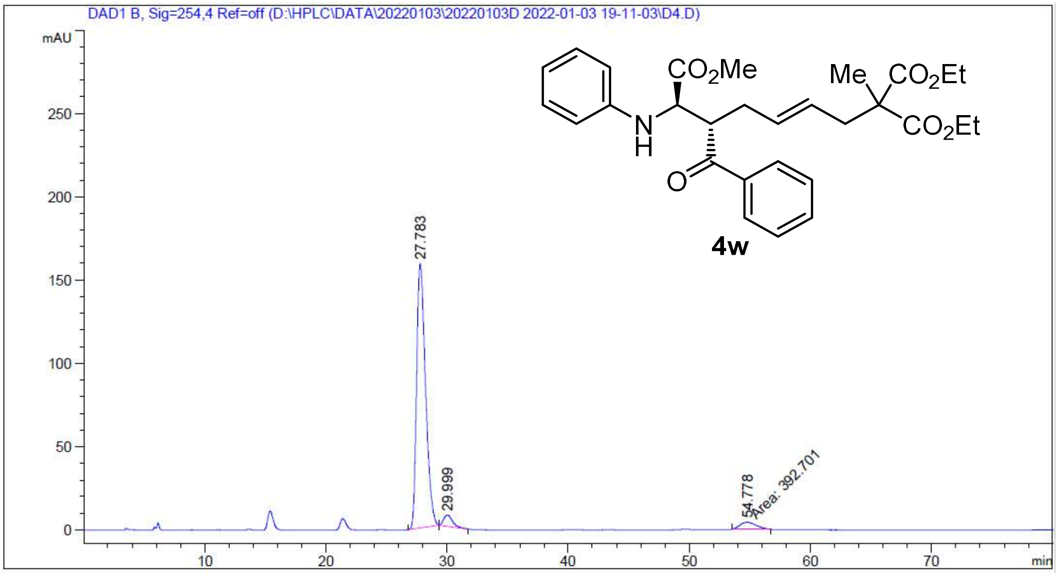

Peak #	RetTime [min]	Type	Width [min]	Area [mAU*s]	Height [mAU]	Area %
1	27.783	BB	0.7540	8087.80273	158.58018	91.6927
2	29.999	BB	0.6038	340.04498	6.63671	3.8551
3	54.778	PP	1.5397	392.70148	4.25078	4.4521

HPLC chromatogram of compound (*rac*)-4x

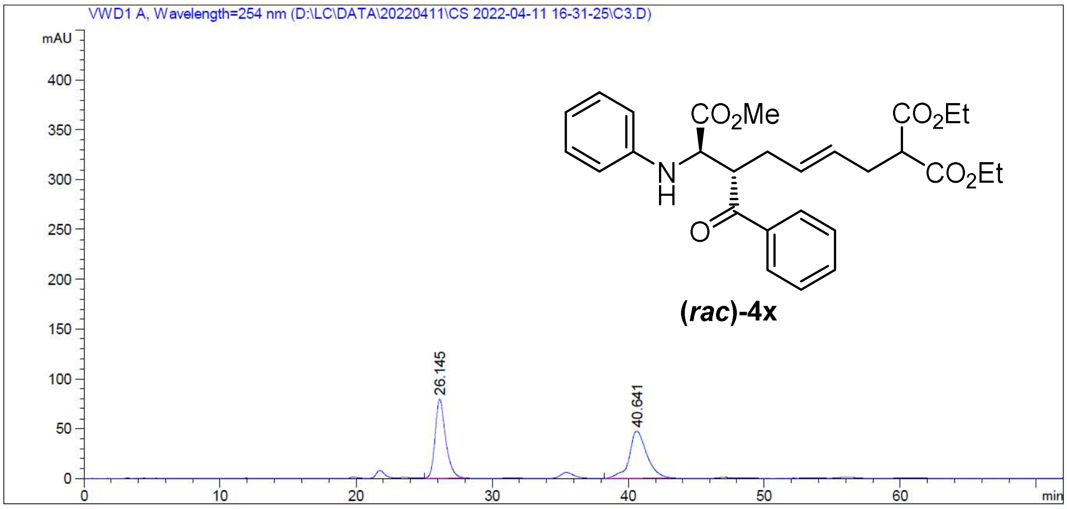

Peak #	RetTime [min]	Type	Width [min]	Area [mAU*s]	Height [mAU]	Area %
1	26.145	VB	0.7917	4239.96582	79.42778	50.2125
2	40.641	BB	1.1078	4204.08447	47.12460	49.7875

HPLC chromatogram of compound 4x

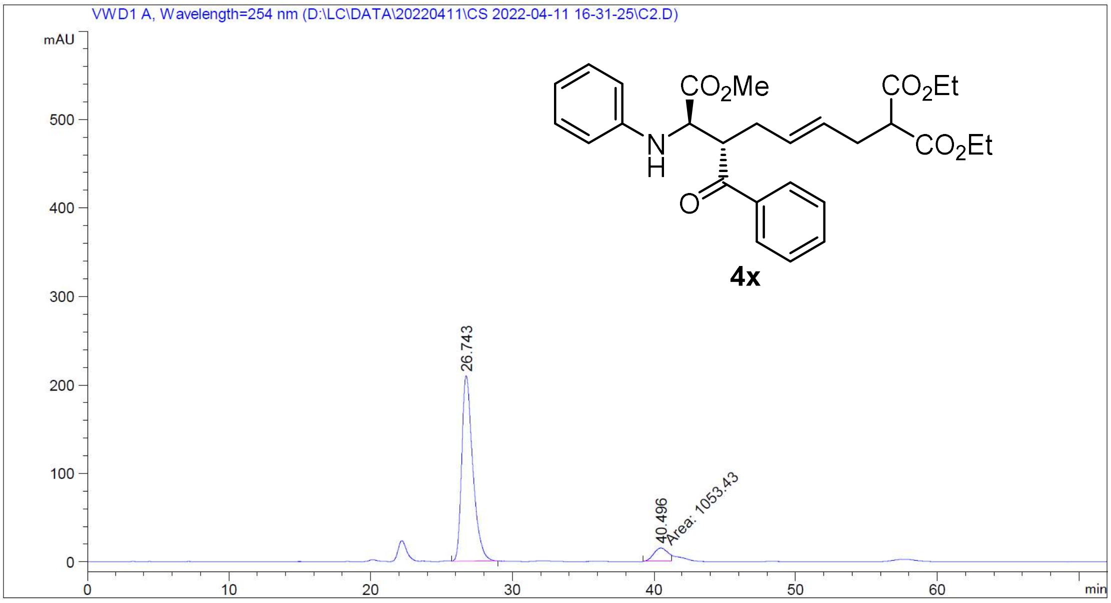

Peak #	RetTime [min]	Type	Width [min]	Area [mAU*s]	Height [mAU]	Area %
1	26.743	VB	0.8139	1.14442e4	209.82944	91.5709
2	40.496	MF	1.1625	1053.43127	15.10245	8.4291

HPLC chromatogram of compound (*rac*)-4x

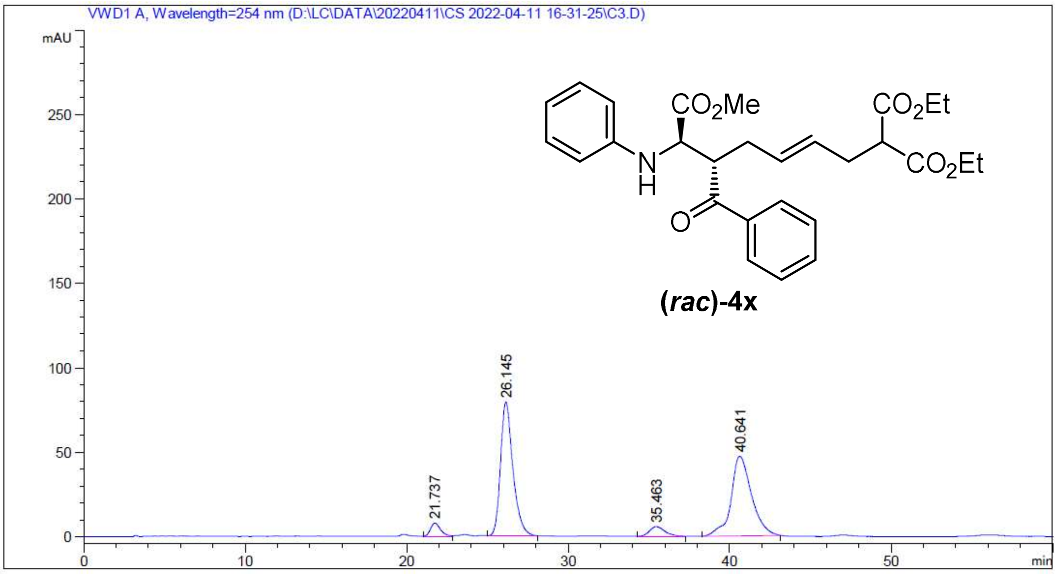

Peak #	RetTime [min]	Type	Width [min]	Area [mAU*s]	Height [mAU]	Area %
1	21.737	BV	0.5207	334.93414	7.85595	3.6534
2	26.145	VB	0.7917	4239.96582	79.42778	46.2484
3	35.463	BB	0.7990	388.81754	5.79358	4.2411
4	40.641	BB	1.1078	4204.08447	47.12460	45.8571

HPLC chromatogram of compound 4x

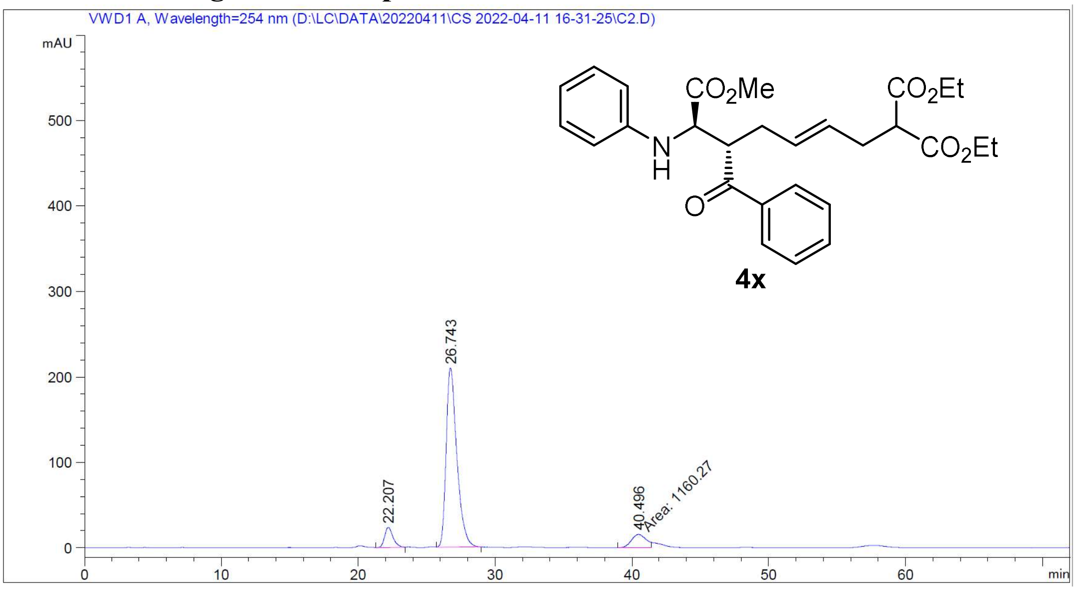

Peak #	RetTime [min]	Type	Width [min]	Area [mAU*s]	Height [mAU]	Area %
1	22.207	VV	0.6182	996.90967	23.52100	7.3295
2	26.743	VB	0.8139	1.14442e4	209.82944	84.1399
3	40.496	MF	1.2493	1160.27380	15.47860	8.5306

HPLC chromatogram of compound (*rac*)-4y

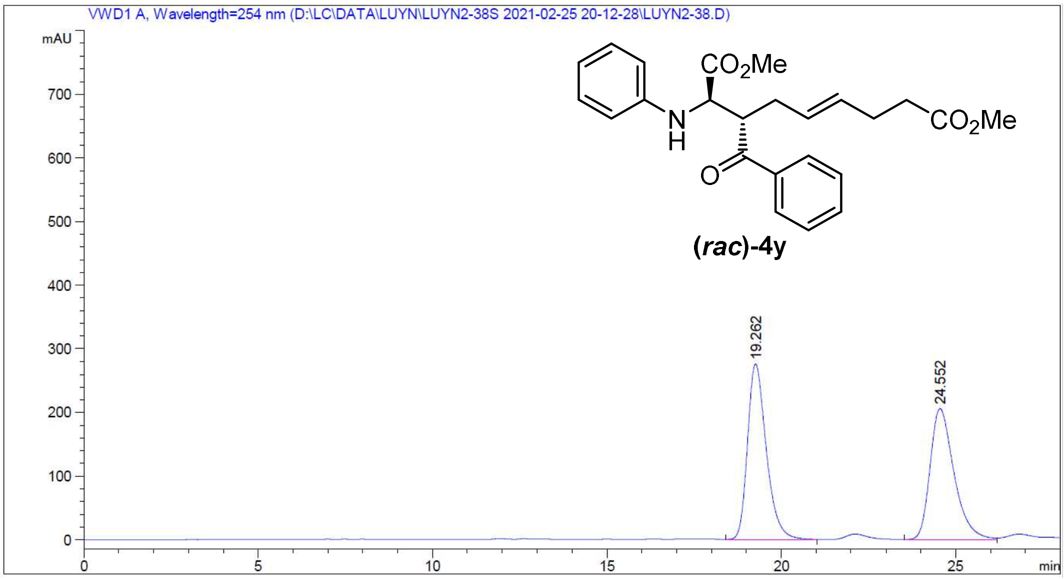

Peak #	RetTime [min]	Type	Width [min]	Area [mAU*s]	Height [mAU]	Area %
1	19.262	VB	0.5749	1.03255e4	275.90378	51.3087
2	24.552	VB	0.7234	9798.82813	205.61108	48.6913

HPLC chromatogram of compound 4y

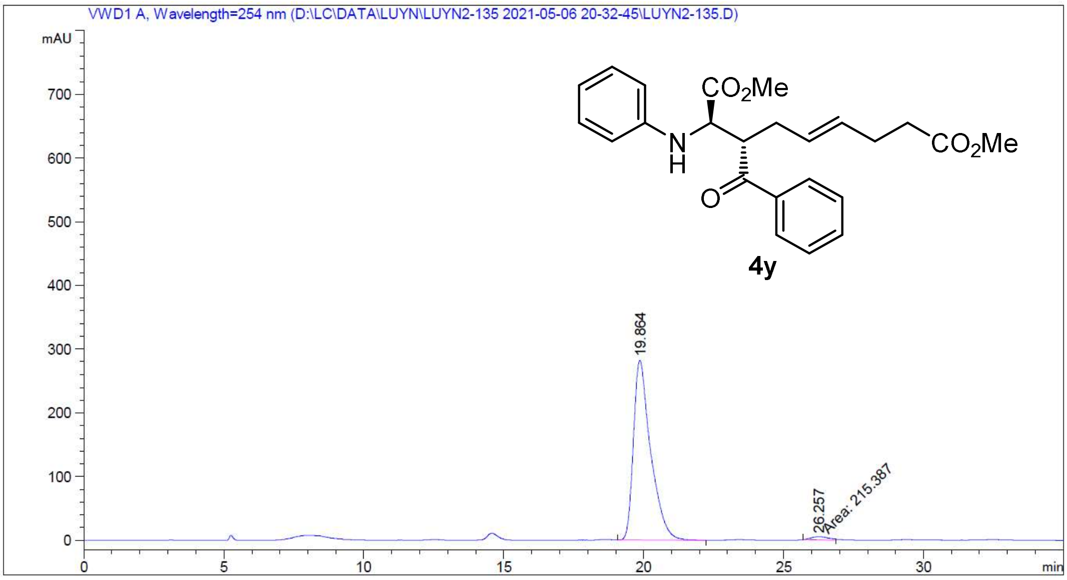

Peak #	RetTime [min]	Type	Width [min]	Area [mAU*s]	Height [mAU]	Area %
1	19.864	BV R	0.6292	1.20388e4	281.97229	98.2423
2	26.257	PM	0.7110	215.38667	5.04913	1.7577

HPLC chromatogram of compound (*rac*)-6

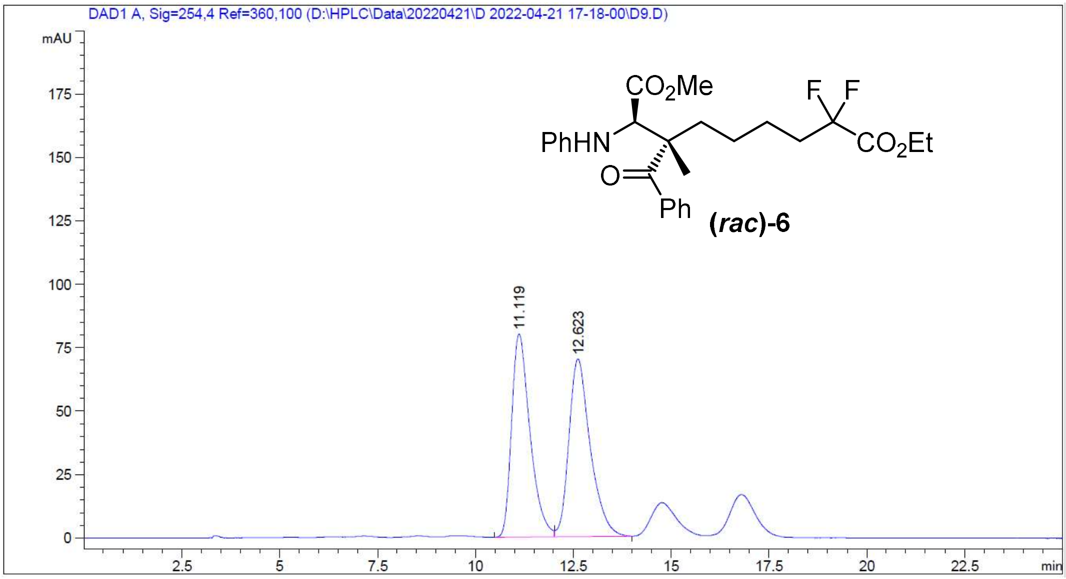

Peak #	RetTime [min]	Type	Width [min]	Area [mAU*s]	Height [mAU]	Area %
1	11.119	BV	0.4817	2618.62500	80.03069	49.5344
2	12.623	VB	0.5574	2667.85742	70.03143	50.4656

HPLC chromatogram of compound 6

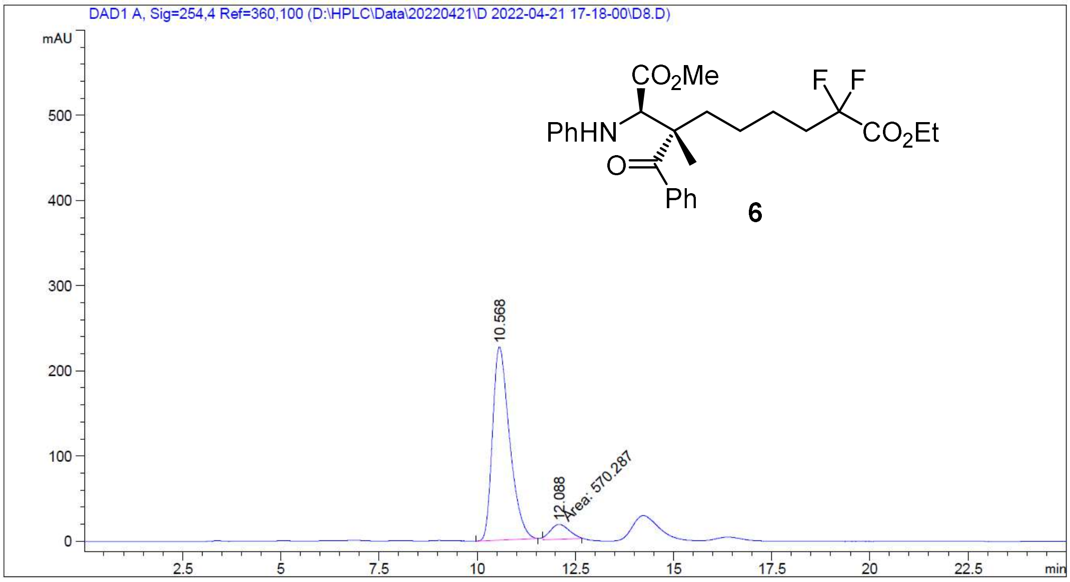

Peak #	RetTime [min]	Type	Width [min]	Area [mAU*s]	Height [mAU]	Area %
1	10.568	BB	0.4520	6864.65674	226.85907	92.3296
2	12.088	MM	0.5495	570.28680	17.29667	7.6704

HPLC chromatogram of compound 10

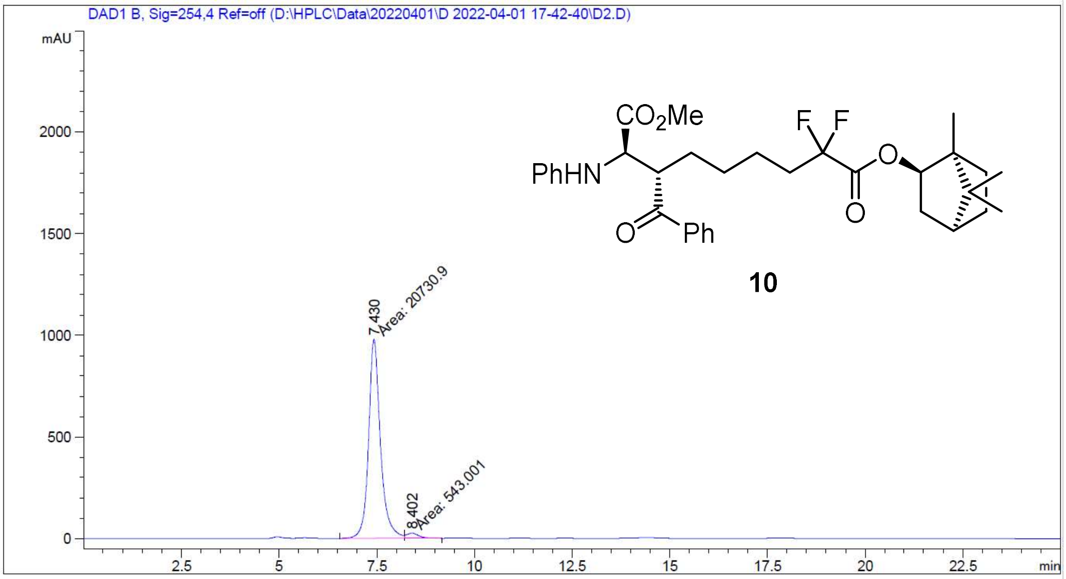

Peak #	RetTime [min]	Type	Width [min]	Area [mAU*s]	Height [mAU]	Area %
1	7.430	MF	0.3531	2.07309e4	978.57361	97.4476
2	8.402	FM	0.3534	543.00128	25.60876	2.5524

HPLC chromatogram of compound 10’

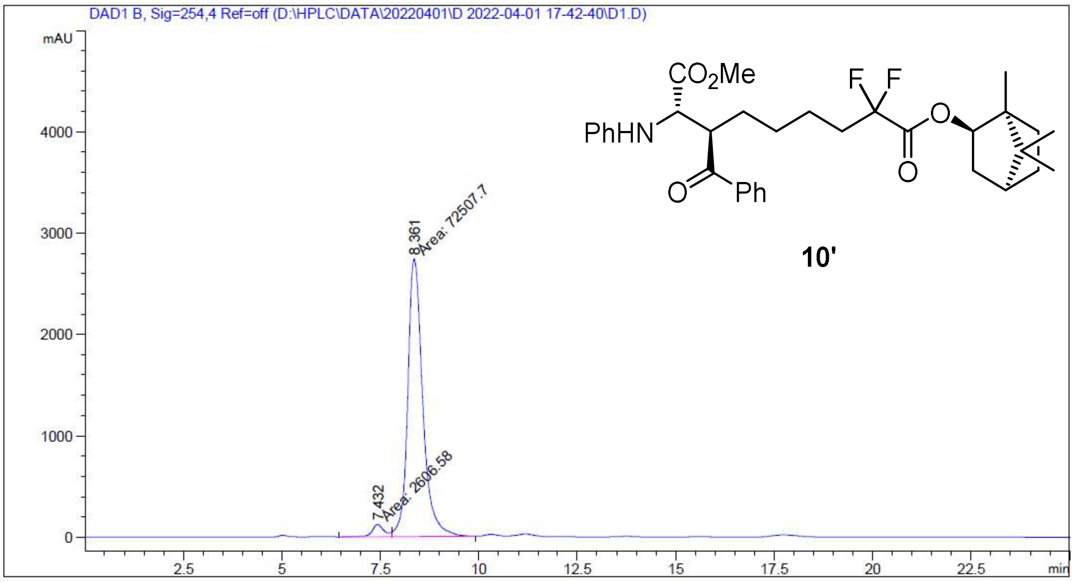

Peak #	RetTime [min]	Type	Width [min]	Area [mAU*s]	Height [mAU]	Area %
1	7.432	MF	0.3584	2606.57837	121.21859	3.4702
2	8.361	FM	0.4409	7.25077e4	2740.76025	96.5298

HPLC chromatogram of compound 11

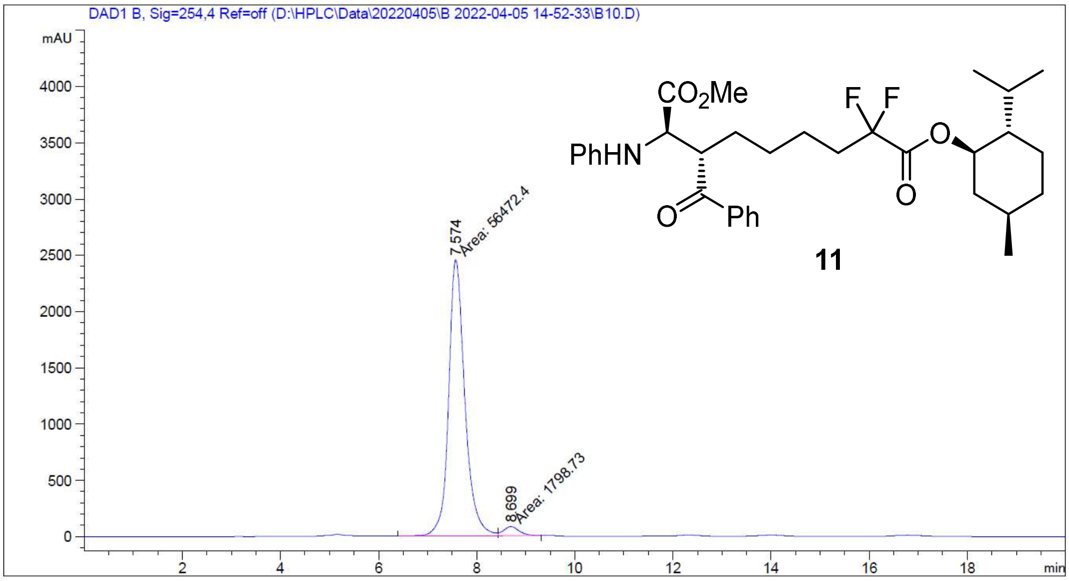

Peak #	RetTime [min]	Type	Width [min]	Area [mAU*s]	Height [mAU]	Area %
1	7.574	MF	0.3838	5.64724e4	2452.27588	96.9132
2	8.699	FM	0.3694	1798.73169	81.16481	3.0868

HPLC chromatogram of compound 11’

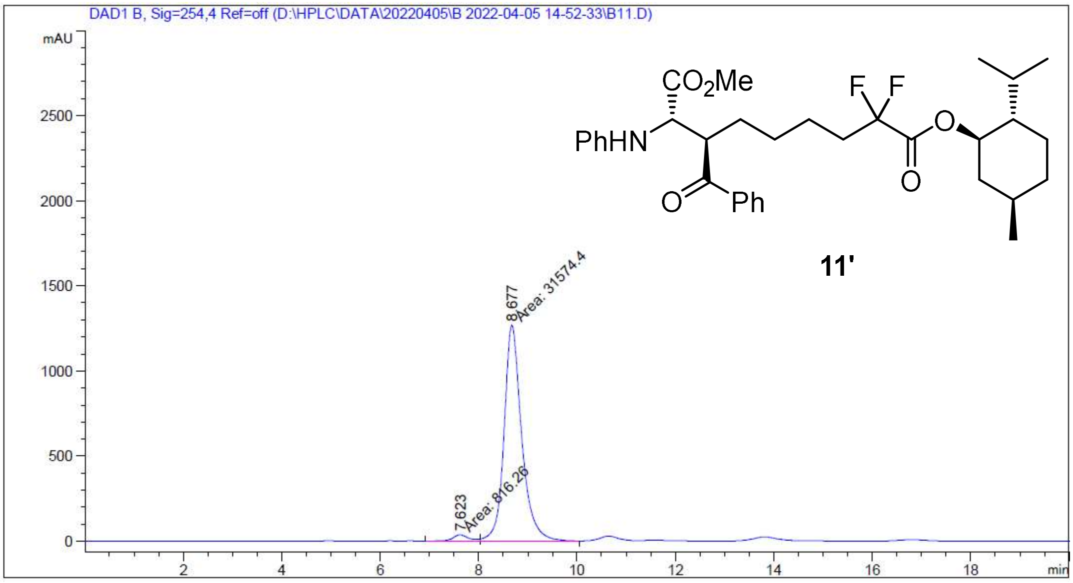

Peak #	RetTime [min]	Type	Width [min]	Area [mAU*s]	Height [mAU]	Area %
1	7.623	MF	0.3749	816.25983	36.28506	2.5201
2	8.677	FM	0.4154	3.15744e4	1266.86816	97.4799

HPLC chromatogram of compound 12

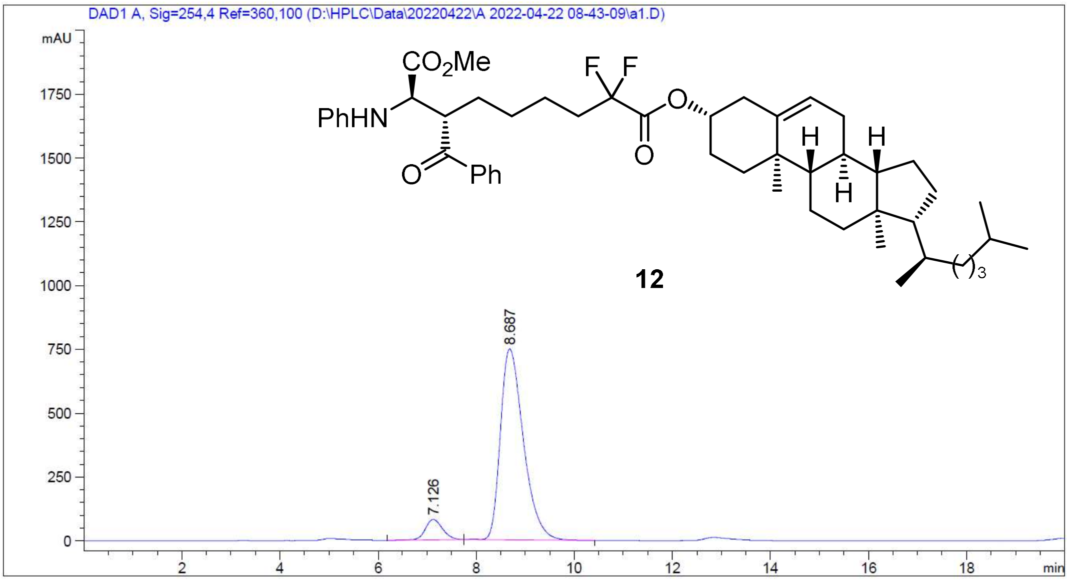

Peak #	RetTime [min]	Type	Width [min]	Area [mAU*s]	Height [mAU]	Area %
1	7.126	BB	0.3757	1937.30505	79.96374	7.3647
2	8.687	VB R	0.5050	2.43679e4	747.59918	92.6353

HPLC chromatogram of compound 12’

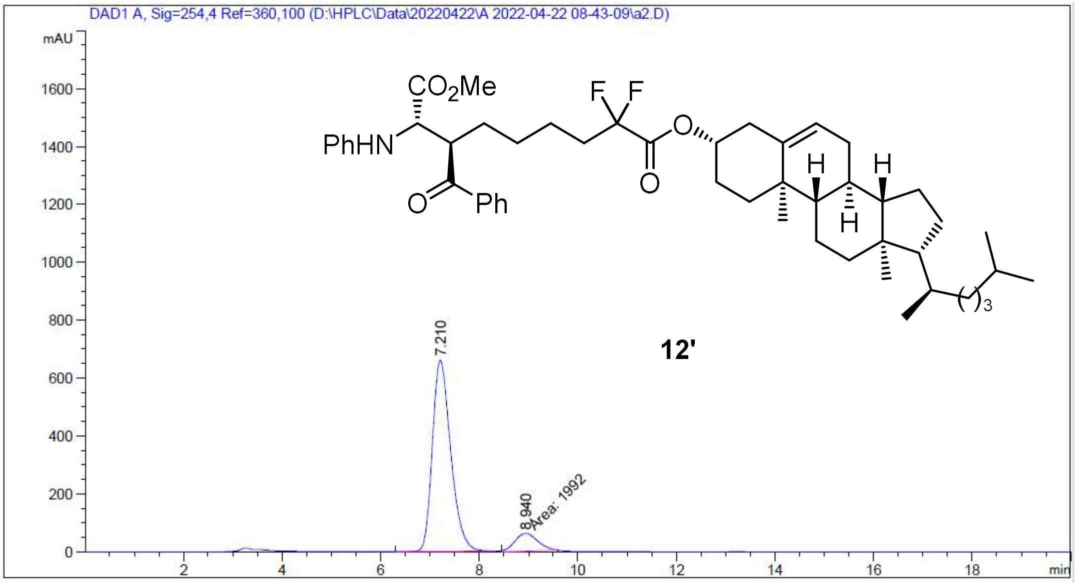

Peak #	RetTime [min]	Type	Width [min]	Area [mAU*s]	Height [mAU]	Area %
1	7.210	BB	0.3973	1.70956e4	659.67053	89.5639
2	8.940	MP	0.5386	1992.00159	61.63934	10.4361

HPLC chromatogram of compound (*rac*)-13

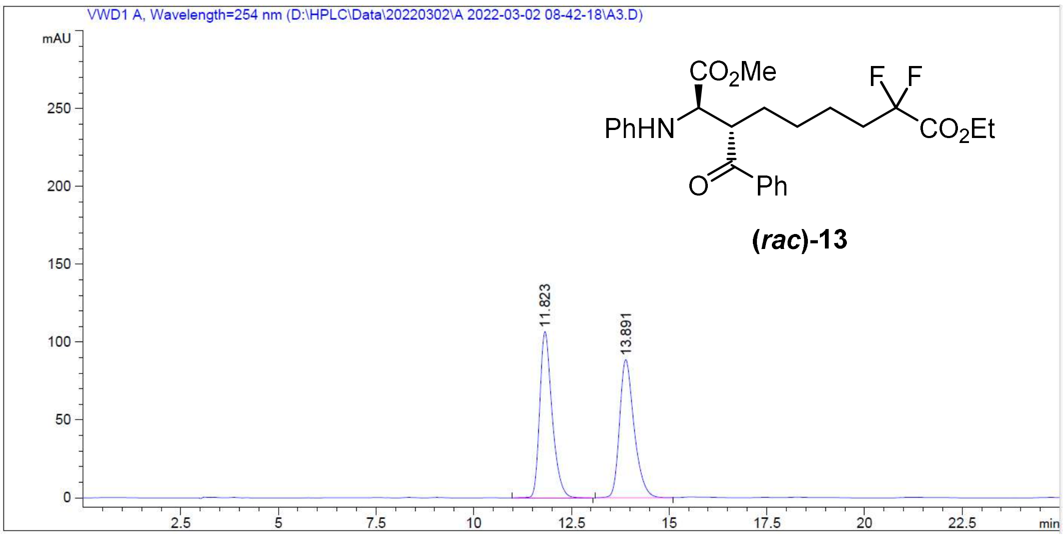

Peak #	RetTime [min]	Type	Width [min]	Area [mAU*s]	Height [mAU]	Area %
1	11.823	BB	0.3326	2348.12793	106.77174	50.5308
2	13.891	BB	0.3904	2298.79297	88.68778	49.4692

HPLC chromatogram of compound 13

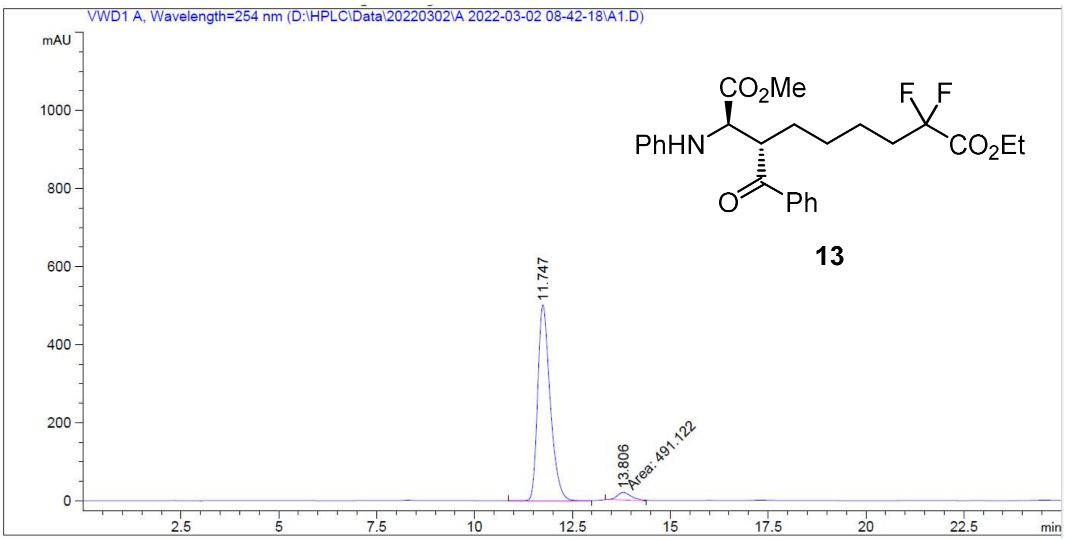

Peak #	RetTime [min]	Type	Width [min]	Area [mAU*s]	Height [mAU]	Area %
1	11.747	BB	0.3340	1.10979e4	501.79736	95.7622
2	13.806	MM	0.4226	491.12192	19.36883	4.2378

HPLC chromatogram of compound 14

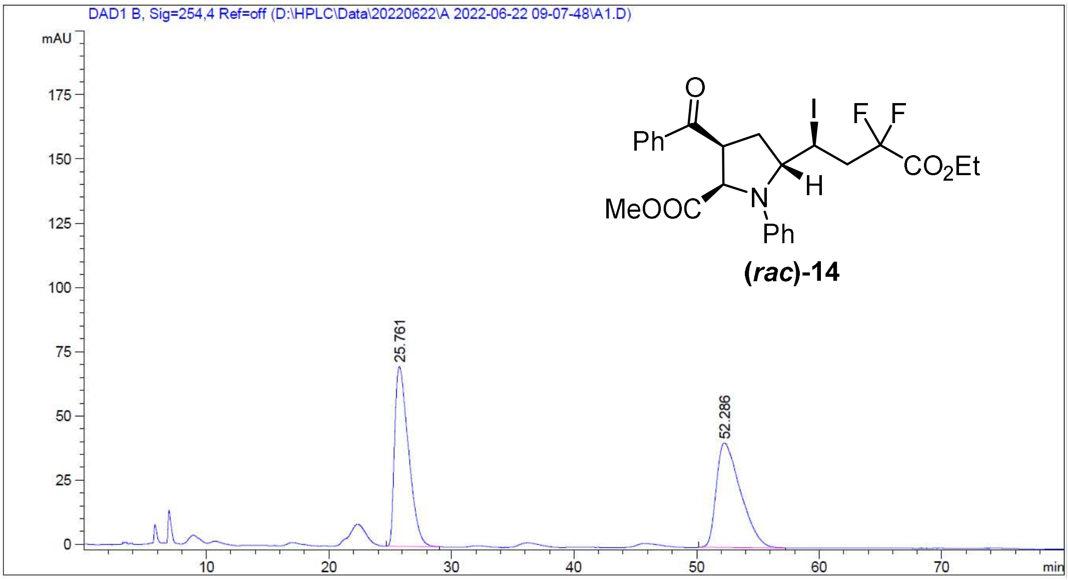

Peak #	RetTime [min]	Type	Width [min]	Area [mAU*s]	Height [mAU]	Area %
1	25.761	BB	1.1748	5505.76660	69.91373	49.6141
2	52.286	BB	1.8179	5591.41846	40.72212	50.3859

HPLC chromatogram of compound 14

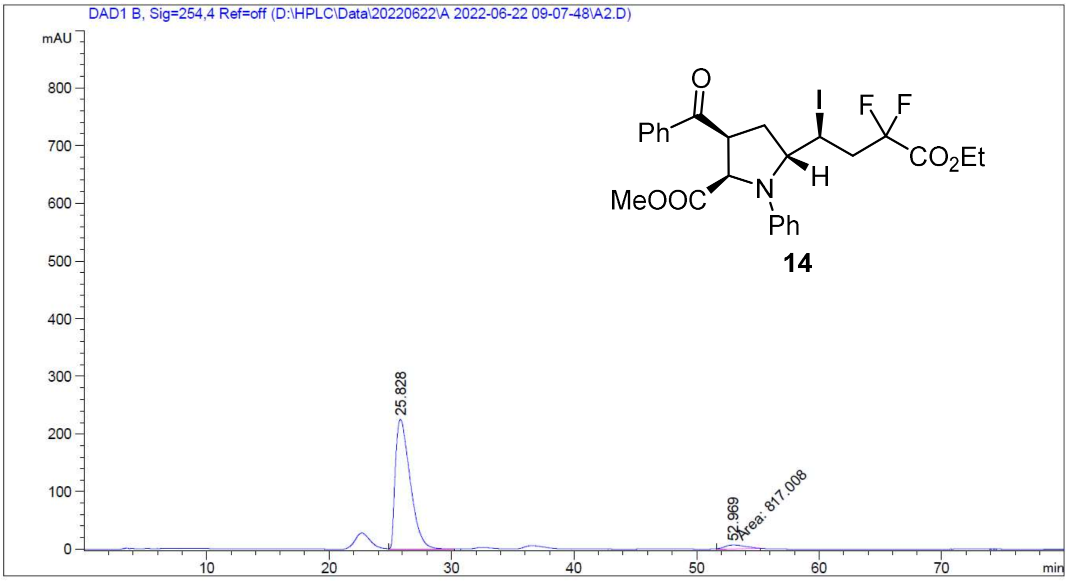

Peak #	RetTime [min]	Type	Width [min]	Area [mAU*s]	Height [mAU]	Area %
1	25.828	BB	1.2461	1.87911e4	224.14488	95.8333
2	52.969	MM	1.4033	817.00751	6.85611	4.1667

HPLC chromatogram of compound (*rac*)-15

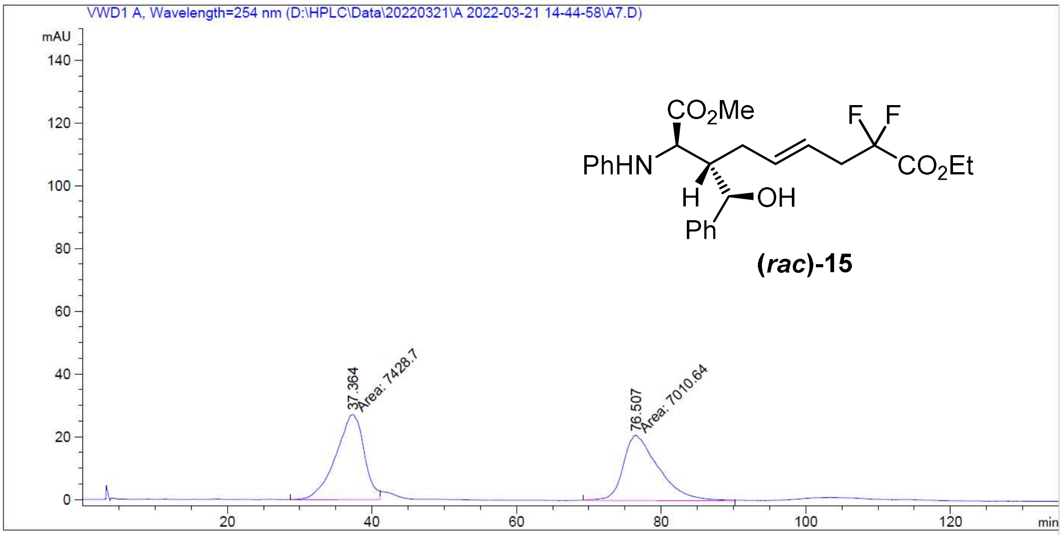

Peak #	RetTime [min]	Type	Width [min]	Area [mAU*s]	Height [mAU]	Area %
1	37.364	MF T	4.5623	7428.70215	27.13804	51.4477
2	76.507	MM	5.6571	7010.63672	20.65429	48.5523

HPLC chromatogram of compound 15

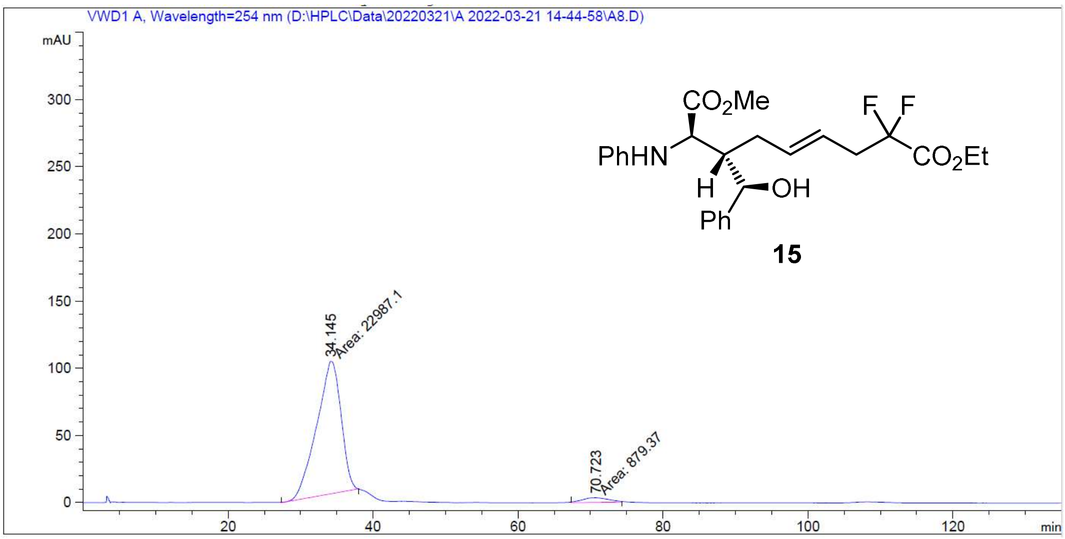

Peak #	RetTime [min]	Type	Width [min]	Area [mAU*s]	Height [mAU]	Area %
1	34.145	MM T	3.8873	2.29871e4	98.55592	96.3155
2	70.723	MM	4.2293	879.37024	3.46537	3.6845

HPLC chromatogram of compound (*rac*)-16

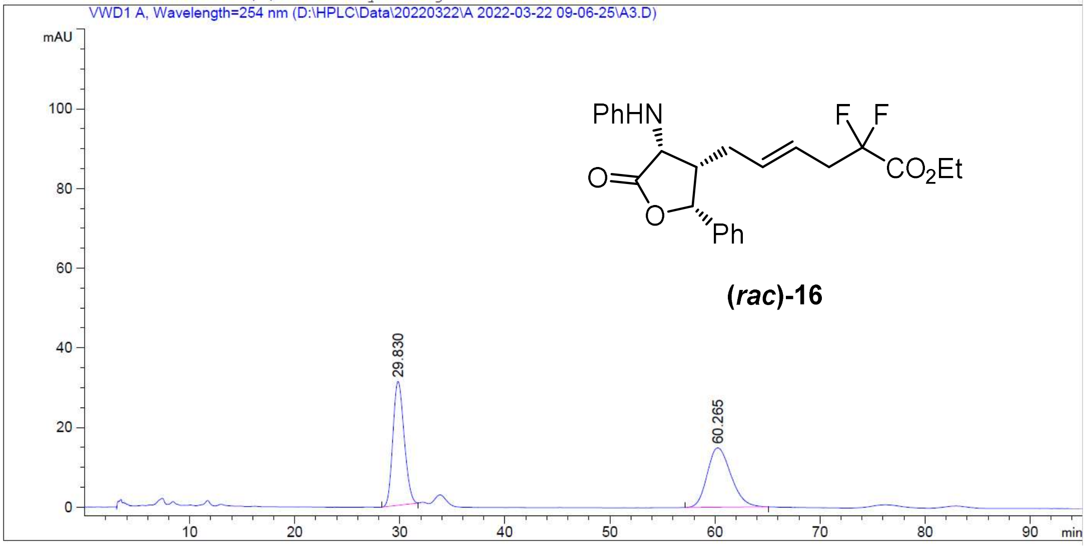

Peak #	RetTime [min]	Type	Width [min]	Area [mAU*s]	Height [mAU]	Area %
1	29.830	BB	1.1257	2317.48120	31.08977	49.7240
2	60.265	BB	1.9082	2343.21240	14.89647	50.2760

HPLC chromatogram of compound 16

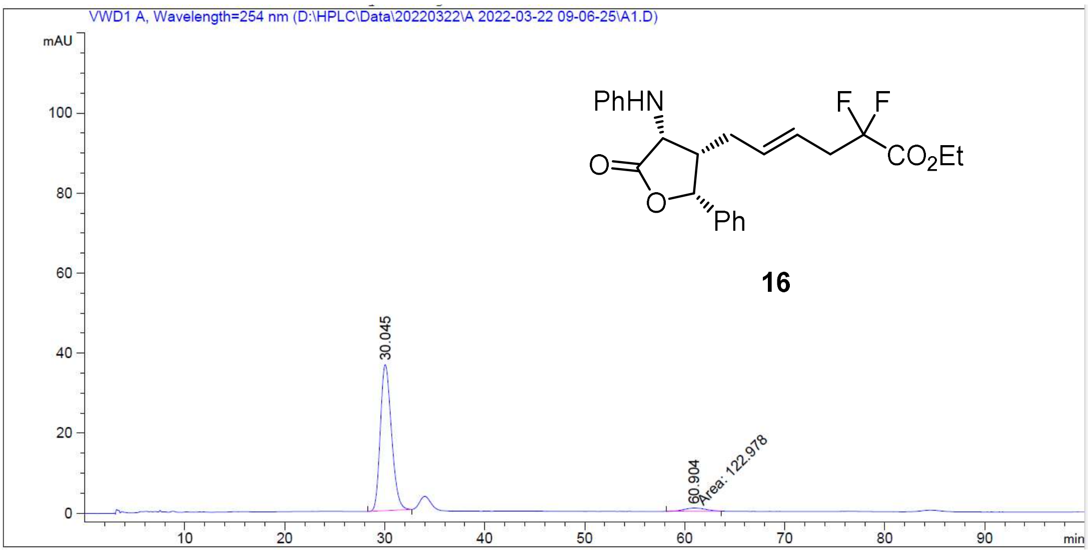

Peak #	RetTime [min]	Type	Width [min]	Area [mAU*s]	Height [mAU]	Area %
1	30.045	BB	1.1827	2827.42310	36.46286	95.8318
2	60.904	MM	2.5019	122.97763	8.19224e-1	4.1682

HPLC chromatogram of compound 17

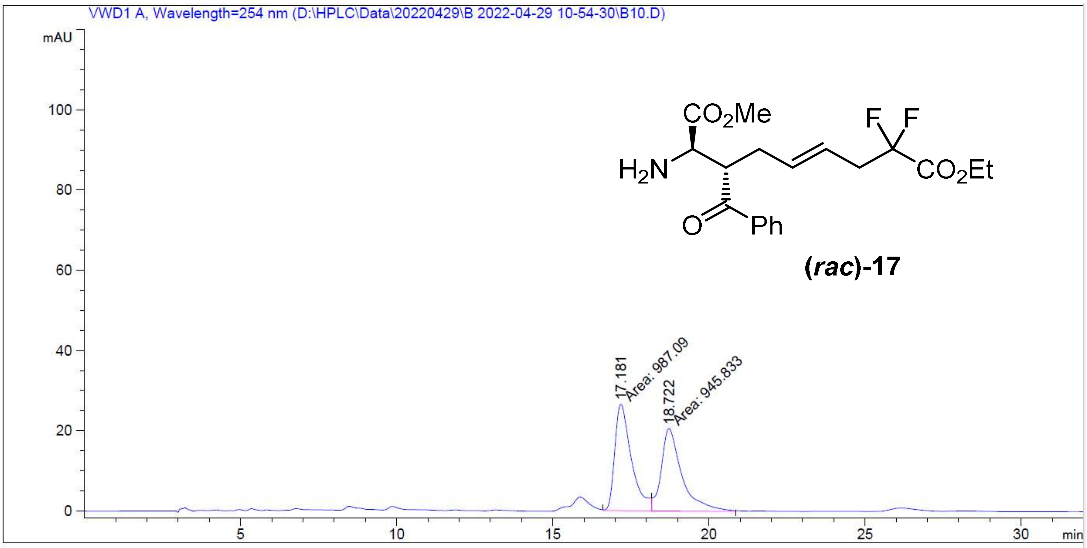

Peak #	RetTime [min]	Type	Width [min]	Area [mAU*s]	Height [mAU]	Area %
1	17.181	MF	0.6203	987.08997	26.51976	51.0672
2	18.722	FM	0.7684	945.83282	20.51599	48.9328

HPLC chromatogram of compound 17

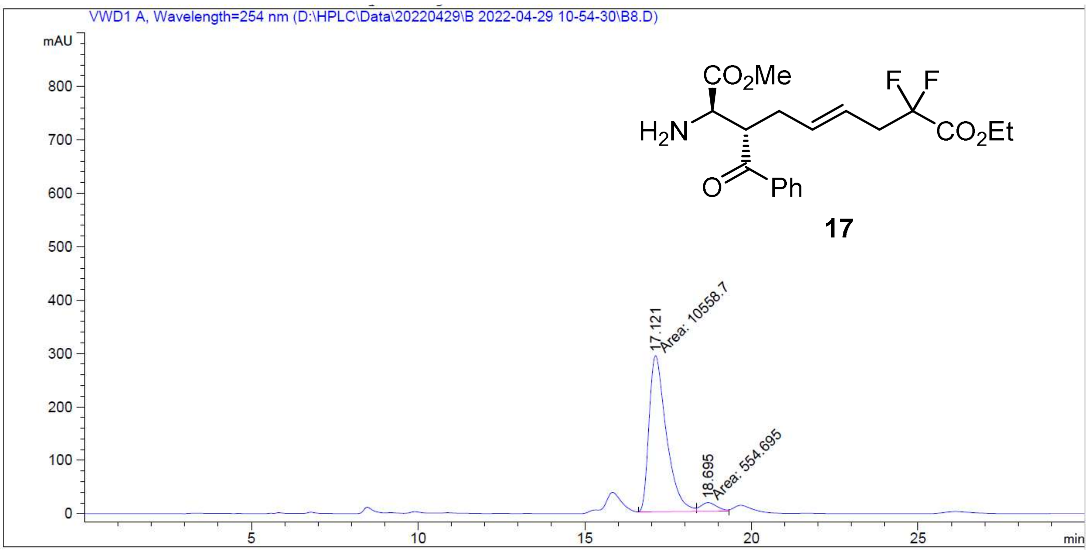

Peak #	RetTime [min]	Type	Width [min]	Area [mAU*s]	Height [mAU]	Area %
1	17.121	MF	0.6021	1.05587e4	292.29556	95.0088
2	18.695	FM	0.5660	554.69470	16.33274	4.9912

XI. Computational details

Computational methods

All density functional theory (DFT) calculations were performed using the Gaussian 16 software package.[1] Geometries were optimized using the M06-2X[2] functional with a basis set of 6-31G(d) for all atoms. Vibrational frequencies were calculated for all the stationary points to confirm if each optimized structure is a local minimum on the respective potential energy surface or a transition state structure with only one imaginary frequency. Solvation energy corrections were calculated in acetonitrile solvent with the SMD continuum solvation model[3] based on the gas phase optimized geometries. The M06-2X functional with a basis set of 6-311+G(d, p) for all atoms were used for single-point energy calculations. The chiral phosphoric acid (*R*)-**C1** is used in DFT calculation. The optimized transition state structures were plotted using CYLview.[4]

Additional computational results

The full free energy profile for the formation of α-carbonyl radical is shown in Figure S1. Both the radical addition to alkene (via **TS-1**) and ring-opening (via **TS-2**) processes are highly exergonic and irreversible. It should be noted that in the ring-opening step, the generation of *E*-dominated internal olefin moiety (via **TS-2**) is both kinetically and thermodynamically favored than that of *Z*-dominated internal olefin moiety (via **TS-2′**).

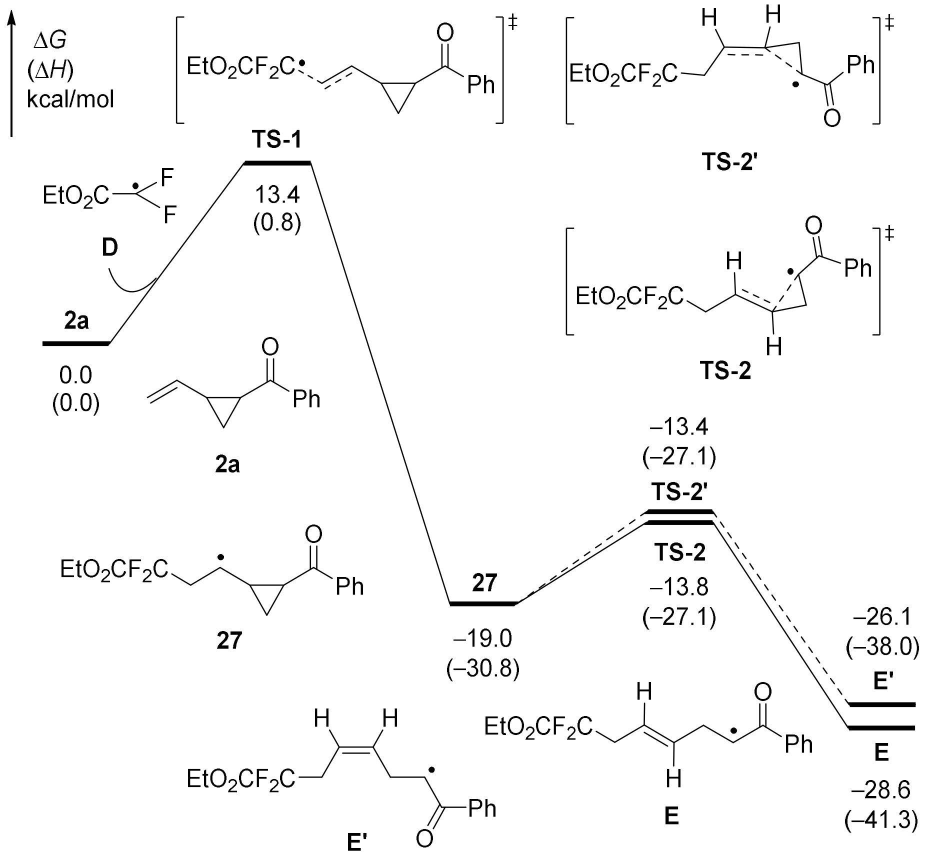

Figure S1. Free energy profile for the formation of α-carbonyl radical.

To reveal the most reliable reaction mechanism, the radical/iminium ion coupling pathway was systematically studied. The single electron oxidation of α-amino radical **B** to iminium ion by the excited photosensitizer ***Ir^III^** was investigated using DFT calculations (Figure S2). This process is supposed to occur through the outer-sphere electron transfer. The estimation of this single electron transfer (SET) barrier between B and ***Ir^III^** was carried out by a protocol based on the Marcus theory in which the free energy gap is calculated by[5]

$$\Delta G^{\ddagger} = \Delta G_0^{\ddagger}\left(1 + \frac{\Delta_r G}{4\Delta G_0^{\ddagger}}\right)^2$$

where $\Delta_r G$ is the free energy change of the SET process and $\Delta G_0^{\ddagger}$ refers to the free energy gap when $\Delta_r G = 0$ calculated by

$$\Delta G_0^{\ddagger} = \frac{1}{4}\left[\frac{N_A e^2}{4\pi\varepsilon_0}\left(\frac{1}{\epsilon_s} - \frac{1}{\epsilon_{op}}\right)\left(\frac{1}{2R_{red}} + \frac{1}{2R_{ox}} - \frac{1}{R_{red} + R_{ox}}\right)\right]$$

where ϵ_s and ϵ_{op} refers to the relative static and optical dielectric constant, respectively, while R_{red} and R_{ox} refers to the radii of the reductant (**B**) and oxidant (***Ir^{III}**) molecules estimated from the volume calculated by the option "volume" in Gaussian 16, respectively. N_A is the Avogadro constant, e is the charge of electron and ε_0 is the vacuum dielectric constant.

The SET process from the α-amino radical **B** to the cationic iminium intermediate **C** is exergonic by 33.2 kcal/mol with an energy barrier of 14.7 kcal/mol, suggesting this process is thermodynamically unfeasible.

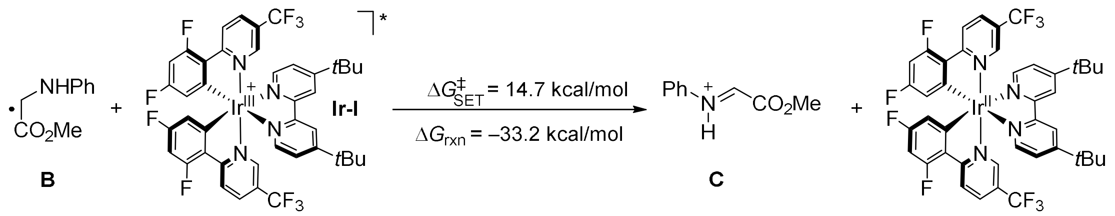

Figure S2. DFT study of the electron-transfer cycle of photocatalyst.

The complete DFT results of the radical addition transition states are shown in Figure S3. Comparison of the enantiomers and diastereomers of **TS-3** shows that (*R*,*S*)-**TS-3** has the lowest energy barrier. The corresponding activation free energy of (*R*,*S*)-**TS-3** ($\Delta G^{\ddagger}$ = 13.9 kcal/mol) is higher than that in the radical-radical coupling pathway (Figure 1c). Therefore, the radical/iminium ion coupling pathway can be ruled out.

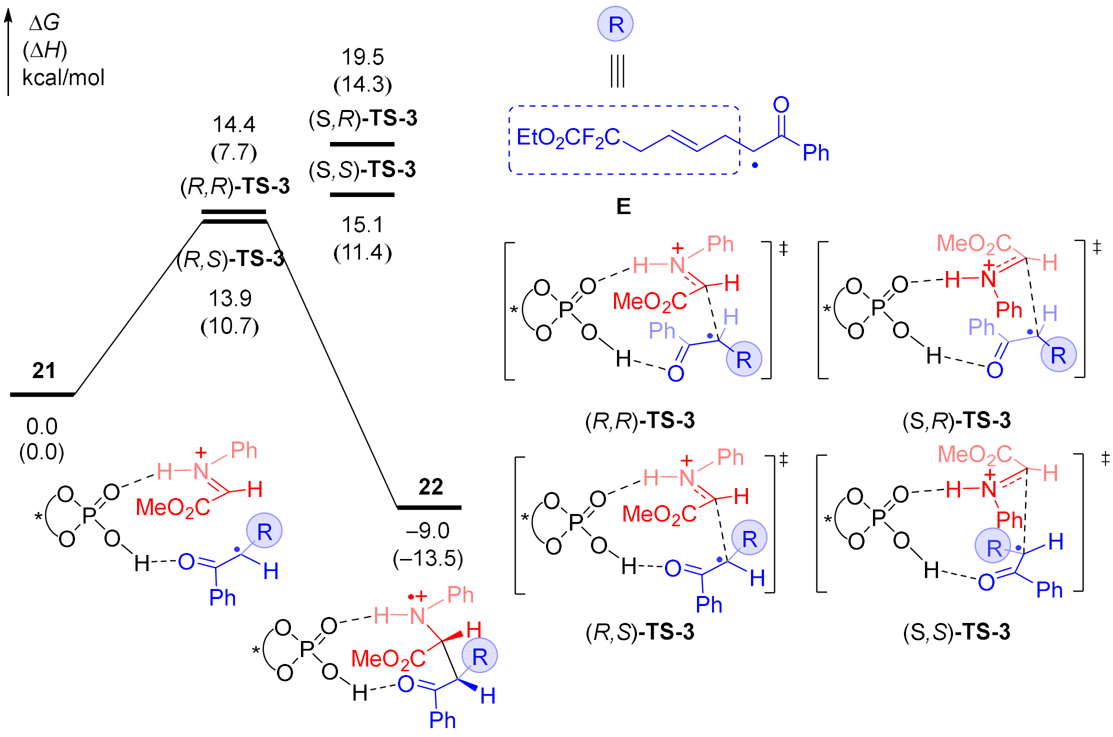

Figure S3. Free energy profile of the radical/iminium ion coupling pathway through radical addition.

In the computational study of the radical-radical coupling pathway, the relative stabilities of various CPA complexes were investigated. Three CPA complexes formed by hydrogen bonding interaction with α-amino radical or α-carbonyl radical were taken into account. As shown in Figure S4, complex **23** has the lowest relative free energy. Therefore, the free energy profile shown in the manuscript starts from the CPA complex **23**.

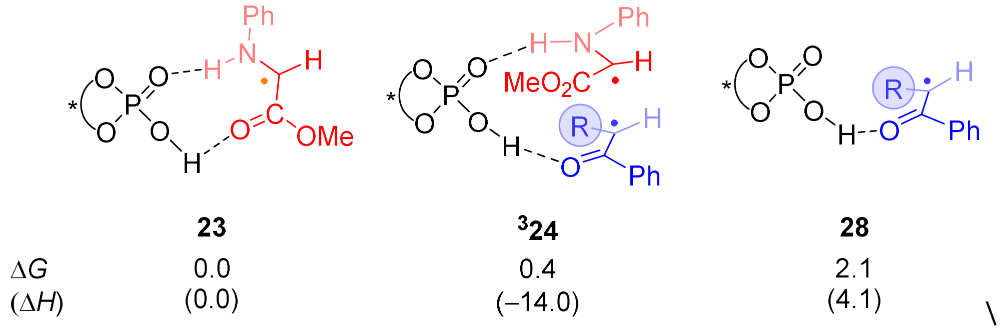

Figure S4. Calculated relative free energies of various CPA complexes. All energies are in kcal/mol and with respect to CPA complex **23**.

A proton-coupled electron transfer (PCET) transition state **^{OSS}TS-4** was located and discussed in the main text. Another conformation of this transition state, **^{OSS}TS-4′**, was also obtained in DFT calculation. The optimized structure and the relative free energy of **^{OSS}TS-4′** are shown in Figure S5. The relative higher energy of **^{OSS}TS-4′** suggests that this conformation is less stable.

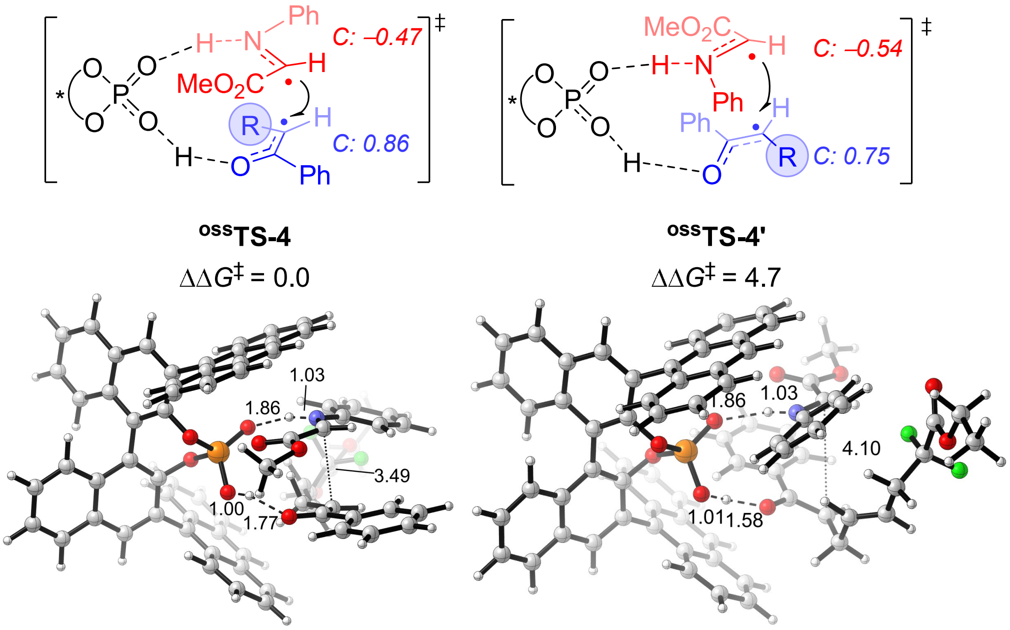

Figure S5. Optimized structures of the proton-coupled electron transfer (PCET) transition states. The italic numbers in **^{OSS}TS-4** denote the Mulliken atomic spin densities on α-carbonyl carbon and α-amino carbon. The bond lengths are in angstrom and energies are in kcal/mol.

The PCET characteristic of PCET transition state **^{OSS}TS-4** can be verified by analyzing the variation of Mulliken atomic spin population and natural population charge (NPA) along the reaction coordinate (Figure S6). According to the IRC results, the distance between the α-amino carbon and the α-carbonyl carbon (C3–C4) will initially decrease to 2.92 Å and then increase (Figure S6a), which indicates the C–C bond was not directly formed after **^{OSS}TS-4**. Moreover, based on the variation of Mulliken atomic spin population and the NPA charge along the IRC (Figure S6b and 6c), the charge transfer only occurs between the imine fragment (fragment 1) and the enol fragment (fragment 2). The Mulliken atomic spin density and NPA charge on the

CPA moiety is barely changed during the ^{OSS}**TS-4**. Taken together, these results support that the transformation through transition state ^{OSS}**TS-4** belongs to a PCET process.

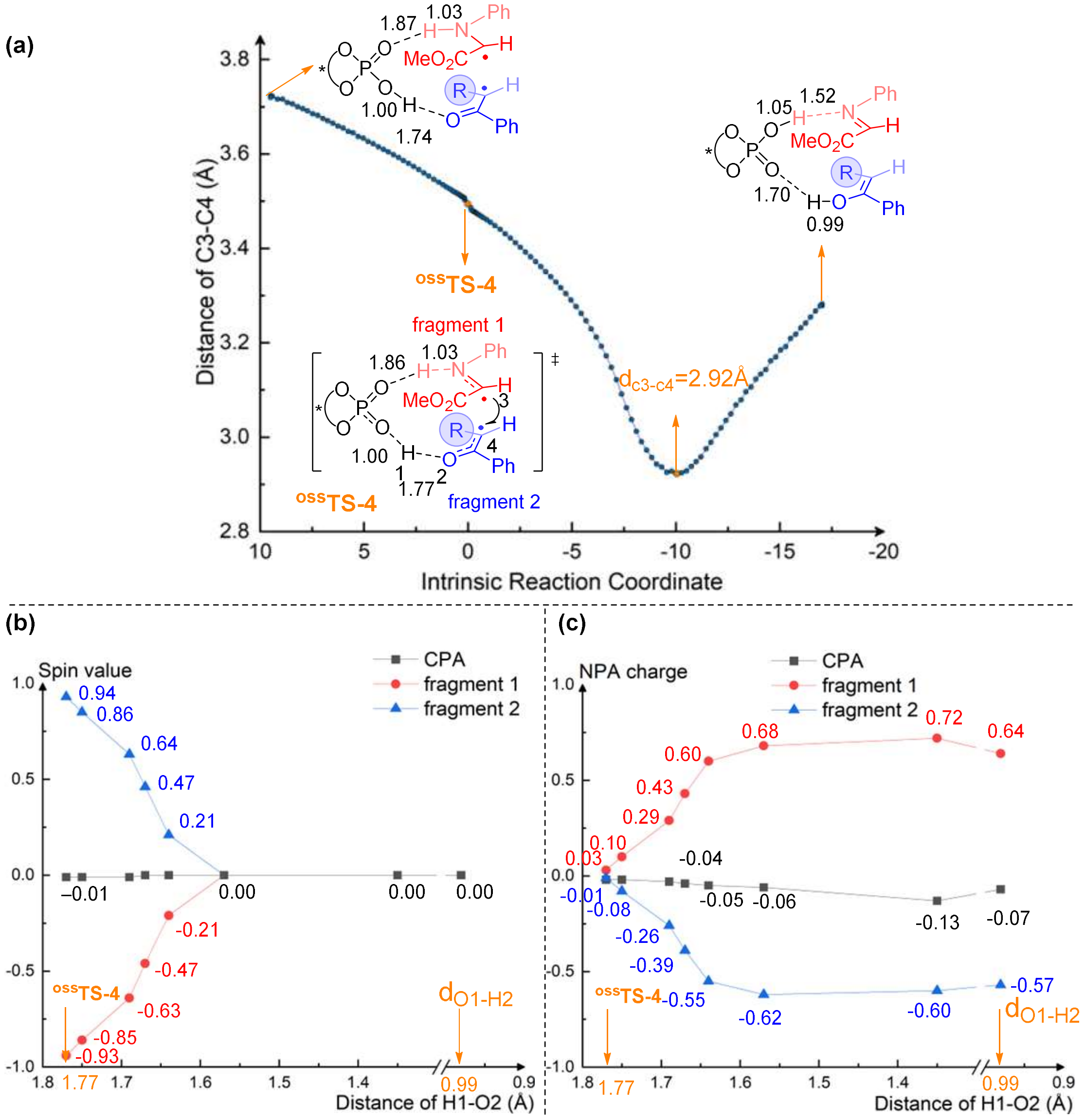

Figure S6. (a) IRC results of open-shell singlet transition state ^{OSS}TS-4. (b) Variation of Mulliken atomic spin densities of fragment 1 and fragment 2 in the selected structures along the IRC of open-shell singlet transition state ^{OSS}**TS-4**. (c) Variation of NPA charge of fragment 1 and fragment 2 in the selected structures along the IRC of open-shell singlet transition state ^{OSS}**TS-4**.

Cartesian coordinates (Å) and energies of optimized structures

2a

M06-2X SCF energy: -539.42701724 a.u.
M06-2X enthalpy: -539.205397 a.u.
M06-2X free energy: -539.256589 a.u.
M06-2X SCF energy in solution: -539.59974812 a.u.
M06-2X enthalpy in solution: -539.378128 a.u.
M06-2X free energy in solution: -539.429320 a.u.

Cartesian coordinates

ATOM	X	Y	Z
C	1.753314	-0.071117	1.563689
C	2.103269	-0.400717	0.143065
C	1.428967	0.922962	0.481365
H	0.938964	-0.593068	2.051267
H	2.571672	0.194264	2.225434
H	1.492259	-1.133186	-0.375157
H	2.076517	1.792556	0.426195
C	0.052197	1.304258	0.037411
O	-0.116594	2.457823	-0.312490
C	-1.124492	0.356835	0.002345
C	-1.098665	-1.002954	0.334705
C	-2.340369	0.921376	-0.406025
C	-2.257948	-1.771891	0.259448
H	-0.187783	-1.492037	0.653000
C	-3.495574	0.155754	-0.479555
H	-2.349829	1.974526	-0.663016
C	-3.457645	-1.197090	-0.145896
H	-2.218221	-2.824733	0.519897
H	-4.427268	0.612801	-0.797470
H	-4.358785	-1.799777	-0.202213
C	3.518163	-0.364117	-0.286097
H	4.147452	0.368059	0.220882
C	4.039843	-1.151330	-1.223184
H	3.435480	-1.888208	-1.745295
H	5.085937	-1.085599	-1.501227

4a

M06-2X SCF energy: -1598.81287049 a.u.
M06-2X enthalpy: -1598.289428 a.u.
M06-2X free energy: -1598.393208 a.u.
M06-2X SCF energy in solution: -1599.33544752 a.u.
M06-2X enthalpy in solution: -1598.812005 a.u.
M06-2X free energy in solution: -1598.915785 a.u.

Cartesian coordinates

ATOM	X	Y	Z
C	7.016476	-2.868550	0.124701
C	5.726445	-3.312072	0.405607
C	4.628339	-2.495251	0.160108
C	4.805696	-1.204396	-0.351556
C	6.103154	-0.759427	-0.624235
C	7.194815	-1.589743	-0.397743
H	7.870751	-3.511172	0.309757
H	5.568961	-4.310251	0.803199
H	3.622927	-2.867084	0.339613
H	6.247120	0.252051	-0.993231
H	8.194275	-1.225450	-0.615469
N	3.692523	-0.390774	-0.645684
H	3.968030	0.483796	-1.083859
C	2.759741	-0.105099	0.424723
H	2.725945	-0.973462	1.092618
C	3.259065	1.075050	1.264285
O	4.367487	1.538147	1.207895
O	2.295405	1.538009	2.079423
C	2.670705	2.668982	2.866490
H	1.792328	2.925320	3.456263
H	3.513193	2.417527	3.513938
H	2.956146	3.498580	2.216238
C	1.221652	1.531199	-0.688106
O	2.215238	2.020694	-1.199380
C	-0.065477	2.294768	-0.670867
C	-1.228326	1.823057	-0.052942
C	-0.083212	3.540063	-1.311095
C	-2.394865	2.582072	-0.087232
H	-1.248415	0.856462	0.436303
C	-1.244645	4.299806	-1.336364
H	0.829330	3.888707	-1.782931
C	-2.403234	3.819809	-0.724754
H	-3.293146	2.197025	0.383308
H	-1.251464	5.264089	-1.834393
H	-3.313232	4.411674	-0.748203
C	1.316949	0.139742	-0.069408
C	0.912777	-0.962275	-1.079239
H	0.654186	0.083497	0.798533
H	1.564822	-0.874566	-1.953423
H	1.134457	-1.931322	-0.615107
C	-0.534916	-0.907519	-1.468741

H	-0.834513	-0.103879	-2.142199
C	-1.467607	-1.739441	-1.010125
H	-1.190503	-2.545313	-0.331222
C	-2.925082	-1.619916	-1.349966
H	-3.111921	-0.755844	-1.994602
H	-3.298614	-2.506642	-1.874198
C	-3.758915	-1.464645	-0.097703
F	-3.569083	-2.527930	0.728210
F	-3.378980	-0.357532	0.602073
C	-5.266297	-1.348955	-0.372179
O	-5.747412	-1.417346	-1.473643
O	-5.937638	-1.173055	0.758075
C	-7.366544	-1.067790	0.616878
H	-7.587891	-0.223628	-0.042509
H	-7.734268	-1.976743	0.132327
C	-7.939753	-0.881935	2.003688
H	-7.541989	0.024686	2.465760
H	-9.027853	-0.795925	1.946647
H	-7.690040	-1.734342	2.639780

21

M06-2X SCF energy: -4086.67195949 a.u.

M06-2X enthalpy: -4085.470170 a.u.

M06-2X free energy: -4085.662761 a.u.

M06-2X SCF energy in solution: -4087.86760805 a.u.

M06-2X enthalpy in solution: -4086.665819 a.u.

M06-2X free energy in solution: -4086.858410 a.u.

Cartesian coordinates

ATOM	X	Y	Z
C	5.891905	5.306426	0.379557
C	5.238451	4.202151	-0.105583
C	3.833172	4.061705	0.054284
C	3.127703	5.074332	0.764811
C	3.832141	6.210452	1.244387
C	5.183037	6.329660	1.052484
H	6.965697	5.394864	0.250246
H	5.797653	3.422862	-0.611299
C	3.104275	2.927063	-0.432581
C	1.739130	4.925415	1.010756
H	3.276311	6.978518	1.774592
H	5.714487	7.199362	1.424078

C	1.045717	3.813628	0.598198
C	1.766126	2.831539	-0.124312
H	1.214258	5.703107	1.559843
C	3.761605	1.819883	-1.178491
C	4.478116	2.030963	-2.401968
C	3.671480	0.532923	-0.693392
C	4.513259	3.291999	-3.055832
C	5.151167	0.932418	-3.007536
C	4.362253	-0.568666	-1.257174
C	5.203757	3.451750	-4.230225
H	3.979380	4.129571	-2.620232
C	5.867789	1.133649	-4.216830
C	5.096990	-0.346064	-2.396204
C	5.897730	2.365633	-4.815038
H	5.215604	4.420318	-4.719218
H	6.385859	0.288016	-4.660183
H	5.649727	-1.169061	-2.842471
H	6.444556	2.511309	-5.740623
P	1.318292	0.349393	0.259072
O	0.872255	0.563049	1.746281
O	0.680970	-0.754069	-0.513184
C	4.366729	-1.903054	-0.590349
C	5.155016	-2.089038	0.563436
C	3.659026	-2.982271	-1.149356
C	5.268809	-3.399114	1.140355
C	5.880435	-1.017839	1.178643
C	3.770801	-4.287486	-0.556093
C	2.817732	-2.836410	-2.301784
C	6.107516	-3.588692	2.285229
C	4.572824	-4.466496	0.570710
C	6.654138	-1.234488	2.280515
H	5.810017	-0.021789	0.754896
C	3.059696	-5.385396	-1.147013
C	2.173981	-3.911091	-2.846423
H	2.704060	-1.850389	-2.740636
C	6.775747	-2.539973	2.842644
H	6.198102	-4.590777	2.696800
H	4.668655	-5.459117	1.006333
H	7.192932	-0.408855	2.733377
H	3.174928	-6.370558	-0.700851
C	2.298573	-5.208260	-2.266489
H	1.546663	-3.780291	-3.722950
H	7.410896	-2.692378	3.709155
H	1.787328	-6.049712	-2.723941

C	-0.407163	3.654419	0.894994
C	-0.826764	3.374932	2.210293
C	-1.350513	3.850586	-0.132356
C	-2.232063	3.321903	2.505927
C	0.095145	3.114995	3.278136
C	-2.751420	3.819843	0.180616
C	-0.968736	4.098104	-1.490985
C	-2.654646	3.028823	3.841007
C	-3.156574	3.560638	1.490396
C	-0.349749	2.827274	4.535151
H	1.158817	3.129825	3.066721
C	-3.703627	4.051611	-0.862003
C	-1.908430	4.289011	-2.461392
H	0.086009	4.134257	-1.743432
C	-1.745860	2.788408	4.826627
H	-3.720996	3.002474	4.048008
H	-4.219404	3.551522	1.726021
H	0.365090	2.628154	5.327220
H	-4.759819	4.039411	-0.605853
C	-3.299026	4.269595	-2.144903
H	-1.598363	4.470929	-3.485446
H	-2.076534	2.567657	5.836320
H	-4.029750	4.438424	-2.929245
H	-0.120678	0.610976	1.808727
O	2.905254	0.280747	0.445088
O	1.061775	1.705882	-0.554075
C	-2.319355	-4.408256	-3.252208
C	-1.857792	-5.395583	-2.379516
C	-0.955050	-5.076797	-1.373932
C	-0.517066	-3.756891	-1.252746
C	-0.963976	-2.759845	-2.120866
C	-1.870559	-3.096217	-3.121756
H	-3.025818	-4.666415	-4.033822
H	-2.204840	-6.417965	-2.481920
H	-0.609622	-5.852451	-0.698990
H	-0.592483	-1.745206	-2.009865
H	-2.221339	-2.328627	-3.803623
N	0.388676	-3.354472	-0.232744
H	0.619763	-2.307728	-0.215789
C	0.925826	-4.097887	0.658343
H	0.742898	-5.164753	0.707999
C	1.781985	-3.430158	1.700191
O	2.047410	-2.257685	1.672105
O	2.123485	-4.300504	2.635540

C	2.870266	-3.741246	3.730318
H	3.135702	-4.585327	4.362781
H	3.758657	-3.232043	3.355024
H	2.242674	-3.028973	4.270804
C	-2.169223	-0.721808	0.798446
O	-1.709504	0.343498	1.252595
C	-1.930157	-2.017675	1.513716
C	-2.510712	-3.226156	1.107479
C	-1.090674	-2.013791	2.635744
C	-2.227688	-4.409741	1.782267
H	-3.182176	-3.265957	0.256313
C	-0.817421	-3.193873	3.316539
H	-0.655595	-1.081497	2.975965
C	-1.371053	-4.400021	2.882759
H	-2.688525	-5.336782	1.455421
H	-0.176028	-3.175479	4.192413
H	-1.155391	-5.320731	3.416384
C	-2.909227	-0.694866	-0.431091
C	-3.199862	0.587545	-1.127054
H	-3.376805	-1.593638	-0.813684
H	-2.443009	1.338928	-0.879835
H	-3.228058	0.440394	-2.211498
C	-4.564039	1.019204	-0.633185
H	-4.595300	1.368100	0.398877
C	-5.679649	0.944149	-1.355088
H	-5.638229	0.583170	-2.382065
C	-7.030827	1.345869	-0.842229
H	-6.977666	1.710802	0.187291
H	-7.467997	2.145410	-1.450676
C	-7.990512	0.174901	-0.880452
F	-8.091101	-0.300907	-2.150111
F	-7.520386	-0.844656	-0.114702
C	-9.398938	0.530348	-0.380193
O	-9.675711	1.604838	0.088031
O	-10.224692	-0.493127	-0.528210
C	-11.576756	-0.268087	-0.077997
H	-11.546585	-0.006381	0.983345
H	-11.982612	0.587726	-0.624384
C	-12.352138	-1.539379	-0.338013
H	-11.914013	-2.376788	0.210043
H	-13.387485	-1.413417	-0.011719
H	-12.350081	-1.780326	-1.403582

22

M06-2X SCF energy: -4086.70297298 a.u.

M06-2X enthalpy: -4085.498318 a.u.

M06-2X free energy: -4085.683732 a.u.

M06-2X SCF energy in solution: -4087.89204834 a.u.

M06-2X enthalpy in solution: -4086.687393 a.u.

M06-2X free energy in solution: -4086.872807 a.u.

Cartesian coordinates

ATOM	X	Y	Z
C	7.017729	1.981574	3.563069
C	6.306820	1.227341	2.664484
C	5.466374	1.846314	1.699702
C	5.345472	3.265240	1.719395
C	6.105359	4.018001	2.653850
C	6.929110	3.393732	3.552556
H	7.651784	1.490009	4.293768
H	6.378212	0.145782	2.691537
C	4.707718	1.101134	0.737177
C	4.438895	3.907782	0.838076
H	6.010253	5.100140	2.646015
H	7.503976	3.975807	4.265093
C	3.652970	3.196190	-0.034882
C	3.825404	1.788990	-0.067049
H	4.339864	4.989638	0.880122
C	4.778915	-0.381872	0.633616
C	6.004262	-1.087823	0.399617
C	3.614744	-1.115669	0.726671
C	7.234741	-0.421893	0.149251
C	5.987108	-2.511466	0.377100
C	3.570423	-2.531704	0.694181
C	8.385059	-1.135519	-0.070848
H	7.255005	0.662038	0.124954
C	7.198258	-3.221124	0.160567
C	4.759825	-3.202327	0.542805
C	8.373278	-2.550792	-0.053504
H	9.314301	-0.610120	-0.265457
H	7.169974	-4.307106	0.159009
H	4.760090	-4.289397	0.524857
H	9.293861	-3.099594	-0.221815
P	1.805134	0.249318	-0.425497
O	0.796748	1.283575	0.194534
O	1.279572	-0.635910	-1.493213

C	2.283016	-3.278790	0.824855
C	1.719915	-3.490204	2.099198
C	1.696285	-3.857936	-0.316058
C	0.555694	-4.321431	2.233484
C	2.287696	-2.931407	3.288679
C	0.523589	-4.679936	-0.173264
C	2.248811	-3.695853	-1.629375
C	0.037698	-4.594937	3.539187
C	-0.026535	-4.879729	1.093925
C	1.759379	-3.208168	4.517069
H	3.158443	-2.289804	3.201768
C	-0.012975	-5.335983	-1.327911
C	1.717055	-4.350275	-2.701759
H	3.109203	-3.048186	-1.756486
C	0.626238	-4.064936	4.648825
H	-0.829289	-5.244943	3.623233
H	-0.894178	-5.527814	1.204152
H	2.210098	-2.781926	5.407409
H	-0.879632	-5.980404	-1.197738
C	0.574017	-5.193419	-2.550482
H	2.164526	-4.232360	-3.683738
H	0.236068	-4.287607	5.636777
H	0.174680	-5.714616	-3.415716
C	2.624254	3.854372	-0.889425
C	1.464060	4.384432	-0.290351
C	2.816818	3.944836	-2.281039
C	0.478486	5.030470	-1.112210
C	1.205414	4.290795	1.117406
C	1.823359	4.589891	-3.092070
C	3.983782	3.426124	-2.929674
C	-0.696068	5.573754	-0.499565
C	0.681552	5.117469	-2.489153
C	0.077286	4.829318	1.663683
H	1.922172	3.778676	1.749515
C	2.030507	4.684486	-4.504761
C	4.144012	3.535205	-4.279339
H	4.750313	2.948025	-2.328360
C	-0.891851	5.485050	0.845655
H	-1.425104	6.066961	-1.136690
H	-0.065500	5.611570	-3.106594
H	-0.081558	4.764117	2.736463
H	1.271305	5.180413	-5.103367
C	3.152917	4.173246	-5.082967
H	5.037300	3.139018	-4.751484

H	-1.780996	5.911744	1.299781
H	3.303714	4.254132	-6.154568
H	-0.088262	1.242901	-0.239002
O	2.403173	-0.450063	0.888239
O	3.056154	1.077733	-0.985767
C	-1.333954	-3.438962	-5.382879
C	-2.271250	-3.890799	-4.432985
C	-2.201791	-3.464772	-3.129444
C	-1.173937	-2.549222	-2.739316
C	-0.207182	-2.121853	-3.708002
C	-0.308887	-2.560721	-5.010682
H	-1.406656	-3.781012	-6.409708
H	-3.047124	-4.586847	-4.732009
H	-2.908594	-3.835407	-2.397113
H	0.575749	-1.443110	-3.383376
H	0.411136	-2.223611	-5.747953
N	-1.064924	-2.052663	-1.504305
H	-0.231642	-1.448629	-1.311915
C	-2.024041	-2.193851	-0.430380
H	-2.457960	-3.197632	-0.431860
C	-1.289514	-1.943337	0.886405
O	-0.225441	-1.382968	0.941605
O	-2.049673	-2.261770	1.924593
C	-1.589716	-1.742708	3.189012
H	-2.146749	-2.284746	3.949740
H	-0.517305	-1.904391	3.297867
H	-1.814427	-0.673220	3.226761
C	-2.581108	0.237497	-0.145572
O	-1.616703	0.598746	-0.809841
C	-3.134358	1.054772	0.955974
C	-4.414010	0.811404	1.476358
C	-2.367653	2.116970	1.461972
C	-4.922605	1.621184	2.484752
H	-5.044168	0.028496	1.070543
C	-2.871216	2.901943	2.489512
H	-1.378522	2.325022	1.065552
C	-4.146855	2.657178	2.999933
H	-5.924404	1.440788	2.858812
H	-2.269482	3.711492	2.888031
H	-4.538259	3.281357	3.797082
C	-3.179667	-1.134718	-0.477541
C	-3.944730	-1.063209	-1.812813
H	-3.871651	-1.448211	0.306329
H	-3.253129	-0.760887	-2.608053

H	-4.338677	-2.055433	-2.057765
C	-5.085268	-0.087344	-1.705563
H	-4.823995	0.971483	-1.682749
C	-6.359730	-0.449361	-1.579411
H	-6.626583	-1.506023	-1.596136
C	-7.489593	0.518637	-1.386917
H	-7.132510	1.550598	-1.335416
H	-8.210409	0.462110	-2.210011
C	-8.244040	0.208682	-0.111271
F	-8.712030	-1.064359	-0.144237
F	-7.405248	0.284994	0.965530
C	-9.420034	1.163076	0.146610
O	-9.642656	2.123319	-0.544416
O	-10.110300	0.782783	1.208272
C	-11.242911	1.614520	1.542506
H	-10.880207	2.629833	1.725175
H	-11.911065	1.645619	0.677640
C	-11.900931	1.006404	2.759650
H	-11.204372	0.975954	3.600760
H	-12.767746	1.606235	3.047695
H	-12.237246	-0.011084	2.547700

23

M06-2X SCF energy: -3042.02739863 a.u.
M06-2X enthalpy: -3041.154623 a.u.
M06-2X free energy: -3041.291414 a.u.
M06-2X SCF energy in solution: -3042.84833113 a.u.
M06-2X enthalpy in solution: -3041.975556 a.u.
M06-2X free energy in solution: -3042.112347 a.u.

Cartesian coordinates

ATOM	X	Y	Z
C	-4.305805	4.292396	2.197156
C	-3.284074	3.725654	1.478446
C	-3.424258	2.430483	0.908762
C	-4.635462	1.719635	1.140704
C	-5.679622	2.339435	1.877286
C	-5.524594	3.599933	2.390329
H	-4.175705	5.280139	2.627744
H	-2.351427	4.263135	1.348222
C	-2.382459	1.801046	0.149709
C	-4.773255	0.393012	0.660356

H	-6.601013	1.785074	2.033287
H	-6.326161	4.064239	2.955583
C	-3.753768	-0.244422	-0.002478
C	-2.561978	0.489451	-0.239418
H	-5.700985	-0.142480	0.846511
C	-1.102516	2.491816	-0.167398
C	-1.048785	3.737699	-0.872694
C	0.086504	1.890047	0.188827
C	-2.204308	4.372415	-1.404258
C	0.218021	4.352207	-1.082587
C	1.364367	2.463524	-0.043095
C	-2.105741	5.568644	-2.068101
H	-3.170830	3.894594	-1.287298
C	0.284513	5.598058	-1.761959
C	1.397619	3.698720	-0.646285
C	-0.849961	6.198395	-2.240102
H	-2.999161	6.034511	-2.471512
H	1.258716	6.057848	-1.903809
H	2.358861	4.173266	-0.827803
H	-0.789053	7.147725	-2.762271
P	-0.251338	-0.620381	-0.097006
O	-0.811660	-1.671197	0.930597
O	0.855890	-1.003556	-0.991644
C	2.620477	1.750106	0.322823
C	2.903253	1.446529	1.670943
C	3.552141	1.434418	-0.690431
C	4.147274	0.814349	2.005909
C	2.009890	1.771711	2.742619
C	4.823115	0.868960	-0.329928
C	3.290067	1.641692	-2.086201
C	4.413433	0.470521	3.368971
C	5.083081	0.560611	1.003305
C	2.308531	1.450088	4.033447
H	1.082736	2.287483	2.518318
C	5.795619	0.616558	-1.349955
C	4.239228	1.375641	-3.028442
H	2.312353	1.997918	-2.389654
C	3.523733	0.775191	4.354543
H	5.346998	-0.036872	3.597728
H	6.036361	0.105950	1.267500
H	1.615291	1.708129	4.828234
H	6.752218	0.195903	-1.052477
C	5.522225	0.874516	-2.657705
H	4.012234	1.531688	-4.078693

H	3.737335	0.513932	5.386768
H	6.261239	0.666576	-3.424711
C	-3.888244	-1.657248	-0.457017
C	-3.939678	-2.693570	0.495175
C	-3.998779	-1.941435	-1.832254
C	-4.134715	-4.046676	0.053833
C	-3.782405	-2.462953	1.901824
C	-4.179080	-3.299091	-2.260818
C	-3.953986	-0.920286	-2.835745
C	-4.203656	-5.095455	1.025554
C	-4.250056	-4.315814	-1.308721
C	-3.848336	-3.490855	2.794850
H	-3.587204	-1.453692	2.246722
C	-4.292989	-3.580364	-3.658885
C	-4.065740	-1.228676	-4.159267
H	-3.829222	0.113187	-2.529472
C	-4.071817	-4.829474	2.354368
H	-4.360595	-6.111237	0.672604
H	-4.395492	-5.342658	-1.637553
H	-3.721029	-3.293782	3.854762
H	-4.425799	-4.614644	-3.964838
C	-4.236044	-2.580088	-4.581660
H	-4.024654	-0.439355	-4.903125
H	-4.125078	-5.632114	3.083120
H	-4.321499	-2.803913	-5.640222
H	-0.083730	-1.920919	1.555483
O	0.038086	0.667240	0.835726
O	-1.549943	-0.164019	-0.928529
C	5.704102	-2.598283	-3.358609
C	6.240059	-2.865305	-2.101114
C	5.474507	-2.702437	-0.952010
C	4.146718	-2.267854	-1.059840
C	3.604485	-1.990502	-2.322959
C	4.383901	-2.159134	-3.457825
H	6.308242	-2.730354	-4.250223
H	7.267929	-3.203008	-2.008439
H	5.917323	-2.899972	0.018054
H	2.579088	-1.639634	-2.388471
H	3.954702	-1.939472	-4.430767
N	3.323520	-2.091498	0.050830
H	2.403531	-1.673108	-0.135812
C	3.606116	-2.415809	1.327256
H	4.548451	-2.876111	1.582622
C	2.627336	-2.168348	2.326412

O	1.518049	-1.656395	2.117680
O	3.019485	-2.550699	3.558081
C	2.060590	-2.332596	4.584514
H	2.560989	-2.593360	5.516682
H	1.741650	-1.287990	4.595018
H	1.185792	-2.972133	4.435199

24

M06-2X SCF energy: -4086.86302743 a.u.

M06-2X enthalpy: -4085.662899 a.u.

M06-2X free energy: -4085.852441 a.u.

M06-2X SCF energy in solution: -4088.02135796 a.u.

M06-2X enthalpy in solution: -4086.821230 a.u.

M06-2X free energy in solution: -4087.010772 a.u.

Cartesian coordinates

ATOM	X	Y	Z
C	-6.217393	-4.277932	1.789085
C	-5.390458	-3.533368	0.986731
C	-3.993930	-3.466500	1.242226
C	-3.478524	-4.165478	2.370485
C	-4.358632	-4.937498	3.173501
C	-5.697860	-4.998306	2.890272
H	-7.282113	-4.310110	1.580870
H	-5.800909	-2.976117	0.151592
C	-3.098668	-2.692500	0.432233
C	-2.094800	-4.083268	2.676963
H	-3.946203	-5.472675	4.024498
H	-6.363328	-5.587335	3.513035
C	-1.244395	-3.293705	1.945579
C	-1.788650	-2.571729	0.849301
H	-1.703005	-4.658952	3.511868
C	-3.533595	-1.998633	-0.812346
C	-4.136974	-2.688815	-1.915277
C	-3.289103	-0.649150	-0.949096
C	-4.316438	-4.098843	-1.935144
C	-4.538764	-1.940512	-3.058838
C	-3.669766	0.115935	-2.082562
C	-4.897666	-4.719344	-3.011770
H	-3.978624	-4.686758	-1.088853
C	-5.147712	-2.611811	-4.151831
C	-4.303911	-0.541061	-3.106503

C	-5.330576	-3.969427	-4.130465
H	-5.020886	-5.797732	-3.008268
H	-5.456659	-2.023451	-5.011511
H	-4.616259	0.017098	-3.985560
H	-5.793470	-4.473400	-4.972716
P	-1.085389	-0.156615	0.248866
O	-0.850110	0.169810	1.759544
O	-0.209098	0.452334	-0.772822
C	-3.390877	1.582979	-2.131067
C	-4.288969	2.478418	-1.519986
C	-2.246776	2.060797	-2.796644
C	-4.045057	3.890692	-1.600582
C	-5.457268	2.037805	-0.818961
C	-2.002593	3.475460	-2.853963
C	-1.292543	1.189261	-3.417195
C	-4.982410	4.796374	-1.011379
C	-2.905237	4.354124	-2.257746
C	-6.327050	2.933039	-0.265866
H	-5.646553	0.972269	-0.736119
C	-0.826061	3.955773	-3.512834
C	-0.183628	1.686676	-4.034594
H	-1.450947	0.117719	-3.362571
C	-6.093922	4.336233	-0.370191
H	-4.781327	5.861875	-1.086424
H	-2.714699	5.424615	-2.301283
H	-7.207714	2.576922	0.259500
H	-0.650839	5.028436	-3.530281
C	0.058884	3.092327	-4.083177
H	0.535108	1.007488	-4.482488
H	-6.800844	5.031377	0.072002
H	0.957942	3.463793	-4.564625
C	0.226472	-3.266076	2.210009
C	0.768832	-2.444540	3.214587
C	1.062684	-4.101626	1.442466
C	2.184090	-2.474254	3.464936
C	-0.035701	-1.550233	3.993773
C	2.472778	-4.134425	1.711654
C	0.559440	-4.937691	0.392829
C	2.732976	-1.607044	4.462856
C	2.997705	-3.324890	2.718378
C	0.527981	-0.728597	4.925270
H	-1.102410	-1.511481	3.803694
C	3.312699	-4.992698	0.932692
C	1.393663	-5.736530	-0.332595

H	-0.503754	-4.925431	0.176292
C	1.934666	-0.755096	5.165961
H	3.805208	-1.641682	4.639250
H	4.068399	-3.346816	2.914854
H	-0.095828	-0.041114	5.487167
H	4.379222	-5.002355	1.145179
C	2.793544	-5.767366	-0.061400
H	0.991616	-6.357237	-1.127055
H	2.361802	-0.093898	5.913409
H	3.440850	-6.409887	-0.649712
H	-0.443782	1.061239	1.956666
O	-2.676700	0.023695	0.088971
O	-0.918644	-1.764771	0.144884
C	4.633761	3.145098	-1.430435
C	4.057724	4.285831	-0.874516
C	2.687560	4.354609	-0.639290
C	1.874624	3.260781	-0.969818
C	2.449032	2.121472	-1.560496
C	3.817858	2.064765	-1.775246
H	5.705193	3.092491	-1.592930
H	4.680570	5.131913	-0.598155
H	2.266884	5.229383	-0.153901
H	1.802261	1.283300	-1.807311
H	4.252186	1.165942	-2.199228
N	0.521535	3.215924	-0.664909
H	0.070320	2.295575	-0.749863
C	-0.225991	4.186331	-0.092743
H	0.103135	5.215171	-0.082394
C	-1.442454	3.757741	0.527342
O	-1.798132	2.586104	0.600109
O	-2.162006	4.769460	1.068437
C	-3.274731	4.327631	1.843009
H	-3.845877	5.224013	2.084216
H	-3.886978	3.622085	1.278410
H	-2.922272	3.839206	2.757080
C	1.612281	2.809589	2.327977
O	0.448110	2.410344	2.520643
C	1.953544	4.231618	2.638803
C	3.272551	4.690718	2.715235
C	0.901853	5.134017	2.830420
C	3.534901	6.034330	2.961711
H	4.102902	4.004095	2.586441
C	1.165272	6.478235	3.067578
H	-0.118061	4.769263	2.768585

C	2.482160	6.931469	3.130380
H	4.561629	6.381177	3.021202
H	0.342231	7.173141	3.200365
H	2.688225	7.981387	3.314420
C	2.606746	1.933091	1.770795
C	2.262474	0.516520	1.486550
H	3.573126	2.320969	1.465607
H	1.712038	0.103912	2.346754
H	1.549818	0.508123	0.648024
C	3.424904	-0.359552	1.134843
H	4.228026	-0.441749	1.869598
C	3.485664	-1.066396	0.009398
H	2.674097	-0.990353	-0.714021
C	4.607355	-2.006693	-0.318024
H	5.261430	-2.161378	0.546390
H	4.222791	-2.988992	-0.613085
C	5.461329	-1.491264	-1.452879
F	4.696437	-1.249932	-2.554098
F	6.048557	-0.310324	-1.122014
C	6.578874	-2.464589	-1.861964
O	6.786093	-3.512586	-1.305274
O	7.268997	-1.986341	-2.889469
C	8.347452	-2.819727	-3.349666
H	9.041825	-2.978650	-2.519517
H	7.936886	-3.793007	-3.633539
C	8.996547	-2.107063	-4.514824
H	9.383182	-1.134098	-4.202505
H	9.826230	-2.705126	-4.900487
H	8.274493	-1.951553	-5.319848

25

M06-2X SCF energy: -4086.90935116 a.u.

M06-2X enthalpy: -4085.707753 a.u.

M06-2X free energy: -4085.893535 a.u.

M06-2X SCF energy in solution: -4088.06615813 a.u.

M06-2X enthalpy in solution: -4086.864560 a.u.

M06-2X free energy in solution: -4087.050342 a.u.

Cartesian coordinates

ATOM	X	Y	Z
C	5.849880	-4.775483	0.519366
C	4.748250	-4.130669	0.017701

C	4.411101	-2.819976	0.453620
C	5.226656	-2.205829	1.444349
C	6.367270	-2.894822	1.935100
C	6.677348	-4.149924	1.482256
H	6.086837	-5.778587	0.179215
H	4.115400	-4.624023	-0.712063
C	3.265819	-2.113593	-0.037334
C	4.887656	-0.915991	1.925220
H	6.983549	-2.406168	2.684782
H	7.548245	-4.671333	1.866059
C	3.757376	-0.258822	1.504605
C	2.935150	-0.906043	0.544942
H	5.539398	-0.434914	2.650247
C	2.409264	-2.651360	-1.127610
C	2.927690	-2.969710	-2.425585
C	1.056740	-2.794861	-0.907260
C	4.281196	-2.735432	-2.791841
C	2.045703	-3.505766	-3.405738
C	0.161469	-3.334398	-1.867850
C	4.734173	-3.051285	-4.047558
H	4.956088	-2.292247	-2.067880
C	2.548245	-3.832948	-4.692844
C	0.675480	-3.690904	-3.089972
C	3.863630	-3.617030	-5.008873
H	5.770060	-2.859618	-4.308804
H	1.862199	-4.250700	-5.424633
H	0.013325	-4.113915	-3.841449
H	4.238221	-3.866516	-5.996335
P	0.359342	-0.862884	0.633312
O	0.078428	-0.652817	2.067794
O	-0.538544	-0.208098	-0.458695
C	-1.281327	-3.545812	-1.553910
C	-1.658517	-4.615103	-0.717361
C	-2.257075	-2.717879	-2.137339
C	-3.050470	-4.880910	-0.493218
C	-0.700424	-5.465499	-0.079000
C	-3.648568	-2.998369	-1.911683
C	-1.921236	-1.577179	-2.940135
C	-3.425536	-5.982873	0.338718
C	-4.012226	-4.069678	-1.097389
C	-1.096159	-6.498301	0.719838
H	0.356425	-5.275527	-0.234680
C	-4.636752	-2.168936	-2.535686
C	-2.892293	-0.804427	-3.505659

H	-0.875478	-1.321047	-3.070214
C	-2.480455	-6.767208	0.930658
H	-4.484876	-6.174424	0.490330
H	-5.066966	-4.280206	-0.931606
H	-0.352481	-7.126158	1.200009
H	-5.684846	-2.409368	-2.373400
C	-4.272508	-1.115233	-3.318902
H	-2.618792	0.067762	-4.090917
H	-2.774784	-7.599608	1.562167
H	-5.026553	-0.491279	-3.788579
C	3.485405	1.129140	1.989044
C	2.832680	1.346973	3.215355
C	4.005914	2.212831	1.253223
C	2.736835	2.682980	3.737147
C	2.259653	0.278485	3.978716
C	3.912194	3.542255	1.788147
C	4.642386	2.043481	-0.020063
C	2.093183	2.891734	4.998227
C	3.284680	3.743797	3.017445
C	1.640311	0.522562	5.168679
H	2.296848	-0.728382	3.578612
C	4.462599	4.632990	1.043125
C	5.138639	3.113679	-0.707435
H	4.717241	1.045288	-0.439703
C	1.562106	1.846905	5.693556
H	2.035636	3.906398	5.383793
H	3.220465	4.751570	3.423056
H	1.195589	-0.297481	5.722925
H	4.383303	5.632438	1.463423
C	5.051583	4.431655	-0.169118
H	5.604284	2.964897	-1.676532
H	1.070775	2.016355	6.646575
H	5.443728	5.269083	-0.736400
H	-1.471304	0.006074	2.738547
O	0.537846	-2.438835	0.322513
O	1.786144	-0.256402	0.136288
C	-4.443789	3.609193	-2.246144
C	-5.356804	2.570965	-2.058714
C	-4.960515	1.400058	-1.426584
C	-3.642227	1.267674	-0.974982
C	-2.720890	2.296557	-1.181824
C	-3.127662	3.470105	-1.811617
H	-4.757293	4.522092	-2.742982
H	-6.378951	2.671960	-2.410463

H	-5.664150	0.583413	-1.300667
H	-1.699927	2.178278	-0.830220
H	-2.409466	4.266295	-1.966472
N	-3.170184	0.117820	-0.301520
H	-1.554571	-0.199624	-0.311592
C	-3.958035	-0.637287	0.349077
H	-5.030088	-0.464646	0.464634
C	-3.339910	-1.782249	1.093168
O	-2.184450	-2.108003	0.985207
O	-4.220481	-2.363478	1.903727
C	-3.639788	-3.316410	2.799673
H	-4.469290	-3.722470	3.376448
H	-3.127674	-4.102739	2.241556
H	-2.923951	-2.805247	3.448341
C	-2.862748	1.389758	2.622949
O	-2.430472	0.130025	2.910680
C	-4.343635	1.439213	2.536368
C	-5.007866	2.387477	1.748254
C	-5.104367	0.473299	3.208901
C	-6.395369	2.375510	1.645023
H	-4.434331	3.109937	1.174101
C	-6.491440	0.463373	3.103867
H	-4.593054	-0.276922	3.801388
C	-7.143774	1.413726	2.320475
H	-6.888429	3.108018	1.012868
H	-7.064418	-0.292967	3.631842
H	-8.225841	1.401267	2.232294
C	-2.085347	2.471906	2.467264
C	-0.586037	2.518006	2.607281
H	-2.586592	3.417382	2.280713
H	-0.331806	2.866349	3.619730
H	-0.138294	1.524991	2.508175
C	0.055887	3.448012	1.613676
H	-0.104533	4.517137	1.769496
C	0.805073	3.053832	0.585782
H	0.970713	1.994395	0.403510
C	1.469437	4.014103	-0.364547
H	1.779444	4.931843	0.145178
H	2.370132	3.577900	-0.807367
C	0.546687	4.425101	-1.483709
F	0.036687	3.341412	-2.128703
F	-0.520261	5.116934	-0.986893
C	1.211985	5.318860	-2.542081
O	2.368675	5.651330	-2.512238

O	0.334168	5.665744	-3.478822
C	0.851146	6.501674	-4.527610
H	1.247890	7.417376	-4.079517
H	1.681630	5.979360	-5.011380
C	-0.288909	6.776314	-5.482719
H	-1.106192	7.287016	-4.967795
H	0.057109	7.410638	-6.302933
H	-0.671704	5.842406	-5.901122

26

M06-2X SCF energy: -4086.93317474 a.u.

M06-2X enthalpy: -4085.728891 a.u.

M06-2X free energy: -4085.911129 a.u.

M06-2X SCF energy in solution: -4088.08676448 a.u.

M06-2X enthalpy in solution: -4086.882481 a.u.

M06-2X free energy in solution: -4087.064719 a.u.

Cartesian coordinates

ATOM	X	Y	Z
C	-5.673820	4.802094	1.553871
C	-4.752271	4.098360	0.820525
C	-4.484715	2.730557	1.102262
C	-5.162106	2.125452	2.199474
C	-6.119432	2.877290	2.930722
C	-6.378482	4.184488	2.613543
H	-5.859388	5.846161	1.322505
H	-4.212206	4.589946	0.019393
C	-3.536029	1.954089	0.355464
C	-4.835556	0.799462	2.584408
H	-6.633382	2.393693	3.756963
H	-7.109243	4.752082	3.180503
C	-3.875542	0.076107	1.923650
C	-3.259791	0.675279	0.791721
H	-5.327937	0.364634	3.450774
C	-2.769659	2.521151	-0.790124
C	-3.389862	3.096092	-1.946592
C	-1.390568	2.482124	-0.749114
C	-4.794935	3.062485	-2.156916
C	-2.567539	3.688334	-2.947112
C	-0.549756	3.077797	-1.723107
C	-5.350122	3.616922	-3.281973
H	-5.426810	2.581173	-1.418073

C	-3.174984	4.264832	-4.094026
C	-1.156404	3.683897	-2.795408
C	-4.534722	4.235656	-4.259117
H	-6.424835	3.575494	-3.427830
H	-2.534239	4.721116	-4.843575
H	-0.541775	4.154688	-3.558653
H	-4.988669	4.674529	-5.141710
P	-0.786373	0.218982	0.192396
O	-0.418216	-0.195837	1.675454
O	-0.031614	-0.360146	-0.927814
C	0.934787	3.070759	-1.562561
C	1.532589	3.919507	-0.605067
C	1.729960	2.277693	-2.406952
C	2.962915	4.003275	-0.531981
C	0.768874	4.733733	0.290823
C	3.163448	2.369851	-2.323891
C	1.170339	1.359181	-3.356553
C	3.564382	4.907434	0.397877
C	3.743139	3.223913	-1.390574
C	1.379267	5.576532	1.175873
H	-0.314555	4.680016	0.256236
C	3.967281	1.590640	-3.217765
C	1.972388	0.636661	-4.187342
H	0.094465	1.227803	-3.384730
C	2.799876	5.676143	1.225578
H	4.649793	4.965306	0.425973
H	4.827594	3.296945	-1.334770
H	0.777882	6.184877	1.844082
H	5.048648	1.675544	-3.141447
C	3.393672	0.760424	-4.129538
H	1.532322	-0.067673	-4.885885
H	3.266098	6.362596	1.925615
H	4.008313	0.153741	-4.787535
C	-3.404576	-1.257812	2.394886
C	-2.565366	-1.326903	3.524786
C	-3.742388	-2.420890	1.680977
C	-2.038773	-2.598908	3.933537
C	-2.169416	-0.168088	4.270584
C	-3.198689	-3.684038	2.091533
C	-4.607506	-2.395353	0.540364
C	-1.175745	-2.665791	5.073294
C	-2.360512	-3.742856	3.204012
C	-1.341753	-0.273265	5.348820
H	-2.526382	0.805854	3.954282

C	-3.525813	-4.857924	1.343994
C	-4.908937	-3.539214	-0.137801
H	-5.022334	-1.444612	0.220663
C	-0.838755	-1.541947	5.764257
H	-0.792233	-3.638581	5.369153
H	-1.947456	-4.702266	3.508906
H	-1.052868	0.618677	5.896067
H	-3.098239	-5.807281	1.655955
C	-4.361409	-4.791547	0.269607
H	-5.568140	-3.501316	-1.000286
H	-0.180823	-1.602596	6.625274
H	-4.607357	-5.696325	-0.277887
H	0.484466	0.126259	1.880913
O	-0.770871	1.836624	0.307847
O	-2.354792	-0.102907	0.090172
C	3.942685	-3.417190	-3.960997
C	4.943391	-2.770852	-3.244660
C	4.630822	-1.835574	-2.257893
C	3.291889	-1.529786	-1.976009
C	2.282627	-2.182515	-2.703162
C	2.607826	-3.110382	-3.678818
H	4.193766	-4.148135	-4.722255
H	5.987216	-2.989462	-3.452014
H	5.432689	-1.317188	-1.739978
H	1.251460	-1.933906	-2.478975
H	1.805902	-3.604142	-4.221105
N	2.914088	-0.581296	-1.041000
H	1.911000	-0.530355	-0.855003
C	3.769015	-0.250448	0.056023
H	4.732798	0.132252	-0.302738
C	3.137711	0.844159	0.891637
O	1.944303	0.949854	1.090576
O	4.045176	1.606992	1.485275
C	3.547592	2.424764	2.553229
H	4.364854	3.093970	2.815349
H	2.675321	2.990832	2.223792
H	3.290396	1.776732	3.395460
C	4.903518	-0.968575	2.182439
O	4.396891	-0.749250	3.267374
C	6.351465	-0.660020	1.948406
C	7.057565	-1.114532	0.831274
C	7.007246	0.123190	2.903998
C	8.402117	-0.788341	0.672620
H	6.573221	-1.738960	0.086768

C	8.345980	0.452914	2.742641
H	6.440591	0.461084	3.765490
C	9.045528	-0.003053	1.624948
H	8.946420	-1.150417	-0.193559
H	8.848149	1.064544	3.485464
H	10.092926	0.252801	1.498362
C	4.047060	-1.467228	1.026794
C	2.774860	-2.152246	1.550443
H	4.609232	-2.192325	0.426229
H	3.084445	-2.762754	2.408750
H	2.077063	-1.416227	1.961268
C	2.069969	-3.043684	0.562148
H	2.704750	-3.650752	-0.086672
C	0.746693	-3.182437	0.491765
H	0.104050	-2.582676	1.135496
C	0.056989	-4.209443	-0.362825
H	0.760547	-4.704910	-1.040119
H	-0.386659	-4.986133	0.272994
C	-1.076840	-3.648608	-1.195806
F	-1.849121	-2.816131	-0.452209
F	-0.617588	-2.927772	-2.251029
C	-1.994318	-4.735623	-1.788202
O	-1.889800	-5.914102	-1.561229
O	-2.888113	-4.185777	-2.603871
C	-3.776663	-5.097536	-3.262702
H	-3.181937	-5.851407	-3.786958
H	-4.375011	-5.614038	-2.505725
C	-4.632760	-4.281240	-4.205754
H	-4.009584	-3.773520	-4.945671
H	-5.338813	-4.931754	-4.728511
H	-5.195562	-3.524109	-3.653885

27

M06-2X SCF energy: -1044.78285179 a.u.

M06-2X enthalpy: -1044.456479 a.u.

M06-2X free energy: -1044.533522 a.u.

M06-2X SCF energy in solution: -1045.13296221 a.u.

M06-2X enthalpy in solution: -1044.806589 a.u.

M06-2X free energy in solution: -1044.883632 a.u.

Cartesian coordinates

ATOM X Y Z

C	-1.881354	-2.580370	-1.143465
C	-0.691984	-1.673423	-1.183433
C	-1.408206	-1.975166	0.148942
H	-2.824552	-2.251844	-1.562631
H	-1.691124	-3.642805	-1.262352
H	-0.836695	-0.657150	-1.540295
H	-0.858587	-2.635885	0.811866
C	-2.100413	-0.924030	0.953237
O	-1.988537	-0.990050	2.163428
C	-2.900766	0.213483	0.361257
C	-3.190765	0.400863	-0.995351
C	-3.392473	1.146853	1.283485
C	-3.950327	1.491551	-1.413225
H	-2.837547	-0.287878	-1.751178
C	-4.147225	2.234680	0.867374
H	-3.164621	0.991289	2.331889
C	-4.429711	2.410574	-0.486212
H	-4.166318	1.618964	-2.469216
H	-4.516871	2.946946	1.598236
H	-5.020727	3.259385	-0.815981
C	0.656861	-2.207513	-1.358370
H	0.807473	-3.273232	-1.216445
C	1.837838	-1.303735	-1.235871
H	1.730200	-0.404532	-1.854639
H	2.760321	-1.814052	-1.530892
C	2.011573	-0.820581	0.205758
F	0.951472	-0.044523	0.555430
F	2.044450	-1.875151	1.046633
C	3.300204	-0.012531	0.400655
O	4.290879	-0.444292	0.924106
O	3.173728	1.193898	-0.151081
C	4.339963	2.031890	-0.063726
C	4.000593	3.342836	-0.736953
H	5.175185	1.517963	-0.548514
H	4.594566	2.160812	0.991935
H	4.859602	4.017147	-0.691250
H	3.740872	3.181436	-1.786098
H	3.154122	3.821763	-0.239226

B

M06-2X SCF energy: -553.90894305 a.u.

M06-2X enthalpy: -553.717835 a.u.

M06-2X free energy: -553.768885 a.u.
M06-2X SCF energy in solution: -554.09435254 a.u.
M06-2X enthalpy in solution: -553.903244 a.u.
M06-2X free energy in solution: -553.954294 a.u.

Cartesian coordinates

ATOM	X	Y	Z
C	-4.087434	-0.267378	-0.000087
C	-3.208484	-1.347493	-0.000161
C	-1.832412	-1.150969	-0.000103
C	-1.318301	0.152208	0.000044
C	-2.201867	1.241224	0.000131
C	-3.572208	1.028424	0.000061
H	-5.159286	-0.432100	-0.000172
H	-3.595450	-2.361681	-0.000275
H	-1.167310	-2.006808	-0.000162
H	-1.801925	2.251695	0.000270
H	-4.242371	1.882168	0.000149
N	0.046795	0.410913	0.000088
H	0.350367	1.382272	-0.000017
C	1.078168	-0.467550	0.000200
H	0.908099	-1.532358	0.000634
C	2.387862	0.115301	0.000045
O	2.592580	1.323115	-0.000282
O	3.373221	-0.808862	0.000265
C	4.686961	-0.263905	-0.000153
H	5.362867	-1.118060	-0.000016
H	4.848495	0.352356	-0.887968
H	4.848834	0.352922	0.887210

C

M06-2X SCF energy: -553.68123613 a.u.
M06-2X enthalpy: -553.488112 a.u.
M06-2X free energy: -553.538301 a.u.
M06-2X SCF energy in solution: -553.93335819 a.u.
M06-2X enthalpy in solution: -553.740234 a.u.
M06-2X free energy in solution: -553.790423 a.u.

Cartesian coordinates

ATOM	X	Y	Z
C	4.070684	-0.195914	0.000224
C	3.249705	-1.328164	0.000199

C	1.870762	-1.193141	0.000081
C	1.331112	0.096404	-0.000016
C	2.136787	1.237013	0.000013
C	3.516904	1.082574	0.000134
H	5.148661	-0.315910	0.000320
H	3.689503	-2.318980	0.000282
H	1.240808	-2.076322	0.000086
H	1.691166	2.228292	-0.000062
H	4.155810	1.958130	0.000159
N	-0.068199	0.315085	-0.000147
H	-0.378272	1.300651	-0.000102
C	-1.050605	-0.508814	-0.000378
H	-0.905867	-1.583721	-0.000528
C	-2.416056	0.136204	-0.000585
O	-2.513756	1.337673	0.000141
O	-3.363041	-0.764757	-0.000265
C	-4.717479	-0.251952	0.000378
H	-5.354842	-1.131499	0.000608
H	-4.872647	0.352533	0.894642
H	-4.873432	0.352642	-0.893675

D

M06-2X SCF energy: -505.30406938 a.u.

M06-2X enthalpy: -505.202275 a.u.

M06-2X free energy: -505.246930 a.u.

M06-2X SCF energy in solution: -505.48119801 a.u.

M06-2X enthalpy in solution: -505.379404 a.u.

M06-2X free energy in solution: -505.424059 a.u.

Cartesian coordinates

ATOM	X	Y	Z
C	1.521341	-0.270563	-0.128139
F	1.541447	-1.568443	0.044806
F	2.685644	0.301034	0.028833
C	0.306829	0.512901	-0.013513
O	0.305819	1.724016	0.017365
O	-0.775815	-0.277346	-0.013104
C	-2.027762	0.426193	0.020949
C	-3.124170	-0.615445	-0.004391
H	-2.075026	1.099457	-0.840169
H	-2.061039	1.040176	0.925771
H	-4.101439	-0.126601	0.021239

H	-3.060044	-1.219032	-0.912929
H	-3.043742	-1.279192	0.859810

E

M06-2X SCF energy: -1044.79796451 a.u.

M06-2X enthalpy: -1044.472771 a.u.

M06-2X free energy: -1044.548455 a.u.

M06-2X SCF energy in solution: -1045.14853636 a.u.

M06-2X enthalpy in solution: -1044.823343 a.u.

M06-2X free energy in solution: -1044.899027 a.u.

Imaginary frequency: -5.4020 cm-1

Cartesian coordinates

ATOM	X	Y	Z
C	3.673167	-1.038588	-0.036921
O	4.124145	-2.078083	-0.527819
C	4.500132	0.213781	-0.042286
C	3.965456	1.484598	0.186469
C	5.865697	0.084157	-0.311786
C	4.787828	2.607291	0.157017
H	2.902534	1.613333	0.365016
C	6.687613	1.203876	-0.333064
H	6.259251	-0.908607	-0.502828
C	6.149769	2.468371	-0.096715
H	4.363086	3.591201	0.328751
H	7.748186	1.093438	-0.535874
H	6.790507	3.344510	-0.115102
C	2.364392	-1.031561	0.573675
C	1.492131	-2.240988	0.547818
H	1.990269	-0.144032	1.071568
H	2.045579	-3.055081	0.069980
H	1.249626	-2.545389	1.574741
C	0.210052	-1.951339	-0.193437
H	0.313289	-1.724280	-1.254557
C	-0.996142	-1.937272	0.367774
H	-1.101063	-2.155118	1.429862
C	-2.262498	-1.636796	-0.379951
H	-2.062787	-1.423151	-1.433735
H	-2.959327	-2.481615	-0.345480
C	-2.976600	-0.444414	0.217937
F	-3.257062	-0.677377	1.529358
F	-2.186122	0.657959	0.173691

C	-4.297494	-0.105595	-0.490078
O	-4.721317	-0.719327	-1.435597
O	-4.883680	0.937793	0.081726
C	-6.133431	1.341887	-0.506434
H	-5.958695	1.584836	-1.558481
H	-6.824244	0.494704	-0.468596
C	-6.635114	2.529718	0.283445
H	-5.917620	3.352328	0.237189
H	-7.587511	2.873558	-0.128151
H	-6.785353	2.258284	1.331005

E'

M06-2X SCF energy: -1044.79114618 a.u.
M06-2X enthalpy: -1044.464891 a.u.
M06-2X free energy: -1044.541774 a.u.
M06-2X SCF energy in solution: -1045.14430146 a.u.
M06-2X enthalpy in solution: -1044.818046 a.u.
M06-2X free energy in solution: -1044.894929 a.u.

Cartesian coordinates

ATOM	X	Y	Z
C	0.708601	-0.108394	0.671080
C	0.270161	-1.435805	1.244980
C	1.662138	0.585312	1.590129
H	1.156600	-0.264061	-0.315645
H	-0.164968	0.533985	0.510667
H	1.072332	-2.141280	1.458405
H	1.272588	0.945793	2.538147
C	3.031447	0.973164	1.340962
O	3.558211	1.806663	2.084102
C	3.806526	0.420823	0.181083
C	3.664631	-0.896093	-0.262721
C	4.758558	1.248378	-0.420044
C	4.448204	-1.372589	-1.310273
H	2.961835	-1.563800	0.228047
C	5.530239	0.778059	-1.475621
H	4.881150	2.257359	-0.039751
C	5.373927	-0.532958	-1.924033
H	4.338778	-2.399809	-1.642968
H	6.259558	1.429940	-1.945992
H	5.979888	-0.902415	-2.745438
C	-0.979681	-1.809430	1.519450

H	-1.149594	-2.809546	1.908187
C	-2.208065	-0.970538	1.286414
H	-2.021671	0.093683	1.455772
H	-3.017169	-1.275892	1.957514
C	-2.715417	-1.125862	-0.139357
F	-1.752499	-0.727620	-1.014450
F	-2.995357	-2.419170	-0.397653
C	-3.980278	-0.298783	-0.407666
O	-5.077953	-0.768067	-0.532005
O	-3.679924	0.999620	-0.428190
C	-4.795111	1.883972	-0.644727
C	-4.255571	3.296547	-0.633286
H	-5.532501	1.712661	0.144839
H	-5.260804	1.624435	-1.599472
H	-5.070467	4.006157	-0.797417
H	-3.786792	3.522742	0.327578
H	-3.512592	3.430596	-1.423028

Ir^{II}

M06-2X SCF energy: -2941.95808357 a.u.

M06-2X enthalpy: -2941.214647 a.u.

M06-2X free energy: -2941.356765 a.u.

M06-2X SCF energy in solution: -2942.51445239 a.u.

M06-2X enthalpy in solution: -2941.771016 a.u.

M06-2X free energy in solution: -2941.913134 a.u.

Cartesian coordinates

ATOM	X	Y	Z
Ir	-0.827283	-0.002767	-0.000106
C	2.107594	-0.161415	-0.692573
C	0.824699	-0.633879	-2.605422
C	3.288232	-0.350896	-1.470858
C	1.937490	-0.822222	-3.389207
H	-0.176342	-0.740459	-3.016885
C	3.230932	-0.678132	-2.802166
H	4.247552	-0.230369	-0.984733
H	1.814283	-1.077049	-4.435086
C	2.106694	0.177179	0.691025
C	0.821326	0.641251	2.604269
C	3.286309	0.374600	1.468883
C	1.933109	0.837142	3.387642
H	-0.180263	0.741144	3.016075

C	3.227279	0.701611	2.800166
H	4.246248	0.260399	0.982453
H	1.808565	1.091292	4.433529
N	0.875481	-0.321358	-1.299509
N	0.873742	0.328873	1.298388
C	4.470994	-0.890301	-3.665667
C	4.452430	0.117698	-4.827598
C	5.769554	-0.697847	-2.877708
C	4.451496	-2.321038	-4.230593
H	3.560778	-0.000573	-5.450385
H	4.466423	1.144680	-4.448455
H	5.331410	-0.026983	-5.465593
H	5.845316	-1.409807	-2.049014
H	6.627107	-0.860353	-3.538422
H	5.846324	0.316584	-2.472229
H	5.331206	-2.488490	-4.861966
H	4.463352	-3.057079	-3.420201
H	3.560614	-2.502917	-4.838989
C	4.466214	0.922083	3.663191
C	5.765705	0.738041	2.874764
C	4.437519	2.352768	4.227865
C	4.454680	-0.085812	4.825308
H	5.849003	-0.275932	2.469427
H	5.836455	1.450339	2.045916
H	6.622422	0.906311	3.535121
H	3.545621	2.528950	4.836449
H	5.316273	2.526073	4.858994
H	4.444377	3.088722	3.417336
H	5.332834	0.064834	5.463058
H	3.562417	0.026631	5.448299
H	4.475418	-1.112751	4.446358
C	-1.812420	2.171926	-1.689420
C	-0.131973	2.918313	-0.231265
C	-1.935562	3.465731	-2.218333
C	-0.214807	4.208786	-0.724705
H	0.559199	2.644159	0.559044
C	-1.135877	4.484442	-1.733183
H	-2.656697	3.657281	-2.998371
H	-1.226554	5.491773	-2.126788
C	-2.258271	-0.209754	-1.379329
C	-2.944381	-1.379453	-1.717680
C	-2.569684	0.985093	-2.084544
C	-3.898512	-1.353568	-2.722684
H	-2.752721	-2.320264	-1.210910

C	-3.542251	0.950373	-3.088557
C	-4.221389	-0.202136	-3.430526
H	-4.970128	-0.203051	-4.211678
C	-2.259073	0.192798	1.379960
C	-2.560132	-1.004429	2.085663
C	-2.954647	1.356841	1.718482
C	-3.532306	-0.977522	3.090293
C	-3.907869	1.323293	2.724127
H	-2.771174	2.299100	1.211370
C	-4.220729	0.169406	3.432453
H	-4.968933	0.164306	4.214101
C	-1.793318	-2.185055	1.690344
C	-0.107850	-2.918056	0.231141
C	-1.905140	-3.479632	2.220073
C	-0.179397	-4.208826	0.725314
H	0.580428	-2.638429	-0.559757
C	-1.097318	-4.491737	1.734763
H	-2.623923	-3.676792	3.000885
H	-1.179019	-5.499585	2.129059
N	-0.911710	1.937150	-0.696714
N	-0.895500	-1.943240	0.696802
F	-3.850512	2.068230	-3.771816
F	-4.544572	-2.482439	-3.035935
F	-4.563056	2.446850	3.037518
F	-3.830867	-2.097709	3.774033
C	0.750241	-5.268881	0.214279
F	1.823124	-5.404496	1.005261
F	1.193209	-4.985337	-1.015765
F	0.141690	-6.462409	0.172070
C	0.706773	5.275711	-0.213299
F	1.144695	4.999163	1.020205
F	1.783288	5.413498	-0.998922
F	0.091966	6.466245	-0.178952

***Ir^{III}**

M06-2X SCF energy: -2941.69539001 a.u.

M06-2X enthalpy: -2940.951371 a.u.

M06-2X free energy: -2941.096273 a.u.

M06-2X SCF energy in solution: -2942.29546113 a.u.

M06-2X enthalpy in solution: -2941.551442 a.u.

M06-2X free energy in solution: -2941.696344 a.u.

Cartesian coordinates

ATOM	X	Y	Z
Ir	-0.869051	-0.165306	0.004741
C	2.049044	0.217596	-0.707244
C	0.853963	-0.505208	-2.607770
C	3.237935	0.222621	-1.492149
C	1.976793	-0.504450	-3.387618
H	-0.107397	-0.796233	-3.019572
C	3.234618	-0.131188	-2.817018
H	4.162833	0.517370	-1.014444
H	1.894145	-0.793581	-4.428289
C	1.990008	0.586543	0.662681
C	0.637331	0.859194	2.574992
C	3.112538	1.008891	1.431353
C	1.695357	1.267735	3.338177
H	-0.360549	0.795445	2.997960
C	2.998271	1.353098	2.753701
H	4.076807	1.055078	0.942976
H	1.526294	1.524784	4.376915
N	0.858119	-0.171074	-1.293582
N	0.745795	0.530572	1.263990
C	4.486069	-0.143630	-3.687310
C	4.287092	0.827098	-4.864774
C	5.735105	0.281302	-2.910249
C	4.704356	-1.567922	-4.228057
H	3.431199	0.545424	-5.485559
H	4.126513	1.848489	-4.505121
H	5.176839	0.823973	-5.502597
H	5.937195	-0.392957	-2.071178
H	6.604373	0.254509	-3.573685
H	5.642981	1.302501	-2.525215
H	5.597833	-1.592698	-4.860218
H	4.844763	-2.279311	-3.407881
H	3.858363	-1.908032	-4.832959
C	4.173549	1.815477	3.606930
C	5.483926	1.850065	2.815685
C	3.880817	3.231134	4.134775
C	4.340667	0.849841	4.793560
H	5.753384	0.858036	2.437754
H	5.426334	2.543813	1.970116
H	6.294803	2.188255	3.467284
H	2.973575	3.258595	4.745904
H	4.713051	3.574784	4.757517
H	3.756621	3.937821	3.307987

H	5.176720	1.173864	5.421705
H	3.445035	0.817501	5.421337
H	4.548601	-0.166263	4.443215
C	-2.184701	1.704180	-1.806484
C	-0.768851	2.831487	-0.308792
C	-2.533412	2.915600	-2.411386
C	-1.081025	4.054719	-0.877218
H	-0.077933	2.736844	0.522716
C	-1.979157	4.095812	-1.939921
H	-3.227766	2.926294	-3.238393
H	-2.243037	5.046405	-2.392922
C	-2.122635	-0.723483	-1.432595
C	-2.491625	-2.032975	-1.745725
C	-2.650435	0.364789	-2.181589
C	-3.392734	-2.251075	-2.779622
H	-2.101480	-2.887922	-1.203690
C	-3.553954	0.083697	-3.205382
C	-3.936797	-1.210669	-3.522544
H	-4.639184	-1.398955	-4.325527
C	-2.198730	-0.147695	1.481729
C	-2.217731	-1.349355	2.243215
C	-3.057434	0.902907	1.810311
C	-3.126995	-1.448221	3.295812
C	-3.938620	0.747224	2.872355
H	-3.063916	1.837200	1.258884
C	-3.991958	-0.416862	3.628801
H	-4.686349	-0.521284	4.453728
C	-1.262429	-2.390573	1.848952
C	0.437292	-2.866840	0.299044
C	-1.073676	-3.633623	2.459527
C	0.664126	-4.106932	0.873393
H	1.001754	-2.512746	-0.557762
C	-0.105674	-4.495738	1.964854
H	-1.678069	-3.915819	3.308746
H	0.049516	-5.468048	2.421427
N	-1.317641	1.698879	-0.760484
N	-0.505052	-2.046725	0.773609
F	-4.084334	1.074565	-3.927212
F	-3.751304	-3.494659	-3.075243
F	-4.762324	1.741162	3.182124
F	-3.188815	-2.561975	4.030818
C	1.761483	-4.990865	0.341006
F	2.897882	-4.796029	1.013889
F	1.999700	-4.731534	-0.948591

F	1.430467	-6.277861	0.462162
C	-0.430984	5.316113	-0.372238
F	0.082004	5.136700	0.848409
F	0.558013	5.697088	-1.183634
F	-1.317977	6.311979	-0.317383

TS-1

M06-2X SCF energy: -1044.73263311 a.u.

M06-2X enthalpy: -1044.408373 a.u.

M06-2X free energy: -1044.484186 a.u.

M06-2X SCF energy in solution: -1045.08044691 a.u.

M06-2X enthalpy in solution: -1044.756187 a.u.

M06-2X free energy in solution: -1044.832000 a.u.

Imaginary frequency: -417.5743 cm-1

Cartesian coordinates

ATOM	X	Y	Z
C	0.245368	0.161320	-0.357168
C	0.474871	-1.090265	0.443699
C	0.941928	0.269429	0.966636
H	0.820081	0.324148	-1.261197
H	-0.776323	0.526557	-0.407047
H	1.262430	-1.756131	0.110510
H	0.348230	0.655881	1.789742
C	2.376898	0.641967	1.169498
O	2.638868	1.307809	2.154422
C	3.500664	0.244036	0.241966
C	3.373157	-0.505582	-0.933747
C	4.777315	0.681079	0.620407
C	4.493248	-0.807270	-1.705195
H	2.412785	-0.868958	-1.274227
C	5.893089	0.380278	-0.148072
H	4.865725	1.260921	1.532150
C	5.753838	-0.366915	-1.316539
H	4.375001	-1.389343	-2.613499
H	6.873069	0.727771	0.163173
H	6.623911	-0.604357	-1.920692
C	-0.655150	-1.764845	1.118796
H	-0.576353	-2.843840	1.221573
C	-1.783977	-1.148162	1.541775
H	-1.850297	-0.061364	1.567121
H	-2.531044	-1.685827	2.116821

C	-3.134389	-1.284822	-0.300896
F	-2.278598	-1.374102	-1.303993
F	-3.776314	-2.417886	-0.098735
C	-3.907881	-0.049361	-0.140253
O	-4.942264	0.014803	0.481826
O	-3.233773	0.998185	-0.643544
C	-3.833027	2.280219	-0.393148
C	-2.894897	3.326082	-0.953493
H	-3.984071	2.391900	0.684642
H	-4.816088	2.305961	-0.871999
H	-3.311406	4.323874	-0.793600
H	-1.920750	3.274124	-0.459938
H	-2.749660	3.177078	-2.026211

TS-2

M06-2X SCF energy: -1044.77124225 a.u.

M06-2X enthalpy: -1044.446468 a.u.

M06-2X free energy: -1044.521100 a.u.

M06-2X SCF energy in solution: -1045.12550841 a.u.

M06-2X enthalpy in solution: -1044.800734 a.u.

M06-2X free energy in solution: -1044.875366 a.u.

Imaginary frequency: -593.3506 cm-1

Cartesian coordinates

ATOM	X	Y	Z
C	-1.774564	-2.436737	-1.099991
C	-0.551246	-1.604556	-1.300164
C	-1.528144	-1.941589	0.276406
H	-2.685690	-2.104148	-1.589894
H	-1.608775	-3.505853	-1.225905
H	-0.682916	-0.542280	-1.486034
H	-0.892497	-2.545254	0.914856
C	-2.096055	-0.799001	0.980739
O	-1.806113	-0.663664	2.168076
C	-3.071976	0.173336	0.362540
C	-3.236963	0.423500	-1.004582
C	-3.857376	0.895088	1.271015
C	-4.167904	1.361381	-1.447082
H	-2.636029	-0.082024	-1.749735
C	-4.792900	1.821116	0.830262
H	-3.708187	0.709811	2.328847
C	-4.952744	2.057183	-0.534196

H	-4.274645	1.547544	-2.511137
H	-5.396817	2.363176	1.551204
H	-5.681167	2.782717	-0.882634
C	0.731041	-2.109063	-1.296068
H	0.886043	-3.174905	-1.150355
C	1.928335	-1.215688	-1.246751
H	1.762171	-0.282315	-1.794875
H	2.817132	-1.705153	-1.657934
C	2.231854	-0.834814	0.202748
F	1.184027	-0.144103	0.715381
F	2.397161	-1.950096	0.945108
C	3.501020	0.016972	0.331364
O	4.554808	-0.403672	0.724216
O	3.271514	1.252084	-0.112366
C	4.403580	2.139173	-0.068246
C	3.943156	3.478935	-0.597342
H	5.208275	1.706903	-0.670267
H	4.754853	2.199488	0.965560
H	4.772499	4.190618	-0.578121
H	3.588915	3.385876	-1.626960
H	3.129781	3.874189	0.015670

TS-2’

M06-2X SCF energy: -1044.77230143 a.u.
M06-2X enthalpy: -1044.447414 a.u.
M06-2X free energy: -1044.521481 a.u.
M06-2X SCF energy in solution: -1045.12558724 a.u.
M06-2X enthalpy in solution: -1044.800700 a.u.
M06-2X free energy in solution: -1044.874767 a.u.
Imaginary frequency: -591.0450 cm-1

Cartesian coordinates

ATOM	X	Y	Z
C	0.646424	0.077042	-0.572181
C	0.573837	-1.080177	0.372126
C	1.230806	0.661644	0.655615
H	1.314910	-0.042630	-1.420483
H	-0.317489	0.480921	-0.873198
H	1.435102	-1.737835	0.416763
H	0.551207	1.143907	1.350539
C	2.625673	0.842302	1.037610
O	2.857099	1.469967	2.070499

C	3.790641	0.376184	0.200717
C	3.771330	-0.673599	-0.724321
C	4.995704	1.058813	0.411560
C	4.923942	-1.020840	-1.426177
H	2.875698	-1.256110	-0.900336
C	6.140972	0.721452	-0.296819
H	5.006424	1.855311	1.147292
C	6.108228	-0.321241	-1.221405
H	4.892210	-1.844434	-2.132410
H	7.062567	1.268863	-0.125830
H	7.002710	-0.590368	-1.774414
C	-0.529734	-1.426974	1.127269
H	-0.465145	-2.298706	1.768194
C	-1.816575	-0.662146	1.124181
H	-1.662796	0.407572	0.942767
H	-2.332933	-0.763274	2.084557
C	-2.768320	-1.171513	0.046632
F	-2.180383	-1.060284	-1.175840
F	-3.047396	-2.473785	0.250229
C	-4.091106	-0.394283	0.013823
O	-5.144094	-0.838847	0.379573
O	-3.878217	0.844800	-0.429442
C	-5.044807	1.687435	-0.480016
C	-4.599910	3.038717	-0.992420
H	-5.476097	1.743225	0.523594
H	-5.782117	1.216014	-1.135712
H	-5.457548	3.713695	-1.050550
H	-3.856458	3.479430	-0.323679
H	-4.161169	2.946786	-1.988795

(*R*,*S*)-**TS-3**

M06-2X SCF energy: -4086.65666134 a.u.

M06-2X enthalpy: -4085.455531 a.u.

M06-2X free energy: -4085.643117 a.u.

M06-2X SCF energy in solution: -4087.84984743 a.u.

M06-2X enthalpy in solution: -4086.648717 a.u.

M06-2X free energy in solution: -4086.836303 a.u.

Imaginary frequency: -400.4692 cm-1

Cartesian coordinates

ATOM	X	Y	Z
C	-6.911731	2.537697	-3.082257

C	-6.199062	1.778914	-2.188867
C	-5.125610	2.344872	-1.448869
C	-4.781087	3.705912	-1.688640
C	-5.546391	4.466973	-2.611716
C	-6.592802	3.900485	-3.290178
H	-7.726634	2.087205	-3.639554
H	-6.449963	0.733549	-2.047048
C	-4.352955	1.593272	-0.503265
C	-3.661051	4.276582	-1.031112
H	-5.277215	5.506858	-2.773726
H	-7.170962	4.488364	-3.995234
C	-2.878053	3.545340	-0.172220
C	-3.259418	2.202066	0.071194
H	-3.402147	5.312944	-1.232840
C	-4.638064	0.166470	-0.189755
C	-5.900040	-0.282985	0.320267
C	-3.640714	-0.768525	-0.363518
C	-6.948351	0.613797	0.660404
C	-6.101195	-1.676340	0.534292
C	-3.821781	-2.161546	-0.178952
C	-8.138679	0.143618	1.153947
H	-6.794944	1.679988	0.534055
C	-7.349840	-2.131753	1.034232
C	-5.052944	-2.589953	0.253580
C	-8.349252	-1.244239	1.334262
H	-8.926596	0.843022	1.413592
H	-7.492077	-3.198740	1.180599
H	-5.228982	-3.653973	0.390801
H	-9.299297	-1.599549	1.719204
P	-1.498444	0.357844	0.372524
O	-0.404442	1.088471	-0.480611
O	-1.028687	-0.505392	1.486163
C	-2.741751	-3.133822	-0.519459
C	-2.490781	-3.427550	-1.875559
C	-2.042333	-3.810133	0.496390
C	-1.552251	-4.459167	-2.215710
C	-3.163300	-2.751489	-2.944532
C	-1.094271	-4.832465	0.143890
C	-2.239634	-3.525137	1.889024
C	-1.347506	-4.790423	-3.593075
C	-0.873534	-5.134480	-1.199882
C	-2.921794	-3.078602	-4.246485
H	-3.877445	-1.970278	-2.707123
C	-0.414596	-5.543678	1.187596

C	-1.584383	-4.235341	2.852864
H	-2.922773	-2.728954	2.165226
C	-2.005323	-4.119528	-4.579744
H	-0.658928	-5.597403	-3.831590
H	-0.173657	-5.926451	-1.460858
H	-3.440673	-2.552474	-5.041026
H	0.274718	-6.335821	0.904277
C	-0.665939	-5.269562	2.500173
H	-1.749751	-4.007671	3.901635
H	-1.846392	-4.379950	-5.621140
H	-0.172350	-5.834649	3.285007
C	-1.663656	4.119159	0.475368
C	-0.491906	4.307173	-0.282336
C	-1.703224	4.473397	1.837416
C	0.667223	4.879148	0.344937
C	-0.390238	3.924589	-1.661017
C	-0.539630	5.046205	2.452170
C	-2.873219	4.293877	2.643881
C	1.859394	5.062014	-0.426580
C	0.613830	5.237929	1.691217
C	0.767627	4.106121	-2.357702
H	-1.249542	3.469052	-2.141303
C	-0.590566	5.411882	3.834404
C	-2.881075	4.652717	3.959582
H	-3.764368	3.871911	2.190341
C	1.912096	4.689070	-1.735266
H	2.722414	5.507053	0.061316
H	1.492260	5.676912	2.159543
H	0.823979	3.807230	-3.399994
H	0.296691	5.850309	4.283142
C	-1.722845	5.221245	4.567716
H	-3.778480	4.511447	4.553354
H	2.820561	4.832470	-2.311345
H	-1.753206	5.505574	5.614546
H	0.518876	0.961130	-0.141111
O	-2.374423	-0.345911	-0.769737
O	-2.478803	1.472227	0.966630
C	1.360756	-2.745353	5.861662
C	1.973839	-3.668124	5.010504
C	1.826792	-3.557943	3.636100
C	1.058545	-2.508090	3.114625
C	0.429522	-1.585255	3.959997
C	0.590852	-1.710139	5.333989
H	1.479327	-2.841272	6.935632

H	2.559627	-4.482643	5.422543
H	2.273666	-4.302936	2.986218
H	-0.176374	-0.794859	3.528361
H	0.109501	-0.997131	5.994206
N	0.868846	-2.335954	1.732425
H	0.133013	-1.618806	1.458045
C	1.542473	-2.916155	0.774804
H	2.203416	-3.749772	0.984749
C	1.070310	-2.679020	-0.633453
O	0.201455	-1.887042	-0.896013
O	1.801556	-3.390770	-1.477341
C	1.544704	-3.216843	-2.884760
H	0.596985	-2.700053	-3.034616
H	2.373817	-2.643723	-3.305598
H	1.510304	-4.216673	-3.315695
C	2.749328	-0.507215	-0.221286
O	1.966703	0.215812	0.398728
C	3.007382	-0.386454	-1.674559
C	4.234160	-0.807406	-2.206239
C	2.035032	0.175097	-2.513448
C	4.483849	-0.672791	-3.567490
H	5.015288	-1.192172	-1.558790
C	2.285074	0.288335	-3.875355
H	1.078943	0.490716	-2.110629
C	3.506070	-0.134007	-4.402844
H	5.443012	-0.980037	-3.970534
H	1.525906	0.707310	-4.527444
H	3.698430	-0.036554	-5.466655
C	3.417776	-1.595051	0.526735
C	3.853576	-1.330910	1.929679
H	3.971875	-2.338444	-0.040273
H	3.084427	-0.783747	2.481096
H	4.098725	-2.255967	2.459308
C	5.092667	-0.477919	1.729629
H	4.926988	0.592273	1.618107
C	6.310985	-0.993017	1.559619
H	6.456450	-2.069788	1.633701
C	7.504254	-0.165123	1.171160
H	7.485496	0.823615	1.637382
H	8.440450	-0.656634	1.450856
C	7.500756	0.051860	-0.336754
F	7.368719	-1.133100	-0.981132
F	6.421953	0.810236	-0.677420
C	8.763377	0.745290	-0.867706

O	9.515485	0.246238	-1.657212
O	8.886600	1.942482	-0.304832
C	10.040736	2.705842	-0.721692
H	10.936930	2.120989	-0.497650
H	9.987117	2.839857	-1.805292
C	10.004445	4.019053	0.025524
H	10.046296	3.851938	1.104530
H	10.863120	4.630092	-0.263085
H	9.091369	4.571378	-0.208570

(*R,R*)-**TS-3**

M06-2X SCF energy: -4086.66306199 a.u.

M06-2X enthalpy: -4085.462779 a.u.

M06-2X free energy: -4085.644800 a.u.

M06-2X SCF energy in solution: -4087.85375242 a.u.

M06-2X enthalpy in solution: -4086.653469 a.u.

M06-2X free energy in solution: -4086.835490 a.u.

Imaginary frequency: -385.1032 cm-1

Cartesian coordinates

ATOM	X	Y	Z
C	-5.378320	5.403533	-1.376625
C	-4.920377	4.234739	-0.823476
C	-3.543246	4.066567	-0.514937
C	-2.643461	5.123932	-0.836979
C	-3.152060	6.325408	-1.397878
C	-4.489559	6.468165	-1.656696
H	-6.433149	5.511290	-1.607395
H	-5.612130	3.423897	-0.624029
C	-3.020564	2.864954	0.068074
C	-1.248524	4.953612	-0.637312
H	-2.453970	7.125620	-1.626822
H	-4.868857	7.388488	-2.088046
C	-0.731169	3.782061	-0.143106
C	-1.652802	2.764685	0.214458
H	-0.573949	5.759616	-0.914990
C	-3.882366	1.700409	0.429180
C	-4.981333	1.779191	1.346958
C	-3.591483	0.465873	-0.108288
C	-5.300410	2.968133	2.055724
C	-5.761335	0.611172	1.591217
C	-4.339206	-0.716158	0.124383

C	-6.358489	3.001491	2.928359
H	-4.692002	3.853187	1.904288
C	-6.857637	0.683762	2.490937
C	-5.426548	-0.614679	0.956554
C	-7.154896	1.851827	3.142359
H	-6.585639	3.917813	3.463348
H	-7.451468	-0.210588	2.657299
H	-6.037650	-1.492795	1.150501
H	-7.992851	1.896763	3.829967
P	-1.091431	0.284625	-0.158405
O	-0.097156	0.533350	-1.333052
O	-0.874434	-0.864069	0.757584
C	-3.943240	-1.990216	-0.548940
C	-4.122112	-2.117371	-1.942505
C	-3.413988	-3.060586	0.199032
C	-3.779208	-3.349569	-2.593859
C	-4.663930	-1.062945	-2.746133
C	-3.073043	-4.290352	-0.465511
C	-3.201742	-2.987212	1.616384
C	-3.992755	-3.478866	-4.003278
C	-3.259108	-4.404589	-1.842525
C	-4.841090	-1.219534	-4.089259
H	-4.942347	-0.128244	-2.271291
C	-2.568291	-5.388469	0.305864
C	-2.741865	-4.063577	2.318647
H	-3.420277	-2.055431	2.127313
C	-4.501932	-2.446079	-4.731843
H	-3.749695	-4.427681	-4.475846
H	-3.016771	-5.343896	-2.337450
H	-5.252709	-0.406689	-4.678375
H	-2.339014	-6.316113	-0.213476
C	-2.422107	-5.287888	1.657861
H	-2.600577	-3.988660	3.392608
H	-4.664586	-2.554544	-5.799202
H	-2.065537	-6.132485	2.239609
C	0.738380	3.538689	-0.017812
C	1.491469	3.214636	-1.164264
C	1.361568	3.622576	1.243160
C	2.895026	2.932305	-1.030728
C	0.910829	3.123488	-2.472749
C	2.773276	3.381490	1.356395
C	0.637076	3.950360	2.435245
C	3.653194	2.572525	-2.192133
C	3.501050	3.018455	0.222311

C	1.666892	2.784136	-3.554620
H	-0.150510	3.313647	-2.589120
C	3.400894	3.503310	2.636498
C	1.272078	4.052827	3.639913
H	-0.430682	4.132557	2.364707
C	3.060203	2.504613	-3.416310
H	4.711367	2.363094	-2.061573
H	4.560655	2.787805	0.311901
H	1.206074	2.720493	-4.535122
H	4.470874	3.324861	2.702148
C	2.678207	3.833161	3.744630
H	0.706076	4.312930	4.528746
H	3.641547	2.243445	-4.295378
H	3.165421	3.931385	4.709423
H	0.733720	-0.009914	-1.321513
O	-2.482258	0.340542	-0.941465
O	-1.116944	1.601231	0.757446
C	1.047796	-4.103195	4.779170
C	1.332747	-5.063274	3.805882
C	1.015838	-4.827563	2.475632
C	0.410302	-3.614215	2.126770
C	0.114559	-2.646714	3.096425
C	0.439708	-2.900265	4.421143
H	1.297360	-4.298347	5.816765
H	1.796293	-6.002489	4.087480
H	1.220325	-5.591592	1.732664
H	-0.346729	-1.711896	2.791003
H	0.222384	-2.152223	5.175960
N	0.090004	-3.293362	0.799733
H	-0.395145	-2.354904	0.653475
C	0.452244	-3.944848	-0.276328
H	0.826685	-4.960746	-0.216712
C	-0.086619	-3.439423	-1.583723
O	-0.617492	-2.367928	-1.710627
O	0.135330	-4.328573	-2.543182
C	-0.282953	-3.910957	-3.855082
H	-0.099245	-4.764021	-4.504240
H	-1.339529	-3.639918	-3.838592
H	0.306638	-3.046334	-4.168179
C	2.382489	-1.813021	-0.296973
O	1.946075	-1.115642	-1.212669
C	2.578641	-1.299484	1.074706
C	3.285256	-2.023108	2.047501
C	2.035697	-0.049751	1.405392

C	3.403154	-1.524554	3.338138
H	3.771374	-2.961011	1.801862
C	2.113645	0.424743	2.707321
H	1.559049	0.556772	0.641786
C	2.796496	-0.314072	3.673444
H	3.960299	-2.082656	4.083137
H	1.649770	1.372254	2.961827
H	2.871322	0.066656	4.687685
C	2.650163	-3.239348	-0.615385
C	3.075178	-3.553488	-2.018033
H	3.003452	-3.891080	0.178839
H	3.037192	-4.623901	-2.230946
H	2.453301	-3.011909	-2.734930
C	4.485564	-2.992409	-2.030377
H	5.266663	-3.598670	-1.574936
C	4.732503	-1.732729	-2.397003
H	3.924404	-1.124371	-2.797721
C	6.014370	-1.016906	-2.087831
H	6.881615	-1.684219	-2.090755
H	6.199566	-0.194567	-2.784632
C	5.886061	-0.442308	-0.676667
F	4.701820	0.217143	-0.554443
F	5.873033	-1.453384	0.222472
C	7.028799	0.513840	-0.312365
O	8.006909	0.176713	0.293964
O	6.789200	1.724319	-0.812821
C	7.809172	2.718662	-0.570815
H	7.949212	2.806789	0.509892
H	8.746792	2.361644	-1.005388
C	7.329373	4.006803	-1.200402
H	6.378880	4.323650	-0.762063
H	8.066845	4.796058	-1.035190
H	7.191004	3.883527	-2.277548

(*S,S*)-**TS-3**

M06-2X SCF energy: -4086.66114250 a.u.

M06-2X enthalpy: -4085.459975 a.u.

M06-2X free energy: -4085.646647 a.u.

M06-2X SCF energy in solution: -4087.84878873 a.u.

M06-2X enthalpy in solution: -4086.647621 a.u.

M06-2X free energy in solution: -4086.834293 a.u.

Imaginary frequency: -420.0591 cm-1

Cartesian coordinates

ATOM	X	Y	Z
C	5.082621	-4.011095	3.836611
C	4.579723	-3.249926	2.812306
C	3.389177	-2.494360	2.987164
C	2.712637	-2.572852	4.237787
C	3.266589	-3.359343	5.282236
C	4.427604	-4.060460	5.090090
H	5.989802	-4.586373	3.683435
H	5.085327	-3.227219	1.853082
C	2.825801	-1.693521	1.939943
C	1.490058	-1.877124	4.422125
H	2.744383	-3.399883	6.233898
H	4.842186	-4.662753	5.891530
C	0.910272	-1.159947	3.404948
C	1.597210	-1.107566	2.165035
H	0.995892	-1.925213	5.389266
C	3.494338	-1.496950	0.622197
C	4.806572	-0.931832	0.490722
C	2.800516	-1.780187	-0.531850
C	5.572546	-0.498798	1.606611
C	5.354196	-0.761019	-0.814069
C	3.294401	-1.556635	-1.841381
C	6.827172	0.028677	1.434402
H	5.154828	-0.584488	2.603472
C	6.660101	-0.221729	-0.956518
C	4.574907	-1.078027	-1.957844
C	7.385969	0.158492	0.141212
H	7.396102	0.354355	2.299141
H	7.066688	-0.108302	-1.957427
H	4.989667	-0.896839	-2.946099
H	8.382350	0.570960	0.022763
P	0.352790	-1.299333	-0.054816
O	-0.686688	-2.299855	0.564968
O	-0.120422	-0.406040	-1.135569
C	2.386323	-1.757934	-3.008510
C	2.135612	-3.054933	-3.494728
C	1.722553	-0.642585	-3.555771
C	1.218816	-3.236748	-4.584668
C	2.757036	-4.215718	-2.932063
C	0.781225	-0.841468	-4.623400
C	1.887265	0.687002	-3.041003
C	0.969101	-4.560080	-5.070243

C	0.568336	-2.126114	-5.125454
C	2.484797	-5.461823	-3.415308
H	3.455658	-4.088389	-2.111193
C	0.033413	0.279343	-5.109549
C	1.146217	1.725541	-3.518557
H	2.586460	0.851597	-2.227786
C	1.577569	-5.639938	-4.501993
H	0.283651	-4.681239	-5.905192
H	-0.141271	-2.269020	-5.938137
H	2.964728	-6.330152	-2.975855
H	-0.673323	0.112813	-5.918550
C	0.200836	1.519018	-4.568292
H	1.250326	2.713429	-3.081993
H	1.383782	-6.639325	-4.877986
H	-0.381015	2.360290	-4.931043
C	-0.364387	-0.407732	3.611717
C	-1.600660	-1.086246	3.638645
C	-0.309237	0.986213	3.823948
C	-2.808237	-0.345863	3.893043
C	-1.711087	-2.496730	3.405927
C	-1.517829	1.709538	4.105721
C	0.920114	1.720550	3.777793
C	-4.061803	-1.037117	3.899650
C	-2.736475	1.028681	4.128567
C	-2.926552	-3.116869	3.417129
H	-0.808990	-3.064100	3.204501
C	-1.447833	3.120052	4.340326
C	0.943782	3.066148	4.003087
H	1.843106	1.188163	3.573315
C	-4.120962	-2.379508	3.671668
H	-4.964471	-0.465898	4.101437
H	-3.650162	1.579458	4.343989
H	-2.987537	-4.185488	3.237687
H	-2.369725	3.649771	4.566875
C	-0.257009	3.781138	4.288935
H	1.887436	3.602534	3.975694
H	-5.076482	-2.894809	3.690275
H	-0.212179	4.849893	4.472990
H	-1.600213	-1.928390	0.650065
O	1.518316	-2.321589	-0.424579
O	0.974155	-0.404888	1.131736
C	-3.426011	-4.145384	-2.933769
C	-4.481155	-3.281903	-2.621440
C	-4.241128	-1.941078	-2.373029

C	-2.920249	-1.469031	-2.426336
C	-1.856353	-2.327044	-2.746677
C	-2.118696	-3.665617	-3.001353
H	-3.629266	-5.193006	-3.128912
H	-5.497757	-3.657084	-2.575394
H	-5.071168	-1.282700	-2.138467
H	-0.843235	-1.940277	-2.789503
H	-1.295419	-4.327727	-3.251375
N	-2.601941	-0.123500	-2.195432
H	-1.588032	0.098847	-2.214509
C	-3.397323	0.860992	-1.843851
H	-4.474140	0.735204	-1.883830
C	-2.790085	2.233501	-1.929939
O	-1.643096	2.402335	-2.248689
O	-3.678155	3.166538	-1.616989
C	-3.181659	4.518281	-1.683100
H	-2.344010	4.630127	-0.995668
H	-2.846607	4.737435	-2.698253
H	-4.023415	5.147734	-1.404539
C	-3.952580	-0.349422	0.557046
O	-3.189131	-1.314746	0.625445
C	-5.423722	-0.568509	0.490973
C	-6.335171	0.449689	0.181912
C	-5.889237	-1.874157	0.698700
C	-7.693682	0.162751	0.083323
H	-6.002714	1.468547	0.003571
C	-7.245787	-2.153726	0.609363
H	-5.164665	-2.649434	0.925343
C	-8.149492	-1.135383	0.300865
H	-8.395526	0.953188	-0.159893
H	-7.603647	-3.163984	0.778521
H	-9.209845	-1.354665	0.229224
C	-3.397354	1.005795	0.472217
C	-1.963673	1.219447	0.785753
H	-4.075075	1.852888	0.519352
H	-1.740948	0.613481	1.679863
H	-1.338393	0.767862	0.006901
C	-1.519493	2.637597	0.988675
H	-2.154807	3.298991	1.578969
C	-0.336443	3.053351	0.537360
H	0.280383	2.360555	-0.036831
C	0.243432	4.411567	0.788333
H	-0.493857	5.105915	1.202516
H	1.062145	4.347094	1.515119

C	0.823003	5.001998	-0.477711
F	1.783569	4.179311	-0.978573
F	-0.134983	5.107050	-1.442195
C	1.439230	6.394924	-0.272492
O	1.395569	6.980338	0.779160
O	1.994615	6.833001	-1.389749
C	2.604715	8.139086	-1.310149
H	1.837890	8.857990	-1.008587
H	3.368504	8.112972	-0.528162
C	3.182551	8.446763	-2.672468
H	2.397752	8.450713	-3.432451
H	3.657148	9.431004	-2.657635
H	3.933024	7.702844	-2.949541

(*S,R*)-**TS-3**

M06-2X SCF energy: -4086.66020738 a.u.

M06-2X enthalpy: -4085.458693 a.u.

M06-2X free energy: -4085.643056 a.u.

M06-2X SCF energy in solution: -4087.84457431 a.u.

M06-2X enthalpy in solution: -4086.643060 a.u.

M06-2X free energy in solution: -4086.827423 a.u.

Imaginary frequency: -464.3518 cm-1

Cartesian coordinates

ATOM	X	Y	Z
C	-7.104454	-0.393573	3.382508
C	-6.284994	-0.138565	2.311997
C	-5.135127	-0.937416	2.070953
C	-4.831254	-1.981898	2.991207
C	-5.706798	-2.227640	4.081655
C	-6.822882	-1.456539	4.272388
H	-7.976184	0.230053	3.551781
H	-6.508995	0.685680	1.644330
C	-4.248485	-0.710107	0.965082
C	-3.645792	-2.745815	2.831112
H	-5.466494	-3.035619	4.766837
H	-7.485140	-1.649125	5.109819
C	-2.761163	-2.494991	1.811793
C	-3.103556	-1.468376	0.895744
H	-3.425590	-3.533840	3.546953
C	-4.488074	0.359408	-0.045636
C	-5.691900	0.420660	-0.823091

C	-3.535949	1.335928	-0.253810
C	-6.667874	-0.611760	-0.796767
C	-5.908039	1.537558	-1.678746
C	-3.751070	2.486819	-1.057591
C	-7.803405	-0.521882	-1.561174
H	-6.501957	-1.482056	-0.170962
C	-7.099657	1.606736	-2.447603
C	-4.935959	2.569336	-1.745040
C	-8.029682	0.602343	-2.390133
H	-8.534736	-1.323060	-1.533621
H	-7.253843	2.470485	-3.088071
H	-5.137210	3.447542	-2.353163
H	-8.935740	0.660797	-2.983933
P	-1.295253	0.086147	0.007940
O	-0.503083	0.020898	1.373156
O	-0.518775	0.265927	-1.237916
C	-2.770512	3.614094	-1.039527
C	-2.979976	4.669372	-0.129785
C	-1.650437	3.616090	-1.891445
C	-2.047217	5.762575	-0.088375
C	-4.090938	4.699217	0.776337
C	-0.705992	4.697909	-1.818086
C	-1.385914	2.551838	-2.811304
C	-2.269623	6.835821	0.832239
C	-0.933910	5.748794	-0.929236
C	-4.263796	5.738452	1.642042
H	-4.799702	3.878135	0.765923
C	0.469907	4.651242	-2.631488
C	-0.243712	2.533211	-3.559069
H	-2.094955	1.734297	-2.884123
C	-3.343108	6.827693	1.670535
H	-1.560617	7.659184	0.842374
H	-0.221981	6.570339	-0.883276
H	-5.111684	5.741864	2.319333
H	1.176612	5.473624	-2.560825
C	0.702832	3.596376	-3.464457
H	-0.049087	1.698416	-4.224266
H	-3.504726	7.647019	2.363289
H	1.601732	3.568279	-4.072801
C	-1.490585	-3.262783	1.654272
C	-0.427022	-3.061412	2.557915
C	-1.370995	-4.201953	0.610896
C	0.773675	-3.840731	2.421128
C	-0.473487	-2.078812	3.601879

C	-0.165804	-4.971102	0.483229
C	-2.428382	-4.446487	-0.324563
C	1.850285	-3.630755	3.341396
C	0.874281	-4.776051	1.392162
C	0.585570	-1.891315	4.442503
H	-1.361382	-1.463979	3.701487
C	-0.060293	-5.934087	-0.569934
C	-2.292599	-5.379270	-1.310076
H	-3.353035	-3.886238	-0.231386
C	1.764746	-2.684085	4.319354
H	2.736096	-4.254886	3.245834
H	1.779915	-5.373444	1.300132
H	0.529768	-1.135923	5.219488
H	0.860229	-6.505668	-0.654865
C	-1.089517	-6.135216	-1.438830
H	-3.108588	-5.555445	-2.003997
H	2.585050	-2.536042	5.014578
H	-1.001174	-6.870070	-2.232805
H	0.216256	-0.643924	1.315520
O	-2.313070	1.257290	0.411155
O	-2.204561	-1.222986	-0.140312
C	2.260573	3.767447	1.817093
C	3.409076	3.012321	1.553913
C	3.402747	2.043432	0.564066
C	2.216312	1.829745	-0.155930
C	1.067089	2.598470	0.086679
C	1.095463	3.567162	1.077811
H	2.281921	4.521700	2.596868
H	4.322375	3.174677	2.113486
H	4.315629	1.489294	0.365664
H	0.178073	2.443298	-0.515698
H	0.207389	4.163398	1.261020
N	2.124390	0.885260	-1.185394
H	1.156667	0.736894	-1.558432
C	3.072540	0.131065	-1.688751
H	4.107553	0.301302	-1.403594
C	2.735010	-0.505707	-3.007423
O	1.675465	-0.332806	-3.550197
O	3.745049	-1.235094	-3.465654
C	3.514213	-1.829812	-4.756809
H	4.363151	-2.487388	-4.930025
H	2.577613	-2.389157	-4.742588
H	3.469747	-1.045086	-5.514019
C	1.757964	-2.243762	-0.700198

O	0.933594	-1.799165	0.095100
C	1.350791	-3.064357	-1.866734
C	2.214539	-4.003458	-2.445655
C	0.061894	-2.882347	-2.381463
C	1.790939	-4.754131	-3.537293
H	3.202909	-4.177943	-2.029216
C	-0.340702	-3.610295	-3.494071
H	-0.594422	-2.152177	-1.918136
C	0.519326	-4.544847	-4.070502
H	2.451062	-5.496933	-3.973530
H	-1.333346	-3.457867	-3.904498
H	0.195007	-5.119502	-4.932642
C	3.174260	-1.891757	-0.523998
C	3.643327	-1.440976	0.811788
H	3.891193	-2.320589	-1.215609
H	3.024615	-0.598290	1.160731
H	3.393256	-2.238014	1.535959
C	5.104197	-1.097899	0.911956
H	5.451132	-0.852599	1.915023
C	5.968542	-1.053370	-0.101826
H	5.643227	-1.273176	-1.117313
C	7.409332	-0.638440	0.018385
H	7.846812	-0.939445	0.975589
H	8.011397	-1.088674	-0.775985
C	7.557609	0.877968	-0.129936
F	8.776449	1.179042	-0.619131
F	6.628883	1.354519	-1.003987
C	7.350252	1.591502	1.211626
O	6.260808	1.925113	1.610328
O	8.484734	1.695592	1.875867
C	8.397504	2.294643	3.189228
H	7.972322	3.295482	3.077512
H	7.707670	1.696807	3.791792
C	9.795531	2.321302	3.762061
H	10.460246	2.911165	3.127211
H	9.775624	2.771048	4.757712
H	10.198973	1.309637	3.846177

^{OSS}TS-4

M06-2X SCF energy: -4086.85964406 a.u.

M06-2X enthalpy: -4085.660515 a.u.

M06-2X free energy: -4085.844909 a.u.

M06-2X SCF energy in solution: -4088.01893646 a.u.
M06-2X enthalpy in solution: -4086.819807 a.u.
M06-2X free energy in solution: -4087.004201 a.u.
Imaginary frequency: -25.1011 cm-1

Cartesian coordinates

ATOM	X	Y	Z
C	-6.190717	-4.217419	1.938881
C	-5.360033	-3.549108	1.075360
C	-3.964508	-3.459155	1.328582
C	-3.454394	-4.048688	2.520809
C	-4.338642	-4.743492	3.387245
C	-5.676402	-4.832509	3.104186
H	-7.254404	-4.269296	1.729295
H	-5.767476	-3.071299	0.191068
C	-3.064639	-2.763028	0.454746
C	-2.074670	-3.927614	2.835598
H	-3.930412	-5.195901	4.286931
H	-6.344539	-5.362028	3.775629
C	-1.219119	-3.212245	2.037569
C	-1.755213	-2.617080	0.863401
H	-1.693627	-4.406667	3.734121
C	-3.496613	-2.149592	-0.834163
C	-4.119181	-2.895852	-1.888755
C	-3.236509	-0.814602	-1.059880
C	-4.318568	-4.301399	-1.819142
C	-4.520234	-2.213531	-3.073877
C	-3.618340	-0.114019	-2.233626
C	-4.916497	-4.979803	-2.850796
H	-3.982669	-4.838836	-0.938990
C	-5.146544	-2.943678	-4.118189
C	-4.269290	-0.821999	-3.211335
C	-5.347189	-4.294851	-4.011156
H	-5.054816	-6.053954	-2.779039
H	-5.454672	-2.406039	-5.010805
H	-4.582894	-0.315815	-4.120906
H	-5.823268	-4.843649	-4.817254
P	-1.014493	-0.279139	0.092578
O	-0.790719	0.099369	1.593827
O	-0.127146	0.258169	-0.958419
C	-3.307329	1.342924	-2.348013
C	-4.147395	2.281290	-1.718288
C	-2.171120	1.766908	-3.060881
C	-3.850734	3.681339	-1.825215

C	-5.304240	1.895064	-0.968908
C	-1.875637	3.170537	-3.148867
C	-1.271150	0.849777	-3.697282
C	-4.726066	4.629804	-1.209151
C	-2.719019	4.091714	-2.530698
C	-6.114493	2.829589	-0.391378
H	-5.533936	0.839056	-0.868381
C	-0.709512	3.595577	-3.862474
C	-0.172627	1.295925	-4.369887
H	-1.465394	-0.214215	-3.615798
C	-5.828438	4.220893	-0.518651
H	-4.485901	5.685639	-1.303343
H	-2.489361	5.153316	-2.597888
H	-6.987947	2.514481	0.170877
H	-0.494820	4.660334	-3.904680
C	0.117733	2.691035	-4.455194
H	0.502859	0.583459	-4.833401
H	-6.488144	4.948394	-0.056024
H	1.008853	3.020949	-4.979730
C	0.244837	-3.103623	2.319175
C	0.734023	-2.190211	3.271697
C	1.133356	-3.927978	1.599533
C	2.149651	-2.102641	3.504281
C	-0.123735	-1.307046	4.006307
C	2.544080	-3.844338	1.853468
C	0.683888	-4.863181	0.611245
C	2.644600	-1.141892	4.443429
C	3.017324	-2.932911	2.797027
C	0.387726	-0.393787	4.880128
H	-1.191695	-1.350983	3.824214
C	3.437625	-4.692240	1.123257
C	1.567178	-5.648156	-0.069901
H	-0.379043	-4.941106	0.407544
C	1.794722	-0.307968	5.105917
H	3.718256	-1.091497	4.606882
H	4.088895	-2.861042	2.976786
H	-0.277997	0.284588	5.403574
H	4.503544	-4.619131	1.328087
C	2.967193	-5.563989	0.187332
H	1.204192	-6.346289	-0.817514
H	2.180271	0.424735	5.808016
H	3.653735	-6.198425	-0.363987
H	-0.599584	1.052069	1.809520
O	-2.604661	-0.079956	-0.079291

O	-0.877932	-1.889666	0.084445
C	4.724640	2.731174	-2.125135
C	4.251387	3.877024	-1.493551
C	2.909559	3.993393	-1.137750
C	2.023075	2.948442	-1.431864
C	2.495477	1.797540	-2.082832
C	3.836525	1.694376	-2.418529
H	5.775443	2.634198	-2.375850
H	4.932396	4.690584	-1.260983
H	2.560468	4.885217	-0.627901
H	1.796315	0.990089	-2.284239
H	4.199056	0.787025	-2.888647
N	0.688250	2.975966	-1.048545
H	0.177217	2.085090	-1.120363
C	0.064287	3.962064	-0.371284
H	0.468154	4.963442	-0.333164
C	-1.162888	3.610804	0.280882
O	-1.609126	2.468206	0.322581
O	-1.758790	4.644174	0.904939
C	-2.850477	4.253482	1.735317
H	-3.226449	5.175335	2.179657
H	-3.628686	3.762382	1.147261
H	-2.499315	3.562762	2.507272
C	1.104945	3.090749	2.420655
O	0.051066	2.464957	2.649001
C	1.170786	4.550176	2.750269
C	2.221047	5.377751	2.337243
C	0.114976	5.103385	3.479946
C	2.212339	6.732998	2.651156
H	3.041629	4.977544	1.750027
C	0.109787	6.455837	3.798544
H	-0.694067	4.450868	3.789707
C	1.158349	7.274471	3.383979
H	3.029072	7.367338	2.321481
H	-0.714243	6.873912	4.368150
H	1.153816	8.332089	3.628558
C	2.251702	2.416016	1.872402
C	2.150648	0.982345	1.503140
H	3.189852	2.939768	1.732669
H	1.557777	0.471110	2.278166
H	1.549290	0.897722	0.582392
C	3.447147	0.256251	1.309512
H	4.197542	0.361381	2.095379
C	3.666550	-0.572514	0.291926

H	2.902866	-0.688682	-0.476665
C	4.874935	-1.452313	0.181190
H	5.577413	-1.273234	1.002029
H	4.576215	-2.505577	0.229675
C	5.621579	-1.275429	-1.118665
F	4.779128	-1.449559	-2.178089
F	6.135662	-0.024561	-1.227946
C	6.768824	-2.285162	-1.293698
O	6.956368	-3.216344	-0.552377
O	7.492973	-1.997013	-2.367709
C	8.575077	-2.903677	-2.643841
H	9.246279	-2.916043	-1.780223
H	8.163181	-3.910067	-2.762035
C	9.260744	-2.411514	-3.898649
H	9.647993	-1.400288	-3.753124
H	10.094734	-3.071993	-4.149632
H	8.561372	-2.398092	-4.737908

^{OSS}TS4'

M06-2X SCF energy: -4086.85337591 a.u.

M06-2X enthalpy: -4085.653771 a.u.

M06-2X free energy: -4085.836505 a.u.

M06-2X SCF energy in solution: -4088.01354769 a.u.

M06-2X enthalpy in solution: -4086.813943 a.u.

M06-2X free energy in solution: -4086.996677 a.u.

Imaginary frequency: -21.3438 cm-1

Cartesian coordinates

ATOM	X	Y	Z
C	6.959825	2.398034	-3.116094
C	6.017351	2.306448	-2.123549
C	5.393414	1.065003	-1.824633
C	5.742182	-0.073665	-2.605205
C	6.730042	0.051872	-3.617243
C	7.331161	1.257552	-3.866608
H	7.421183	3.356298	-3.332456
H	5.732011	3.189062	-1.561512
C	4.399853	0.928777	-0.800561
C	5.090051	-1.312991	-2.373993
H	6.991865	-0.829483	-4.196311
H	8.082073	1.344537	-4.645183
C	4.097268	-1.438439	-1.435067

C	3.763040	-0.287832	-0.670811
H	5.377193	-2.179217	-2.965004
C	3.986789	2.053652	0.084609
C	4.902348	2.751679	0.938452
C	2.652932	2.395165	0.149062
C	6.276503	2.402253	1.039480
C	4.412067	3.813418	1.751652
C	2.135286	3.429116	0.972107
C	7.119315	3.095868	1.870333
H	6.654871	1.570361	0.455407
C	5.313166	4.517825	2.593195
C	3.028550	4.132088	1.740268
C	6.638221	4.174184	2.649713
H	8.164524	2.810661	1.935777
H	4.923955	5.330435	3.200471
H	2.665498	4.933436	2.378722
H	7.317594	4.716406	3.299370
P	1.319133	0.238200	-0.148121
O	0.903922	-0.386585	-1.525276
O	0.371157	0.138464	0.968472
C	0.666495	3.695362	0.990923
C	0.093124	4.486864	-0.022178
C	-0.135777	3.109823	1.987450
C	-1.321561	4.722058	-0.016219
C	0.868814	5.065753	-1.077972
C	-1.554570	3.336612	1.969086
C	0.394283	2.245329	3.001141
C	-1.898988	5.515685	-1.056053
C	-2.109241	4.147155	0.981601
C	0.280398	5.814968	-2.054616
H	1.941267	4.897915	-1.092211
C	-2.378568	2.687808	2.944735
C	-0.425833	1.622902	3.891532
H	1.460611	2.045853	3.014341
C	-1.127140	6.045584	-2.046962
H	-2.973823	5.677633	-1.036741
H	-3.185757	4.303248	0.965568
H	0.885930	6.241208	-2.848461
H	-3.450686	2.862177	2.904674
C	-1.835743	1.844484	3.864588
H	-0.017116	0.918851	4.607389
H	-1.575914	6.644765	-2.833128
H	-2.468949	1.322395	4.575350
C	3.422116	-2.743938	-1.168303

C	2.431130	-3.237258	-2.039497
C	3.797010	-3.479863	-0.024906
C	1.799843	-4.496820	-1.749540
C	1.994038	-2.519079	-3.201400
C	3.184389	-4.751550	0.233256
C	4.771069	-3.001498	0.910965
C	0.761362	-4.973940	-2.612149
C	2.200623	-5.227403	-0.632752
C	1.000947	-3.008330	-3.997612
H	2.448356	-1.559377	-3.421027
C	3.562652	-5.486152	1.400141
C	5.100269	-3.729015	2.017771
H	5.248321	-2.043935	0.730360
C	0.374148	-4.256109	-3.703221
H	0.294848	-5.926653	-2.374309
H	1.717516	-6.178025	-0.416780
H	0.677406	-2.441456	-4.864681
H	3.079539	-6.442204	1.582793
C	4.488575	-4.992387	2.269569
H	5.836611	-3.344818	2.716560
H	-0.409483	-4.628957	-4.356165
H	4.763969	-5.553859	3.156623
H	0.272487	-1.161328	-1.409456
O	1.749264	1.706660	-0.646021
O	2.745086	-0.420885	0.260656
C	-4.078523	2.700128	-1.498095
C	-4.804937	2.267466	-0.389839
C	-4.296207	1.289729	0.462133
C	-3.028809	0.741468	0.214167
C	-2.297275	1.176699	-0.901799
C	-2.821945	2.146013	-1.746959
H	-4.477980	3.467171	-2.154206
H	-5.773559	2.703571	-0.161725
H	-4.865366	0.976972	1.331572
H	-1.325891	0.737862	-1.103945
H	-2.235437	2.483516	-2.595901
N	-2.463980	-0.202492	1.074040
H	-1.448440	-0.152074	1.227156
C	-3.173738	-0.991953	1.913560
H	-4.218520	-1.182535	1.709262
C	-2.508200	-1.521186	3.068087
O	-1.363918	-1.258460	3.405400
O	-3.317235	-2.354794	3.784388
C	-2.809878	-2.691558	5.065048

H	-3.489507	-3.440754	5.472423
H	-1.794133	-3.085478	4.994228
H	-2.794368	-1.810163	5.715245
C	-1.330812	-3.130310	-0.488512
O	-0.849103	-2.269430	-1.250900
C	-0.724902	-3.445133	0.829667
C	-1.394285	-4.202464	1.800819
C	0.565127	-2.982721	1.094929
C	-0.766694	-4.500112	3.002881
H	-2.415727	-4.533443	1.644774
C	1.182367	-3.256399	2.307955
H	1.104317	-2.444709	0.323941
C	0.518856	-4.023342	3.260390
H	-1.288525	-5.094755	3.746393
H	2.187537	-2.886858	2.490439
H	1.002711	-4.251766	4.205373
C	-2.490228	-3.845956	-0.954062
C	-3.090782	-3.478634	-2.268661
H	-2.958036	-4.604703	-0.340410
H	-2.283787	-3.355108	-3.001254
H	-3.776265	-4.257141	-2.614079
C	-3.824028	-2.165895	-2.109772
H	-3.200375	-1.303898	-1.874121
C	-5.143402	-2.031580	-2.204752
H	-5.771712	-2.895162	-2.419699
C	-5.839462	-0.717838	-2.003215
H	-5.111605	0.082311	-1.836765
H	-6.452353	-0.446653	-2.869250
C	-6.777084	-0.791898	-0.812308
F	-7.755684	-1.710778	-1.069520
F	-6.125756	-1.210487	0.295066
C	-7.490549	0.521567	-0.459497
O	-7.552437	0.986549	0.646820
O	-8.044953	1.048660	-1.550403
C	-8.788846	2.261231	-1.341332
H	-9.569221	2.066563	-0.600378
H	-8.114379	3.016228	-0.925926
C	-9.354417	2.675921	-2.681180
H	-10.014029	1.899693	-3.076370
H	-9.928950	3.599344	-2.572494
H	-8.550183	2.848685	-3.400566

(*R,S*)-**TS-5**

M06-2X SCF energy: -4086.88503761 a.u.

M06-2X enthalpy: -4085.684378 a.u.

M06-2X free energy: -4085.870774 a.u.

M06-2X SCF energy in solution: -4088.05383646 a.u.

M06-2X enthalpy in solution: -4086.853177 a.u.

M06-2X free energy in solution: -4087.039573 a.u.

Imaginary frequency: -97.7678 cm-1

Cartesian coordinates

ATOM	X	Y	Z
C	-7.611024	2.105106	-2.278088
C	-6.727352	1.463443	-1.447841
C	-5.546089	2.113614	-0.995959
C	-5.282905	3.433401	-1.461111
C	-6.222525	4.073582	-2.311769
C	-7.365106	3.430243	-2.709008
H	-8.505424	1.589121	-2.613028
H	-6.922079	0.443930	-1.133115
C	-4.596055	1.482008	-0.129918
C	-4.073828	4.078180	-1.093602
H	-6.008975	5.084583	-2.648376
H	-8.075572	3.927116	-3.361859
C	-3.135058	3.455738	-0.309247
C	-3.423155	2.147481	0.164741
H	-3.878121	5.082163	-1.462502
C	-4.773256	0.099767	0.390239
C	-5.894079	-0.297386	1.187926
C	-3.783383	-0.828322	0.130765
C	-6.894398	0.619497	1.612250
C	-5.996118	-1.652509	1.611449
C	-3.880801	-2.188758	0.527977
C	-7.949884	0.202200	2.382566
H	-6.807884	1.662180	1.325774
C	-7.106648	-2.055435	2.399148
C	-4.983952	-2.576928	1.247131
C	-8.066524	-1.152706	2.774578
H	-8.700461	0.919142	2.700168
H	-7.173354	-3.096235	2.704677
H	-5.083559	-3.615927	1.552281
H	-8.909817	-1.469281	3.379946
P	-1.540285	0.397203	0.278966
O	-0.640309	1.033183	-0.739170
O	-1.001601	-0.416596	1.416941

C	-2.854272	-3.189289	0.112138
C	-2.868039	-3.670035	-1.212905
C	-1.944513	-3.712977	1.047759
C	-1.984751	-4.735699	-1.591112
C	-3.756424	-3.147792	-2.207764
C	-1.050213	-4.767279	0.653211
C	-1.866675	-3.229744	2.396607
C	-2.048235	-5.253520	-2.923762
C	-1.093284	-5.256341	-0.652001
C	-3.769268	-3.650930	-3.475522
H	-4.423522	-2.335416	-1.939961
C	-0.144974	-5.312034	1.622103
C	-1.000168	-3.784341	3.292051
H	-2.493196	-2.392805	2.683648
C	-2.907931	-4.727461	-3.840690
H	-1.390059	-6.078586	-3.185965
H	-0.427843	-6.068438	-0.940234
H	-4.445651	-3.235139	-4.215431
H	0.512358	-6.121223	1.312307
C	-0.133804	-4.850781	2.904635
H	-0.943289	-3.396897	4.304758
H	-2.946230	-5.126486	-4.849521
H	0.543195	-5.278814	3.637810
C	-1.848274	4.122661	0.041950
C	-0.824553	4.223098	-0.917958
C	-1.678274	4.666391	1.329245
C	0.390264	4.912813	-0.583798
C	-0.928724	3.633889	-2.220892
C	-0.459651	5.351558	1.653002
C	-2.687022	4.569607	2.341451
C	1.428464	5.010394	-1.564747
C	0.540699	5.464587	0.687380
C	0.087609	3.739090	-3.122504
H	-1.821376	3.069646	-2.466120
C	-0.300898	5.907642	2.961590
C	-2.496401	5.109930	3.578987
H	-3.613051	4.053720	2.109180
C	1.284331	4.444592	-2.794792
H	2.338110	5.541944	-1.297632
H	1.461992	5.988139	0.934753
H	-0.002196	3.269074	-4.096949
H	0.627348	6.424914	3.190122
C	-1.284668	5.791711	3.897052
H	-3.272137	5.021890	4.333172

H	2.080538	4.517844	-3.529092
H	-1.153007	6.216591	4.887299
H	0.859699	0.728024	-0.616611
O	-2.665488	-0.445553	-0.570533
O	-2.495228	1.534733	0.971806
C	2.386988	-1.884041	5.407271
C	2.853165	-2.898256	4.571483
C	2.444973	-2.959512	3.244907
C	1.569100	-1.989788	2.752420
C	1.086252	-0.976448	3.582528
C	1.503408	-0.930013	4.908723
H	2.707693	-1.843797	6.442984
H	3.530880	-3.653561	4.956135
H	2.782901	-3.775505	2.614172
H	0.385855	-0.252737	3.177005
H	1.131172	-0.141229	5.554076
N	1.100099	-2.005731	1.414662
H	0.239455	-1.395830	1.234834
C	1.642323	-2.643269	0.420057
H	2.453773	-3.344991	0.572936
C	0.865363	-2.685530	-0.858773
O	-0.135391	-2.035317	-1.023954
O	1.451149	-3.496230	-1.735100
C	0.864828	-3.573881	-3.043330
H	-0.129308	-3.126895	-3.041345
H	1.521163	-3.048692	-3.740860
H	0.806978	-4.633245	-3.295718
C	2.441723	-0.429254	-1.069682
O	1.763881	0.383158	-0.284760
C	2.179276	-0.494303	-2.530620
C	3.224419	-0.855530	-3.395866
C	0.925868	-0.177800	-3.073680
C	3.015596	-0.932340	-4.767378
H	4.215831	-1.041464	-2.996458
C	0.724204	-0.259325	-4.449169
H	0.101320	0.110694	-2.430379
C	1.759193	-0.640670	-5.298342
H	3.837524	-1.203845	-5.422155
H	-0.254876	-0.023609	-4.854289
H	1.594667	-0.697576	-6.370066
C	3.390303	-1.231199	-0.471462
C	3.985096	-0.876376	0.868494
H	3.954149	-1.912237	-1.100319
H	3.225390	-0.386428	1.485792

H	4.331683	-1.769815	1.400651
C	5.143717	0.061460	0.653123
H	4.889530	1.025105	0.210077
C	6.413469	-0.228243	0.924986
H	6.670218	-1.195291	1.355115
C	7.557698	0.707489	0.662610
H	7.198582	1.649160	0.233915
H	8.108539	0.937530	1.581673
C	8.552710	0.094464	-0.308882
F	9.038570	-1.066487	0.213332
F	7.951052	-0.205357	-1.477706
C	9.739880	1.019013	-0.612252
O	9.906791	1.580570	-1.660220
O	10.517041	1.136084	0.464585
C	11.652796	2.005162	0.308788
H	12.267015	1.627799	-0.513654
H	11.294144	2.999917	0.028760
C	12.396530	2.010585	1.625579
H	12.728298	1.002008	1.883350
H	13.273711	2.658739	1.554173
H	11.754967	2.381338	2.428764

(*R,R*)-**TS-5**

M06-2X SCF energy: -4086.88456310 a.u.
M06-2X enthalpy: -4085.683388 a.u.
M06-2X free energy: -4085.867708 a.u.
M06-2X SCF energy in solution: -4088.05222721 a.u.
M06-2X enthalpy in solution: -4086.851052 a.u.
M06-2X free energy in solution: -4087.035372 a.u.
Imaginary frequency: -52.2464 cm-1

Cartesian coordinates

ATOM	X	Y	Z
C	-7.461255	2.108878	-2.457947
C	-6.543819	1.439399	-1.688713
C	-5.348510	2.077597	-1.254874
C	-5.115194	3.417427	-1.672184
C	-6.089190	4.087920	-2.458134
C	-7.241302	3.453333	-2.840667
H	-8.363330	1.599556	-2.782320
H	-6.721319	0.405524	-1.414171
C	-4.362587	1.414806	-0.452459

C	-3.906429	4.058324	-1.303274
H	-5.894110	5.114366	-2.757054
H	-7.978898	3.971466	-3.445048
C	-2.929412	3.405821	-0.593600
C	-3.163827	2.058175	-0.199424
H	-3.746678	5.092761	-1.597810
C	-4.574528	0.031189	0.051825
C	-5.692182	-0.338861	0.869130
C	-3.646356	-0.936374	-0.268275
C	-6.626413	0.610163	1.365553
C	-5.860735	-1.705790	1.230198
C	-3.812758	-2.310090	0.058059
C	-7.681318	0.216120	2.149306
H	-6.489399	1.658828	1.124259
C	-6.969611	-2.083052	2.032456
C	-4.918688	-2.670388	0.786371
C	-7.864382	-1.147061	2.480984
H	-8.380069	0.957857	2.523209
H	-7.088199	-3.132296	2.289667
H	-5.076844	-3.716641	1.037783
H	-8.706715	-1.445033	3.097039
P	-1.346000	0.173176	-0.135685
O	-0.403853	0.692818	-1.178790
O	-0.910351	-0.643416	1.042885
C	-2.840429	-3.321398	-0.446320
C	-2.805030	-3.610752	-1.826483
C	-2.007940	-4.020981	0.446512
C	-1.925921	-4.633458	-2.314790
C	-3.643147	-2.939233	-2.774259
C	-1.112487	-5.026364	-0.058306
C	-2.017205	-3.778712	1.860485
C	-1.931873	-4.952667	-3.710215
C	-1.088310	-5.305644	-1.423599
C	-3.609952	-3.260650	-4.099207
H	-4.314454	-2.162199	-2.425285
C	-0.277902	-5.743178	0.859885
C	-1.240976	-4.516075	2.705713
H	-2.650702	-2.987575	2.246979
C	-2.743433	-4.286043	-4.578566
H	-1.270388	-5.742537	-4.058737
H	-0.418539	-6.077551	-1.798762
H	-4.251271	-2.734531	-4.798866
H	0.394255	-6.498140	0.458906
C	-0.353627	-5.513176	2.201008

H	-1.262320	-4.319219	3.773085
H	-2.740030	-4.535493	-5.635042
H	0.268244	-6.072481	2.893308
C	-1.694553	4.148098	-0.195742
C	-0.565750	4.166324	-1.035791
C	-1.714381	4.910845	0.988332
C	0.563077	4.986594	-0.688898
C	-0.495039	3.389637	-2.237160
C	-0.588893	5.741413	1.317112
C	-2.828694	4.897433	1.889830
C	1.715658	4.977850	-1.536869
C	0.517635	5.766227	0.467255
C	0.624491	3.404957	-3.015745
H	-1.332891	2.753609	-2.497161
C	-0.625546	6.519130	2.518018
C	-2.822512	5.649848	3.027092
H	-3.680774	4.267930	1.655615
C	1.748516	4.208943	-2.661588
H	2.564571	5.595736	-1.256740
H	1.370077	6.393218	0.720459
H	0.668354	2.789113	-3.908057
H	0.232868	7.143709	2.751059
C	-1.705365	6.477270	3.347584
H	-3.673753	5.620494	3.699902
H	2.630784	4.202489	-3.294082
H	-1.721888	7.070763	4.256314
H	0.999284	0.304996	-1.429397
O	-2.531855	-0.580648	-0.982318
O	-2.188846	1.424607	0.525025
C	1.700601	-3.015710	5.374868
C	2.457662	-3.732339	4.452449
C	2.248019	-3.568116	3.085291
C	1.271945	-2.673938	2.646312
C	0.507387	-1.946834	3.564871
C	0.726568	-2.123355	4.923829
H	1.868341	-3.150794	6.438226
H	3.215630	-4.430064	4.793466
H	2.830500	-4.154198	2.382068
H	-0.236714	-1.249435	3.188249
H	0.134266	-1.557371	5.635761
N	0.986566	-2.466734	1.279148
H	0.171185	-1.810004	1.070065
C	1.696059	-2.888204	0.263539
H	2.497157	-3.604424	0.406480

C	1.051458	-2.803277	-1.085834
O	0.066271	-2.152424	-1.311660
O	1.715873	-3.532760	-1.982338
C	1.289110	-3.320505	-3.334822
H	1.957821	-3.921245	-3.949334
H	0.251528	-3.633402	-3.457516
H	1.376254	-2.258762	-3.579167
C	2.559439	-0.318281	-0.425856
O	1.968606	-0.046865	-1.569077
C	2.227713	0.500254	0.758447
C	2.558608	0.082018	2.054041
C	1.591430	1.742143	0.591283
C	2.205088	0.845397	3.159300
H	3.072327	-0.858342	2.216761
C	1.224285	2.494067	1.698733
H	1.372942	2.114596	-0.403758
C	1.518429	2.043076	2.984101
H	2.450509	0.488968	4.154751
H	0.703570	3.434031	1.557461
H	1.219543	2.632916	3.845162
C	3.450476	-1.378274	-0.364829
C	4.096630	-1.953955	-1.593328
H	4.021502	-1.489299	0.552037
H	4.156983	-3.048506	-1.529265
H	3.478828	-1.738012	-2.471874
C	5.489586	-1.419417	-1.830748
H	6.059292	-1.938295	-2.602829
C	6.055760	-0.393087	-1.200714
H	5.512505	0.145320	-0.426798
C	7.450562	0.082840	-1.488008
H	7.882645	-0.456118	-2.338100
H	7.466929	1.154403	-1.716414
C	8.360996	-0.134263	-0.289860
F	7.855997	0.518427	0.791867
F	8.427995	-1.445810	0.018033
C	9.791372	0.365360	-0.535973
O	10.728255	-0.355400	-0.748258
O	9.821565	1.697687	-0.531331
C	11.110849	2.282437	-0.785799
H	11.810781	1.920609	-0.027359
H	11.465114	1.933926	-1.760388
C	10.938539	3.784163	-0.738571
H	10.570674	4.097547	0.241297
H	11.897681	4.274280	-0.924391

H 10.226210 4.114266 -1.498663

(*S,S*)-**TS-5**
M06-2X SCF energy: -4086.89036606 a.u.
M06-2X enthalpy: -4085.689741 a.u.
M06-2X free energy: -4085.874717 a.u.
M06-2X SCF energy in solution: -4088.05244619 a.u.
M06-2X enthalpy in solution: -4086.851821 a.u.
M06-2X free energy in solution: -4087.036797 a.u.
Imaginary frequency: -100.2281 cm-1

Cartesian coordinates

ATOM	X	Y	Z
C	-4.420599	-6.087156	1.901201
C	-3.940328	-5.064760	1.122905
C	-2.651591	-4.512181	1.358183
C	-1.881925	-5.029771	2.437640
C	-2.402457	-6.095378	3.218050
C	-3.642149	-6.617458	2.957280
H	-5.410118	-6.489985	1.709261
H	-4.548444	-4.658467	0.321571
C	-2.120230	-3.439701	0.570556
C	-0.603197	-4.478824	2.709965
H	-1.796871	-6.482850	4.032993
H	-4.033554	-7.429004	3.562274
C	-0.116133	-3.409714	2.001452
C	-0.923361	-2.867325	0.962135
H	0.005239	-4.918537	3.496470
C	-2.812502	-2.921314	-0.643396
C	-3.119899	-3.751471	-1.771391
C	-3.091015	-1.574025	-0.726688
C	-2.774831	-5.129158	-1.832972
C	-3.759848	-3.165731	-2.900966
C	-3.711420	-0.965668	-1.851766
C	-3.085740	-5.884725	-2.935188
H	-2.251126	-5.580605	-0.997692
C	-4.077079	-3.977388	-4.021794
C	-4.049269	-1.776107	-2.906319
C	-3.754725	-5.308924	-4.040574
H	-2.809593	-6.934149	-2.962339
H	-4.573768	-3.514402	-4.870270
H	-4.533360	-1.342590	-3.778136

H	-3.998180	-5.920061	-4.903737
P	-1.167154	-0.334087	0.365123
O	-0.980817	0.314781	1.704691
O	-0.785690	0.379816	-0.891263
C	-4.010133	0.498788	-1.875441
C	-5.110073	0.989773	-1.143024
C	-3.227380	1.375172	-2.652783
C	-5.465350	2.376941	-1.238835
C	-5.902142	0.150201	-0.294047
C	-3.579920	2.767525	-2.720309
C	-2.052413	0.944321	-3.354352
C	-6.580915	2.864770	-0.488167
C	-4.699502	3.230123	-2.032819
C	-6.953838	0.654553	0.413260
H	-5.648151	-0.901700	-0.214021
C	-2.749613	3.663570	-3.468157
C	-1.277329	1.831917	-4.038818
H	-1.754865	-0.094550	-3.280500
C	-7.302357	2.034470	0.316722
H	-6.830756	3.919812	-0.570412
H	-4.958499	4.285946	-2.084395
H	-7.534610	0.002205	1.057648
H	-3.031230	4.713183	-3.501050
C	-1.629755	3.214424	-4.099818
H	-0.368494	1.490435	-4.522566
H	-8.144604	2.415473	0.885937
H	-0.999563	3.903061	-4.655058
C	1.273662	-2.906617	2.227741
C	1.526875	-1.825902	3.092317
C	2.336402	-3.552641	1.565664
C	2.880257	-1.394646	3.311127
C	0.475514	-1.115528	3.755156
C	3.687541	-3.126377	1.806423
C	2.126292	-4.629556	0.642175
C	3.121646	-0.263039	4.152566
C	3.926632	-2.065049	2.677920
C	0.745012	-0.028947	4.535665
H	-0.549779	-1.425014	3.588913
C	4.760745	-3.791397	1.130559
C	3.175073	-5.232880	0.013182
H	1.111114	-4.954899	0.440919
C	2.087749	0.407998	4.736543
H	4.150620	0.053588	4.305123
H	4.951400	-1.742354	2.855799

H	-0.070381	0.519582	4.995681
H	5.776997	-3.449208	1.312336
C	4.515472	-4.810492	0.259740
H	2.992090	-6.041199	-0.687764
H	2.279994	1.275522	5.360440
H	5.336609	-5.301468	-0.252876
H	-0.330201	1.642149	2.069487
O	-2.744171	-0.769951	0.335111
O	-0.420149	-1.789059	0.279027
C	-3.856908	5.121444	0.875647
C	-2.913200	5.767895	0.080165
C	-1.822698	5.064281	-0.421171
C	-1.670948	3.714088	-0.106204
C	-2.626532	3.050003	0.663321
C	-3.713146	3.762936	1.156126
H	-4.709675	5.669616	1.263826
H	-3.027539	6.820052	-0.160900
H	-1.103564	5.551709	-1.072841
H	-2.498472	2.003001	0.917533
H	-4.457857	3.245002	1.752060
N	-0.539459	3.002103	-0.599780
H	-0.665762	1.987227	-0.892114
C	0.676374	3.488959	-0.572291
H	0.828256	4.522065	-0.273360
C	1.695989	2.856405	-1.479669
O	1.475175	1.875803	-2.139703
O	2.834086	3.551258	-1.475349
C	3.853256	3.031004	-2.340239
H	4.763411	3.567864	-2.081351
H	3.977866	1.962267	-2.176640
H	3.576704	3.213591	-3.381329
C	0.915479	3.224152	2.037494
O	-0.175531	2.598973	2.402753
C	0.930899	4.654257	2.430450
C	2.126683	5.371628	2.548549
C	-0.287864	5.307756	2.654221
C	2.103686	6.723943	2.872091
H	3.077503	4.865787	2.413700
C	-0.305760	6.661609	2.969050
H	-1.213746	4.747524	2.562905
C	0.887785	7.372730	3.078038
H	3.036494	7.269736	2.971068
H	-1.256423	7.161481	3.126134
H	0.871814	8.428934	3.328267

C	1.887388	2.656207	1.241875
C	1.941156	1.165892	0.999093
H	2.765641	3.251348	1.014984
H	1.552725	0.649658	1.889097
H	1.282920	0.867304	0.171914
C	3.320685	0.647315	0.727011
H	4.074186	0.846430	1.491947
C	3.636381	-0.114056	-0.319521
H	2.883800	-0.322622	-1.078965
C	4.978518	-0.772193	-0.470529
H	5.532278	-0.755853	0.473721
H	4.864845	-1.824309	-0.752670
C	5.847176	-0.106820	-1.509083
F	5.205517	-0.035550	-2.706618
F	6.133839	1.176409	-1.147328
C	7.189118	-0.825073	-1.725682
O	7.563875	-1.751374	-1.053080
O	7.864953	-0.275416	-2.727165
C	9.148907	-0.864324	-3.000316
H	9.761343	-0.796289	-2.096555
H	9.002944	-1.924406	-3.226627
C	9.750419	-0.105708	-4.161973
H	9.871343	0.950451	-3.909606
H	10.731473	-0.520148	-4.407933
H	9.108883	-0.181274	-5.043094

(*S,R*)-**TS-5**

M06-2X SCF energy: -4064.57225791 a.u.

M06-2X enthalpy: -4085.685879 a.u.

M06-2X free energy: -4085.868317 a.u.

M06-2X SCF energy in solution: -4088.05062625 a.u.

M06-2X enthalpy in solution: -4109.164247 a.u.

M06-2X free energy in solution: -4109.346685 a.u.

Imaginary frequency: -30.1907 cm-1

Cartesian coordinates

ATOM	X	Y	Z
C	-4.494509	5.576959	-2.536388
C	-4.287826	4.436265	-1.803430
C	-3.034571	4.187610	-1.178764
C	-1.986572	5.131839	-1.369806
C	-2.235597	6.308191	-2.125426

C	-3.462294	6.532655	-2.692297
H	-5.457752	5.746091	-3.007291
H	-5.083248	3.706271	-1.700464
C	-2.776392	3.011478	-0.403896
C	-0.700987	4.866858	-0.833553
H	-1.426093	7.022041	-2.251856
H	-3.642365	7.432791	-3.271163
C	-0.430850	3.712832	-0.140556
C	-1.497256	2.796894	0.071812
H	0.098251	5.585118	-0.999635
C	-3.805269	1.962133	-0.170578
C	-5.049793	2.214513	0.489967
C	-3.512593	0.669751	-0.555841
C	-5.391138	3.487369	1.022923
C	-5.968342	1.140331	0.660692
C	-4.408927	-0.417742	-0.382837
C	-6.593182	3.685905	1.653026
H	-4.682574	4.303678	0.932363
C	-7.209052	1.381338	1.308215
C	-5.622922	-0.157630	0.204877
C	-7.520452	2.625526	1.789842
H	-6.833738	4.664370	2.056628
H	-7.902747	0.552566	1.421231
H	-6.331756	-0.970412	0.343724
H	-8.469667	2.798949	2.286489
P	-1.057831	0.268723	-0.056841
O	0.222737	0.267660	-0.819988
O	-1.362684	-0.858998	0.901134
C	-4.062312	-1.795490	-0.839445
C	-4.034244	-2.087518	-2.217223
C	-3.827283	-2.814217	0.107308
C	-3.840131	-3.442219	-2.651568
C	-4.200916	-1.078399	-3.221845
C	-3.643739	-4.166305	-0.340497
C	-3.723332	-2.551101	1.513797
C	-3.810128	-3.729990	-4.053223
C	-3.674927	-4.450982	-1.705158
C	-4.154747	-1.390913	-4.548075
H	-4.357418	-0.050423	-2.912924
C	-3.392004	-5.193587	0.624005
C	-3.466536	-3.555262	2.400509
H	-3.799563	-1.527079	1.859867
C	-3.956054	-2.738450	-4.975073
H	-3.657097	-4.761677	-4.359979

H	-3.534039	-5.476717	-2.039469
H	-4.272401	-0.608832	-5.291423
H	-3.267917	-6.212764	0.265229
C	-3.302597	-4.900474	1.953016
H	-3.359389	-3.323752	3.454925
H	-3.926663	-2.965192	-6.036276
H	-3.110298	-5.687908	2.676371
C	0.941073	3.423214	0.370739
C	1.926149	2.918481	-0.499264
C	1.248122	3.695304	1.716758
C	3.244327	2.648589	0.006607
C	1.667529	2.656581	-1.884581
C	2.568718	3.426306	2.210901
C	0.283280	4.239423	2.626395
C	4.246599	2.143198	-0.884315
C	3.530089	2.898930	1.348551
C	2.651291	2.195826	-2.706299
H	0.665342	2.815462	-2.266425
C	2.864584	3.695427	3.584069
C	0.606964	4.486926	3.929211
H	-0.716430	4.452770	2.261747
C	3.961468	1.939237	-2.201843
H	5.237424	1.926279	-0.493256
H	4.526737	2.684566	1.728209
H	2.435266	2.002800	-3.752210
H	3.864758	3.472709	3.945998
C	1.917383	4.208505	4.419553
H	-0.138131	4.898803	4.602736
H	4.736043	1.573976	-2.871652
H	2.153626	4.408222	5.460091
H	1.402203	-0.739042	0.001997
O	-2.284851	0.410324	-1.130571
O	-1.238644	1.651589	0.798934
C	-0.476101	-4.659338	-3.369497
C	-0.463174	-5.550453	-2.297160
C	-0.449146	-5.072345	-0.993345
C	-0.429474	-3.693450	-0.766449
C	-0.445271	-2.793590	-1.831166
C	-0.470469	-3.288027	-3.131473
H	-0.501963	-5.034520	-4.387660
H	-0.485406	-6.621120	-2.474035
H	-0.510050	-5.763522	-0.158644
H	-0.377657	-1.725322	-1.656851
H	-0.492136	-2.588696	-3.960514

N	-0.425603	-3.183353	0.565751
H	-0.880031	-2.127492	0.747733
C	0.133353	-3.817581	1.526833
H	0.662411	-4.750567	1.355570
C	-0.018979	-3.339735	2.939872
O	-0.712856	-2.421870	3.279251
O	0.680414	-4.134487	3.756967
C	0.505401	-3.849164	5.147876
H	0.719883	-2.797832	5.344195
H	-0.523370	-4.071505	5.442863
H	1.203465	-4.499326	5.672245
C	2.339643	-1.969161	1.298629
O	1.859777	-1.606742	0.097939
C	2.125709	-1.106566	2.490041
C	2.594210	-1.500696	3.754401
C	1.415956	0.094656	2.396286
C	2.333501	-0.734990	4.882838
H	3.171054	-2.412265	3.865974
C	1.123074	0.841186	3.534122
H	1.096848	0.488068	1.437330
C	1.576631	0.431698	4.781082
H	2.720653	-1.054764	5.846472
H	0.538706	1.749586	3.433121
H	1.357287	1.026110	5.662563
C	2.974367	-3.165706	1.331701
C	3.261056	-3.929960	0.068349
H	3.395729	-3.531146	2.258549
H	3.571651	-4.953326	0.311622
H	2.344966	-4.019296	-0.534801
C	4.304359	-3.295423	-0.830086
H	4.568982	-3.876307	-1.714486
C	4.839490	-2.091572	-0.657722
H	4.569266	-1.497000	0.210423
C	5.739763	-1.414700	-1.647653
H	6.052280	-2.089528	-2.451250
H	5.202349	-0.569496	-2.096970
C	6.992449	-0.850870	-1.001771
F	6.662805	-0.112714	0.094760
F	7.807205	-1.845233	-0.589266
C	7.777013	0.038613	-1.975186
O	8.660631	-0.364534	-2.681911
O	7.289088	1.280173	-1.972262
C	7.905398	2.194961	-2.896758
H	8.989832	2.134839	-2.772988

H	7.665298	1.869594	-3.914006
C	7.364705	3.573900	-2.589390
H	7.623398	3.865495	-1.568311
H	7.796110	4.303490	-3.279866
H	6.276375	3.596609	-2.687084

28

M06-2X SCF energy: -3532.90591258 a.u.

M06-2X enthalpy: -3531.898617 a.u.

M06-2X free energy: -3532.063346 a.u.

M06-2X SCF energy in solution: -3533.89637570 a.u.

M06-2X enthalpy in solution: -3532.889080 a.u.

M06-2X free energy in solution: -3533.053809 a.u.

Cartesian coordinates

ATOM	X	Y	Z
C	3.413764	-2.593518	-5.263227
C	3.171132	-2.262654	-3.954406
C	1.904812	-1.754115	-3.555152
C	0.904690	-1.577055	-4.549816
C	1.181166	-1.941105	-5.893891
C	2.407054	-2.441069	-6.246104
H	4.390257	-2.970699	-5.549843
H	3.953608	-2.374239	-3.211618
C	1.617392	-1.386607	-2.199365
C	-0.358959	-1.057123	-4.173713
H	0.400617	-1.806401	-6.637701
H	2.613187	-2.711692	-7.276575
C	-0.615884	-0.635311	-2.892985
C	0.424914	-0.741894	-1.927762
H	-1.149334	-0.998135	-4.917881
C	2.565435	-1.694362	-1.095617
C	2.999310	-3.031881	-0.810682
C	2.999698	-0.679723	-0.275257
C	2.514343	-4.167777	-1.514081
C	3.927173	-3.239146	0.249121
C	3.918459	-0.861568	0.790360
C	2.957341	-5.428709	-1.204179
H	1.777508	-4.031465	-2.297761
C	4.374760	-4.555562	0.536983
C	4.376507	-2.135104	1.018694
C	3.907312	-5.628419	-0.175213

H	2.570218	-6.283089	-1.750277
H	5.090237	-4.692837	1.343094
H	5.086320	-2.307008	1.824006
H	4.252270	-6.631308	0.054642
P	1.049612	0.907848	0.033869
O	0.772078	2.229188	-0.767144
O	0.839735	0.888912	1.486602
C	4.374606	0.292122	1.616024
C	5.277146	1.222807	1.066190
C	3.931157	0.420090	2.945529
C	5.765403	2.298137	1.881427
C	5.746093	1.135801	-0.284474
C	4.440217	1.491246	3.756305
C	2.969599	-0.468601	3.528770
C	6.685134	3.237616	1.317613
C	5.343192	2.399951	3.207062
C	6.621564	2.053063	-0.786280
H	5.393251	0.323791	-0.912165
C	4.005293	1.603879	5.115100
C	2.575479	-0.322252	4.825020
H	2.534750	-1.246801	2.911804
C	7.100383	3.123568	0.025129
H	7.043577	4.046389	1.948971
H	5.724325	3.210166	3.825299
H	6.960093	1.971165	-1.814327
H	4.407387	2.414653	5.716846
C	3.107592	0.723154	5.637116
H	1.835701	-0.997279	5.243079
H	7.796883	3.843087	-0.393527
H	2.781207	0.818882	6.667950
C	-2.001081	-0.217009	-2.519620
C	-2.381990	1.134754	-2.538949
C	-2.945025	-1.221217	-2.219436
C	-3.741404	1.493645	-2.242618
C	-1.449621	2.184557	-2.826477
C	-4.310698	-0.855777	-1.965104
C	-2.600314	-2.611831	-2.158804
C	-4.101651	2.878984	-2.214939
C	-4.675038	0.490931	-1.983423
C	-1.830365	3.494058	-2.779985
H	-0.421672	1.922841	-3.052370
C	-5.266890	-1.881721	-1.680772
C	-3.541289	-3.558726	-1.871698
H	-1.570297	-2.904162	-2.335108

C	-3.177674	3.849831	-2.471228
H	-5.133813	3.137415	-1.989485
H	-5.708746	0.764838	-1.780059
H	-1.102538	4.276629	-2.968188
H	-6.295284	-1.585695	-1.489970
C	-4.898122	-3.192395	-1.630220
H	-3.256548	-4.604522	-1.815537
H	-3.459262	4.898261	-2.438665
H	-5.623168	-3.960022	-1.383328
H	0.203873	2.915335	-0.313349
O	2.533639	0.601722	-0.516249
O	0.158558	-0.273123	-0.656610
C	-1.901731	4.390043	0.469943
O	-0.709449	4.245773	0.139876
C	-2.490780	5.761959	0.465460
C	-3.869487	5.980767	0.380051
C	-1.621435	6.855783	0.508980
C	-4.371622	7.277924	0.352525
H	-4.552551	5.139981	0.301595
C	-2.125328	8.150748	0.492795
H	-0.554181	6.667053	0.557112
C	-3.500980	8.363598	0.416046
H	-5.441863	7.441391	0.277515
H	-1.446153	8.996057	0.537427
H	-3.894256	9.375232	0.399975
C	-2.697475	3.262014	0.864865
C	-2.113555	1.904595	0.804200
H	-3.701378	3.411970	1.246678
H	-1.632233	1.810057	-0.181870
H	-1.285265	1.824772	1.526325
C	-3.063884	0.756166	0.980040
H	-4.110135	0.923166	0.722761
C	-2.652904	-0.461380	1.324541
H	-1.601208	-0.625515	1.559282
C	-3.548938	-1.662548	1.313102
H	-4.587435	-1.384951	1.107243
H	-3.242537	-2.354213	0.519013
C	-3.512584	-2.433893	2.609219
F	-2.234443	-2.786086	2.917747
F	-3.974127	-1.680804	3.639416
C	-4.336690	-3.731924	2.560030
O	-4.854863	-4.161442	1.560049
O	-4.380173	-4.308975	3.755210
C	-5.103051	-5.549575	3.815443

H	-6.131775	-5.369356	3.489829
H	-4.647948	-6.252149	3.111105
C	-5.030042	-6.040024	5.244250
H	-5.480174	-5.311891	5.923145
H	-5.567688	-6.986892	5.340723
H	-3.990847	-6.196260	5.543072

. References:

[1] Frisch, M. J.; Trucks, G. W.; Schlegel, H. B.; Scuseria, G. E.; Robb, M. A.; Cheeseman, J. R.; Scalmani, G.; Barone, V.; Mennucci, B.; Petersson, G. A.; Nakatsuji, H.; Caricato, M.; Li, X.; Hratchian, H. P.; Izmaylov, A. F.; Bloino, J.; Zheng, G.; Sonnenberg, J. L.; Hada, M.; Ehara, M.; Toyota, K.; Fukuda, R.; Hasegawa, J.; Ishida, M.; Nakajima, T.; Honda, Y.; Kitao, O.; Nakai, H.; Vreven, T.; Montgomery, J. A., Jr.; ; Peralta, J. E.; Ogliaro, F.; Bearpark, M.; Heyd, J. J.; Brothers, E.; Kudin, K. N.; Staroverov, V. N.; Kobayashi, R.; Normand, J.; Raghavachari, K.; Rendell, A.; Burant, J. C.; Iyengar, S. S.; Tomasi, J.; Cossi, M.; Rega, N.; Millam, N. J.; Klene, M.; Knox, J. E.; Cross, J. B.; Bakken, V.; Adamo, C.; Jaramillo, J.; Gomperts, R.; Stratmann, R. E.; Yazyev, O.; Austin, A. J.; Cammi, R.; Pomelli, C.; Ochterski, J. W.; Martin, R. L.; Morokuma, K.; Zakrzewski, V. G.; Voth, G. A.; Salvador, P.; Dannenberg, J. J.; Dapprich, S.; Daniels, A. D.; Farkas, O.; Foresman, J. B.; Ortiz, J. V.; Cioslowski, J.; Fox, D. J., *Gaussian 16*, Revision C.01; Gaussian, Inc.: Wallingford, CT, **2019**

[2] Zhao, Y.; Truhlar, D. G. *Theor. Chem. Acc.* **2008,** *120*, 215-241.

[3] Marenich, A. V.; Cramer, C. J.; Truhlar, D. G. *J. Phys. Chem. B* **2009,** *113*, 6378-6396.

[4] Legault, C. Y. CYLView, 1.0b; Université de Sherbrooke: Canada, 2009, http://www.cylview.org.

[5] Morales-Rivera, C. A.; Floreancig, P. E.; Liu, P. *J. Am. Chem. Soc.* **2017**, *139*, 17935-17944.
