# Supplementary material for: Asymmetric Three-Component Radical Cascade Reactions Enabled by Synergistic Photoredox/Brønsted Acid Catalysis: Access to α-Amino Acid Derivatives
Source: ACS Cent Sci. 2024 Aug 16;11(1):36–45. doi: 10.1021/acscentsci.4c00970 (PMC11758273; doi:10.1021/acscentsci.4c00970)
Supplement: Supplementary file 3 — oc4c00970_si_004.pdf [file oc4c00970_si_004.pdf]

oc-2024-009703.R1

Name: Peer Review Information for "Asymmetric Three-Component Radical Cascade Reactions Enabled by Synergistic Photoredox/Brønsted Acid Catalysis: Access to α-Amino Acid Derivatives"

First Round of Reviewer Comments

Reviewer: 1

Comments to the Author

This manuscript by Wang, Qi, and coworker reported photoredox/brønsted acid co-catalyzed three-component radical cascade reaction. This strategy enables the synthesis of a series of valuable enantioenriched unnatural alpha-amino acid derivatives bearing two contiguous stereogenic centers with high diastereoselectivity and enantioselectivity. Control experiments combined with systematic DFT studies clarified the C-C bond formation mechanism. Instead of the radical-radical coupling pathway, a brand-new C-C coupling mechanism through PCET and Mannich-type reaction was proven to be more favored. Considering the high novelty of the mechanism and the high quality of the experimental results, I think this work will be of great interest to other chemistry researchers. I would like to recommend the publication of this work in ACS Central Science after minor revisions. The following suggestions need to be addressed:

1. Considering racemic 2 was used in this asymmetric three-component radical cascade reaction, this reviewer is curious about whether kinetic resolution occurred in this process, which may be caused by the potential effect of chiral phosphoric acid. The authors are suggested to interrupt the reaction and recover the unreacted compound 2 to check the ee value;

2. For the competition radical coupling of the initially-formed radical species to the in-situ formed α-amino radical, the authors gave an explanation on the chemoselectivity in the description related to Scheme 5 : "…….the direct radical addition of strongly-electrophilic radical D to the cationic iminium intermediate C is also not occurring probably due to a strong polarity mismatching, which explains the excellent chemoselectivity for this challenging radical relay cascade reaction". If this is the case, the authors are suggested to investigate other bromo-containing compounds (e.g. methyl 2-bromo-2-methylpropanoate), which can produce less electrophilic radical species and thus may give rise to two component reaction without the participation of VCP. The corresponding experimental results will help to further validate the mechanism of chemoselectivity.

3. The computational studies were well-performed. In Figure 1c, the key transition states TS-4 and TS-5 are labeled with keywords, like "proton-coupled electron transfer" and "Mannich-type reaction", which can enhance the readability of this manuscript. The analogous strategy should be applied to Figure 1a and 1b.

Reviewer: 2

Comments to the Author

Wang and his co-workers describe a conceptually novel catalytic asymmetric three-component radical cascade reaction of readily accessible glycine esters, α-bromo carbonyl compounds and 2-vinyl-cyclopropyl ketones via synergistic photoredox/Brønsted acid catalysis. This novel protocol provides a facile buildup of enantioenriched □-amino acid derivatives with molecular complexity. Meanwhile, a unique PCET-promoted radical-radical coupling is supported by mechanistic investigations and quantum mechanics calculations.

Thus, I recommend that this manuscript is accepted and published in ACS Central Science after minor revision.

To improve this manuscript, please address the following points.

1) Page 5 right column: Please check the number of the radical-trapping adduct which was detected by HMRS analysis [Eq. (1)].

2) Please add the control experiments to further demonstrate the relation between the product yield and the configuration of the 2-vinylcyclopropyl ketone.

3) Some relative literatures should be cited,such as, a) ACS Catal. 2022, 12, 12984–12992; b) J. Am. Chem. Soc. 2024, 146, 13347–13355; c) Angew. Chem. Int. Ed. 2023, 62, e202217887. D) Chem. Sci., 2022,13, 8576-8582;e)CCS Chem. 2024, 10.31635/ccschem.024.202404257.

Reviewer: 3

Comments to the Author

Wang and co-workers describe in this manuscript three-component radical cascade reaction by CPA/PC catalysis. alpha-Amino acid derivatives, which are important substructure, were obtained in good yields and with high to excellent enantioselectivities. Vicinal stereogenic center including quaternary carbon stereocenters were also constructed highly stereoselectively. Mechanistic studies and theoretical study elucidated the mechanism. This is a nice piece of work and this reviewer recommends publication of the manuscript in ACS Central Science after addressing following issues.

(1) One of the weak points of this manuscript is moderate yields of the products. What are the major byproducts?

(2) Compounds 2 and 5 are racemic. This reviewer is wondering if kinetic resolution is operative in the ring opening reaction in the presence of CPA. According to the proposed reaction mechanism, kinetic resolution is not operative, but it is curious to know.

(3) Following review article should be cited.

Liu, G.; Cao, Y. Asymmetric Catalytic Synthesis by Synergistic Chiral Phosphoric Acid Photoredox Catalyzed Reactions. Adv. Synth. Catal. 2023, 365, 3044-3062.

(4) There are grammatical error and typo.

Page 4, right column, line 2, please change moderated to moderate.

Page 7, left column, line 12, minishing should read minimizing?

Author's Response to Peer Review Comments:

Dear Prof. Editor,

Enclosed please find our revised manuscript entitled "**Asymmetric Three-Component Radical Cascade Reactions Enabled by Synergistic Photoredox/Brønsted Acid Catalysis: Access to α-Amino Acid Derivatives**" (manuscript ID: oc-2024-009703). We revised our manuscript and supporting information carefully according to the suggestions from the reviewers, and all the issues raised by the referees are now fully addressed. Please find out our response to all questions point-by-point as follows:

For Reviewer 1

Recommendation: Publish in ACS Central Science after minor revisions noted

Q1: *Considering racemic 2 was used in this asymmetric three-component radical cascade reaction, this reviewer is curious about whether kinetic resolution occurred in this process, which may be caused by the potential effect of chiral phosphoric acid. The authors are suggested to interrupt the reaction and recover the unreacted compound 2 to check the ee value*

A1: Thank you very much for your kind suggestion. As suggested, the corresponding control experiment has been performed with racemic *trans*-**2a**. When the reaction was interrupted under the standard reaction conditions within 4 h, the recovered **2a** was observed as racemate, which demonstrates no kinetic resolution of racemic *trans*-**2a** in this cascade transformation. The related experimental results have been added in the revised manuscript.

Q2: *For the competition radical coupling of the initially-formed radical species to the in-situ formed α-amino radical, the authors gave an explanation on the chemoselectivity in the description related to Scheme 5 : "…….the direct radical addition of strongly-electrophilic radical D to the cationic iminium intermediate C is also not occurring probably due to a strong polarity mismatching, which explains the excellent chemoselectivity for this challenging radical relay cascade reaction". If this is the case, the authors are suggested to investigate other bromo-containing compounds (e.g. methyl 2-bromo-2-methylpropanoate), which can produce less electrophilic radical species and thus may give rise to two component reaction without the participation of VCP. The corresponding experimental results will help to further validate the*

mechanism of chemoselectivity.

A2: Thank you very much for your kind suggestion. As suggested, control experiment has been performed with methyl 2-bromo-2-methylpropanoate to further validate the mechanism of chemoselectivity, and the corresponding experimental results have been documented in the revised manuscript as below:

When methyl 2-bromo-2-methylpropanoate was employed under the standard reaction conditions, two component radical addition reaction occurred without the participation of VCP (**2a**) since the corresponding tertiary radical generated from methyl 2-bromo-2-methylpropanoate is less electrophilic (see Supporting Information for details).

Q3: *The computational studies were well-performed. In Figure 1c, the key transition states TS-4 and TS-5 are labeled with keywords, like "proton-coupled electron transfer" and "Mannich-type reaction", which can enhance the readability of this manuscript. The analogous strategy should be applied to Figure 1a and 1b.*

A3: Thank you very much for your kind suggestion. As suggested, we have added keywords to Figure 1a and 1b.

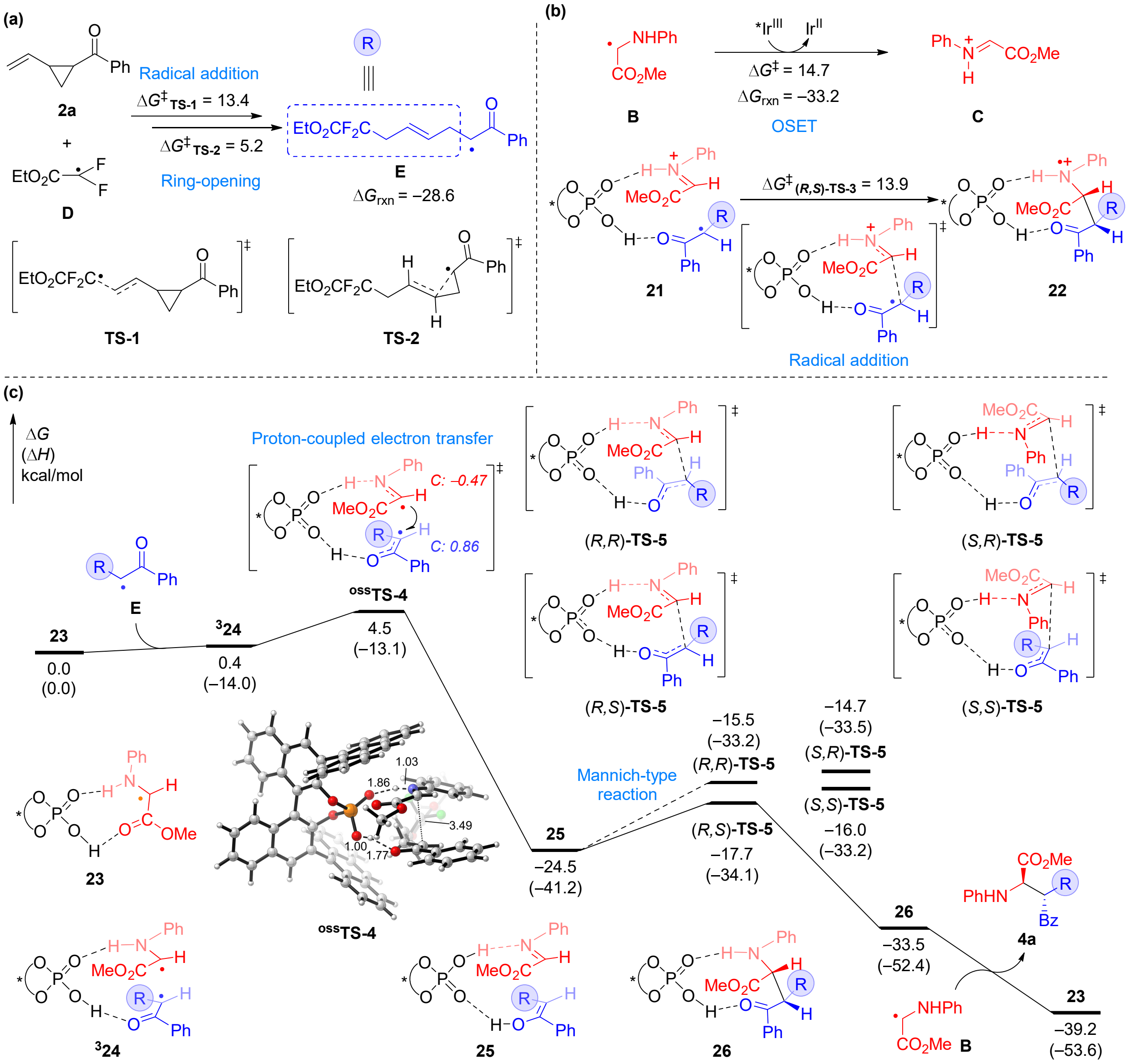

Figure 1. Computational studies of the three-component radical cascade reaction mechanism. (a) DFT study of the formation of α-carbonyl radical. (b) DFT study of the radical/iminium ion coupling pathway through radical addition. (c) Free energy profile of the radical-radical coupling pathway. The chiral phosphoric acid (*R*)-**C1** is used in DFT calculation. The italic numbers in **ossTS-4** denote the Mulliken atomic spin densities on α-carbonyl carbon and α-amino carbon. The bond lengths are in angstrom and energies are in kcal/mol. All energies were calculated at M06-2X/6-311+G(d,p)-SDD/SMD(acetonitrile)//M06-2X/6-31G(d) level of theory. See SI for computational details.

For Reviewer 2

Recommendation: Publish in ACS Central Science after minor revisions noted.

Q1: *Page 5 right column: Please check the number of the radical-trapping adduct which was detected by HMRS analysis [Eq. (1)].*

A1: Thank you very much for your kind reminder. We corrected the mistake in the revised manuscript.

Q2: *Please add the control experiments to further demonstrate the relation between the product yield and the configuration of the 2-vinylcyclopropyl ketone.*

A2: Thank you very much for your kind suggestion. As suggested, the control experiments have been performed with two enantioenriched (1*S*,2*R*)-**2a** and (1*R*,2*S*)-**2a**, and the related experimental results have been added in the revised manuscript as below:

The performance of two enantioenriched (1*S*,2*R*)-**2a** and (1*R*,2*S*)-**2a** was also investigated in this cascade transformation, and the same reactivities were observed with maintained stereoselectivity control, which further confirmed no kinetic resolution occurred in this three-component radical cascade reaction.

Q3: *Some relative literatures should be cited, such as, a) ACS Catal. 2022, 12, 12984–12992; b) J. Am. Chem. Soc. 2024, 146, 13347–13355; c) Angew. Chem. Int. Ed. 2023, 62, e202217887. D) Chem. Sci., 2022,13, 8576-8582; e) CCS Chem. 2024, 10.31635/ccschem.024.202404257.*

A3: Thank you very much for your kind suggestion. The mentioned literature has been cited as ref. 4e-4i.

For Reviewer 3

Recommendation: Publish in ACS Central Science after minor revisions noted.

Q1: *One of the weak points of this manuscript is moderate yields of the products. What are the major byproducts?*

A1: Thank you so much for your time to review our work and comments. The major byproducts are compound **I** (generated from the homocoupling of α-amino radical) and **II** (generated from the radical addition between **3** and **2**), which has been supplemented in the revised Supporting Information. For your convenience, the corresponding equation has been copied as below:

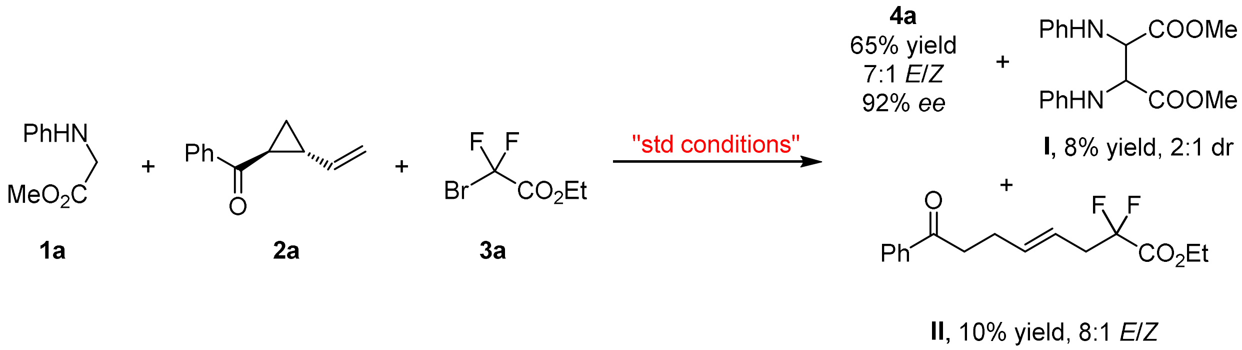

Q2: *Compounds 2 and 5 are racemic. This reviewer is wondering if kinetic resolution is operative in the ring opening reaction in the presence of CPA. According to the proposed reaction mechanism, kinetic resolution is not operative, but it is curious to know.*

A2: Thank you very much for your kind suggestion. We conducted the mentioned control experiments with racemic compounds **2a** and **5**. When the reactions were interrupted under the standard reaction conditions within 4 h, the recovered **2a** and **5** were determined to be racemate, which demonstrates no kinetic resolution in this cascade transformation. The corresponding experimental results have been supplemented in the revised manuscript.

Q3: *Following review article should be cited. Liu, G.; Cao, Y. Asymmetric Catalytic Synthesis by Synergistic Chiral Phosphoric Acid Photoredox Catalyzed Reactions. Adv. Synth. Catal. 2023, 365, 3044-3062..*

A3: Thank you very much for your kind suggestion. The mentioned literature has been cited as ref. 9f.

Q4: *There are grammatical error and typo. Page 4, right column, line 2, please change moderated to moderate. Page 7, left column, line 12, minishing should read minimizing?*

A4: Thank you very much for your kind reminder. The mistakes have been corrected in the revised manuscript. The word “minishing” on page 7 was revised to “diminishing”.

For Formatting Needs:

Q1: AU EMAIL: Please include the email address of the corresponding author on the first page of the manuscript, and the Supporting Information if submitted, with an asterisk next to their name in the author list. Please be sure to label “email.”

A1: Thank you very much for your kind reminder. The email addresses of the corresponding authors have already been included on the first page of the manuscript, and the Supporting Information.

Q2: SYNOPSIS MISSING: The synopsis should be no more than 200 characters (including spaces) and should reasonably correlate with the TOC graphic. The synopsis is intended to explain the importance of the article to a broader readership across the sciences. Please place your synopsis in the manuscript file after the TOC graphic, and label it as “Synopsis.”

A2: Thank you very much for your kind reminder. The synopsis has been placed in the revised manuscript file after the TOC graphic, and label it as “Synopsis.”

We have taken into consideration all the reviewers’ comments and constructive criticisms. Finally, we would like to extend our thanks to all the reviewers for their helpful suggestions and comments. We look forward to hearing from you soon.

Best regards,

Chun-Jiang Wang

Professor of Chemistry

CCMS, Wuhan University

oc-2024-009703.R2

Name: Peer Review Information for "Asymmetric Three-Component Radical Cascade Reactions Enabled by Synergistic Photoredox/Brønsted Acid Catalysis: Access to α-Amino Acid Derivatives"

Second Round of Reviewer Comments

Reviewer: 1

Comments to the Author

The authors have addressed my questions properly. I would like to recommend the publication of this manuscript in ACS Central Science.

Reviewer: 2

Comments to the Author

Th revised manuscript has been improved according to referee reports.

Author's Response to Peer Review Comments:

Dear Prof. Editor,

Enclosed please find our revised manuscript entitled “**Asymmetric Three-Component Radical Cascade Reactions Enabled by Synergistic Photoredox/Brønsted Acid Catalysis: Access to α-Amino Acid Derivatives**” (manuscript ID: oc-2024-009703). We revised our manuscript and supporting information carefully according to the Formatting Needs from your office.

For Formatting Needs:

Q: AU EMAIL: Please include the email address of the corresponding author on the first page of the manuscript, and the Supporting Information if submitted, with an asterisk next to their name in the author list. Please be sure to label “email.”

A: Thank you very much for your kind reminder. The email addresses of the corresponding authors have already been included on the first page of the manuscript, and the Supporting Information.

We have taken into consideration all the reviewers’ comments and constructive criticisms. Finally, we would like to extend our thanks to all the reviewers and your office for the helpful suggestions and comments. We look forward to hearing from you soon.

Best regards,

Chun-Jiang Wang

Professor of Chemistry

CCMS, Wuhan University
